# MicroRNA expression profiling of goat peripheral blood mononuclear cells in response to peste des petits ruminants virus infection

**DOI:** 10.1186/s13567-018-0565-3

**Published:** 2018-07-16

**Authors:** Xuefeng Qi, Ting Wang, Qinghong Xue, Zhen Li, Bo Yang, Jingyu Wang

**Affiliations:** 1 College of Veterinary Medicine, Northwest A&F University, Yangling, 712100 Shaanxi China; 2 China Institute of Veterinary Drug Control, Beijing, 100000 China

## Abstract

Peste des petits ruminants virus (PPRV) belongs to the genus *Morbillivirus* that causes an acute and highly contagious disease in goats and sheep. Virus infection can trigger the change in the cellular microRNA (miRNA) expression profile, which play important post-transcriptional regulatory roles in gene expression and can greatly influence viral replication and pathogenesis. Here, we employed deep sequencing technology to determine cellular miRNA expression profile in goat peripheral blood mononuclear cells (PBMC) infected with Nigeria 75/1 vaccine virus, a widely used vaccine strain for mass vaccination programs against Peste des petits ruminants. Expression analysis demonstrated that PPRV infection can elicit 316 significantly differentially expressed (DE) miRNA including 103 known and 213 novel miRNA candidates in infected PBMC at 24 hours post-infection (hpi) as compared with a mock control. Target prediction and functional analysis of these DEmiRNA revealed significant enrichment for several signaling pathways including TLR signaling pathways, PI3K-Akt, endocytosis, viral carcinogenesis, and JAK-STAT signaling pathways. This study provides a valuable basis for further investigation of the roles of miRNA in PPRV replication and pathogenesis.

**Electronic supplementary material:**

The online version of this article (10.1186/s13567-018-0565-3) contains supplementary material, which is available to authorized users.

## Introduction

Peste des petits ruminants (PPR) is an acute, highly contagious fatal disease in domestic and small wild ruminants, causing great economic losses in goat and sheep productivity [1]. Peste des petits ruminants virus (PPRV), the causative agent of the disease, belongs to the genus *Morbillivirus* within the family *Paramyxoviridae* [2]. Given its economic relevance and severity, PPR is classified as a world organization for animal health (OIE) listed disease [3, 4]. Based on phylogenetic analysis of partial “N” and “F” gene sequences, PPRV can be divided into four different lineages [3–5]. Live attenuated vaccines have already been used for control of PPR and showed a good immunological effect on both sheep and goats [6, 7]. Among these live attenuated vaccines, Nigeria 75/1 and Sungri/96 have been demonstrated to be safe and efficacious in conferring protection to sheep and goats against PPRV infection [7, 8]. PPRV is both lymphotropic and epitheliotropic in nature [9]. PPRV enters lymphoid cells through the signaling lymphocyte activation molecule (SLAM), which is widely expressed on the surface of all immune cells [9]. Peripheral blood mononuclear cells (PBMC) are widely used as a standard in vitro model to study host-PPRV interactions as in other *morbillivirus* infections [10–13]. Recently, transcriptome analysis of PBMC infected with PPRV uncovered transcription factors modulating immune responses [13, 14]. However, our understanding about the role of cellular microRNA (miRNA) during PPRV infection is still obscure.

MicroRNA are highly conserved small non-coding RNA of 19–24 nucleotides in length and play a critical role in many physiological and pathological processes such as development, proliferation, differentiation, apoptosis, immune response, and tumorigenesis in animals and plants [15–17]. Recently, a substantial number of research has implicated the role of miRNA in viral replication and has indicated they can inhibit or promote viral infection [18, 19]. Virus infection can trigger the changes in the cellular miRNA profile, which can greatly influence viral life cycles, viral tropism, and the pathogenesis of viral diseases [20–23].

In the current study, to explore the importance of miRNA regulation in PPRV infection, a deep sequencing approach was employed to identify differentially expressed miRNA in goat PBMC infected with PPRV Nigeria 75/1 vaccine virus. A bioinformatics analysis demonstrates that the differentially expressed miRNA play a crucial role in the PPRV-host cell interactions. The data suggest that host miRNA might have significant roles in modulating PPRV replication and pathogenesis.

## Materials and methods

### Ethics statement and experimental animals

The animal experiments were carried out in strict accordance with guidelines established by the Ethics of Animal Experiments of Northwest A&F University, Yangling, China. All the protocols were approved by this committee (Permit Number: 2014BAD23B11). Healthy 6-month-old goats used for blood collection were housed in appropriate containment facilities and had ad libitum access to feed and water. Goats were screened and shown negative for PPRV antibodies using competitive ELISA as well as a virus neutralization test.

### PBMC isolation and virus infection

Goat PBMC were isolated using Histopaque-1077 (Sigma, USA) by density gradient centrifugation following the manufacturer’s instructions. Then, isolated cells from each goat were suspended into 70 mL RPMI-1640 medium (Hyclone, Logan, UT, USA) supplemented with 10% fetal calf serum (FCS), 2% l-glutamine, 100 mg/mL penicillin, and 100 IU/mL streptomycin. The PPRV vaccine strain, Nigeria 75/1, was obtained from the Lanzhou Veterinary Research Institute, Chinese Academy of Agricultural Sciences (Lanzhou, China). Virus stock was prepared by collecting the infected cell supernatant when cytopathic effect (CPE) affected about 80% of the cells. The virus was harvested by three cycles of freezing–thawing and stored at −80 °C and purified by banding on sucrose gradient. The purified virus titers were estimated by calculating 50% tissue culture infective doses (TCID_50_) using vero cells in 96 well microtitre plates. The purified virus was tested for its infectivity in vero cells and was used further for infection in goat PBMC.

For virus infection, goat PBMC were seeded into six well plates at a density of 1 × 10^5^ cells/mL and were inoculated with Nigeria 75/1 at a multiplicity of infection (MOI) of 1.0. After 1 h of adsorption, infected cells were maintained in RPMI-1640 medium (Hyclone, Logan, UT, USA) containing 2% FCS, 2% l-glutamine and antibiotics. PBMC cells inoculated with similarly purified preparation from triple freezing–thawing vero cells were used as the mock-infected group. Viral infection in PBMC was confirmed with CPE and western blot. The CPE were observed under a light microscope at 0, 24, 48, and 72 hours post-inoculation (hpi). Western blot was performed using a polyclonal antibody against PPRV N protein to determine virus replication at the different time points. Three independent biological replicates of PPRV- and mock-inoculated cultures were prepared at each time point.

### Western blot analysis

Protein homogenates from goat PBMC were extracted as previously described [24]. Briefly, the cells were lysed for 20 min on ice in ice-cold lysis buffer (4906837001, Roche). The lysates were centrifuged at 12 000 ×* g* for 20 min at 4 °C to obtain a clear lysate. The protein content of each sample was determined using the BCA Protein Assay Kit (Thermo Scientific). Then, equal amounts of protein were separated on a 12% SDS–polyacrylamide gel and transferred to polyvinylidene difluoride membranes. Membranes were probed overnight at 4 °C with anti-PPRV-N monoclonal antibody provided by China Animal Health and Epidemiology Center (Qingdao, China). All membranes were also probed with anti-β-actin antibody (MA1-744, Invitrogen). The bands were visualized using HRP-conjugated goat anti-mouse IgG (1:15 000, Boster) prior to the ECL protocol (Amersham Biosciences, Piscataway, NJ, USA). Changes in protein expression were determined after normalizing the band intensity of each lane to that of β-actin. Signal was visualized using the Konica SRX 101A developer (Konica Minolta Medical Imaging, Wayne, NJ, USA) and the Quantity One software (Bio-Rad, Mississauga, ON, Canada) was used for densitometrical analysis.

### RNA isolation and small RNA library construction

PPRV-infected (*n* = 3) and mock-infected (*n* = 3) goat PBMC in six-well cell culture plates (6 × 10^5^ cells/plate) were harvested at 24 hpi and washed three times with ice-cold phosphate buffered saline (PBS). Total RNA from PPRV-infected to mock-infected cells at 24 hpi was extracted using TRIzol reagent (Invitrogen, Carlsbad, CA, USA) according to the manufacturer’s instructions. The total RNA quantity and concentration of both samples were measured with an Agilent 2100 Bioanalyzer (Agilent Technologies, Santa Clara, CA, USA) and the NanoDrop ND-2000 spectrophotometer (Thermo Fisher Scientific, Waltham, MA, USA). An RNA integrity number score ≥ 8 and rRNA 28S/18S ≥ 1.6 were required in the study. Two groups of total RNA were used for library preparation and sequencing by pooling equal quantities (10 µg) of total RNA isolated from three individual PPRV-infected and mock-infected goat PBMC. Briefly, total RNA were purified by polyacrylamide gel electrophoresis (PAGE) to enrich 15–35 nt molecules, then proprietary adapters were ligated to the 5′ and 3′ terminal of the RNA and the samples were used as templates for cDNA synthesis. The cDNA was amplified using the appropriate number of PCR cycles to produce sequencing libraries, which were subsequently subjected to the proprietary Solexa sequencing-by-synthesis method using the Illumina Genome Analyzer (SanDiego, CA, USA). Sequencing was carried out at the BGI Bio-tech Inc. (Guangzhou, China).

### RNA sequencing and data processing

The various steps followed in the present study are summarized in Additional file 1. Raw sequencing reads from each library were first cleaned by removing the low-quality tags and adapter sequences. The reads between 18 and 30 nt were screened against the GenBank database and Rfam (10.1) database [25] to remove rRNA, tRNA, small nucleolar RNA, small nuclear RNA, repeats, exon and intron sequences. The clean reads were mapped to the capra hircus genome [26], and their expression and distribution patterns were analyzed using the Short Oligonucleotide Alignment Program (SOAP) [27]. The sRNA were aligned to the miRNA precursors of the reference species (Capra hircus) in the Sanger miRBase 21.0 database to identify known miRNA, as well as the base bias for the first position of the identified miRNA with a certain length and for each position of all of the identified miRNA. By comparing our sequences with those in the databases, sRNA can be annotated into different categories. After excluding sRNA that were identified by the above categories, the unannotated short reads were subjected to novel miRNA prediction using the miReap program. Differential expression analysis between PPRV-infected and mock-infected cells of the two samples was performed using the DEGseq R package. A *P* value < 0.01 and a │log2 (fold change) │ > 1 were set as the default thresholds for significant differential expression.

### miRNA target identification and gene ontology analysis

The target genes for each differentially expressed miRNA were predicted using the miRanda and RNAhybrid algorithms. The predicted target genes were then subjected to gene ontology (GO) functional analysis utilizing the WEGO program to create histograms of GO annotation against cell components, biological processes, and molecular functions [28]. To further explore the biological function of the predicted target genes, a Kyoto Encyclopedia of Genes Genomes (KEGG) pathways enrichment analysis was performed using the KOBAS 2.0 annotation tool.

### Quantitative real-time RT-PCR

To validate the sequencing data, we randomly selected nine differentially expressed miRNA for quantitative real-time RT-PCR analysis using Bulge-loop™ miRNA qRT-PCR Primer Sets (one RT primer and a pair of qPCR primers for each set) and performed on ABI 7500 System (Applied Biosystems, Warrington, UK). The primers for selected miRNA and internal standard 5S snRNA are designed by RiboBio Inc. (GuangZhou. China) and the sequences are covered by a patent. Briefly, 10 µg of cDNA was added to 10 µL of the 2 × SYBR green PCR master mix with 0.2 µL of Taq polymerase enzyme (200 U/µL, RiboBio, China), 200 nM of each primer and ddH_2_O to a final volume of 20 µL. The reactions were amplified for 10 s at 95 °C and 20 s at 60 °C for 40 cycles. The thermal denaturation protocol was run at the end of the PCR to determine the number of products that were presented in the reaction mix. Reactions were typically run in duplicate. The gene 5S was used as the internal reference gene. The relative expression level of each miRNA was calculated using the 2^−ΔΔCt^ method [29].

## Results

### Characterization of PPRV replication in goat PBMC

**Figure 1:**
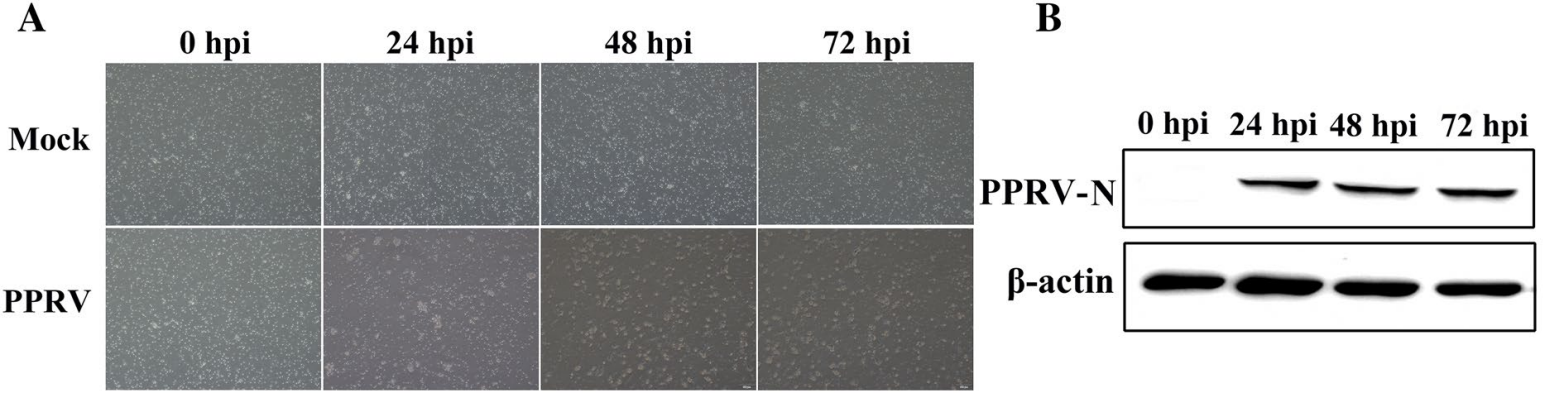
**Characterization of PPRV infection in goat PBMC. A** Morphological changes in goat PBMC at different time points after PPRV infection (MOI = 1), with mock-infected cells as a control. **B** Western blot analysis of N protein in PPRV-infected and mock-infected goat PBMC. Equal amounts of protein from PPRV to mock-infected cells were separated using SDS-PAGE and transferred to PVDF membranes. The membranes were probed with N antibody. β-actin was used as the internal reference.

To determine the kinetics of PPRV replication in goat PBMC, CPE and viral N protein expression were all detected at 0, 24, 48 and 72 hpi. Compared to mock-infected cells, CPE of the PPRV-infected cells exhibited ballooning and clumping at 24 hpi (Figure 1A). From 48 to 72 hpi, almost all the PPRV-infected cells show swelling or granular degeneration as presented in the Figure 1A. Western blot analysis revealed that significant expression levels of N protein were detected as early as 24 hpi in PPRV-infected goat PBMC (Figure 1B). In general, no excessive CPE but with high virus levels is regarded as the optimal time for miRNA expression analysis in host cells during early stage virus infection, because it may trigger global and strong antiviral immune responses [18, 20, 22]. Here PPRV- and mock-infected cells were harvested at 24 hpi in triplicates for further miRNA sequencing analysis to guarantee a higher proportion of infected cells and to avoid excessive CPE.

### Construction and sequencing data analysis of small RNA libraries

**Figure 2:**
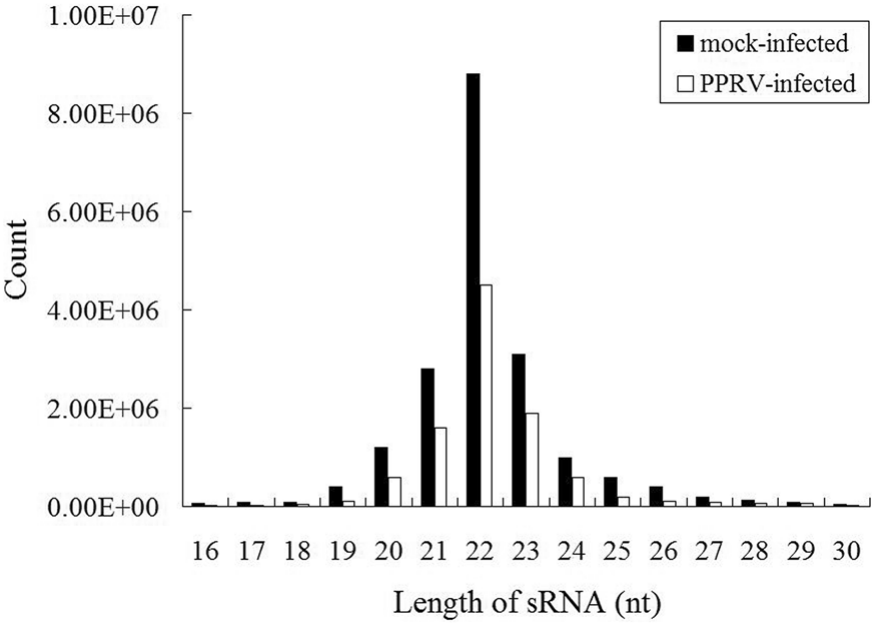
**Length distribution of the clean reads of the sequences.** The abundances of the sequences in the peaks are shown at 22 nt. The majority of miRNA in PPRV-infected and mock-infected goat PBMC were at 21–23 nt.

To identify miRNA changes in goat PBMC infected with PPRV, two small RNA libraries pooled from mock-infected to PPRV-infected groups were constructed at 24 hpi. We performed high-throughput small RNA sequencing on small RNA libraries obtained from mock- to PPRV-infected goat PBMC. In total, 33 933 810 and 33 852 635 raw reads were obtained from the uninfected and infected groups, respectively (Additional file 2). After removing low-quality sequences, adapter sequences, and sequences smaller than 18 nt, 30 573 869 and 30 644 798 clean reads were identified in the mock-infected and PPRV-infected groups, respectively. A total of 29 487 110 (mock-infected) and 26 296 606 (PPRV-infected) clean reads were perfectly mapped to the goat hircus genome [26, 30]. All of the clean reads were annotated and classified as snRNA, rRNA, snoRNA, Rfam other sncRNA, precursor miRNA, mature miRNA, intergenic, intron, exon, and repeats (Additional file 2). Data show that the length distributions of the small RNA from both libraries were 21–23 nt (Figure 2), which was consistent with the typical size of mature miRNA from Dicer-derived products [31]. These results indicate that miRNA have been enriched successively from both libraries.

### Different expression analysis of miRNA

**Figure 3:**
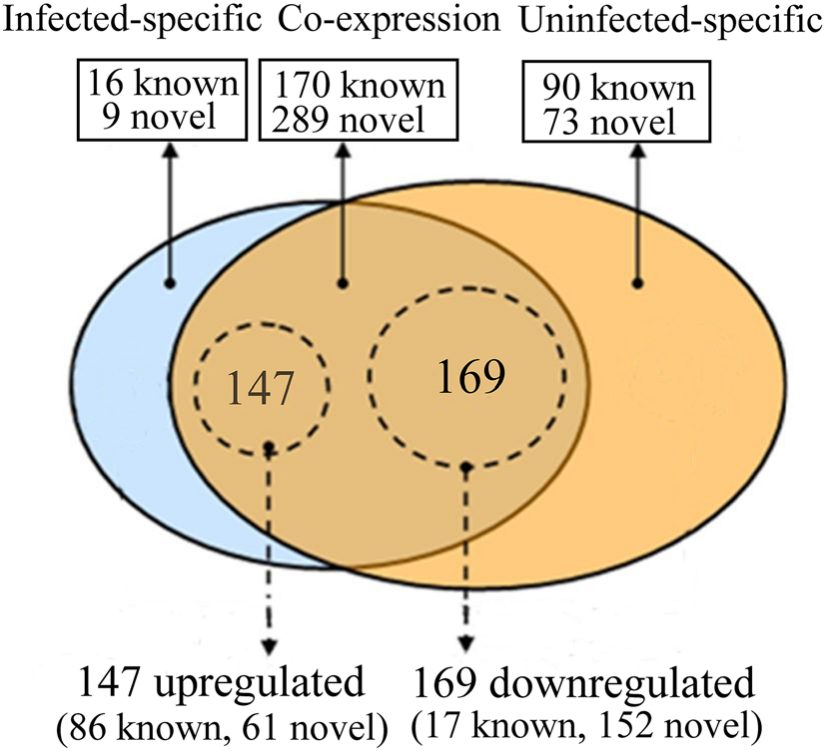
**Comparison of differentially expressed miRNA between the PPRV-infected and mock-infected goat PBMC.** The venn diagram displays the distribution of 647 unique miRNA across the uninfected and infected groups. The dashed circles indicate the miRNA that were significantly differentially expressed in PPRV-infected group compare to the mock-infected group.

To identify known miRNA in goat PBMC, we aligned the small RNA from our libraries to the known mature miRNA and their precursors of the reference species (Capra hircus) in the Sanger miRBase 21.0 database using BLASTN searches to obtain the miRNA count as well as the base bias at the first position. As shown in Figure 3, 459 of 647 unique miRNA genes were co-expressed in both libraries, and 25 and 163 of these miRNA appeared to be preferentially expressed in the PPRV-infected and mock-infected PBMC cell libraries, respectively. Using a *P*-value < 0.01 and a│log2 (fold change)│ > 1 as the cut-off values, a total of 316 miRNA (including 103 known miRNA and 213 novel miRNA) were differentially expressed in the two groups (Additional file 3). Among these 316 DEmiRNA, 147 miRNA were upregulated and 169 miRNA were downregulated in the PPRV-infected cells compared to the mock-infected cells (Figure 3 and Additional file 3).

### Target gene prediction for miRNA

To understand the molecular function and biological processes of miRNA during PPRV infection, two independent algorithms, miRanda and RNAhybrid, were used to predict the mRNA targets for each of the miRNA that was differentially expressed. 12 065 target genes for 103 known miRNA and 213 novel miRNA differentially expressed in the mock-infected and PPRV-infected groups were predicted (Additional file 4). Among these 316 differentially expressed (DE) miRNA co-expressed in the two groups identified, 15 miRNA were identified based on fold change values, and the role of their putative target genes in pro viral and antiviral response was identified (Table 1). Among these 15 DEmiRNA identified, ten miRNA were found to be upregulated while another five miRNA were downregulated in PPRV-infected cells. Particularly, most of the genes targeted by these DEmiRNA have profound immune evasive functions.

**Table 1:** **Candidate target genes for differentially expressed microRNA (miRNA) in PPRV- versus mock-infected goat PBMC**

miRNA name	Up down regulation	*P* value	Target gene
chi-miR-1	DOWN	0	*TLR4*, *CD84*, *LOC108635363*, *LOC102182927*, *JKAMP*, *TNFRSF9*, *IL4R*, *TAL1*, *BMF*, *TRAF3*, *IFNGR1*, *IL2RG*, *TRAF5*, *HSPA13*, *IGSF10*
chi-miR-143-3p	DOWN	1.86E−290	*NECTIN4*, *IKBKG*, *IL4R*
chi-miR-323a-3p	UP	6.59E−241	*IRF3*
chi-miR-485-5p	UP	1.06E−45	*NECTIN4*, *LOC102182927*, *CSF2RB*
chi-miR-1291	UP	6.33E−259	*TMEM173*, *IFNAR1*, *TRADD*, *TCP11L1*, *CDON*
novel_mir3	DOWN	0	*SLAMF6*, *TMEM173*, *CCAR2*, *NECTIN4*, *PBX1*, *LOC108637844*, *IL17B*, *TRIL*, *GDF2*, *NFATC3*, *CD6*, *LOC106503960*, *BCL11A*, *LOC108635335*, *APAF1*, *IFRD1*, *ISLR2*, *TLR4*, *IL1RAP*, *HSPA2*, *IRF2BPL*, *NFKB2*, *IRAK1*
novel_mir85	UP	0	*IL1RAP*, *STAM2*, *TNFSF13B*, *TLR9*
novel_mir218	DOWN	9.37E−14	*IL23A*, *TAL2*, *INSIG1*, *CD82, SLAM*
chi-miR-150	UP	0	*LOC102172159*, *SLC13A4*, *IGFLR1*, *TLR6*, *RTP1*, *ITGB1*
novel_mir70	UP	0	*TLR4*, *JAM2*, *IL1RAP*, *HSPA2*, *MTOR*, *ITGAV*, *STAM2*, *TNFSF13B*, *TLR9*
chi-miR-30b-3p	DOWN	4.26E−48	*SRP19*, *IL22RA1*, *SOCS5*, *LOC102170232*, *LOC102178826*, *TMEM173*, *AREL1*, *IL18BP*, *IGF2BP3*
novel_mir330	DOWN	1.09E−10	*PBXIP1*, *SOCS2*, *ACKR1*, *TNIK*, *CD244, NCR1*, *LOC102182927*, *CASP8*, *ATG9A*, *TNIK*
chi-miR-671-5p	UP	8.28E−63	*CADM2*, *APAF1*, *TMEM173*, *IGF2*
chi-miR-182	UP	8.31E−304	*IL10*, *TNFRSF18*, *BCL11B*, *LOC106503930*, *BAX*
chi-miR-155-3p	UP	4.01E−197	*PBXIP1*, *NFKBIA*

### Gene ontology and KEGG pathway analysis of target genes

**Figure 4:**
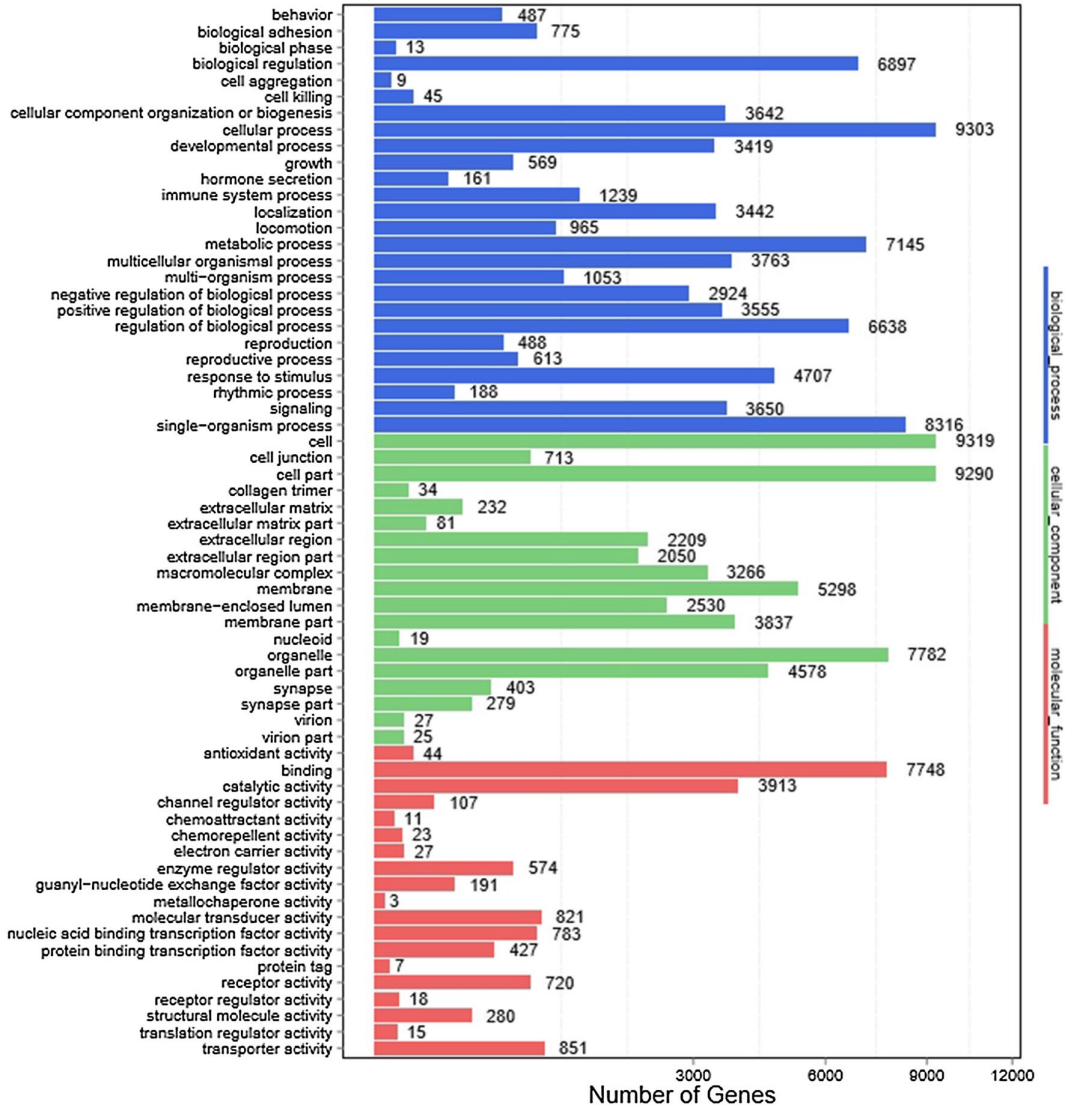
**GO functional classification of all differentially expressed genes (DEG) predicated from the known miRNA.** The GO distribution of the DEG in the PPRV-infected goat PBMC versus mock-infected were classified into three categories: biological process (26 subcategories), cellular components (19 subcategories), and molecular function (19 subcategories).

WEGO analysis shows that a total of 12 065 target genes were successfully annotated for 103 known miRNA and 213 novel miRNA differentially expressed in two groups. The annotated target genes were mainly involved in immune system processes, response to stimulus, metabolic process, binding, organelle and cell processes (Figure 4, Additional file 5). To analyze the roles that these miRNA might play in regulatory networks, we also assigned the putative miRNA targets to the Kyoto Encyclopedia of Genes and Genomes (KEGG) pathways using the KEGG Orthology Based Annotation System (KOBAS). KEGG pathway annotation revealed that 10 364 background genes were annotated for 317 biological processes such as metabolic pathways (ko01100), MAPK signaling pathway (ko04010), PI3K-Akt signaling pathway (ko04151), endocytosis (ko04144), Hippo signaling pathway (ko04390), NF-κB signaling pathway (ko04064), viral carcinogenesis (ko05203), FoxO signaling pathway (ko04068), Jak-STAT signaling pathway (ko04630) and Toll-like receptor signaling pathway (ko04620) (Additional file 6). The results indicate that the differentially expressed miRNA may play a crucial role in the virus-host interactions.

### Validation of deep sequencing results by quantitative RT-PCR

**Figure 5:**
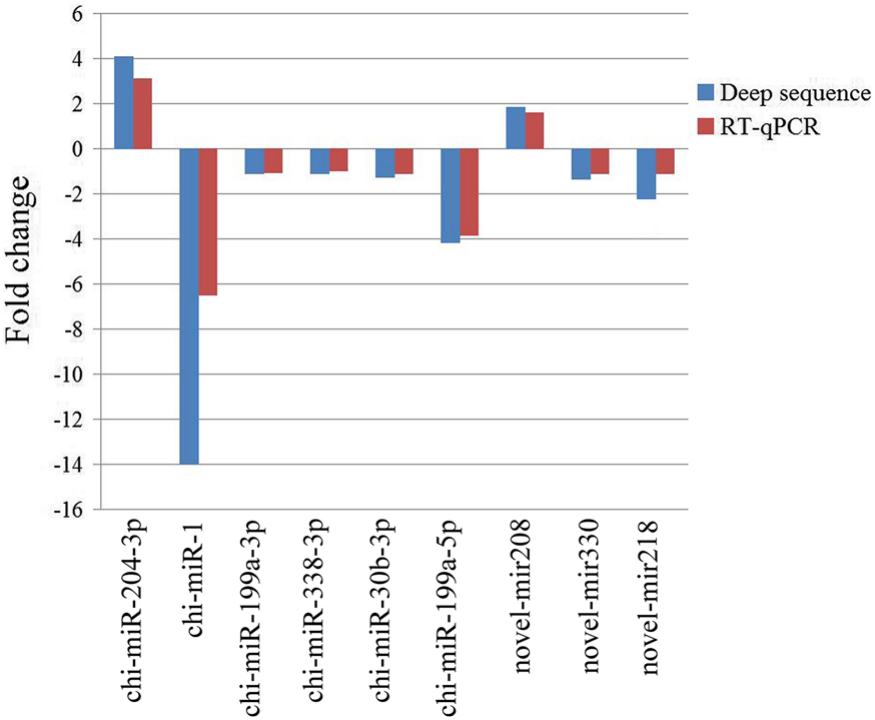
**Validation of miRNA expression by quantitative RT-PCR.** The relative expression level of each miRNA in PPRV-infected goat PBMC was calculated using the 2^−ΔΔCT^ method and represented as the n-fold change compared to the mock-infected sample. The gene 5S was used as the reference gene.

Quantitative RT-PCR was performed to validate the deep sequencing data of nine selected differentially expressed miRNA, including six known miRNA and three novel miRNA. Collectively, the relative expression levels of the selected miRNA detected with qRT-PCR were consistent with that determined with the deep sequencing results. The results confirmed the upregulation of two miRNA expression (chi-miR-204-3p, novel-mir218) and the downregulation of seven miRNA (chi-miR-338-3p, chi-miR-30b-3p, chi-miR-199a-5p, chi-miR-199a-3p, chi-miR-1, novel-mir330, novel-mir208) in mock-infected goat PBMC compared with the PPRV-infected cells (Figure 5).

## Discussion

Deep sequencing has provided a powerful tool to identify differentially expressed miRNA, especially low abundance ones, under conditions of physiological perturbation. Some miRNA can inhibit virus replication by regulating host immune responses or targeting viruses, whereas other miRNA can also promote viral replication by modulating the cellular environment [22, 32, 33]. Although many miRNA have been reported to be key regulators of virus-host interaction, to our knowledge, host miRNA expression profile in goat PBMC affected by PPRV infection has not been investigated. PBMC play an important role in pathogen recognition and induction of early immune response [10, 13, 14]. In the present study, we identified differentially expressed miRNA in goat PBMC in response to PPRV infection. We first determined the morphological changes taking place in cells in succession cells following PPRV infection and analyzed viral replication in PPRV-infected PBMC. Compared to mock-infected cells, significant CPE was observed in PPRV-infected PBMC at 24 hpi, and most cells show swelling or granular degeneration from 48 to 72 hpi. In concordance with high PPRV production detected at 24 hpi by Western blot assay, we chose 24 hpi as the time point to measure the miRNA expression profiles, because it might trigger global and strong antiviral immune responses, and avoid excessive CPE in the PPRV-infected PBMC cells. Detailed analysis revealed many differences in the global expression profile of miRNA among mock- and PPRV-infected goat PBMC. A total of 316 differently expressed miRNA were identified in PPRV-infected groups compared to the mock-infected group.

Individual miRNA have the potential to regulate the expression of multiple mRNA, and the changes in miRNA expression following viral infection are predicted to have profound effects on the host responses [34–36]. In order to evaluate the potential biological roles of DEmiRNA expressed in goat PBMC in response to PPRV infection, we predicated the potential target genes for the DEmiRNA identified in our miRNA sequencing datasets. A total of 12 065 target genes were annotated for 103 known miRNA and 213 novel miRNA differentially expressed both in mock- and PPRV-infected groups. Using the predicted target genes, the GO enrichment analysis revealed that these differentially expressed genes were functionally involved in responses to stimuli, immune system processes, biological regulation, and other cellular processes. The host innate immune system is stimulated once the TLR are engaged with pathogen associated molecular patterns (PAMP) [37]. Previous studies have shown that mRNA levels of TLR3, 7/10 of goat PBMC are upregulated by PPRV vaccine virus Sungri/96 strain infection and these control both the sensing and the proinflammatory and antiviral responses to PPRV [13, 14]. The KEGG pathway analysis in our study further determined that most genes targeted by DEmiRNA were mainly involved in important cellular pathways, including MAPK, PI3K-Akt, endocytosis, Hippo, NF-κB, viral carcinogenesis, FoxO, Jak-STAT and Toll-like receptor signaling pathways. All these functional analyses suggest that, by regulating the target genes, cellular miRNA have a profound effect on the regulation of innate immunity of goat PBMC to PPRV early infection.

Identification of differentially expressed host miRNA is just the first step towards understanding miRNA regulation of host-virus interactions. Then we must identify how the underlying mechanisms that are targeted by differentially expressed miRNA promote or inhibit PPRV replication. Dissection of miRNA modulation during PPRV infection will provide insight into the cellular mechanisms of host-virus interaction. The innate immune response is the first line of defence against viruses [38–40]. Emerging data have identified an important contribution of miRNA to the regulation of innate immune systems [41, 42]. Here, PPRV infection in goat PBMC triggered the expression of many immune-related miRNA, including miR-150, miR-146, and let-7 family as previously observed in spleen and lung tissues of goats infected with PPRV [43]. Furthermore, 15 miRNA were identified from the 316 DEmiRNA co-expressed in two groups based on fold change values and their targeted gene roles in viral infection, antiviral response and apoptosis. Interferon (IFN)-mediated pathway is a crucial part of the cellular response against viral infection [44]. It is known that most of the Morbillivruses antagonize the production of Type I IFN [45–47]. There is evidence that PPRV accessory proteins (V and C) are involved in controlling the host’s interferon responses [45, 46]. However, the latest research indicates that PPRV has a V-independent mechanism for actively inhibiting IFN-β induction in goat non-immune cells infected with wild-type and vaccine strains of PPRV [47]. Although an important role of PPRV protein interaction with host protein in evasion of IFN-induced antivirus effects has been identified [46, 47], little is known about the mechanisms for miRNA regulation of the suppression of IFN induction in PPRV infection. In this study, we found that a member of the interferon regulatory transcription factor IRF3 and interferon alpha/beta receptor IFNAR1 were annotated as gene targets by upregulated miR, chi-miR-323a-3p and chi-miR-1291, respectively. IRF3 and IRF7 play an essential role in the induction of Type I IFN (IFN-α/β). It has been reported that IFN-α/β mRNA were not detected in goat PBMC in response to Sungri/96 PPRV vaccine virus infection [13]. It is important to note that, TMEM173, a major stimulator in IFN production, was targeted by three downregulated DEmiRNA, including chi-miR-1, chi-miR-30b-3p and novel_mir3, and by two upregulated chi-miR-1291 and chi-miR-671-5p in PPRV-infected cells. This may give a new insight into how the post-transcriptional gene regulation mechanisms of PPRV escape IFN signaling.

Previous studies indicated that PPRV infection in goat PBMC can induce a classic inflammatory response characterized by enhanced expression of transcription of cytokines such as TNFα, IL-18, IL-12, IFNγ, IFNβ, as well as anti-inflammatory cytokines, including IL-4 and IL-10 [48]. In this study, it was found that tumor necrosis factor receptor superfamily member TNFRSF9 genes were targeted by chi-miR-1, chi-miR-1291, novel_mir3, while tumor necrosis factor ligand superfamily member TNFSF13B genes were targeted by novel_mir1, novel_mir85 and novel_mir70. Furthermore, it has been previously demonstrated that miRNA play an important role in regulating NF-κB signaling pathway during viral infections [49] and activation of NF-κB [50]. Our study revealed that NF-κB signaling-related molecules NFKB2 and NFKBIA were targeted by novel_mir3 and chi-miR-155-3p in PPRV infected goat PBMC, respectively.

There is evidence demonstrating that in vitro infection of goat PBMC with PPRV caused apoptosis [51, 52]. Particularly, most of the genes targeted by DEmiRNA in this study are involved in the regulation of cellular apoptosis. In this study, we found that PPRV infection triggered several DEmiRNA, including chi-miR-182, chi-miR-671-5p, novel_mir3, and novel_mir330, that target apoptosis-related genes AREL1, CASP8, APAF1, and BAX in goat PBMC. Henceforth by regulating the target genes, cellular miRNA have a profound effect on the regulation of apoptosis of goat PBMC to PPRV early infection. Further investigations are needed to clarify the underlying mechanisms on how these genes are targeted by differentially expressed miRNA and their role in promoting or inhibiting PPRV replication. Although numerous studies demonstrate that PPRV can induce apoptotic cell death in vitro, there is no evidence for in vivo host cell apoptosis. The reasons may be associated with the different expression levels of cellular receptor, infection route, strain-specific and virus infection dose between in vitro and in vivo studies.

In summary, we identified miRNA and evaluated their expression patterns in goat PBMC in response to PPRV infection using deep sequencing. This study supports previous studies indicating the importance of the miRNA landscape in the replication and pathogenesis of PPRV. One-hundred and three known differentially expressed miRNA and 213 novel miRNA candidates were identified. The target genes of these differential miRNA were estimated by GO enrichment and KEGG pathway analyses. In addition, while these data demonstrate the differentially expressed miRNA profiles in goat PBMC in response to vaccine strain of PPRV infection, more studies are needed to understand how wild-type PPRV infection affect miRNA expression patterns.

## Additional files

**Additional file 1.** **Flow chart of the miRNA prediction and differentially expressed (DE) miRNA analysis from goat PBMC infected with PPRV at 1.0 multiplicity of infection (MOI).** The 49nt sequence tags from Hiseq sequencing will go through the data cleaning analysis first, then the standard analysis will annotate the clean tags into different categories and take those which cannot be annotated to any category to predict the novel miRNA and seed edit of potential known miRNA. After getting miRNA result, target prediction for miRNA and GO enrichment and KEGG pathway for target genes will be analyzed.

**Additional file 2.** **Summary of deep sequencing data for small RNA (sRNA) in mock- and PPRV-infected goat PBMC.** A total of 30 573 869 and 30 644 798 clean reads were obtained from the uninfected and infected groups, respectively. The clean reads were annotated and classified as snRNA, rRNA, snoRNA, Rfam other sncRNA, precursor miRNA, mature miRNA, intergenic, intron, exon, and repeats.

**Additional file 3.** **Details of differentially expressed known and novel microRNA (miRNA) in PPRV- versus mock-infected goat PBMC.** A total of 316 miRNA (including 103 known miRNA and 213 novel miRNA) were differentially expressed in mock- and PPRV-infected groups. Among these 316 DEmiRNA, 147 miRNA were upregulated and 169 miRNA were downregulated in the PPRV-infected cells compared to the mock-infected cells.

**Additional file 4.** **Differentially expressed miRNA in the PPRV-infected goat PBMC compared to the mock-infected groups.** Prediction of 12 065 target genes for 103 known miRNA and 213 novel miRNA differentially expressed in the mock-infected and PPRV-infected goat PBMC.

**Additional file 5.** **WEGO analysis of target genes annotated for DEmiRNA in mock- and PPRV-infected goat PBMC.** WEGO analysis showed that a total of 12 065 target genes were successfully annotated for 103 known miRNA and 213 novel miRNA differentially expressed in two groups.

**Additional file 6.** **KEGG analysis of target genes annotated for miRNA differentially expressed in mock- and PPRV-infected goat PBMC.** KEGG pathway annotation revealed that 10 364 background genes were annotated for 317 biological processes.
