# Supplementary material for: MicroRNA expression profiling of goat peripheral blood mononuclear cells in response to peste des petits ruminants virus infection
Source: Vet Res. 2018 Jul 16;49:62. doi: 10.1186/s13567-018-0565-3 (PMC6048839; doi:10.1186/s13567-018-0565-3)
Supplement: Supplementary file 1 — Additional file 1. Flow chart of the miRNA prediction and differentially expressed (DE) miRNA analysis from goat PBMC infected with PPRV at 1.0 multiplicity of infection (MOI). The 49nt sequence tags from Hiseq sequencing will go through the data cleaning analysis first, then the standard analysis will annotate the clean tags into different categories and take those which cannot be annotated to any category to predict the novel miRNA and seed edit of potential known miRNA. After getting miRNA result, target prediction for miRNA and GO enrichment and KEGG pathway for target genes will be analyzed. [file 13567_2018_565_MOESM1_ESM.doc]

Isolation of PBMCs from PPRV negative goats tested by c-ELISA and neutralization

Mock-infected PBMCs

24 h

PPRV-infected PBMCs (MOI=1.0)

24 h

cDNA library preparation and illumina Sequencing (HiSeq-2000)

Raw data-control

Raw data-infected

Quality check and filtering of raw reads

Clean data

Length distribution

miRNA identification

KEGG Pathway analysis

Cluster

Expression/Diff

Family analysis

Target Prediction

GO enrichment

Alignment
