# Supplementary material for: MicroRNA expression profiling of goat peripheral blood mononuclear cells in response to peste des petits ruminants virus infection
Source: Vet Res. 2018 Jul 16;49:62. doi: 10.1186/s13567-018-0565-3 (PMC6048839; doi:10.1186/s13567-018-0565-3)
Supplement: Supplementary file 2 — Additional file 2. Summary of deep sequencing data for small RNA (sRNA) in mock- and PPRV-infected goat PBMC. A total of 30 573 869 and 30 644 798 clean reads were obtained from the uninfected and infected groups, respectively. The clean reads were annotated and classified as snRNA, rRNA, snoRNA, Rfam other sncRNA, precursor miRNA, mature miRNA, intergenic, intron, exon, and repeats. [file 13567_2018_565_MOESM2_ESM.docx]

| Categories | Mock-infected  Total sRNAs | PPRV-infected  Total sRNAs |
| --- | --- | --- |
| Raw reads | 33 933 810 | 33 852 635 |
| Clean reads | 30 573 869 (100%) | 30 644 798 (100%) |
| Map to genome | 29 487 110 (96.45%) | 26 296 606 (85.81%) |
| snRNAs | 231 410 (0.76%) | 89 631 (0.29%) |
| Intergenic | 4 411 718 (14.43%) | 12 238 904 (19.97%) |
| rRNAs | 154 270 (0.5%) | 478 849 (1.56%) |
| Rfam other sncRNA | 1 460 068 (4.78%) | 325 579 (1.06%) |
| Repeats | 16 (0) | 34 (0) |
| snoRNAs | 506 691 (1.66%) | 104 190 (0.34%) |
| Exon | 4 837 212 (15.82%) | 4 085 433 (13.33%) |
| Intron | 3 136 995 (10.26%) | 5 604 555 (18.29%) |
| Precursor miRNAs  Mature miRNAs | 2 249 295 (7.36%)  12 634 104 (41.32%) | 385 756 (4.59%)  8 269 704 (26.99%) |
| Unannotated sRNAs | 940 616 (3.08%) | 4 137 340 (13.5%) |
