# Supplementary material for: MicroRNA expression profiling of goat peripheral blood mononuclear cells in response to peste des petits ruminants virus infection
Source: Vet Res. 2018 Jul 16;49:62. doi: 10.1186/s13567-018-0565-3 (PMC6048839; doi:10.1186/s13567-018-0565-3)
Supplement: Supplementary file 3 — Additional file 3. Details of differentially expressed known and novel microRNA (miRNA) in PPRV- versus mock-infected goat PBMC. A total of 316 miRNA (including 103 known miRNA and 213 novel miRNA) were differentially expressed in mock- and PPRV-infected groups. Among these 316 DEmiRNA, 147 miRNA were upregulated and 169 miRNA were downregulated in the PPRV-infected cells compared to the mock-infected cells. [file 13567_2018_565_MOESM3_ESM.docx]

| **No.** | **miRNA name** | **Sequence** | **Up down**  **regulation** | **Log2 ratio** | ***P* value** |
| --- | --- | --- | --- | --- | --- |
| 1 | chi-miR-150 | ucucccaacccuuguaccagug | UP | 1.299303769 | 0 |
| 2 | chi-miR-155-5p | uuaaugcuaaucgugauaggggu | UP | 1.147744386 | 0 |
| 3 | chi-miR-24-3p | uggcucaguucagcaggaac | UP | 1.24016608 | 0 |
| 4 | chi-let-7b-5p | ugagguaguagguugugugguu | DOWN | -1.027359193 | 0 |
| 5 | chi-miR-376b-3p | aucauagaggaaaauccau | UP | 7.161538318 | 0 |
| 6 | chi-miR-376c-3p | aacauagaggaaauuccacgu | UP | 5.991763228 | 0 |
| 7 | chi-let-7d-5p | agagguaguagguugcauaguu | DOWN | -1.05392157 | 0 |
| 8 | chi-miR-206 | uggaauguaaggaaguguguggu | DOWN | -13.36148539 | 0 |
| 9 | chi-miR-423-5p | ugaggggcagagagcgagacuuu | DOWN | -1.327396564 | 0 |
| 10 | chi-miR-204-5p | uucccuuugucauccuaugccu | UP | 5.314425054 | 0 |
| 11 | chi-miR-1 | uggaauguaaagaaguauguau | DOWN | -13.97623414 | 0 |
| 12 | chi-miR-133b | uuugguccccuucaaccagcu | DOWN | -10.46815393 | 0 |
| 13 | chi-miR-10a-5p | uacccuguagauccgaauuugu | UP | 1.639029086 | 0 |
| 14 | chi-miR-369-5p | agaucgaccguguuauauucg | UP | 6.073646955 | 0 |
| 15 | chi-miR-374a-5p | uuauaauacaaccugauaagu | UP | 1.392470354 | 0 |
| 16 | chi-miR-96 | uuuggcacuagcacauuuuug | UP | 4.397653562 | 0 |
| 17 | chi-miR-200b | uaauacugccugguaaugauga | UP | 5.645998641 | 0 |
| 18 | chi-miR-369-3p | aauaauacaugguugaucuuu | UP | 4.17526114 | 0 |
| 19 | chi-miR-409-3p | gaauguugcucggugaacccc | UP | 6.141080339 | 0 |
| 20 | chi-miR-200a | uaacacugucugguaacgaug | UP | 3.77027362 | 0 |
| 21 | chi-miR-656 | aauauuauacagucaaccucu | UP | 7.436536223 | 0 |
| 22 | chi-miR-100-5p | aacccguagauccgaacuugu | UP | 1.012182744 | 0 |
| 23 | chi-miR-2332 | cgguuuaaggucuuggagacaaag | UP | 3.958357425 | 0 |
| 24 | chi-miR-411a-5p | auaguagaccguauagcguac | UP | 3.622727274 | 0 |
| 25 | chi-miR-183 | uauggcacugguagaauucacu | UP | 5.452010995 | 0 |
| 26 | chi-let-7d-3p | cuauacgaccugcugccuuuc | UP | 1.322608987 | 0 |
| 27 | chi-miR-193a | aacuggcccacaaaguccc | UP | 2.151460906 | 0 |
| 28 | chi-miR-10b-5p | uacccuguagaaccgaauuugu | UP | 4.239954298 | 0 |
| 29 | chi-miR-135b-5p | uauggcuuuucauuccuauguga | UP | 4.38694498 | 0 |
| 30 | chi-miR-410-3p | aauauaacacagauggccugu | UP | 5.944065402 | 0 |
| 31 | chi-miR-543-3p | aaacauucgcggugcacuucu | UP | 6.670660696 | 0 |
| 32 | chi-miR-34c-3p | aaucacuaaccacacggccagg | UP | 5.455525679 | 0 |
| 33 | chi-miR-381 | uauacaagggcaagcucucugu | UP | 6.651853127 | 0 |
| 34 | chi-miR-133a-3p | uuugguccccuucaaccagcugu | DOWN | -9.863118991 | 0 |
| 35 | chi-miR-182 | uuuggcaaugguagaacucac | UP | 3.246711664 | 8.31E-304 |
| 36 | chi-miR-2411-3p | cugaacugucuuacucccacauc | UP | 2.853416 | 1.48E-302 |
| 37 | chi-miR-30a-3p | cuuucagucggauguuugcag | UP | 2.636123355 | 3.85E-295 |
| 38 | chi-miR-143-3p | ugagaugaagcacuguagcucg | DOWN | -2.350697408 | 1.86E-290 |
| 39 | chi-miR-379-5p | ugguagacuauggaacguagg | UP | 3.323286189 | 7.46E-279 |
| 40 | chi-miR-487b-3p | aaucguacagggucauccacuu | UP | 4.436332466 | 1.34E-266 |
| 41 | chi-miR-1291 | gaggcccagagcacuguc | UP | 2.973693169 | 6.33E-259 |
| 42 | chi-miR-202-5p | uuccuaugcauauacuucuuu | UP | 1.110398445 | 4.92E-257 |
| 43 | chi-miR-323a-3p | cacauuacacggucgaccucu | UP | 5.843262139 | 6.59E-241 |
| 44 | chi-miR-382-5p | gaaguuguucgugguggauucg | UP | 5.37916196 | 7.02E-219 |
| 45 | chi-miR-34c-5p | aggcaguguaguuagcugauugc | UP | 4.192555894 | 1.01E-214 |
| 46 | chi-miR-155-3p | cuccuacauguuagcauuaaca | UP | 2.552559153 | 4.01E-197 |
| 47 | chi-miR-365-3p | uaaugccccuaaaaauccuuau | UP | 2.285705795 | 1.22E-177 |
| 48 | chi-miR-411a-3p | uauguaacacgguccacuaac | UP | 5.540625382 | 3.04E-164 |
| 49 | chi-miR-496-3p | ugaguauuacauggccaaucu | UP | 6.621751095 | 1.42E-157 |
| 59 | chi-miR-877-5p | guagaggagauggcgcagggg | DOWN | -1.112478979 | 1.32E-145 |
| 51 | chi-miR-495-3p | aaacaaacauggugcacuucuu | UP | 6.01224641 | 8.64E-132 |
| 52 | chi-miR-193b-3p | aacuggcccacaaagucccgcu | UP | 1.567099241 | 5.08E-120 |
| 53 | chi-miR-134 | ugugacugguugaccagagggg | UP | 5.153602259 | 1.38E-110 |
| 54 | chi-miR-196a | uagguaguuucauguuguugg | UP | 1.043742506 | 2.59E-110 |
| 55 | chi-miR-99b-3p | caagcucgugucuguggguc | UP | 1.672253599 | 1.12E-98 |
| 56 | chi-miR-494 | ugaaacauacacgggaaaccucu | UP | 5.044667888 | 7.94E-98 |
| 57 | chi-miR-376d | aucauagaggaaaauccacau | UP | 4.242306485 | 1.23E-97 |
| 58 | chi-let-7g-3p | cuguacaggccacugccuugcc | UP | 1.371501752 | 8.56E-90 |
| 59 | chi-miR-379-3p | uauguaacaugguccacuaac | UP | 5.114608128 | 8.88E-71 |
| 60 | chi-miR-218 | uugugcuugaucuaaccaugu | UP | 1.547881198 | 1.79E-63 |
| 61 | chi-miR-199c-3p | acaguagucugcacauugguu | DOWN | -6.004376191 | 3.21E-63 |
| 62 | chi-miR-671-5p | aggaagcccuggaggggcuggagg | UP | 1.032371515 | 8.28E-63 |
| 63 | chi-miR-199a-3p | acaguagucugcacauugguu | DOWN | -1.116806112 | 2.45E-62 |
| 64 | chi-miR-377 | aaucacacaaaggcaacuuuug | UP | 5.592486501 | 3.35E-60 |
| 65 | chi-miR-382-3p | aaucauucacggacaacacuu | UP | 4.878979879 | 1.18E-59 |
| 66 | chi-miR-199a-5p | cccaguguucagacuaccuguuc | DOWN | -4.175820372 | 4.33E-49 |
| 67 | chi-miR-184 | uggacggagaacugauaagggu | UP | 1.373653495 | 3.68E-48 |
| 68 | chi-miR-30b-3p | cugggagguggauguuuacuuc | DOWN | -1.252011986 | 4.26E-48 |
| 69 | chi-miR-542-5p | ucggggaucaucaugucacgaga | UP | 1.156573826 | 4.30E-48 |
| 70 | chi-miR-485-5p | agaggcuggccgugaugaauuc | UP | 4.227602841 | 1.06E-45 |
| 71 | chi-miR-1185-5p | agaggauacccuuuguaugu | UP | 4.864095642 | 9.55E-44 |
| 72 | chi-miR-130a-3p | cagugcaauguuaaaagggca | UP | 1.208365352 | 3.09E-42 |
| 73 | chi-miR-204-3p | gcugggaaggcaaagggac | UP | 4.104692659 | 8.37E-27 |
| 74 | chi-miR-10a-3p | caaauucguaucuaggggaau | UP | 1.471778219 | 1.20E-26 |
| 75 | chi-miR-301b | cagugcaaugauauugucaaagc | UP | 1.56717435 | 1.24E-26 |
| 76 | chi-miR-153 | uugcauagucacaaaagugauc | UP | 1.072919881 | 4.89E-25 |
| 77 | chi-miR-199b-3p | acaguagucugcacauugguu | UP | 1.470118995 | 1.50E-23 |
| 78 | chi-miR-214-3p | uacagcaggcacagacaggc | DOWN | -5.416253349 | 4.12E-22 |
| 79 | chi-let-7e-3p | cuauacggccuccuagcuuucc | UP | 1.058743073 | 1.02E-18 |
| 80 | chi-miR-154a-5p | uagguuauccguguagccu | UP | 7.001599166 | 2.81E-18 |
| 81 | chi-miR-655 | auaauacaugguuaaccucucu | UP | 3.749212004 | 1.56E-15 |
| 82 | chi-miR-124a | uaaggcacgcggugaaugcc | UP | 1.163053735 | 1.53E-14 |
| 83 | chi-miR-1197-3p | uaggacacauggucuacuucu | UP | 6.532113883 | 6.45E-14 |
| 84 | chi-miR-34b-5p | aggcaguguaauuagcugauugu | UP | 2.620170059 | 7.92E-13 |
| 85 | chi-miR-329b-3p | aacacaccugguuaaccucu | UP | 3.416636665 | 2.15E-08 |
| 86 | chi-miR-135b-3p | auguagggcuaaaagccaugg | UP | 4.639029086 | 5.79E-08 |
| 87 | chi-miR-483 | cacuccucuccucccgucuucu | UP | 3.639029086 | 4.18E-07 |
| 88 | chi-miR-877-3p | uccucuucucccuccucccagg | UP | 1.138651918 | 4.28E-07 |
| 89 | chi-miR-193b-5p | cgggguuuugagggcgagauga | UP | 1.494639177 | 6.85E-07 |
| 90 | chi-miR-199b-5p | cccaguguuuagacuaucuguuc | DOWN | -2.812182025 | 6.16E-06 |
| 91 | chi-miR-429 | uaauacugucugguaaugccg | DOWN | -1.416253349 | 1.19E-05 |
| 92 | chi-miR-485-3p | agucauacacggcucuccucucu | UP | 3.568639759 | 2.18E-05 |
| 93 | chi-miR-412-5p | uggucgaccaguuggaaaguaau | UP | 3.568639759 | 2.18E-05 |
| 94 | chi-miR-376c-5p | gguggauauuccuucuauguuu | UP | 2.831674164 | 2.75E-05 |
| 95 | chi-miR-214-5p | ugccugucuacacuugcugugc | DOWN | -2.660178932 | 3.11E-05 |
| 96 | chi-miR-487a-3p | aaucauacagggacauccaguu | UP | 2.609281743 | 5.92E-05 |
| 97 | chi-miR-409-5p | agguuacccgagcaacuuugcau | UP | 4.416636665 | 0.000441249 |
| 98 | chi-miR-338-3p | uccagcaucagugauuuuguu | DOWN | -1.088891368 | 0.000590808 |
| 99 | chi-miR-3955-5p | uuugauggcugauccucucacu | UP | 3.416636665 | 0.001225841 |
| 100 | chi-miR-145-5p | guccaguuuucccaggaaucccu | DOWN | -1.660178932 | 0.001992036 |
| 101 | chi-miR-105a | ucaaaugcucagacuccugugg | DOWN | -1.338250837 | 0.00389661 |
| 102 | chi-miR-450-3p | auugggaacauuuugcau | UP | 1.391101573 | 0.004289648 |
| 103 | chi-miR-758 | uuugugaccugguccacuaacc | UP | 2.024319242 | 0.004874562 |
| 104 | chi-miR-137 | auugcuuaagaauacgcguagu | UP | 3.831674164 | 0.005873033 |
| 105 | chi-miR-493-5p | uuguacaugguaggcuuucauu | UP | 1.024319242 | 0.007394879 |
| 106 | novel_mir70 | cggcggcggcgguggcggcgg | UP | 8.546165897 | 0 |
| 107 | novel_mir237 | cggcggcggcgguggcggcgg | UP | 8.547651609 | 0 |
| 108 | novel_mir85 | cggcggcggcgguggcggcgg | UP | 8.46306464 | 0 |
| 109 | novel_mir327 | uccaacguggauaccccgg | UP | 3.505216411 | 0 |
| 110 | novel_mir6 | ucgauucccggccaaugcaca | UP | 1.411311096 | 0 |
| 111 | novel_mir57 | gcucagucauguccgacucuuugc | UP | 1.715575734 | 0 |
| 112 | novel_mir3 | cacgcucaugcacacacccac | DOWN | -1.118168932 | 0 |
| 113 | novel_mir328 | ggcagagaggaaagaggcucgg | UP | 5.043235491 | 0 |
| 114 | novel_mir130 | ccuccccccgccgccccu | UP | 7.640655269 | 0 |
| 115 | novel_mir187 | gaucaacagcacggcuggccucgcc | UP | 7.498301662 | 0 |
| 116 | novel_mir257 | gggagaaagggggcgggg | UP | 5.702369555 | 0 |
| 117 | novel_mir243 | uaugucugcugaccaucac | UP | 7.578442934 | 0 |
| 118 | novel_mir281 | uauaccaggaugccagcacagu | DOWN | -2.598651663 | 0 |
| 119 | novel_mir92 | ggccccggcggcgggggcg | UP | 5.358604544 | 0 |
| 120 | novel_mir68 | ggaucagaagauucuagguuc | DOWN | -1.678044588 | 0 |
| 121 | novel_mir143 | cgcggcggcgguggcggcgg | UP | 13.576508 | 2.13E-305 |
| 122 | novel_mir332 | gaguucugggcuguagugcacuau | UP | 13.04599329 | 3.92E-235 |
| 123 | novel_mir345 | uggggguggggucggcgggg | UP | 12.97633241 | 3.75E-227 |
| 124 | novel_mir353 | gggcugggucggucgggc | UP | 2.684488087 | 8.96E-165 |
| 125 | novel_mir106 | cgcgggggcggggagcgg | UP | 12.31548994 | 2.59E-163 |
| 126 | novel_mir111 | ucggcgggcggcgggcgg | UP | 12.17152417 | 6.74E-152 |
| 127 | novel_mir45 | uauugcacucgucccggccuc | UP | 1.728580671 | 3.34E-124 |
| 128 | novel_mir319 | uuggagaaagggggcggggg | UP | 11.72041741 | 7.09E-121 |
| 129 | novel_mir29 | uaugugggacgguaaaccgcu | UP | 11.68342321 | 1.27E-118 |
| 130 | novel_mir198 | cggggcccggggccgggg | UP | 11.39391659 | 2.56E-102 |
| 131 | novel_mir156 | uaauuuuugcaaggcuuuuc | DOWN | -2.228328863 | 6.66E-91 |
| 132 | novel_mir42 | cggggccggggguggggugg | UP | 11.11707638 | 9.35E-89 |
| 133 | novel_mir223 | gggggguggggucggcggg | UP | 11.03134651 | 6.43E-85 |
| 134 | novel_mir333 | aacagucuguucagguuuuc | DOWN | -3.305083973 | 4.18E-82 |
| 135 | novel_mir12 | cagucguguccgacucuuugc | UP | 2.598789369 | 1.02E-81 |
| 136 | novel_mir64 | guugggaaagguuuccugg | UP | 10.70203888 | 1.32E-71 |
| 137 | novel_mir167 | cggggcggcggcggccgcggg | UP | 10.61303388 | 2.29E-68 |
| 138 | novel_mir337 | augccacaggaacugcagggg | UP | 10.51817469 | 4.54E-65 |
| 139 | novel_mir117 | cgcccccggagccccgcgg | UP | 10.41663667 | 1.04E-61 |
| 140 | novel_mir293 | ucggcguugguugaaaauc | UP | 3.00783112 | 8.22E-61 |
| 141 | novel_mir166 | ucgcgcucccucccccgcgc | UP | 10.38626302 | 9.74E-61 |
| 142 | novel_mir309 | ucuacagcgccuuguccaugca | DOWN | -6.892839689 | 7.24E-57 |
| 143 | novel_mir295 | ggcgggacucacgugcuuccucucg | UP | 10.2410651 | 2.73E-56 |
| 144 | novel_mir358 | uggcggggccgggggugag | UP | 10.22399159 | 8.67E-56 |
| 145 | novel_mir250 | ucacuucauccggcgacuagc | DOWN | -6.819377527 | 2.41E-54 |
| 146 | novel_mir343 | guucugagaacggacugagacc | DOWN | -6.808570772 | 5.55E-54 |
| 147 | novel_mir101 | cgcgcggggcccggggccgg | UP | 10.15360226 | 9.16E-54 |
| 148 | novel_mir24 | gucaaaaaauucguuugggu | DOWN | -1.749088348 | 4.36E-53 |
| 149 | novel_mir180 | ccucccccgccgccccuc | UP | 10.04112753 | 1.12E-50 |
| 150 | novel_mir275 | cggcucugggucuguggggag | DOWN | -6.680078489 | 7.65E-50 |
| 151 | novel_mir86 | ccgguccgccuccucccg | UP | 9.897763355 | 5.49E-47 |
| 152 | novel_mir269 | cucgggaagcuagcuggccuug | DOWN | -6.50371619 | 1.23E-44 |
| 153 | novel_mir22 | aaaaaaguuuguuuggauuuu | DOWN | -6.453728054 | 2.97E-43 |
| 154 | novel_mir176 | ugagacaggguggagaccagcc | DOWN | -6.449096606 | 3.97E-43 |
| 155 | novel_mir190 | cugacugccgccccccgcag | UP | 1.257682782 | 4.49E-40 |
| 156 | novel_mir313 | gucaguuggacccgcccucc | DOWN | -6.307877188 | 1.92E-39 |
| 157 | novel_mir311 | cgaggcgauucugaucug | UP | 3.77027362 | 1.97E-39 |
| 158 | novel_mir25 | uugcuuccuuguguccacagg | DOWN | -6.261082976 | 2.74E-38 |
| 159 | novel_mir354 | agccuaaagaagucucuuucu | UP | 9.504099506 | 3.86E-38 |
| 160 | novel_mir194 | cugcgggccggucccccccgccg | UP | 9.475530354 | 1.45E-37 |
| 161 | novel_mir285 | guggcgugguggcuugccuag | DOWN | -6.185134123 | 1.76E-36 |
| 162 | novel_mir56 | gggggugcggggggaagg | UP | 9.386263016 | 8.05E-36 |
| 163 | novel_mir352 | ugagaaagggggcggggc | UP | 9.35523612 | 3.11E-35 |
| 164 | novel_mir336 | uuggccaaaaauuucaugca | DOWN | -6.063143599 | 9.73E-34 |
| 165 | novel_mir59 | aaaaaguugauuuagguuuuu | DOWN | -6.03251198 | 4.43E-33 |
| 166 | novel_mir268 | uggcucugcgaggucggcuc | DOWN | -6.026306831 | 6.00E-33 |
| 167 | novel_mir284 | aagcccaugaacuuuuuguu | DOWN | -2.252248319 | 5.09E-30 |
| 168 | novel_mir31 | aaaauccgaacgaacuuuuug | DOWN | -5.868765554 | 9.27E-30 |
| 169 | novel_mir355 | aacagcguggauuucaguuggc | DOWN | -1.007389617 | 2.66E-29 |
| 170 | novel_mir135 | ccggggaaagcaggagugag | DOWN | -5.826537318 | 5.92E-29 |
| 171 | novel_mir172 | ucuggugcuuagacucugugcu | DOWN | -5.804950456 | 1.50E-28 |
| 172 | novel_mir32 | uuggacacuucaguacugcuac | DOWN | -5.78303568 | 3.81E-28 |
| 173 | novel_mir185 | uacuuguuagauguugaauag | DOWN | -5.775656149 | 5.20E-28 |
| 174 | novel_mir33 | uucagccguguccucuuugcg | DOWN | -5.707484647 | 8.62E-27 |
| 175 | novel_mir83 | agaucuguccugaaaccagc | DOWN | -5.684025674 | 2.21E-26 |
| 176 | novel_mir27 | uagccaguuggggaagaaug | DOWN | -2.285189593 | 2.87E-26 |
| 177 | novel_mir244 | ucagcaccauccuucccuggc | DOWN | -5.676120476 | 3.02E-26 |
| 178 | novel_mir116 | ggcggcggcggcgguggcg | UP | 8.785870475 | 5.70E-26 |
| 179 | novel_mir300 | ucccagggauguagcuccuagu | DOWN | -5.602954063 | 5.14E-25 |
| 180 | novel_mir102 | caguuaccgcuuccgcuaccg | UP | 1.654796402 | 2.19E-24 |
| 181 | novel_mir197 | ggggagcagcuagccgcguu | DOWN | -5.560643258 | 2.50E-24 |
| 182 | novel_mir80 | acaucucagucacuuucugu | DOWN | -5.53464805 | 6.48E-24 |
| 183 | novel_mir193 | aacucccgguaggcaacgc | DOWN | -1.342823567 | 4.14E-23 |
| 184 | novel_mir34 | uucucauuggccucacguccug | DOWN | -5.453728054 | 1.14E-22 |
| 185 | novel_mir107 | cgcucugacgcggucucucguc | DOWN | -5.36799818 | 2.05E-21 |
| 186 | novel_mir170 | ucgugccgcccccugcccag | DOWN | -2.094325254 | 5.37E-21 |
| 187 | novel_mir238 | uggcucugcgaggucggcuc | DOWN | -1.333232762 | 6.38E-21 |
| 188 | novel_mir76 | ggcggggccggggguggc | UP | 8.35523612 | 1.38E-20 |
| 189 | novel_mir254 | gaucuccggaggccucuucag | DOWN | -5.245141433 | 9.98E-20 |
| 190 | novel_mir81 | ccggcccgcccccggagccg | UP | 8.223991587 | 3.56E-19 |
| 191 | novel_mir227 | ccagggacagcugugaccagg | DOWN | -5.168325836 | 9.78E-19 |
| 192 | novel_mir52 | gaaucucuacggguaagugug | DOWN | -5.145605759 | 1.88E-18 |
| 193 | novel_mir26 | cgucucgucccgcccggggag | DOWN | -1.368752925 | 2.39E-18 |
| 194 | novel_mir179 | ccagggcaggauggcuaucucgg | DOWN | -5.122522146 | 3.62E-18 |
| 195 | novel_mir234 | ugucaagguggaggggucugu | DOWN | -5.122522146 | 3.62E-18 |
| 196 | novel_mir273 | ccagggcagccugugguaacag | DOWN | -5.063143599 | 1.87E-17 |
| 197 | novel_mir144 | cagggggagggcccauccaa | DOWN | -5.026306831 | 5.04E-17 |
| 198 | novel_mir28 | uugguccuagucugggugcaaa | DOWN | -5.026306831 | 5.04E-17 |
| 199 | novel_mir344 | cagccuuuguucucccugcag | DOWN | -5.026306831 | 5.04E-17 |
| 200 | novel_mir270 | cugggggagccaggaaugagg | DOWN | -4.923213338 | 7.12E-16 |
| 201 | novel_mir124 | ugccucuccgccaccuccac | DOWN | -4.89624629 | 1.38E-15 |
| 202 | novel_mir178 | uagucuccuguugucacugguc | DOWN | -4.89624629 | 1.38E-15 |
| 203 | novel_mir310 | aaaaacccgaacgagcucuuug | DOWN | -4.868765554 | 2.70E-15 |
| 204 | novel_mir324 | aaaaacccgaacgagcucuuug | DOWN | -4.868765554 | 2.70E-15 |
| 205 | novel_mir277 | uacggcaucgucuacacccg | DOWN | -4.768238678 | 2.80E-14 |
| 206 | novel_mir79 | ugucgcguccgcgggggccccg | DOWN | -4.753288336 | 3.91E-14 |
| 207 | novel_mir149 | uucggcgccgcguucugcucg | DOWN | -1.777135078 | 4.32E-14 |
| 208 | novel_mir209 | cugggcggggcgggaggc | DOWN | -4.738181444 | 5.47E-14 |
| 209 | novel_mir329 | uuugauaagcugacaugggac | DOWN | -4.738181444 | 5.47E-14 |
| 210 | novel_mir204 | cgcggggcccggguggggg | UP | 7.639029086 | 5.82E-14 |
| 211 | novel_mir218 | cucccagcgcugucaccac | DOWN | 1.831674164 | 9.37E-14 |
| 212 | novel_mir271 | uucaacggguauuuauugag | UP | 1.043684567 | 9.40E-14 |
| 213 | novel_mir267 | uagaaaguuucuuugggguuu | DOWN | -4.691887792 | 1.50E-13 |
| 214 | novel_mir90 | ucacggccucucugcccccag | DOWN | -4.691887792 | 1.50E-13 |
| 215 | novel_mir88 | acuacugagggccugaguug | DOWN | -4.676120476 | 2.10E-13 |
| 216 | novel_mir98 | ggccccggcggcgggggcg | UP | 7.532113883 | 3.57E-13 |
| 217 | novel_mir191 | cggggcccgggugggggg | UP | 7.532113883 | 3.57E-13 |
| 218 | novel_mir359 | uguggaagggcaugaagcag | DOWN | -4.644059267 | 4.12E-13 |
| 219 | novel_mir145 | ugucgcggccgcuccugcug | DOWN | -4.644059267 | 4.12E-13 |
| 220 | novel_mir114 | gaccgcgcucucucccggcag | DOWN | -1.577716772 | 1.30E-12 |
| 221 | novel_mir95 | ugggggggccggggccgggg | UP | 1.276459007 | 1.52E-12 |
| 222 | novel_mir196 | cggacgucuacgcggugagcc | DOWN | -4.560643258 | 2.23E-12 |
| 223 | novel_mir296 | cucugcccgcucucugucuuac | DOWN | -4.560643258 | 2.23E-12 |
| 224 | novel_mir96 | ggcugucggcagucugccc | UP | 7.416636665 | 2.26E-12 |
| 225 | novel_mir263 | cugucaccuuggccaccacugcuag | UP | 7.416636665 | 2.26E-12 |
| 226 | novel_mir261 | ggggaaagggggcgggga | UP | 7.416636665 | 2.26E-12 |
| 227 | novel_mir360 | cucccucccccgccgccc | UP | 7.416636665 | 2.26E-12 |
| 228 | novel_mir118 | cccccccccgcccgcgcc | UP | 7.416636665 | 2.26E-12 |
| 229 | novel_mir192 | guuuccggucgcugugcucuc | DOWN | -4.543365267 | 3.13E-12 |
| 230 | novel_mir262 | cgguggucggggcggguca | DOWN | -4.508175838 | 6.18E-12 |
| 231 | novel_mir37 | ucagucucaucugcaaagaag | DOWN | -4.49025393 | 8.67E-12 |
| 232 | novel_mir274 | cgggagaaagggggcaggg | UP | 7.291105783 | 1.47E-11 |
| 233 | novel_mir87 | ccucccggcgcccccccccccgcgg | UP | 7.291105783 | 1.47E-11 |
| 234 | novel_mir315 | uguuugacaugagagcuuccag | DOWN | -1.155977443 | 6.07E-11 |
| 235 | novel_mir78 | ggccugcacacgagcaucagcg | DOWN | -1.151837713 | 7.33E-11 |
| 236 | novel_mir340 | gagaagccggcggcggcggc | UP | 7.153602259 | 9.99E-11 |
| 237 | novel_mir330 | ucugcccacuguuuccuuccag | DOWN | -1.338250837 | 1.09E-10 |
| 238 | novel_mir184 | ccagcuugaguccaccuacaua | UP | 1.39557505 | 2.79E-10 |
| 239 | novel_mir255 | cgugcuaagucccgugugacc | DOWN | -4.276850292 | 3.69E-10 |
| 240 | novel_mir199 | cggguggucggggcggguca | DOWN | -4.212719955 | 1.03E-09 |
| 241 | novel_mir182 | aacccugacgcgccgcuguc | DOWN | -4.190693649 | 1.46E-09 |
| 242 | novel_mir228 | gcguaccaaaaguaauaaugu | DOWN | -1.483022361 | 2.12E-09 |
| 243 | novel_mir38 | aguaaguucuuuuggguuuuu | DOWN | -4.122522146 | 4.08E-09 |
| 244 | novel_mir168 | ccuccggcugccgaagauugucgag | DOWN | -4.050968885 | 1.15E-08 |
| 245 | novel_mir40 | uggugggaugucugcaggacag | DOWN | -3.975680758 | 3.22E-08 |
| 246 | novel_mir181 | ugccccgccccuugguugc | DOWN | -3.975680758 | 3.22E-08 |
| 247 | novel_mir233 | uuagcccugaccggucgcugg | DOWN | -3.949685549 | 4.55E-08 |
| 248 | novel_mir128 | ugcgcuccgucugccccugca | DOWN | -3.949685549 | 4.55E-08 |
| 249 | novel_mir189 | aauguacuuguggaguuggag | DOWN | -3.949685549 | 4.55E-08 |
| 250 | novel_mir303 | ucucugucuucaauccuguag | DOWN | -3.923213338 | 6.42E-08 |
| 251 | novel_mir280 | uugacucugcuauuugccucu | DOWN | -3.923213338 | 6.42E-08 |
| 252 | novel_mir39 | gcuauuucacgacaccagggu | DOWN | -3.89624629 | 9.07E-08 |
| 253 | novel_mir258 | uccuucauuccaccggaguc | DOWN | -3.89624629 | 9.07E-08 |
| 254 | novel_mir206 | cuggggcacucuugagggcag | DOWN | -3.89624629 | 9.07E-08 |
| 255 | novel_mir289 | uucgguuggucagaaaguucgu | DOWN | -3.812182025 | 2.56E-07 |
| 256 | novel_mir47 | acugcccuucugccccugccag | DOWN | -3.812182025 | 2.56E-07 |
| 257 | novel_mir100 | uggcuucugucgccgcaccgg | DOWN | -3.812182025 | 2.56E-07 |
| 258 | novel_mir229 | uucuucccacgcguguccgcag | DOWN | -3.78303568 | 3.61E-07 |
| 259 | novel_mir132 | cgggcuugugggugugcu | DOWN | -3.78303568 | 3.61E-07 |
| 260 | novel_mir205 | ucucuucccuugccccccaag | DOWN | -3.78303568 | 3.61E-07 |
| 261 | novel_mir148 | ggaacauggacucugggcccag | DOWN | -3.78303568 | 3.61E-07 |
| 262 | novel_mir312 | ucggggucggaggaagguuc | DOWN | -3.753288336 | 5.10E-07 |
| 263 | novel_mir77 | aaggcaaccccaugacugu | DOWN | -3.722914687 | 7.21E-07 |
| 264 | novel_mir290 | augaggauuuugcuuguuuc | DOWN | -3.691887792 | 1.02E-06 |
| 265 | novel_mir208 | uuuucacucccuccuucgcag | UP | -3.691887792 | 1.02E-06 |
| 266 | novel_mir115 | uguacucugggacucggguguc | DOWN | -3.660178932 | 1.44E-06 |
| 267 | novel_mir133 | uaacccacucuguccuucccgc | DOWN | -3.660178932 | 1.44E-06 |
| 268 | novel_mir62 | uagugguuaugguguugg | DOWN | -3.660178932 | 1.44E-06 |
| 269 | novel_mir151 | cagggggccguggccaagug | DOWN | -3.627757454 | 2.04E-06 |
| 270 | novel_mir291 | aaggaguagcgggaacgugga | DOWN | -3.560643258 | 4.06E-06 |
| 271 | novel_mir183 | ugaaauguuuaggaccacua | DOWN | -3.560643258 | 4.06E-06 |
| 272 | novel_mir249 | ugggucacagaagagggucugg | DOWN | -3.52587784 | 5.74E-06 |
| 273 | novel_mir165 | cgcguaaccacaauauugc | DOWN | -3.52587784 | 5.74E-06 |
| 274 | novel_mir146 | ucacccugcuggucucccucag | DOWN | -3.49025393 | 8.11E-06 |
| 275 | novel_mir302 | ugggccucucgccccucccag | DOWN | -3.453728054 | 1.15E-05 |
| 276 | novel_mir50 | aaaaccugaacgagccuuuug | DOWN | -3.416253349 | 1.62E-05 |
| 277 | novel_mir272 | ucugaacgaacuuuguggca | DOWN | -3.416253349 | 1.62E-05 |
| 278 | novel_mir215 | gagcguccuggcagugucuccgcgu | DOWN | -3.338250837 | 3.22E-05 |
| 279 | novel_mir91 | ugucuccuggcccuggccugcag | DOWN | -3.212719955 | 9.06E-05 |
| 280 | novel_mir256 | cgggcgggcggacgagggu | DOWN | -3.212719955 | 9.06E-05 |
| 281 | novel_mir131 | augggcccgucucuccuccag | DOWN | -3.168325836 | 0.0001277 |
| 282 | novel_mir153 | acgcgcgggagcugagag | DOWN | -3.168325836 | 0.0001277 |
| 283 | novel_mir214 | agcgcggccugcaggaggugagg | DOWN | -3.122522146 | 0.0001799 |
| 284 | novel_mir226 | uggagagaacagguggcuu | DOWN | -3.075216431 | 0.0002534 |
| 285 | novel_mir123 | agcuccagggcccgggcgcccac | DOWN | -3.075216431 | 0.0002534 |
| 286 | novel_mir251 | ccugagcccugccuccacccug | DOWN | -3.075216431 | 0.0002534 |
| 287 | novel_mir138 | gaaaguguaaggcaaggucugg | DOWN | -3.075216431 | 0.0002534 |
| 288 | novel_mir286 | cgcagaggcgggagguaugacc | DOWN | -3.026306831 | 0.0003567 |
| 289 | novel_mir139 | cggagaacagaggccucuuag | DOWN | -3.026306831 | 0.0003567 |
| 290 | novel_mir297 | ucgcugugaccuccccacgac | DOWN | -2.975680758 | 0.0005017 |
| 291 | novel_mir335 | guauccuuucugcggccguu | DOWN | -2.975680758 | 0.0005017 |
| 292 | novel_mir126 | uggagccggagcugguuag | DOWN | -2.975680758 | 0.0005017 |
| 293 | novel_mir213 | ugccagagcggucacugccag | DOWN | -2.868765554 | 0.0009904 |
| 294 | novel_mir342 | cccuggauucugagugguugug | DOWN | -2.868765554 | 0.0009904 |
| 295 | novel_mir122 | acugcccggcugccgcgcgccau | DOWN | -2.812182025 | 0.0013897 |
| 296 | novel_mir93 | cggccuaacgacgcccgucgg | DOWN | -2.812182025 | 0.0013897 |
| 297 | novel_mir259 | ugggcacuggccgccacgcg | DOWN | -2.812182025 | 0.0013897 |
| 298 | novel_mir283 | cugacccuucugcccucuccag | DOWN | -2.812182025 | 0.0013897 |
| 299 | novel_mir339 | aucgcgucaacacucguccau | DOWN | -2.812182025 | 0.0013897 |
| 300 | novel_mir248 | cuccgugaggcugaccgguuc | DOWN | -2.753288336 | 0.001948 |
| 301 | novel_mir318 | uugcugcgggugucaggaag | DOWN | -2.753288336 | 0.001948 |
| 302 | novel_mir174 | ugccggguggacgggugugc | DOWN | -2.753288336 | 0.001948 |
| 303 | novel_mir137 | cgggacugguccuucucugag | DOWN | -2.753288336 | 0.001948 |
| 304 | novel_mir245 | gaccucucugcuauucccccag | DOWN | -2.691887792 | 0.0027273 |
| 305 | novel_mir211 | uggaaggccuggcuuugcagcg | DOWN | -2.691887792 | 0.0027273 |
| 306 | novel_mir299 | ucuggaugaugauaaauguucugac | DOWN | -2.691887792 | 0.0027273 |
| 307 | novel_mir84 | cggcgcgcggcggcggcggcgg | DOWN | -2.691887792 | 0.0027273 |
| 308 | novel_mir36 | aaacccgaaugaacuuuuug | DOWN | -2.691887792 | 0.0027273 |
| 309 | novel_mir121 | cugaccgcgccgucuguccgca | DOWN | -2.627757454 | 0.0038136 |
| 310 | novel_mir173 | cgcggcugugugucuggcuc | DOWN | -2.627757454 | 0.0038136 |
| 311 | novel_mir177 | cuguccccucccuccgcccag | DOWN | -2.627757454 | 0.0038136 |
| 312 | novel_mir186 | aaaggccugaaugaacuuuuug | DOWN | -2.560643258 | 0.005325 |
| 313 | novel_mir109 | ugaccgcgcucugcccgcag | DOWN | -2.560643258 | 0.005325 |
| 314 | novel_mir325 | aggggggcggggaggguc | DOWN | -2.49025393 | 0.0074234 |
| 315 | novel_mir119 | uccauuacacuacccugccuc | DOWN | -2.49025393 | 0.0074234 |
| 316 | novel_mir239 | cagggggcggggaggguc | DOWN | -2.49025393 | 0.0074234 |
