# Supplementary material for: MicroRNA expression profiling of goat peripheral blood mononuclear cells in response to peste des petits ruminants virus infection
Source: Vet Res. 2018 Jul 16;49:62. doi: 10.1186/s13567-018-0565-3 (PMC6048839; doi:10.1186/s13567-018-0565-3)
Supplement: Supplementary file 5 — Additional file 5. WEGO analysis of target genes annotated for DEmiRNA in mock- and PPRV-infected goat PBMC. WEGO analysis showed that a total of 12 065 target genes were successfully annotated for 103 known miRNA and 213 novel miRNA differentially expressed in two groups. [file 13567_2018_565_MOESM5_ESM.doc]

**Unigene GO ID**

XM_018055695.1 GO:0045859 GO:0038180 GO:0019887 GO:0016021 GO:0001701 GO:0030165 GO:0048813

XM_005678090.3 GO:0000122 GO:0003677 GO:0043966 GO:0003714 GO:0005671 GO:0046982 GO:0017025

XM_018049493.1 GO:0045665 GO:0043065 GO:0006357 GO:0046872 GO:0000981 GO:0005634 GO:0003676

XM_018052686.1 GO:0030335 GO:0045666 GO:0051155 GO:0043621 GO:0032897 GO:0045087 GO:0000209 GO:0005863 GO:0061630 GO:0045862 GO:1903265 GO:0048147 GO:0031369 GO:0045787 GO:0003723 GO:0030307 GO:0042787 GO:1902230 GO:0007014 GO:0008270 GO:0061564 GO:0005634 GO:0009411 GO:0051092 GO:1902187 GO:0001894 GO:0046716 GO:0043123 GO:0043130 GO:0045444 GO:0045732 GO:0017022

XM_018048224.1 GO:0005654 GO:0044822 GO:0000166

XM_018041366.1 GO:0090575 GO:0042754 GO:0044822 GO:0042826 GO:0000785 GO:0016363 GO:0000380 GO:0042382 GO:0000122 GO:1902177 GO:0003682 GO:0000166 GO:0045876 GO:0000724 GO:0000980 GO:0070932 GO:0001047

XM_018053747.1 GO:0045295 GO:0030335 GO:0030877 GO:0007094 GO:0032587 GO:0007050 GO:0019901 GO:0090090 GO:0000281 GO:0043065 GO:0005913 GO:0019887 GO:0007026 GO:0051988 GO:0051010 GO:0005923 GO:0016328 GO:0006974 GO:0008285 GO:0005634 GO:0005813 GO:0045736 GO:0031274 GO:0030027 GO:0016342 GO:0045732 GO:0000776 GO:0006461 GO:0008013

XM_005690789.3 GO:0072562 GO:0016787 GO:0016180 GO:0032039 GO:0005737

XR_001917024.1 GO:0007399 GO:0043209 GO:0005739 GO:0019911

XM_018059379.1 GO:0007200 GO:0045028 GO:0035589 GO:0070257 GO:0016021 GO:0042312 GO:0005886

XM_018048562.1 GO:0016021

XM_005697668.3 GO:0070062 GO:0016021 GO:0008889 GO:0006629

XM_005689706.3 GO:0003677 GO:0046872

XM_005701851.3 GO:0070062 GO:1990254 GO:0045104 GO:0005198 GO:0045095 GO:0005737

XM_018063837.1 GO:0005634 GO:0005737

XM_018051975.1 GO:0070062 GO:0035335 GO:0016021 GO:0004725

XM_018040438.1 GO:0032364 GO:0002244 GO:0016226 GO:0046872 GO:0006355 GO:0051539 GO:0097361 GO:0001666

XM_005680173.2 GO:0005615 GO:0038128 GO:0035335 GO:0030054 GO:0001701 GO:0004725 GO:0010633 GO:0005737 GO:0019901 GO:0070373 GO:0016021 GO:0005886

XM_018040418.1 GO:0008270

XM_018045953.1 GO:0007275 GO:0016021

XM_018055020.1 GO:0000287 GO:0005615 GO:0004477 GO:0006730 GO:0046653 GO:0055114 GO:0009396 GO:0004487 GO:0004488 GO:0042301 GO:0005829 GO:0005739 GO:0004329

XM_018039643.1 GO:0005814 GO:0043234 GO:0046599

XM_005681879.3 GO:0016229 GO:0043231 GO:0006710 GO:0005811 GO:0016021 GO:0055114 GO:0005737

XM_005682996.3 GO:0060291 GO:0016021 GO:0002091 GO:0051965

XM_013967244.2 GO:0070062 GO:0005654 GO:0035749 GO:0043219 GO:0043220 GO:0090162 GO:0005737 GO:0032287 GO:0002011 GO:0070830 GO:0019904 GO:0035750 GO:0005886

XM_018064921.1 GO:0001958 GO:0035630 GO:0052732 GO:0016462 GO:0005578 GO:0065010 GO:0052731 GO:0016311

XM_018047612.1 GO:0042384 GO:0005814 GO:0036064 GO:0000922 GO:0008283 GO:0001895

XM_005680585.3 GO:0072669 GO:0044822 GO:0005524 GO:0006388 GO:0005635 GO:0001701 GO:0001890 GO:0017166 GO:0046872 GO:0005789 GO:0003972

XM_005699905.3 GO:0000978 GO:0005634 GO:2000144 GO:0001701 GO:0007296 GO:0000982 GO:0006357 GO:0060674 GO:0031668

XM_005691389.3 GO:0044822

XR_001917940.1 GO:0005524 GO:0044822 GO:0016032 GO:1900260 GO:0048471 GO:0006446 GO:0003743 GO:0005515 GO:0004004 GO:0000289 GO:0005829 GO:0010501 GO:0016281

XM_005682526.3 GO:0070062 GO:0051897 GO:0005615 GO:0014068 GO:0043066 GO:0005793 GO:0045944 GO:0043410 GO:0001938 GO:0045766

XM_013973045.2 GO:0015937 GO:0004633

XM_018067013.1 GO:0016020 GO:0014066 GO:0006513 GO:0036312 GO:0031146 GO:0019903 GO:0004842 GO:0019005 GO:0010506

XM_018055557.1 GO:0005634 GO:0007264 GO:0070374 GO:0005525 GO:0005739 GO:0071701 GO:0003924

XM_005684689.3 GO:0007165 GO:0005230 GO:0034220 GO:0030054 GO:0016021 GO:0004890 GO:0005886 GO:0045202

XM_018063433.1 GO:0046872 GO:0006355 GO:0005622 GO:0003676

XM_018041264.1 GO:0030324 GO:0048771 GO:0000902 GO:0070062 GO:0016042 GO:0048873 GO:0001816 GO:0004771 GO:0006954 GO:0008283

XM_018047610.1 GO:0009953 GO:0051409 GO:0042663 GO:0035335 GO:0060420 GO:0004725 GO:0000188 GO:0005737 GO:0017017 GO:0070373 GO:0043065 GO:0040036

XM_018047946.1 GO:0004535 GO:0016020 GO:0001226 GO:0005634 GO:0001829 GO:0000288 GO:0031047 GO:0000932 GO:0030015 GO:0000122 GO:2000036 GO:0017148 GO:0090503 GO:0010606 GO:0033147

XM_005683401.3 GO:0070062 GO:0005178 GO:0005509 GO:0010811

XM_005686838.3 GO:0016020 GO:0070062 GO:0000981 GO:0098641 GO:0003729 GO:0005654 GO:0000398 GO:0005737 GO:0005913 GO:0039694 GO:0045944 GO:0008494 GO:0030529

XM_005674990.2 GO:0007264 GO:0005622 GO:0005525

XM_018061982.1 GO:0004993 GO:0005887 GO:0007210

XM_005692127.3 GO:0005856 GO:0005634 GO:0097191 GO:0042802 GO:0070513 GO:0005737 GO:0043235 GO:0043123 GO:0060090 GO:0051798 GO:0019900 GO:0004871 GO:0005886

XM_018055764.1 GO:0010008 GO:0005886

XM_005683620.2 GO:0005615 GO:0005125 GO:0051607 GO:0043330 GO:0002250 GO:0005132 GO:0042100 GO:0006959 GO:0002286 GO:0033141 GO:0002323 GO:0030183 GO:0019221 GO:0045087

XM_013975774.2 GO:0010923 GO:0007268 GO:0019233 GO:0019900 GO:0030054 GO:0044224 GO:0045211

XM_005700482.2 GO:0007186 GO:0042802 GO:0008502 GO:0016021 GO:0005886

XM_013963800.2 GO:0008083 GO:0005634 GO:0008344 GO:0010765 GO:0098908 GO:0007254 GO:0044325 GO:0003254 GO:2001258 GO:0017080 GO:0007268 GO:0008201 GO:0050905 GO:0008543 GO:0005104

XM_018061961.1 GO:0005634 GO:0006511 GO:0007275 GO:0016567 GO:0008270 GO:0004842 GO:0016874

XM_018046991.1 GO:0051665 GO:0031625 GO:0051117 GO:0017157 GO:0031755 GO:0005886 GO:0070062 GO:0031532 GO:0051491 GO:0005925 GO:0019003 GO:0000910 GO:0043209 GO:0003924 GO:0032154 GO:0030139 GO:0017022 GO:0005525 GO:0001843 GO:0007265

XM_018054874.1 GO:0021549 GO:0005615 GO:0032874 GO:0014069 GO:0021535 GO:0042692 GO:0030672 GO:0001843 GO:0071526

XM_005689163.3 GO:0030210 GO:0050731 GO:0007171 GO:0005615 GO:0007162 GO:0002092 GO:0032680 GO:0043116 GO:2000446 GO:0042308 GO:0070062 GO:0043393 GO:0030154 GO:0014068 GO:0043524 GO:2000352 GO:0048014 GO:0043122 GO:0001933 GO:0045121 GO:0033138 GO:0043536 GO:0050918 GO:0002040 GO:0034394 GO:0014842 GO:0051897 GO:0045785 GO:0002740 GO:0005886 GO:0030097 GO:0072012 GO:0030971 GO:0031398 GO:0001701 GO:0031589 GO:0005902 GO:0070374

XM_013973892.2 GO:0007186 GO:0005654 GO:0001816 GO:0032959 GO:0006954 GO:0005543 GO:0001530 GO:0016021 GO:0048015 GO:0006935 GO:0031663 GO:0005886 GO:0004992 GO:0001875

XM_018040726.1 GO:0005765 GO:0006821 GO:0005524 GO:0005247 GO:0016021 GO:0015297

XM_018061643.1 GO:0000902 GO:0016020 GO:0005634 GO:0005737 GO:0000122 GO:0008340 GO:0007431 GO:0007028 GO:0002070 GO:0045927

XM_018057884.1 GO:0005665 GO:0001221 GO:0005524 GO:0036091 GO:0008094 GO:0003676 GO:0008026

XM_018055981.1 GO:0005938 GO:0005509 GO:0030054 GO:0005604

XM_018039607.1 GO:0000103 GO:0006457 GO:0015035 GO:0070062 GO:0055114 GO:0045454 GO:0006662 GO:0016671 GO:0034599 GO:0005739

XM_018048004.1 GO:0043547 GO:0043066 GO:0046427 GO:0060749 GO:0033601 GO:0005730 GO:0045944 GO:0007264 GO:0005739 GO:0005525 GO:0005096

XM_005699593.3 GO:0005615 GO:0000781 GO:0035407 GO:0071260 GO:0005524 GO:0042127 GO:0010569 GO:0031572 GO:0005654 GO:0035402 GO:0006975 GO:0000785 GO:0005813 GO:0048096 GO:0046602 GO:0000794 GO:0010767 GO:0045839 GO:0000086 GO:2000615 GO:0005657

XM_018048194.1 GO:0001537 GO:0016051 GO:0050659 GO:0030206 GO:0002063 GO:0042127 GO:0042733 GO:0036342 GO:0033037 GO:0009791 GO:0030512 GO:0016021 GO:0047756 GO:0007585 GO:0048703 GO:0043066 GO:0048589

XM_013965740.2 GO:0000287 GO:0005783 GO:0045332 GO:0005524 GO:0016021 GO:0004012

XM_005684933.3 GO:0031167 GO:0044822 GO:0042645 GO:0000179

XM_018041660.1 GO:0000226 GO:0015630 GO:0005198

XM_018060532.1 GO:0007186 GO:0050917 GO:0033041 GO:0016021 GO:0001582 GO:0005886 GO:0004930

XM_018057349.1 GO:0008152 GO:0016021 GO:0003824

XM_018044652.1 GO:0032007 GO:0031588 GO:0035690 GO:0005524 GO:0006355 GO:0035404 GO:0035174 GO:0042149 GO:0005737 GO:0042593 GO:0014850 GO:0004679 GO:0003682 GO:0006633 GO:0016568 GO:0016241 GO:0005634 GO:0004712 GO:0050405 GO:0006695 GO:0043066 GO:0010508 GO:0042752 GO:0046872 GO:0071380 GO:0047322 GO:0016055

XM_018038538.1 GO:0098532 GO:0044822 GO:0006334 GO:0031490 GO:0016584 GO:0000122 GO:0005719 GO:0080182 GO:0000786

XM_005677163.3 GO:0001812 GO:0009897 GO:0031623 GO:0002283 GO:0002292 GO:0038094 GO:0002554 GO:0033026 GO:0010543 GO:0032760 GO:0005887 GO:0045087 GO:0019863 GO:0001805 GO:0019767 GO:0042590 GO:0032755 GO:0032733 GO:0006911 GO:0071404 GO:0042742 GO:0050766 GO:0032998 GO:0007229 GO:0030593 GO:0072659 GO:0043306 GO:0038095 GO:0032765 GO:0001798 GO:0019886 GO:0002431 GO:0019864

XM_005678936.3 GO:0008217 GO:0043589 GO:0070062 GO:0005584 GO:0005201 GO:0005615 GO:0042802 GO:0030674 GO:0030199 GO:0005622 GO:0001568 GO:0071230 GO:0007179 GO:0048407 GO:0007266 GO:0070208 GO:0046332 GO:0001501

XM_005701086.3 GO:0016021

XM_018042419.1 GO:0016324 GO:0006970 GO:0034465 GO:0060083 GO:0043065 GO:0005901 GO:0030007 GO:0060072 GO:0042391 GO:0008076 GO:0070062 GO:0005249 GO:0045794 GO:0034765 GO:0001666 GO:0003779 GO:0046872 GO:0051592 GO:0071805

XM_018042457.1 GO:0008117 GO:0005783 GO:0048008 GO:0001822 GO:0040014 GO:0097190 GO:0007283 GO:0006672 GO:0009791 GO:0006631 GO:0016021 GO:0060021 GO:0001570 GO:0030170 GO:0030097 GO:0030149 GO:0048705 GO:0008209 GO:0010761 GO:0016831 GO:0033327 GO:0001553 GO:0008210 GO:0060325

XM_005676282.3 GO:0007093 GO:0003697 GO:0003723 GO:0005654 GO:0000784 GO:0010212 GO:0000724 GO:0005737 GO:0070876

XM_018043216.1 GO:0050890 GO:0070062 GO:0006486 GO:0003836 GO:0030173 GO:0097503

XM_005675114.3 GO:0007399 GO:0008083 GO:0005615 GO:0008344 GO:0003254 GO:0017080 GO:0008201 GO:0008543 GO:0005104 GO:0005634 GO:0007507 GO:0098908 GO:0010765 GO:0086002 GO:0007254 GO:0044325 GO:2001258 GO:0007268 GO:0050905

XM_018063742.1 GO:0004252 GO:0050709 GO:0042058 GO:0016485 GO:0016021 GO:0005789 GO:0005886

XM_018050069.1 GO:0016197 GO:0006622 GO:0005547 GO:0046872 GO:0008565 GO:0005545 GO:0005769

XM_013966730.2 GO:0006397 GO:0002244 GO:0004652 GO:0005634 GO:0005524 GO:0043631 GO:0005737 GO:0046872 GO:0071044

XM_005695992.3 GO:0005615 GO:0051431 GO:0007586 GO:0006950 GO:0042562 GO:0006171 GO:0005179

XM_018055177.1 GO:0003779 GO:0030864 GO:0042802 GO:0030835 GO:0005886 GO:0030027

XM_005699184.3 GO:0016702 GO:0016020 GO:0005788 GO:0005506 GO:0030199 GO:0018401 GO:0016222 GO:0055114 GO:0031418 GO:0005515 GO:0004656 GO:0005581 GO:0005739

XM_005688774.2 GO:0005634 GO:0006355 GO:0005737

XM_018066645.1 GO:1900037 GO:0071108 GO:0004843 GO:0030433 GO:0031625 GO:0051879 GO:0005829 GO:0005789 GO:1990380 GO:0050821 GO:1901799 GO:0048642

XM_018056818.1 GO:2000807 GO:0007156 GO:0050805 GO:0005509 GO:0030534 GO:0016021 GO:0005886

XM_018056227.1 GO:0009124 GO:0046872 GO:0005524 GO:0009133 GO:0017111 GO:0016021 GO:0005886 GO:0017110

XM_013969912.2 GO:0007186 GO:0050911 GO:0005549 GO:0004984 GO:0016021 GO:0005886 GO:0004930

XM_005694801.1 GO:0005509

XM_013973419.2 GO:0000166 GO:0045727 GO:0003730

XM_005691196.3 GO:0003707 GO:0003677 GO:0005634 GO:0006355 GO:0043401 GO:0005496 GO:0005737

XM_018047086.1 GO:0009952 GO:0071837 GO:0005654 GO:0001525 GO:0060017 GO:0006355 GO:0021615 GO:0048538 GO:0051216 GO:0001974 GO:0030878 GO:0010159 GO:0048645 GO:0048704 GO:0043565 GO:0003700 GO:0008284

XM_018062623.1 GO:0016020 GO:0017186 GO:0005794 GO:0008270 GO:0016603

XM_018039630.1 GO:0019904 GO:0007268 GO:0030054 GO:0014069 GO:0032403 GO:0045211

XM_018052253.1 GO:0008017 GO:0044772

XM_005685442.2 GO:0007186 GO:0050911 GO:0004984 GO:0016021 GO:0005886 GO:0004930

XM_018042372.1 GO:0007399 GO:0014733 GO:0005524 GO:0030666 GO:0043547 GO:0030073 GO:1901897 GO:0051924 GO:0005829 GO:0004683 GO:0006470 GO:0005516 GO:0033017 GO:0005886 GO:0051259 GO:0060333 GO:0005654 GO:0000165 GO:0030154 GO:0042803 GO:0046777 GO:1900034 GO:0005088 GO:0000082 GO:0004723

XM_013963305.2 GO:0051454 GO:0005260 GO:0005524 GO:0016324 GO:0006904 GO:1902943 GO:0035774 GO:0045921 GO:0071320 GO:0030301 GO:0015106 GO:0031205 GO:1902161 GO:0019869 GO:0005829 GO:0030165 GO:0055037 GO:1902476 GO:0070062 GO:0006695 GO:0034707 GO:0060081 GO:0015701 GO:0031901 GO:0009986 GO:0048240 GO:0019899

XM_018050555.1 GO:0030425 GO:0030335 GO:0030822 GO:0008200 GO:0019903 GO:0032436 GO:0051901 GO:0043025 GO:0051343 GO:0071363 GO:0050765 GO:0051898 GO:0070062 GO:0033137 GO:0044822 GO:0001891 GO:0061099 GO:0048471 GO:0071333 GO:0006919 GO:0051726 GO:0030292 GO:0042998 GO:0043547 GO:0010629 GO:0030496 GO:0005829 GO:0001934 GO:0005739 GO:0030308 GO:0005634 GO:0008656 GO:0032464 GO:0030971 GO:0035591 GO:0042803 GO:0042169 GO:0005080 GO:2001244 GO:1903208

XM_005674802.2 GO:0070507 GO:0005524 GO:0048013 GO:0010976 GO:0032956 GO:0071300 GO:0005004 GO:0043087 GO:0051893 GO:0005887 GO:0005769 GO:0018108

XM_013962624.2 GO:0006886 GO:0005794 GO:0005829 GO:0031338 GO:0042147 GO:0017137 GO:0005096

XM_005684007.3 GO:0016020 GO:0005615 GO:0003714 GO:0030199 GO:0035064 GO:0055114 GO:0016570 GO:0032332 GO:0005044 GO:0005507 GO:0003682 GO:0001837 GO:0004720 GO:0016568 GO:0001935 GO:0005604 GO:0045892 GO:0005654 GO:0043542 GO:0005694 GO:0046688 GO:0070492 GO:0001666 GO:0018277 GO:0006898 GO:0002040

XM_018050890.1 GO:0010216 GO:0010390 GO:0061630 GO:0031493 GO:0042802 GO:0035064 GO:0016363 GO:0044729 GO:0000122 GO:0051865 GO:0008270 GO:0005720 GO:0005657

XM_018063528.1 GO:0046951 GO:0006642 GO:0000038 GO:0004467 GO:0001676 GO:0009925 GO:0015245 GO:0030176 GO:0031957 GO:0015911 GO:0006699

XM_018060564.1 GO:0005605 GO:0070062 GO:0043113 GO:0007213 GO:0043236 GO:0005737 GO:0005509 GO:0005886

XM_018059377.1 GO:0007200 GO:0045028 GO:0035589 GO:0070257 GO:0016021 GO:0042312 GO:0005886

XM_013966844.2 GO:0080008 GO:0005654 GO:0016567

XM_018064683.1 GO:0009306 GO:0030173 GO:0016192

XM_005680804.1 GO:0003779 GO:0016020 GO:0005634 GO:0051016 GO:0001669 GO:0007286 GO:0008290 GO:0030863

XM_018040904.1 GO:0000784 GO:0047485 GO:0006295 GO:0006296 GO:0034644 GO:0003697 GO:0032205 GO:0009650 GO:0070522 GO:0000712 GO:0000014 GO:0005669 GO:0000110 GO:0043566 GO:0008022 GO:0003684 GO:1901255 GO:0000724 GO:0001094

XM_013970652.2 GO:0044822

XM_018060240.1 GO:0070062 GO:0031965 GO:0006355 GO:0000785 GO:0000910 GO:0016363 GO:0005737 GO:0031080 GO:0000777 GO:0003700 GO:0051292

XM_005684185.3 GO:0050850 GO:0050731 GO:0032481 GO:0090237 GO:0019370 GO:0002092 GO:0010803 GO:0004716 GO:0090330 GO:0002554 GO:0019901 GO:0019815 GO:0005178 GO:0031234 GO:0045087 GO:0042101 GO:0070372 GO:0045579 GO:0002250 GO:0007257 GO:0018105 GO:0048514 GO:0050764 GO:0032928 GO:0071404 GO:0005524 GO:0038083 GO:0032009 GO:0046641 GO:0042742 GO:0007169 GO:0045401 GO:0045425 GO:0071226 GO:0007229 GO:0002281 GO:0043366 GO:0050853 GO:0005634 GO:0030593 GO:0004715 GO:0001945 GO:0043306 GO:0046638 GO:0051090 GO:0042991 GO:0045780 GO:0045588 GO:0004674 GO:0050715 GO:0043313 GO:0033630

XM_018052345.1 GO:0051260

XM_018050113.1 GO:0016020 GO:0070062 GO:0005634 GO:0016337 GO:0097202 GO:0030054 GO:0006915 GO:0048471 GO:0043005 GO:0005515 GO:0005829 GO:0038096 GO:0048010 GO:0045202

XM_018051247.1 GO:0021680 GO:0001228 GO:0005634 GO:0006260 GO:0021707 GO:0000122 GO:0048708 GO:0000977 GO:0045944

XM_005694750.3 GO:0008081 GO:0006629

XM_005684173.2 GO:0016021 GO:0031966 GO:0005789

XM_018048215.1 GO:0005759 GO:0006355 GO:0042803 GO:0044212 GO:0003690 GO:0006393

XM_018066170.1 GO:0016021

XM_018040731.1 GO:0004864 GO:0019902 GO:0010923

XM_018055645.1 GO:0003677 GO:0071339 GO:0000083 GO:0005667 GO:0003700

XM_005677234.3 GO:0051935 GO:0016323 GO:0055075 GO:0005242 GO:0034765 GO:0007601 GO:0022010 GO:0060075 GO:0007628 GO:0016021 GO:0048169 GO:0071805

XM_005675315.3 GO:0070062 GO:0005856 GO:0031532 GO:0005654 GO:0072659 GO:0005524 GO:0016324 GO:0005737 GO:0030033 GO:0046777 GO:0007256 GO:0004702 GO:0048814 GO:0002230

XM_018064109.1 GO:0046872 GO:0003676

XR_001296636.2 GO:0016020 GO:0097421 GO:0044822 GO:0005925 GO:0000184 GO:0022625 GO:0006364 GO:0006614 GO:0006413 GO:0005730 GO:0005515 GO:0019083 GO:0003735 GO:0070180

XM_005694133.3 GO:0005654 GO:0006355 GO:0045111 GO:0010942

XM_005684188.3 GO:0050850 GO:0050731 GO:0032481 GO:0090237 GO:0019370 GO:0002092 GO:0010803 GO:0004716 GO:0090330 GO:0002554 GO:0019901 GO:0019815 GO:0005178 GO:0031234 GO:0045087 GO:0042101 GO:0070372 GO:0045579 GO:0002250 GO:0007257 GO:0018105 GO:0048514 GO:0050764 GO:0032928 GO:0071404 GO:0005524 GO:0038083 GO:0032009 GO:0046641 GO:0042742 GO:0007169 GO:0045401 GO:0045425 GO:0071226 GO:0007229 GO:0002281 GO:0043366 GO:0050853 GO:0005634 GO:0030593 GO:0004715 GO:0001945 GO:0043306 GO:0046638 GO:0051090 GO:0042991 GO:0045780 GO:0045588 GO:0004674 GO:0050715 GO:0043313 GO:0033630

XM_018053976.1 GO:0055085 GO:0005524 GO:0042626 GO:0016021 GO:0005789 GO:0005777 GO:0009235

XM_013976688.2 GO:0005615 GO:0030335 GO:0001755 GO:0016525 GO:0048843 GO:0050919 GO:0008360 GO:0045063 GO:0030215 GO:0045499 GO:0016021 GO:0038191 GO:0010594 GO:0005886 GO:0071526

XM_005680371.3 GO:0016020 GO:0003677 GO:0031625 GO:0007050 GO:0005737 GO:0008283 GO:0005730 GO:0016787 GO:0045597 GO:0003700 GO:0070062 GO:0045892 GO:0005654 GO:0044822 GO:0043066 GO:0006364 GO:0006417 GO:0030529

XM_013973238.2 GO:0004674 GO:0005524 GO:0006468

XM_005696851.3 GO:0007205 GO:0051482 GO:0005615 GO:0043179 GO:0030335 GO:0006885 GO:0007166 GO:0051216 GO:0042313 GO:0005179 GO:0060298 GO:0048016 GO:0042474 GO:0030185 GO:0001516 GO:0009953 GO:0005125 GO:0007507 GO:0019722 GO:0045793 GO:0001666 GO:0015758 GO:0014824 GO:0001569 GO:0010870 GO:0007589 GO:0019229 GO:0014065 GO:0051771 GO:0000122 GO:0031583 GO:0014826 GO:0005829 GO:0045840 GO:0030818 GO:0060585 GO:0014032 GO:0007585 GO:0030072 GO:0010460 GO:0003100 GO:0001701 GO:0043507 GO:0032269 GO:0010613 GO:0046887 GO:0035815 GO:0031708 GO:0048661 GO:0035810 GO:0031707

XR_001295787.2 GO:0005759 GO:0046872 GO:0006470 GO:0004722

XM_018061079.1 GO:0018117 GO:0016021 GO:0070733

XM_018051103.1 GO:0070563 GO:0043069 GO:2000134 GO:0006915 GO:0000122 GO:0005739 GO:0008285 GO:0033147

XM_018057198.1 GO:0032467 GO:0051015 GO:0036449 GO:0071437 GO:0030054 GO:0005737 GO:0030496 GO:0032154 GO:0002102 GO:0007010 GO:0005886

XM_013967308.2 GO:0005634 GO:0005829 GO:0043066 GO:0042787 GO:0004842 GO:0016874

XM_018067008.1 GO:0006357 GO:0005654 GO:0001525 GO:0043565 GO:0003700 GO:0005737

XM_018038312.1 GO:0005615 GO:0010951 GO:0004867 GO:0042802 GO:0035987 GO:0005604

XM_005684795.2 GO:0006198 GO:0046872 GO:0005829 GO:0007268 GO:0004115 GO:0019933

XM_018050641.1 GO:0031175 GO:0005886 GO:0051219

XM_005677166.3 GO:0070062 GO:0006486 GO:0046872 GO:0032580 GO:0003945 GO:0016021 GO:0003831

XM_018046781.1 GO:0003677 GO:0031965 GO:2000191 GO:0044822 GO:0006260 GO:0046872 GO:0005811 GO:2001273 GO:0022626

XM_005674662.2 GO:1902260 GO:0035690 GO:1901800 GO:0071435 GO:1901979 GO:1902282 GO:0060307 GO:0008076 GO:0015459 GO:0005242 GO:0005764 GO:0005251 GO:0086091 GO:0010107 GO:0098915 GO:0044325 GO:0009986

XM_005676052.2 GO:0000090 GO:0045471 GO:0090090 GO:0016920 GO:0070628 GO:0000209 GO:0070536 GO:0070062 GO:0005654 GO:0090263 GO:0002479 GO:0000724 GO:0038061 GO:0008541 GO:0006521 GO:0010950 GO:0051437 GO:0008237 GO:0061133 GO:0043488 GO:0033209 GO:0004175 GO:0000165 GO:0050852 GO:0002223 GO:0031597 GO:0061136 GO:0038095 GO:0046872 GO:0060071 GO:0051436 GO:0031145 GO:0006303

XM_018048136.1 GO:0016021 GO:0005789

XM_005674816.2 GO:0007165 GO:0005230 GO:0034220 GO:0030054 GO:1902711 GO:0016021 GO:0004890 GO:0005886 GO:0045202

XM_005692170.3 GO:0050660 GO:0055114 GO:0003725 GO:0004860 GO:0017150 GO:0002943 GO:0005829 GO:0060548 GO:0005739

XR_001919023.1 GO:0005765 GO:0005770 GO:0006886 GO:0097352 GO:0030897 GO:0008333 GO:0005769 GO:0055037

XM_018063858.1 GO:0009611 GO:0098655 GO:0042177 GO:0030054 GO:0014069 GO:0005261 GO:0004972 GO:0017146 GO:0035235 GO:0033058 GO:0008104 GO:0005234 GO:0050885 GO:0060079 GO:0045211

XM_018054390.1 GO:0007018 GO:0005654 GO:0008152 GO:0005524 GO:0051225 GO:0008017 GO:0005814 GO:0008289 GO:0005794 GO:0003777

XM_018041147.1 GO:0060444 GO:0005737 GO:0045893 GO:0046983 GO:0019005 GO:0060828 GO:0071407 GO:0045309 GO:0006470 GO:0031648 GO:0000209 GO:0045892 GO:0005634 GO:0061630 GO:0045862 GO:0033598 GO:0043122 GO:0061136 GO:0043161 GO:0042753 GO:0051726 GO:0008013

XM_018039188.1 GO:1901981 GO:0005634 GO:0043547 GO:0043507 GO:0001726 GO:0046872 GO:0035023 GO:0005769 GO:0005089

XM_018057760.1 GO:0008083 GO:0005615 GO:0040014 GO:0035137 GO:0007184 GO:0030513 GO:0005160 GO:0032332 GO:2001054 GO:0010862 GO:0048468 GO:0005125 GO:0042802 GO:0005622 GO:0043408 GO:0030326 GO:0035136 GO:0060395 GO:0032331 GO:0050680 GO:0060591 GO:0036122

XM_013976469.2 GO:0002128 GO:0002181 GO:0008175 GO:0005737

XM_018044617.1 GO:0046872

XM_005680898.3 GO:0007186 GO:0090023 GO:0010575 GO:0010759 GO:0004943 GO:0005829 GO:0016021 GO:0007204 GO:0045766

XM_018049411.1 GO:0061092 GO:0000287 GO:0070062 GO:0005783 GO:0030335 GO:0005524 GO:0007612 GO:0005794 GO:0016021 GO:0004012 GO:0005886

XM_018061709.1 GO:0070936 GO:0016020 GO:0005768 GO:0061630 GO:0043161 GO:0005829

XM_018038223.1 GO:0007264 GO:0043547 GO:0005085 GO:0005737

XM_018059798.1 GO:0000287 GO:0005634 GO:0005524 GO:0035556 GO:0032870 GO:0005737 GO:0046777 GO:0004674

XM_018060153.1 GO:0008277 GO:0043547 GO:0005737 GO:0005096

XM_018045893.1 GO:0034765 GO:0031594 GO:0051260 GO:0005251 GO:0043679 GO:0071805 GO:0046928 GO:0008076

XM_018058380.1 GO:0046872

XM_005681976.3 GO:0006457 GO:0005759 GO:0005634 GO:0051087 GO:0050790 GO:0042803 GO:0051082 GO:0000774

NM_001285540.1 GO:0034150 GO:0050707 GO:0042088 GO:0042495 GO:0016021 GO:0004888 GO:0035663 GO:0045410 GO:0034130 GO:0042116 GO:0043507 GO:0006954 GO:0043123 GO:0071726 GO:0045121 GO:0042496 GO:0005794 GO:0002755

XM_018043401.1 GO:0035176 GO:0007416 GO:0007269 GO:0046872 GO:0042297 GO:0043234 GO:0030534 GO:0016021 GO:0005246 GO:0071625

XM_005687003.3 GO:0030177 GO:0070062 GO:0005765 GO:0015991 GO:0008553 GO:0033180 GO:0046983

XM_005698291.3 GO:0018345 GO:0005783 GO:0005794 GO:0008270 GO:0016021 GO:0019706

XM_018059600.1 GO:0090136 GO:0045218 GO:0070062 GO:0005634 GO:0005813 GO:0005915 GO:0070097 GO:0005737

XM_018058519.1 GO:0016020 GO:0006479 GO:0004719 GO:0005737

XM_018057591.1 GO:0070129 GO:0000287 GO:0005761 GO:0005743 GO:0005525 GO:0044065 GO:0003924

XM_005684773.3 GO:0016020 GO:0005654 GO:0010243 GO:0044822 GO:0000184 GO:0005763 GO:0022625 GO:0006364 GO:0006614 GO:0006413 GO:0032543 GO:0019083 GO:0022627 GO:0003735

XM_018042709.1 GO:1902043 GO:0005634 GO:0005794 GO:0006977 GO:0006919 GO:0043066

XM_013967784.2 GO:0051015 GO:0070062 GO:0031532 GO:0007165 GO:0034446 GO:0022414 GO:0031258 GO:0009986 GO:0005518 GO:0016021 GO:0004888 GO:0031527

XM_018056356.1 GO:0004817 GO:0006423 GO:0005524

XM_018043172.1 GO:0006865 GO:0005887 GO:0036233 GO:0003333 GO:0015375 GO:0005328 GO:0006836 GO:0060012

XM_018051927.1 GO:0003677 GO:0005634 GO:0060485 GO:0003416 GO:0005737 GO:0043586 GO:0046872 GO:0060021 GO:0005886

XM_018056002.1 GO:0044822

XM_018059278.1 GO:0005874

XM_005699602.3 GO:0031119 GO:0003723 GO:0009982

XM_018060763.1 GO:0051092 GO:0016567 GO:0031625 GO:0005813 GO:0004842 GO:0043123 GO:0005829 GO:0008270 GO:0031996 GO:0042981

XM_005680473.3 GO:0045892 GO:0000978 GO:0005654 GO:0001525 GO:0003714 GO:0045944 GO:0042060 GO:0001077 GO:0005739 GO:0032422

XM_013963001.2 GO:0016020 GO:0031012 GO:0070062 GO:0030049 GO:0005903 GO:0003774 GO:0005515 GO:0005829 GO:0005509 GO:0007519 GO:0008307 GO:0016461 GO:0030898

XM_005691827.3 GO:0006694 GO:0003854 GO:0016021 GO:0055114

XM_018048952.1 GO:0060044 GO:0000165 GO:0005524 GO:0006950 GO:0005622 GO:0010628 GO:2001184 GO:0098586 GO:0004707

XM_018048175.1 GO:0003677 GO:0035093 GO:0051301 GO:0051321 GO:0000800 GO:0000775

XM_018039476.1 GO:0003713 GO:0045944 GO:0016311 GO:0008195 GO:0006629

XM_005698524.3 GO:0035773 GO:0030010 GO:0005768 GO:0005654 GO:0045055 GO:0042803 GO:0019901

XM_005700431.3 GO:0004252 GO:0070062 GO:0005615 GO:0007596 GO:0005509 GO:0031638

XM_018059140.1 GO:0070062 GO:0007264 GO:0016021 GO:0043547 GO:0005737 GO:0005096

XR_001917835.1 GO:0009301 GO:0005730 GO:0070940 GO:0008420 GO:0005737 GO:0016591

XM_013976840.2 GO:0045028 GO:0035589 GO:0007596 GO:0015057 GO:0016021 GO:0070493

XM_018043330.1 GO:0061304 GO:0046982 GO:0031625 GO:0017147 GO:0045893 GO:0031987 GO:0035426 GO:0004930 GO:0051091 GO:0007223 GO:0005911 GO:0016021 GO:0042813 GO:0030165 GO:0001570 GO:0005886 GO:0030947 GO:0007186 GO:0070062 GO:0042701 GO:0019955 GO:0034446 GO:0042803 GO:0043507 GO:0060070 GO:0009986 GO:0007605 GO:0010812 GO:0061301

XM_018046602.1 GO:0004920 GO:0016021 GO:0019221 GO:0005886

XM_018058242.1 GO:0071447 GO:0007050 GO:0045893 GO:0034644 GO:0016209 GO:0005829 GO:0048102 GO:0005776 GO:0008285 GO:0071361 GO:0005634 GO:0072703 GO:0030336 GO:0010508 GO:0009408 GO:2001235

XM_005689800.3 GO:0006914 GO:0016567 GO:0051607 GO:0008270 GO:0005622 GO:0045087 GO:0004842

XM_018049191.1 GO:0070062 GO:0005783 GO:0005506 GO:0055114 GO:0008475 GO:0031418 GO:0001666

XM_005685753.3 GO:0046872 GO:0007268 GO:0005622 GO:0035556 GO:0019992

XM_018053868.1 GO:0070062

XM_013973038.2 GO:0000978 GO:0005634 GO:0046982 GO:0001701 GO:0001077 GO:0007420 GO:0017162 GO:0005737 GO:0001666 GO:0005667 GO:0045944

XM_013965352.2 GO:0016021

XM_018062464.1 GO:0055113 GO:0003143 GO:0097155 GO:0061371 GO:0060971 GO:0048842 GO:0016021 GO:0042074 GO:0060976 GO:0070062 GO:0005634 GO:0071907 GO:0030509 GO:0030326 GO:0048704 GO:0005509 GO:0097094

XM_018041283.1 GO:0035257 GO:0000226 GO:0008283 GO:0021987 GO:0005730 GO:0015630 GO:0005737

XM_005686292.3 GO:0004910 GO:0016021 GO:0019221

XM_018063473.1 GO:0003677 GO:0005634 GO:0008270

XR_001919473.1 GO:0000122 GO:0003677 GO:0046872 GO:0005634 GO:0043392 GO:0045893 GO:0001078

XM_018053054.1 GO:0042127 GO:0042177 GO:0045471 GO:0071560 GO:0046875 GO:0031234 GO:0045087 GO:0008360 GO:0002250 GO:0005768 GO:0005884 GO:0001764 GO:0030900 GO:0001664 GO:0045121 GO:0031397 GO:0005524 GO:0038083 GO:0010629 GO:0015631 GO:0070851 GO:0007169 GO:0036120 GO:0050798 GO:0005634 GO:1900182 GO:0004715 GO:0050852 GO:0010976 GO:0048813 GO:0043123 GO:0046872 GO:0044325 GO:0042523 GO:0001948 GO:0050966 GO:0042981

XM_005677640.2 GO:0070062 GO:0005654

XM_018048721.1 GO:0016709 GO:0005856 GO:0005654 GO:0055114 GO:0005737 GO:0003779 GO:0008270 GO:0030042 GO:0005886 GO:0017137 GO:0006887 GO:0071949

XM_018038371.1 GO:0005634 GO:0006355 GO:0043565 GO:0042551 GO:0048665 GO:0010628 GO:0003700

XM_005683789.3 GO:0009952 GO:0005615 GO:0001822 GO:0043043 GO:0030323 GO:0003279 GO:0007566 GO:0006465 GO:0019058 GO:0016486 GO:0016021 GO:0060976 GO:0004252 GO:0007368 GO:0002001 GO:0042277 GO:0035108 GO:0030141 GO:0005794 GO:0048706 GO:0042089 GO:0048566

XM_018065773.1 GO:0016605 GO:0000781 GO:0003721 GO:0005737 GO:0042162 GO:0046872 GO:0005730 GO:0007004 GO:0000333 GO:0070034

XM_018050053.1 GO:0016020 GO:0043231 GO:0001649 GO:0005201 GO:0005615 GO:0007417 GO:0005540 GO:0005578 GO:0007155 GO:0030246 GO:0008347 GO:0005509 GO:0001501

XM_018043283.1 GO:0000287 GO:0031532 GO:0050321 GO:0005634 GO:0005783 GO:0005524 GO:0005813 GO:0051117 GO:0007409 GO:0019901 GO:0030010 GO:0018105 GO:0004674 GO:0070059 GO:0061178 GO:0000086

XM_013968307.2 GO:0008270 GO:0030155 GO:0023051

XM_013962921.2 GO:0016021

NM_001285641.1 GO:0030054 GO:0043491 GO:0045471 GO:0046627 GO:0071363 GO:0009749 GO:0051721 GO:0009611 GO:0042493 GO:0031929 GO:0009612 GO:0005654 GO:0042802 GO:0006468 GO:0042277 GO:0009408 GO:0001662 GO:0043005 GO:0048471 GO:0014732 GO:0007281 GO:0034612 GO:0000082 GO:0046324 GO:0005524 GO:0004711 GO:0032496 GO:0014065 GO:0005741 GO:0045948 GO:0007616 GO:0009636 GO:2001237 GO:0071407 GO:0033574 GO:0005829 GO:0007584 GO:0030165 GO:0045202 GO:0003009 GO:0051384 GO:0014911 GO:0004712 GO:0014878 GO:0045931 GO:0007568 GO:0009986 GO:0048633 GO:0043201 GO:0048661 GO:0033762

XM_018042916.1 GO:1900121 GO:0070062 GO:0016477 GO:0035335 GO:0004725 GO:0043025 GO:0043005 GO:0035373 GO:0048679 GO:0016021 GO:0032403

XM_018049025.1 GO:0007186 GO:0050911 GO:0004984 GO:0016021 GO:0005886 GO:0004930

XM_018060337.1 GO:0070829 GO:0072341 GO:0004489 GO:0050660 GO:0055114 GO:0006555 GO:0031060 GO:0050667 GO:0035999 GO:0032403

XM_013962627.2 GO:0006886 GO:0005794 GO:0005829 GO:0031338 GO:0042147 GO:0017137 GO:0005096

XM_018051074.1 GO:0043252 GO:0015132 GO:0005887 GO:0015732 GO:0015347

XM_018063568.1 GO:0042130 GO:0008270 GO:0002643

XM_018062447.1 GO:0031397 GO:0043161 GO:0031463

XM_018052608.1 GO:0005654 GO:0006281 GO:0010212 GO:0070876

XR_001296225.2 GO:0070062 GO:0005929

XM_018041955.1 GO:0045892 GO:0003677 GO:0016605 GO:0004402 GO:0006334 GO:0030099 GO:0070776 GO:0045893 GO:0043966 GO:0090398 GO:0003713 GO:0005794 GO:0008270 GO:0008134 GO:0000786

XM_005690906.3 GO:0070062 GO:0005654 GO:0030877 GO:0042803 GO:0005641

XM_005676764.3 GO:0003677 GO:0004402 GO:0046982 GO:0000125 GO:0005669 GO:0033276 GO:0043966 GO:0051091 GO:0003713 GO:0030914 GO:0006367 GO:0003700 GO:0008134

XM_018043018.1 GO:0031965 GO:0008536 GO:0008565 GO:0006606 GO:0005737 GO:0008139

XM_005682900.3 GO:0005665 GO:0045815 GO:0032481 GO:0003677 GO:0006361 GO:0042795 GO:0006370 GO:0006283 GO:0035019 GO:0001054 GO:0006368 GO:0006362 GO:0005515 GO:0005829 GO:0008543 GO:0005736 GO:0006363 GO:0000398 GO:0001055 GO:0050434 GO:0005666 GO:0031047 GO:0001056 GO:0006367 GO:0006383

XM_018044158.1 GO:0005730 GO:0007062 GO:0000785 GO:0045111 GO:0032876 GO:0015629 GO:0005886

XM_018045191.1 GO:0046034 GO:0070062 GO:0005759 GO:0005524 GO:0006165 GO:0006172 GO:0004017 GO:0004550 GO:0009142 GO:0046899 GO:0046033 GO:0005525 GO:0046039

XM_013965775.2 GO:0030833 GO:0046580 GO:0043524 GO:0043547 GO:0005102 GO:0005737 GO:0001726 GO:0000281 GO:0009790 GO:0001953 GO:0051020 GO:0001948 GO:0031235 GO:0001570 GO:0005096

XM_018039483.1 GO:0070062 GO:0004843 GO:0010951 GO:0000502 GO:1903070 GO:0016023 GO:0009986 GO:0007268 GO:0050920 GO:0016579 GO:0070628 GO:0004866 GO:0005886 GO:0045202

XM_018039633.1 GO:0003148 GO:0030513 GO:0043627 GO:0032912 GO:0051891 GO:0003705 GO:0003309 GO:0008584 GO:0019901 GO:0032911 GO:0003682 GO:0045766 GO:0048468 GO:0060486 GO:0042493 GO:0055007 GO:0043066 GO:0060575 GO:0045944 GO:0071456 GO:0001228 GO:0014898 GO:0001103 GO:0035239 GO:0000122 GO:0071371 GO:0048645 GO:0000979 GO:0060510 GO:0005667 GO:0008270 GO:0007493 GO:0005634 GO:0060430 GO:0001889 GO:0003310 GO:0060045 GO:0060947 GO:0001701 GO:0071158 GO:0006644

XM_018053075.1 GO:0031209 GO:0005856 GO:0048365 GO:0005741 GO:0030027 GO:0016601 GO:0003779 GO:2000601 GO:0072673 GO:0032403

XM_018060916.1 GO:0046872 GO:0003676

XM_005678515.3 GO:0006493 GO:0016021 GO:0047223 GO:0000139

XM_005692396.3 GO:0010390 GO:0016020 GO:0031062 GO:0071222 GO:0030054 GO:0045638 GO:0006378 GO:0005737 GO:0000993 GO:0000122 GO:0032968 GO:0003682 GO:0016593 GO:0001711 GO:0016584 GO:0033523 GO:0019827 GO:0031442 GO:0034504

XM_018064545.1 GO:0005783 GO:0035418 GO:0030054 GO:0032281 GO:0030863 GO:0097110 GO:0060997 GO:0044300 GO:0031234 GO:0016188 GO:0019900 GO:0035255 GO:0044224 GO:0043197 GO:0045211 GO:0045184 GO:2000821 GO:0035176 GO:0008021 GO:0035641 GO:0014069 GO:0008022 GO:0044306 GO:0048169 GO:0050885 GO:0006461 GO:0071625

XM_018058590.1 GO:0071456 GO:0005874 GO:0032287 GO:0043015 GO:0055038 GO:0005913 GO:0045576 GO:0017137 GO:0005886 GO:0008285 GO:0030330 GO:0070062 GO:0005634 GO:0043209 GO:0005813 GO:0008017 GO:0048471 GO:0045296 GO:0090232

XM_005676250.3 GO:0031012

XM_018066399.1 GO:0016787

XM_018039762.1 GO:0004798 GO:0005524 GO:0046939 GO:0006233

XM_018043382.1 GO:0009952 GO:0045666 GO:0051057 GO:0043497 GO:0005887 GO:0021987 GO:0007224 GO:0002088 GO:0014816 GO:0007520 GO:0060059 GO:2000179 GO:0010172 GO:0001708 GO:0048643 GO:0045944 GO:0043410 GO:0045663

XM_018050649.1 GO:0008270

XM_018063108.1 GO:0009644 GO:0005739 GO:0010842 GO:0042462

XM_018055277.1 GO:0046872

NM_001285750.1 GO:0001649 GO:0008286 GO:0043491 GO:0032436 GO:0042593 GO:0090201 GO:0060709 GO:0010748 GO:0001893 GO:0042802 GO:0010765 GO:0045944 GO:0045429 GO:0033138 GO:0043536 GO:0046889 GO:0031659 GO:0005524 GO:0048009 GO:0030307 GO:0032287 GO:0036064 GO:0051091 GO:0001938 GO:0005739 GO:0043325 GO:0005886 GO:0004712 GO:0071364 GO:0006954 GO:0006469 GO:0097194 GO:0004674 GO:0010507 GO:0043234 GO:0072655 GO:0010975 GO:0043154 GO:0030235 GO:0010763 GO:0031641 GO:0072656 GO:0000060 GO:0031982 GO:0019901 GO:0070141 GO:0046326 GO:0005911 GO:0006924 GO:1990418 GO:0031663 GO:0090004 GO:0045600 GO:0018107 GO:0005547 GO:0007281 GO:0045725 GO:0071380 GO:0032079 GO:0034405 GO:0051146 GO:0051000 GO:0016567 GO:0032094 GO:0035924 GO:0010629 GO:1990090 GO:1990314 GO:0071889 GO:0005829 GO:0005819 GO:0060416 GO:0031999 GO:1900182 GO:0005634 GO:0060716 GO:0006417 GO:0036294 GO:0097011

XR_001918998.1 GO:0005911

XM_005682256.3 GO:0006406 GO:0016020 GO:0010468 GO:0000398 GO:0044822 GO:0005524 GO:0005737 GO:0005681 GO:0004004 GO:0010501 GO:0006974

NM_001286975.1 GO:0003796 GO:0035036 GO:0005576 GO:0001669 GO:0005764

XM_018053316.1 GO:0010468 GO:0043547 GO:0030193 GO:0017157 GO:0030141 GO:0050708 GO:0016021 GO:0031201 GO:0019905 GO:0005886 GO:0017137 GO:0005096

XM_005691340.3 GO:0030729 GO:0006629

XM_018050409.1 GO:0000122 GO:0000978 GO:0046872 GO:0005634 GO:0007212 GO:0001078

XM_018042238.1 GO:0070062 GO:0016020 GO:0030246 GO:0005829

XM_018051489.1 GO:0006396 GO:0044822

XM_018039958.1 GO:0006506 GO:0017176 GO:0016021

XM_018038746.1 GO:0045892 GO:0042802 GO:0043621 GO:0016363 GO:0005737 GO:0042405 GO:0042272 GO:0051168 GO:0008022 GO:0008266 GO:0034046

XM_013975655.2 GO:0008117 GO:0005783 GO:0048008 GO:0001822 GO:0040014 GO:0097190 GO:0007283 GO:0006672 GO:0009791 GO:0006631 GO:0016021 GO:0060021 GO:0001570 GO:0030170 GO:0030097 GO:0030149 GO:0048705 GO:0008209 GO:0010761 GO:0016831 GO:0033327 GO:0001553 GO:0008210 GO:0060325

XM_018049609.1 GO:0000902 GO:0051015 GO:0002064 GO:0001843

XM_018047367.1 GO:0070062 GO:0005765 GO:0016021 GO:0032418 GO:0048813

XM_013976805.2 GO:0005525

XM_018038243.1 GO:0035368 GO:0005634 GO:0003746 GO:0001514 GO:0000049 GO:0003924 GO:0030529 GO:0005525 GO:0005739 GO:0043021

XM_018039838.1 GO:0003723 GO:0005737

XM_018044819.1 GO:0006396 GO:0044822 GO:0001510 GO:0008173

XM_018045549.1 GO:0015175 GO:0016324 GO:0031526 GO:0016021 GO:0003333 GO:0015804 GO:0015184 GO:0015811

XM_018049285.1 GO:0008270 GO:0055114 GO:0016491

XM_005694534.2 GO:0033179 GO:0015991 GO:0016021 GO:0016049 GO:0046961

XM_005695937.3 GO:0003729 GO:0005654 GO:0000381 GO:0043065 GO:0005681 GO:0000166 GO:0008270 GO:0000245

NM_001287574.1 GO:0006704 GO:0016709 GO:0020037 GO:0008203 GO:0005506 GO:0055114 GO:0071375 GO:0034650 GO:0006700 GO:0044550 GO:0005743 GO:0008386 GO:0042359

XM_018040951.1 GO:0055085 GO:0005634 GO:0005524 GO:0042626 GO:0016021 GO:0005886

XM_005691342.3 GO:0005634 GO:0005737

XM_018065555.1 GO:0005737

XM_018063805.1 GO:0003677 GO:0005524 GO:0006260 GO:0034244 GO:0005694 GO:0032508 GO:0005737 GO:0007067 GO:0000993 GO:0051304 GO:0009378 GO:0043140 GO:0000724 GO:0016591

XM_018061700.1 GO:0070062 GO:0060349 GO:0005578 GO:0000139 GO:0010955 GO:0030512 GO:0016021 GO:0017134 GO:0032330

XM_018042044.1 GO:0001964 GO:0016021 GO:0042593

XM_018052067.1 GO:0016020

XM_005686879.3 GO:0003677 GO:0046872 GO:0005634 GO:0006355

XM_018050524.1 GO:0003677 GO:0005634 GO:0006355

XM_018048623.1 GO:0005887 GO:0007166

XM_018066183.1 GO:0035197 GO:0070578 GO:0005524 GO:0004525 GO:0070883 GO:0004386 GO:0003725 GO:0035087 GO:0033168 GO:0019904 GO:0090502 GO:0030423 GO:0035280 GO:0031054

XM_005686919.3 GO:0046835 GO:0070062 GO:0004454 GO:0005737

XM_018056661.1 GO:0060348 GO:0003677 GO:0005654 GO:0030901 GO:0006468 GO:0030509 GO:0051216 GO:0005737 GO:0060395 GO:0007179 GO:0071407 GO:0005667 GO:0001657 GO:0003700 GO:0030902

XM_018049473.1 GO:0050714 GO:0048015 GO:0005737 GO:0098592

XM_005691457.3 GO:0004407 GO:0021762 GO:0042826 GO:0016580 GO:0005737 GO:0000122 GO:0043065 GO:0016575 GO:0016568

XM_018039612.1 GO:0005634 GO:0007165 GO:0009409 GO:0060612 GO:0006366 GO:0045893 GO:0046872 GO:0050873 GO:0000987 GO:0033613 GO:0003700

XM_005679262.2 GO:0046872 GO:0006355 GO:0043565 GO:0003700

XM_018045944.1 GO:0016021 GO:0005737

XM_005679100.3 GO:0005737

XM_005675392.3 GO:0003677 GO:0005634 GO:0019904 GO:0002318 GO:0006351 GO:0007050 GO:0005737

XM_018050792.1 GO:0005794 GO:0016021 GO:0005886

XM_018054475.1 GO:0043154 GO:0010751 GO:0010763 GO:0009897 GO:0032026 GO:0034976 GO:0030823 GO:0051918 GO:0002581 GO:0018149 GO:0010748 GO:0005178 GO:0009749 GO:0008201 GO:0045766 GO:0001786 GO:0070051 GO:0001937 GO:0070062 GO:0070052 GO:0042493 GO:0043032 GO:2001027 GO:0040037 GO:0043652 GO:0002605 GO:0051592 GO:1902043 GO:0050921 GO:0002544 GO:0001953 GO:0017134 GO:0043536 GO:0002040 GO:0051897 GO:0006986 GO:0005577 GO:0001968 GO:0032695 GO:0045727 GO:0007050 GO:0016529 GO:2000353 GO:0031012 GO:0010754 GO:0000187 GO:2000379 GO:0030169 GO:0016525 GO:0010757 GO:0043236 GO:0042535 GO:0048266 GO:0031091 GO:0005509 GO:0006955 GO:0043537 GO:0030511

XM_013965370.2 GO:0030324 GO:0005615 GO:0007507 GO:0030199 GO:0055114 GO:0048251 GO:0005578 GO:0005507 GO:0005581 GO:0004720 GO:0035904

XM_005697416.2 GO:0005654 GO:0000781 GO:0000445 GO:0043066 GO:0046784 GO:0007417

XM_018044115.1 GO:0007156 GO:0005509 GO:0016021 GO:0005886

XM_018042922.1 GO:0005765 GO:0000220 GO:0016324 GO:0070072 GO:0051117 GO:0007035 GO:0015986 GO:0015991 GO:0007039 GO:0016021 GO:0005739 GO:0046961 GO:0016236

XM_018048287.1 GO:0016020 GO:0044822 GO:0001731 GO:0016282 GO:0071541 GO:0006446 GO:0003743 GO:0001732 GO:0033290

XM_018062560.1 GO:0070062 GO:0015266 GO:0005654 GO:0005742 GO:0030150 GO:0032592

XM_018042414.1 GO:0016324 GO:0006970 GO:0034465 GO:0060083 GO:0043065 GO:0005901 GO:0030007 GO:0060072 GO:0042391 GO:0008076 GO:0070062 GO:0005249 GO:0045794 GO:0034765 GO:0001666 GO:0003779 GO:0046872 GO:0051592 GO:0071805

XM_018061507.1 GO:0016787

XM_005688519.3 GO:0042110 GO:0005070 GO:0005770 GO:0047485 GO:0016023 GO:0000122 GO:0009967 GO:0005886 GO:0050849

XM_013962531.2 GO:0005615 GO:0035279 GO:0044822 GO:0005524 GO:0004386 GO:0000932

XM_018047665.1 GO:0070062 GO:0016020 GO:0075522 GO:0005654 GO:0000398 GO:0044822 GO:0005925 GO:0031625 GO:0050687 GO:0043161 GO:0039694 GO:0005829 GO:0030529 GO:0032480 GO:0045087

XM_018047457.1 GO:0016020 GO:0005938 GO:0001757 GO:0043025 GO:0030041 GO:0048669 GO:0033504 GO:0000578 GO:0051639 GO:0044295 GO:1900006 GO:0001889 GO:0044294 GO:0030903 GO:0003785 GO:0048565 GO:1900029 GO:0048471 GO:0001726 GO:0005884 GO:0001843

XM_005676450.2 GO:0005212

XM_013970632.2 GO:0045859 GO:0031588 GO:0005634 GO:0007165 GO:0004672 GO:0006633 GO:0010628

XM_018064720.1 GO:0034551 GO:0005743

XM_005681618.3 GO:0044822 GO:0005847

XM_018054655.1 GO:0005615 GO:0045202

XM_018039244.1 GO:0005615 GO:0005794 GO:0046658

XM_005685737.3 GO:0030331 GO:0005634 GO:0007368 GO:0036158 GO:0033146 GO:0005737 GO:0061136 GO:0003341 GO:0036159 GO:0005886

XM_018050499.1 GO:0004252 GO:0070062 GO:0005615 GO:0006508 GO:0006957

NM_001285731.1 GO:0071456 GO:0020037 GO:0050433 GO:0006121 GO:0048039 GO:0006099 GO:0005749 GO:0000104 GO:0046872 GO:0016021

XM_018045753.1 GO:0005615 GO:0060317 GO:0009897 GO:0034695 GO:0035556 GO:0015026 GO:0043235 GO:0007179 GO:0034673 GO:0016021 GO:0050431 GO:0070062 GO:0005114 GO:0005622 GO:0030509 GO:0032354 GO:0034699 GO:0060389 GO:0006955 GO:0070123 GO:0046332 GO:0051271

XM_018044324.1 GO:0007165 GO:0005230 GO:0034220 GO:0030054 GO:0016021 GO:0004890 GO:0005886 GO:0045202

XM_018059141.1 GO:0070062 GO:0007264 GO:0016021 GO:0005622 GO:0043547 GO:0005096

XM_018064326.1 GO:0016323 GO:0046982 GO:0044291 GO:0010629 GO:0001931 GO:1902992 GO:0090002 GO:0030864 GO:0005913 GO:0001765 GO:0002080 GO:0070062 GO:0005925 GO:0045661 GO:0030027 GO:0048471 GO:0044860 GO:0005768 GO:0030139 GO:0016600 GO:0050821

XM_005695919.3 GO:0016021

XM_018050742.1 GO:0005856 GO:0005515 GO:0043209 GO:0005525 GO:0005737

XM_018056878.1 GO:0001937 GO:0032780 GO:0042030 GO:0051289 GO:0043532 GO:0051117 GO:0042803 GO:0030272 GO:0030218 GO:0009396 GO:0006783 GO:0009986 GO:0035999 GO:0005739 GO:0005516

XM_018052588.1 GO:0051482 GO:0010977 GO:0005887 GO:0060326 GO:0005829 GO:0030165 GO:0035727 GO:0007193 GO:0032060 GO:0000187 GO:0008360 GO:0070915 GO:0005768 GO:0043123 GO:0051496 GO:0009986 GO:0030139 GO:0035025 GO:0007202

XM_018042034.1 GO:0044295 GO:0051491 GO:0043025 GO:0030175 GO:0048863 GO:0007416 GO:0003407 GO:0048812 GO:0001764 GO:0016021 GO:0043197 GO:0005886

XM_018047427.1 GO:0044822

XM_018061644.1 GO:0000902 GO:0016020 GO:0005634 GO:0005737 GO:0000122 GO:0008340 GO:0007431 GO:0007028 GO:0002070 GO:0045927

XM_013963942.2 GO:0006397 GO:0004535 GO:0005634 GO:0031251 GO:0000932 GO:0003676 GO:0000289 GO:0010606 GO:0090503

XM_018038374.1 GO:0051018 GO:0042470 GO:0001750 GO:0006886 GO:0003779 GO:0000145 GO:0015629 GO:0017022 GO:0017137 GO:0045202

XM_005675434.3 GO:0007186 GO:0070062 GO:0016021 GO:0004930

XM_005682272.3 GO:0032434 GO:0005634 GO:0003684 GO:0031593 GO:0045070 GO:1990381 GO:0006289

XM_013970253.2 GO:0005623 GO:0045454

XR_001919894.1 GO:0006820 GO:0016020 GO:0005216 GO:0034220

XM_018052542.1 GO:0016323 GO:0009897 GO:0016324 GO:0071222 GO:0034188 GO:0005102 GO:0045332 GO:0005887 GO:0030819 GO:0098656 GO:0055091 GO:0055098 GO:0048471 GO:0071300 GO:0038027 GO:0045121 GO:0033700 GO:0032489 GO:0043691 GO:0034186 GO:0005548 GO:0031267 GO:0010875 GO:0050702 GO:0006911 GO:0005524 GO:0008203 GO:0051117 GO:0002790 GO:0045335 GO:0042632 GO:0034616 GO:0007040 GO:0016197 GO:0007186 GO:0042626 GO:0017127 GO:0034380 GO:0060155 GO:0032367 GO:0006497 GO:0005794 GO:0019905 GO:0008509

XM_018042239.1 GO:0070062 GO:0016020 GO:0030246 GO:0005829

XM_018063797.1 GO:0031994 GO:0031252 GO:0043588 GO:0008305 GO:0072001 GO:0035878 GO:0048870 GO:0006914 GO:0007229 GO:0048333 GO:0038132 GO:0070062 GO:0009611 GO:0005634 GO:0004872 GO:0048565 GO:0031581 GO:0097186 GO:0007160 GO:0009986 GO:0001664 GO:0030198 GO:0030056

XM_005677656.3 GO:0005615 GO:0051603 GO:0001968 GO:1903146 GO:0005764 GO:0043394 GO:0005518 GO:0004197 GO:0030574

XM_005693991.3 GO:0000453 GO:0044822 GO:0000466 GO:0030687 GO:0005730 GO:0008650 GO:0016435 GO:0030688 GO:0000463

XM_018050549.1 GO:0005634 GO:0008270 GO:0005737

XM_018066357.1 GO:0016021 GO:1902616 GO:0005739 GO:0015227

XM_005677486.3 GO:0007093 GO:0010212 GO:0006281 GO:0016180 GO:0032039 GO:0070876

XM_018043947.1 GO:0007186 GO:0070062 GO:0009986 GO:0016021 GO:0007166 GO:0004930

XR_001917272.1 GO:0007059 GO:0006334 GO:0000139 GO:0004596 GO:0043967 GO:0017196 GO:0008283 GO:0010485 GO:0016568

XM_018057201.1 GO:0005911

XM_018067229.1 GO:0005887 GO:0003333 GO:0005369 GO:0005328 GO:0006836 GO:0015734

XM_018054513.1 GO:0003779 GO:0016567 GO:0015629 GO:0031463 GO:0004842

XM_018066256.1 GO:0010452 GO:0042800 GO:0051568 GO:0000790 GO:0051149 GO:0003713 GO:0046975 GO:0045944 GO:0001102

XM_013964626.2 GO:0005737

XM_018065529.1 GO:0071481 GO:0047485 GO:0007420 GO:0045778 GO:0032116 GO:0003682 GO:0048557 GO:0061038 GO:0070062 GO:0042634 GO:0042826 GO:0048703 GO:0035261 GO:0003151 GO:0050890 GO:0019827 GO:0008022 GO:0045944 GO:0034613 GO:0070087 GO:0034088 GO:0048592 GO:0031065 GO:0000122 GO:0006974 GO:0036033 GO:0000785 GO:0045995 GO:0035115 GO:0042471 GO:0061010 GO:0007605 GO:0045444 GO:0040018 GO:0060325

XM_005684222.3 GO:0071456 GO:0005783 GO:0031593 GO:1902175 GO:0035973 GO:0019900 GO:0005776 GO:0005886 GO:2000785 GO:0005654 GO:0016235 GO:0042802 GO:1901097 GO:1901340 GO:0031398 GO:0048471 GO:0043234 GO:1903071 GO:0034140

XM_018056700.1 GO:0007595 GO:0016323 GO:0006878 GO:0005524 GO:0005770 GO:0016023 GO:0032588 GO:0005887 GO:0005507 GO:0004008 GO:0015680 GO:0051208 GO:0006882 GO:0046688 GO:0048471 GO:0060003 GO:0015677

XM_018045481.1 GO:0007186 GO:0050911 GO:0004984 GO:0016021 GO:0005886 GO:0004930

XM_018061386.1 GO:0003677 GO:0005654

XM_018047068.1 GO:0070062 GO:0005886

XM_005680155.3 GO:0005654 GO:0044822 GO:0005794

XM_018062970.1 GO:0046872 GO:0006355 GO:0005622 GO:0003676

XM_005679249.3 GO:0003839 GO:0005829 GO:0006750 GO:0042803 GO:0001836

XM_013970799.2 GO:0005856 GO:0005615 GO:0030054 GO:0014069 GO:0008139 GO:0048471 GO:0000139 GO:0005509 GO:0048306 GO:0042308 GO:0045211

XM_018061543.1 GO:0045892 GO:0006357 GO:0005654 GO:0043565 GO:0003700 GO:0005737 GO:0045893

XM_018059692.1 GO:0006089 GO:0001780 GO:0042593 GO:0008202 GO:0005977 GO:0015760 GO:0005623 GO:0042632 GO:0016021 GO:0002318 GO:0006874 GO:0043085 GO:0006820 GO:0055085 GO:0030593 GO:0006641 GO:0032682 GO:0045730 GO:0035166 GO:0022857

XM_018057862.1 GO:0005634 GO:0042995 GO:0014066 GO:1903589 GO:0035307 GO:1902309 GO:0050790 GO:0048471 GO:0008599 GO:0061028 GO:0005886 GO:0051489

XM_018041732.1 GO:0031649 GO:0046982 GO:0043547 GO:0051379 GO:0005887 GO:0040015 GO:0030165 GO:0030819 GO:0005769 GO:0043950 GO:0001996 GO:0002024 GO:0005057 GO:0009409 GO:0007267 GO:0001997 GO:0051380 GO:0071880 GO:0005088 GO:0050873 GO:0031694 GO:0002025 GO:0042596 GO:0004940

XM_005676356.3 GO:0003688 GO:0016020 GO:0000784 GO:0006260 GO:0005664 GO:0005813 GO:0000939 GO:0000792

XM_018052836.1 GO:0003676

XM_018046968.1 GO:0008083 GO:0005615 GO:0006111 GO:2000366 GO:0042102 GO:0001890 GO:0032310 GO:0005737 GO:0042593 GO:0050796 GO:0005179 GO:0070093 GO:0032760 GO:0032615 GO:0046850 GO:0032817 GO:0006909 GO:0007260 GO:1900015 GO:0030300 GO:0014068 GO:0050810 GO:0072606 GO:0001936 GO:0051726 GO:0030217 GO:0090335 GO:0008206 GO:0051897 GO:0042445 GO:0008203 GO:0048639 GO:0002021 GO:0032008 GO:0038108 GO:0000122 GO:0019953 GO:0006635 GO:0046325 GO:0032868 GO:0042755 GO:0021954 GO:0045639 GO:0060612 GO:0042269 GO:0072604 GO:1990051 GO:1900745 GO:0045765 GO:0010507 GO:0008343 GO:0050999

XM_018066738.1 GO:0016525 GO:0030169 GO:0007267 GO:0042742 GO:0005540 GO:0007155 GO:0005041 GO:0005887 GO:0005044 GO:0006898 GO:0005509

XM_018046735.1 GO:0005925 GO:0008270 GO:0005737

XM_005676440.2 GO:0005212 GO:0034614 GO:0007601

XM_005680475.3 GO:0000922 GO:0005654 GO:0005814 GO:0036064 GO:0005730 GO:0000242 GO:0016021 GO:0045177 GO:0005886

XM_013972744.2 GO:0007186 GO:0005634 GO:0009968 GO:0005886 GO:0005737

XM_018043736.1 GO:0016020 GO:0070062 GO:0005654 GO:0006284 GO:0044822 GO:0006513 GO:0005737 GO:0004842 GO:0016874 GO:0016574 GO:0000209

XM_018060773.1 GO:0016021

XM_005680908.2 GO:0030514 GO:0008083 GO:0032525 GO:0002021 GO:0005737 GO:0045605 GO:0019901 GO:0090009 GO:0005576 GO:0048859 GO:0007492 GO:0010453 GO:0007498 GO:0001654 GO:0030903 GO:0001701 GO:0045662 GO:0001501

XM_018055678.1 GO:0047485 GO:0055114 GO:0071889 GO:0007264 GO:0005829 GO:0005739 GO:0034766 GO:0030659 GO:0070062 GO:0006605 GO:0045892 GO:0021762 GO:1900740 GO:0005925 GO:0044325 GO:0008022 GO:0019904 GO:0043234 GO:0004497

NM_001314231.1 GO:0046982 GO:0001077 GO:0003705 GO:0005737 GO:0019901 GO:0000122 GO:0071277 GO:0003682 GO:0005667 GO:0033613 GO:0061337 GO:0005654 GO:0000978 GO:0042826 GO:0001105 GO:0070375 GO:0048311 GO:0055005 GO:0000790 GO:0001085 GO:0045944 GO:0000002 GO:0046332 GO:0035035

XM_018052537.1 GO:0046872 GO:0006355 GO:0005622 GO:0002230 GO:0003676

XM_018050989.1 GO:0050731 GO:0071456 GO:0007171 GO:0051005 GO:0030297 GO:0070328 GO:0004857 GO:0045766 GO:0072562 GO:0030154 GO:0016525 GO:0030971 GO:2000352 GO:0005578 GO:0007219 GO:0043537 GO:0043536

XM_018046761.1 GO:0005829 GO:0005525

XM_005693519.2 GO:0031100 GO:0016020 GO:0005524 GO:0000339 GO:0003725 GO:0003743 GO:0005515 GO:0000289 GO:0005829 GO:0070062 GO:0031012 GO:0005634 GO:0003729 GO:0016032 GO:0006446 GO:0004004 GO:0010501 GO:0016281

XM_018061743.1 GO:0072332 GO:0090575 GO:0001649 GO:0030178 GO:0001105 GO:0005902 GO:0071560 GO:0045944 GO:2001241 GO:0005794 GO:0005829 GO:0005739 GO:0005886 GO:0019899 GO:0048705

XM_018041133.1 GO:0045165 GO:0030182 GO:0048645 GO:0048536 GO:0045944 GO:0046982 GO:0048535 GO:0007417 GO:0008284

XM_018056959.1 GO:0046835 GO:0006003 GO:0004331 GO:0005654 GO:0005524 GO:0006000 GO:0016311 GO:0003873

XM_018054712.1 GO:0072356 GO:0048365 GO:1990023 GO:0010762 GO:0019901 GO:0030496 GO:1900027 GO:0007229 GO:0005886 GO:0045184 GO:0005634 GO:0034506 GO:0044822 GO:1900025 GO:0008017 GO:0051895 GO:0031901 GO:0051987 GO:0019904 GO:0010971 GO:0034260

XM_005698484.3 GO:2001236 GO:0005783 GO:0005730 GO:0004467 GO:0005743 GO:0016021 GO:0001676

XM_018045949.1 GO:0035092 GO:0000801 GO:0001673 GO:0032880 GO:0000711 GO:0051878 GO:0051026 GO:0000802

XM_005679971.3 GO:0051015 GO:0031529 GO:0005925 GO:0005903 GO:0030835 GO:0003785 GO:0005737 GO:0001725 GO:0032154 GO:0051017 GO:0008270 GO:0005886

XM_018046884.1 GO:0005654 GO:0005615

NM_001314266.1 GO:0010839 GO:0042393 GO:0070062 GO:0046697 GO:0016540 GO:0005615 GO:0051603 GO:0031410 GO:0005764 GO:2000249 GO:0031069 GO:0005730 GO:0016807 GO:0004197

XM_018039624.1 GO:0007268 GO:0014069

XM_005687987.3 GO:0070588 GO:1990454 GO:0070509 GO:0007268 GO:0007601 GO:0008331 GO:0051928

XM_018048863.1 GO:0016020 GO:0009306 GO:0005829 GO:0005794 GO:0043547 GO:0005096

XM_013968548.2 GO:0005654 GO:0004795 GO:0009088 GO:0005739 GO:0030170

XM_018053442.1 GO:0010468 GO:0004709 GO:0005524 GO:0060718 GO:0051973 GO:0001890 GO:0043507 GO:0005737 GO:0010225 GO:0019100 GO:1900745 GO:0032212 GO:0000186

XM_018051860.1 GO:0008375 GO:0005783 GO:0015012 GO:0016021 GO:0006024

XM_018050779.1 GO:0007186 GO:0070062 GO:0005834 GO:0004871

XR_001918006.1 GO:0008188 GO:0005887 GO:0005000 GO:0007218 GO:0051281 GO:0005737

XM_013974253.2 GO:0043234 GO:0008270

XM_005680249.3 GO:0061649 GO:0004843 GO:0005634 GO:0042802 GO:0030509 GO:0035616 GO:0005160 GO:0005737 GO:0007179 GO:0006511 GO:0060389 GO:0004197 GO:0035520 GO:0046332

XM_018050172.1 GO:2000273 GO:0030546 GO:0030331 GO:0034614 GO:0045672 GO:0001503 GO:0006390 GO:0007015 GO:0051091 GO:0060346 GO:0000166 GO:0005739 GO:0030520 GO:0045892 GO:0050682 GO:0016592 GO:0001104 GO:0030374 GO:0003676 GO:0045780 GO:0042327 GO:0045944 GO:0010694 GO:0008134

XM_005683021.3 GO:0032481 GO:0002218 GO:0031625 GO:0005741 GO:0005777 GO:0061507 GO:0032092 GO:0019901 GO:0035458 GO:0016021 GO:0005886 GO:0033160 GO:0071360 GO:0035438 GO:0042803 GO:0006915 GO:0048471 GO:0032608 GO:0045944 GO:0005794 GO:0005789 GO:0008134 GO:0002230 GO:0042993

XM_018059757.1 GO:0033306 GO:0019166 GO:0005102 GO:0005739 GO:0005777 GO:0055114

XM_018056134.1 GO:0004381 GO:0004380 GO:0005975 GO:0016021

XM_005680310.3 GO:0005637 GO:0016021

XM_018044671.1 GO:0016020 GO:0010468 GO:0007059 GO:0005524 GO:0003676 GO:0006413 GO:0004004 GO:0036464 GO:0010501

XM_018048060.1 GO:0042393 GO:0001164 GO:0005654 GO:0008270 GO:0005737

XM_018039798.1 GO:0005730

XM_018039546.1 GO:0004471 GO:1902031 GO:0046872 GO:0051287 GO:0005739 GO:0055114 GO:0006108

XM_005689506.3 GO:0007155 GO:0030198 GO:0016021 GO:0042633

XM_018043492.1 GO:0016020 GO:0003777 GO:0005871

XM_005678719.3 GO:0008270 GO:0046983 GO:0003676

XM_005685557.3 GO:0042802 GO:0090502 GO:0005576 GO:0004522 GO:0003676

XM_018038828.1 GO:0016021

XM_018041512.1 GO:0030120

XM_018064594.1 GO:0046875 GO:0007628 GO:0016198 GO:0048013 GO:0016021 GO:0031295

XM_018056982.1 GO:0006376 GO:0003723 GO:0005634 GO:0000166 GO:0005737

XM_013974025.2 GO:0003676

XM_018039373.1 GO:0019367 GO:0009922 GO:0005783 GO:0030148 GO:0016021 GO:0042761 GO:0034625

XM_005680514.3 GO:0001824 GO:0000981 GO:0005634 GO:0030154 GO:0045944 GO:0043565

XM_018039095.1 GO:0000122 GO:0046872 GO:0005634 GO:0001206 GO:0000980 GO:0005739

XM_005680250.3 GO:0061649 GO:0004843 GO:0005634 GO:0042802 GO:0030509 GO:0035616 GO:0005160 GO:0005737 GO:0007179 GO:0006511 GO:0060389 GO:0004197 GO:0035520 GO:0046332

XM_018054072.1 GO:0046872 GO:0003676

XM_018056664.1 GO:0000124 GO:0035948 GO:0003712

XM_018056763.1 GO:0046872 GO:0044822 GO:0016607

XM_018039400.1 GO:0045666 GO:0003714 GO:0046982 GO:0001093 GO:0043621 GO:0001077 GO:0000122 GO:0003682 GO:0005667 GO:0016021 GO:1900746 GO:0065004 GO:0005634 GO:0000978 GO:0070888 GO:0016525 GO:0001011 GO:0045944 GO:0001087 GO:0042118 GO:0006367

XM_018052651.1 GO:0046872 GO:0003676

XM_018060429.1 GO:0051015 GO:0007626 GO:0051491 GO:0005903 GO:0030046 GO:0032426 GO:0015629 GO:0051494

XM_018057590.1 GO:0070129 GO:0000287 GO:0005761 GO:0005743 GO:0005525 GO:0044065 GO:0003924

XM_018049587.1 GO:0005634 GO:0005770 GO:0005794 GO:0016021 GO:0005886 GO:0005769

XM_005691815.3 GO:0070198 GO:0051092 GO:1901985 GO:0005635 GO:0031848 GO:0010569 GO:0005737 GO:0043123 GO:0042162 GO:0010833 GO:0048239 GO:0030870 GO:0033138 GO:0070187 GO:0032205

XM_013962599.2 GO:0005980 GO:0005634 GO:0031593 GO:0030247 GO:0004134 GO:0005737 GO:0016234 GO:0004135 GO:0005978

XM_018060668.1 GO:0016020 GO:0005654 GO:0005813 GO:0034453 GO:0008017 GO:0005737

NM_001314257.1 GO:0019367 GO:0009922 GO:0005515 GO:0042759 GO:0030176 GO:0035338

XM_018047636.1 GO:0008542 GO:0001505 GO:0005737 GO:0042053 GO:0048101 GO:0005516 GO:0004117 GO:0007165 GO:0046069 GO:0036006 GO:0006198 GO:0007626 GO:0046872 GO:0001975 GO:0042428 GO:0097011 GO:0030224

XM_013966480.2 GO:0000287 GO:0007252 GO:0005524 GO:0006355 GO:0005671 GO:0008385 GO:0097110 GO:0032743 GO:0005886 GO:0000186 GO:0004709 GO:0000187 GO:0007250 GO:0043507 GO:0006915 GO:0043123 GO:0043966 GO:0002726

XM_005679323.3 GO:0005759 GO:0021766 GO:0006096 GO:0006099 GO:0005829 GO:0021794 GO:0030976 GO:0021860 GO:0061034 GO:0022028 GO:0046872 GO:0045252 GO:0021695 GO:0034602 GO:0004591 GO:0021756

XM_018044851.1 GO:0072686 GO:0005827 GO:0006513 GO:0005813 GO:0007094 GO:0005737 GO:0004842 GO:0051301 GO:0031463

XM_018052045.1 GO:0001771 GO:0016020 GO:0031252 GO:0036336 GO:0005622 GO:0043547 GO:0005085 GO:0061485 GO:0007264 GO:0070233

XM_018048792.1 GO:0090141 GO:0016021 GO:0005739

XM_018046199.1 GO:0016020 GO:0001649 GO:0002244 GO:1900369 GO:0005737 GO:0005730 GO:0005515 GO:0045071 GO:0060216 GO:0044530 GO:0006611 GO:0035280 GO:0005654 GO:0003726 GO:0044822 GO:0051607 GO:0043066 GO:0001701 GO:0030218 GO:0035455 GO:0006382 GO:0061484 GO:0006606 GO:0045070 GO:0044387 GO:0002566 GO:0098586 GO:0060339 GO:0031054

XM_013968612.2 GO:0010951 GO:0005576 GO:0004869

XM_018046650.1 GO:0043589 GO:0006338 GO:0045747 GO:0001077 GO:0005737 GO:0030859 GO:2000381 GO:0045617 GO:0003682 GO:0005654 GO:0007499 GO:0051289 GO:0060529 GO:0042802 GO:0042771 GO:0043066 GO:0002053 GO:0000790 GO:0042475 GO:2001235 GO:0000989 GO:0003684 GO:0031069 GO:0043281 GO:0045944 GO:0061436 GO:0009954 GO:0060197 GO:2000773 GO:0060157 GO:2000271 GO:0060513 GO:0010259 GO:0048485 GO:0002064 GO:0051402 GO:0048745 GO:0036342 GO:0000122 GO:0050699 GO:0043565 GO:0048807 GO:0010838 GO:0030326 GO:0044212 GO:0045669 GO:0001736 GO:1902808 GO:0001501 GO:0010482

XM_005687443.3 GO:0050731 GO:0051378 GO:0050795 GO:0014065 GO:0007210 GO:0045821 GO:0005887 GO:0008144 GO:0071886 GO:0042493 GO:0004993 GO:0010513 GO:0046883 GO:0044380 GO:0045600 GO:0005622 GO:0006939 GO:0043198 GO:0007202 GO:0033674 GO:0070374 GO:0051209

XM_018044886.1 GO:0005856

XM_018056961.1 GO:0042393 GO:0005654 GO:0006355 GO:0016235 GO:0010629

XM_018043204.1 GO:0016477 GO:0014043 GO:0050321 GO:0006349 GO:0021766 GO:0031625 GO:0030877 GO:0071109 GO:0032436 GO:0032092 GO:0043025 GO:0030426 GO:0032091 GO:0036016 GO:0001837 GO:2000738 GO:0035729 GO:0043066 GO:0001085 GO:0046777 GO:0048471 GO:0010800 GO:0031334 GO:1901030 GO:0070059 GO:0045944 GO:0033138 GO:0006983 GO:0031333 GO:0051534 GO:0097192 GO:0001954 GO:0005524 GO:0043547 GO:0000320 GO:0045773 GO:0005977 GO:0010977 GO:0005829 GO:0035372 GO:0005739 GO:0044027 GO:0005886 GO:0032886 GO:0046827 GO:0007623 GO:0005634 GO:0002039 GO:0007520 GO:0005813 GO:0044337 GO:0010614 GO:0043198 GO:0009887 GO:0004674 GO:0051059 GO:0045444 GO:0030529 GO:0034236 GO:0008013

XM_005680810.3 GO:0020037 GO:0005737

XM_005682183.3 GO:0005762 GO:0070131 GO:0005743 GO:0016021 GO:0061668

XM_018049872.1 GO:1903758 GO:0050872 GO:0055114 GO:0031065 GO:0000122 GO:0017053 GO:0005667 GO:0003700 GO:0005654 GO:0016616 GO:0090241 GO:0070491 GO:0051287 GO:0042803 GO:0001106 GO:0019904 GO:0051726

XM_013962705.2 GO:0007186 GO:0030246 GO:0016021 GO:0007166 GO:0004930

XM_005696626.3 GO:0005515 GO:0005637 GO:0016021

XM_018061157.1 GO:0048008 GO:0048011 GO:0005737 GO:0042593 GO:0007173 GO:0033277 GO:0060020 GO:0005158 GO:0035265 GO:0021697 GO:0000077 GO:0048013 GO:0032528 GO:0007409 GO:0051428 GO:0061582 GO:0048806 GO:0036302 GO:2001275 GO:0042445 GO:0035335 GO:0040014 GO:0051463 GO:0048873 GO:0004726 GO:0030220 GO:0007229 GO:0048839 GO:0009755 GO:0060125 GO:0005070 GO:0005634 GO:0000187 GO:0035855 GO:0033629 GO:0043254 GO:0006641 GO:0030971 GO:0045931 GO:0046825 GO:0046887 GO:0046676 GO:0070374 GO:0048609 GO:0060325

XM_018042534.1 GO:0005829

XM_018060752.1 GO:0008152 GO:0016746 GO:0016021 GO:0005737

XM_018042866.1 GO:0006357 GO:0001104 GO:0016592

XM_018038704.1 GO:0005654 GO:0005737

XM_005682408.3 GO:0070062 GO:0051000 GO:0051712 GO:0051770 GO:0009897 GO:0016021 GO:0002925

XM_018041360.1 GO:0090575 GO:0042754 GO:0044822 GO:0042826 GO:0000785 GO:0016363 GO:0000380 GO:0042382 GO:0000122 GO:1902177 GO:0003682 GO:0000166 GO:0045876 GO:0000724 GO:0000980 GO:0070932 GO:0001047

XM_018044490.1 GO:0080008 GO:0051298 GO:0031146 GO:0016567 GO:0005737 GO:0007067 GO:0019901 GO:0019005

XM_018042981.1 GO:0050995 GO:0019915 GO:0045444 GO:0030176 GO:0034389

XM_005681305.3 GO:0003171 GO:0060412 GO:0031490 GO:0060577 GO:0060460 GO:0001077 GO:0005737 GO:0008584 GO:0070986 GO:0003253 GO:0033189 GO:0001570 GO:0048557 GO:0001102 GO:0035315 GO:0000978 GO:0007368 GO:0035116 GO:0021855 GO:0001105 GO:0031076 GO:2000288 GO:0060127 GO:0042475 GO:0055009 GO:0001764 GO:0045944 GO:0016055 GO:0001569 GO:0060578 GO:0055015 GO:0061325 GO:0000122 GO:0060126 GO:0030334 GO:0005667 GO:0048536 GO:0035886 GO:0061031 GO:0043388 GO:0051219 GO:0005634 GO:0003350 GO:0007520 GO:0001701 GO:0021763 GO:0035993 GO:0042803 GO:0001078 GO:0061072 GO:0008585 GO:0002074 GO:0009725 GO:0043021

XM_018048528.1 GO:0016477 GO:0005057 GO:0030676 GO:0044291 GO:0007160 GO:0016601 GO:0031234 GO:0035023

XM_005677834.2 GO:0070062 GO:0000165 GO:0038128 GO:0050900 GO:0002223 GO:0072600 GO:0000139 GO:0038095 GO:0007173 GO:0005525 GO:0005886 GO:0007411 GO:0007265 GO:0032403

XM_018051353.1 GO:0004407 GO:0030849 GO:0000805 GO:0048738 GO:0016580 GO:0005737 GO:0001741 GO:0000122 GO:0001106 GO:0003682 GO:0007519 GO:0016575 GO:0000806

XM_018039801.1 GO:0006790 GO:0001537 GO:0016051 GO:0016021

XM_005678498.3 GO:0016705 GO:0020037 GO:0005506 GO:0016021 GO:0004497 GO:0055114

XM_018044563.1 GO:0007186 GO:0097211 GO:2001223 GO:0004968 GO:0016021 GO:0005886

XM_018065451.1 GO:0006646 GO:0004306

XM_018041390.1 GO:0038061 GO:0000978 GO:0005654 GO:0033257 GO:0001077 GO:0005737 GO:0002268 GO:0002467 GO:0048536 GO:0045944 GO:0030198

XM_018039616.1 GO:0005634 GO:0007165 GO:0009409 GO:0060612 GO:0006366 GO:0045893 GO:0046872 GO:0050873 GO:0000987 GO:0033613 GO:0003700

XM_018049275.1 GO:0016020

XM_005680781.3 GO:0007165 GO:0005524 GO:0005267 GO:0051607 GO:0042626 GO:0008281 GO:0030017 GO:0044325 GO:0042383 GO:0071805 GO:0008076

XM_005701133.3 GO:0005634 GO:0005737

XM_005680499.3 GO:0005313 GO:0089711 GO:0030672 GO:0016021

XM_005683552.3 GO:0045859 GO:0030335 GO:0005737 GO:0000993 GO:0006368 GO:0006357 GO:0043967 GO:0005730 GO:0008607 GO:0000123 GO:0010485 GO:0002098 GO:0010484 GO:0051536 GO:0007417 GO:0046872 GO:0008023 GO:0001764 GO:0043966 GO:0033588

XM_005682388.3 GO:0007399 GO:0030154 GO:0017091 GO:0000166

XM_018061381.1 GO:0003677 GO:0005654

XM_013966270.2 GO:0005768 GO:0016050 GO:0019898 GO:0006897 GO:0035091

XM_018062722.1 GO:0003376 GO:0005765 GO:0016310 GO:0046512 GO:0017050 GO:0005829 GO:0001568 GO:0007420 GO:0038036 GO:0008284

XR_311260.2 GO:0003729 GO:0005762 GO:0005743 GO:0070124 GO:0003735 GO:0070125

XM_018061063.1 GO:0016020 GO:0042827 GO:0007596 GO:0042470 GO:0005764 GO:0042803 GO:0031085 GO:0030318 GO:0050821 GO:0007040

XM_018044749.1 GO:0000287 GO:0005765 GO:0005783 GO:0005524 GO:0045332 GO:0016021 GO:0004012 GO:0055037 GO:0005886

XM_005691649.3 GO:0044390 GO:0070738 GO:0070740 GO:0030433 GO:0070736 GO:0005741 GO:0018169 GO:0008766 GO:0070737 GO:0006914 GO:0051865 GO:0016021 GO:0008270 GO:0043773 GO:0043774

XM_005701148.3 GO:0034198 GO:0005765 GO:0032008 GO:0071230 GO:0003924 GO:0005794 GO:0034613 GO:0005525

XM_005682919.3 GO:0009055 GO:1902600 GO:0008121 GO:0016021 GO:0005743 GO:0070469 GO:0055114

XM_018050284.1 GO:0005452 GO:0016021 GO:0045177 GO:0015698 GO:0098656

XM_018045364.1 GO:0046872 GO:0006355 GO:0005622 GO:0003676

XM_013974907.2 GO:0000784 GO:0010792 GO:0006260 GO:0072429 GO:0030054 GO:0005737 GO:0006289 GO:0043085 GO:0005654 GO:0048257 GO:0070522 GO:0017108 GO:0000790 GO:0048476 GO:0008821 GO:0033557 GO:0000724 GO:0008047

XM_018055152.1 GO:0000185 GO:0005524 GO:0008349 GO:0034612 GO:0009411 GO:0005737

XM_013967213.2 GO:0003779 GO:0017048 GO:0001725 GO:0030036 GO:0042802 GO:0005886 GO:0005737

XM_018057437.1 GO:0046872 GO:0005634 GO:0007275 GO:0045944 GO:0000977 GO:0001077 GO:0008134

XM_018043802.1 GO:0070062 GO:0005654 GO:0008270 GO:0005737

XM_013973047.2 GO:0015937 GO:0004633

XM_018056372.1 GO:0010839 GO:0005925 GO:0001945 GO:0048013 GO:0031295 GO:0002042 GO:0009887 GO:0046875 GO:0016021 GO:0050920 GO:2000727 GO:0072178 GO:0005886

XM_005691174.3 GO:0097027 GO:0030674 GO:0031146 GO:0031625 GO:0005737 GO:0051443 GO:0019005 GO:1901800 GO:0034644 GO:2000060 GO:0005730 GO:0006974 GO:0050816 GO:0030332 GO:0005654 GO:0045741 GO:0042802 GO:1903378 GO:1903146 GO:1990452 GO:0007062 GO:0032876 GO:0070374 GO:0050821

XM_018056813.1 GO:0007585 GO:0016525 GO:0005576 GO:0016021

XM_018043156.1 GO:0007608 GO:0005654 GO:0005524 GO:0021564 GO:0003025 GO:0007626 GO:0007605 GO:0005614 GO:0008201 GO:0021554 GO:0021563

XM_013965682.2 GO:0042384 GO:0030032 GO:0003779 GO:0001725 GO:0030036 GO:0045944 GO:0008270 GO:0030027

XM_018053560.1 GO:0046872 GO:0005524 GO:0003774 GO:0016459 GO:0043547 GO:0035556 GO:0005096

XM_005676786.3 GO:0031571 GO:0000784 GO:0006284 GO:0006260 GO:0019903 GO:0031625 GO:0010569 GO:0006289 GO:0003697 GO:0016605 GO:0000785 GO:0006298 GO:2000001 GO:0035861 GO:0003684 GO:0005662 GO:0000723

XM_005679064.3 GO:0015908 GO:0008458 GO:0006091 GO:0015936 GO:0005102 GO:0005777 GO:0006635 GO:0009437 GO:0005739 GO:0051791

XM_018060689.1 GO:0012505 GO:0019898 GO:0005543

XM_005687650.3 GO:0008274 GO:0033566 GO:0005813 GO:0005819

XM_005681738.3 GO:0016310 GO:0003677 GO:0005634 GO:0005524 GO:0009165 GO:0006220 GO:0006334 GO:0046982 GO:0004137 GO:0042803 GO:0009157 GO:0008144 GO:0000786

XM_005698332.2 GO:0034184 GO:0005634 GO:0005615 GO:0000785 GO:0031625 GO:1990166 GO:0035861 GO:0031334 GO:2000781 GO:0032403

XM_005684899.2 GO:0006457 GO:0003755 GO:0005654 GO:0044822 GO:0000166 GO:0000413 GO:0005737

XM_018065633.1 GO:0019800 GO:0030054 GO:0005509 GO:0030198 GO:0005614 GO:0005539 GO:0045202 GO:0010811 GO:0005604

XM_005679997.3 GO:0016021

XM_018049472.1 GO:0050714 GO:0048015 GO:0005737 GO:0098592

XM_018045669.1 GO:0018095 GO:0070740 GO:0048487 GO:0043014

XM_018066843.1 GO:0005509 GO:0016021

XM_013964725.2 GO:0005634 GO:0005524 GO:0004674 GO:0006468 GO:0005813

XM_013962879.2 GO:0060999 GO:0007613 GO:0006357 GO:0000981 GO:0005634 GO:0043565 GO:0005737

XM_005697723.3 GO:0046872 GO:0003676

XM_005678500.3 GO:0005509 GO:0016021

XM_018043472.1 GO:0005119 GO:0036064 GO:0005113 GO:0035058 GO:0005813 GO:0034464 GO:0061512 GO:0005930

XM_018042366.1 GO:0007399 GO:0030154 GO:0005524 GO:0006816 GO:0042803 GO:0014069 GO:0001666 GO:0046777 GO:0006979 GO:1901897 GO:0004683 GO:0000082 GO:0048169 GO:0005516 GO:0033017 GO:0051259

XM_018041602.1 GO:0004114 GO:0007601 GO:0050728 GO:0004930 GO:0005887 GO:0050710 GO:0007186 GO:0046879 GO:0042277 GO:0008527 GO:0043066 GO:0032870 GO:0046872 GO:0007603 GO:0030139 GO:0045444 GO:0005504 GO:0010827 GO:1901652 GO:0070374 GO:0046549 GO:0050912

XM_018061457.1 GO:0043234 GO:0016021 GO:0005778

XM_005681449.3 GO:0060170 GO:0007259 GO:0072218 GO:0035725 GO:0048763 GO:0042127 GO:0071464 GO:0072235 GO:0005102 GO:0045180 GO:0003127 GO:0001892 GO:0001658 GO:0005887 GO:0072177 GO:0005911 GO:0061441 GO:0021915 GO:0060674 GO:0072686 GO:0070062 GO:0002133 GO:0072075 GO:0005267 GO:0031512 GO:0072208 GO:0030027 GO:0042805 GO:0030814 GO:0071556 GO:0071458 GO:0045944 GO:0045429 GO:0060315 GO:0031941 GO:0031659 GO:0042994 GO:0071498 GO:0005245 GO:0021510 GO:0051117 GO:2000134 GO:0060078 GO:0035502 GO:0036064 GO:0072219 GO:0035904 GO:0051219 GO:0072214 GO:0072284 GO:0042803 GO:0009925 GO:0001947 GO:0071470 GO:0071158 GO:0044325 GO:0071910 GO:0005509 GO:0050982 GO:0005248 GO:0071805 GO:0043398 GO:0031587 GO:0031513

XM_005699805.3 GO:0055085 GO:0016021

XM_005699438.2 GO:0035335 GO:0008138 GO:0004725

XM_005684506.3 GO:0006810 GO:0005654 GO:0042995 GO:0071944 GO:0060134 GO:0005737 GO:0043025 GO:0021846 GO:0008289 GO:0022008 GO:0005911 GO:0005215

XM_018058143.1 GO:0031012 GO:0070062 GO:0005615 GO:0044822

XM_018047555.1 GO:0000287 GO:0070062 GO:0005615 GO:0004634 GO:0001917 GO:0006096 GO:0043209 GO:0043204 GO:0000015

XM_018065489.1 GO:0038061 GO:0031146 GO:0045893 GO:0046983 GO:0019005 GO:0005829 GO:0006470 GO:0031648 GO:0000209 GO:0045892 GO:0005634 GO:0061630 GO:0050852 GO:0045862 GO:0005813 GO:0002223 GO:0038095 GO:0042347 GO:0042753 GO:0016055 GO:0000086 GO:0051403

XM_018060112.1 GO:0046835 GO:0006003 GO:0005524 GO:0006000 GO:0006096 GO:0003873 GO:0019901

XM_005675213.3 GO:0006486 GO:0016021 GO:0008457 GO:0007417 GO:0008378 GO:0000139

XM_018043910.1 GO:0007018 GO:0005524 GO:0008017 GO:0008574 GO:0005871

XR_001918531.1 GO:0002244 GO:0046872 GO:0003676

XM_018039405.1 GO:0045666 GO:0003714 GO:0046982 GO:0001093 GO:0043621 GO:0001077 GO:0000122 GO:0003682 GO:0005667 GO:0016021 GO:1900746 GO:0065004 GO:0005634 GO:0000978 GO:0070888 GO:0016525 GO:0001011 GO:0045944 GO:0001087 GO:0042118 GO:0006367

XM_018064273.1 GO:0016020 GO:0005783 GO:0005524 GO:0008022 GO:0004672 GO:0043234 GO:0043410 GO:0005078

XM_018052302.1 GO:0005634 GO:0005925 GO:0031589 GO:0017166 GO:0005737 GO:0000122 GO:0051496 GO:0043410 GO:0008134

XM_005690644.3 GO:0001649 GO:0005615 GO:0030335 GO:0016023 GO:0051901 GO:0005109 GO:0038031 GO:0022011 GO:0070062 GO:0051894 GO:0014068 GO:0043408 GO:0001953 GO:0005743 GO:0005929 GO:0051897 GO:0032027 GO:0005791 GO:0001968 GO:0038133 GO:0005741 GO:1900026 GO:0051497 GO:0031175 GO:0060348 GO:0030971 GO:0005758 GO:0045162 GO:0005578 GO:0046872 GO:0051496 GO:0005794 GO:0033268 GO:0014734 GO:0035024

NM_001285760.1 GO:0071837 GO:0000978 GO:0005654 GO:0042826 GO:0042803 GO:0000790 GO:0043923 GO:0000982 GO:0046872 GO:0008022 GO:0045944 GO:0008134

XM_013970370.2 GO:0007264 GO:0008321 GO:0005622 GO:0043547

XM_018061939.1 GO:1901233 GO:2000096 GO:1901231 GO:0005737 GO:0090090 GO:0045732 GO:0005509 GO:0030165 GO:0000159

XM_018040686.1 GO:0006886 GO:0070062 GO:0030425 GO:0017137 GO:0048471

XM_018051486.1 GO:0005654 GO:0005524 GO:0035556 GO:1901990 GO:0031573 GO:0005730 GO:0004674 GO:0031572 GO:0016572

XM_005697831.3 GO:0070062 GO:0009247 GO:0005794 GO:0016021 GO:0001733

NM_001285629.1 GO:0001649 GO:0004320 GO:0031177 GO:0004313 GO:0016296 GO:0047451 GO:0004319 GO:0042470 GO:0055114 GO:0004316 GO:0047117 GO:0004315 GO:0071353 GO:0006633 GO:0005739 GO:0005886 GO:0030879 GO:0070062 GO:0004317 GO:0044822 GO:0042587 GO:0004314 GO:0016295 GO:0005794

XM_005698381.3 GO:0005856 GO:0003713 GO:0005925 GO:0006355 GO:0005667 GO:0008270 GO:0005737

XM_018059304.1 GO:0097025 GO:0016323 GO:1903361 GO:0097016 GO:0005737 GO:0002011 GO:0007269 GO:0005923 GO:0030165 GO:0015031 GO:0045211 GO:0070062 GO:0008092 GO:0014069 GO:0043005 GO:0045199 GO:0006887

XM_005684180.3 GO:0046872 GO:0006355 GO:0005622 GO:0003676

XM_018061766.1 GO:0004726 GO:0005856 GO:0035335 GO:0008092 GO:0009898 GO:0005737

XM_005699656.3 GO:0070062 GO:0016021 GO:0005622 GO:0042771 GO:0071480 GO:0060548

XM_005687916.3 GO:0051015 GO:0070062 GO:0001725 GO:0005523 GO:0030018 GO:0071691 GO:0031005

XM_018061294.1 GO:0070062 GO:0005856 GO:0007015 GO:0007264 GO:0005525 GO:0005886 GO:0005737

XM_018050496.1 GO:0016020 GO:0070062 GO:0005634 GO:0044822 GO:0000381 GO:0036002 GO:0051148 GO:0000166 GO:0048025

XM_018040580.1 GO:0003677 GO:0005654 GO:0044822 GO:0006355 GO:0005737 GO:0031053 GO:0050769 GO:0008283 GO:0000166 GO:0097150

XM_005682259.3 GO:0005576 GO:0005179

XM_018051176.1 GO:0005938 GO:0005102 GO:0012506 GO:0005829 GO:0043197 GO:0070062 GO:0032467 GO:0006605 GO:0043542 GO:0008021 GO:0042803 GO:0043198 GO:0003779 GO:0031647 GO:0017022 GO:0030511 GO:0032435 GO:0048167 GO:0014047

XM_018057584.1 GO:0070062 GO:0005634 GO:0035335 GO:0004725 GO:0005886 GO:0005769

XM_018064773.1 GO:0005654 GO:0005815 GO:0030036 GO:0005903 GO:0005737 GO:0003779 GO:0007275 GO:0051014

XM_005689482.3 GO:0046470 GO:0007159 GO:0033344 GO:0034372 GO:0070328 GO:0005507 GO:0034361 GO:0034445 GO:0019430 GO:0070062 GO:0031210 GO:0030300 GO:0006695 GO:0006982 GO:0033700 GO:0044240 GO:0051006 GO:0043691 GO:0042744 GO:0002227 GO:0010898 GO:0015485 GO:0016209 GO:0042627 GO:0034364 GO:0031102 GO:0042632 GO:0072562 GO:0035634 GO:0017127 GO:0042803 GO:0034380 GO:0060228 GO:0009986 GO:0045723 GO:0010873

XM_018061195.1 GO:2000984 GO:0006754 GO:0006119 GO:0005758 GO:0007005

XM_018057049.1 GO:0005923

XM_018052427.1 GO:0007528 GO:0048403 GO:0051896 GO:0048935 GO:0051965 GO:0021987 GO:0005887 GO:0001570 GO:0046548 GO:0048709 GO:0010628 GO:0005768 GO:0001764 GO:0007631 GO:0033138 GO:0060041 GO:0005524 GO:0035584 GO:0043235 GO:0042490 GO:0005829 GO:0060291 GO:0038179 GO:0045211 GO:0018108 GO:0007623 GO:0060175 GO:0021954 GO:0031547 GO:0007612 GO:0010976 GO:0043195 GO:0071230 GO:2000811 GO:0043087 GO:0014047

XM_018043083.1 GO:0043153 GO:0044822 GO:0000166 GO:0008270 GO:0032922 GO:0010628 GO:0006417

XM_013968498.2 GO:0016020 GO:0005840 GO:0005524 GO:0005829

XM_005692426.3 GO:0020037 GO:0070330 GO:0005506 GO:0031090 GO:0005789 GO:0055114 GO:0019825

XM_018060831.1 GO:0000226 GO:0033043 GO:0031175 GO:0005813 GO:0005516 GO:0051011 GO:0030507

XM_018048296.1 GO:0070062

XM_018058816.1 GO:0044255 GO:0052689

XM_005690738.3 GO:2001183 GO:2001180 GO:0019955 GO:0005615 GO:0009897 GO:0008625 GO:0042127 GO:0032496 GO:0005031 GO:0006954 GO:0005887 GO:0007275 GO:0006955 GO:0033209 GO:0042981 GO:0070207

XM_018045182.1 GO:0046034 GO:0070062 GO:0005759 GO:0005524 GO:0006165 GO:0006172 GO:0004017 GO:0004550 GO:0009142 GO:0046899 GO:0046033 GO:0005525 GO:0046039

XM_005675094.3 GO:0005977 GO:0004864 GO:0043666 GO:0043086 GO:0000164 GO:0009966

XM_005678045.3 GO:0008152 GO:0016746

XM_018060621.1 GO:0006886 GO:0005634 GO:0032880 GO:0005794 GO:0031338 GO:0017137 GO:0005769 GO:0005096

XM_013965333.2 GO:0016702 GO:0019511 GO:0005783 GO:0005634 GO:0005506 GO:0055114 GO:0031418 GO:0004656

XM_018054380.1 GO:0016021

XM_005688485.3 GO:0000287 GO:0070062 GO:0046686 GO:0005524 GO:0043295 GO:0004363 GO:0006750 GO:0042803

XM_018066080.1 GO:0070062 GO:0031090 GO:0016021 GO:0005789 GO:0006465 GO:0008236

XM_018042394.1 GO:0016324 GO:0006970 GO:0034465 GO:0060083 GO:0043065 GO:0005901 GO:0030007 GO:0060072 GO:0042391 GO:0008076 GO:0070062 GO:0005249 GO:0045794 GO:0034765 GO:0001666 GO:0003779 GO:0046872 GO:0051592 GO:0071805

XM_005696051.3 GO:0005634 GO:0000178

XM_018067240.1 GO:0003779 GO:0030036 GO:0044822 GO:0007507 GO:0030054

XM_005687505.3 GO:0060348 GO:0003677 GO:0005654 GO:0030901 GO:0006468 GO:0030509 GO:0051216 GO:0005737 GO:0060395 GO:0007179 GO:0071407 GO:0005667 GO:0001657 GO:0003700 GO:0030902

NM_001314327.1 GO:0035690 GO:0001205 GO:0010499 GO:0016567 GO:0071356 GO:1902037 GO:0042149 GO:0045454 GO:0071499 GO:0046326 GO:0005829 GO:0005886 GO:0001102 GO:1902176 GO:0005634 GO:2000379 GO:0000785 GO:0005813 GO:0045995 GO:0036003 GO:2000352 GO:0006954 GO:1903206 GO:2000121 GO:0019904 GO:0043161 GO:0000980 GO:0032993 GO:0030194

XM_013968970.2 GO:0071260 GO:0009897 GO:0031625 GO:0043491 GO:0043547 GO:0030890 GO:0032735 GO:2000353 GO:0042511 GO:0006874 GO:0090037 GO:0043231 GO:0070062 GO:0042832 GO:0051092 GO:0043406 GO:0051607 GO:0043123 GO:0048304 GO:0002768 GO:0045944 GO:0051023 GO:0035631

XM_018053732.1 GO:0070062 GO:0007340 GO:0016567 GO:0008270 GO:0001669 GO:0051726 GO:0004842

XM_005677674.3 GO:0000244 GO:0044822 GO:0042802 GO:0016607 GO:0005737 GO:0046540 GO:0005681 GO:0043234 GO:0015030 GO:0019013

XM_005680835.2 GO:0006397 GO:0044822 GO:0000184 GO:0035145 GO:0008380 GO:0051028 GO:0071013

XM_018038716.1 GO:0003729 GO:0005654 GO:0035281 GO:0070883 GO:0008565 GO:0000049 GO:0042565 GO:0005737 GO:0008536 GO:1900370 GO:0006611

XM_005686936.2 GO:0097051 GO:0071786 GO:0000139 GO:0030859 GO:0055038 GO:0000145 GO:0005913 GO:0007264 GO:0031489 GO:0005886 GO:0016197 GO:0070062 GO:0071782 GO:0019882 GO:0098641 GO:0005925 GO:0071236 GO:0019003 GO:0045200 GO:0030670 GO:0005802 GO:0007409 GO:0006893 GO:0048471 GO:0003924 GO:0006886 GO:0032869 GO:0032593 GO:0043001 GO:0072372 GO:0005789 GO:0005525 GO:0061467

XM_005687742.3 GO:0097680 GO:0006260 GO:0003910 GO:0035019 GO:0005737 GO:0010332 GO:0002328 GO:0000012 GO:0000793 GO:0005654 GO:0005925 GO:0048146 GO:0043524 GO:0033152 GO:0051102 GO:0071897 GO:0008022 GO:0033153 GO:0003677 GO:0045190 GO:0005524 GO:0032807 GO:0033077 GO:0051103 GO:0005886 GO:0010165 GO:0051276 GO:0001701 GO:0007417 GO:0005958 GO:0050769 GO:0006297

XM_018055349.1 GO:0034198 GO:0016020 GO:0005524 GO:0010575 GO:0019903 GO:0051260 GO:0070417 GO:0004694 GO:0042149 GO:0001503 GO:0030968 GO:0001525 GO:0042802 GO:0031018 GO:0046777 GO:1902235 GO:0045943 GO:0006983

XM_018066739.1 GO:0016525 GO:0030169 GO:0007267 GO:0042742 GO:0005540 GO:0007155 GO:0005041 GO:0005887 GO:0005044 GO:0006898 GO:0005509

XM_018041577.1 GO:0036312 GO:0042802 GO:0014068 GO:0034123 GO:0034134 GO:0034122 GO:0050727 GO:0034162 GO:0005829 GO:0034154 GO:0005886 GO:0034142

XM_018059279.1 GO:0005874

XM_018064254.1 GO:0019725 GO:0097345 GO:0007264 GO:0047497 GO:0005509 GO:0031307 GO:0005525 GO:0003924

XM_018064391.1 GO:0015807 GO:1902475 GO:0015179 GO:0016021 GO:0005886

XM_018044489.1 GO:0080008 GO:0051298 GO:0031146 GO:0016567 GO:0005737 GO:0007067 GO:0019901 GO:0019005

XM_018061415.1 GO:0010923

XR_001917192.1 GO:0045665 GO:0000122 GO:0005634 GO:0046982 GO:0016568

XM_005675491.2 GO:0080008 GO:0045116 GO:0061630 GO:0005654 GO:0031146 GO:0031467 GO:0042787 GO:0005737 GO:0019788 GO:0031462 GO:0031466 GO:0008270 GO:0097602 GO:0031463 GO:0043224

XM_018059259.1 GO:0001228 GO:0070888 GO:0042789 GO:0001190 GO:0043425 GO:0045944 GO:0005667 GO:0000977 GO:0008270 GO:0048037 GO:0097067 GO:0001102

XM_005696387.3 GO:0007186 GO:0016021 GO:0007166 GO:0004930

XM_005680723.3 GO:0005925

XM_018060103.1 GO:0016021

XM_018053204.1 GO:0016571 GO:0050798 GO:0016605 GO:0006338 GO:0016363 GO:0001227 GO:0000122 GO:0060004 GO:0008544 GO:0043367 GO:0043374 GO:0000977 GO:0003682 GO:0005720

NM_001314299.1 GO:0001558 GO:0031994 GO:0070062 GO:0005615 GO:0032868 GO:0043567 GO:0031995 GO:0042104

XM_013965041.2 GO:0016705 GO:0020037 GO:0005506 GO:0016021 GO:0004497 GO:0055114

XM_018041698.1 GO:0007186 GO:0016021 GO:0007166 GO:0004930

XM_018053308.1 GO:0003677 GO:0005634 GO:0005524 GO:0006334 GO:0004842 GO:0008270 GO:0000786 GO:0000209

XM_018064736.1 GO:0004028 GO:0005783 GO:0050061 GO:0046577 GO:0005777 GO:0055114 GO:0033306 GO:0006081 GO:0016021 GO:0052814 GO:0070062 GO:0006714 GO:0007417 GO:0004030 GO:0008544 GO:0005743 GO:0007422

XM_005681533.3 GO:0019210 GO:0042326 GO:0005737 GO:0001772 GO:0007264 GO:0045576 GO:0005525 GO:0030217 GO:0043124

XM_018061156.1 GO:0048008 GO:0048011 GO:0005737 GO:0042593 GO:0007173 GO:0033277 GO:0060020 GO:0005158 GO:0035265 GO:0021697 GO:0000077 GO:0048013 GO:0032528 GO:0007409 GO:0051428 GO:0061582 GO:0048806 GO:0036302 GO:2001275 GO:0042445 GO:0035335 GO:0040014 GO:0051463 GO:0048873 GO:0004726 GO:0030220 GO:0007229 GO:0048839 GO:0009755 GO:0060125 GO:0005070 GO:0005634 GO:0000187 GO:0035855 GO:0033629 GO:0043254 GO:0006641 GO:0030971 GO:0045931 GO:0046825 GO:0046887 GO:0046676 GO:0070374 GO:0048609 GO:0060325

XM_005678573.3 GO:0019367 GO:0009922 GO:0005783 GO:0030148 GO:0016021 GO:0042761 GO:0034625

XM_005680320.3 GO:0055114 GO:0016491

XM_005709697.3 GO:0031965 GO:0005730

XM_005689136.3 GO:0006486 GO:0046982 GO:0050508 GO:0021772 GO:0006024 GO:0001503 GO:0000139 GO:0072498 GO:0007411 GO:0007492 GO:0007369 GO:0007498 GO:0015014 GO:0042803 GO:0050509 GO:0046872 GO:0030176

XM_018060921.1 GO:0046982 GO:0035259 GO:0005737 GO:0007088 GO:0045893 GO:0006713 GO:2000649 GO:0086010 GO:0017080 GO:0014704 GO:0005886 GO:0070062 GO:0021762 GO:0050774 GO:0043066 GO:0042921 GO:0006886 GO:0003779 GO:0044325 GO:0019904 GO:0007010 GO:0019899

XM_005675211.3 GO:0050853 GO:0002467

XM_005693163.3 GO:0016020 GO:0044822 GO:0005524 GO:0005730 GO:0004386

XM_018043895.1 GO:0005576

XM_005687457.3 GO:0070062

XM_018056867.1 GO:0008289

XM_005679478.3 GO:0018095 GO:0016337 GO:0036064 GO:0005814 GO:0042384 GO:0009986 GO:0005911 GO:0072372 GO:0015031 GO:0005886

XM_018042169.1 GO:0005544 GO:0005635 GO:0010629 GO:0008283 GO:0005178 GO:0005829 GO:0006914 GO:0009651 GO:0048306 GO:0007599 GO:0006874 GO:0042584 GO:0005886 GO:0070062 GO:0044822 GO:0035176 GO:0009992 GO:0008360 GO:0005509 GO:0005789 GO:0030855

XM_005683697.3 GO:0030317 GO:0009566 GO:0005576 GO:0043066 GO:0007286 GO:0008584 GO:0005179

XM_018050638.1 GO:0005654 GO:0043524 GO:0016234 GO:0043195 GO:0043025 GO:0030426 GO:0048487 GO:0042417 GO:0043014 GO:0005794 GO:0005509 GO:0007268 GO:0050808 GO:0005739

XM_018038866.1 GO:0001952 GO:0005615 GO:0038083 GO:0005524 GO:0014909 GO:0060444 GO:0007566 GO:0043235 GO:0005887 GO:0044319 GO:0008285 GO:0070062 GO:0001558 GO:0043583 GO:0010715 GO:0038062 GO:0038063 GO:0060749 GO:0005518 GO:0061302

XM_018062283.1 GO:0007275 GO:0051260

NM_001285553.1 GO:0032007 GO:0033596 GO:0043547 GO:0051056 GO:0005096

XM_013970377.2 GO:0005634 GO:0045111 GO:0051721 GO:0005737

XM_018054688.1 GO:0032725 GO:2000667 GO:0042802 GO:0005576 GO:2000778 GO:0006915 GO:0019841

XM_018046429.1 GO:0009952 GO:0043433 GO:0046982 GO:0001077 GO:0005737 GO:0001658 GO:0035162 GO:0048536 GO:0005667 GO:0005634 GO:0000978 GO:0048538 GO:0030326 GO:0030278 GO:0045665 GO:0045944 GO:0030325 GO:0009954 GO:0010971 GO:0048706 GO:0008134 GO:0008284

XM_005678198.3 GO:0070062 GO:0005737

XM_018047823.1 GO:0003779 GO:0016477 GO:0017048 GO:0030036 GO:0008360 GO:0005737 GO:0032794

XR_001919837.1 GO:0046872 GO:0005829 GO:0043103 GO:0004000 GO:0046103 GO:0006154 GO:0009117

XM_018049881.1 GO:0070062 GO:0030209 GO:0003940 GO:0005975

XM_005675036.2 GO:0006810 GO:0016021

XM_005685789.3 GO:0042423 GO:0001694 GO:0006548 GO:0004398 GO:0030170

XM_013976668.2 GO:0006506 GO:0016021 GO:0005789 GO:0000506

XM_013975155.2 GO:0046872 GO:0005634 GO:0006355 GO:0003700

XM_005683053.3 GO:0005759 GO:0003677 GO:0005634 GO:0005524 GO:0002161 GO:0044822 GO:0006260 GO:0006450 GO:0042803 GO:0019478 GO:0046872 GO:0004821 GO:0032543 GO:0006427 GO:0051500

XM_005680354.3 GO:0006397 GO:0004535 GO:0005634 GO:0031251 GO:0000932 GO:0003676 GO:0000289 GO:0010606 GO:0090503

XM_018061811.1 GO:0005634 GO:0006260

XM_018050676.1 GO:0090083 GO:0042417 GO:0042802 GO:0031625 GO:0046928 GO:0008219 GO:0005737

XM_018066147.1 GO:0005634 GO:0006355 GO:0046982 GO:0051056 GO:0005737 GO:0005096

XM_018041749.1 GO:0005654 GO:0005737

XM_018054679.1 GO:0016020 GO:0046872 GO:0005783 GO:0006096 GO:0005576 GO:0043843

XM_018042992.1 GO:0003755 GO:0000413 GO:0005528 GO:0005789 GO:0061077

XM_018053006.1 GO:0060831 GO:0060271 GO:0007368 GO:0003406 GO:0007507 GO:0061512 GO:0002088 GO:0042733

XM_018065386.1 GO:0005874 GO:0043547 GO:0007021 GO:0005912 GO:0070830 GO:0034333 GO:0048487 GO:0005829 GO:0010812 GO:0005923 GO:0016328 GO:0007023 GO:0005096 GO:0031115

XM_018064043.1 GO:0051015 GO:0005874 GO:0007050 GO:0008093 GO:0008017 GO:0005737 GO:0001725 GO:0007026 GO:0001578

XM_005681421.3 GO:0003677 GO:0005654 GO:0005524 GO:0070933 GO:0004386 GO:0000729 GO:0035861 GO:0051304 GO:0043044 GO:0000018 GO:0000792 GO:0070932 GO:0043596 GO:0016568

XM_018044174.1 GO:0045944 GO:0031307 GO:0034613

XM_005690949.3 GO:0030425 GO:0060159 GO:0007213 GO:0005634 GO:0009968 GO:0043547 GO:0005737 GO:0043204 GO:0031234 GO:0001965 GO:0032809 GO:0005096

XM_018062674.1 GO:0016020 GO:0044822 GO:0005524 GO:2000623 GO:0004386

XM_018039398.1 GO:0070062 GO:0034765 GO:0051260 GO:0005251 GO:0050796 GO:0008016 GO:0071805 GO:0008076

XM_005697651.2 GO:0071541 GO:0016282 GO:0006446 GO:0003743 GO:0033290 GO:0031369 GO:0001731

XM_018066216.1 GO:0005783 GO:0007264 GO:0005525 GO:0042169

XM_013972172.2 GO:0002943 GO:0017150 GO:0050660 GO:0055114

XM_005692406.3 GO:0038061 GO:0016020 GO:0000090 GO:0005524 GO:0008233 GO:0006521 GO:0036402 GO:0016234 GO:1901800 GO:0090090 GO:0051437 GO:0001824 GO:0031595 GO:0043488 GO:0033209 GO:0000209 GO:0008540 GO:0000165 GO:0005654 GO:0050852 GO:0045899 GO:0031597 GO:0030433 GO:0002223 GO:0090263 GO:0038095 GO:0060071 GO:0051436 GO:0031145 GO:0002479 GO:0017025

XM_018043891.1 GO:0000981 GO:0042127 GO:0060217 GO:0021527 GO:2000036 GO:0003682 GO:0000118 GO:0001525 GO:0070888 GO:0042826 GO:0043249 GO:0000790 GO:0001085 GO:0060375 GO:0031334 GO:0045648 GO:0045944 GO:0000980 GO:0033193 GO:0046982 GO:0051781 GO:0060018 GO:0000122 GO:0000979 GO:0045799 GO:0005667 GO:0030220 GO:0060216 GO:0030221 GO:0035855 GO:0060218 GO:0045931 GO:0007626

XM_005686634.3 GO:0038061 GO:0005634 GO:0001205 GO:0034097 GO:0006954 GO:0033256 GO:0043123 GO:0000122 GO:0045944 GO:0005829 GO:0003682 GO:0000980 GO:0045087 GO:0032688

XM_005686937.3 GO:0072384 GO:0005524 GO:0005874 GO:0003777 GO:0008017 GO:0005737 GO:0016887 GO:0005871

NM_001285563.1 GO:0000902 GO:0001701 GO:0005385 GO:0043029 GO:0048701 GO:0060173 GO:0016021 GO:0005886 GO:0071577

XM_018066991.1 GO:0050811 GO:0008333 GO:0005739 GO:0005769

XM_018046273.1 GO:0050731 GO:0000132 GO:0032755 GO:0005874 GO:0048365 GO:0043547 GO:0032587 GO:0071225 GO:0005737 GO:0031982 GO:0043025 GO:0032760 GO:0007015 GO:0007026 GO:0000902 GO:0050768 GO:0005925 GO:0051092 GO:0030676 GO:0008017 GO:0043198 GO:0046872 GO:0045944 GO:0043234 GO:0035023

XM_005675962.3 GO:0005634 GO:0021766 GO:0043524 GO:0048715 GO:0042475 GO:0000122 GO:0021893 GO:0000977 GO:0003682 GO:0021882 GO:0009954 GO:0045746 GO:0048706 GO:0021544

XM_005680143.3 GO:0045892 GO:0031965 GO:0031398 GO:0032436 GO:0090090 GO:0005829 GO:0006606 GO:0008270 GO:0001843 GO:2000691 GO:0035904 GO:0060976

XM_018038265.1 GO:0030362 GO:0005654 GO:0030674 GO:0030289 GO:0050790 GO:0010569

XM_005684253.3 GO:0016021

XM_018057942.1 GO:0040029 GO:0048854 GO:0050671 GO:0051569 GO:0021895 GO:0001701 GO:0035097 GO:0046872 GO:0000979 GO:0048812 GO:0080182 GO:0002052

XM_013964082.2 GO:0010468 GO:0005615 GO:0005654 GO:0005179 GO:0051428 GO:0032331 GO:0005794 GO:0007189 GO:0030819 GO:0002076

XM_018050891.1 GO:0010216 GO:0010390 GO:0061630 GO:0031493 GO:0042802 GO:0035064 GO:0016363 GO:0044729 GO:0000122 GO:0051865 GO:0008270 GO:0005720 GO:0005657

XM_018059393.1 GO:0051491 GO:0007165 GO:0051270 GO:0043547 GO:0008360 GO:0005547 GO:0051497 GO:0005794 GO:0005096

XM_013971062.2 GO:0005622

XR_001917495.1 GO:0000287 GO:0050321 GO:0005783 GO:0005524 GO:0051117 GO:0007067 GO:0019901 GO:0061178 GO:0031532 GO:0005634 GO:0005813 GO:0043462 GO:0048471 GO:0007409 GO:0030010 GO:0018105 GO:0004674 GO:0070059 GO:0051301 GO:0060590 GO:0000086 GO:0006887

XM_018050025.1 GO:0030154 GO:0007275 GO:0016021 GO:0007283

XM_018040558.1 GO:0008305 GO:0070062 GO:0007155 GO:0009986 GO:0005615 GO:0007229 GO:0001948 GO:0010668

XM_018054596.1 GO:0008630 GO:0070062 GO:0097192 GO:0060011 GO:2001243 GO:0046982 GO:0051400 GO:0016607 GO:0005741 GO:0007283 GO:0042803 GO:0005829 GO:0016021

XM_018046851.1 GO:0070062 GO:0030246 GO:0016160 GO:0005975 GO:0016021 GO:0004558

XM_018050465.1 GO:0045892 GO:0030282 GO:0071222 GO:0072675 GO:0061430 GO:0050727 GO:0071354 GO:0045944 GO:0002281 GO:0071348

XM_018061796.1 GO:0016567 GO:0031463 GO:0004842

XM_018043086.1 GO:0005615 GO:0016324 GO:0030054 GO:0005200 GO:0035264 GO:0016192 GO:0005543 GO:0043025 GO:0007416 GO:0003779 GO:0008091 GO:0030534 GO:0021692

XM_018042230.1 GO:0070062 GO:0010719 GO:0016853 GO:0010633 GO:0005737 GO:0060392 GO:0009058 GO:0030512 GO:0050680 GO:0060394 GO:0030277

XM_018041961.1 GO:0009790 GO:0001893

XM_018066961.1 GO:0005524 GO:0060324 GO:0045104 GO:0048011 GO:0035019 GO:0030878 GO:0005829 GO:1902042 GO:0005739 GO:0031143 GO:0005886 GO:0008285 GO:0000186 GO:0035994 GO:0005634 GO:0030154 GO:0005057 GO:0042802 GO:0048538 GO:0017016 GO:0035773 GO:0071550 GO:0046872 GO:0004674 GO:0045944 GO:0005794 GO:0033138 GO:0031333

XM_013975419.2 GO:0043022 GO:0016021

XM_013967565.2 GO:0032797 GO:0034719 GO:0097504 GO:0005829 GO:0000245 GO:0000387

XM_018048802.1 GO:0044822 GO:0000166 GO:0060213 GO:0031047

XM_018048094.1 GO:0042128 GO:0020037 GO:0030151 GO:0005739 GO:0055114 GO:0016491

XM_005682512.3 GO:0016021 GO:0055114 GO:0016491

XM_018054143.1 GO:0045666 GO:2000379 GO:0042743 GO:2000609 GO:0050727 GO:0016021 GO:0005789 GO:0005886 GO:0015031

XM_018046143.1 GO:0016941 GO:0005524 GO:0006468 GO:0008074 GO:0035556 GO:0042562 GO:0043235 GO:0006182 GO:0004672 GO:0004016 GO:0016021 GO:0007168 GO:0005886 GO:0004383

XM_018038313.1 GO:0005615 GO:0010951 GO:0004867 GO:0042802 GO:0035987 GO:0005604

XM_018052244.1 GO:0090090

XM_018065023.1 GO:0006397 GO:0008298 GO:0044822 GO:0042802 GO:0000184 GO:0031625 GO:0016607 GO:0006417 GO:0051028 GO:0048471 GO:0030529 GO:0035145 GO:0008380

XM_005700426.3 GO:0005622 GO:0035023 GO:0043547 GO:0005089

XM_018066495.1 GO:0003723 GO:0005524 GO:0004829 GO:0006435 GO:0005737

XM_018061296.1 GO:0048188 GO:0000166 GO:0042800 GO:0051568 GO:0005737 GO:0003676

XM_005677363.3 GO:0016021 GO:0005789

XM_005679190.3 GO:0003677 GO:0006281

XM_018051050.1 GO:0030552 GO:0016020 GO:0007608 GO:0010738 GO:0035690 GO:0043949 GO:0048471 GO:0006198 GO:0046872 GO:0005829 GO:0004115

XM_005695543.3 GO:0045773 GO:0070062 GO:0005654 GO:0043001 GO:0005794 GO:0000042 GO:0051020

NM_001285568.1 GO:0006508 GO:0030163 GO:0004190

XM_005684802.3 GO:0045892 GO:0003677 GO:0044822 GO:0016607 GO:2000144 GO:0043620 GO:0005730 GO:2001244 GO:2001022

XM_018061480.1 GO:0005794

XM_018046256.1 GO:0005654 GO:0044822 GO:0005761 GO:0015935 GO:0006915

XM_005685386.3 GO:0005654 GO:0008168 GO:0005507 GO:0006412 GO:0005739 GO:0032259

XM_005701006.3 GO:0043154 GO:0000287 GO:0005634 GO:0005524 GO:0043027 GO:0006468 GO:0035556 GO:0032496 GO:0002224 GO:0005737 GO:0019901 GO:0004674 GO:0045944

XM_005675697.3 GO:0070936 GO:0006511 GO:0005730 GO:0008270 GO:0005737 GO:0004842

XM_018040461.1 GO:0005654 GO:0044822 GO:0000166 GO:0005794 GO:0035068 GO:0035278 GO:0000932

XM_018063939.1 GO:0005887 GO:0015129 GO:0035879

XM_018042332.1 GO:0043547 GO:0051056 GO:0005096

XM_018043602.1 GO:0016021 GO:0009607

XM_018041551.1 GO:0005634 GO:0035335 GO:0004725 GO:0007185 GO:0042803 GO:0005737 GO:0046627 GO:0033003 GO:0016021 GO:0005886

XM_005676337.3 GO:0051260

XR_001918996.1 GO:0050829 GO:0042802 GO:0030674 GO:0031410 GO:0001920 GO:0005802 GO:0048471 GO:0070530 GO:0043001 GO:0008022 GO:0000042 GO:0090161 GO:0043124 GO:0017137

XM_005698544.3 GO:0031161 GO:0014898 GO:0005622 GO:0048015 GO:0042578

XM_005700578.2 GO:0070062 GO:0046872 GO:0005634 GO:0000977 GO:0003700 GO:0045893

XM_018052363.1 GO:0045347 GO:0044822 GO:0005737 GO:0001078 GO:0000122 GO:0005730 GO:0000977 GO:0008270 GO:0005886

XM_018044177.1 GO:0070062 GO:0030182 GO:0004467 GO:0030307 GO:0001676 GO:0047676 GO:0060136 GO:0032307 GO:0044233 GO:0005811 GO:0016021 GO:0005739 GO:0008610 GO:0031957

XM_005693480.2 GO:0016021

XM_018061124.1 GO:0005615 GO:0014069 GO:0008139 GO:0000139 GO:0005509 GO:0004857 GO:0048306 GO:0005886 GO:0042308

XM_018038452.1 GO:0048786

XM_018051784.1 GO:0010839 GO:0042393 GO:0070062 GO:0046697 GO:0016540 GO:0005615 GO:0051603 GO:0031410 GO:0005764 GO:2000249 GO:0031069 GO:0005730 GO:0016807 GO:0004197

XM_018046146.1 GO:0000978 GO:0006306 GO:0000790 GO:0000122 GO:0016581 GO:0008270 GO:0043044 GO:0000980 GO:0003700 GO:0031492

XM_018046930.1 GO:0070062 GO:0000978 GO:0005634 GO:0001205 GO:0001046 GO:0001077 GO:0042803 GO:0030218 GO:0001190 GO:0034599 GO:0045944 GO:0016021 GO:0005789 GO:0000980

XM_005681556.3 GO:0008270

XM_018050777.1 GO:0005638 GO:0005637 GO:0005198

XM_018039937.1 GO:0038061 GO:0005654 GO:0005815 GO:0030289 GO:0006468 GO:0010569 GO:0004722 GO:0005521 GO:0005737 GO:0046872 GO:0071347 GO:0004704 GO:0006470 GO:0005886 GO:0032403

XM_005680751.3 GO:0071560 GO:2000741 GO:0055059 GO:0032332

XM_018064619.1 GO:0010369 GO:0005524 GO:0000780 GO:0051973 GO:0035174 GO:0032133 GO:0051256 GO:0030496 GO:0000122 GO:0034644 GO:0032212 GO:0002903 GO:0034501 GO:0032091 GO:0043988 GO:0032467 GO:0036089 GO:0005654 GO:0031577 GO:0045171 GO:0000776

XM_005699478.3 GO:0007030 GO:0005801 GO:0005802 GO:0003924 GO:0000139 GO:0007264 GO:0031985 GO:0005525

XM_018040494.1 GO:0000122 GO:0046872 GO:0045944 GO:0043433 GO:0016607 GO:0001077 GO:0044212 GO:0045879

XM_018051578.1 GO:0005759 GO:0016226 GO:0044822

XM_018042397.1 GO:0016324 GO:0006970 GO:0034465 GO:0060083 GO:0043065 GO:0005901 GO:0030007 GO:0060072 GO:0042391 GO:0008076 GO:0070062 GO:0005249 GO:0045794 GO:0034765 GO:0001666 GO:0003779 GO:0046872 GO:0051592 GO:0071805

NM_001285765.1 GO:0030324 GO:0030335 GO:0004222 GO:0035987 GO:0030307 GO:0042470 GO:0010831 GO:0016021 GO:0008270 GO:0005886 GO:0048754 GO:0001958 GO:0031012 GO:0005925 GO:0044354 GO:0045579 GO:0035988 GO:0048701 GO:0005509 GO:0045746 GO:0097094 GO:0031638 GO:0030574

XM_005674708.3 GO:0016323 GO:0005783 GO:0016327 GO:0005923 GO:0016021 GO:0005198

NM_001314245.1 GO:0005615 GO:0051603 GO:0042470 GO:0043394 GO:0005730 GO:0005739 GO:0070062 GO:0046697 GO:0005764 GO:0050790 GO:0048471 GO:0005518 GO:0004197 GO:0097067 GO:0046718 GO:0030855 GO:0030574

XM_018066138.1 GO:0043252 GO:0005887 GO:0015732 GO:0015347

XM_005679162.3 GO:0005634 GO:0044822 GO:0005925 GO:0043234 GO:0008270 GO:0005886 GO:0005737 GO:0008285

XM_018047847.1 GO:0033148 GO:0008168 GO:0043627 GO:0044212 GO:0035097 GO:0045944 GO:0008270 GO:0032259 GO:0008284

XM_018067167.1 GO:0016787 GO:0016021

XM_018046935.1 GO:0016477 GO:0007010 GO:0008360 GO:0005737

XM_005676012.3 GO:0007059 GO:0007052 GO:0000777 GO:0031262

XM_018061370.1 GO:0005814

XM_005680196.3 GO:0005654 GO:0016567 GO:0043066 GO:0005737 GO:0016874 GO:0000122 GO:0005730 GO:0008270 GO:0071157

XM_018058359.1 GO:0017075 GO:0005654 GO:0005874 GO:0019894 GO:0005739 GO:0031982

XM_018060694.1 GO:2001252 GO:0072687 GO:0007283

XM_005680678.3 GO:0007389 GO:0046872 GO:0045747 GO:0033829 GO:0030173 GO:0002315 GO:0032092

XM_018039721.1 GO:0046501 GO:0051537 GO:0046872 GO:0005743 GO:0004325 GO:0006783

XM_018060995.1 GO:2000785 GO:0005654 GO:0035335 GO:0019898 GO:0004725 GO:0042149 GO:0004722 GO:0052629 GO:0004438 GO:0005737 GO:0046872 GO:0046856

XM_018067203.1 GO:0051015 GO:0070062 GO:0071800 GO:0005938 GO:0030027 GO:0016601 GO:0001726 GO:0007416 GO:0097178 GO:0048812 GO:0019904 GO:0006898 GO:0002102

XM_018042357.1 GO:0005765 GO:0061630 GO:0045347 GO:0002250 GO:0016874 GO:0042289 GO:0031901 GO:0016021 GO:0008270 GO:0002495 GO:0000209 GO:0030659

XM_005682389.3 GO:0007399 GO:0030154 GO:0017091 GO:0000166

XM_018061578.1 GO:0001710 GO:0001077 GO:0005737 GO:0019901 GO:0070410 GO:0008285 GO:0000978 GO:0042592 GO:0030509 GO:1901522 GO:0070411 GO:0005637 GO:0030902 GO:0030901 GO:0046982 GO:0007183 GO:0007179 GO:0060038 GO:0000979 GO:0009880 GO:0071407 GO:0005667 GO:0007276 GO:0060348 GO:0061036 GO:0000165 GO:0042803 GO:0045669 GO:0006954 GO:0060395 GO:0046872 GO:0002051 GO:0001657

XM_018043970.1 GO:0043966 GO:0043982 GO:0043981 GO:0008270 GO:0043983 GO:0000123

XM_018043924.1 GO:0005509 GO:0016021 GO:0005576

XM_018064094.1 GO:0045776 GO:0070062 GO:0051603 GO:0045909 GO:0004185

XM_018048663.1 GO:0005524 GO:0006468 GO:2000021 GO:0035556 GO:0003084 GO:0032414 GO:0004674 GO:0005829 GO:0019869 GO:0019902 GO:0090188 GO:0010923

XM_005678046.3 GO:0008168 GO:0032259 GO:0008033

XM_018039067.1 GO:0006432 GO:0004826 GO:0005524 GO:0000049 GO:0005739 GO:0008033

XM_018054570.1 GO:0006397 GO:0016020 GO:0003723 GO:0005634 GO:0000166

XM_005680816.3 GO:0031464 GO:0019903 GO:0031116 GO:0071407 GO:0005829 GO:0048839 GO:0048102 GO:0030308 GO:0045892 GO:0006813 GO:0071850 GO:0005634 GO:0045930 GO:0008656 GO:0016301 GO:0071236 GO:0043066 GO:0045736 GO:0004861 GO:0045732 GO:0060770 GO:0007605 GO:0000082 GO:0007219 GO:0006919 GO:0071285 GO:0051271 GO:0008284

XM_018057444.1 GO:0006099 GO:0000287 GO:0005634 GO:0004449 GO:0051287 GO:0005739

XM_018060507.1 GO:0043123 GO:0061630 GO:0000151 GO:0016567 GO:0008270 GO:0004871 GO:0005769

XM_018050573.1 GO:0042802 GO:0008483 GO:0005739 GO:0030170

XM_018064345.1 GO:0046330 GO:0070062 GO:0006281 GO:0005524 GO:0030295 GO:0005737 GO:0019901 GO:0097194 GO:0051493 GO:0004702 GO:0031572

XM_018060597.1 GO:0001533 GO:0030057

XM_018062860.1 GO:0002098 GO:0032447 GO:0034227 GO:0005829 GO:0016779 GO:0000049

XM_018041834.1 GO:0003677 GO:0044822 GO:0006355 GO:0005673 GO:0006367 GO:0005737

XM_013966997.2 GO:0003677 GO:0071339 GO:0006355 GO:0003700 GO:0005737 GO:0046983

XM_005696319.3 GO:0007283

XM_005678577.2 GO:0038163 GO:0009986 GO:0005794 GO:0016021 GO:0038164 GO:0005886

XM_018047246.1 GO:0042119 GO:0048384 GO:0042800 GO:0030854 GO:0051568 GO:0006306 GO:0030218 GO:0002446 GO:0045893 GO:0071300 GO:0045171 GO:0003713 GO:0070688 GO:0008270 GO:0019899

XM_018058024.1 GO:0005737

XM_018058999.1 GO:0048741

XM_005693503.2 GO:0035095 GO:0005892 GO:0006936 GO:0098655 GO:0048747 GO:0030054 GO:0007274 GO:0015464 GO:0042166 GO:0004889 GO:0001941 GO:0007271 GO:0050877 GO:0042391 GO:0045211

XM_018060576.1 GO:0042393 GO:0005654 GO:0044822 GO:0003714 GO:2001243 GO:0070491 GO:0032066 GO:0005737 GO:0035067 GO:0034644 GO:0000122 GO:0031491 GO:0005730 GO:0002903 GO:0031497

XM_018062585.1 GO:0042493 GO:0031083 GO:0001654 GO:0030168 GO:0060155 GO:0033299 GO:0071806 GO:0008320 GO:0032816 GO:0048490 GO:0032402 GO:0030133 GO:0031175 GO:0035646 GO:0032438

XM_018057302.1 GO:0005634 GO:0043547 GO:1900026 GO:0043065 GO:0071479 GO:0017049 GO:0035023 GO:0051451 GO:0005886 GO:0005089 GO:0070301

XM_018052121.1 GO:0000287 GO:0005524 GO:0001669 GO:0007030 GO:0045332 GO:0005794 GO:0016021 GO:0004012 GO:0005886

XM_013976149.2 GO:0001540 GO:0005737

XM_018043390.1 GO:0006401 GO:0005634 GO:0004523 GO:0090502 GO:0032299

XM_018066175.1 GO:0000287 GO:0016310 GO:0052725 GO:0005524 GO:0016324 GO:0016853 GO:0005622 GO:0032957 GO:0047325 GO:0016787 GO:0021915 GO:0052726

XM_005700754.3 GO:0006914

XM_018043864.1 GO:0007399 GO:0046710 GO:0016323 GO:0046037 GO:0005615 GO:0019903 GO:0031625 GO:0032281 GO:0005737 GO:0043025 GO:0030426 GO:0010923 GO:0019900 GO:0035255 GO:0005923 GO:0030165 GO:0045211 GO:0043113 GO:0097120 GO:0014069 GO:0004385 GO:0043198 GO:0008022 GO:0001736 GO:0007268 GO:0045197

XM_005683134.3 GO:0005634 GO:0010976 GO:0005737

XM_018045247.1 GO:0030122 GO:0017124 GO:0048260 GO:0097320 GO:0008017 GO:0005543

XM_018058049.1 GO:0010468 GO:0003677 GO:0046872

XM_005675619.3 GO:0006281 GO:0090305 GO:0004518

XM_005692636.2 GO:0046314 GO:0016310 GO:0005615 GO:0005524 GO:0004111 GO:0009408 GO:0005737

XM_018066102.1 GO:0016799 GO:0006517 GO:0006491 GO:0004559 GO:0000139 GO:0030246 GO:0006013 GO:0016021 GO:0008270

XM_018050873.1 GO:0070062 GO:0005634 GO:0005815 GO:0005737

XM_018061971.1 GO:2000312 GO:0035255 GO:0016021 GO:0014069

XM_018058492.1 GO:0005758

XM_013975275.2 GO:0006511 GO:0045860 GO:0000082 GO:0031625 GO:0008284 GO:0019901

XM_018050112.1 GO:0016020 GO:0070062 GO:0005634 GO:0016337 GO:0097202 GO:0030054 GO:0006915 GO:0048471 GO:0043005 GO:0005515 GO:0005829 GO:0038096 GO:0048010 GO:0045202

XM_018049686.1 GO:0016605 GO:0005545 GO:0005635 GO:0035973 GO:0016234 GO:0016239 GO:0046872 GO:0034274 GO:0097635 GO:0003831

XM_005686520.3 GO:0046872 GO:0005524 GO:0044822 GO:0004386

XM_018038297.1 GO:0046872 GO:0016787 GO:0043234 GO:0016021 GO:0003676

XM_018057378.1 GO:0005634 GO:0046592 GO:0055114 GO:0046208 GO:0005737

XM_018044164.1 GO:0005622 GO:0035023 GO:0043547 GO:0005089

XM_018066021.1 GO:0000287 GO:0070062 GO:0006097 GO:0051287 GO:0005777 GO:0006099 GO:0004450 GO:0006102 GO:0005829 GO:0005743 GO:0006103

XM_018053007.1 GO:0070062 GO:0005783 GO:0004571 GO:0005509 GO:0016021 GO:0005793 GO:0006491 GO:0000139

XM_005679525.3 GO:0070062 GO:0007264 GO:0005622 GO:0005525

XM_005698847.1 GO:0008542 GO:0004222 GO:0009986 GO:0007155 GO:0006508 GO:0016021 GO:0008270 GO:0030534

XM_018050898.1 GO:0021549 GO:0070062 GO:0090557 GO:0021766 GO:0035335 GO:0021510 GO:0004725 GO:0021987 GO:0016021 GO:0022038

XM_005690832.3 GO:0000448 GO:0016020 GO:0016310 GO:0003723 GO:0005524 GO:0005730 GO:0051731

XM_005676299.3 GO:0009952 GO:0045815 GO:0005686 GO:0005515 GO:0003682 GO:0034693 GO:0001825 GO:0071011 GO:0003729 GO:0000785 GO:0016607 GO:0016363 GO:0071013 GO:0071004 GO:0005689 GO:0000245

XM_018066983.1 GO:0006355

XM_005701024.3 GO:0005783 GO:0022417 GO:0007283 GO:0055114 GO:0008584 GO:0045454 GO:0005829 GO:0005739 GO:0070062 GO:0005634 GO:0072593 GO:0042803 GO:0051920 GO:2000255 GO:0019471 GO:0004601 GO:0030198

XM_018047793.1 GO:0008152 GO:0003824

XM_005686394.3 GO:0016197 GO:0000781 GO:0042800 GO:0051568 GO:0044666 GO:0006348 GO:0042803 GO:0005802 GO:0048188 GO:0016568

XM_018061089.1 GO:0004843 GO:0044313 GO:0005741 GO:0000422 GO:0008053 GO:0006511 GO:0004197 GO:0016021 GO:0035871

XM_018056899.1 GO:0071862 GO:0043086 GO:0043085 GO:0004864 GO:0030036 GO:0001755 GO:2001045 GO:0005622 GO:0030027 GO:0003779 GO:0048484 GO:0061386 GO:0007266 GO:0051726 GO:0001843 GO:0008157

XM_013970902.2 GO:0005125 GO:0005576 GO:0006954

XM_018051070.1 GO:0045892 GO:0016020 GO:0003677 GO:0005654 GO:0044822 GO:0006468 GO:0003725 GO:0045893 GO:0005730 GO:0017148 GO:0045071 GO:0030529 GO:0005739

XM_018062526.1 GO:0005525

XM_005687862.3 GO:0007147 GO:0005654 GO:0005524 GO:0006468 GO:0005813 GO:0034048 GO:0007067 GO:0032154 GO:0004674 GO:0000086 GO:0051726

XM_018059672.1 GO:0045824 GO:0039536 GO:0005524 GO:0032715 GO:0050728 GO:0005739 GO:0043124 GO:0032688

XM_018038969.1 GO:0006810 GO:0043065 GO:0016021 GO:0005739

XM_018055864.1 GO:0008344 GO:0019901 GO:0046983 GO:0051932 GO:1901998 GO:0070062 GO:0001917 GO:0044822 GO:0042802 GO:0072583 GO:0043209 GO:0051262 GO:0003924 GO:0030117 GO:0007605 GO:0007032 GO:0005525

XM_018057532.1 GO:0016477 GO:0030154 GO:0038083 GO:0004715 GO:0005524 GO:0042127 GO:0005102 GO:0005737 GO:0007169 GO:0031234 GO:0045087

XM_018056662.1 GO:0006506 GO:0035269 GO:0007368 GO:0019348 GO:0004582 GO:0006487 GO:0005789 GO:0004169

XM_005684936.3 GO:0019752 GO:0005975 GO:0004459 GO:0055114 GO:0005737

XM_018061548.1 GO:0045892 GO:0006357 GO:0005654 GO:0043565 GO:0003700 GO:0005737 GO:0045893

XM_005691431.2 GO:0010008 GO:0030904 GO:0005770 GO:0008565 GO:0042147 GO:0006886 GO:0046872 GO:1990126 GO:0005829 GO:0005769

XM_018051815.1 GO:0070062 GO:0030246 GO:0004653 GO:0006493 GO:0016021 GO:0000139

XM_018049719.1 GO:0036064 GO:0045880 GO:0016021 GO:0003416

XM_018052065.1 GO:0005882

XM_013964639.2 GO:0031490 GO:0050729 GO:0007283 GO:0050796 GO:0046983 GO:0001190 GO:0005667 GO:0032922 GO:0045892 GO:0005654 GO:0000978 GO:0001046 GO:0070888 GO:0051092 GO:0000077 GO:0042634 GO:0004402 GO:0016573 GO:0005694 GO:0051775 GO:0000982 GO:2000074 GO:0043161 GO:0033391 GO:0071479 GO:0045944 GO:2000323

XM_018056761.1 GO:0046872 GO:0044822 GO:0016607

XM_005696421.3 GO:0016021

XM_018066104.1 GO:0016799 GO:0006517 GO:0006491 GO:0004559 GO:0000139 GO:0030246 GO:0006013 GO:0016021 GO:0008270

XM_018058061.1 GO:0016021

XR_310467.3 GO:0006508 GO:0004298 GO:0045893

XM_005675144.3 GO:0006612 GO:0009986 GO:0031849 GO:0016021 GO:0005622 GO:0001580 GO:0051205

XM_018065097.1 GO:0016477 GO:0098911 GO:0043588 GO:0042127 GO:0019903 GO:0090002 GO:0002159 GO:0051091 GO:0030018 GO:0005913 GO:0005829 GO:0045294 GO:0004871 GO:0014704 GO:0030057 GO:0070062 GO:0086073 GO:0007165 GO:0005925 GO:0086091 GO:0071665 GO:0005882 GO:0045296 GO:0016342 GO:0071681 GO:0034333 GO:0003713 GO:0050982 GO:0032993 GO:0005198 GO:0042307

XM_005675359.3 GO:0005615 GO:0010951 GO:0004867

XM_018048816.1 GO:0046872 GO:0044822

XM_018046416.1 GO:0050777 GO:0005737

XM_018057598.1 GO:0003677 GO:0046982 GO:0016032 GO:0001541 GO:0005669 GO:0005737 GO:0045893 GO:1901796 GO:0006368 GO:0033276 GO:0006357 GO:0071339 GO:0003713 GO:0006367 GO:0003700

XM_018060969.1 GO:0005634 GO:0008270 GO:0005737

XR_001295631.2 GO:0005462 GO:0006111 GO:0005464 GO:0015783 GO:0015790 GO:0030173 GO:0030176 GO:0005457 GO:1990569

XM_018062584.1 GO:0042493 GO:0031083 GO:0001654 GO:0030168 GO:0060155 GO:0033299 GO:0071806 GO:0008320 GO:0032816 GO:0048490 GO:0032402 GO:0030133 GO:0031175 GO:0035646 GO:0032438

XM_018042256.1 GO:0060441 GO:0045186 GO:0030901 GO:0008092 GO:0071896 GO:0045176 GO:0030859 GO:0072205 GO:0005913 GO:0042981 GO:0006461 GO:0008013 GO:0045197

XM_018040468.1 GO:0060041 GO:0008589 GO:1902017 GO:0021532 GO:0061512 GO:0035721 GO:0036064 GO:0072001 GO:0048705 GO:0035845 GO:0007368 GO:0032391 GO:0007507 GO:0005813 GO:0001750 GO:0035108 GO:0005930 GO:0030991

XM_005692185.3 GO:0044822 GO:0005730 GO:0005737 GO:0042255

XM_005688486.3 GO:0000287 GO:0070062 GO:0046686 GO:0005524 GO:0043295 GO:0004363 GO:0006750 GO:0042803

XM_018065831.1 GO:0046982 GO:0001077 GO:0003705 GO:0005737 GO:0019901 GO:0000122 GO:0071277 GO:0003682 GO:0005667 GO:0033613 GO:0061337 GO:0005654 GO:0000978 GO:0042826 GO:0001105 GO:0070375 GO:0048311 GO:0055005 GO:0000790 GO:0001085 GO:0006915 GO:0048813 GO:0045944 GO:0000002 GO:0046332 GO:0035035

XR_001295542.2 GO:0003677 GO:0005524 GO:0006355 GO:0005674 GO:0004386 GO:0006367

XM_018066660.1 GO:0005730 GO:0008440 GO:0005737 GO:0046854

XM_018038983.1 GO:0032007 GO:0006342 GO:0061630 GO:0005654 GO:0071596 GO:0007141 GO:0000785 GO:0033522 GO:0007283 GO:0005737 GO:0071233 GO:0000151 GO:0008270 GO:0005886 GO:0070728

XM_005691362.3 GO:0016020 GO:0070062 GO:1903445 GO:0003382 GO:0042802 GO:0005813 GO:0005737 GO:0072372

XM_013962734.2 GO:0070062 GO:0008970 GO:0002080 GO:0006629

XM_018065852.1 GO:0016021

XM_018041795.1 GO:0010763 GO:0051897 GO:0045785 GO:0031625 GO:0071356 GO:0051087 GO:0097178 GO:0005829 GO:0030838 GO:0005886 GO:0005634 GO:1903215 GO:0044822 GO:0072659 GO:0071364 GO:0051496 GO:2001145 GO:0033138

XM_018056122.1 GO:0048208 GO:0000139

XM_018049466.1 GO:0031490 GO:0050729 GO:0007283 GO:0050796 GO:0046983 GO:0001190 GO:0005667 GO:0032922 GO:0045892 GO:0005654 GO:0000978 GO:0001046 GO:0070888 GO:0051092 GO:0000077 GO:0042634 GO:0004402 GO:0016573 GO:0005694 GO:0051775 GO:0000982 GO:2000074 GO:0043161 GO:0033391 GO:0071479 GO:0045944 GO:2000323

XM_018039430.1 GO:0018345 GO:0072659 GO:0008270 GO:0016021 GO:0019706

XM_018051442.1 GO:0003697

XM_005683223.3 GO:0016021

XM_018064261.1 GO:0005547 GO:0007507 GO:0043547 GO:0005886 GO:0005737 GO:0043533 GO:0005096

XM_018039108.1 GO:0005765 GO:0055085 GO:0005524 GO:0042626 GO:0016021

XM_005701131.3 GO:0005634 GO:0005737

XM_018042369.1 GO:0007399 GO:0030154 GO:0005524 GO:0006816 GO:0042803 GO:0014069 GO:0001666 GO:0046777 GO:0006979 GO:1901897 GO:0004683 GO:0000082 GO:0048169 GO:0005516 GO:0033017 GO:0051259

XM_018063743.1 GO:0004252 GO:0050709 GO:0042058 GO:0016485 GO:0016021 GO:0005789 GO:0005886

XM_018049994.1 GO:0060271 GO:0035869 GO:0015031 GO:0005737

XM_018049137.1 GO:0055085 GO:0005634 GO:0005524 GO:0042626 GO:0016021 GO:0042803 GO:0005886 GO:0046415

XM_005676108.3 GO:0005025 GO:0030335 GO:0003143 GO:0048179 GO:2000017 GO:0016361 GO:0004702 GO:0001755 GO:0007368 GO:0001702 GO:0060037 GO:0030509 GO:0018107 GO:0007281 GO:0032926 GO:0045944 GO:0000082 GO:0001569 GO:0045177 GO:0023014 GO:0060923 GO:0005524 GO:0048185 GO:0007179 GO:0001707 GO:2001237 GO:0010862 GO:0050431 GO:0061445 GO:0001701 GO:0042803 GO:0045669 GO:0002526 GO:0051145 GO:0030501 GO:0046872 GO:0003289 GO:0003183 GO:0046332

XM_018046044.1 GO:0046886 GO:0010575 GO:0046982 GO:0033235 GO:0003705 GO:0017162 GO:0005737 GO:0001892 GO:0043619 GO:0004874 GO:0035326 GO:0043565 GO:0090575 GO:0030154 GO:0001666 GO:0030522 GO:0003713 GO:0045944

XM_018041409.1 GO:0070062 GO:0010494 GO:0005634 GO:0044822 GO:0017091 GO:0007281 GO:0000166 GO:0017145 GO:0008284

XM_018040785.1 GO:0050863 GO:0005070 GO:0019722 GO:0001772 GO:0006954 GO:0019901 GO:0010467 GO:0009967 GO:0005911 GO:0008180 GO:0045860 GO:0007229 GO:0016021 GO:0006955 GO:0002260 GO:0007265

NM_001287562.1 GO:0048538 GO:0046982 GO:0033162 GO:0055114 GO:0042803 GO:0048471 GO:0042438 GO:0043473 GO:0008283 GO:0005507 GO:0016021 GO:0004503

NM_001290120.1 GO:0008073 GO:0005634 GO:0090316 GO:0006521 GO:1902268 GO:0006596 GO:0005515 GO:0005829 GO:0045732 GO:0043086

XM_005701100.3 GO:0030501 GO:0051893 GO:0085029 GO:0016021 GO:0032956

XM_018052525.1 GO:0030177 GO:0005730 GO:0005819 GO:0009303 GO:0035064 GO:0007276

XM_005676497.3 GO:0046872 GO:0005634 GO:0045944 GO:0046982 GO:0003700 GO:0042803 GO:0044212

XM_013964599.2 GO:0002244 GO:0005634 GO:0016554 GO:0044822 GO:0000166

XM_005683005.3 GO:0005634 GO:0000166 GO:0008270 GO:0003676

XM_018041490.1 GO:0016020 GO:0003725 GO:0000932 GO:0005515 GO:0007223 GO:0005829 GO:0035280 GO:0003727 GO:0070578 GO:0006402 GO:0035279 GO:0035198 GO:0035068 GO:0010501 GO:0016442 GO:0048015 GO:0035278 GO:0031054

XM_018040103.1 GO:0016323 GO:0072218 GO:0007259 GO:0005737 GO:0032092 GO:0019901 GO:0001892 GO:0072205 GO:0072177 GO:0072287 GO:0001502 GO:0021915 GO:0060236 GO:0060674 GO:0048754 GO:0070062 GO:0002133 GO:0072237 GO:0016337 GO:0060428 GO:0031512 GO:0007507 GO:0030010 GO:0018105 GO:0045944 GO:0048806 GO:0036303 GO:0034405 GO:0070588 GO:0031659 GO:0042994 GO:0043588 GO:0021510 GO:0007050 GO:0016021 GO:0006611 GO:0030155 GO:0005634 GO:0001889 GO:0048565 GO:0061136 GO:0044325 GO:0009986 GO:0019904 GO:0050982 GO:0005262

XM_018065696.1 GO:0003677 GO:0005634

XM_005683135.3 GO:0005634 GO:0010976 GO:0005737

XM_018065631.1 GO:0019800 GO:0030054 GO:0005509 GO:0030198 GO:0005614 GO:0005539 GO:0045202 GO:0010811 GO:0005604

XM_018066163.1 GO:0016021

XM_018050456.1 GO:0005730 GO:0005829

NM_001285740.1 GO:0008299 GO:0004421 GO:0005743

XM_005690940.2 GO:0000062

XM_018038256.1 GO:0006338 GO:0006281 GO:0005524 GO:0006355 GO:0005813 GO:0031011 GO:0007067 GO:0051301 GO:0006310

XM_018041113.1 GO:0070588 GO:0050916 GO:0015867 GO:0042802 GO:0005245 GO:0034765 GO:0005227 GO:0051260 GO:0050917 GO:0005887 GO:0050913

XM_018055895.1 GO:0043231 GO:0070062 GO:0015630

XM_018047102.1 GO:0031965 GO:0006508 GO:0016805

XM_018041866.1 GO:0070062 GO:0035725 GO:0015319 GO:0005315 GO:0005887 GO:0035435 GO:0016032

XM_018046016.1 GO:0006359 GO:0005666 GO:0000790

XM_018064744.1 GO:0005634 GO:0045666 GO:0045687 GO:0005737 GO:0003924 GO:0071158 GO:0016021 GO:0008270 GO:0005525

XM_013966501.2 GO:0005634 GO:0007368 GO:0032525 GO:0060349 GO:0001503 GO:0036342 GO:0000122 GO:0007219

XM_013972667.2 GO:0003148 GO:0060412 GO:0010667 GO:0045666 GO:0007512 GO:0001077 GO:0051891 GO:0003705 GO:0005737 GO:0003211 GO:0090090 GO:0030878 GO:0003682 GO:0001570 GO:0000978 GO:0003166 GO:0010832 GO:0003221 GO:0060928 GO:0060037 GO:0030509 GO:0055117 GO:0010765 GO:0010735 GO:0003285 GO:0003168 GO:0003222 GO:0046982 GO:0010736 GO:0000122 GO:0001190 GO:0060971 GO:0048536 GO:0030097 GO:0060043 GO:0090575 GO:0060261 GO:0003350 GO:0060929 GO:0001104 GO:0045214 GO:0045823 GO:0003278 GO:0055005 GO:0042803 GO:0001947 GO:0060413 GO:0003342 GO:0060347 GO:0008134 GO:0008284

XM_005681121.3 GO:0016020

NM_001286091.1 GO:0030904 GO:0048365 GO:0034045 GO:0070062 GO:0006622 GO:0007174 GO:0019003 GO:0031902 GO:0030670 GO:0003924 GO:0005811 GO:0005525 GO:0000421 GO:1903543 GO:0045022 GO:0090385 GO:0032419 GO:0090383 GO:0022615 GO:0005829 GO:0007264 GO:0008333 GO:0045453 GO:2000785 GO:0019076 GO:0048524 GO:0042147 GO:0033162 GO:0043195 GO:0097208 GO:0045732 GO:0005794 GO:0019886

XM_018066872.1 GO:0031088 GO:0016529 GO:0042045 GO:0048016 GO:0009791 GO:0005730 GO:0050882 GO:0016021 GO:0005955 GO:0050849 GO:0019855 GO:0014069 GO:0035091 GO:0001666 GO:0005220 GO:0070059 GO:0032469 GO:0031094 GO:0005637 GO:0005789 GO:0051209

XM_018061277.1 GO:0030901 GO:0030307 GO:0007283 GO:0000122 GO:0000979 GO:0008270 GO:0021555 GO:0048596 GO:0035518 GO:0021670 GO:0043524 GO:2000178 GO:0070544 GO:1902459 GO:0021993 GO:0031519 GO:0021592 GO:0021678

XM_018049221.1 GO:0007249 GO:0005622

XM_005700155.3 GO:0003723 GO:0005847 GO:0000166 GO:0006378

XM_005686364.3 GO:0005634 GO:0005829 GO:0019901

XM_018038585.1 GO:0003707 GO:0001228 GO:0005634 GO:0004879 GO:0030522 GO:0000977 GO:0045944 GO:0008270 GO:0043401

XR_001919095.1 GO:0001937 GO:0016020 GO:0005125 GO:0050900 GO:0007267 GO:0000049 GO:0042803 GO:0005737 GO:0006954 GO:0009986 GO:0051020 GO:0017101

XM_018055854.1 GO:0008344 GO:0019901 GO:0046983 GO:0051932 GO:1901998 GO:0070062 GO:0001917 GO:0044822 GO:0042802 GO:0072583 GO:0043209 GO:0051262 GO:0003924 GO:0030117 GO:0007605 GO:0007032 GO:0005525

XM_018052903.1 GO:0007093 GO:0016020 GO:0004712 GO:0051304 GO:0005524 GO:0006468

XM_018051813.1 GO:0005634 GO:0016021 GO:0005886 GO:0005737

XM_018050804.1 GO:0043254 GO:0016021 GO:0009966 GO:0005789 GO:0031648

XM_018058857.1 GO:0046872 GO:0003676

XM_005686314.3 GO:0051308 GO:0007292 GO:0007283 GO:0005737

XM_018038985.1 GO:0032007 GO:0006342 GO:0061630 GO:0005654 GO:0007141 GO:0000785 GO:0033522 GO:0007283 GO:0006511 GO:0071233 GO:0000151 GO:0008270 GO:0005886 GO:0070728

XM_018052425.1 GO:0007528 GO:0048403 GO:0051896 GO:0048935 GO:0051965 GO:0021987 GO:0005887 GO:0001570 GO:0046548 GO:0048709 GO:0010628 GO:0005768 GO:0001764 GO:0007631 GO:0033138 GO:0060041 GO:0005524 GO:0035584 GO:0043235 GO:0042490 GO:0005829 GO:0060291 GO:0038179 GO:0045211 GO:0018108 GO:0007623 GO:0060175 GO:0021954 GO:0031547 GO:0007612 GO:0010976 GO:0043195 GO:0071230 GO:2000811 GO:0043087 GO:0014047

XM_013963915.2 GO:0051661 GO:0003779 GO:0030334 GO:0007010 GO:0051684 GO:0008360 GO:0031616

XM_018066218.1 GO:0005783 GO:0007264 GO:0005525 GO:0042169

XM_005681111.3 GO:0003899 GO:0003677 GO:0005813 GO:0005666 GO:0006383

XM_018048382.1 GO:0005887 GO:0030133 GO:0016010

XM_018065251.1 GO:0005922 GO:0034220 GO:0048738 GO:0007601 GO:0005216 GO:0016021 GO:0007268 GO:0001570 GO:0048468

XM_005685632.3 GO:0016279 GO:0018023

XM_018060997.1 GO:2000785 GO:0005654 GO:0035335 GO:0019898 GO:0004725 GO:0042149 GO:0004722 GO:0052629 GO:0004438 GO:0005737 GO:0046872 GO:0046856

XM_005676642.3 GO:0071934 GO:0016021 GO:0015403

XM_005676172.3 GO:0003688 GO:0005654 GO:0000784 GO:0005524 GO:0005664 GO:0006270 GO:0005737 GO:0005730 GO:0015629

XM_013963384.2 GO:0045892 GO:0090004 GO:0001921 GO:0031623 GO:0086103 GO:0008565 GO:0006816 GO:0005764 GO:0015026 GO:0043235 GO:0006886 GO:0008277 GO:0009986 GO:0016021 GO:0005886 GO:0071392

XM_013969295.2 GO:0043484 GO:0003729 GO:0005654 GO:0000166

XM_018051961.1 GO:0007155 GO:0016327 GO:0008022 GO:0016324 GO:0031410 GO:0005923 GO:0043220

XM_018049129.1 GO:0006810 GO:0005576 GO:0005215

XM_005690991.3 GO:0007186 GO:0015271 GO:0030322 GO:0071805 GO:0022841 GO:0008076

XM_018056652.1 GO:0005829 GO:0008104 GO:0005886 GO:0005802 GO:0019901

XM_013962541.2 GO:0035690 GO:0097421 GO:2000301 GO:0030054 GO:0050796 GO:0046326 GO:0005769 GO:0000186 GO:0070062 GO:0043209 GO:0017016 GO:0030033 GO:0043005 GO:0048471 GO:0003924 GO:0071333 GO:0038180 GO:0005525 GO:0061028 GO:0032486 GO:0005770 GO:0008565 GO:0043547 GO:0071320 GO:0050766 GO:0005829 GO:1901888 GO:0005886 GO:0032966 GO:2001214 GO:0017034 GO:0010976 GO:0097327 GO:0070374 GO:0032403 GO:0032045

XM_005684247.2 GO:0046872 GO:0006355 GO:0005622 GO:0002230 GO:0003676

XR_001919091.1 GO:0010468 GO:0003677 GO:0046872

XM_005687181.3 GO:0015035 GO:0009055 GO:0020037 GO:0055114 GO:0045454 GO:0000139 GO:0016829 GO:0043295 GO:0016021 GO:0001516 GO:0050220

XM_005694938.3 GO:0044822 GO:0006412 GO:0005763 GO:0003735

XM_018056043.1 GO:0005524 GO:0006165 GO:0021591 GO:0004017 GO:0004550 GO:0009142 GO:0036126 GO:0005930 GO:0004127

XM_018050287.1 GO:0005452 GO:0016021 GO:0045177 GO:0015698 GO:0098656

XM_005683543.2 GO:0045892 GO:0003691 GO:0000784 GO:0051973 GO:0035563 GO:0003720 GO:0005737 GO:0045893 GO:0032212 GO:0005697 GO:0032403

XM_005679346.3 GO:0047369 GO:0005739

XM_018057659.1 GO:0005887 GO:0007605 GO:0032218 GO:0032217 GO:0034605

XM_018051459.1 GO:0003677 GO:0005524 GO:0006260 GO:0005737 GO:0006449 GO:0071044 GO:0003682 GO:0044530 GO:0008270 GO:0005654 GO:0006281 GO:0044822 GO:0000785 GO:0000184 GO:0009048 GO:0004004 GO:0035145

XM_005692186.3 GO:0003723 GO:0005634 GO:0042255

XM_018063684.1 GO:0008732 GO:0005829 GO:0006545 GO:0006567

XM_018039549.1 GO:0031683 GO:0070062 GO:0007608 GO:0007191 GO:0004871 GO:0005525 GO:0003924

XR_001920008.1 GO:0042802 GO:0035023 GO:0007032 GO:0043547 GO:0005089

XM_018062237.1 GO:0000122 GO:1902035 GO:0003723 GO:0005654 GO:0005730 GO:0030198 GO:0048286

XM_005678748.3 GO:0070588 GO:0051289 GO:0009897 GO:0009409 GO:0050955 GO:0042803 GO:0016048 GO:0016021 GO:0005262 GO:0006874 GO:0070207

XM_018043338.1 GO:0005765 GO:0046872 GO:0016021 GO:0031902 GO:0055114 GO:0016491

XM_005686929.1 GO:0005634

NM_001285712.1 GO:0006979 GO:0047066 GO:0070062 GO:0005634 GO:0007275 GO:0004602 GO:0005739 GO:0055114

XM_018064765.1 GO:0003677 GO:0016605 GO:0003917 GO:0008270 GO:0005694 GO:0006265

XM_018048119.1 GO:0016021

XM_018045912.1 GO:0021762 GO:0000978 GO:0005654 GO:0001078 GO:0000122 GO:0046872 GO:0005794 GO:0045944 GO:0007276

XM_018054976.1 GO:0046835 GO:0001678 GO:0005524 GO:0005623 GO:0006096 GO:0005536 GO:0004396

XM_005680471.3 GO:0070062 GO:0005654 GO:0044822 GO:0019370 GO:0004301 GO:0004463 GO:0043171 GO:0005737 GO:0006508 GO:0004177 GO:0008237 GO:0008270

XM_018053132.1 GO:0004843 GO:0006511 GO:0016579 GO:0008270

XM_018062054.1 GO:0045218 GO:0090136 GO:0070062 GO:0007018 GO:0005524 GO:0005874 GO:0008569 GO:0005813 GO:0007030 GO:0005915 GO:0008017 GO:0005794 GO:0005871

XM_018055113.1 GO:0001558 GO:0005520 GO:0070062 GO:0010951 GO:0004867 GO:0016021 GO:0030165

XM_018039384.1 GO:0005654 GO:0005737

XM_018062862.1 GO:0008083 GO:0005615 GO:0008284

XM_018043842.1 GO:0005488

XM_013976575.2 GO:0016020 GO:0048675 GO:0036459 GO:0030509 GO:0005737 GO:0007179 GO:0030426 GO:0006511 GO:0070410 GO:0001764 GO:0016579

XM_005678200.3 GO:0051015 GO:0030334 GO:0005925 GO:0030018 GO:0048739 GO:0051493 GO:0008307

XM_018040266.1 GO:0030515 GO:0044822 GO:0016787

XM_005689234.3 GO:0036396 GO:0044822 GO:0016607 GO:0080009

XM_005677897.3 GO:0015986 GO:0070062 GO:0021762 GO:0005654 GO:0000276 GO:0043209 GO:0046933

XM_018063823.1 GO:0008286 GO:0031623 GO:0070436 GO:0019903 GO:0046854 GO:0019901 GO:0046875 GO:0008180 GO:0005911 GO:0008543 GO:0007411 GO:0042059 GO:0070062 GO:0005654 GO:0044822 GO:0014066 GO:0042802 GO:0043408 GO:0016032 GO:0046934 GO:0005768 GO:0042770 GO:0001784 GO:0071479 GO:0048646 GO:0036092 GO:0043547 GO:0031295 GO:0012506 GO:0005730 GO:0005829 GO:0017124 GO:0030838 GO:0051291 GO:0005070 GO:2000379 GO:0050900 GO:0038128 GO:0007267 GO:0005154 GO:0007568 GO:0038095 GO:0060670 GO:0005088 GO:0005168 GO:0016303 GO:0009967 GO:0043560 GO:0005794 GO:0038096 GO:0007265

XM_018055810.1 GO:0016020 GO:0061630 GO:0045806 GO:0046755 GO:0030163 GO:0005737 GO:0070086 GO:0008270 GO:0051865 GO:2000786 GO:0000209

XM_018040484.1 GO:0016020 GO:0005654 GO:0016525

XM_018056300.1 GO:0055085 GO:0005524 GO:0042626 GO:0016021

XM_018061960.1 GO:0005634 GO:0006511 GO:0007275 GO:0016567 GO:0008270 GO:0004842 GO:0016874

XM_005684608.3 GO:0008270 GO:0005739 GO:0055114 GO:0016491

XM_005682625.3 GO:0016702 GO:0019511 GO:0005783 GO:0005634 GO:0005506 GO:0055114 GO:0031418 GO:0004656

XM_018052010.1 GO:0042393 GO:0061630 GO:0005654 GO:0030154 GO:0071158 GO:0008283 GO:0006511 GO:0051865 GO:0008270 GO:0005720

XM_018057463.1 GO:0006479 GO:0004719

XM_018066308.1 GO:0000978 GO:0005634 GO:0046982 GO:0001701 GO:0043066 GO:0001077 GO:0007420 GO:0017162 GO:0005737 GO:0001666 GO:0032355 GO:0045944 GO:0005667 GO:0008284

XM_005679127.2 GO:0051897 GO:0005524 GO:0036092 GO:0014065 GO:0071320 GO:0005737 GO:0046854 GO:0002376 GO:0046875 GO:0006897 GO:0006935 GO:0005886 GO:0005944 GO:0007186 GO:2000270 GO:0001525 GO:0043406 GO:0006954 GO:0046934 GO:1903169 GO:0004674 GO:0016303 GO:0035005

NM_001314348.1 GO:0070062 GO:0009897 GO:0006954 GO:0008305 GO:0007160 GO:0005178 GO:0007229 GO:0001618 GO:0046718

XM_005688516.3 GO:0000244

XM_018045736.1 GO:0046872 GO:0003676

XM_005701641.2 GO:0005212 GO:0016021 GO:0002088

XR_001917015.1 GO:0046872 GO:0016787 GO:0043234 GO:0016021 GO:0003676

XR_001297163.2 GO:0007093 GO:0005634 GO:0007096 GO:0005737

XM_018056676.1 GO:0070588 GO:0016323 GO:0051480 GO:0030863 GO:0005901 GO:0015279 GO:0005887 GO:0005911 GO:0006828 GO:0034704 GO:0048709 GO:0014051 GO:0045296 GO:0070679 GO:0009986 GO:0070509 GO:0008013

XM_018053016.1 GO:0003677 GO:0005634 GO:0005524 GO:0006260 GO:0000724 GO:0004386 GO:0097362 GO:0007292

NM_001314204.1 GO:0014902 GO:0045666 GO:0055012 GO:0001568 GO:0005737 GO:0003211 GO:0071560 GO:2000310 GO:2000987 GO:0000978 GO:0051966 GO:0042826 GO:0048703 GO:0016607 GO:0003151 GO:0002062 GO:0002634 GO:0001764 GO:0045944 GO:2000727 GO:0045663 GO:0014898 GO:0071498 GO:0060998 GO:0071374 GO:0051963 GO:0000122 GO:0072102 GO:0090073 GO:0030220 GO:0003139 GO:0046928 GO:0030318 GO:0060079 GO:0071864 GO:0050853 GO:0003138 GO:0001958 GO:2001013 GO:0000165 GO:0060045 GO:0003680 GO:0045669 GO:0045652 GO:0043234 GO:0050680 GO:0009615 GO:0035690 GO:0072160 GO:0071222 GO:0001077 GO:0060297 GO:0030890 GO:0007521 GO:0006959 GO:0035984 GO:0003682 GO:0033613 GO:0071837 GO:0001046 GO:0043524 GO:0048667 GO:2000311 GO:0000983 GO:0001974 GO:0035198 GO:0060025 GO:0001782 GO:0000980 GO:0048167 GO:0001205 GO:0046982 GO:0071277 GO:0060536 GO:0060021 GO:0003185 GO:0007611 GO:2000111 GO:0014033 GO:0051145 GO:0001947 GO:0030501 GO:0030279 GO:0010694 GO:2001016 GO:0030224

XM_005700702.3 GO:0016323 GO:0007269 GO:0008022 GO:1903361 GO:0005911 GO:0005829 GO:0045199 GO:0006887

XM_013974012.2 GO:0000710 GO:0005524 GO:0003684 GO:0032300 GO:0030983 GO:0051026 GO:0000795

XM_018057856.1 GO:0046982 GO:0043547 GO:0051056 GO:0005096

XM_018041789.1 GO:0071222 GO:0050713 GO:0071356 GO:0010595 GO:0000932 GO:0055118 GO:0004521 GO:2000627 GO:0090503 GO:0003682 GO:0035925 GO:0045766 GO:0032088 GO:0051259 GO:1902714 GO:0005856 GO:0005654 GO:0045600 GO:0061014 GO:0045019 GO:1900165 GO:1900016 GO:0042406 GO:0045944 GO:0035198 GO:0003677 GO:0035613 GO:0005791 GO:0010884 GO:0010656 GO:0005886 GO:0010942 GO:2000379 GO:0004532 GO:0010508 GO:0061158 GO:0046872 GO:0042347 GO:0090502

XM_018056120.1 GO:0048208 GO:0000139

XM_005677501.2 GO:0005730 GO:0005509 GO:0008270 GO:0005737

XM_018039935.1 GO:0004332 GO:0006096

XM_018047564.1 GO:0005249 GO:0034765 GO:0071805 GO:0008076

XM_018052674.1 GO:0016021

XM_005676826.3 GO:0016042 GO:0003847

XM_018053122.1 GO:0070936 GO:0005634 GO:0005783 GO:0032580 GO:0006355 GO:0048365 GO:0007030 GO:0042787 GO:0004842 GO:0016874 GO:0061025 GO:0030334 GO:0017137 GO:0007049

XR_001917871.1 GO:0005654 GO:0030904 GO:0007165 GO:0032266 GO:0071203 GO:0001772 GO:0006886 GO:0031901 GO:1990126 GO:0005829 GO:0008333

XM_005681276.3 GO:0016422 GO:0003723 GO:0005654 GO:0036396 GO:0019827 GO:0000398 GO:0061157 GO:0080009

XM_018051126.1 GO:0003723 GO:0005783 GO:0016324 GO:0006654 GO:0004630 GO:0006807 GO:0010977 GO:0032534 GO:0030334 GO:0072001 GO:0005765 GO:0048017 GO:0001889 GO:0001701 GO:0006491 GO:0035091 GO:0005768 GO:0030139 GO:0005509 GO:0005794 GO:0050764

XM_013974056.2 GO:0003677 GO:0046872 GO:0005634 GO:0006355

XM_018059921.1 GO:0007186 GO:0019395 GO:0031226 GO:0097003 GO:0042802 GO:0046982 GO:0042593 GO:0019901 GO:0033211 GO:0055100 GO:0010906 GO:0016021 GO:0019216

XM_013972268.2 GO:0005654 GO:0007165 GO:0015914 GO:0008526 GO:0005737

XM_018058459.1 GO:0003723

XM_005696184.2 GO:0000122 GO:0001106 GO:0005634 GO:0045666 GO:0003682 GO:0045746

XM_005686920.3 GO:0046835 GO:0070062 GO:0004454 GO:0005737

XM_018063853.1 GO:0006694 GO:0005759 GO:0008203 GO:0050660 GO:0055114 GO:0015039 GO:0050661 GO:0005515 GO:0005743 GO:0004324

XM_018044194.1 GO:0070062 GO:0005634 GO:0007165 GO:0048365 GO:0030136 GO:0001701 GO:0043547 GO:0005802 GO:0001750 GO:0042384 GO:0004439 GO:0046856 GO:0005769 GO:0005886 GO:0005096

XR_001918188.1 GO:0070847 GO:0019827 GO:0045944 GO:0001104 GO:0016592

XM_005690924.3 GO:0000287 GO:0016477 GO:0030036 GO:0005524 GO:0004715 GO:0030100 GO:0010976 GO:0010863 GO:0071300 GO:0005623 GO:0051353 GO:0007204 GO:0010506 GO:0030155 GO:0018108 GO:0030145

XM_018058062.1 GO:0003836 GO:0018279 GO:0071354 GO:0052798 GO:0030173 GO:0009311 GO:0097503 GO:0001574

XM_018047322.1 GO:0005654 GO:0042787 GO:0042803 GO:0005737 GO:0004842 GO:0005730 GO:0031463 GO:0005886

NM_001291820.1 GO:0005615 GO:0009897 GO:0046666 GO:0008625 GO:0005164 GO:0000122 GO:2000353 GO:0070231 GO:0016021 GO:0070062 GO:0005634 GO:0005125 GO:0016525 GO:0097527 GO:0043123 GO:0006955 GO:0006919

XM_018057448.1 GO:0005524 GO:0004672 GO:0006468

XM_005694858.3 GO:0031514 GO:0005737

XM_005677354.3 GO:0055085 GO:0016021

XR_001918239.1 GO:0070062 GO:0030209 GO:0003940 GO:0005975

XM_018044125.1 GO:0007179 GO:0046872 GO:0031901 GO:0005545 GO:0007183 GO:0007184

XM_018042373.1 GO:0007399 GO:0030154 GO:0005524 GO:0006816 GO:0042803 GO:0014069 GO:0001666 GO:0046777 GO:0006979 GO:1901897 GO:0004683 GO:0000082 GO:0048169 GO:0005516 GO:0033017 GO:0051259

XM_018059002.1 GO:0030658 GO:0030054 GO:0000302 GO:0016021 GO:0043679 GO:0005886 GO:0030507 GO:0008021 GO:0004725 GO:0043204 GO:0005768 GO:0035773 GO:0030141 GO:0005794 GO:0001553 GO:1990502

XM_013971259.2 GO:0000122 GO:0003677 GO:0046872 GO:0005634 GO:0043392 GO:0045893 GO:0001078

XM_018057570.1 GO:0005634 GO:0044822 GO:0097190 GO:0008270 GO:0006351

XM_018056270.1 GO:0007186 GO:0005605 GO:0070062 GO:0030168 GO:0009181 GO:0016021 GO:0017111 GO:0005886 GO:0017110

XM_018063951.1 GO:0005813

XM_005675569.3 GO:0016021

XM_018041500.1 GO:0003725 GO:0000932 GO:0000993 GO:0005829 GO:0010586 GO:0035280 GO:0003727 GO:0001047 GO:0070578 GO:0005634 GO:0000978 GO:0044822 GO:0045944 GO:0035198 GO:0035068 GO:0010501 GO:0016442 GO:0035278 GO:0000956 GO:0005844 GO:0031054

XM_013967480.2 GO:0040014 GO:0044257 GO:0048666 GO:0035458 GO:0071363 GO:0005829 GO:0009898 GO:0005856 GO:0004252 GO:0008630 GO:0005634 GO:0000785 GO:0005758 GO:0034605 GO:0071300 GO:0030900 GO:2001241 GO:2001269 GO:0007628 GO:0035631 GO:0031966

XM_005698076.3 GO:0007507 GO:0016021 GO:0005789

XM_013975851.2 GO:0046872 GO:0003676

XM_018060678.1 GO:0012505 GO:0019898 GO:0005543

XM_018052606.1 GO:0005654 GO:0006281 GO:0010212 GO:0070876

XM_013966193.2 GO:0046872 GO:0035023 GO:0043547 GO:0005089

XR_001918297.1 GO:0005452 GO:0016021 GO:0045177 GO:0015698 GO:0098656

XM_005690513.3 GO:0016020 GO:0005654 GO:0044822 GO:0005524 GO:0051973 GO:0042254 GO:0005730 GO:0005697 GO:0005739 GO:1990275

XM_018059494.1 GO:0060041 GO:0006910 GO:0045494 GO:0050766 GO:0097500 GO:0007605 GO:0005829 GO:1903546 GO:0005576 GO:0032403

XM_018057232.1 GO:0010944 GO:0001228 GO:0010837 GO:0001842 GO:0060214 GO:0045617 GO:0003682 GO:0048557 GO:0009953 GO:0005634 GO:0001525 GO:0001755 GO:0060716 GO:0010719 GO:0045618 GO:0044212 GO:0001947 GO:0046872 GO:2000647 GO:0045944 GO:0060347 GO:0045746 GO:0051726

XR_001918678.1 GO:0007528 GO:0005938 GO:0031623 GO:0050815 GO:0016874 GO:0031698 GO:0070063 GO:2000650 GO:0048814 GO:0050807 GO:0042110 GO:0070062 GO:0006622 GO:0061630 GO:0050847 GO:0014068 GO:0003151 GO:0002250 GO:0048471 GO:0046824 GO:0048514 GO:0000151 GO:0043162 GO:0003197 GO:0042787 GO:0019089 GO:0010768 GO:0019871 GO:0030948 GO:0005829 GO:0005886 GO:0070064 GO:0050816 GO:0042391 GO:0032801 GO:0005634 GO:0006513 GO:0000785 GO:0042921 GO:0043130 GO:1901016 GO:0019904 GO:0045732 GO:0005794 GO:0044111

XR_001917123.1 GO:0000287 GO:0005524 GO:0006468 GO:0035556 GO:0042384 GO:0036064 GO:0097542 GO:0004674 GO:0042073

XM_018065183.1 GO:0016020 GO:0003725 GO:0042474 GO:0048705 GO:0043085 GO:0005654 GO:0044822 GO:0016301 GO:0006468 GO:0042803 GO:0048471 GO:0042473 GO:0030422 GO:2001244 GO:0034599 GO:0008047 GO:0019899 GO:0031054

XM_005692424.3 GO:0030100 GO:0048471 GO:0032593 GO:0005515 GO:0046323 GO:0007264 GO:0005739 GO:0005525 GO:0015031 GO:0055037 GO:0005886

XM_018062384.1 GO:0009986 GO:0016021 GO:0007409

XM_018038744.1 GO:0045892 GO:0042802 GO:0043621 GO:0016363 GO:0005737 GO:0042405 GO:0042272 GO:0051168 GO:0008022 GO:0008266 GO:0034046

XM_013971316.2 GO:0006470 GO:0005739 GO:0004722

XM_018059179.1 GO:0042393 GO:0000122 GO:1990391 GO:0000977 GO:0003682 GO:0003712 GO:0008270 GO:0000118

XM_018042008.1 GO:0034198 GO:0032006 GO:0005634 GO:0019003 GO:0046982 GO:0005525 GO:0005764 GO:0003924

XR_001918566.1 GO:0010667 GO:0042759 GO:0048714 GO:0005737 GO:0051028 GO:0017124 GO:0001570 GO:0006397 GO:0042552 GO:0005634 GO:0003729 GO:0007286 GO:0010976 GO:0010628 GO:0061158 GO:0042692 GO:0008380 GO:0048255

XM_005698298.3 GO:0070062 GO:0042866 GO:0019470 GO:0008700 GO:0009436 GO:0005739 GO:0042803 GO:0033609

XM_018060386.1 GO:0071156 GO:0030307 GO:1903426 GO:1990314 GO:0006390 GO:0034551 GO:0016021 GO:0002082 GO:0003735 GO:0031930 GO:0032869 GO:0006264 GO:0015218 GO:0006412 GO:0005743 GO:1990519 GO:0051881 GO:0008284

XM_018050581.1 GO:0031410

XM_018063044.1 GO:0005654 GO:0005730 GO:0005737

XM_018043969.1 GO:0043966 GO:0043982 GO:0043981 GO:0008270 GO:0043983 GO:0000123

XM_018054369.1 GO:0035690 GO:0001205 GO:0010499 GO:0016567 GO:0071356 GO:1902037 GO:0042149 GO:0045454 GO:0071499 GO:0046326 GO:0005829 GO:0005886 GO:0001102 GO:1902176 GO:0005634 GO:2000379 GO:0000785 GO:0005813 GO:0045995 GO:0036003 GO:2000352 GO:0006954 GO:1903206 GO:2000121 GO:0019904 GO:0043161 GO:0000980 GO:0032993 GO:0030194

XM_005682848.2 GO:0007186 GO:0050911 GO:0004984 GO:0016021 GO:0005886 GO:0004930

XM_018055863.1 GO:0045920 GO:0005874 GO:0008344 GO:0046983 GO:0019901 GO:0051932 GO:1901998 GO:0042584 GO:0070062 GO:0001917 GO:0044822 GO:0072583 GO:0042802 GO:0043209 GO:0051262 GO:0003924 GO:0030117 GO:0008022 GO:0007605 GO:0005525 GO:0007032

XM_018042271.1 GO:0000462 GO:0034511 GO:0030686 GO:0044822 GO:0005730 GO:0000479 GO:0005525 GO:0003924

XM_018063963.1 GO:0005813

XM_018062059.1 GO:0006397 GO:0034477 GO:0045171 GO:0005634 GO:0090503 GO:0008380 GO:0000175

XM_005674664.3 GO:0055085 GO:0015798 GO:0016021 GO:0007422 GO:0043576 GO:0006020 GO:0005215

XM_018057106.1 GO:0072384 GO:0007165 GO:0005794 GO:0043547 GO:0007030 GO:0051684 GO:0051683 GO:0005096

XM_018044213.1 GO:0032502 GO:0030626 GO:0000398 GO:0000166 GO:0097157 GO:0005689

XM_013969267.2 GO:0017075 GO:0005654 GO:0005874 GO:0019894 GO:0005739 GO:0031982

XM_018060316.1 GO:0006506 GO:0017176 GO:0016021 GO:0005789 GO:0000506

XM_005683154.3 GO:0004674 GO:0005524 GO:0006468

XM_013965867.2 GO:0060179 GO:0005634 GO:0006355 GO:0000987 GO:0001541 GO:0003700 GO:0042803

XM_018063926.1 GO:0006810 GO:0005524 GO:0016021 GO:0005739 GO:0005215 GO:0016887

XM_005701098.3 GO:0030501 GO:0051893 GO:0085029 GO:0016021 GO:0032956

XM_018063949.1 GO:0070016 GO:0005938 GO:0005881 GO:0031625 GO:0030877 GO:0016023 GO:0019901 GO:0090090 GO:0045668 GO:0008285 GO:0005654 GO:0090244 GO:0014069 GO:0035414 GO:0061181 GO:0070411 GO:0006355 GO:0043547 GO:0071407 GO:0001934 GO:0003139 GO:0005886 GO:0042476 GO:0010942 GO:0032423 GO:0030282 GO:0070602 GO:0005813 GO:0001957 GO:0010718 GO:0003413 GO:0009950 GO:0005794 GO:0043570 GO:0048255 GO:0005096 GO:0008013

XM_018043886.1 GO:0005654 GO:0004674 GO:0005524 GO:0005737 GO:0046777

XM_018062253.1 GO:0045665 GO:0000122 GO:0046872 GO:0000978 GO:0044323 GO:0048387 GO:0001078

XM_005694540.3 GO:0002931 GO:0043022 GO:0030425 GO:2000766 GO:0008135 GO:0005634 GO:0005783 GO:0003730 GO:0014069 GO:0071230 GO:0000900 GO:0000166 GO:1990124

XM_018041545.1 GO:0005759 GO:0055129 GO:0004587 GO:0042802 GO:0019544 GO:0034214 GO:0030170 GO:0010121

XM_018064975.1 GO:0008270

XM_018058577.1 GO:0005654 GO:0046966 GO:2000780 GO:0042787 GO:0005737 GO:1901315 GO:0004842 GO:0016874 GO:0009790 GO:0008270

XM_018045558.1 GO:0006098 GO:0004801 GO:0005975 GO:0005737

XM_018039198.1 GO:0070062 GO:0016020 GO:0005615 GO:0010951 GO:0004867

XM_005675183.3 GO:0051897 GO:0005125 GO:0045654 GO:0005179 GO:0038163 GO:1902035 GO:0005576 GO:0070374

XR_001918835.1 GO:0003677 GO:0000791 GO:0006349 GO:0007283 GO:0005737 GO:0001741 GO:0000122 GO:0043046 GO:0003682 GO:0044027 GO:0090116 GO:0005720 GO:0005654 GO:0042802 GO:0016363 GO:0000775 GO:0071230 GO:0043045 GO:0006346 GO:0003886

XM_005693569.2 GO:0051924 GO:0005515 GO:0005509 GO:0005829 GO:0007601 GO:0022400

XR_001917854.1 GO:0003677 GO:0005730 GO:0006355 GO:0016021 GO:0005737

XM_018052298.1 GO:0070062 GO:0004721 GO:0006470

XM_018064863.1 GO:0070588 GO:0045956 GO:0034765 GO:0060371 GO:0002027 GO:0097110 GO:0008332 GO:0086010 GO:0070509 GO:0007268 GO:0010045 GO:0005891

XM_018044353.1 GO:0016020 GO:0005524 GO:0006468 GO:0007030 GO:0031982 GO:0010977 GO:0005794 GO:0004672

XM_018066016.1 GO:0006357 GO:0003677 GO:0046872 GO:0005634 GO:0003712

XM_018040389.1 GO:0005634 GO:0042771 GO:0033209

XM_005686554.3 GO:0004252 GO:0005856 GO:0006508 GO:0005794 GO:0070008

XM_018048867.1 GO:0018095 GO:0005524 GO:0005874 GO:0035082 GO:0005737 GO:0003351 GO:0016874

XM_013971172.2 GO:0045807 GO:0010828 GO:0043065 GO:0055038 GO:0032588 GO:0001934 GO:0044091 GO:0005886 GO:0031115 GO:0090004 GO:0008017 GO:0018230 GO:0035594 GO:0031901 GO:0045121 GO:0045444 GO:0072321

XM_018061047.1 GO:0000781 GO:0005524 GO:0044257 GO:0031625 GO:0010332 GO:0045893 GO:0019901 GO:0006302 GO:0072428 GO:0008630 GO:0016605 GO:0006975 GO:0042803 GO:0046777 GO:0090307 GO:0004674 GO:0001302 GO:0005794 GO:0042176 GO:0000086 GO:0050821

XM_018060526.1 GO:1901409 GO:0000307 GO:0005654 GO:0045944 GO:0016538 GO:0045737 GO:0019901

XM_005682656.3 GO:0005634 GO:0006355 GO:0050896 GO:0043565 GO:0007601

XM_018056619.1 GO:0070062 GO:0005654 GO:0005524 GO:0005874 GO:0051085 GO:0061098 GO:1903751 GO:1903753 GO:1903748 GO:0045944 GO:0005829 GO:0043014

XM_005683204.2 GO:0044308 GO:0034220 GO:0031623 GO:0030666 GO:0048208 GO:0030054 GO:0004971 GO:0033116 GO:0032281 GO:0007616 GO:0043025 GO:0000139 GO:0035235 GO:0060292 GO:0005234 GO:0030165 GO:0043197 GO:0055037 GO:0045211 GO:0008021 GO:0014069 GO:0009986 GO:0005789 GO:0012507 GO:0032591

XM_018064966.1 GO:0007186 GO:0072659 GO:0044292 GO:0016021 GO:0007601 GO:0005886 GO:0004930

XM_018046024.1 GO:0071108 GO:0003677 GO:0004843 GO:0005634 GO:0005737 GO:0070530 GO:0000122 GO:1900181 GO:0008270 GO:0032717 GO:1990380 GO:0043124 GO:0070536 GO:0002385 GO:0035871

XM_018066374.1 GO:0015937 GO:0004633

XM_005677473.3 GO:0048701 GO:0060173 GO:0016021 GO:0001701 GO:0005385 GO:0005886 GO:0071577

XM_018044120.1 GO:0030833 GO:0017048 GO:0000187 GO:0005524 GO:0060996 GO:0005622 GO:0007409 GO:0004674 GO:0071407 GO:0004708

XM_005681624.3 GO:0006406 GO:0000398 GO:0005515 GO:0044822 GO:0005847 GO:0031124 GO:0006369

XM_005677499.3 GO:0070062 GO:0051592 GO:0044822 GO:0005730 GO:0005509 GO:0005829 GO:0042803 GO:0005886

XM_005680381.3 GO:0016310 GO:0007205 GO:0005524 GO:0035556 GO:0005543 GO:0004143 GO:0005829 GO:0005509 GO:0005886

XM_018062731.1 GO:0016021 GO:0005576

XM_018065435.1 GO:0015035 GO:0005788 GO:0051117 GO:0051087 GO:0055114 GO:0045454 GO:0051787 GO:0034663 GO:0034975 GO:0016021 GO:0030544 GO:0001671 GO:0030433 GO:0016671 GO:0001933 GO:0032781 GO:0070059

XM_005676314.3 GO:0007126 GO:0000166 GO:0008494 GO:0045948 GO:0005737

XM_018066302.1 GO:0050731 GO:0045453 GO:0005524 GO:0042802 GO:0090263 GO:1902533 GO:0007264 GO:0004672 GO:0050732 GO:0005739 GO:0005525 GO:0036035

XM_005675012.2 GO:0030308 GO:0001228 GO:0045944 GO:0060770 GO:0000977 GO:0032783

XM_018043593.1 GO:0007602 GO:0005509 GO:0005829 GO:0060040 GO:0005246 GO:0007601 GO:0008594 GO:0046549

NM_001285761.1 GO:0016021 GO:0003841 GO:0005789 GO:0001819 GO:0016024 GO:0006654

XM_013970338.2 GO:0005783 GO:0032781 GO:0001671 GO:0016021 GO:0051117 GO:0090435 GO:0007029

XM_005695088.3 GO:0000287 GO:0072562 GO:0001917 GO:0031594 GO:0045494 GO:0042803 GO:0001750 GO:0005178 GO:0071318 GO:0005829 GO:0005509 GO:0007204 GO:0032420 GO:0042383 GO:0005927

XM_018049980.1 GO:0070062 GO:0005654 GO:0005524 GO:0035556 GO:0005737 GO:0046777 GO:0007616 GO:0045893 GO:0018105 GO:0005730 GO:0004683 GO:0009931 GO:0005516 GO:0043011

XM_018059178.1 GO:0042393 GO:0000122 GO:1990391 GO:0000977 GO:0003682 GO:0003712 GO:0008270 GO:0000118

XM_005678085.3 GO:0007264 GO:0002089 GO:0005622 GO:0033138 GO:0043547 GO:0005085

XM_018051116.1 GO:0016021

XM_013972411.2 GO:0043303 GO:0033004 GO:0005887

XM_018049284.1 GO:0004022 GO:0008270 GO:0055114 GO:0005737

XM_005697012.2 GO:0070062 GO:0005615 GO:0042572 GO:0042802 GO:0005179 GO:0070327 GO:0070324 GO:0036094

XM_018055694.1 GO:0045859 GO:0038180 GO:0019887 GO:0016021 GO:0001701 GO:0030165 GO:0048813

XM_005680147.3 GO:0005654 GO:0000398 GO:0044822 GO:0000166 GO:0008270 GO:0005689

XM_018066127.1 GO:0007186 GO:0035774 GO:0071467 GO:2001206 GO:0004930 GO:0016021 GO:0045656 GO:0005886

XM_005679538.3 GO:0007186 GO:0016021 GO:0001580 GO:0033038 GO:0004930

XM_013963930.2 GO:0070062 GO:0005634 GO:0042645 GO:0015630 GO:0006563 GO:0004372 GO:0003682 GO:0006544 GO:0005743 GO:0035999 GO:0030170

XM_005675493.3 GO:0045116 GO:0005654 GO:0031466 GO:0008270 GO:0097602 GO:0005737 GO:0019788

XM_018051283.1 GO:0016020

XM_018066128.1 GO:0007186 GO:0035774 GO:0071467 GO:2001206 GO:0004930 GO:0016021 GO:0045656 GO:0005886

XM_005697901.3 GO:0046872 GO:0006355 GO:0005622 GO:0003676

XM_018057117.1 GO:0050829 GO:0042802 GO:0030674 GO:0031410 GO:0001920 GO:0005802 GO:0048471 GO:0070530 GO:0043001 GO:0008022 GO:0000042 GO:0090161 GO:0043124 GO:0017137

XM_018052314.1 GO:0005759 GO:0090311 GO:0000993 GO:1902230 GO:0043065 GO:0005681 GO:0004857 GO:0043086 GO:2000003 GO:0030308 GO:0045892 GO:0005654 GO:0044822 GO:0009411 GO:0000790 GO:0090263 GO:0042752 GO:0032784 GO:0031647 GO:0008380 GO:0044609 GO:0043653 GO:0032435

XM_018053577.1 GO:0003723 GO:0000166 GO:0000380

XM_005676392.3 GO:0008154 GO:0005737

XM_005690007.3 GO:0005654

XM_018062409.1 GO:2001275 GO:0008286 GO:0005524 GO:0005938 GO:0031340 GO:0035556 GO:0032587 GO:0032287 GO:0010748 GO:2000147 GO:0001934 GO:0032000 GO:0005634 GO:0072659 GO:0065002 GO:0045725 GO:0004674 GO:0018105 GO:0090314

XM_005685714.2 GO:0019433 GO:0005615 GO:0008203 GO:0034374 GO:0030301 GO:0034372 GO:0070328 GO:0034375 GO:0042632 GO:0004806 GO:0006633

XM_018051832.1 GO:0003197 GO:0001228 GO:0008584 GO:0070410 GO:0003682 GO:0008270 GO:0048468 GO:0090575 GO:0045165 GO:0042493 GO:0001076 GO:0001085 GO:0003290 GO:0060575 GO:0003713 GO:0045944 GO:0000977 GO:0048646

XR_001919624.1 GO:0060041 GO:0030324 GO:0070079 GO:0001822 GO:0005506 GO:0033077 GO:0007166 GO:0055114 GO:0070815 GO:0070078 GO:0048821 GO:0033746 GO:0005730 GO:0005829 GO:0005886 GO:0003727 GO:0005654 GO:0007507 GO:0004872 GO:0042116 GO:0042803 GO:0033749 GO:0018395 GO:0043654 GO:0030529 GO:0048024 GO:0002040

XM_018047437.1 GO:0016567 GO:0008270 GO:0004842

XM_005681398.3 GO:0070062 GO:0005783 GO:0005829 GO:0005739 GO:0032471 GO:0051561

XM_018067201.1 GO:0051015 GO:0070062 GO:0071800 GO:0005938 GO:0030027 GO:0016601 GO:0001726 GO:0007416 GO:0097178 GO:0048812 GO:0019904 GO:0006898 GO:0002102

XM_018057665.1 GO:0071797 GO:0051092 GO:0050852 GO:0060546 GO:0042346 GO:0004842 GO:0043130 GO:0043161 GO:0008270 GO:2001238 GO:0032088 GO:0097039

XM_018053415.1 GO:0016567 GO:0035556 GO:0005737

XM_013965891.2 GO:0070588 GO:0035725 GO:0007612 GO:0031402 GO:0030955 GO:0060292 GO:0007613 GO:0005887 GO:0060291 GO:0005509 GO:0005262 GO:0006874 GO:0008273

XM_013973506.2 GO:0070062 GO:0005634 GO:0055114 GO:0046872 GO:0006177 GO:0000166 GO:0046651 GO:0003938 GO:0071353 GO:0005778

XM_018050520.1 GO:0000187 GO:0030971 GO:0005622

XM_018047763.1 GO:0001228 GO:0005634 GO:0045944 GO:0019902 GO:0010923 GO:0043565

XM_018046695.1 GO:0005730 GO:0005737

XM_018045141.1 GO:0046872 GO:0005634 GO:0006355 GO:0043565 GO:0003700

XM_018062046.1 GO:0045218 GO:0090136 GO:0070062 GO:0007018 GO:0005524 GO:0005874 GO:0008569 GO:0005813 GO:0007030 GO:0005915 GO:0008017 GO:0005794 GO:0005871

XM_018054132.1 GO:0005654 GO:0034446 GO:0005925 GO:0072657 GO:0033622 GO:0005737 GO:0007179 GO:0001725 GO:0005547 GO:0031234 GO:0016055 GO:0007229 GO:0031941 GO:0048041

XM_018046461.1 GO:0007186 GO:0016021 GO:0004930

XM_005687164.3 GO:0007264 GO:0005622 GO:0043547 GO:0005085

XM_018066632.1 GO:0031397 GO:0000244 GO:0031685 GO:0004843 GO:0005634 GO:0042802 GO:0005737 GO:0046872 GO:0006511 GO:0031647 GO:0016579 GO:0005886 GO:0034394

XR_309793.3 GO:0007093 GO:0003697 GO:0003723 GO:0005654 GO:0000784 GO:0010212 GO:0000724 GO:0005737 GO:0070876

XM_018057995.1 GO:0030201 GO:0005783 GO:0003094 GO:0032836 GO:0097421 GO:0010575 GO:0004065 GO:0002063 GO:0008449 GO:0035860 GO:0005886 GO:0009611 GO:0060348 GO:2000345 GO:0060384 GO:0040037 GO:0035413 GO:0090263 GO:0009986 GO:0048706 GO:0014846

XM_013971746.2 GO:0006355 GO:0003700 GO:0005488

XM_005676655.3 GO:0016021

XM_018042272.1 GO:0000462 GO:0034511 GO:0030686 GO:0044822 GO:0005730 GO:0000479 GO:0005525 GO:0003924

XM_018063068.1 GO:0048172 GO:0045202 GO:0032281

XM_018045937.1 GO:0009952 GO:0097191 GO:0005524 GO:0060235 GO:0010842 GO:0010803 GO:0005737 GO:0004672 GO:0007224 GO:0048596 GO:0030182 GO:0016605 GO:0016607 GO:0006468 GO:0042771 GO:0060059 GO:0061072 GO:0034333 GO:0072577 GO:0008284

XM_013966140.2 GO:0044822

XM_005700133.3 GO:0016021

XM_018044172.1 GO:0016021

XM_005681512.3 GO:0016021

XM_018061062.1 GO:0016020 GO:0042827 GO:0007596 GO:0042470 GO:0005764 GO:0042803 GO:0031085 GO:0030318 GO:0050821 GO:0007040

XM_018060470.1 GO:0050872 GO:0035019 GO:0045893 GO:0030853 GO:0000122 GO:0022008 GO:0017053 GO:0030512 GO:0043565 GO:0060021 GO:0033613 GO:0005634 GO:0043586 GO:0046872 GO:0003713 GO:0043457 GO:0046332 GO:0090336

XM_005678878.3 GO:0070062 GO:0001649 GO:0005524 GO:2001243 GO:0033687 GO:0010569 GO:0005737 GO:0000228 GO:0071479 GO:0051726

XM_018038383.1 GO:2000124 GO:0009395 GO:0070062 GO:0030336 GO:0032281 GO:0004620 GO:0060292 GO:0047372 GO:0005739 GO:0046464 GO:0046889

XM_005686065.3 GO:0003677 GO:0046872 GO:0005634 GO:0006355

XM_018057991.1 GO:0030201 GO:0005783 GO:0003094 GO:0032836 GO:0097421 GO:0010575 GO:0004065 GO:0002063 GO:0008449 GO:0035860 GO:0005886 GO:0009611 GO:0060348 GO:2000345 GO:0060384 GO:0040037 GO:0035413 GO:0090263 GO:0009986 GO:0048706 GO:0014846

XM_013965804.2 GO:0016310 GO:0000828 GO:0033857 GO:0003993 GO:0016311

XM_018051117.1 GO:0016021

XM_018040741.1 GO:0070062 GO:0005615 GO:0010951 GO:0004867 GO:0042383 GO:0005581 GO:0005578

XM_005683753.3 GO:0070062 GO:0042572 GO:0001758 GO:0055114 GO:0005737 GO:0004029

XM_005683194.3 GO:0006979 GO:0006878 GO:0015680 GO:0032767 GO:0016531 GO:0005737

NM_001314293.1 GO:0016337 GO:0030216 GO:0030509 GO:0005509 GO:0007156 GO:0016021 GO:0005886 GO:0001942 GO:0030057

XM_005678556.3 GO:0046872 GO:0004527 GO:0044822 GO:0000467

XM_013962568.2 GO:0030308 GO:0000922 GO:0031116 GO:0045737 GO:0008017 GO:0045893 GO:0030496 GO:0007080 GO:0005829 GO:0051301 GO:0060236 GO:0001578 GO:0005876

XM_018046590.1 GO:0000122 GO:0005634 GO:0043565 GO:0001227

XM_018059076.1 GO:0004565 GO:0005975

XM_018062382.1 GO:0023014 GO:0000278 GO:0071407 GO:0005524 GO:0005925 GO:0060996 GO:0004702 GO:0005737

XM_005699655.3 GO:0070062 GO:0016021 GO:0005622 GO:0042771 GO:0071480 GO:0060548

XM_018066573.1 GO:0035095 GO:0005892 GO:0098655 GO:0030054 GO:0007274 GO:0015464 GO:0042166 GO:0004889 GO:0007271 GO:0045211

XM_018044382.1 GO:0070062 GO:0005615

XM_018049897.1 GO:0030331 GO:0060065 GO:0001077 GO:0031624 GO:0043028 GO:0046881 GO:0001555 GO:0000122 GO:0019101 GO:0007338 GO:0033686 GO:0006309 GO:0043280 GO:0005634 GO:0000978 GO:0001541 GO:0042703 GO:0060014 GO:0002074 GO:0048048 GO:0045944 GO:0008210

XM_018046740.1 GO:0005925 GO:0008270 GO:0005737

XM_018049286.1 GO:0008270 GO:0055114 GO:0016491

XM_018052690.1 GO:0071300 GO:0045930 GO:0045666 GO:0008219 GO:0007050 GO:0005737

XM_018040427.1 GO:0097233 GO:0055085 GO:0005615 GO:0005524 GO:0006869 GO:0042626 GO:0016021 GO:0005886

XM_018053078.1 GO:0000287 GO:0070062 GO:0044208 GO:0005737 GO:0005525 GO:0046040 GO:0005886 GO:0004019

XM_005688510.3 GO:0006397 GO:0005815 GO:0005515 GO:0044822 GO:0006355 GO:0000166 GO:0016607 GO:0008380

XM_018061650.1 GO:0032930 GO:0008083 GO:0005737 GO:0045216 GO:1900126 GO:0009611 GO:0007182 GO:0005114 GO:0045599 GO:0010800 GO:0045662 GO:0002062 GO:0031334 GO:0050921 GO:0045944 GO:0033138 GO:0043536 GO:0002244 GO:0006754 GO:0010862 GO:0034714 GO:0006611 GO:0008156 GO:0030308 GO:0032801 GO:0006954 GO:0050680 GO:0043537 GO:0010763 GO:2000679 GO:0097191 GO:1901666 GO:0000060 GO:0032740 GO:0032570 GO:0032967 GO:0010936 GO:0017015 GO:0022408 GO:0032355 GO:0007173 GO:0031663 GO:0003823 GO:0048642 GO:0045892 GO:0007093 GO:0043552 GO:0043932 GO:0001933 GO:0048298 GO:0051897 GO:0060965 GO:0035307 GO:0030214 GO:0007183 GO:0007050 GO:0071407 GO:0085029 GO:0019049 GO:0034713 GO:0070723 GO:0005634 GO:0072562 GO:0043406 GO:0060391 GO:0010718 GO:0030501 GO:0009986 GO:0007435 GO:0001948 GO:0008284

NM_001314210.1 GO:0043462 GO:0030172 GO:0003779 GO:0005523 GO:0030899 GO:0048306 GO:0006942 GO:0005861 GO:0003009 GO:0031013

XM_005690613.3 GO:0070062 GO:0007155 GO:0005615 GO:0030199 GO:0008285 GO:0005578

XM_018041436.1 GO:0005524 GO:0005938 GO:0005007 GO:0005887 GO:0008543 GO:0018108 GO:0031012 GO:0005654 GO:0042803 GO:0046777 GO:0009986 GO:0048701 GO:0010518 GO:0017134 GO:0043410 GO:0008284

XM_018043919.1 GO:0006541 GO:0005524 GO:0044210 GO:0005739 GO:0003883

XM_005675194.3 GO:0016486 GO:0004222 GO:0016021 GO:0030659

XM_005690923.3 GO:0000287 GO:0016477 GO:0030036 GO:0005524 GO:0004715 GO:0030100 GO:0010976 GO:0010863 GO:0071300 GO:0005623 GO:0051353 GO:0007204 GO:0010506 GO:0030155 GO:0018108 GO:0030145

XM_005690492.3 GO:0015630 GO:0006360 GO:0000120

XM_005679048.3 GO:0046872 GO:0005654 GO:0044822 GO:0005737

XM_018045570.1 GO:0005524 GO:0032300 GO:0030983 GO:0001541 GO:0007283 GO:0007292 GO:0000710 GO:0007131 GO:0003684 GO:0000794

XM_018057140.1 GO:0008289 GO:0000062

XM_018048309.1 GO:0005615 GO:0019902 GO:0010923 GO:0016021

XM_005700705.3 GO:0016021 GO:0016747

XM_018060419.1 GO:0005827 GO:0005813 GO:0035853 GO:0042787 GO:0005737 GO:0007067 GO:0004842 GO:0032465 GO:0031463

XM_018049232.1 GO:0042802 GO:0008483 GO:0005739 GO:0050459 GO:0030170

XM_018039062.1 GO:0070062 GO:0031415 GO:0004596 GO:0052858 GO:0005515 GO:0043967 GO:0006474 GO:0071962 GO:0010485

XM_005676358.3 GO:0005622

XM_005691461.3 GO:0070062 GO:0005634 GO:0010951 GO:0044822 GO:0005524 GO:0004867 GO:0005737 GO:0019901 GO:0008289

XM_018059799.1 GO:0070062 GO:0008601 GO:0045121 GO:2001241 GO:0060561 GO:0006461 GO:0034047

XM_018064514.1 GO:0035094 GO:0005892 GO:0098655 GO:0030054 GO:0007274 GO:0015464 GO:0042166 GO:0004889 GO:0007271 GO:0042391 GO:0045211

XM_018064713.1 GO:0016020 GO:0003677 GO:0072368 GO:0003714 GO:0051225 GO:0042826 GO:0016580 GO:0035257 GO:0072362 GO:0017053 GO:0046329 GO:0001102 GO:0005876

XM_018048237.1 GO:0003688 GO:0016020 GO:0005654 GO:0000784 GO:0005524 GO:0006270 GO:0032508 GO:0042555 GO:0003682 GO:0007049 GO:0003678

XM_005676340.3 GO:0009055 GO:0016614 GO:0051537 GO:0005506 GO:0050660 GO:0051287 GO:0055114

XM_018039099.1 GO:0000122 GO:0046872 GO:0005634 GO:0001206 GO:0000980 GO:0005739

NM_001285679.1 GO:0003419 GO:0040014 GO:0050880 GO:0030823 GO:0001503 GO:0005179 GO:0006182 GO:0030814 GO:0051447 GO:0009791 GO:0005576 GO:0003418 GO:0007168 GO:1900194

XM_018050756.1 GO:0046425 GO:0006342 GO:0005654 GO:2000677 GO:0051726 GO:0008134 GO:0034729 GO:0031151 GO:0016568

XM_018050270.1 GO:0007281 GO:0005634 GO:0017091 GO:0000166 GO:0060965 GO:0005737

XM_018052234.1 GO:0005025 GO:0030335 GO:0060317 GO:0010717 GO:0030199 GO:0008354 GO:0008584 GO:0009791 GO:0004702 GO:0002088 GO:0000186 GO:0001937 GO:0031396 GO:0043393 GO:0001525 GO:0043542 GO:0060017 GO:0005114 GO:0060037 GO:0032924 GO:0018107 GO:2001235 GO:0005768 GO:0018105 GO:0048663 GO:0070411 GO:0009952 GO:0051897 GO:0005524 GO:0001822 GO:0030307 GO:0045893 GO:0043235 GO:0007179 GO:0001824 GO:2001237 GO:0010862 GO:0005923 GO:0016021 GO:0001938 GO:0060021 GO:0005886 GO:0050431 GO:0070723 GO:0051491 GO:0060391 GO:0048538 GO:0048701 GO:0032331 GO:0048844 GO:0042118

XM_018052251.1 GO:0008017 GO:0044772

XM_018048378.1 GO:0000287 GO:0018105 GO:0005524 GO:0004674 GO:0051128 GO:0015629 GO:0035556 GO:0005737

XM_018065644.1 GO:0005634 GO:0005874 GO:0032273 GO:0046785 GO:0043209 GO:0001578 GO:0008017 GO:0048471

XM_005698308.3 GO:0000166 GO:0035145

NM_001285728.1 GO:1902895 GO:0051541 GO:0031625 GO:0051216 GO:0003705 GO:0001892 GO:0021987 GO:0001837 GO:0097411 GO:0061030 GO:0001755 GO:0001525 GO:0042826 GO:0016607 GO:0003151 GO:0003208 GO:0070244 GO:0045648 GO:0019896 GO:0010870 GO:0007595 GO:0032007 GO:0045926 GO:0006089 GO:0046886 GO:0032909 GO:1903599 GO:0042541 GO:0006110 GO:0032364 GO:0001922 GO:0043565 GO:1903377 GO:0050790 GO:0060574 GO:0046716 GO:0061072 GO:0016239 GO:0008134 GO:0035035 GO:0002248 GO:0006879 GO:0021502 GO:0035774 GO:0001077 GO:0019901 GO:2001054 GO:0014850 GO:0071347 GO:0002052 GO:0061419 GO:0071542 GO:2000378 GO:0061298 GO:0008542 GO:0010575 GO:0030502 GO:0046982 GO:1903715 GO:0048546 GO:0051879 GO:0010634 GO:0043619 GO:0035162 GO:0005829 GO:0031514 GO:0090575 GO:0001076 GO:0030949 GO:0032963 GO:0035257 GO:0001947

XM_005691995.3 GO:0003729 GO:0000398 GO:0017091 GO:0042826 GO:0005813 GO:0051262 GO:0006369 GO:0006378 GO:0042803 GO:0042382 GO:0016787 GO:0006379 GO:0005849

XM_018064980.1 GO:0009952 GO:0001739 GO:0016573 GO:0001701 GO:0000122 GO:0035102 GO:0048704 GO:0016604 GO:0003682 GO:0008270 GO:2001234 GO:0070301

XM_005675610.2 GO:0005615 GO:0009117 GO:0046085 GO:0012506 GO:0006772 GO:0006144 GO:0016021 GO:0016311 GO:0005886 GO:0060168 GO:0005765 GO:0070062 GO:0052642 GO:0005634 GO:0008253 GO:0042802 GO:0030175 GO:0051930 GO:0042131

XM_005675042.3 GO:0021762 GO:0005813

XM_018039778.1 GO:0061630 GO:0008270 GO:0005622 GO:0032436 GO:0000209 GO:0016874 GO:0031624

XM_005699571.3 GO:0007186 GO:0050911 GO:0005549 GO:0004984 GO:0016021 GO:0005886 GO:0004930

XM_005692050.3 GO:0030331 GO:0005615 GO:0070016 GO:0000932 GO:0000122 GO:2000036 GO:0090503 GO:0010606 GO:0033147 GO:0005778 GO:0004535 GO:0032947 GO:0044822 GO:0042974 GO:0030014 GO:0048387 GO:0060213 GO:0035195

XM_005684592.3 GO:0043015 GO:0042802 GO:0034453 GO:0008017

XM_005688844.3 GO:0070062 GO:0005929

XM_018045854.1 GO:0051005 GO:0010468 GO:0030379 GO:0014902 GO:0006895 GO:0008625 GO:0048011 GO:0048406 GO:0032509 GO:0016050 GO:0005829 GO:0008333 GO:0006897 GO:0016021 GO:0007218 GO:0005769 GO:0005886 GO:0030140 GO:0032868 GO:0045599 GO:0010465 GO:0048471 GO:0009986 GO:0038180 GO:0032596 GO:0046323 GO:0005905 GO:0019899 GO:0048227

XM_005684800.3 GO:0045892 GO:0003677 GO:0044822 GO:0016607 GO:2000144 GO:0043620 GO:0005730 GO:2001244 GO:2001022

XM_005678321.2 GO:0006612 GO:0006501 GO:0000422 GO:0016485 GO:0005829 GO:0004197 GO:0051697 GO:0005576 GO:0044804 GO:0000045

XM_018045837.1 GO:0046658 GO:0007409

XM_018057179.1 GO:0002011 GO:0007165

XM_018058431.1 GO:2000273 GO:0001162 GO:0005737 GO:0046983 GO:0000122 GO:0003682 GO:0010906 GO:0032922 GO:0005654 GO:0000978 GO:0004402 GO:0016573 GO:0032870 GO:0030375 GO:0016922 GO:0030522 GO:0035257 GO:0045944 GO:0045475 GO:0008134

XM_018051197.1 GO:0008063 GO:0032496 GO:0001960

XM_013970367.2 GO:0045930 GO:0070935 GO:0015030 GO:0003730 GO:0005737

XM_005691285.2 GO:0005615 GO:0005125 GO:0042102 GO:0005622 GO:0032740 GO:0030890 GO:0048469 GO:0042517 GO:0042511 GO:0005134 GO:0045954

XM_005684604.3 GO:0098532 GO:0036124 GO:0000182 GO:0006306 GO:0000122 GO:0034773 GO:0005730 GO:0043967 GO:0080182 GO:0000183 GO:0005720

XM_005699609.2 GO:0005634 GO:0005813 GO:0005886 GO:0005737

XM_005681606.3 GO:0016021

XM_018053541.1 GO:0008083 GO:0045666 GO:0060113 GO:0060235 GO:2000137 GO:0030513 GO:0048392 GO:0032092 GO:0070700 GO:0072205 GO:0060592 GO:0008201 GO:0072192 GO:0007224 GO:0000186 GO:0002320 GO:0010453 GO:0072104 GO:0007182 GO:0045843 GO:0060363 GO:0045662 GO:0072015 GO:0070244 GO:0045944 GO:0048663 GO:0002043 GO:0003130 GO:0061151 GO:0072101 GO:0021978 GO:0000122 GO:0033088 GO:0072125 GO:0072097 GO:0010862 GO:0003139 GO:0001938 GO:0042056 GO:0003337 GO:0001958 GO:2000105 GO:0072138 GO:0045669 GO:0045603 GO:0060687 GO:0072193 GO:0030218 GO:0060395 GO:0072200 GO:0060686 GO:0045839 GO:0050680 GO:2000007 GO:0039706 GO:0060272 GO:0090184 GO:0060433 GO:0005615 GO:0061155 GO:0060502 GO:0043407 GO:0007500 GO:0010595 GO:2000005 GO:0005160 GO:0032967 GO:0043065 GO:0009791 GO:0021983 GO:0005125 GO:0035116 GO:0021904 GO:0072161 GO:0010159 GO:0007281 GO:0035990 GO:0042487 GO:0060197 GO:0001843 GO:0050918 GO:0060684 GO:0051150 GO:0060440 GO:0003014 GO:0042733 GO:0003323 GO:0061149 GO:0055020 GO:0030225 GO:0071893 GO:0061036 GO:0045606 GO:0060391 GO:0035993 GO:0005578 GO:0061047 GO:0048701 GO:0030501 GO:0032331 GO:0048661 GO:0060449 GO:0070374 GO:0048286 GO:0043401 GO:0009948 GO:0030224 GO:0060503

XM_018064515.1 GO:0006886 GO:0005634 GO:0005524 GO:0004674 GO:0006468 GO:0001672 GO:0035556

XM_018060868.1 GO:0001764 GO:0015630 GO:0001578

XM_005685851.3 GO:0008542 GO:0007595 GO:0007412 GO:0000981 GO:0061379 GO:0021510 GO:0006357 GO:0005515 GO:0021794 GO:0001756 GO:0043565 GO:0061030 GO:0033504 GO:0005634 GO:0001655 GO:0022029 GO:0021855 GO:0043524 GO:0061374 GO:0061381

XM_018053946.1 GO:0030317 GO:0018095 GO:0009566 GO:0007288 GO:0005813

XM_005686525.3 GO:0004674 GO:0005524 GO:0006468

XM_005700128.3 GO:0070062

XM_018054873.1 GO:0016021

XM_018040497.1 GO:0006006 GO:0016020 GO:0016477 GO:0005634 GO:0010212 GO:0051646 GO:0016049 GO:0005737 GO:0006915 GO:0008283 GO:0001934 GO:0006974

XM_005688283.3 GO:0045121 GO:0005887 GO:0016492 GO:0008344 GO:0007218 GO:0043066

XM_005674673.3 GO:0070062 GO:0005524 GO:0009113 GO:0006189 GO:0005737 GO:0046872 GO:0004641 GO:0004637 GO:0004644

XM_018042987.1 GO:0050995 GO:0019915 GO:0045444 GO:0030176 GO:0034389

XM_018066497.1 GO:0016021

XM_005682490.2 GO:0005856 GO:0005737

XM_005678732.3 GO:0005922 GO:0060713 GO:0007154 GO:0016021 GO:0060708 GO:0060707

XM_005700158.3 GO:0005654 GO:0042043 GO:0019898 GO:0005813 GO:0045921 GO:0071985 GO:0005543 GO:0006886 GO:0050714 GO:0005768 GO:0030141 GO:0046676 GO:0017137

XM_005676652.3 GO:0055085 GO:0008028 GO:0016021 GO:0015718

XM_018044259.1 GO:0035235 GO:0005654 GO:0034220 GO:0005234 GO:0030054 GO:0004971 GO:0032281 GO:0045211

XM_018052860.1 GO:0016477 GO:0003064 GO:0055131 GO:0000981 GO:0035690 GO:0055075 GO:0031625 GO:0097110 GO:0003682 GO:1902282 GO:0060307 GO:1902303 GO:0086091 GO:0005251 GO:0048471 GO:0000155 GO:0003197 GO:0023014 GO:2000504 GO:1901381 GO:0006357 GO:0000160 GO:0086010 GO:0008283 GO:0043565 GO:0003199 GO:0008076 GO:0005634 GO:0005242 GO:0042803 GO:0098915 GO:0097623 GO:0009986 GO:0030529

XM_013967823.2 GO:0046872

XM_005693135.2 GO:0008270 GO:0016021

XM_018055731.1 GO:0016020 GO:0003677 GO:0061470 GO:0010608 GO:0005737 GO:0009791 GO:0048536 GO:0008270 GO:1901224 GO:0000209 GO:0005654 GO:0003729 GO:0048535 GO:0035264 GO:0004842 GO:0009986 GO:0043029 GO:0042098 GO:0060173 GO:0001782 GO:0048286

XM_018044150.1 GO:0000122 GO:0000978 GO:0005654 GO:0001078

XM_005684248.3 GO:0046872 GO:0006355 GO:0005622 GO:0002230 GO:0003676

XR_001918028.1 GO:0009952 GO:0071837 GO:0005634 GO:0006355 GO:0048562 GO:0051216 GO:0043565 GO:0003700

XM_005680014.3 GO:0070062 GO:0044822 GO:0005524 GO:0000375 GO:0071013 GO:0005730 GO:0004004 GO:0005682 GO:0010501

XM_005678091.1 GO:0061087 GO:0035064 GO:0005737 GO:0035098 GO:0048863 GO:0000122 GO:0061086 GO:0019827 GO:0045944 GO:0008270 GO:0007379

XM_018048288.1 GO:0016020 GO:0044822 GO:0001731 GO:0016282 GO:0071541 GO:0006446 GO:0003743 GO:0001732 GO:0033290

XM_005679435.3 GO:0044822 GO:0000166

XM_018059049.1 GO:0000214 GO:0016310 GO:0005524 GO:0006388 GO:0031124 GO:0005737 GO:0035087 GO:0051736 GO:0021695 GO:0030423 GO:0005849 GO:0051733

XM_005675652.2 GO:0005829 GO:0007283 GO:0006366

XM_005690737.3 GO:0051444 GO:2000157 GO:1902958 GO:0008233 GO:0006517 GO:0051583 GO:0005747 GO:0032091 GO:0006914 GO:1903136 GO:0070062 GO:0003729 GO:0016605 GO:0070491 GO:0007005 GO:0043005 GO:2000277 GO:0045944 GO:0036471 GO:0033138 GO:1902236 GO:0036470 GO:0005759 GO:0036478 GO:0090073 GO:2000825 GO:0018323 GO:0005886 GO:1990422 GO:0019955 GO:0016532 GO:0000785 GO:0005758 GO:0042803 GO:0006469 GO:1903197 GO:0051899 GO:0032435 GO:1903181 GO:2000679 GO:0005783 GO:1903428 GO:0008344 GO:1901671 GO:0010273 GO:0044388 GO:0033234 GO:0097110 GO:0050681 GO:0046826 GO:0019249 GO:0045340 GO:0044390 GO:1903094 GO:0042743 GO:0032757 GO:1990381 GO:1903178 GO:1901984 GO:0050787 GO:1903384 GO:0045121 GO:1903168 GO:1903200 GO:0046295 GO:0010629 GO:1902177 GO:0005829 GO:0007338 GO:0019900 GO:1900182 GO:2001268 GO:0019243 GO:0019172 GO:0060081 GO:0051920 GO:1903122 GO:0050727 GO:0044297 GO:1903190 GO:0003713 GO:1903135 GO:0051881 GO:0050821

XM_005686707.2 GO:0044822 GO:0005743 GO:0070124 GO:0043024 GO:0006417 GO:0070125 GO:0019843

XM_005682607.3 GO:0010008 GO:0005765 GO:0005654 GO:0072657 GO:0048499 GO:0043195 GO:0048007 GO:0061088 GO:0000139 GO:0006886 GO:0048490 GO:0033365 GO:0051138 GO:0030123 GO:0035646

XR_001917122.1 GO:0008542 GO:0043113 GO:0046580 GO:0016358 GO:0043524 GO:0043547 GO:0043408 GO:0005737 GO:0007389 GO:0043198 GO:0050771 GO:0048169 GO:0031235 GO:0005096

XM_018063485.1 GO:0003677 GO:0005634 GO:0008270

XM_018056407.1 GO:0016020 GO:0007264 GO:0005622 GO:0043547 GO:0005085

XM_018050413.1 GO:2000045 GO:0045666 GO:0046982 GO:0005737 GO:0030890 GO:0000122 GO:0051091 GO:0002326 GO:0090575 GO:0070644 GO:0000978 GO:0070888 GO:0070491 GO:0031435 GO:0000790 GO:0042803 GO:0001078 GO:0033152 GO:0003713 GO:0043425 GO:0045944 GO:0045787

XM_018046080.1 GO:0003677 GO:0046872 GO:0005654 GO:0007064 GO:0005737 GO:0000790 GO:0051382

XM_018040649.1 GO:0042832 GO:0045630 GO:0016064 GO:0043032 GO:0043306 GO:0090197 GO:0004896 GO:0002532 GO:0002639 GO:0043235 GO:0016021 GO:0045626 GO:0019221 GO:1901741

XM_005684181.3 GO:0035339 GO:0046512 GO:0004758 GO:0016021 GO:0006686 GO:0046511 GO:0030170 GO:0046513

XR_001295751.2 GO:0016020 GO:0046907 GO:0001103 GO:0007420 GO:0032436 GO:0036064 GO:0006357 GO:0007224 GO:0060021 GO:0005634 GO:0001654 GO:0007507 GO:0005813 GO:0034464 GO:0001750 GO:0060173 GO:0035058 GO:0045444 GO:0008104 GO:0005930

XM_005678201.2 GO:0051015 GO:0030334 GO:0005925 GO:0051493 GO:0008307

XM_005699194.3 GO:2000045 GO:0005654 GO:0008283 GO:0005737

XM_018048005.1 GO:0043547 GO:0043066 GO:0046427 GO:0060749 GO:0033601 GO:0005730 GO:0045944 GO:0007264 GO:0005739 GO:0005525 GO:0005096

XM_005686878.3 GO:0005654 GO:0005739 GO:0003924

XM_018050313.1 GO:0005634 GO:0000166 GO:0008270 GO:0003676

XM_018063006.1 GO:0046872 GO:0006355 GO:0005622 GO:0003676

XM_005688846.3 GO:0070062 GO:0005929

XM_013965960.2 GO:0005634 GO:1900025 GO:0030336 GO:0035413 GO:0032587 GO:0005737 GO:0030177 GO:0090303 GO:0046627 GO:2000114 GO:2000393 GO:0030837 GO:0010977 GO:0035024 GO:1900028 GO:0008013

XM_005684815.3 GO:0019905 GO:0005737

XM_005688140.3 GO:0003723 GO:0005737

XM_005698968.3 GO:0090181 GO:0016021 GO:0008206 GO:0030301

XM_005684095.3 GO:0046330 GO:0051897 GO:0001768 GO:0005615 GO:0031735 GO:0034695 GO:0071356 GO:0043547 GO:0071346 GO:0071347 GO:0045860 GO:0002548 GO:0008009 GO:0007186 GO:0048247 GO:0090023 GO:0071731 GO:0005622 GO:0070098 GO:0006954 GO:0042346 GO:0002408 GO:0043552 GO:2000107 GO:0070374 GO:0098586 GO:0051209

XM_018049684.1 GO:0016605 GO:0005545 GO:0005635 GO:0035973 GO:0016234 GO:0016239 GO:0046872 GO:0034274 GO:0097635 GO:0003831

XM_018046868.1 GO:0006820 GO:0008643 GO:0055085 GO:0030176 GO:0022857

XM_013967602.2 GO:0004252 GO:0005856 GO:0006508 GO:0005794 GO:0070008

XM_018050582.1 GO:0031410

XM_018056670.1 GO:0000124 GO:0035948 GO:0003712

XM_005674852.3 GO:0016021

XR_001295516.2 GO:0005509

XM_005678169.2 GO:0005739

XM_005677572.2 GO:0008544

XM_018054684.1 GO:0030125 GO:0006897 GO:0015031 GO:0005886

XM_018050328.1 GO:0070062 GO:0050916 GO:0005881 GO:0071786 GO:0032596 GO:0032386 GO:0005887 GO:0050913 GO:0005789 GO:0002230 GO:0031883

XM_018047834.1 GO:0023014 GO:0000155 GO:0005249 GO:0000160 GO:0005887 GO:0005622 GO:0071805 GO:0042391

XM_013964476.2 GO:0031267 GO:0005052 GO:0005829 GO:0043235

XM_005685135.3 GO:0008422 GO:0005783 GO:0005903 GO:1901657 GO:0005975 GO:0016021

XM_018053744.1 GO:0045295 GO:0030335 GO:0030877 GO:0007094 GO:0032587 GO:0007050 GO:0019901 GO:0090090 GO:0000281 GO:0043065 GO:0005913 GO:0019887 GO:0007026 GO:0051988 GO:0051010 GO:0005923 GO:0016328 GO:0006974 GO:0008285 GO:0005634 GO:0005813 GO:0045736 GO:0031274 GO:0030027 GO:0016342 GO:0045732 GO:0000776 GO:0006461 GO:0008013

XM_018048773.1 GO:0034993 GO:0000784 GO:0030335 GO:0042802 GO:0005639 GO:0005521 GO:0051642 GO:0000794 GO:0031022 GO:0090286 GO:0090292 GO:0006998

XM_018054777.1 GO:0008083 GO:0005615 GO:0045666 GO:0030901 GO:0048853 GO:0005160 GO:0021527 GO:0045893 GO:0048608 GO:0010862 GO:0060571 GO:0007411 GO:0048754 GO:0045165 GO:0005125 GO:0030509 GO:0043408 GO:0032924 GO:0042803 GO:0060395 GO:0021509 GO:2001051 GO:0040007 GO:0042981 GO:0030855 GO:0022612

XM_005674873.1 GO:0016021

XM_005689031.3 GO:0000978 GO:0045944 GO:0001077

XM_005688524.2 GO:0070062 GO:0007165 GO:0005737

XM_018044859.1 GO:0005759 GO:0004657 GO:0010133 GO:0055114 GO:0071949

XM_018041656.1 GO:0003723 GO:0006400 GO:1990481 GO:0009982

XM_005686996.3 GO:0003677 GO:0071339 GO:0000083 GO:0005667 GO:0003700

XM_018061533.1 GO:0007156 GO:0005509 GO:0016021 GO:0005886

XM_005688330.3 GO:0016020 GO:0005783 GO:0006511 GO:0000502 GO:0005829 GO:0070628 GO:1901799

XM_018063584.1 GO:0071377 GO:0004967 GO:0007166 GO:0070873 GO:0042593 GO:0005887 GO:0042594 GO:0007188

XM_018059071.1 GO:0055085 GO:0006955 GO:0016021

XM_005680366.2 GO:0005509

XM_018057654.1 GO:0016020 GO:0019905 GO:0042113 GO:0005737

XM_005674658.3 GO:0005249 GO:0015459 GO:0016324 GO:0016021 GO:0071805 GO:0086091 GO:0002070

XM_005685393.3 GO:0090305 GO:0004519 GO:0003676

XM_018066948.1 GO:0070062 GO:0006730 GO:0009258 GO:0047105 GO:0055114 GO:0019145 GO:0009058 GO:0016742 GO:0016155 GO:0005739

XM_018063971.1 GO:0005783 GO:0090330 GO:0035408 GO:0097110 GO:0002159 GO:0070555 GO:0045651 GO:0045766 GO:0070062 GO:0048471 GO:0018105 GO:0043536 GO:0035403 GO:0005524 GO:0045785 GO:0031666 GO:2000707 GO:0005829 GO:0007194 GO:0008270 GO:0022400 GO:0001938 GO:0005634 GO:0097381 GO:0045931 GO:0004697 GO:0034351 GO:0010613 GO:0007190 GO:0031966 GO:0070374 GO:0019899

XM_005700907.3 GO:0045028 GO:0035589 GO:0005887

XM_018052445.1 GO:0070062 GO:0006997 GO:0005615 GO:0005794 GO:0019216 GO:0016021

XM_005687472.3 GO:0016021

XM_005675406.3 GO:0006614 GO:0005784 GO:0016021

XM_018065521.1 GO:0005524 GO:0006468 GO:0043547 GO:0007185 GO:0043065 GO:0004674 GO:0005829 GO:0035023 GO:0019899 GO:0005089

XM_005700344.3 GO:1900087 GO:0070062 GO:0005654 GO:0035518 GO:0031625 GO:0005737 GO:0003684 GO:0006511 GO:0045732 GO:0031465 GO:0070914

XM_005675281.3 GO:0005761 GO:0006412 GO:0003735

XM_005696983.3 GO:0046872 GO:0005634 GO:0006355 GO:0043565 GO:0003700

XM_018052301.1 GO:0070062 GO:0004721 GO:0006470

XM_018057375.1 GO:0016021

XM_005678341.3 GO:0008395 GO:0020037 GO:0019369 GO:0005506 GO:0055114 GO:0016712 GO:0008392

XM_005699919.3 GO:0009987

XM_005698317.3 GO:0006810 GO:0006412 GO:0016021 GO:0003735 GO:0005739

XM_005691492.3 GO:0035725 GO:0016021 GO:0005272 GO:0015992 GO:0015252

XM_005676421.3 GO:0005887 GO:0007268 GO:0007218 GO:0042923 GO:0007187 GO:0004930 GO:0043005

XM_018040348.1 GO:0048009

XM_013970488.2 GO:0006886 GO:0032588 GO:0019904 GO:0070273 GO:0005829 GO:0050708

XM_005699135.3 GO:0007217 GO:0005887 GO:0007268 GO:0016497 GO:0070472 GO:0007166 GO:0045987

XM_018053259.1 GO:0032012 GO:0010923 GO:0043547 GO:0005086

XM_005677471.3 GO:0048701 GO:0060173 GO:0016021 GO:0001701 GO:0005385 GO:0005886 GO:0071577

XR_001295582.2 GO:0016607 GO:0015030 GO:0019013 GO:0071001

XM_013967248.2 GO:0017176 GO:0016021 GO:0000506

XM_005696275.3 GO:0042327 GO:0005737

XM_005692642.3 GO:0070584 GO:0019216 GO:0040007 GO:0007601 GO:0050905 GO:0005739

NM_001314253.1 GO:0070062 GO:0005634 GO:0005524 GO:0006165 GO:0004550 GO:0005737 GO:0009142 GO:0009041 GO:0006221 GO:0006207 GO:0004127

XM_013976556.2 GO:0016020

XM_005688390.2 GO:0008289 GO:0045087 GO:0005737

XM_005682406.3 GO:0070062 GO:0005615 GO:0005179

XM_018050668.1 GO:0043154 GO:0043027 GO:0005737

XM_005686632.3 GO:0031123 GO:0016020 GO:0004652 GO:0003723 GO:0005654 GO:0043631 GO:0005737 GO:0046872

XM_018047786.1 GO:0048017 GO:0005765 GO:0016324 GO:0006654 GO:0035091 GO:0005768 GO:0004630 GO:0030334 GO:0032534 GO:0030139 GO:0005794 GO:0050764

XM_005674937.3 GO:0046034 GO:0070062 GO:0005765 GO:0005524 GO:0016324 GO:0043209 GO:0005902 GO:0015991 GO:0005829 GO:0005739 GO:0033180 GO:0046961

XM_018042526.1 GO:0016031 GO:0031012 GO:0031307 GO:0030943 GO:0005742 GO:0015266 GO:0070096 GO:0044233 GO:0051082 GO:0015450 GO:0030150 GO:0016236

XM_018038202.1 GO:0002020 GO:0005615 GO:0010951 GO:0005578 GO:0046872 GO:0008191 GO:0010033 GO:0051045 GO:0007219

NM_001285539.1 GO:0032725 GO:2000667 GO:0042802 GO:0005576 GO:2000778 GO:0006915 GO:0019841

XM_018065700.1 GO:0070779 GO:0043200 GO:0016597 GO:0009416 GO:0006537 GO:0043025 GO:0046677 GO:0016021 GO:0005886 GO:0009611 GO:0089711 GO:0042493 GO:0050806 GO:0009449 GO:0017153 GO:0043205 GO:0048667 GO:0051938 GO:0043005 GO:0016595 GO:0009986 GO:0031223 GO:0010035 GO:0021545 GO:0007605 GO:0050885 GO:0005314 GO:0002230

XM_005690462.3 GO:0008277 GO:0043547 GO:0005886 GO:0005737 GO:0005096

XM_005675963.3 GO:0005634 GO:0021766 GO:0043524 GO:0048715 GO:0042475 GO:0000122 GO:0021893 GO:0000977 GO:0003682 GO:0021882 GO:0009954 GO:0045746 GO:0048706 GO:0021544

XM_018053193.1 GO:0016571 GO:0050798 GO:0016605 GO:0006338 GO:0016363 GO:0001227 GO:0000122 GO:0060004 GO:0008544 GO:0043367 GO:0043374 GO:0000977 GO:0003682 GO:0005720

XM_005684022.3 GO:0003677 GO:0046872 GO:0005634 GO:0007275 GO:0045944 GO:0001077

XM_018062024.1 GO:0070062 GO:0016324 GO:0005829 GO:0016021 GO:0006814 GO:0015377 GO:1902476

XM_018060779.1 GO:0016021 GO:2001238 GO:0005739

XM_013967464.2 GO:0050727 GO:0032755 GO:0004909 GO:0045582 GO:0016021 GO:0070498

XM_013976477.2 GO:0045184 GO:0010738 GO:0030317 GO:0051018 GO:0005634 GO:0007178 GO:0044458 GO:0048471 GO:0030018 GO:0097228 GO:0035686

XM_018063144.1 GO:0034198 GO:0032008

XM_005675525.3 GO:0045779 GO:2001205 GO:1900181 GO:0005923 GO:0016021 GO:0005198 GO:0048565 GO:0005886 GO:0071847

XM_018061263.1 GO:0008217 GO:0050850 GO:0005524 GO:0097190 GO:0098655 GO:0030054 GO:0005887 GO:0071318 GO:0055119 GO:0035590 GO:0005765 GO:0070062 GO:0004931 GO:0005639 GO:0055117 GO:0048471 GO:0045296 GO:0010614 GO:0051899 GO:0010524 GO:0034405 GO:0001614

XM_005682508.3 GO:0046872 GO:0002943 GO:0044822 GO:0017150 GO:0005829 GO:0050660 GO:0055114

XM_018042413.1 GO:0016324 GO:0006970 GO:0034465 GO:0060083 GO:0043065 GO:0005901 GO:0030007 GO:0060072 GO:0042391 GO:0008076 GO:0070062 GO:0005249 GO:0045794 GO:0034765 GO:0001666 GO:0003779 GO:0046872 GO:0051592 GO:0071805

XM_005683007.3 GO:0005634 GO:0000166 GO:0008270 GO:0003676

XM_018040723.1 GO:0006851 GO:0007186 GO:0001228 GO:0005634 GO:0010832 GO:0019722 GO:0007267 GO:0007030 GO:0042149 GO:0042803 GO:0042593 GO:0048469 GO:0045944 GO:0000977 GO:0030968 GO:0048312

NM_001314340.1 GO:0006013 GO:0030246 GO:0008270 GO:0004559

XM_005676623.3 GO:0043687 GO:0004252 GO:0010954 GO:0034620 GO:0043066 GO:0044322 GO:0034644 GO:0048515 GO:0030176 GO:0045732 GO:0031293 GO:0051047

XM_005691246.3 GO:0007156 GO:0005509 GO:0016021 GO:0007420 GO:0005886

XM_005697159.2 GO:0007186 GO:0016021 GO:0004977 GO:0030819 GO:0005886

XM_018058022.1 GO:0008286 GO:0030100 GO:0043407 GO:0098554 GO:2000646 GO:1902202 GO:0030948 GO:0046875 GO:0097443 GO:0016021 GO:0008270 GO:0005886 GO:0005769 GO:0031532 GO:0044822 GO:0061098 GO:0030971 GO:0004725 GO:0007257 GO:0070373 GO:0035791 GO:0033157 GO:1990264 GO:1902236

XM_005676561.3 GO:0006616 GO:0008270 GO:0005622

XM_018043756.1 GO:0030154 GO:0002088

XM_013963554.2 GO:0006821 GO:0005247 GO:0016021

XM_018056614.1 GO:0005615 GO:0008283 GO:0070140 GO:0032183 GO:0015030 GO:0016926 GO:0030576

XM_005686084.3 GO:0000462 GO:0005654 GO:0044822 GO:0032040 GO:0005730

XR_001917210.1 GO:0006506 GO:0017176 GO:0016021

XM_005692482.3 GO:0001558 GO:0005665 GO:2001243 GO:0001106 GO:0000122 GO:0003682 GO:0071363 GO:0010923 GO:0071383 GO:0005739 GO:0009615 GO:0051219

XM_018060830.1 GO:0000226 GO:0033043 GO:0031175 GO:0005813 GO:0005516 GO:0051011 GO:0030507

XM_018049986.1 GO:0030335 GO:0048008 GO:0030054 GO:0010762 GO:0044331 GO:0005737 GO:0035426 GO:0036119 GO:0070102 GO:0031234 GO:0050904 GO:0033007 GO:0034446 GO:0038028 GO:0015630 GO:0000790 GO:0030027 GO:0046777 GO:0036006 GO:0008289 GO:0008157 GO:0000278 GO:0005524 GO:0034614 GO:0042503 GO:0032496 GO:0038109 GO:0001932 GO:0010591 GO:0015629 GO:0030838 GO:0006935 GO:0031532 GO:0000226 GO:0004715 GO:0051092 GO:0005154 GO:0038095 GO:0042058 GO:0008284

XM_005694739.3 GO:0008299 GO:0004421 GO:0005654 GO:0005886 GO:0005737

XM_005699629.3 GO:0005515 GO:0005887 GO:0034765 GO:0015467 GO:0010107

XM_018066094.1 GO:0005634 GO:0009267 GO:0048742 GO:0032436 GO:0005737 GO:2000060 GO:0006913 GO:0000209

XM_018065948.1 GO:0006508

XM_005686361.3 GO:0005634 GO:0005829 GO:0019901

XM_018059919.1 GO:0045859 GO:0050731 GO:0030425 GO:0031641 GO:0048714 GO:0051896 GO:0009791 GO:0003179 GO:0006112 GO:0060048 GO:0050882 GO:0001032 GO:0031669 GO:0001031 GO:0005765 GO:0031529 GO:0031929 GO:0016605 GO:0046777 GO:0035264 GO:0045792 GO:0018107 GO:0007281 GO:0031931 GO:0010592 GO:0018105 GO:0090559 GO:0006207 GO:0055013 GO:0046889 GO:0045945 GO:0071456 GO:0043200 GO:0051534 GO:0005524 GO:0045727 GO:0016049 GO:0010831 GO:0003007 GO:1901838 GO:0012505 GO:0031932 GO:0005829 GO:0001030 GO:0030838 GO:0051219 GO:0043022 GO:0006281 GO:0032868 GO:0043087 GO:0051496 GO:0004674 GO:0001156 GO:0045670 GO:0016242

XM_018066404.1 GO:0007030 GO:0042803 GO:0005801 GO:0000301 GO:0000139 GO:0016021 GO:0031985 GO:0017137

XM_018061874.1 GO:0090409 GO:0090410 GO:0006633 GO:0005739

XM_018038707.1 GO:0005654 GO:0005737

XM_018041934.1 GO:0046330 GO:0010008 GO:0007252 GO:0032755 GO:0071260 GO:0097191 GO:0034123 GO:0050729 GO:0043330 GO:0003725 GO:0032760 GO:0032735 GO:0005887 GO:0045078 GO:0004888 GO:0034346 GO:0002756 GO:0005769 GO:0034138 GO:0005765 GO:0051092 GO:0042802 GO:0032757 GO:0051607 GO:0097527 GO:0042346 GO:0045359 GO:0045080 GO:0045944 GO:0005789 GO:0002730 GO:0045356

XM_013965603.2 GO:0030425 GO:0001822 GO:0046449 GO:0004222 GO:0008238 GO:0005903 GO:0050435 GO:0019233 GO:0008270 GO:0016021 GO:0071345 GO:0005886 GO:0071493 GO:0030424 GO:0070062 GO:0005925 GO:0008021 GO:0042277 GO:0090399 GO:0006508 GO:0044306 GO:0071492

XM_018066446.1 GO:0016021 GO:0035542

XM_005676781.3 GO:0005654 GO:0010212 GO:0006355 GO:0016576 GO:0035335 GO:0005813 GO:0004725 GO:0006302 GO:0046872 GO:0007275 GO:0005667 GO:0045739

XM_018042311.1 GO:0051533 GO:0035690 GO:0034097 GO:0035774 GO:0017156 GO:0031987 GO:0046983 GO:0001915 GO:0030018 GO:0008144 GO:0006470 GO:0033173 GO:0005516 GO:0005955 GO:0030315 GO:0035176 GO:0007507 GO:0006468 GO:0043029 GO:0045944 GO:0005509 GO:0030346 GO:0001946 GO:0030217 GO:0033192

XM_005698193.3 GO:0008395 GO:0008203 GO:0005506 GO:0016021 GO:0006633 GO:0055114

XM_018064485.1 GO:0005634 GO:0014898 GO:0042826 GO:0001077 GO:0003682 GO:0045944 GO:0043565 GO:0008134

XM_005675161.3 GO:0050727 GO:0050766 GO:0070062 GO:0072562 GO:0010951 GO:0030502 GO:0006953 GO:0004869

NM_001285748.1 GO:0030425 GO:0007420 GO:0043025 GO:0003179 GO:0043610 GO:0001032 GO:0005765 GO:0031929 GO:0016605 GO:0031998 GO:0014736 GO:0046777 GO:0045792 GO:0018105 GO:0090559 GO:0045429 GO:0005789 GO:0090335 GO:0046889 GO:0045945 GO:0051534 GO:0043200 GO:0005524 GO:0043278 GO:0021510 GO:0045727 GO:0003007 GO:1901838 GO:0001938 GO:0032868 GO:0007569 GO:0030163 GO:0060999 GO:1900034 GO:0043087 GO:0004674 GO:0045670 GO:0001156 GO:0005942 GO:0042220 GO:0048255 GO:0016242 GO:0045859 GO:0060252 GO:0050731 GO:0060135 GO:0031641 GO:0048714 GO:0019901 GO:0009791 GO:0060048 GO:0050882 GO:0031669 GO:0001031 GO:0031529 GO:0035176 GO:1901216 GO:0035264 GO:0018107 GO:0032095 GO:0031931 GO:0007281 GO:0001933 GO:0010592 GO:0006207 GO:0048015 GO:0061051 GO:0008542 GO:0031397 GO:0071456 GO:0051897 GO:0005741 GO:0031295 GO:0007050 GO:0010831 GO:0000139 GO:0007616 GO:0031932 GO:0014042 GO:0005829 GO:0007584 GO:0001030 GO:0030838 GO:0051219 GO:0043022 GO:0006281 GO:0042060 GO:0051496 GO:0005979 GO:0019904 GO:0048661

XM_018042333.1 GO:0043547 GO:0051056 GO:0005096

XM_005683368.3 GO:0030424 GO:0005230 GO:0007214 GO:0030054 GO:1902711 GO:0009791 GO:0005887 GO:0030534 GO:0007268 GO:0071420 GO:0004890 GO:0005254 GO:0045202 GO:1902476

XR_001917497.1 GO:0000287 GO:0031532 GO:0050321 GO:0005634 GO:0005783 GO:0005524 GO:0005813 GO:0051117 GO:0007409 GO:0019901 GO:0030010 GO:0018105 GO:0004674 GO:0070059 GO:0061178 GO:0000086

XM_018049087.1 GO:0004843 GO:0006511 GO:0005730 GO:0015630 GO:0016579 GO:0008270 GO:0090231

XM_018064048.1 GO:0005615 GO:0007586 GO:0007267 GO:0042742 GO:0005184 GO:0001664 GO:0007631 GO:0032098 GO:0007218 GO:0005516

XM_018056910.1 GO:0005790 GO:0030425 GO:0016042 GO:0005634 GO:0004435 GO:0035556 GO:0014069 GO:0005509 GO:0004871

XM_018053561.1 GO:0046872 GO:0005524 GO:0003774 GO:0016459 GO:0043547 GO:0035556 GO:0005096

XM_018042032.1 GO:0070062 GO:0002178 GO:0018230 GO:0005795 GO:0005515 GO:0043001 GO:0031228 GO:0050821

XM_018057457.1 GO:0016021

XM_018059341.1 GO:0043154 GO:0031434 GO:0032715 GO:0035774 GO:0031625 GO:0002092 GO:0016023 GO:0032092 GO:0031143 GO:0032088 GO:0005654 GO:0035025 GO:0045944 GO:0032717 GO:0033138 GO:0090240 GO:0031397 GO:0043027 GO:0043547 GO:0007602 GO:0005829 GO:0005159 GO:0005886 GO:0000785 GO:0044212 GO:0043161 GO:0005905 GO:0031701 GO:0070374 GO:0002031 GO:0008134 GO:0005096 GO:0043149

XM_018057618.1 GO:0030425 GO:0051216 GO:0045672 GO:0060789 GO:0040032 GO:0031683 GO:0031698 GO:0006112 GO:0040015 GO:0004871 GO:0030819 GO:0043950 GO:0070062 GO:0042493 GO:0035116 GO:0007606 GO:0003924 GO:0050890 GO:0071880 GO:0031748 GO:0005525 GO:0043547 GO:0005829 GO:0035255 GO:0005159 GO:0007191 GO:0005886 GO:0001958 GO:0071514 GO:0031852 GO:0071107 GO:0045669 GO:0006306 GO:0001894 GO:0046872 GO:2000828 GO:0048701 GO:0030133 GO:0051430 GO:0070527

XM_018052645.1 GO:0046872 GO:0003676

XM_018059495.1 GO:0060041 GO:0005929 GO:0006910 GO:0045494 GO:0035091 GO:0050766 GO:0097500 GO:0007605 GO:1903546 GO:0005829 GO:0005576 GO:0032403

XM_018056934.1 GO:0045022 GO:0005524 GO:0006895 GO:0001704 GO:0080025 GO:0007173 GO:0005829 GO:0008543 GO:0043325 GO:0030705 GO:0005769 GO:0017137 GO:0008574 GO:0005871 GO:0007492 GO:0007018 GO:0032801 GO:0032266 GO:0008017 GO:0005547 GO:0001919

XM_018055619.1 GO:0008143 GO:0010494 GO:0016020 GO:0003677 GO:0005524 GO:0006355 GO:0043330 GO:0032508 GO:0003725 GO:0006302 GO:0003682 GO:0003712 GO:0071920 GO:0006397 GO:0072669 GO:0044822 GO:0006388 GO:0004527 GO:0004004 GO:0033677 GO:0007275 GO:0090305 GO:0010501 GO:0009615

XM_018049475.1 GO:0050714 GO:0048015 GO:0005737 GO:0098592

XM_018047852.1 GO:0070062 GO:0044822 GO:0005524 GO:0000375 GO:0071013 GO:0005730 GO:0004004 GO:0005682 GO:0010501

XM_018054276.1 GO:0005654 GO:0044822 GO:0000166

XM_005690616.3 GO:0010882 GO:0016323 GO:0086009 GO:0016324 GO:0031402 GO:0005391 GO:0030955 GO:1903288 GO:0055119 GO:1901018 GO:0014704 GO:0070062 GO:0043209 GO:0005622 GO:0023026 GO:0007155 GO:0008022 GO:0010248 GO:0046034 GO:0010468 GO:0005524 GO:1903281 GO:0051117 GO:0030007 GO:0008144 GO:0006883 GO:0006874 GO:0001671 GO:0072659 GO:1903278 GO:0005890 GO:0032781 GO:0042383 GO:0050821 GO:0002230

XM_005675006.3 GO:0070062 GO:0005654 GO:0032391 GO:0005813 GO:0045494 GO:0001750 GO:0048496 GO:0042384 GO:0045171 GO:0005516 GO:0019899

XM_005676107.3 GO:0005025 GO:0030335 GO:0003143 GO:0048179 GO:2000017 GO:0016361 GO:0004702 GO:0001755 GO:0007368 GO:0001702 GO:0060037 GO:0030509 GO:0018107 GO:0007281 GO:0032926 GO:0045944 GO:0000082 GO:0001569 GO:0045177 GO:0023014 GO:0060923 GO:0005524 GO:0048185 GO:0007179 GO:0001707 GO:2001237 GO:0010862 GO:0050431 GO:0061445 GO:0001701 GO:0042803 GO:0045669 GO:0002526 GO:0051145 GO:0030501 GO:0046872 GO:0003289 GO:0003183 GO:0046332

XM_013967603.2 GO:0004252 GO:0005856 GO:0006508 GO:0005794 GO:0070008

XM_018057410.1 GO:0042552 GO:0021549 GO:0070062 GO:0005615 GO:0040014 GO:0004872 GO:0043473 GO:0016021

XM_005681660.3 GO:0004252 GO:0006508 GO:0005887 GO:0005576

XM_018053809.1 GO:0080154 GO:0022409 GO:0003676 GO:0016021 GO:0005576 GO:1902093 GO:0034113 GO:0007338

XR_001918318.1 GO:0042802 GO:0008483 GO:0005739 GO:0030170

XM_018056208.1 GO:0006486 GO:0015012 GO:0016021 GO:0006024

XM_018038998.1 GO:0046330 GO:0044257 GO:0031625 GO:0051260 GO:0071356 GO:0032760 GO:0071363 GO:0070231 GO:0045651 GO:2001238 GO:0060545 GO:0070926 GO:0042802 GO:0032757 GO:1901026 GO:0045944 GO:2001240 GO:0036289 GO:0005524 GO:0097342 GO:0043235 GO:1990000 GO:0005739 GO:0043124 GO:0051291 GO:0070513 GO:0051092 GO:0097527 GO:0043123 GO:0031264 GO:0004674 GO:0005123 GO:2000377 GO:0032403

XM_013971602.2 GO:0046872 GO:0003676

XR_001917226.1 GO:0044822 GO:0070742 GO:0047485 GO:0016607 GO:0015030 GO:0071013

XM_018065409.1 GO:0031965 GO:0044822 GO:0005887 GO:0015129 GO:0008028 GO:0015629 GO:0035879

XM_005694547.3 GO:0000470 GO:0000460 GO:0044822 GO:0005730 GO:0030687

XM_018054162.1 GO:0030215 GO:0014912 GO:0016021 GO:0021591 GO:0014911

XM_018038582.1 GO:0003677 GO:0046872 GO:0005634

XM_018053610.1 GO:0007093 GO:1990423

XM_018060140.1 GO:0005874 GO:0021954 GO:0002162 GO:0008017 GO:0005737 GO:0048813 GO:0043005 GO:0005875 GO:0005730 GO:0001578

XM_013976022.2 GO:0009987

XM_018059734.1 GO:0008270

XM_018061293.1 GO:0070062 GO:0005856 GO:0007015 GO:0007264 GO:0005525 GO:0005886 GO:0005737

XM_018048894.1 GO:0046907 GO:0005654 GO:0031965 GO:0005643 GO:0005737

NM_001285755.1 GO:0046983

XM_018046636.1 GO:0043154 GO:0016020 GO:0003677 GO:0045109 GO:0031072 GO:0051087 GO:0060710 GO:0060717 GO:0030018 GO:0005829 GO:0045892 GO:0006457 GO:0005654 GO:0001671 GO:0030036 GO:0048471 GO:0032781 GO:0060715 GO:0032880 GO:0051082 GO:0030198 GO:0034504 GO:0090084

XM_013967789.2 GO:0031965

XM_018057154.1 GO:0030488 GO:0008175 GO:0005737

XM_013965713.2 GO:0016021 GO:0005886

XR_001919147.1 GO:0043547 GO:0005086 GO:0034237 GO:0000139 GO:0005730 GO:0005829 GO:0005654 GO:0007030 GO:0048471 GO:0090303 GO:2000114 GO:0030837 GO:0030532 GO:0032012 GO:0017022 GO:0034260 GO:0090284

XM_018047962.1 GO:0016020 GO:0003729 GO:0000166 GO:0030529 GO:0051262 GO:0006378 GO:0005849 GO:0042382

XM_018047761.1 GO:0001228 GO:0005634 GO:0045944 GO:0019902 GO:0010923 GO:0043565

XM_013971232.2 GO:0070062 GO:0005216 GO:0034220 GO:0016021 GO:0051117 GO:0005789 GO:0050790

XM_013974831.2 GO:0008305 GO:0070062 GO:0007155 GO:0009986 GO:0005615 GO:0007229 GO:0001948 GO:0010668

XM_018062633.1 GO:0005654 GO:0035307 GO:0006378 GO:0005886 GO:0005737

XM_018061057.1 GO:0006478 GO:0070062 GO:0005783 GO:0005794 GO:0008476

XM_018056307.1 GO:0000287 GO:0005524 GO:0005802 GO:0048666 GO:0000139 GO:0045332 GO:0048194 GO:0016021 GO:0004012 GO:0005886

XM_018061054.1 GO:0006810 GO:0008289 GO:0070062 GO:0005794

XM_005699124.3 GO:0044822 GO:0005524 GO:0005730 GO:0004386 GO:0005886

XM_005693553.3 GO:0006810 GO:0016021 GO:0005739

XM_018059368.1 GO:0008022 GO:0016021 GO:0042803

XM_005678524.3 GO:0005634 GO:0003700 GO:0045893

XM_005695115.3 GO:0045665 GO:0000122 GO:0005634 GO:0046982 GO:0016568

XM_005676777.3 GO:0070847 GO:0006357 GO:0061630 GO:0001104 GO:0016592 GO:0000151 GO:0016567 GO:0006369

XM_018041065.1 GO:0042802 GO:0039689 GO:0000045 GO:0005930 GO:0000421

XM_018041956.1 GO:0045892 GO:0003677 GO:0016605 GO:0004402 GO:0006334 GO:0030099 GO:0070776 GO:0045893 GO:0043966 GO:0090398 GO:0003713 GO:0005794 GO:0008270 GO:0008134 GO:0000786

XM_018056435.1 GO:0070062 GO:0005788 GO:0006986 GO:0051603 GO:0070417 GO:0043066 GO:0051087 GO:0019901 GO:0051787 GO:0031205 GO:0004860 GO:0005829

XM_005686521.3 GO:0046872 GO:0005524 GO:0044822 GO:0004386

XM_005677732.3 GO:0051782 GO:0048008 GO:0030216 GO:0031625 GO:0005737 GO:0006979 GO:0000122 GO:0006606 GO:0004857 GO:0043086 GO:0071228

XM_013964537.2 GO:0031623 GO:0045502 GO:0032026 GO:0043030 GO:0014059 GO:0042416 GO:0070555 GO:0051585 GO:0042802 GO:0043205 GO:0060732 GO:0035067 GO:0034599 GO:0033138 GO:0006919 GO:0031092 GO:0042393 GO:0000287 GO:0043027 GO:0070495 GO:0016234 GO:0032769 GO:0060291 GO:0010642 GO:0005739 GO:0010040 GO:0060079 GO:0005886 GO:0016491 GO:0030424 GO:0001921 GO:0005543 GO:0005509 GO:0048169 GO:0043154 GO:0014048 GO:0005615 GO:0045920 GO:0005938 GO:0008344 GO:0030054 GO:0007006 GO:0045807 GO:0050544 GO:0010517 GO:0030426 GO:0043014 GO:0005507 GO:0071902 GO:0001774 GO:1903285 GO:0031115 GO:1903284 GO:0042493 GO:0043524 GO:0071280 GO:0050812 GO:0032410 GO:0048471 GO:0051622 GO:0008198 GO:0006638 GO:0032496 GO:0048156 GO:0051612 GO:0001956 GO:0005829 GO:0034341 GO:0008270 GO:0015629 GO:0050808 GO:0031648 GO:0051219 GO:0005634 GO:0048488 GO:0019894 GO:0051281 GO:0006644 GO:0040012 GO:0042775

XM_018048470.1 GO:0031410 GO:0034185 GO:0017147 GO:0005109 GO:0043025 GO:0019534 GO:0021987 GO:1901998 GO:0014029 GO:0021794 GO:0042813 GO:0005769 GO:0071901 GO:0090244 GO:0035261 GO:2000055 GO:0042475 GO:0090263 GO:0071397 GO:0045944 GO:0001843 GO:0045787 GO:0071936 GO:0021587 GO:0003344 GO:0030901 GO:0034392 GO:0060026 GO:0043235 GO:0090009 GO:0005901 GO:0051091 GO:0009880 GO:0090118 GO:0016021 GO:0060021 GO:0045202 GO:0060535 GO:0072659 GO:0030917 GO:0014033 GO:0019210 GO:0042803 GO:0060059 GO:0009986 GO:0005041 GO:0005794 GO:0007268 GO:0060325 GO:0090245

XM_018051596.1 GO:0030324 GO:0043588 GO:0004222 GO:0030199 GO:0007283 GO:0032964 GO:0005578 GO:0016485 GO:0008270 GO:0043206 GO:0030574

XM_018054789.1 GO:0046872 GO:0021521 GO:0003676

XM_018065785.1 GO:2001240 GO:0048485 GO:0008083 GO:0005615 GO:0072107 GO:0030116 GO:0005160 GO:0051584 GO:0043235 GO:0007179 GO:0007169 GO:0031175 GO:0001759 GO:0009611 GO:0001755 GO:0043524 GO:0042803 GO:0021784 GO:0071549 GO:0032770 GO:0030432 GO:0072108 GO:0001941 GO:0048484 GO:0090190 GO:0045944 GO:0048255 GO:0008284

XM_018056922.1 GO:0042393 GO:0005654 GO:0031965 GO:0007059 GO:0051225 GO:0043547 GO:0005737 GO:0000790 GO:0007088 GO:0005087 GO:0000794 GO:0000082 GO:0007052 GO:0031492

XM_018053970.1 GO:0070062 GO:0005615 GO:0019838 GO:0050436 GO:0071953 GO:0001527 GO:0005509 GO:0008201 GO:0043206

XM_005680336.3 GO:0044822 GO:0000166

XM_005679263.3 GO:0046872 GO:0006355 GO:0043565 GO:0003700

XM_005683824.3 GO:0008152 GO:0016021 GO:0003824

XM_005697039.3 GO:0005634 GO:0046982 GO:0005737 GO:0019904 GO:0005509 GO:0097228 GO:0097229 GO:0019899 GO:0035686

XR_001917010.1 GO:0000214 GO:0005654 GO:0006388 GO:0005813 GO:0000213 GO:0005737 GO:0003676 GO:0016829 GO:0090502

XM_018050201.1 GO:0043402 GO:0000978 GO:0005815 GO:0005654 GO:0001077 GO:0042921 GO:0045944 GO:0005819 GO:0008270 GO:0005739 GO:0016568 GO:0038051 GO:1990239

XM_018047797.1 GO:0016020 GO:0044822 GO:0000166 GO:0007010 GO:0022604

XM_018041090.1 GO:0035023 GO:0043547 GO:0005089

XM_018049575.1 GO:0008083 GO:0005615 GO:0060326 GO:0006955 GO:0008009 GO:0006954

XM_005686027.3 GO:2000010 GO:0002092 GO:0005741 GO:0016021 GO:0070699 GO:0032927 GO:0006605 GO:0001937 GO:0008593 GO:0010596 GO:1903671 GO:0048471 GO:0070373 GO:0007266 GO:0009986 GO:0032926

XM_018065590.1 GO:0005654 GO:0015629 GO:0005739

XM_005691549.3 GO:0045824 GO:0046872

XM_005677351.2 GO:0005765 GO:0045684 GO:0042335 GO:0045055 GO:0070268 GO:0032588 GO:0031069 GO:0061436 GO:0016021 GO:0002070

XM_018051746.1 GO:0016021

XM_005675639.3 GO:0072687 GO:0035082 GO:0000794 GO:0005829 GO:0007286 GO:0001520

XM_018061111.1 GO:0048266 GO:0005654 GO:0005925 GO:0043547 GO:0032403 GO:0005096

XM_018053891.1 GO:0030970 GO:0016021

XM_018058579.1 GO:0046872

XM_018041794.1 GO:0090141 GO:0046872 GO:0005815 GO:0005737

XM_018064737.1 GO:0004028 GO:0005783 GO:0050061 GO:0046577 GO:0005777 GO:0055114 GO:0033306 GO:0006081 GO:0016021 GO:0052814 GO:0070062 GO:0006714 GO:0007417 GO:0004030 GO:0008544 GO:0005743 GO:0007422

XM_018055153.1 GO:0000185 GO:0005524 GO:0008349 GO:0034612 GO:0009411 GO:0005737

XM_018052112.1 GO:0019079 GO:0019903 GO:0031625 GO:0032436 GO:0006734 GO:0006302 GO:0010918 GO:0034098 GO:0035800 GO:0042288 GO:0016887 GO:0070062 GO:0036435 GO:0005654 GO:0071712 GO:2001171 GO:0044822 GO:0043209 GO:1990381 GO:0048471 GO:0008289 GO:0019985 GO:0031334 GO:0005811 GO:0006919 GO:0030970 GO:0000502 GO:0005524 GO:1903715 GO:0016567 GO:0034214 GO:0005829 GO:0018279 GO:0072389 GO:0035861 GO:1903007 GO:0019904 GO:0070842

XM_018059496.1 GO:0060041 GO:0006910 GO:0045494 GO:0050766 GO:0097500 GO:0007605 GO:0005829 GO:1903546 GO:0005576 GO:0032403

XM_005675163.3 GO:0051661 GO:0003779 GO:0030334 GO:0007010 GO:0051684 GO:0008360 GO:0031616

XM_018050831.1 GO:0007186 GO:0019229 GO:0005622 GO:0032496 GO:0045987 GO:0006954 GO:0004960 GO:0019932 GO:0016021 GO:0005886

XM_005693434.3 GO:0000070 GO:0034501 GO:0051301 GO:0000818 GO:0051382

XM_018046725.1 GO:0023014 GO:0055131 GO:0035690 GO:0055075 GO:1901381 GO:0031625 GO:0097110 GO:0000160 GO:0086010 GO:1902282 GO:0060307 GO:0008076 GO:0005242 GO:0005251 GO:1902303 GO:0086091 GO:0042803 GO:0048471 GO:0000155 GO:0098915 GO:0097623 GO:0009986

XM_013964217.2 GO:0005765 GO:0005770 GO:0015578 GO:0005802 GO:0005537 GO:0048471 GO:0016021 GO:0015761

XM_005678037.3 GO:0070062 GO:0010043 GO:0016023 GO:0005385 GO:0061088 GO:0048471 GO:0005794 GO:0016021 GO:0071577

XM_013969639.2 GO:0097084 GO:0005615 GO:0050829 GO:0045906 GO:0005737 GO:0005179 GO:0043116 GO:0060712 GO:0050830 GO:0045766 GO:0030819 GO:2001214 GO:0007507 GO:0060670 GO:0048589 GO:0002031 GO:0001843 GO:0019731 GO:0008284

XR_001297097.2 GO:0005739

XM_018050223.1 GO:0005518 GO:0016021 GO:0010811 GO:0005886 GO:0005604

XM_005695496.3 GO:0016020

XM_018047207.1 GO:0031290 GO:0086080 GO:0030424 GO:0001525 GO:0019227 GO:0045162 GO:0007417 GO:0008104 GO:0016021 GO:0034113 GO:0030506 GO:0010975 GO:0045202 GO:0005886

XM_018058964.1 GO:0007186 GO:0050911 GO:0004984 GO:0016021 GO:0005886 GO:0004930

XM_018057568.1 GO:0005654 GO:0030054

XM_005695795.3 GO:0070062 GO:0030036 GO:0044822 GO:0005925 GO:0005903 GO:0003382 GO:0042802 GO:0005737 GO:0003334 GO:0003779 GO:0001725 GO:0007519 GO:0005886

XM_018055353.1 GO:0050850 GO:0045065 GO:0009897 GO:0051607 GO:0007166 GO:0002456 GO:0042803 GO:0016021

XM_018055404.1 GO:0031965 GO:0000056 GO:0005487 GO:0008565 GO:0005642 GO:0046825 GO:0010824 GO:0005737 GO:0000055 GO:0008536 GO:0042176 GO:0030529 GO:0034504 GO:0000776

XM_005684039.3 GO:0050731 GO:0046330 GO:0043267 GO:0045428 GO:0010595 GO:0007172 GO:0010752 GO:2000060 GO:0031234 GO:0045766 GO:0004871 GO:0032960 GO:0008285 GO:0005856 GO:0005654 GO:0005925 GO:0070098 GO:0043066 GO:0030027 GO:0008360 GO:2000249 GO:0048471 GO:0043552 GO:0071300 GO:0050848 GO:0002040 GO:0051000 GO:0001954 GO:0005524 GO:0038083 GO:0071498 GO:0030502 GO:0051279 GO:0045638 GO:2000538 GO:0007229 GO:0030838 GO:0033209 GO:0045453 GO:0004715 GO:0030826 GO:0010976 GO:2000114 GO:0042976 GO:0010758 GO:0048010 GO:0070374 GO:0002315 GO:0008284

XM_005676853.3 GO:0006376 GO:0005654 GO:0044822 GO:0000166 GO:0005829 GO:0016482 GO:0048025

XM_018052124.1 GO:0031386 GO:0070062 GO:0016925 GO:0016605 GO:0005737

XM_018065768.1 GO:0031146 GO:0031398 GO:0042803 GO:0005737 GO:0004842 GO:0019005 GO:0000209 GO:0000723

XM_018060550.1 GO:0000151 GO:0030433 GO:0016567 GO:0031625 GO:0016021 GO:0061631

XR_001919076.1 GO:0045892 GO:0000793 GO:0031493 GO:0005654 GO:0042802 GO:0000785 GO:0035064 GO:0045652 GO:0032093 GO:0007088 GO:0008270 GO:0003700 GO:0005886 GO:0016568

XM_018042079.1 GO:0006952 GO:0005576

XM_005677500.3 GO:0043542 GO:0005509 GO:0042802

XM_018062106.1 GO:0006486 GO:0016021 GO:0008378 GO:0000139

XM_018052395.1 GO:0006869 GO:0004872 GO:0030054 GO:0014069 GO:0043523 GO:0030426 GO:0005811 GO:0005637 GO:0016021 GO:0005789 GO:0005640 GO:0045211

XM_018060567.1 GO:0014850 GO:0005634 GO:0006355 GO:0005737

XM_005677583.3 GO:0070062 GO:0005634 GO:0005615 GO:0042127 GO:0042803 GO:0005737 GO:0001726 GO:0044548 GO:0005509 GO:0048306

XM_018050856.1 GO:0036289 GO:0005783 GO:0005524 GO:0005874 GO:0060502 GO:0060324 GO:0043539 GO:0010629 GO:0070371 GO:0060440 GO:0030878 GO:0097110 GO:2000147 GO:0005911 GO:0004702 GO:0009898 GO:0030165 GO:0005778 GO:0032947 GO:0000187 GO:0007507 GO:0048538 GO:0050772 GO:0048471 GO:0005794 GO:0004708 GO:0048679

XM_018058559.1 GO:0016197 GO:0005783 GO:0090306 GO:0001556 GO:0071203 GO:0005829 GO:0040038 GO:0005769 GO:0015031

XM_018053300.1 GO:0070062 GO:0001954 GO:0005654 GO:0031594 GO:0017166 GO:0005737 GO:0019901 GO:0003779 GO:0005178 GO:0008270 GO:0042383 GO:0031527 GO:0016010 GO:0070938

XR_001919233.1 GO:0006457 GO:0005783 GO:0043025 GO:0043005 GO:0033130 GO:0007271 GO:0016021 GO:0044183 GO:0043623 GO:0034394

XM_018044571.1 GO:0016020 GO:0048675 GO:0036459 GO:0030509 GO:0005737 GO:0007179 GO:0030426 GO:0006511 GO:0070410 GO:0001764 GO:0016579

XM_018044853.1 GO:0072686 GO:0005827 GO:0006513 GO:0005813 GO:0007094 GO:0005737 GO:0004842 GO:0051301 GO:0031463

XM_018051858.1 GO:0008375 GO:0005783 GO:0015012 GO:0016021 GO:0006024

XM_018054620.1 GO:0042060 GO:0035023 GO:0043547 GO:0032956 GO:0005089

XM_005693304.3 GO:0016020 GO:0070062 GO:0034719 GO:0032797 GO:0005730 GO:0005829 GO:0030532 GO:0006364 GO:0000387

XM_018061800.1 GO:0030330 GO:0004843 GO:0005654 GO:0006281 GO:0044822 GO:0002039 GO:0044325 GO:0006511 GO:0004197 GO:0016579 GO:0010506 GO:0005769

NM_001285673.1 GO:0043567 GO:0001077 GO:0005737 GO:0005667 GO:0003682 GO:0043565 GO:0030183 GO:0001102 GO:0005654 GO:0001105 GO:0008340 GO:0060133 GO:0045944 GO:0001708 GO:0040018 GO:0032962 GO:0008284

XM_005676359.3 GO:0005622

XM_005683428.3 GO:0006359 GO:0032728 GO:0045089 GO:0005666 GO:0000790 GO:0008283 GO:0001056 GO:0006384

XM_005683278.2 GO:0007186 GO:0050911 GO:0004984 GO:0016021 GO:0005886 GO:0004930

XM_018041462.1 GO:0008135 GO:0005634 GO:0003730 GO:0005737 GO:0071230 GO:0043005 GO:0000122 GO:0061158 GO:0030014 GO:0000166 GO:0060213

XM_018054848.1 GO:0016020

XM_018066211.1 GO:0070062 GO:0005615 GO:0048251 GO:0042803 GO:0071953 GO:2000121 GO:0008022 GO:0005178 GO:0005509 GO:0034394 GO:0046903

XM_005683151.3 GO:0005615 GO:0005125 GO:0007166 GO:1900017 GO:0005126

XM_018066249.1 GO:0005615 GO:0030335 GO:0001755 GO:0048843 GO:0050919 GO:0030215 GO:0045499 GO:0016021 GO:0038191 GO:0005886 GO:0045202 GO:0071526

XM_005685736.3 GO:0030331 GO:0005634 GO:0007368 GO:0036158 GO:0033146 GO:0005737 GO:0061136 GO:0003341 GO:0036159 GO:0005886

XM_005700492.3 GO:0006694 GO:0005783 GO:0008203 GO:0060716 GO:0055114 GO:0005811 GO:0016021 GO:0003854 GO:0007224 GO:0001942

XM_018067162.1 GO:0006357 GO:0003677 GO:0000981 GO:0005654 GO:0007275 GO:0007010 GO:0022604 GO:0005737

XM_018058264.1 GO:0051897 GO:0003725 GO:0000122 GO:0005730 GO:0016604 GO:0016021 GO:0031663 GO:0045766 GO:0044822 GO:0051092 GO:0043066 GO:0001085 GO:0010508 GO:0048471 GO:0043123 GO:0051059 GO:0003713 GO:0005789

XM_005684239.2 GO:0004674 GO:0005524 GO:0006468

XM_018065904.1 GO:0008565 GO:0005794 GO:0005905 GO:0015031

XM_018046617.1 GO:0050702 GO:0071404 GO:0016324 GO:0031623 GO:0070543 GO:0031526 GO:0007166 GO:1990000 GO:1900227 GO:0016021 GO:0070508 GO:0044539 GO:0050909 GO:0034197 GO:0071726 GO:0007155 GO:0045121 GO:0030299 GO:0005794 GO:0006955 GO:0007204 GO:0070542 GO:0070374 GO:2000505

XM_005674709.3 GO:0005923 GO:0016021 GO:0005198 GO:0005886

XM_018057555.1 GO:0030100 GO:0043547 GO:0045202 GO:0005096

XM_018054195.1 GO:0030424 GO:0050839 GO:0004872 GO:0005102 GO:0007157 GO:0042803 GO:0034332 GO:0005887 GO:0005913 GO:0007156 GO:0008037 GO:0045202

XM_018044979.1 GO:0005615 GO:0005622 GO:0035023 GO:0043547 GO:0005089

XM_018038990.1 GO:0044425

XM_005677488.3 GO:0000149 GO:0043393 GO:0031083 GO:0031629 GO:0008021 GO:0048471 GO:0006886 GO:0097352 GO:0010977 GO:0005730 GO:0048490 GO:0005794 GO:0008333 GO:0016188 GO:0072553

XM_013976675.2 GO:0045732 GO:0016567 GO:0005622 GO:0035556

XM_005688252.3 GO:0007193 GO:0043005 GO:0005887 GO:0005829 GO:0007268 GO:0007204 GO:0019233 GO:0007218 GO:0042923 GO:0038003 GO:0001626

XM_013976175.2 GO:0007256 GO:0007567 GO:0005524 GO:0004672 GO:0005622 GO:0060721 GO:0008285

XM_018041159.1 GO:0046872 GO:0008253 GO:0016311

XM_005686665.3 GO:0097192 GO:0005615 GO:0010575 GO:0051781 GO:0005149 GO:0035234 GO:0005829 GO:0005507 GO:0045766 GO:0045840 GO:0008285 GO:0005125 GO:0001660 GO:0034605 GO:0046688 GO:0045086 GO:0045944 GO:0006955 GO:0050715 GO:0019221 GO:0002248

XM_018055980.1 GO:0070588 GO:0000287 GO:0030425 GO:0005245 GO:0008427 GO:0030054 GO:0045921 GO:0019901 GO:0005829 GO:0031045 GO:0045211 GO:0030424 GO:0070062 GO:0050806 GO:0014069 GO:0048471 GO:0006469 GO:0005794 GO:0005509 GO:0048015 GO:0010975

XM_018063638.1 GO:0050700 GO:0051092 GO:0001934 GO:0043066 GO:0033209 GO:0005737

XM_018057033.1 GO:0005783 GO:0030054 GO:0030296 GO:0008154 GO:0030426 GO:0005829 GO:0001756 GO:0072673 GO:0008285 GO:0045211 GO:0031209 GO:0005856 GO:0070062 GO:0005634 GO:0035855 GO:0008092 GO:0061098 GO:0016032 GO:0030027 GO:0014069 GO:0030175 GO:0006928 GO:0038096 GO:0048010 GO:0032403

XM_018042402.1 GO:0016324 GO:0006970 GO:0034465 GO:0060083 GO:0043065 GO:0005901 GO:0030007 GO:0060072 GO:0042391 GO:0008076 GO:0070062 GO:0005249 GO:0045794 GO:0034765 GO:0001666 GO:0003779 GO:0046872 GO:0051592 GO:0071805

XR_001918084.1 GO:0016589

XM_018038279.1 GO:0008270 GO:0005737

XM_018053295.1 GO:0035914 GO:0046872 GO:0001228 GO:0005654 GO:0045944 GO:0000977 GO:0005794

XM_005696697.3 GO:0006979 GO:0004602 GO:0055114

XM_018039642.1 GO:0044257 GO:0019003 GO:0005764 GO:0055038 GO:0016239 GO:0007264 GO:0008333 GO:0005525 GO:0005776

XM_013974052.2 GO:0006355 GO:0003700

XM_013966004.2 GO:0045892 GO:0046872 GO:0005634 GO:0072562 GO:0006275 GO:0006974 GO:0042803 GO:0008327

XR_001919887.1 GO:0006820 GO:0016020 GO:0005216 GO:0034220

XM_005677878.3 GO:0005615 GO:0035279 GO:0044822 GO:0005524 GO:0004386 GO:0000932

XM_018059031.1 GO:0005739 GO:0047961

XM_013964572.2 GO:0005737

XM_005691552.3 GO:0070062 GO:0005788 GO:0016020 GO:0000187 GO:0050709 GO:0010629 GO:0010628 GO:0009986 GO:1902235

XM_018050159.1 GO:0050731 GO:0035718 GO:0005783 GO:0016064 GO:0009897 GO:0002606 GO:0004896 GO:0045060 GO:0030890 GO:0045581 GO:0050998 GO:0001516 GO:0002906 GO:0044183 GO:0070062 GO:0042613 GO:0048146 GO:0005764 GO:0042658 GO:0006886 GO:1902166 GO:0005771 GO:0006461 GO:0051085 GO:0001961 GO:0043518 GO:0016021 GO:0045582 GO:0060907 GO:0002792 GO:0001540 GO:0090023 GO:0035691 GO:0035692 GO:0035693 GO:0005794 GO:0002830 GO:0006952 GO:0019886 GO:0045059 GO:0070374 GO:2000343

XM_018051434.1 GO:0016920 GO:0006508 GO:0005829

NM_001285601.1 GO:0003148 GO:0060364 GO:0090427 GO:0030513 GO:0060444 GO:0070166 GO:0061312 GO:0035880 GO:0045617 GO:0008285 GO:0032792 GO:0035116 GO:0043066 GO:0045599 GO:0001227 GO:0000977 GO:0009952 GO:2000678 GO:0003198 GO:0002063 GO:0023019 GO:0000122 GO:0060346 GO:0003712 GO:0005634 GO:2001055 GO:0003416 GO:0045669 GO:0035115 GO:0048863 GO:0035313 GO:0008134 GO:0051795 GO:0071392 GO:0002076

XM_018053611.1 GO:0007093 GO:1990423

XM_018041454.1 GO:0000978 GO:0005634 GO:0042127 GO:0046982 GO:0001077 GO:0042803 GO:0045944 GO:0048856

XM_018066242.1 GO:0016310 GO:0005654 GO:0005524 GO:0033857 GO:0052724 GO:0003993 GO:0000827 GO:0006020 GO:0052723 GO:0000832 GO:0005829 GO:0016311 GO:0005886

XM_005689247.3 GO:0016323 GO:0015106 GO:0005768 GO:0015701 GO:0019531 GO:1902358 GO:0016021 GO:0005254 GO:0019532 GO:0008271 GO:0001696 GO:1902476

XM_018049244.1 GO:0070062 GO:0005737

XM_018052134.1 GO:0070062 GO:1903364 GO:0005634 GO:0002039 GO:0031398 GO:0005737 GO:0043518 GO:1902254 GO:0006606 GO:0016021

XM_018054800.1 GO:0070062 GO:0005925 GO:0005509

XM_018043965.1 GO:0032027 GO:0044458 GO:0007283 GO:0097224

XM_018057034.1 GO:0005783 GO:0030054 GO:0030296 GO:0008154 GO:0030426 GO:0005829 GO:0001756 GO:0072673 GO:0008285 GO:0045211 GO:0031209 GO:0005856 GO:0070062 GO:0005634 GO:0035855 GO:0008092 GO:0061098 GO:0016032 GO:0030027 GO:0014069 GO:0030175 GO:0006928 GO:0038096 GO:0048010 GO:0032403

XM_018067183.1 GO:0030308 GO:0043065 GO:0005634 GO:0045111 GO:0008440 GO:0046854

XM_005689709.2 GO:0016021

XM_018049443.1 GO:0016020 GO:0005768 GO:0005739

XM_013973975.2 GO:0005654 GO:0008022 GO:0005925 GO:0070975

XM_005700903.1 GO:0017017 GO:0005634 GO:0035335 GO:0004725 GO:0000188 GO:0005737

XM_018044793.1 GO:0017017 GO:0035335 GO:0004725 GO:0000188 GO:0005737

XM_018063101.1 GO:0070062 GO:0030676 GO:0043547 GO:0032587 GO:1900029 GO:0005737 GO:0042608 GO:0003779 GO:0043234 GO:0035023

XM_005701732.3 GO:0030133 GO:0016021

XM_018058529.1 GO:0005634 GO:0006355 GO:0043565 GO:0003700

XM_018050685.1 GO:0018105 GO:0004674 GO:0005524

XM_018041461.1 GO:0008135 GO:0005634 GO:0003730 GO:0005737 GO:0071230 GO:0043005 GO:0000122 GO:0061158 GO:0030014 GO:0000166 GO:0060213

XM_018057046.1 GO:0061630 GO:0005654 GO:0030891 GO:0031625 GO:0016032 GO:0007050 GO:0042787 GO:0031462 GO:0005730 GO:0005829 GO:0000082 GO:0061418 GO:0097193 GO:0008285 GO:0032403

XM_005700421.2 GO:0031989 GO:0016021 GO:0004946 GO:0005886

XR_001918069.1 GO:0010369 GO:0003677 GO:0005654 GO:0005730 GO:0003682

XM_018056727.1 GO:0001669

XM_005675420.2 GO:0043484 GO:0010494 GO:0046872 GO:0005654 GO:0044822 GO:0005813 GO:0003725

XM_018044144.1 GO:0000122 GO:0000978 GO:0005654 GO:0001078

XM_018044435.1 GO:0008081 GO:0006629

XM_005680826.3 GO:0043229 GO:0005829 GO:2001238 GO:0019901

XM_013967643.2 GO:0032755 GO:0005615 GO:0005125 GO:0006955 GO:0045582 GO:0006954 GO:0005149

XM_018053672.1 GO:0006397 GO:0002244 GO:0004652 GO:0005634 GO:0005524 GO:0043631 GO:0005737 GO:0046872 GO:0071044

XM_005679052.2 GO:0048027 GO:0000166 GO:0003730 GO:0005737

XM_018039463.1 GO:0003677 GO:0005634

XM_018066688.1 GO:0007623 GO:0000187 GO:0043066 GO:0007283 GO:0045987 GO:0045765 GO:0001664 GO:0005576 GO:0007218 GO:0006935 GO:0008284

XM_018056322.1 GO:0055085 GO:0005524 GO:0042626 GO:0016021

XM_018053135.1 GO:0005654 GO:0005758

XM_018055565.1 GO:0003677 GO:0045666 GO:0047485 GO:0000435 GO:2001038 GO:0046983 GO:0043065 GO:0043967 GO:0003682 GO:0004402 GO:0060713 GO:0030374 GO:0000790 GO:0016922 GO:0035257 GO:0002155 GO:0019899 GO:0008134

XM_018053968.1 GO:0016020 GO:0070062 GO:0005634 GO:0004149 GO:0043209 GO:0006099 GO:0045252 GO:0033512 GO:0005739

XM_018056870.1 GO:0005634 GO:0004579 GO:0008250 GO:0006487 GO:0016021

XM_018048457.1 GO:0032060 GO:0016021 GO:0008219 GO:0016049 GO:0005886

XM_013970207.2 GO:0008270 GO:0016021

XM_005696348.3 GO:0006412 GO:0005763 GO:0003735

XM_018049294.1 GO:0046872 GO:0005634 GO:0006508 GO:0070084 GO:0070527 GO:0070006 GO:0022626

XM_018043084.1 GO:0005615 GO:0016324 GO:0030054 GO:0005200 GO:0035264 GO:0016192 GO:0005543 GO:0043025 GO:0007416 GO:0003779 GO:0008091 GO:0030534 GO:0021692

XM_005678626.3 GO:0000978 GO:0005654 GO:0016602 GO:0045944 GO:0046982 GO:0001077 GO:0032993

XM_013975172.2 GO:0005794 GO:0044291 GO:0016021 GO:0005886

XR_001295907.2 GO:0006006 GO:0005759 GO:0070062 GO:0005654 GO:0034604 GO:0006099 GO:0045254 GO:0004739 GO:0006086

XM_018048244.1 GO:0030520 GO:0045892 GO:0010724 GO:0003729 GO:0005654 GO:0003714 GO:0000381 GO:0005737 GO:0048813 GO:0021942 GO:0000166 GO:0050885 GO:0008134

XM_018039742.1 GO:0006612 GO:0006501 GO:0010508 GO:0000422 GO:0005829 GO:0004197 GO:0051697 GO:0016485 GO:0044804 GO:0000045

XM_018050057.1 GO:0003697

XM_018059290.1 GO:0005739

XM_005676270.3 GO:0003729 GO:0005634 GO:0043565 GO:0005737

XM_018066419.1 GO:0016310 GO:0005524 GO:0032091 GO:0004111 GO:0043209 GO:0005743 GO:0043066

XM_018052641.1 GO:0046872 GO:0003676

XM_018052833.1 GO:0001558 GO:0005520 GO:0005576

XM_018063035.1 GO:0031397 GO:0030425 GO:0005524 GO:0043278 GO:0035556 GO:0005911 GO:0005829 GO:0048265 GO:0007635 GO:0008270 GO:1901799 GO:0005634 GO:0004712 GO:0060384 GO:0043524 GO:0004697 GO:0097060 GO:0048471 GO:0042752 GO:0032095 GO:0018105 GO:0032425 GO:0007268 GO:0050764

XM_018060508.1 GO:0043123 GO:0061630 GO:0000151 GO:0016567 GO:0008270 GO:0004871 GO:0005769

XM_018038437.1 GO:0070588 GO:0051924 GO:0005246 GO:0005891

XM_018044119.1 GO:0030833 GO:0017048 GO:0000187 GO:0005524 GO:0060996 GO:0005622 GO:0007409 GO:0004674 GO:0071407 GO:0004708

XM_005678499.3 GO:0016705 GO:0020037 GO:0005506 GO:0016021 GO:0004497 GO:0055114

XM_018051238.1 GO:0030425 GO:0032353 GO:0007214 GO:0008331 GO:0005737 GO:0043025 GO:0021750 GO:0030644 GO:0000096 GO:0006006 GO:0021679 GO:0043524 GO:0042133 GO:0007628 GO:0035249 GO:0021522 GO:0050885 GO:0005891 GO:0070588 GO:0048791 GO:0019226 GO:0014056 GO:0016049 GO:0007274 GO:0007416 GO:0021590 GO:0050770 GO:0043113 GO:0005634 GO:0050883 GO:0060024 GO:0034765 GO:0014051 GO:0048813 GO:0048266 GO:0021702 GO:0007204 GO:0051899 GO:0019905 GO:0017158

XM_005676409.3 GO:0021612 GO:0097491 GO:0050919 GO:0021649 GO:1902285 GO:0021828 GO:0097374 GO:0017154 GO:0061551 GO:0016021 GO:0061549 GO:0038084 GO:1903375 GO:0030424 GO:0036486 GO:0048846 GO:0001525 GO:0007507 GO:0097490 GO:0005021 GO:1901166 GO:0046872

XR_001918730.1 GO:0000122 GO:0000978 GO:0046872 GO:0005634 GO:0007165 GO:0007275 GO:0001078

XM_018054831.1 GO:0070062 GO:0042995 GO:0031346 GO:0048550 GO:0071914 GO:0009986 GO:0043087 GO:0001934 GO:0016021 GO:2001287 GO:0005886

XM_005689107.3 GO:0003707 GO:0005634 GO:0006355 GO:0043565 GO:0008270 GO:0043401 GO:0003700

XM_018064275.1 GO:0006457 GO:0003755 GO:0005654 GO:0044822 GO:0000413 GO:0005737

XM_005674982.3 GO:0016477 GO:0014043 GO:0050321 GO:0006349 GO:0021766 GO:0031625 GO:0030877 GO:0071109 GO:0032436 GO:0032092 GO:0043025 GO:0030426 GO:0032091 GO:0036016 GO:0001837 GO:2000738 GO:0035729 GO:0043066 GO:0001085 GO:0046777 GO:0048471 GO:0010800 GO:0031334 GO:1901030 GO:0070059 GO:0045944 GO:0033138 GO:0006983 GO:0031333 GO:0051534 GO:0097192 GO:0001954 GO:0005524 GO:0043547 GO:0000320 GO:0045773 GO:0005977 GO:0010977 GO:0005829 GO:0035372 GO:0005739 GO:0044027 GO:0005886 GO:0032886 GO:0046827 GO:0007623 GO:0005634 GO:0002039 GO:0007520 GO:0005813 GO:0044337 GO:0010614 GO:0043198 GO:0009887 GO:0004674 GO:0051059 GO:0045444 GO:0030529 GO:0034236 GO:0008013

XM_018050789.1 GO:0005634 GO:0045666 GO:0005938 GO:0006355 GO:0043234

XM_018065772.1 GO:0031290 GO:0003148 GO:0021524 GO:0001158 GO:0001077 GO:0050728 GO:0005737 GO:0090090 GO:0021520 GO:0032760 GO:0048936 GO:0021983 GO:0003203 GO:0003682 GO:0045766 GO:0003215 GO:0001102 GO:0032729 GO:0032731 GO:0031103 GO:0005654 GO:0000978 GO:0001755 GO:0001105 GO:0043524 GO:0060037 GO:0055010 GO:0060379 GO:0060913 GO:0032725 GO:0043425 GO:0048880 GO:0045944 GO:0032755 GO:0032024 GO:0030331 GO:0010575 GO:0032730 GO:0071385 GO:0000122 GO:0032735 GO:0008270 GO:0016021 GO:0003139 GO:0035066 GO:0071657 GO:0043388 GO:0033147 GO:0003266 GO:0060384 GO:0090074 GO:0016922 GO:0045665 GO:0031016 GO:0021559 GO:0060413 GO:0042517 GO:0008284

XM_005697608.3 GO:0051233 GO:0032436 GO:0005737 GO:0019901 GO:0051297 GO:0043393 GO:0045143 GO:0016321 GO:0043066 GO:0045736 GO:0070194 GO:0008017 GO:0010800 GO:0010997 GO:0018105 GO:0000922 GO:0005524 GO:1901673 GO:0007094 GO:0030496 GO:0000122 GO:0000281 GO:0005730 GO:0031572 GO:0031648 GO:0005876 GO:0045184 GO:0071168 GO:0000942 GO:0000785 GO:0005813 GO:0004674 GO:0000795 GO:0000086 GO:0001578

XM_013971511.2 GO:0032060 GO:0016021 GO:0008219 GO:0016049 GO:0005886

XM_018065404.1 GO:0008217 GO:0007200 GO:0005887 GO:0007218 GO:0042312 GO:0001604

XM_005698846.1 GO:0008542 GO:0004222 GO:0009986 GO:0007155 GO:0006508 GO:0016021 GO:0008270 GO:0030534

XM_013964253.2 GO:0006958 GO:0004252 GO:0070062 GO:0072562 GO:0006508 GO:0005509 GO:0042802 GO:0045087

XM_005681335.3 GO:0001937 GO:0016020 GO:0005125 GO:0050900 GO:0007267 GO:0000049 GO:0042803 GO:0005737 GO:0006954 GO:0009986 GO:0051020 GO:0017101

XM_018048537.1 GO:0016560 GO:0007006 GO:0021795 GO:0005052 GO:0006635 GO:0005829 GO:0005739 GO:0048468 GO:0016561 GO:0005778 GO:0000038 GO:0005782 GO:0021895 GO:0051262 GO:0001764 GO:0008022 GO:0040018 GO:0050905 GO:0007029

NM_001285656.1 GO:0030425 GO:0048714 GO:0032008 GO:0007050 GO:0019901 GO:0043025 GO:0000139 GO:0005681 GO:0007264 GO:0048168 GO:0005829 GO:0005765 GO:0070062 GO:0019003 GO:0003924 GO:0046872 GO:2000074 GO:0005789 GO:0005525 GO:0016236

XM_018066274.1 GO:0005929 GO:0007018 GO:0005524 GO:0003777 GO:0008017 GO:0016887 GO:0005871

XM_018041847.1 GO:0003707 GO:0031490 GO:0000122 GO:0004887 GO:0008016 GO:0008270 GO:0043565 GO:0070062 GO:0000790 GO:0030522 GO:0009887 GO:0070324 GO:0060509 GO:0045944 GO:0007605 GO:0007621 GO:0008050 GO:0043401 GO:0019899

XM_018062019.1 GO:0006810 GO:0005643 GO:0031965 GO:0034399 GO:0017056 GO:0002230 GO:0051292

XM_018062628.1 GO:0002028 GO:0005524 GO:0051823 GO:0014853 GO:0010657 GO:0008016 GO:0005829 GO:0006874 GO:0033017 GO:0005886 GO:0006998 GO:0031965 GO:0014722 GO:0031307 GO:0050790 GO:0010830 GO:0004674 GO:0018105 GO:0017020

XM_018039084.1 GO:0000166 GO:0019722 GO:0005622 GO:0016021

XM_018050847.1 GO:0008270 GO:0003676

XM_018043426.1 GO:0030324 GO:0005783 GO:0030154 GO:0042127 GO:0006695 GO:0001568 GO:0055114 GO:0035264 GO:0047598 GO:0009791 GO:0016021 GO:0005640

XM_005685407.2 GO:0003906 GO:0003677 GO:0046872 GO:0005634 GO:0006281 GO:0090305 GO:0005739

XM_013967263.2 GO:0031532 GO:0048013 GO:0043547 GO:0046875 GO:0048814 GO:0050770 GO:0051056 GO:0005096

XM_018039382.1 GO:0005654 GO:0005737

NM_001285659.1 GO:0042175 GO:0016324 GO:0008543 GO:0031489 GO:0015031 GO:0055037 GO:0005765 GO:0070062 GO:0030140 GO:0019003 GO:0090387 GO:0003924 GO:0048471 GO:0031901 GO:0032880 GO:0009790 GO:0005525 GO:0007589 GO:0005770 GO:0006895 GO:0005791 GO:0042742 GO:0000139 GO:0045176 GO:0045335 GO:0005795 GO:0005829 GO:0007264 GO:0030659 GO:0005802 GO:0061024 GO:0097208 GO:0072372 GO:0001948 GO:0032456

XM_005695354.3 GO:0051055 GO:0005615 GO:0010951 GO:0004867 GO:0046628 GO:0014068 GO:0045721 GO:0090181 GO:0090207 GO:0005886

XM_018052949.1 GO:0035257 GO:0045944 GO:0030374 GO:0005622

XM_005689593.3 GO:0006886 GO:0030126 GO:0005829 GO:0005198 GO:0005793 GO:0005886 GO:0016192 GO:0000139

XM_005676151.3 GO:0001939 GO:0000781 GO:0019827 GO:0001940 GO:2001034 GO:0005886

XM_018054045.1 GO:0051015 GO:0051764 GO:1990357 GO:0097433 GO:0051017 GO:0005923 GO:0016328 GO:0005925 GO:0045214 GO:0005915 GO:0030027 GO:0017166 GO:0042803 GO:0048741 GO:0030486 GO:0001725 GO:0005509 GO:0051393 GO:0042383

XM_018062251.1 GO:0045665 GO:0000122 GO:0046872 GO:0000978 GO:0044323 GO:0048387 GO:0001078

XM_018054496.1 GO:0000398 GO:0044822 GO:0016021 GO:0071013

XM_018039374.1 GO:0030148 GO:0016021 GO:0047560 GO:0005789 GO:0055114 GO:0006666

XM_018050024.1 GO:0070062 GO:0005524 GO:0042147 GO:0005829 GO:0007264 GO:0005525 GO:0017137 GO:0016887

XM_018043220.1 GO:0051015 GO:0005634 GO:0015630 GO:0003785 GO:0005737 GO:0006357 GO:0000977 GO:0015629

XM_018048665.1 GO:0005524 GO:0006468 GO:2000021 GO:0035556 GO:0003084 GO:0032414 GO:0004674 GO:0005829 GO:0019869 GO:0019902 GO:0090188 GO:0010923

XM_005698579.3 GO:0046872 GO:0006355 GO:0000977 GO:0003700

XM_018065385.1 GO:0005874 GO:0043547 GO:0007021 GO:0005912 GO:0070830 GO:0034333 GO:0048487 GO:0005829 GO:0010812 GO:0005923 GO:0016328 GO:0007023 GO:0005096 GO:0031115

XM_018059502.1 GO:0070062 GO:0005654 GO:0008420 GO:2000134 GO:0045665 GO:0006357 GO:0001933 GO:0006470

XM_005678022.3 GO:0005654 GO:0000166 GO:0005689 GO:0003676

XM_005683723.3 GO:0098655 GO:0050951 GO:0016048 GO:0005227 GO:0016021 GO:0051262

XM_018059787.1 GO:0070507 GO:0021799 GO:0021869 GO:0032956 GO:0090263 GO:0045665 GO:0043015 GO:0019904 GO:0005829 GO:0016021 GO:0007049

XM_018042424.1 GO:0016324 GO:0006970 GO:0034465 GO:0060083 GO:0043065 GO:0005901 GO:0030007 GO:0060072 GO:0042391 GO:0008076 GO:0070062 GO:0005249 GO:0045794 GO:0034765 GO:0001666 GO:0003779 GO:0046872 GO:0051592 GO:0071805

XM_018047018.1 GO:0005524 GO:0004683 GO:0005516 GO:0042803 GO:0046777

XM_005678349.3 GO:0006006 GO:0042407 GO:0002024 GO:0004222 GO:0006950 GO:0010637 GO:0097009 GO:0046872 GO:0016021 GO:0005743 GO:0006515 GO:0034982 GO:0006629

XM_018043224.1 GO:0002020 GO:0005654 GO:0031011

XM_013964566.2 GO:0016785 GO:0005634 GO:0097056 GO:0001514 GO:0000049 GO:0005737

XM_018058383.1 GO:0045453 GO:2001240 GO:0010468 GO:0008083 GO:0005615 GO:0005125 GO:0048873 GO:0030890 GO:0046622 GO:0045582 GO:0006955 GO:0005139 GO:0043086 GO:0002360

XM_018052689.1 GO:0030335 GO:0045666 GO:0051155 GO:0043621 GO:0032897 GO:0045087 GO:0000209 GO:0005863 GO:0061630 GO:0045862 GO:1903265 GO:0048147 GO:0031369 GO:0045787 GO:0003723 GO:0030307 GO:0042787 GO:1902230 GO:0007014 GO:0008270 GO:0061564 GO:0005634 GO:0009411 GO:0051092 GO:1902187 GO:0001894 GO:0046716 GO:0043123 GO:0043130 GO:0045444 GO:0045732 GO:0017022

XM_018039464.1 GO:0003677 GO:0005634

XM_018066949.1 GO:0070062 GO:0006730 GO:0009258 GO:0047105 GO:0055114 GO:0019145 GO:0009058 GO:0016742 GO:0016155 GO:0005739

XM_018053499.1 GO:0005634

XM_005678948.3 GO:0061630 GO:0031462 GO:2001214 GO:0031466 GO:0051865 GO:0031625 GO:0035556

XM_005687495.3 GO:0008593 GO:0007155 GO:0030198 GO:0009888 GO:0008201 GO:0005802 GO:0005578

XM_018050761.1 GO:0046425 GO:0006342 GO:0005654 GO:2000677 GO:0051726 GO:0008134 GO:0034729 GO:0031151 GO:0016568

XM_018053440.1 GO:0010468 GO:0004709 GO:0005524 GO:0060718 GO:0051973 GO:0001890 GO:0043507 GO:0005737 GO:0010225 GO:0019100 GO:1900745 GO:0032212 GO:0000186

XM_018056520.1 GO:0015631 GO:0005814 GO:0019904 GO:0051301 GO:0046785 GO:0046599 GO:0019901

XM_018054115.1 GO:0010608 GO:0042149 GO:0045335 GO:0035032 GO:0008333 GO:0001934 GO:0005776 GO:0005634 GO:0097629 GO:0010508 GO:0097632 GO:0043552 GO:0001933 GO:0044233 GO:0090207 GO:0005930 GO:0000045

XM_013971101.2 GO:0035023 GO:0043547 GO:0005089

XM_018039493.1 GO:0008643 GO:0045815 GO:0005794 GO:0016021 GO:0042946 GO:0005886 GO:0042947

XM_018042278.1 GO:0007264 GO:0005622 GO:0005525

XM_005680281.3 GO:0016020 GO:0007018 GO:0005524 GO:0003777 GO:0035253 GO:0008017 GO:0005737 GO:0043025 GO:0043005

XM_018060832.1 GO:0000226 GO:0033043 GO:0031175 GO:0005813 GO:0005516 GO:0051011 GO:0030507

XM_018047734.1 GO:0043154 GO:0071376 GO:0003707 GO:0045786 GO:0046982 GO:0061469 GO:0001077 GO:0005737 GO:0043065 GO:0005667 GO:0043565 GO:0008270 GO:0031965 GO:0004879 GO:0042803 GO:0030522 GO:0035914 GO:0045944 GO:0045444 GO:0043401

XR_001918935.1 GO:0005615 GO:0005125 GO:0007166 GO:0006954 GO:0005126

XM_018045925.1 GO:0019898 GO:0005154 GO:1903595 GO:0035091 GO:0016050 GO:0031901 GO:0005868 GO:0006897 GO:0031201 GO:1990460 GO:0032456 GO:1990459 GO:0015031 GO:0005886 GO:0005158

XM_018043863.1 GO:0007399 GO:0016323 GO:0005615 GO:0019903 GO:0031625 GO:0032281 GO:0005737 GO:0043025 GO:0030426 GO:0010923 GO:0019900 GO:0035255 GO:0005923 GO:0030165 GO:0045211 GO:0043113 GO:0097120 GO:0014069 GO:0004385 GO:0043198 GO:0008022 GO:0001736 GO:0007268 GO:0045197

XM_005702078.3 GO:0000287 GO:0005634 GO:0005524 GO:0001669 GO:0007286 GO:0035556 GO:0046777 GO:0005814 GO:0004674

XM_018065380.1 GO:0005654 GO:0007165 GO:0015914 GO:0008526 GO:0005737

XM_005677960.3 GO:0070062 GO:0005524 GO:0004828 GO:0097056 GO:0006434 GO:0005737

XM_018042396.1 GO:0016324 GO:0006970 GO:0034465 GO:0060083 GO:0043065 GO:0005901 GO:0030007 GO:0060072 GO:0042391 GO:0008076 GO:0070062 GO:0005249 GO:0045794 GO:0034765 GO:0001666 GO:0003779 GO:0046872 GO:0051592 GO:0071805

XM_018056888.1 GO:0010951 GO:0004869

XM_018039226.1 GO:0005654 GO:0005737 GO:0045893 GO:0005667 GO:0070345 GO:0003700 GO:0001047 GO:0008284

XM_018046564.1 GO:0005634 GO:0021796 GO:0006355 GO:0046982 GO:0048665 GO:0071542 GO:0042803 GO:0035914 GO:0046872 GO:0007548 GO:0000987 GO:0002052 GO:0003700

XM_018038464.1 GO:0016021

XM_018062183.1 GO:0000978 GO:0005654 GO:0005730 GO:0045944 GO:0001077 GO:0005737

XM_005697818.2 GO:0005615 GO:0005128 GO:0030295 GO:0042541 GO:0045893 GO:0007566 GO:0005179 GO:0000122 GO:1902219 GO:0010523 GO:0046579 GO:1902251 GO:0043249 GO:0071474 GO:0032147 GO:0045740 GO:0001666 GO:0009986 GO:0042523 GO:0018105 GO:2001258 GO:0008284

XM_018051296.1 GO:0016787 GO:0016021

XM_013974080.2 GO:0003677 GO:0005634 GO:0006334 GO:0046982 GO:0006352 GO:0000786

XM_018049970.1 GO:0032481 GO:0002218 GO:0031625 GO:0005741 GO:0005777 GO:0061507 GO:0032092 GO:0019901 GO:0035458 GO:0016021 GO:0005886 GO:0033160 GO:0071360 GO:0035438 GO:0042803 GO:0006915 GO:0048471 GO:0032608 GO:0045944 GO:0005794 GO:0005789 GO:0008134 GO:0002230 GO:0042993

XM_013963327.2 GO:0030514 GO:0005615 GO:0060393 GO:0048839 GO:0042118 GO:0002043 GO:0045446 GO:0070374 GO:0001657 GO:0010594

XM_018064710.1 GO:0016020 GO:0003677 GO:0072368 GO:0003714 GO:0051225 GO:0042826 GO:0016580 GO:0035257 GO:0072362 GO:0017053 GO:0046329 GO:0001102 GO:0005876

XM_013962903.2 GO:0005524 GO:0006970 GO:0090316 GO:0000302 GO:0005795 GO:0000122 GO:0005730 GO:0032465 GO:0006974 GO:0031122 GO:0090166 GO:2000777 GO:0002039 GO:0005813 GO:0006468 GO:0043066 GO:0009314 GO:0004674 GO:0000082

XR_001919544.1 GO:0001939 GO:0000781 GO:0019827 GO:0001940 GO:2001034 GO:0005886

XM_018065509.1 GO:0030552 GO:0071222 GO:0031625 GO:0051117 GO:0071320 GO:0010880 GO:0097110 GO:0045822 GO:0032743 GO:0032754 GO:0005829 GO:0008144 GO:0060314 GO:0086004 GO:1901898 GO:0032729 GO:0033137 GO:0030593 GO:0050852 GO:0071872 GO:0006939 GO:0005813 GO:0007568 GO:0035264 GO:0002027 GO:0030814 GO:0046872 GO:0044325 GO:0006198 GO:0004115 GO:0005891

XM_018053461.1 GO:0000287 GO:0005840 GO:0010659 GO:0005524 GO:0004711 GO:0035556 GO:0043065 GO:0045835 GO:0005819 GO:0008285 GO:0005654 GO:0031965 GO:0004712 GO:0002035 GO:0007507 GO:0001556 GO:0010628 GO:0070613 GO:0071322 GO:0018105 GO:0060047

XM_005677959.3 GO:0030308 GO:0000922 GO:0031116 GO:0045737 GO:0008017 GO:0045893 GO:0030496 GO:0007080 GO:0005829 GO:0051301 GO:0060236 GO:0001578 GO:0005876

XM_018050687.1 GO:0018105 GO:0004674 GO:0005524

XM_018055915.1 GO:0016021

XM_018058498.1 GO:0060252 GO:0000978 GO:0005634 GO:0010629 GO:0001077 GO:0035264 GO:0046872 GO:0045944 GO:0060736 GO:0022612

XM_013973556.2 GO:0031088 GO:0016529 GO:0042045 GO:0048016 GO:0009791 GO:0005730 GO:0050882 GO:0016021 GO:0005955 GO:0050849 GO:0019855 GO:0014069 GO:0035091 GO:0001666 GO:0005220 GO:0070059 GO:0032469 GO:0031094 GO:0005637 GO:0005789 GO:0051209

XM_018055566.1 GO:0070062 GO:0005509 GO:0006897 GO:0005813 GO:0035023 GO:0043547 GO:0005089

XM_018040486.1 GO:0016020 GO:0005654 GO:0016525

XM_005682153.3 GO:0016705 GO:0020037 GO:0005506 GO:0016021 GO:0004497 GO:0055114

XM_005675438.3 GO:0045028 GO:0035589 GO:0016021

XM_005683520.3 GO:2000679 GO:0010667 GO:0007512 GO:0043433 GO:0001967 GO:0001077 GO:0048935 GO:2000764 GO:0045668 GO:0003253 GO:0033613 GO:0001525 GO:0070888 GO:0000790 GO:0042475 GO:0071300 GO:0043392 GO:0000977 GO:0003219 GO:0048485 GO:0061325 GO:0046982 GO:0042733 GO:2000763 GO:0060536 GO:0071407 GO:0005667 GO:0060021 GO:0010463 GO:0003266 GO:0060982 GO:0048538 GO:0001701 GO:0003680 GO:0003278 GO:0042803 GO:0043586 GO:0001947 GO:0003357 GO:0061032 GO:0003713

XM_018049421.1 GO:0000287 GO:0005783 GO:0005654 GO:0005524 GO:0045332 GO:0016021 GO:0004012 GO:0005886

XM_005687861.3 GO:0007147 GO:0005654 GO:0005524 GO:0006468 GO:0005813 GO:0034048 GO:0007067 GO:0032154 GO:0004674 GO:0000086 GO:0051726

XM_018057569.1 GO:0007186 GO:0016525 GO:0016021 GO:0007166 GO:0007422 GO:0051965 GO:0004930

XM_018063579.1 GO:0005634 GO:0010717

XM_018056413.1 GO:0016020 GO:0007264 GO:0005622 GO:0043547 GO:0005085

XM_018053278.1 GO:0007186 GO:0016021 GO:0007166 GO:0004930

XM_005684191.3 GO:0050850 GO:0050731 GO:0032481 GO:0090237 GO:0019370 GO:0002092 GO:0010803 GO:0004716 GO:0090330 GO:0002554 GO:0019901 GO:0019815 GO:0005178 GO:0031234 GO:0045087 GO:0042101 GO:0070372 GO:0045579 GO:0002250 GO:0007257 GO:0018105 GO:0048514 GO:0050764 GO:0032928 GO:0071404 GO:0005524 GO:0038083 GO:0032009 GO:0046641 GO:0042742 GO:0007169 GO:0045401 GO:0045425 GO:0071226 GO:0007229 GO:0002281 GO:0043366 GO:0050853 GO:0005634 GO:0030593 GO:0004715 GO:0001945 GO:0043306 GO:0046638 GO:0051090 GO:0042991 GO:0045780 GO:0045588 GO:0004674 GO:0050715 GO:0043313 GO:0033630

XM_018055669.1 GO:0000122 GO:0043065 GO:0046872 GO:0000981 GO:0000083 GO:0044212 GO:0008285

XM_018050764.1 GO:0046425 GO:0006342 GO:0005654 GO:2000677 GO:0051726 GO:0008134 GO:0034729 GO:0031151 GO:0016568

XM_018049707.1 GO:0008023

XM_013962625.2 GO:0006886 GO:0005794 GO:0005829 GO:0031338 GO:0042147 GO:0017137 GO:0005096

XM_018049266.1 GO:0046873 GO:0031090 GO:0016021 GO:0006829 GO:0005886 GO:0070574

XM_018047218.1 GO:0031290 GO:0086080 GO:0030424 GO:0001525 GO:0019227 GO:0045162 GO:0007417 GO:0008104 GO:0016021 GO:0034113 GO:0030506 GO:0010975 GO:0045202 GO:0005886

XM_018058793.1 GO:0046872 GO:0006355 GO:0005622 GO:0003700 GO:0003676

XM_013975418.2 GO:0043022 GO:0016021

XM_018040929.1 GO:0005634 GO:2001014 GO:0000381 GO:0000166 GO:0050885 GO:0005737 GO:0003676

NM_001285570.1 GO:0010744 GO:2001240 GO:0008083 GO:0005615 GO:0071803 GO:0001892 GO:0005125 GO:0032747 GO:0010628 GO:0045740 GO:0042523 GO:0006955 GO:0005129 GO:0045918 GO:0043011 GO:0008284

XM_018047597.1 GO:0044822 GO:0008270

XM_005699277.3 GO:0016209 GO:0070062 GO:0045670 GO:0016021 GO:0005739 GO:0055114

XM_018064722.1 GO:0006506 GO:0000225 GO:0016021 GO:0005789

XM_018047277.1 GO:0050702 GO:0071404 GO:0016324 GO:0031623 GO:0070543 GO:0031526 GO:0007166 GO:1990000 GO:1900227 GO:0016021 GO:0070508 GO:0044539 GO:0050909 GO:0034197 GO:0071726 GO:0007155 GO:0045121 GO:0030299 GO:0005794 GO:0006955 GO:0007204 GO:0070542 GO:0070374 GO:2000505

XM_005682087.3 GO:0007507

XM_005681629.3 GO:0005783 GO:0005524 GO:0038085 GO:0035584 GO:0010595 GO:0050927 GO:0051770 GO:0005887 GO:0005178 GO:0001938 GO:0045766 GO:0038084 GO:0018108 GO:0051894 GO:0014068 GO:0008360 GO:2000352 GO:0046777 GO:0005021 GO:0005768 GO:0045121 GO:0005794 GO:0048010 GO:0070374

XM_018040957.1 GO:0005524 GO:0006355

XM_018055856.1 GO:0045920 GO:0005874 GO:0008344 GO:0046983 GO:0019901 GO:0051932 GO:1901998 GO:0042584 GO:0070062 GO:0001917 GO:0044822 GO:0072583 GO:0042802 GO:0043209 GO:0051262 GO:0003924 GO:0030117 GO:0008022 GO:0007605 GO:0005525 GO:0007032

XM_018047916.1 GO:0035252 GO:0016266

XM_018057743.1 GO:0001649 GO:0005634 GO:0010508 GO:0001894 GO:0045893 GO:0043130 GO:0006511 GO:0005829 GO:0005776 GO:0000045

XM_013971174.2 GO:0045807 GO:0010828 GO:0043065 GO:0055038 GO:0032588 GO:0001934 GO:0044091 GO:0005886 GO:0031115 GO:0090004 GO:0008017 GO:0018230 GO:0035594 GO:0031901 GO:0045121 GO:0045444 GO:0072321

XM_005675162.2 GO:0006457 GO:0016020 GO:0005788 GO:0005634 GO:0032781 GO:0051082 GO:0016556

XM_005675143.3 GO:0004252 GO:0005615 GO:0006508 GO:0001867 GO:0005509

XM_018040546.1 GO:0030276 GO:0097190 GO:0072583 GO:0030136 GO:0035091 GO:0003779 GO:0005794 GO:0048268 GO:0006919 GO:0048260

XM_018038210.1 GO:0005622 GO:0035023 GO:0043547 GO:0005089

XM_018041047.1 GO:0004896 GO:0016021 GO:0019221

XM_018043502.1 GO:0001771 GO:0042101 GO:0031226 GO:0042102 GO:0007157 GO:0001772 GO:0001530 GO:0005044 GO:0006898 GO:0016021 GO:0002438 GO:0031663 GO:0070891 GO:1900017

XM_005688483.3 GO:0006085 GO:0003987 GO:0005654 GO:0005737 GO:0008610

XM_018051295.1 GO:0016787 GO:0016021

XM_005687813.3 GO:0047485 GO:0030054 GO:0043025 GO:0030252 GO:0042734 GO:0048306 GO:0031629 GO:0008021 GO:0043209 GO:0030027 GO:0007409 GO:0030175 GO:0048471 GO:0005768 GO:0070032 GO:0045121 GO:0070033 GO:0005484 GO:0032024 GO:0048791 GO:0007616 GO:0070044 GO:0051963 GO:0060291 GO:0015629 GO:0001504 GO:0008076 GO:0016197 GO:0017075 GO:0044295 GO:0031083 GO:0005249 GO:0043195 GO:0005802 GO:0016082 GO:0007626 GO:0044325 GO:0019904 GO:0008306 GO:0030431 GO:0017022 GO:0016081 GO:0071805 GO:0010975 GO:0014047

XM_018060994.1 GO:2000785 GO:0005654 GO:0035335 GO:0019898 GO:0004725 GO:0042149 GO:0004722 GO:0052629 GO:0004438 GO:0005737 GO:0046872 GO:0046856

XM_005679305.3 GO:0010008 GO:0055114 GO:0055072 GO:0030173 GO:0005887 GO:0005829 GO:0006897 GO:0005769 GO:0097461 GO:0008823 GO:0030140 GO:0045055 GO:0052851 GO:0006893 GO:0046872 GO:0015677 GO:0009725 GO:0005215

XM_018062016.1 GO:0051015 GO:0034314 GO:0005524 GO:0005903 GO:0060076 GO:0051653 GO:0000139 GO:0046677 GO:0060271 GO:0007163 GO:0002102 GO:0005911 GO:0005829 GO:0016344 GO:0070062 GO:0005885 GO:0050775 GO:0051491 GO:0008356 GO:0005925 GO:0048013 GO:0005200 GO:0030027 GO:0033206 GO:0005884 GO:0010592 GO:0009743 GO:0030056 GO:0038096 GO:0043519

XM_005682686.3 GO:0055001 GO:0048641 GO:0001891 GO:0014841 GO:0043654 GO:0014719 GO:0016021 GO:0034109 GO:0051147 GO:0014816

XM_018054851.1 GO:0006357 GO:0005654 GO:0005730 GO:0044212

XM_018059316.1 GO:0016021

XM_018053003.1 GO:0001228 GO:0005634 GO:0007283 GO:0001162 GO:0042803 GO:0005737 GO:0000979 GO:0045944

XM_005693893.2 GO:0070062 GO:0016021

XM_018056070.1 GO:0015269 GO:0016021 GO:0071805

XM_018048130.1 GO:0045892 GO:0000978 GO:0005654 GO:0001525 GO:0003714 GO:0045944 GO:0042060 GO:0001077 GO:0005739 GO:0032422

XM_005684598.3 GO:0005634 GO:0030316 GO:0005829 GO:0016021

XM_018048633.1 GO:0009952 GO:0060434 GO:0007420 GO:0042733 GO:0008277 GO:1901621 GO:0005730 GO:0021953 GO:0005886 GO:0060348 GO:0048702 GO:0031076 GO:0035091 GO:0021914 GO:0061548 GO:0072372 GO:0001843 GO:0005930 GO:0030991 GO:0019899 GO:0032403 GO:0097546

XM_018061934.1 GO:0070507 GO:0004843 GO:0007346 GO:1902017 GO:0005881 GO:0019901 GO:0030496 GO:0090090 GO:0036064 GO:0045581 GO:0097542 GO:0006511 GO:0005829 GO:0031234 GO:0008270 GO:0005819 GO:2001238 GO:0032088 GO:0070064 GO:0070536 GO:1901026 GO:0005813 GO:2001242 GO:0048471 GO:0061578 GO:0042347

XM_018050202.1 GO:0043402 GO:0000978 GO:0005815 GO:0005654 GO:0001077 GO:0042921 GO:0045944 GO:0005819 GO:0008270 GO:0005739 GO:0016568 GO:0038051 GO:1990239

XM_018064721.1 GO:0006506 GO:0000225 GO:0016021 GO:0005789

XM_005685410.3 GO:0000408 GO:0046872 GO:0005634 GO:0002949 GO:0005737

XM_018038888.1 GO:2000535 GO:0016020 GO:0043231 GO:0001927 GO:0047485 GO:0006893 GO:0019901 GO:0000145 GO:0017160

XM_018062594.1 GO:0003688 GO:0005654 GO:0000784 GO:0005524 GO:0005664 GO:0006270 GO:0005737 GO:0005730 GO:0015629

XM_005677184.3 GO:0070062 GO:0007155 GO:0016021 GO:0046718

XM_005684350.3 GO:0005615 GO:0005125 GO:0007250 GO:0005622 GO:0005164 GO:0042107 GO:0006955 GO:0006919 GO:0005886

XM_018048861.1 GO:0070062 GO:0006695 GO:0005741 GO:0055114 GO:0004128 GO:0005811 GO:0005743 GO:0005789 GO:0071949

XM_018065465.1 GO:0003723 GO:0051321 GO:0000166

XM_005680859.1 GO:0016021

XM_013972369.2 GO:0007099 GO:0005813 GO:0005737

XM_018043992.1 GO:0045184 GO:0010738 GO:0030317 GO:0051018 GO:0005634 GO:0007178 GO:0044458 GO:0048471 GO:0030018 GO:0097228 GO:0035686

XM_005700911.3 GO:0043154 GO:0048027 GO:0008625 GO:0017148 GO:0043273 GO:0045087 GO:0070062 GO:2001243 GO:0043024 GO:0003924 GO:0004003 GO:0008190 GO:0004004 GO:0045944 GO:0016055 GO:0045070 GO:0010501 GO:0031333 GO:0010494 GO:0008143 GO:0003677 GO:0035613 GO:0005524 GO:0007059 GO:0071243 GO:0030307 GO:0045948 GO:0032508 GO:0042256 GO:0030308 GO:1900087 GO:0043280 GO:0034063 GO:0005634 GO:0071470 GO:0022627 GO:0009615 GO:0005852 GO:0008134

XM_005675526.3 GO:0045779 GO:2001205 GO:1900181 GO:0005923 GO:0016021 GO:0005198 GO:0048565 GO:0005886 GO:0071847

XM_013975550.2 GO:0005634 GO:0043161 GO:0016567

XM_005678744.3 GO:0002046 GO:0007165 GO:0001917 GO:0001750 GO:0051219

XM_005676555.3 GO:0070419 GO:0003677 GO:0005634 GO:0010212 GO:0030217 GO:0030183 GO:0006310 GO:0006303

XM_005699636.3 GO:0016021

XM_018059708.1 GO:0010494 GO:0043231 GO:0016020 GO:0005524 GO:0044822 GO:0006417 GO:0000932 GO:0045665 GO:0019827 GO:0019904 GO:0004004 GO:0019074 GO:0033962 GO:0010501 GO:0016442

XM_018065060.1 GO:0005198 GO:0005882

XM_018063977.1 GO:0016020 GO:0046872 GO:0044822

XM_018058004.1 GO:0070062 GO:0030425 GO:0005783 GO:0044822 GO:0044297 GO:0046726 GO:0036464 GO:0045070 GO:0005886

XM_018063968.1 GO:0005813

XM_018050231.1 GO:0044822 GO:0007156 GO:0005509 GO:0016021 GO:0005886

XM_005697305.3 GO:0070062 GO:0002009 GO:0005615 GO:0010951 GO:0004867 GO:0050678 GO:0005737 GO:0030198 GO:0060512

XM_018056555.1 GO:0005634 GO:0070628 GO:0030544 GO:0051087 GO:0005739 GO:0090084

XM_018052126.1 GO:0070062 GO:0031410 GO:0017137

XM_005678210.3 GO:0005654 GO:0044822 GO:0010628

XM_013974043.2 GO:0097502 GO:0006506 GO:0031501 GO:0004584 GO:0016021 GO:0005789

XM_018038495.1 GO:0016021 GO:0003841 GO:0005789 GO:0001819 GO:0016024 GO:0006654

XM_018059904.1 GO:0016567 GO:0031463 GO:0004842

XM_018039558.1 GO:1903025 GO:0046872 GO:0005634 GO:0045944 GO:0045600 GO:0003676

XM_018064781.1 GO:0070062 GO:0005524 GO:0003774 GO:0007626 GO:0042472 GO:0016459 GO:0007605 GO:0032420

XM_018064937.1 GO:0048857 GO:0021612 GO:0021570 GO:0009952 GO:0009953 GO:0005634 GO:0006355 GO:0005737 GO:0002011 GO:0048704 GO:0043565 GO:0003700 GO:0021569

XM_005675118.3 GO:0005615 GO:0045668 GO:0046325 GO:0003416 GO:0007166 GO:0005102 GO:0030828

XM_018043494.1 GO:0048295 GO:0070062 GO:0005125 GO:0043524 GO:0046982 GO:0005622 GO:0007166 GO:0005127 GO:0097058 GO:0030890 GO:0042517 GO:0048711 GO:0030183 GO:0097059

XM_018064864.1 GO:0070588 GO:0045956 GO:0034765 GO:0060371 GO:0002027 GO:0097110 GO:0008332 GO:0086010 GO:0070509 GO:0007268 GO:0010045 GO:0005891

XM_018060820.1 GO:0000978 GO:0005654 GO:0003714 GO:0045893 GO:0001078 GO:0010172 GO:0000122 GO:0048863 GO:0046872 GO:0001047

XM_018056406.1 GO:0016020 GO:0007264 GO:0005622 GO:0043547 GO:0005085

XM_018050662.1 GO:0001755 GO:0048843 GO:0050919 GO:0030215 GO:0051642 GO:0005887 GO:0045499 GO:0004888 GO:0071526 GO:2001224

XM_013970321.2 GO:0016814 GO:0008270

XM_018065036.1 GO:0005925 GO:0008104

XM_005685346.3 GO:0016337 GO:0070097 GO:0005911 GO:0005509 GO:0007156 GO:0016021 GO:0045294 GO:0005886 GO:0008013

XM_013973616.2 GO:0070062 GO:0005615 GO:0030335 GO:0001755 GO:0048843 GO:0050919 GO:0030215 GO:0045499 GO:0038191 GO:0071526

XR_001918094.1 GO:0009897 GO:0019976 GO:0016021 GO:0043066 GO:0038110 GO:0004911

XM_018048782.1 GO:0016814 GO:0008270

XM_018046463.1 GO:0007186 GO:0016021 GO:0004930

XM_013962890.2 GO:0070062 GO:0005765 GO:0016477 GO:0007166 GO:0045807 GO:0048471 GO:0005887 GO:0008283 GO:0050821

XM_018041368.1 GO:0046872 GO:0003676

XM_018053448.1 GO:0010667 GO:0042759 GO:0048714 GO:0005737 GO:0051028 GO:0017124 GO:0001570 GO:0006397 GO:0042552 GO:0005634 GO:0003729 GO:0007286 GO:0010976 GO:0010628 GO:0061158 GO:0042692 GO:0008380 GO:0048255

XM_005694740.3 GO:0008299 GO:0004421 GO:0005654 GO:0005886 GO:0005737

XR_309899.3 GO:0000470 GO:0000460 GO:0044822 GO:0005730 GO:0000027 GO:0042134 GO:0030687

XR_001919578.1 GO:0071376 GO:0003707 GO:0008344 GO:0046982 GO:0001077 GO:0035259 GO:0042053 GO:0042416 GO:0000122 GO:0009791 GO:0000979 GO:0008270 GO:0031668 GO:2001234 GO:0043085 GO:0051866 GO:0005654 GO:0043524 GO:0071542 GO:0021986 GO:0004879 GO:0001666 GO:0030522 GO:0001975 GO:0001764 GO:0034599 GO:0045444 GO:0045944 GO:0021952 GO:0043401 GO:0043576 GO:0042551

XM_018048533.1 GO:0016560 GO:0007006 GO:0021795 GO:0005052 GO:0006635 GO:0005829 GO:0005739 GO:0048468 GO:0016561 GO:0005778 GO:0000038 GO:0005782 GO:0021895 GO:0051262 GO:0001764 GO:0008022 GO:0040018 GO:0050905 GO:0007029

XM_018060338.1 GO:0070829 GO:0072341 GO:0004489 GO:0050660 GO:0055114 GO:0006555 GO:0031060 GO:0050667 GO:0035999 GO:0032403

XM_018044689.1 GO:0055085 GO:0005524 GO:0042626 GO:0016021

XM_018040483.1 GO:0016020 GO:0005654 GO:0016525

XM_018064448.1 GO:0007099 GO:0005813 GO:0005737

XM_018062007.1 GO:0060170 GO:0021766 GO:0001103 GO:0051216 GO:0010629 GO:0038108 GO:0005737 GO:0060296 GO:0036064 GO:0021987 GO:0031514 GO:0040015 GO:0042311 GO:0048854 GO:0034464 GO:0045494 GO:0043001 GO:0035058 GO:0033365 GO:0007288 GO:0045444 GO:0014824 GO:0040018 GO:0030534 GO:0021756

XM_013964591.2 GO:0070062 GO:0005654 GO:0001702 GO:0006065 GO:0003979 GO:0051287 GO:0055114 GO:0006024 GO:0005829 GO:0005975

XM_018064973.1 GO:0034446 GO:0061001 GO:0061098 GO:0005737 GO:0061099 GO:0019901 GO:0030334 GO:0015629

XM_018064986.1 GO:0005654 GO:0016308 GO:2000786 GO:0005776 GO:0046854

XM_005676289.3 GO:0006882 GO:1903615 GO:0050861 GO:0005385 GO:0071578 GO:0030890 GO:0005887 GO:0002903

XM_018047033.1 GO:0005654 GO:0045944 GO:0030374 GO:0008270 GO:0005739 GO:0043596

XM_018057639.1 GO:0016020 GO:0019905 GO:0042113 GO:0005737

XM_005678536.3 GO:0031419 GO:0009236 GO:0005739

XM_005699223.3 GO:0043297 GO:0030032 GO:0051015 GO:0002009 GO:0048675 GO:0031625 GO:0002162 GO:0030334 GO:0015629 GO:0045294 GO:0005886 GO:0090136 GO:0070062 GO:0005925 GO:0043034 GO:0005916 GO:0034333 GO:0045121 GO:0043234 GO:0005198 GO:0070527 GO:0034394

XM_018058513.1 GO:0070062 GO:0005765 GO:0015991 GO:0006897 GO:0000221 GO:0046961

XM_018050759.1 GO:0046425 GO:0006342 GO:0005654 GO:2000677 GO:0051726 GO:0008134 GO:0034729 GO:0031151 GO:0016568

XM_018056113.1 GO:0006627 GO:0005759 GO:0005615 GO:0004222 GO:0008270 GO:0005743

XM_018062280.1 GO:0070062 GO:0006508 GO:0004177 GO:0030145

XM_018063752.1 GO:0000287 GO:0004749 GO:0009165

XM_005689915.1 GO:0007186 GO:0050911 GO:0004984 GO:0016021 GO:0005886 GO:0004930

XM_018049989.1 GO:0044763 GO:0044421 GO:0043227 GO:0005737 GO:0065007

XM_018040923.1 GO:0070062 GO:0022820 GO:0016021 GO:0071805 GO:0015379 GO:1902476

XM_018061918.1 GO:0006997 GO:0045014 GO:1901673 GO:0016192 GO:0007076 GO:0000920 GO:0007080 GO:0005769 GO:0070062 GO:0005815 GO:0016458 GO:0016363 GO:0010824 GO:0042803 GO:0007034 GO:0019904 GO:0000794 GO:0000815

XM_005684072.3 GO:0005739 GO:0008796

XR_001295453.2 GO:0052852 GO:0070062 GO:0019395 GO:0005102 GO:0005777 GO:0010181 GO:0052854 GO:0005739 GO:0052853

XM_018043857.1 GO:0005524 GO:2000021 GO:0005737 GO:0019869 GO:0005923 GO:0005634 GO:0090004 GO:0043066 GO:0005912 GO:2000651 GO:0046777 GO:0010800 GO:0004674 GO:0090188 GO:2000688 GO:0051928

XM_018057456.1 GO:0016021

XM_013974718.2 GO:0031386 GO:0016925 GO:0005634 GO:0045944 GO:0001816

NM_001287564.1 GO:0008083 GO:0045666 GO:0030335 GO:0035054 GO:0070724 GO:0032092 GO:0070700 GO:0090090 GO:0043569 GO:0008285 GO:0042482 GO:2000065 GO:0060804 GO:1901522 GO:0002062 GO:0007219 GO:0033690 GO:0003130 GO:0035630 GO:0055114 GO:0055008 GO:0021978 GO:0000122 GO:0010862 GO:0004745 GO:0001938 GO:0003181 GO:0003308 GO:0072138 GO:0001701 GO:0006029 GO:0045669 GO:0006954 GO:0060395 GO:0048711 GO:0046332 GO:0060039 GO:0039706 GO:0060129 GO:0005615 GO:0060317 GO:0045786 GO:0005160 GO:0051042 GO:0043065 GO:0001658 GO:0003203 GO:0010922 GO:0005125 GO:0045600 GO:0001666 GO:0032348 GO:0042487 GO:0060128 GO:0019211 GO:0046982 GO:0071407 GO:0048839 GO:0031648 GO:0045165 GO:0061036 GO:0000187 GO:0010718 GO:0009986 GO:0030501 GO:1900745 GO:2000726 GO:0040007 GO:0070374

XM_005688190.3 GO:0016197 GO:0005634 GO:0030119 GO:0005770 GO:0000724 GO:0005764

XM_018050650.1 GO:0061630 GO:0005654 GO:0043161 GO:0006355 GO:0016567 GO:0008270 GO:0005737

XM_018041306.1 GO:0044822 GO:0000166

XM_005679206.3 GO:0000287 GO:0005783 GO:0008253 GO:0000166 GO:0016311 GO:0005739 GO:0009117

XM_018044262.1 GO:0005654

XM_018064276.1 GO:0016020 GO:0005783 GO:0005524 GO:0008022 GO:0004672 GO:0043234 GO:0043410 GO:0005078

XM_018056273.1 GO:0002522 GO:0006486 GO:0002361 GO:0008417 GO:0032580 GO:0036065 GO:0016021

XM_005699201.3 GO:0070062

XM_018042219.1 GO:0005654 GO:0007569 GO:0048146 GO:0001701 GO:0007296 GO:0003007 GO:0048844 GO:0045944 GO:0008270 GO:0048589 GO:0001570

XM_018063952.1 GO:0005813

XM_018044641.1 GO:0050731 GO:1902068 GO:0001568 GO:0042577 GO:0051091 GO:0005178 GO:0044329 GO:0016021 GO:0016311 GO:0034109 GO:0060020 GO:0005886 GO:0070062 GO:0044328 GO:0001702 GO:0042392 GO:0030111 GO:0001933 GO:0006644 GO:0005794 GO:0044330 GO:0050821

XM_005696976.2 GO:0071578 GO:0031258 GO:0005783 GO:0009986 GO:0005887 GO:0006882 GO:0005385

XM_018040517.1 GO:2000984 GO:0006119 GO:0006754 GO:0005739

XM_013971768.2 GO:0006605 GO:0070062 GO:0005654 GO:0008286 GO:0045111 GO:0010838 GO:0005737 GO:0015758 GO:0061178 GO:1902808 GO:0019905 GO:0006974 GO:0050821

XM_013975432.2 GO:0000784 GO:0051973 GO:0003950 GO:0032212 GO:0005829 GO:0008270 GO:0000209 GO:0070198 GO:0051225 GO:0070212 GO:0090263 GO:0018107 GO:0018105 GO:0005794 GO:0070213 GO:0045944

XM_018041842.1 GO:0070062 GO:0005634 GO:0044822 GO:0006412 GO:0022625 GO:0003735

XR_001918040.1 GO:0033617 GO:0016021 GO:0031966

XM_005682752.3 GO:1990050 GO:0070234 GO:0005654 GO:0010950 GO:0005758 GO:0045580 GO:0090201 GO:0097035 GO:0010917 GO:0043234 GO:1901857 GO:2001140

XM_018044905.1 GO:0048050 GO:0048048 GO:0001527

XR_001918032.1 GO:0030424 GO:0004709 GO:0005524 GO:2000672 GO:0042803 GO:0007254 GO:0046777 GO:0019901 GO:0018107 GO:0030426 GO:0018105 GO:0005829 GO:0016572 GO:0005886 GO:0000186

XM_018039575.1 GO:0046872 GO:0005634 GO:0019904 GO:0048663 GO:0003676

XM_005696339.3 GO:0005654 GO:0071494 GO:0005737 GO:0003887 GO:0003684 GO:0000731 GO:0006301 GO:0006290

XM_018059356.1 GO:0016702 GO:0005788 GO:0019511 GO:0004656 GO:0005506 GO:0055114 GO:0031418

XM_013974525.2 GO:0046872 GO:0005634 GO:0006974 GO:0003676

XM_018047105.1 GO:0007275

XM_005676653.3 GO:0055085 GO:0008028 GO:0016021 GO:0015718

XM_018046826.1 GO:0061630 GO:0006513 GO:0031146 GO:0031625 GO:0016032 GO:0042787 GO:1990452 GO:0006915 GO:0019005 GO:0009887 GO:0008283

XM_013963479.2 GO:0007186 GO:0050911 GO:0004984 GO:0016021 GO:0005886 GO:0004930

XM_018056698.1 GO:0007595 GO:0016323 GO:0006878 GO:0005524 GO:0005770 GO:0016023 GO:0032588 GO:0005507 GO:0016021 GO:0004008 GO:0015680 GO:0051208 GO:0006882 GO:0046688 GO:0048471 GO:0060003 GO:0015677

XM_005685763.3 GO:0000165 GO:0005634 GO:0005524 GO:0046982 GO:0004707 GO:0005737 GO:0019901

XM_018042948.1 GO:0004817 GO:0006423 GO:0005524 GO:0005829 GO:0000049 GO:0042803

XR_001918144.1 GO:0006970 GO:0006355 GO:0003700

XM_018062593.1 GO:0008088 GO:0031514 GO:0003777 GO:0019894 GO:0035253 GO:0008017 GO:0005737 GO:0005871 GO:0043005

XM_005677470.3 GO:0035767 GO:0030866 GO:0006904 GO:0090002 GO:0055038 GO:0007264 GO:1902463 GO:0031175 GO:0005923 GO:0016328 GO:0044795 GO:0030659 GO:0097368 GO:0010737 GO:0005802 GO:0030027 GO:0043005 GO:0032869 GO:0032593 GO:0009306 GO:0030139 GO:0070830 GO:0005525 GO:0032456

XM_018043988.1 GO:0005524 GO:0004715 GO:0005622 GO:0035556 GO:0018108

XM_018061438.1 GO:0000812 GO:0035267 GO:0043967 GO:0005524 GO:0043968 GO:0016607

XM_018040789.1 GO:0031012 GO:0004222 GO:0060022 GO:0006954 GO:0006508 GO:0005509 GO:0016021 GO:0008270 GO:0005886

XM_013972518.2 GO:0001649 GO:0000981 GO:0001851 GO:0042177 GO:0071354 GO:0060766 GO:0005887 GO:0005769 GO:0008285 GO:0070062 GO:0005654 GO:0050847 GO:0042826 GO:0043209 GO:0007005 GO:0070373 GO:0071897 GO:0008022 GO:0005743 GO:0016575 GO:2000323 GO:0010944 GO:0045893 GO:0000122 GO:0030308 GO:0044212 GO:0001850 GO:0045745 GO:0009986 GO:0045917 GO:0070374 GO:0031871 GO:0042981 GO:0050821

XR_001919088.1 GO:0016477 GO:0001077 GO:0014904 GO:0030890 GO:0000122 GO:0003682 GO:0005829 GO:0044798 GO:0015629 GO:0033173 GO:1901741 GO:0006974 GO:0005886 GO:0050853 GO:0042493 GO:0005654 GO:0000978 GO:0001816 GO:0001078 GO:0045944 GO:0030529 GO:0008134

XM_018067188.1 GO:0016020 GO:0046872 GO:0043161 GO:0031464 GO:0005730 GO:0016567 GO:0004176 GO:0005737

XM_005677369.3 GO:0003723 GO:0005634

XM_018048086.1 GO:0097191 GO:0070738 GO:0070740 GO:0005128 GO:0051896 GO:0018169 GO:0045637 GO:0070737 GO:0051091 GO:0005829 GO:0051865 GO:0008270 GO:0017160 GO:0010498 GO:0045619 GO:0008285 GO:0000209 GO:0005135 GO:0061630 GO:2000379 GO:0038128 GO:0070736 GO:0043408 GO:0030336 GO:0008766 GO:0031386 GO:2000114 GO:1901525 GO:0043774 GO:0043773

XM_005675077.3 GO:0000339 GO:0000166 GO:0045292 GO:0005846

XM_018038829.1 GO:0016021

XM_018039757.1 GO:0031430 GO:0006936 GO:0002230 GO:0042803

XM_005689869.3 GO:0070588 GO:0005634 GO:0051480 GO:0005887 GO:0003684 GO:0015279 GO:0007338 GO:0000012 GO:0006828

XM_018044300.1 GO:0000910 GO:0016567 GO:0031463 GO:0004842

XM_018055487.1 GO:0019433 GO:0000784 GO:0005981 GO:0019901 GO:0017018 GO:0005730 GO:0072357 GO:0005829 GO:0006470 GO:0000164 GO:0070688 GO:0032922 GO:0030155 GO:0043153 GO:0070062 GO:0005925 GO:0042587 GO:0046872 GO:0005979 GO:0051301 GO:0050115 GO:0000086

XM_018043425.1 GO:0030324 GO:0005783 GO:0030154 GO:0042127 GO:0006695 GO:0001568 GO:0055114 GO:0035264 GO:0047598 GO:0009791 GO:0016021 GO:0005640

XM_005690715.3 GO:0003222 GO:0005634 GO:0005524 GO:0009411 GO:0006513 GO:0034976 GO:0005737 GO:0008626 GO:0034450 GO:0043161 GO:0000151 GO:0031175 GO:0051865 GO:0019899 GO:0000209

XM_018060562.1 GO:0005605 GO:0070062 GO:0043113 GO:0007213 GO:0043236 GO:0005737 GO:0005509 GO:0005886

XM_005701147.2 GO:0034198 GO:0005765 GO:0032008 GO:0071230 GO:0003924 GO:0005794 GO:0034613 GO:0005525

XM_018062710.1 GO:0003376 GO:0005765 GO:0016310 GO:0046512 GO:0017050 GO:0005829 GO:0001568 GO:0007420 GO:0038036 GO:0008284

XM_018040479.1 GO:0005623 GO:0045454

XM_005681621.3 GO:0044822 GO:0005847

XM_018039238.1 GO:0048488 GO:0045806 GO:0008092 GO:0072657 GO:0043209 GO:0030054 GO:0030137 GO:0032587 GO:0005543 GO:0005768 GO:0007015 GO:0048812 GO:0005829 GO:0043679 GO:0097320 GO:0045202 GO:0030659

XM_018052687.1 GO:0030335 GO:0045666 GO:0051155 GO:0043621 GO:0032897 GO:0045087 GO:0000209 GO:0005863 GO:0061630 GO:0045862 GO:1903265 GO:0048147 GO:0031369 GO:0045787 GO:0003723 GO:0030307 GO:0042787 GO:1902230 GO:0007014 GO:0008270 GO:0061564 GO:0005634 GO:0009411 GO:0051092 GO:1902187 GO:0001894 GO:0046716 GO:0043123 GO:0043130 GO:0045444 GO:0045732 GO:0017022

XM_005675285.3 GO:0007399 GO:0035267 GO:0000978 GO:0043968 GO:0031011 GO:1903146 GO:0071564 GO:0003407 GO:0043967 GO:0043044 GO:0016514 GO:0000980 GO:0005886 GO:0031492

XM_005684663.3 GO:0005634 GO:0003713 GO:0045944 GO:0008270

XM_005690119.3 GO:0008270 GO:0005622

XM_005680673.3 GO:0070062 GO:0005615 GO:0035928 GO:0004792 GO:0005743 GO:0008097 GO:0030855

XM_005694519.3 GO:0015459 GO:0005509 GO:0031234 GO:0005737 GO:1901379 GO:0008076

XM_005685208.2 GO:0005783 GO:0005743

XM_018041994.1 GO:0005634 GO:0005545 GO:0030165 GO:0005886 GO:0005737

XM_005693259.3 GO:0005524 GO:0004672 GO:0006468

XM_018058262.1 GO:0051897 GO:0003725 GO:0000122 GO:0005730 GO:0016604 GO:0016021 GO:0031663 GO:0045766 GO:0044822 GO:0051092 GO:0043066 GO:0001085 GO:0010508 GO:0048471 GO:0043123 GO:0051059 GO:0003713 GO:0005789

XM_018047013.1 GO:0003677 GO:0003887 GO:0071897 GO:0006260

XM_005676017.3 GO:0003677 GO:0031965 GO:0016021 GO:0050291 GO:0005789 GO:0046513

XM_018056000.1 GO:0044822

XM_005695021.3 GO:0016021

XM_018052042.1 GO:0005634 GO:1900025 GO:0030336 GO:0035413 GO:0032587 GO:0005737 GO:0030177 GO:0090303 GO:0046627 GO:2000114 GO:2000393 GO:0030837 GO:0010977 GO:0035024 GO:1900028 GO:0008013

XM_018040428.1 GO:0097233 GO:0055085 GO:0005615 GO:0005524 GO:0006869 GO:0042626 GO:0016021 GO:0005886

XM_013967872.2 GO:0006412 GO:0005739 GO:0032790

XM_018046014.1 GO:0006406 GO:0030425 GO:0003729 GO:0000381 GO:0000184 GO:0016607 GO:0006369 GO:0031124 GO:0006417 GO:0043025 GO:0071013 GO:0005515 GO:0005829 GO:0000166 GO:0035145

XM_018044056.1 GO:0060348 GO:0005634 GO:0060612 GO:0005546 GO:0090263 GO:0090090 GO:0072161 GO:0005886 GO:0008013

XM_018056034.1 GO:0033120 GO:0006376 GO:0043491 GO:0005737 GO:0006302 GO:0030426 GO:0008543 GO:0001147 GO:0070301 GO:0007623 GO:0030424 GO:2000806 GO:0005654 GO:0000165 GO:0042802 GO:2000144 GO:0043066 GO:0010976 GO:0000228 GO:0071300

XM_018045362.1 GO:0046872 GO:0006355 GO:0005622 GO:0003676

XM_018044882.1 GO:0005856

XM_018044337.1 GO:0016021

XM_018044577.1 GO:0010839 GO:0016323 GO:0005524 GO:0042043 GO:0031982 GO:0090280 GO:0005730 GO:0004672 GO:0005911 GO:0005829 GO:0016021 GO:0061045 GO:0045202 GO:0005604 GO:0005652 GO:0005925 GO:0006468 GO:0016363 GO:0090288 GO:0045944 GO:0001953

XM_005691159.3 GO:1900087 GO:0080008 GO:0031965 GO:0000398 GO:0000974 GO:0016607 GO:0071013 GO:0005730 GO:0034504 GO:0071011 GO:0005662

XM_018048532.1 GO:0016560 GO:0007006 GO:0021795 GO:0005052 GO:0006635 GO:0005829 GO:0005739 GO:0048468 GO:0016561 GO:0005778 GO:0000038 GO:0005782 GO:0021895 GO:0051262 GO:0001764 GO:0008022 GO:0040018 GO:0050905 GO:0007029

XM_018044573.1 GO:0010839 GO:0016323 GO:0005524 GO:0042043 GO:0031982 GO:0090280 GO:0005730 GO:0004672 GO:0005911 GO:0005829 GO:0016021 GO:0061045 GO:0045202 GO:0005604 GO:0005652 GO:0005925 GO:0006468 GO:0016363 GO:0090288 GO:0045944 GO:0001953

XM_018064517.1 GO:0055085 GO:0016021 GO:0005789 GO:0006629

XM_005697268.2 GO:0045666 GO:0003714 GO:0046982 GO:0001093 GO:0043621 GO:0001077 GO:0000122 GO:0003682 GO:0005667 GO:0016021 GO:1900746 GO:0065004 GO:0005634 GO:0000978 GO:0070888 GO:0016525 GO:0001011 GO:0045944 GO:0001087 GO:0042118 GO:0006367

XM_013974773.2 GO:0009083 GO:0005524 GO:0004674 GO:0006468 GO:0047323 GO:0005947

XM_018039756.1 GO:0031430 GO:0006936 GO:0002230 GO:0042803

XM_018039079.1 GO:0008542 GO:0043113 GO:0046580 GO:0016358 GO:0043524 GO:0043547 GO:0043408 GO:0005737 GO:0007389 GO:0043198 GO:0050771 GO:0048169 GO:0031235 GO:0005096

XM_018066545.1 GO:0005856 GO:0016021

XM_018039468.1 GO:0070062 GO:0005654 GO:0005770 GO:0006869 GO:0015485

XM_005687493.3 GO:0008593 GO:0007155 GO:0030198 GO:0009888 GO:0008201 GO:0005802 GO:0005578

XM_018058046.1 GO:0046872 GO:0003676

XM_018065616.1 GO:0050731 GO:0043022 GO:0031532 GO:0010468 GO:0051896 GO:0033135 GO:0032008 GO:0019901 GO:0050727 GO:2000114 GO:0043087 GO:0031932 GO:0009790 GO:0030838

XR_310548.3 GO:0006627 GO:0006801 GO:0004252 GO:0042720 GO:0030728 GO:0007420 GO:0007283 GO:0001541 GO:0006465 GO:0008015 GO:0022904 GO:0016021 GO:0061300 GO:0033108 GO:0006974

XM_013971020.2 GO:0031396 GO:0030970 GO:0032092 GO:0044325 GO:1990037 GO:0032469 GO:0034704 GO:0005789 GO:0030968 GO:1903071 GO:1902236

XM_013975160.2 GO:0003824

XM_018062725.1 GO:0003376 GO:0005765 GO:0016310 GO:0046512 GO:0017050 GO:0005829 GO:0001568 GO:0007420 GO:0038036 GO:0008284

XM_005686253.3 GO:0016226 GO:0007059 GO:0071817 GO:0097361

XM_005675497.3 GO:0045892 GO:0046872 GO:0005634 GO:0072562 GO:0006275 GO:0006974 GO:0042803 GO:0008327

XM_018067046.1 GO:0000166 GO:0045727 GO:0003730

XM_018060437.1 GO:0003677 GO:0005634 GO:0006355 GO:0046983

XM_005693675.3 GO:0045121 GO:0005509 GO:0005911 GO:0042383 GO:0016021 GO:0016012

XM_005685043.3 GO:0042412 GO:0000097 GO:0005506 GO:0017172 GO:0005829 GO:0019451 GO:0055114

XM_005695806.3 GO:0006936 GO:0016021

XM_018059070.1 GO:0055085 GO:0006955 GO:0016021

XM_018038564.1 GO:0046983

XM_018041291.1 GO:0055070 GO:0005730 GO:0005507 GO:0051262 GO:0005737

XM_018066811.1 GO:0005615 GO:0030335 GO:0001755 GO:0048843 GO:0050919 GO:0030215 GO:0045499 GO:0038191 GO:0071526

XM_018039073.1 GO:0046872 GO:0003676

XM_018047799.1 GO:0016020 GO:0044822 GO:0000166 GO:0007010 GO:0022604

XM_018058393.1 GO:0004364 GO:0006749 GO:0006626 GO:0008053 GO:0005634 GO:0031307 GO:0032526 GO:0000266

NM_001287560.1 GO:0070062 GO:0071782 GO:0001654 GO:0019003 GO:0034389 GO:0007420 GO:0071786 GO:0007264 GO:0005525 GO:0015031 GO:0005886

XM_013963599.2 GO:2001199 GO:0016021

XM_005694161.3 GO:2000751 GO:0005634 GO:0005524 GO:0005813 GO:0035556 GO:0005694 GO:0005737 GO:0005819 GO:0072354 GO:0007064 GO:0090231 GO:0016568

XM_005689638.3 GO:0043559 GO:0035774 GO:0016021

XM_018043503.1 GO:0001771 GO:0042101 GO:0031226 GO:0042102 GO:0007157 GO:0001772 GO:0001530 GO:0005044 GO:0006898 GO:0016021 GO:0002438 GO:0031663 GO:0070891 GO:1900017

XM_018066145.1 GO:0002020 GO:0070062 GO:0003677 GO:0042645 GO:0006287 GO:0005760 GO:0003887 GO:0008408 GO:0006264 GO:0071897 GO:0003682 GO:0090305

XM_018066740.1 GO:0046872 GO:0008253 GO:0016311

XM_005684237.3 GO:0007193 GO:0003376 GO:0038036 GO:0006954 GO:0032651 GO:1903141 GO:0005178 GO:0016021 GO:0007219 GO:0005886

XM_005679399.1 GO:0008270 GO:0016021

XM_018046732.1 GO:0055085 GO:0005524 GO:0005730 GO:0042626 GO:0016021 GO:0005739

XM_018062351.1 GO:0051015 GO:0070830 GO:0005509 GO:1902396

XM_018048397.1 GO:0071560 GO:2000741 GO:0055059 GO:0032332

XM_018065459.1 GO:0060029 GO:0005615 GO:0061053 GO:0017147 GO:0090103 GO:0004930 GO:0090090 GO:0070367 GO:0043065 GO:0016021 GO:0042813 GO:0008285 GO:0007186 GO:0030308 GO:0035567 GO:0014033 GO:0045600 GO:0061037 GO:0010721

NM_001319278.1 GO:0032481 GO:0002218 GO:0031625 GO:0005741 GO:0005777 GO:0061507 GO:0032092 GO:0019901 GO:0035458 GO:0016021 GO:0005886 GO:0033160 GO:0071360 GO:0035438 GO:0042803 GO:0006915 GO:0048471 GO:0032608 GO:0045944 GO:0005794 GO:0005789 GO:0008134 GO:0002230 GO:0042993

XM_018053907.1 GO:0035176 GO:0007612 GO:0051965 GO:0007269 GO:0043234 GO:0030534 GO:0005246 GO:0071625 GO:0090129

XM_013966816.2 GO:0005615 GO:0045202

XM_018046886.1 GO:0046872

XM_018063471.1 GO:0006357 GO:0046872 GO:0000981 GO:0005634 GO:0003676

XM_018066315.1 GO:0004407 GO:0005654 GO:0007596 GO:0003714 GO:0070933 GO:0016032 GO:0044212 GO:0001078 GO:0000122 GO:0017053 GO:1990391 GO:0005667 GO:0008134 GO:0016568

XM_005682906.3 GO:0015986 GO:0000275 GO:0046933 GO:0046961

XM_018048066.1 GO:0006397 GO:0004535 GO:0005634 GO:0031251 GO:0000932 GO:0003676 GO:0000289 GO:0010606 GO:0090503

XM_018044580.1 GO:0010839 GO:0046710 GO:0030425 GO:0060170 GO:0016323 GO:0005615 GO:0042043 GO:0031982 GO:0007269 GO:0005911 GO:0042734 GO:0005604 GO:0005652 GO:0005925 GO:0006468 GO:0090288 GO:0006886 GO:0008022 GO:0045944 GO:0001953 GO:0007595 GO:0046037 GO:0005524 GO:0090280 GO:0005730 GO:0005829 GO:0016021 GO:0015629 GO:0005516 GO:0030165 GO:0061045 GO:0055085 GO:0016363 GO:0004385 GO:0004674 GO:0043234 GO:0050821 GO:0032403

XM_005683534.3 GO:0031397 GO:0070198 GO:0000784 GO:0051974 GO:0010521 GO:0005730 GO:0007080 GO:0031647 GO:0005819 GO:0000776 GO:0005739 GO:0032211 GO:0070034

XM_018061329.1 GO:0007186 GO:0016021 GO:0002230 GO:0004930

XM_018048490.1 GO:0006396 GO:0005634 GO:0006355 GO:0000079 GO:0019901

XM_018046260.1 GO:0005654 GO:0044822 GO:0005761 GO:0015935 GO:0006915

XM_018050959.1 GO:0016021

XM_018065643.1 GO:0005634 GO:0005874 GO:0032273 GO:0046785 GO:0043209 GO:0001578 GO:0008017 GO:0048471

XM_005695777.3 GO:0001558 GO:0005634 GO:0043005

XM_018053136.1 GO:0005654 GO:0005758

NM_001285631.1 GO:1901860 GO:0031490 GO:0031625 GO:0002021 GO:0014850 GO:0022904 GO:0051091 GO:0000166 GO:0043565 GO:0032922 GO:0005634 GO:2001171 GO:0043524 GO:0030374 GO:0060612 GO:0016922 GO:0042752 GO:2000507 GO:0034599 GO:1901857 GO:0045944 GO:1901863 GO:0008134

XM_013964635.2 GO:0031490 GO:0050729 GO:0007283 GO:0050796 GO:0046983 GO:0001190 GO:0005667 GO:0032922 GO:0045892 GO:0005654 GO:0000978 GO:0001046 GO:0070888 GO:0051092 GO:0000077 GO:0042634 GO:0004402 GO:0016573 GO:0005694 GO:0051775 GO:0000982 GO:2000074 GO:0043161 GO:0033391 GO:0071479 GO:0045944 GO:2000323

XM_018057035.1 GO:0005783 GO:0030054 GO:0030296 GO:0008154 GO:0030426 GO:0005829 GO:0001756 GO:0072673 GO:0008285 GO:0045211 GO:0031209 GO:0005856 GO:0070062 GO:0005634 GO:0035855 GO:0008092 GO:0061098 GO:0016032 GO:0030027 GO:0014069 GO:0030175 GO:0006928 GO:0038096 GO:0048010 GO:0032403

XM_005676159.3 GO:0070062 GO:0030036 GO:0005925 GO:0003924 GO:0000139 GO:0007155 GO:0005515 GO:0007264 GO:0005525 GO:0005886

XM_005675197.3 GO:0036020 GO:0035615 GO:0005829 GO:0005739 GO:0070062 GO:0005048 GO:0042059 GO:0007018 GO:1903077 GO:0072583 GO:0030122 GO:0050690 GO:0048013 GO:0043195 GO:0008289 GO:0006886 GO:0060071 GO:0030141 GO:0044325 GO:0019886 GO:0050750 GO:0005215

XM_018061778.1 GO:0004252 GO:0005795 GO:0006508 GO:0016021 GO:0007040 GO:0042990 GO:0006629

XM_018048953.1 GO:0035235 GO:0034220 GO:0005234 GO:0016021 GO:0030054 GO:0015277 GO:0045211

XM_018048156.1 GO:0016021 GO:0061588 GO:0046983

XM_018047125.1 GO:0016477 GO:0055037

XM_005680491.3 GO:0046875 GO:0030054 GO:0015030 GO:0043197 GO:0014069 GO:0005737 GO:0045211

XM_005684668.3 GO:0031290 GO:0005524 GO:0050919 GO:0050730 GO:0007420 GO:0005887 GO:0031952 GO:0001934 GO:0022407 GO:0048671 GO:0043525 GO:0070372 GO:0048013 GO:0008046 GO:0051964 GO:0005004 GO:0045499 GO:0043281 GO:0048755 GO:0072178

XM_018061123.1 GO:0008289 GO:0060271 GO:0035869 GO:0042953

XM_018046332.1 GO:0030154 GO:0000790 GO:0001227 GO:0000122 GO:0017151 GO:0000977 GO:0090571 GO:0008285 GO:0097011

XR_001295618.2 GO:0051260 GO:0009925

XM_018054422.1 GO:0003899 GO:0003677 GO:0005634 GO:0006366

XM_018041271.1 GO:0000166 GO:0005739

XM_018038769.1 GO:0001889 GO:0048538 GO:0051574 GO:0001227 GO:0035098 GO:0000122 GO:0048863 GO:0048536 GO:0000977 GO:0003682 GO:0031061 GO:0008285

XM_018062063.1 GO:0031012 GO:0006508 GO:0004222 GO:0005887 GO:0005509 GO:0008270 GO:0035987

XM_005686262.3 GO:1902600 GO:0046872 GO:0004129 GO:0045277 GO:0005743

XM_013965840.2 GO:0005634 GO:0016446 GO:0006974

XM_005686845.3 GO:0005685 GO:0008334 GO:0005686 GO:0006369 GO:0005515 GO:0005829 GO:0043186 GO:0071011 GO:0097526 GO:0034719 GO:0005654 GO:0044822 GO:0005732 GO:0051170 GO:0034709 GO:0005687 GO:0071013 GO:0071004 GO:0005682 GO:0000245 GO:0005689 GO:0005683 GO:0019013 GO:0000387 GO:1990446

XM_018063682.1 GO:0008654 GO:0005783 GO:0016780 GO:0005739

XM_018039399.1 GO:0045666 GO:0003714 GO:0046982 GO:0001093 GO:0043621 GO:0001077 GO:0000122 GO:0003682 GO:0005667 GO:0016021 GO:1900746 GO:0065004 GO:0005634 GO:0000978 GO:0070888 GO:0016525 GO:0001011 GO:0045944 GO:0001087 GO:0042118 GO:0006367

XM_005682573.3 GO:0017048 GO:0030335 GO:0007346 GO:0005524 GO:0097190 GO:0035556 GO:0071346 GO:0043065 GO:0051893 GO:0017148 GO:0016605 GO:0042803 GO:0008360 GO:2000249 GO:0046777 GO:0090263 GO:0004674 GO:0043519 GO:0043522

XM_018047915.1 GO:0035252 GO:0016266

XR_001917577.1 GO:0044822 GO:0070084 GO:0005737 GO:0070006 GO:0031365 GO:0046872 GO:0016485 GO:0005886 GO:0018206

XM_018054260.1 GO:0090575 GO:0000978 GO:0045666 GO:0070888 GO:0046982 GO:0001077 GO:0042803 GO:0000790 GO:0005737 GO:0043425 GO:0045944 GO:0046332

XM_018052466.1 GO:0042384 GO:0016020 GO:0005814 GO:0000226 GO:0090316 GO:0007052 GO:0034451

XM_005676753.3 GO:0019166 GO:0006631 GO:0008270 GO:0005739 GO:0055114

XM_018044697.1 GO:0006006 GO:0042407 GO:0002024 GO:0004222 GO:0006950 GO:0010637 GO:0097009 GO:0046872 GO:0016021 GO:0005743 GO:0006515 GO:0034982 GO:0006629

XM_005678746.3 GO:0043231 GO:2001030 GO:0052697 GO:0009813 GO:0046982 GO:0015020 GO:0042803 GO:0005887 GO:0043086 GO:0004857 GO:0051552 GO:0001972 GO:0005496 GO:0019899 GO:0052696

XM_005678674.3 GO:0007399 GO:0043065 GO:0005615 GO:0044822 GO:0032040 GO:0005730 GO:0006364 GO:0005737

XM_018047455.1 GO:0070062 GO:0001649 GO:0005524 GO:2001243 GO:0033687 GO:0010569 GO:0005737 GO:0000228 GO:0071479 GO:0051726

XM_018064530.1 GO:0045786 GO:0008270 GO:0016021 GO:0005737 GO:0000209 GO:0004842 GO:0016874

XR_001918619.1 GO:0000187 GO:0000077 GO:0046325 GO:0043278 GO:1902043 GO:0005875 GO:0005515 GO:0005829 GO:1902042

XM_018053715.1 GO:0005737

XM_018053100.1 GO:0010259 GO:0001889 GO:0044822 GO:0005622 GO:0006614 GO:0006807 GO:0072001 GO:0016021 GO:0006620

XM_005675566.3 GO:0006396 GO:0005634 GO:0006355 GO:0000079 GO:0019901

XM_005699191.3 GO:2000045 GO:0005654 GO:0008283 GO:0005737

XM_013966586.2 GO:0070062 GO:0001954 GO:0005654 GO:0031594 GO:0017166 GO:0005737 GO:0019901 GO:0003779 GO:0005178 GO:0008270 GO:0042383 GO:0031527 GO:0016010 GO:0070938

XM_018052650.1 GO:0046872 GO:0003676

XM_018056035.1 GO:0033120 GO:0006376 GO:0043491 GO:0005737 GO:0006302 GO:0030426 GO:0008543 GO:0001147 GO:0070301 GO:0007623 GO:0030424 GO:2000806 GO:0005654 GO:0000165 GO:0042802 GO:2000144 GO:0043066 GO:0010976 GO:0000228 GO:0071300

XM_018048301.1 GO:0042730 GO:0006879 GO:0030514 GO:0004252 GO:0005615 GO:0000122 GO:0045944 GO:0033619 GO:0016021 GO:0097264 GO:0005886

XM_018039732.1 GO:0006376 GO:0003729 GO:0005634 GO:0000381 GO:0005737 GO:0048026 GO:0090394 GO:0000166 GO:0048025

NM_001285772.1 GO:0016021 GO:0016023 GO:0005385 GO:0005886 GO:0071577

XM_018052135.1 GO:0030335 GO:0035023 GO:0043547 GO:0005886 GO:0005089

XM_013966482.2 GO:0002020 GO:0003677 GO:0008625 GO:0008656 GO:0042802 GO:0006919

XM_005685642.3 GO:0030425 GO:0005856 GO:0008021 GO:0016567 GO:0030054 GO:0042803 GO:0004842 GO:0016874 GO:0043161 GO:0008270

XM_018048007.1 GO:0045892 GO:0043069 GO:0016235 GO:0016607 GO:0042803 GO:0045893 GO:0003676 GO:0046872 GO:0046329 GO:0005794 GO:0051726 GO:0000118 GO:0003700

XM_018054760.1 GO:0006561 GO:0005759 GO:0042802 GO:0006537 GO:0055114 GO:0010133 GO:0003842 GO:0004029

XM_005683400.3 GO:0070062 GO:0005178 GO:0005509 GO:0010811

XM_018061043.1 GO:0061630 GO:0016567 GO:2000051 GO:0016874 GO:0005109 GO:0090090 GO:0060071 GO:0005887 GO:0006511 GO:0008270 GO:0072089 GO:0038018

XM_005690672.3 GO:0070829 GO:0072341 GO:0004489 GO:0050660 GO:0055114 GO:0006555 GO:0031060 GO:0050667 GO:0035999 GO:0032403

XM_005679994.3 GO:0046972 GO:0043984 GO:0043981 GO:0043995 GO:0030054 GO:0031011 GO:0051974 GO:0043996 GO:0010521 GO:0005737 GO:0071339 GO:0043982 GO:0015629 GO:0000123

XM_018047079.1 GO:0000122 GO:0000978 GO:0005634 GO:0005737 GO:0045893 GO:0001078

XM_013967707.2 GO:0045892 GO:0001958 GO:0014037 GO:0042552 GO:0005634 GO:0045682 GO:0008134

XM_018063974.1 GO:0070588 GO:0019226 GO:0005245 GO:0016247 GO:2000311 GO:0032281

XM_018042989.1 GO:0016021

XM_018044020.1 GO:0008152 GO:0008484 GO:0016021

XM_013969814.2 GO:0014068 GO:0031625 GO:0010762 GO:0038203 GO:0031932 GO:0001933 GO:0001934 GO:0005739

XM_018064582.1 GO:0005634 GO:0042803 GO:0008327 GO:0019901 GO:0001227 GO:0010428 GO:0000122 GO:0046872 GO:0000977 GO:0006974

XM_018066321.1 GO:0045892 GO:0007492 GO:0001228 GO:0005634 GO:0000977 GO:0071363 GO:0045944 GO:0060325 GO:0042481

XM_018058789.1 GO:0046872 GO:0006355 GO:0005622 GO:0003700 GO:0003676

XM_018050304.1 GO:0005654 GO:0005737 GO:0043123 GO:0000122 GO:0008270 GO:0043565 GO:0004871 GO:0008134

XM_018061942.1 GO:0071361 GO:0005622 GO:0035556 GO:0004016 GO:0000166 GO:0016021 GO:0005886 GO:0030819

XM_018048536.1 GO:0016560 GO:0007006 GO:0021795 GO:0005052 GO:0006635 GO:0005829 GO:0005739 GO:0048468 GO:0016561 GO:0005778 GO:0000038 GO:0005782 GO:0021895 GO:0051262 GO:0001764 GO:0008022 GO:0040018 GO:0050905 GO:0007029

XM_018053468.1 GO:0016021 GO:0005886

XM_005685071.3 GO:0070062 GO:0071267 GO:0008270 GO:0006577 GO:0047150 GO:0032259 GO:0005737

XM_018038933.1 GO:0005840 GO:0044822 GO:0005743 GO:0070124 GO:0003735 GO:0070125

XM_018066966.1 GO:0003779 GO:0007010

XM_018055465.1 GO:0010494 GO:0005654 GO:0042036 GO:0044822 GO:0017091 GO:0097165 GO:0017148 GO:0000166 GO:0048024

XM_005692123.3 GO:0005654 GO:0043010 GO:0019903 GO:0007601 GO:0000122 GO:0045944 GO:0043565 GO:0033169 GO:0003700 GO:0045597 GO:0048468 GO:0070207 GO:0008284

XM_005687812.3 GO:0047485 GO:0030054 GO:0043025 GO:0030252 GO:0042734 GO:0048306 GO:0031629 GO:0008021 GO:0043209 GO:0030027 GO:0007409 GO:0030175 GO:0048471 GO:0005768 GO:0070032 GO:0045121 GO:0070033 GO:0005484 GO:0032024 GO:0048791 GO:0007616 GO:0070044 GO:0051963 GO:0060291 GO:0015629 GO:0001504 GO:0008076 GO:0016197 GO:0017075 GO:0044295 GO:0031083 GO:0005249 GO:0043195 GO:0005802 GO:0016082 GO:0007626 GO:0044325 GO:0019904 GO:0008306 GO:0030431 GO:0017022 GO:0016081 GO:0071805 GO:0010975 GO:0014047

XM_018065703.1 GO:0016020 GO:0030424 GO:0005634 GO:0071156 GO:0030100 GO:0042802 GO:0045664 GO:0060987 GO:0005737 GO:0048156 GO:0043065 GO:0042692 GO:0070063 GO:0060988 GO:0048711

XM_018050472.1 GO:1902430 GO:0010875 GO:0005524 GO:0016324 GO:0030054 GO:0034188 GO:0032587 GO:0018149 GO:1900223 GO:0016021 GO:0016887 GO:0017121 GO:0032780 GO:1902995 GO:0034380 GO:0001891 GO:0038027 GO:0007613 GO:0009986 GO:1901076 GO:0032781 GO:0005794 GO:0042985 GO:0034504 GO:0070374 GO:0005548

XM_005683732.3 GO:0098655 GO:0050951 GO:0016048 GO:0005227 GO:0016021 GO:0051262

XR_001296591.2 GO:0043280 GO:0009055 GO:0020037 GO:0046872 GO:0006123 GO:0005758 GO:0070469 GO:0006122

XM_005692179.3 GO:0000122 GO:0003677 GO:0046872 GO:0005634 GO:0043392 GO:0045893 GO:0001078

XM_018057893.1 GO:0031523 GO:0000978 GO:0005654 GO:0090307 GO:0045944 GO:0001077

XM_005678419.3 GO:0006397 GO:0031965 GO:0044822 GO:0005515 GO:0008380 GO:0071011

XM_018048949.1 GO:0000122 GO:0004407 GO:0042826 GO:0000118 GO:0016575 GO:0005737

XM_018040276.1 GO:0016310 GO:0046872 GO:0016301 GO:0006470 GO:0007229 GO:0004722 GO:0005737

XM_005681537.3 GO:0002244 GO:0005634 GO:0016554 GO:0044822 GO:0000166

NM_001314283.1 GO:0032007 GO:0045859 GO:0001822 GO:0005938 GO:0043666 GO:0021766 GO:0047485 GO:0051087 GO:0017148 GO:0021987 GO:0050808 GO:0005886 GO:0008285 GO:0051492 GO:0043379 GO:0042552 GO:0006813 GO:0033596 GO:0055007 GO:0051894 GO:0006407 GO:0032868 GO:0030030 GO:0030027 GO:0002250 GO:0045792 GO:0005884 GO:0046323 GO:0001843 GO:0050821

XM_005691832.3 GO:0004303 GO:0016021 GO:0001701 GO:0001890 GO:0047035 GO:0032526 GO:0055114

XM_018061685.1 GO:0003836 GO:0018279 GO:0030173 GO:0009311 GO:0097503 GO:0001574

XM_018060667.1 GO:0005783 GO:0032781 GO:0001671 GO:0016021 GO:0051117 GO:0090435 GO:0007029

XM_018044771.1 GO:0045022 GO:0042802 GO:0015630 GO:0007286 GO:0008017 GO:0003779 GO:0070695 GO:0030897 GO:0005829 GO:0008333 GO:0007032 GO:0007040

XM_018039837.1 GO:0070062 GO:0005524 GO:0050772 GO:0032874 GO:0090168 GO:0042803 GO:0046777 GO:0046872 GO:0036481 GO:0005794 GO:0007163 GO:0004702 GO:0051683

XM_018045741.1 GO:0046872 GO:0003676

XM_018058027.1 GO:0005737

XM_018045692.1 GO:0015276 GO:0005229 GO:0005634 GO:0005887 GO:0030054 GO:1902476

XM_013965679.2 GO:0042384 GO:0030032 GO:0003779 GO:0001725 GO:0030036 GO:0045944 GO:0008270 GO:0030027

XM_013970213.2 GO:0051015 GO:0005615 GO:0032839 GO:0030036 GO:0016567 GO:0007420 GO:0004842 GO:0015629 GO:0031463

XM_018056165.1 GO:0005542 GO:0005759 GO:0008480 GO:0042426 GO:0050660 GO:0035999 GO:0055114

XM_018045029.1 GO:0031988 GO:0007283 GO:0050727 GO:0003840 GO:0002682 GO:0016021 GO:0019344 GO:0006750

XM_018049386.1 GO:0002244 GO:0005634 GO:0016554 GO:0044822 GO:0000166

XM_018041131.1 GO:0046872 GO:0005634 GO:0006355 GO:0000977 GO:0003700

XM_018061789.1 GO:0010466 GO:0005576 GO:0061045 GO:0050728 GO:0030414

XM_018039882.1 GO:0046872 GO:0005730 GO:0006355 GO:0005737 GO:2000134 GO:0003676

XM_018044963.1 GO:0010008 GO:0009897 GO:0090160 GO:0072594 GO:0044194 GO:0042470 GO:0008626 GO:0005901 GO:0005829 GO:0016021 GO:0070062 GO:0008021 GO:0061474 GO:0048471 GO:0043323 GO:0019904 GO:0005771 GO:0042383 GO:0050821 GO:0019899 GO:1902513

XM_018051179.1 GO:0005938 GO:0005102 GO:0012506 GO:0005829 GO:0043197 GO:0070062 GO:0032467 GO:0006605 GO:0043542 GO:0008021 GO:0042803 GO:0043198 GO:0003779 GO:0031647 GO:0017022 GO:0030511 GO:0032435 GO:0048167 GO:0014047

XM_018047346.1 GO:0004407 GO:0042826 GO:0070933 GO:0070491 GO:0090050 GO:0005737 GO:0032869 GO:0000122 GO:0005080 GO:0046872 GO:0005667 GO:0000118 GO:0070932 GO:0016568 GO:0034983

XM_018059737.1 GO:0008270

XM_018058757.1 GO:0003677 GO:0005634 GO:0006284 GO:0005524 GO:0006260 GO:0005694 GO:0032508 GO:0005737 GO:0006302 GO:0009378 GO:0043140 GO:0006310

NM_001285545.1 GO:0005576 GO:0042742

XM_018047023.1 GO:0005524 GO:0004683 GO:0005516 GO:0042803 GO:0046777

XM_018040233.1 GO:0035267 GO:0000812 GO:0016310 GO:1903506 GO:0006281 GO:0016578 GO:0016301 GO:0043968 GO:0033276 GO:0043967 GO:0005794 GO:0030914 GO:0003712

XM_005685278.3 GO:0019915 GO:0052689 GO:0005811 GO:0006629

XM_018067090.1 GO:1990244 GO:0016567 GO:1990245 GO:0035212 GO:0000122 GO:0005730 GO:0033151 GO:0008180 GO:0030183

XM_005678555.3 GO:0005634 GO:0016021

XM_018054160.1 GO:0030215 GO:0014912 GO:0016021 GO:0021591 GO:0014911

XM_005675409.3 GO:1902260 GO:0060539 GO:0007420 GO:0032867 GO:0019152 GO:0070402 GO:0045445 GO:0018453 GO:0004495 GO:0031234 GO:0000252 GO:0032839 GO:0007507 GO:0018451 GO:0034840 GO:0034831 GO:0007519 GO:0052677 GO:0033709 GO:0048258 GO:0035380 GO:0055114 GO:0044103 GO:0004448 GO:0008875 GO:0005829 GO:0044224 GO:0007611 GO:0008076 GO:0035410 GO:0005249 GO:0015459 GO:0043713 GO:0051990 GO:0018452 GO:0032866 GO:0043204 GO:0044325 GO:0044105 GO:0033765 GO:0032442

XM_018041569.1 GO:0048593 GO:0050673 GO:0031012 GO:0005581

XR_001919199.1 GO:0000187 GO:0032483 GO:0043547 GO:0017112 GO:0005737 GO:1902041 GO:0097194 GO:0016021 GO:0051726 GO:0005886

XM_013974613.2 GO:0003723

XM_018046304.1 GO:0023014 GO:0000278 GO:0005524 GO:0005737 GO:0019901 GO:0030296 GO:0071407 GO:0004702 GO:2001238 GO:0005654 GO:0042802 GO:0061098 GO:0060996 GO:0046777 GO:0018105 GO:2001271

XM_005690450.3 GO:0015269 GO:0016021 GO:0071805

XM_018064370.1 GO:0016020 GO:0070062 GO:0034719 GO:0032797 GO:0005730 GO:0005829 GO:0030532 GO:0006364 GO:0000387

XM_018046201.1 GO:0016020 GO:0001649 GO:0002244 GO:1900369 GO:0005737 GO:0005730 GO:0005515 GO:0045071 GO:0060216 GO:0044530 GO:0006611 GO:0035280 GO:0005654 GO:0003726 GO:0044822 GO:0051607 GO:0043066 GO:0001701 GO:0030218 GO:0035455 GO:0006382 GO:0061484 GO:0006606 GO:0045070 GO:0044387 GO:0002566 GO:0098586 GO:0060339 GO:0031054

XM_005698816.3 GO:0046920 GO:0006486 GO:0021799 GO:0032580 GO:0036065 GO:0016021 GO:0097150

XM_018049748.1 GO:0045892 GO:0046872 GO:0005634 GO:0072562 GO:0006275 GO:0006974 GO:0042803 GO:0008327

XM_018055175.1 GO:0003723

XM_005697258.3 GO:0070062 GO:0030140 GO:0019003 GO:0032585 GO:0045921 GO:0042470 GO:0071985 GO:0003924 GO:0005795 GO:0019904 GO:0007264 GO:0031489 GO:0005525

XM_005678549.3 GO:0005654 GO:0004853 GO:0005829 GO:0006782

XM_018059529.1 GO:0097084 GO:0005615 GO:0050829 GO:0045906 GO:0005737 GO:0005179 GO:0043116 GO:0060712 GO:0050830 GO:0045766 GO:0030819 GO:2001214 GO:0007507 GO:0060670 GO:0048589 GO:0002031 GO:0001843 GO:0019731 GO:0008284

XM_018058187.1 GO:0010587 GO:0051306 GO:0000287 GO:0034427 GO:0000175 GO:0000932 GO:0019827 GO:0090503 GO:0008266 GO:0051301 GO:1990074 GO:0008285

XM_018038287.1 GO:0003677 GO:0005654 GO:0035518 GO:0003714 GO:0005737 GO:0006915 GO:0000122 GO:0031519 GO:0005515 GO:0007275 GO:0008270

XM_018046846.1 GO:0048013

XM_005677636.3 GO:0040009 GO:0005634 GO:0042802 GO:0005829 GO:0006915 GO:0008285

XM_018062318.1 GO:0007399 GO:0008270 GO:0071565 GO:0003676

XM_018058826.1 GO:0015018 GO:0005975 GO:0050650 GO:0016021 GO:0030204

XM_018050081.1 GO:0005237 GO:0007214 GO:0030054 GO:0051932 GO:0005887 GO:0005829 GO:0071420 GO:0004890 GO:0005254 GO:1902476 GO:0045211 GO:0070062 GO:0060384 GO:0043524 GO:0034707 GO:1902711 GO:0007605 GO:0060119 GO:0090102

XM_018065799.1 GO:0005654 GO:0071260 GO:0005524 GO:0006468 GO:0005737 GO:0045893 GO:0019901 GO:0004672

XM_005675625.3 GO:0016021

XR_001919297.1 GO:0005814

XR_001918360.1 GO:0042393 GO:0005654 GO:0006355 GO:0000790 GO:0006335 GO:0043234 GO:0006336 GO:0016568

XM_018057576.1 GO:0007186 GO:0016525 GO:0016021 GO:0007166 GO:0007422 GO:0051965 GO:0004930

XM_005677164.3 GO:0070062 GO:0006486 GO:0046872 GO:0032580 GO:0003945 GO:0016021 GO:0003831

XM_005681555.3 GO:0008270

XM_005684606.2 GO:0008270 GO:0005739 GO:0055114 GO:0016491

XM_018041555.1 GO:0005929 GO:0030317 GO:0005634 GO:0005874 GO:0036159 GO:0005737

XM_018040338.1 GO:0048009

XM_018038613.1 GO:0003676

XM_013971698.2 GO:0007186 GO:0016021 GO:0016503

XM_005697147.3 GO:0005654 GO:0016787 GO:0005794 GO:0016021 GO:0006888

XM_005678194.3 GO:0007186 GO:0004958 GO:0016021 GO:0005886

XM_018066751.1 GO:0070534 GO:0070936 GO:0005634 GO:0042787 GO:0048588 GO:0005737 GO:0004842 GO:0071425 GO:0008270

XM_018063561.1 GO:0016323 GO:0070062 GO:0036393 GO:0020037 GO:0005615 GO:0055114 GO:0005737 GO:0006979 GO:0018969 GO:0005509 GO:0001580 GO:0042744 GO:0019731

XM_018045778.1 GO:0070062 GO:0051491 GO:0072583 GO:0043547 GO:0005086 GO:0016023 GO:0005802 GO:0006900 GO:0042384 GO:0010324 GO:0097320 GO:0030050

XM_018046608.1 GO:0031625 GO:0005737

XM_018064727.1 GO:0008104 GO:0005739 GO:0005886

XM_018063725.1 GO:0016021

XM_005680367.3 GO:0070062 GO:0005509

XR_001919786.1 GO:0005524 GO:0006468 GO:0043547 GO:0007185 GO:0043065 GO:0004674 GO:0005829 GO:0035023 GO:0019899 GO:0005089

XM_018063745.1 GO:0070534 GO:0061630 GO:0005634 GO:0044822 GO:0006513 GO:0042147 GO:0031625 GO:0030513 GO:0061631 GO:0005829

NM_001314142.1 GO:0032930 GO:0008083 GO:0008354 GO:0005737 GO:0002513 GO:0070306 GO:0045216 GO:0042130 GO:0014003 GO:0060751 GO:0007492 GO:1900126 GO:0045591 GO:0009611 GO:0042482 GO:0007182 GO:0005114 GO:0048146 GO:0060744 GO:0045599 GO:0010800 GO:0033601 GO:0045662 GO:0002062 GO:0043029 GO:0031334 GO:0050921 GO:0045944 GO:0007219 GO:0033138 GO:0043536 GO:0002244 GO:0000122 GO:0006754 GO:0010862 GO:0034714 GO:0035066 GO:0006611 GO:1903620 GO:0006874 GO:0008156 GO:0030308 GO:0032801 GO:0048535 GO:0005902 GO:0006954 GO:0050714 GO:0060395 GO:0032700 GO:0050680 GO:0043537 GO:0060325 GO:0050731 GO:0010763 GO:2000679 GO:0097191 GO:0002028 GO:1901666 GO:0032740 GO:0000060 GO:0032570 GO:0032967 GO:0010936 GO:0017015 GO:0032355 GO:0007173 GO:0031663 GO:0003823 GO:0043117 GO:0048642 GO:1903077 GO:0005125 GO:2000249 GO:0007093 GO:0043552 GO:0001933 GO:0043932 GO:0048298 GO:0061035 GO:0043011 GO:0051897 GO:0060965 GO:0030214 GO:0035307 GO:0043539 GO:0007183 GO:0051781 GO:0031065 GO:0071407 GO:0085029 GO:0019049 GO:0034713 GO:0070723 GO:0005634 GO:0072562 GO:0060391 GO:0043406 GO:0010718 GO:0005578 GO:0060762 GO:0009986 GO:0030501 GO:0071158 GO:0030279 GO:0007435 GO:0032667 GO:0001948 GO:0001657 GO:0042981 GO:0019899

XM_018051872.1 GO:0005634 GO:0016446 GO:0006974

XM_005684344.3 GO:0070062 GO:0005765 GO:1902600 GO:0005829 GO:0051117 GO:0008553 GO:0005886 GO:0016471

XM_005679865.3 GO:0005634 GO:0006355 GO:0007275 GO:0043565 GO:0003700

XM_018047106.1 GO:0061630 GO:0043234 GO:0016567 GO:0008270 GO:0005886 GO:0030659

XM_018038776.1 GO:0051291 GO:0009986 GO:0005509 GO:0051260

XM_018041542.1 GO:0016020 GO:0070578 GO:0044822 GO:0072091 GO:0003725 GO:0005737 GO:0006402 GO:0035198 GO:0035068 GO:0010501 GO:0016442 GO:0035280 GO:0035278 GO:0031054 GO:0003727

XM_018052935.1 GO:0030276 GO:0005545 GO:0030136 GO:0005905 GO:0048268 GO:2000369 GO:0007268 GO:0005886

XM_018064450.1 GO:0007099 GO:0005813 GO:0005737

XM_018064235.1 GO:0016021

XM_018061846.1 GO:0046872 GO:0005654

XM_018040169.1 GO:0005524

XM_018059473.1 GO:0005524 GO:0043491 GO:0030513 GO:0045893 GO:0019901 GO:0001658 GO:0007229 GO:0022011 GO:0005886 GO:0005654 GO:0034446 GO:0005925 GO:0030030 GO:2000178 GO:0003151 GO:0030017 GO:0030027 GO:0045669 GO:0090263 GO:0010761 GO:0021675 GO:0042327 GO:0018105 GO:0004674 GO:0070527 GO:0045197 GO:0008284

XM_018045689.1 GO:0005520 GO:0030335 GO:0030513 GO:0060710 GO:0005178 GO:0002041 GO:0045860 GO:0008201 GO:0003181 GO:0010811 GO:0044319 GO:0050840 GO:0003281 GO:0001558 GO:0043280 GO:0061036 GO:0060716 GO:0070372 GO:0007267 GO:0043066 GO:0003278 GO:0072593 GO:0045669 GO:0005578 GO:2000304 GO:0060413 GO:0010518 GO:0030198 GO:0045944 GO:0060591 GO:0033690

XM_018051845.1 GO:0016021

XM_005682380.3 GO:0070062 GO:0016021

XM_018048230.1 GO:0016589

XM_013973459.2 GO:0046872 GO:0006355 GO:0005622 GO:0003676

XM_018044176.1 GO:0045944 GO:0031307 GO:0034613

XM_018049492.1 GO:0045665 GO:0043065 GO:0006357 GO:0046872 GO:0000981 GO:0005634 GO:0003676

XM_018058980.1 GO:0006509 GO:0070062 GO:0003729 GO:0000381 GO:0051260 GO:0071013 GO:0048026 GO:0071347 GO:0045944 GO:0000166 GO:0003682 GO:0005719 GO:0044530 GO:0048025 GO:0001047

XM_018057623.1 GO:0097494 GO:0070062 GO:0019003 GO:0003924 GO:0045335 GO:0007264 GO:0006897 GO:0015629 GO:0007032 GO:0005525 GO:0005769 GO:0005886

XM_018058162.1 GO:0045022 GO:0006622 GO:0005770 GO:0042802 GO:0005829 GO:0008333 GO:0031313 GO:0005769 GO:0035091

XM_005675377.3 GO:0070062 GO:0000165 GO:0046872 GO:0006470 GO:0005622 GO:0007178 GO:0004722

XM_005684278.2 GO:0005856 GO:0001673 GO:0031514 GO:0005794 GO:0043234

XM_005700424.3 GO:0045190 GO:0032733 GO:0005615 GO:0009897 GO:0005174 GO:0042102 GO:0030168 GO:0005164 GO:0032735 GO:2000353 GO:0016021 GO:0030183 GO:0032753 GO:0005125 GO:0051092 GO:0005622 GO:0043066 GO:0007257 GO:0006954 GO:0042100 GO:0051023

XM_005691805.3 GO:0060170 GO:0043010 GO:0001701 GO:0042733 GO:0042384 GO:0001944 GO:0032880 GO:0016021 GO:0036038 GO:0007224 GO:0060563

XM_018052249.1 GO:0016021 GO:0015238 GO:0006855

XM_005683211.3 GO:0003219 GO:0001829 GO:0043433 GO:0046982 GO:0060485 GO:0005737 GO:0001707 GO:0000122 GO:0060536 GO:0005730 GO:0090575 GO:0005654 GO:0000978 GO:0060411 GO:0001525 GO:1903026 GO:0003218 GO:0055010 GO:0042803 GO:0042475 GO:0060707 GO:0001078 GO:0001947 GO:0003713 GO:0043425 GO:0045944 GO:0019899 GO:0003144

XM_018059294.1 GO:0009786 GO:0000132 GO:0031625 GO:0001568 GO:0007601 GO:0001077 GO:0005737 GO:0003309 GO:0042593 GO:0019901 GO:0070410 GO:0021983 GO:0030858 GO:0003682 GO:0007224 GO:0002052 GO:0002088 GO:0007411 GO:0009611 GO:0071837 GO:0005654 GO:0000978 GO:0021796 GO:0061303 GO:0021905 GO:0000790 GO:0021778 GO:0001227 GO:0048505 GO:0001764 GO:0001933 GO:0003322 GO:0045944 GO:0033365 GO:0021798 GO:0042462 GO:0060041 GO:0030216 GO:0016567 GO:0023019 GO:0000122 GO:0021797 GO:0021902 GO:0000979 GO:0030334 GO:0032808 GO:0021913 GO:0070412 GO:0048596 GO:0001709 GO:2000178 GO:0004842 GO:0021918 GO:0061072 GO:0045665 GO:0009950 GO:0048708 GO:0007435 GO:0050680 GO:0021912 GO:0035035 GO:0008134

XM_005675973.2 GO:0005634 GO:0045444 GO:0004402 GO:0016573 GO:0007519 GO:0005737

XM_018060510.1 GO:0043123 GO:0061630 GO:0000151 GO:0016567 GO:0008270 GO:0004871 GO:0005769

XM_005696939.3 GO:0006364

XM_018050191.1 GO:0005230 GO:0034220 GO:0016021

XM_013971512.2 GO:0032060 GO:0016021 GO:0008219 GO:0016049 GO:0005886

XM_005681921.3 GO:0005634 GO:0035019 GO:0000989 GO:0030274 GO:0005667 GO:0045944 GO:0010669 GO:0001942 GO:0019899 GO:0044089

XM_018039329.1 GO:0007601 GO:0001750 GO:0071277 GO:0007602 GO:0031284 GO:0005515 GO:0005509 GO:0008048 GO:0005886

XR_001919509.1 GO:0005525

XM_018053143.1 GO:0000122 GO:0005634 GO:0001206 GO:0000980

XM_005680671.3 GO:0004896 GO:0016021 GO:0019221

XM_018044162.1 GO:0007165 GO:0016021

XR_001917840.1 GO:0005887 GO:0005246 GO:0007166 GO:1903169

XR_001917755.1 GO:0001533

NM_001286092.1 GO:0007186 GO:0051924 GO:0032781 GO:0016021 GO:0010628 GO:0005886 GO:0004930

XM_005696535.3 GO:0005634 GO:0030326 GO:0005667 GO:0003682 GO:0045944 GO:0043565 GO:0009954 GO:0008134 GO:0003700

XM_005677344.2 GO:0051087 GO:0005737

XM_013976584.2 GO:0043154 GO:0048027 GO:0008625 GO:0017148 GO:0043273 GO:0045087 GO:0070062 GO:2001243 GO:0043024 GO:0003924 GO:0004003 GO:0008190 GO:0004004 GO:0045944 GO:0016055 GO:0045070 GO:0010501 GO:0031333 GO:0010494 GO:0008143 GO:0003677 GO:0035613 GO:0005524 GO:0007059 GO:0071243 GO:0030307 GO:0045948 GO:0032508 GO:0042256 GO:0030308 GO:1900087 GO:0043280 GO:0034063 GO:0005634 GO:0071470 GO:0022627 GO:0009615 GO:0005852 GO:0008134

XM_005690474.3 GO:0010923 GO:0003676

XM_005681382.3 GO:0070062 GO:0044822 GO:0005730 GO:0008168 GO:0030488 GO:0000049

XM_018038248.1 GO:0023014 GO:0000287 GO:0070062 GO:2000687 GO:0005524 GO:0035556 GO:0005737 GO:0018107 GO:0006979 GO:1901017 GO:0071476 GO:0004702

XM_018051274.1 GO:0031965 GO:0006607 GO:0008565 GO:0000060 GO:0005737 GO:0008139 GO:0034399 GO:0008536 GO:0006610 GO:0000059

XM_005683127.3 GO:1900004 GO:0004867 GO:0005576

XM_018054687.1 GO:0032725 GO:2000667 GO:0042802 GO:0005576 GO:2000778 GO:0006915 GO:0019841

XM_005675898.2 GO:0003723 GO:0000398 GO:0071006 GO:0005794 GO:0016607 GO:0071013

XM_013968513.2 GO:0008283 GO:0005730 GO:0006260 GO:0042802 GO:0006974 GO:0019899 GO:0005737

XM_018062433.1 GO:0045742 GO:0051260

XM_018047392.1 GO:0005313 GO:0089711 GO:0015183 GO:0043490 GO:0006754 GO:0051592 GO:0089712 GO:0005509 GO:0005743 GO:0016021

XM_018040224.1 GO:0016874 GO:2000060 GO:0030512 GO:0070412 GO:0006611 GO:0005739 GO:0005886 GO:0000209 GO:0071211 GO:0070062 GO:0032801 GO:0005634 GO:0061630 GO:0030509 GO:0030579 GO:0030279 GO:0043161 GO:0070411 GO:0002230 GO:0034394

XM_018049410.1 GO:0061092 GO:0000287 GO:0070062 GO:0005783 GO:0030335 GO:0005524 GO:0031410 GO:0007612 GO:0005802 GO:0048194 GO:0005515 GO:0016021 GO:0004012 GO:0005886

XM_005682415.2 GO:0005759 GO:0005743 GO:0051087 GO:0030150

XM_005680988.3 GO:0016020 GO:0032237 GO:0005730 GO:0005794 GO:0005509 GO:0007264 GO:0005525 GO:0002115

NM_001285645.1 GO:0016020

NM_001285560.1 GO:0006508 GO:0004190

XM_018042454.1 GO:0005634

XM_013963581.2 GO:0033179 GO:0015991 GO:0016021 GO:0015078

XM_005697544.3 GO:0007200 GO:0008188 GO:0016021 GO:0007218 GO:0046983

XM_005691841.3 GO:0050832 GO:0003779 GO:0070062 GO:0005856 GO:0005634 GO:0019899 GO:0005737

XM_018061274.1 GO:0030901 GO:0030307 GO:0007283 GO:0000122 GO:0000979 GO:0008270 GO:0021555 GO:0048596 GO:0035518 GO:0021670 GO:0043524 GO:2000178 GO:0070544 GO:1902459 GO:0021993 GO:0031519 GO:0021592 GO:0021678

XM_005679979.3 GO:0051233 GO:0035556 GO:0043547 GO:0007283 GO:0051256 GO:0019901 GO:0030496 GO:0007405 GO:0000281 GO:0043015 GO:0048487 GO:0043014 GO:0031234 GO:0051988 GO:0070062 GO:0032467 GO:0005654 GO:0000915 GO:0097149 GO:0008272 GO:0008017 GO:0005547 GO:0046872 GO:0032154 GO:0009790 GO:0005096

XM_018042296.1 GO:0016567 GO:0007283 GO:0004842 GO:0016874

XM_013967845.2 GO:2000344 GO:0016298 GO:0016021 GO:0006629

XM_018053985.1 GO:0046872 GO:0003676

XM_018041849.1 GO:0070062 GO:0005524 GO:0005887 GO:0048013 GO:0006468 GO:0005005

XM_005675479.3 GO:0006396 GO:0005654 GO:0044822 GO:0000166

XM_018060397.1 GO:0003677 GO:0006338 GO:0001105 GO:0021930 GO:0048813 GO:0021942 GO:0001106 GO:0000122 GO:0045944 GO:0003682 GO:0048755 GO:0000118 GO:0021691

XM_013972159.2 GO:0070062 GO:0005654 GO:0000220 GO:0051117 GO:0015991 GO:1901998 GO:0005794 GO:0016021 GO:0015078 GO:0005886

XM_018051865.1 GO:0000978 GO:0046872 GO:0005634 GO:0005737

XM_005683394.3 GO:0051351 GO:0003677 GO:0005654 GO:0010165 GO:0005813 GO:0032807 GO:0030054 GO:0005958 GO:0008022 GO:0051103 GO:0005829 GO:0006303 GO:0006310

XM_018050615.1 GO:0044341 GO:0035725 GO:0016324 GO:0005903 GO:0030643 GO:0005436 GO:0005737 GO:0031982 GO:0046686 GO:0010288 GO:0046689 GO:0005315 GO:0016021 GO:0035435 GO:0015321

XM_013974854.2 GO:0004535 GO:0005634 GO:0044822 GO:0000184 GO:0005737 GO:0019901 GO:0046872 GO:0000166 GO:0090503

XM_013971017.2 GO:0043484 GO:0005634 GO:0005524 GO:0046777 GO:0018107 GO:0004713 GO:0004674 GO:0018105 GO:0018108

XM_018047832.1 GO:0005685 GO:0003723 GO:0071004 GO:0000398

XM_018047096.1 GO:0000122 GO:0046872 GO:0017053 GO:0003714 GO:0005730 GO:0005737 GO:0006629 GO:0003676

XM_018041056.1 GO:0031119 GO:0003723 GO:0019239 GO:0005739 GO:0009982

XM_018054363.1 GO:0000185 GO:0005524 GO:0008349 GO:0005737

XM_018061871.1 GO:0045892 GO:0005634 GO:0003714 GO:1903715 GO:0032436 GO:0001666 GO:0045820 GO:0030851 GO:0003700 GO:0008285

XM_005681254.3 GO:0017091 GO:0000176 GO:0030307 GO:0034476 GO:0071028 GO:0000467 GO:0071035 GO:0005730 GO:0000177 GO:0071038 GO:0071042 GO:0043928 GO:0034473 GO:0070062 GO:0005654 GO:0044822 GO:0034427 GO:0000228 GO:0034475

XM_018046697.1 GO:0005730 GO:0005737

XM_018044881.1 GO:0005856

XM_018044683.1 GO:0046872

XM_018063875.1 GO:0007186 GO:0016021 GO:0004930

XM_005685087.3 GO:0006626 GO:0005741

XM_018054447.1 GO:0045892 GO:0003682 GO:0031507 GO:0005677

XM_018063902.1 GO:0005634 GO:0005794 GO:0016021 GO:0005385 GO:0005886 GO:0071577

XM_018039521.1 GO:0006446 GO:0003723 GO:0000184 GO:0048471

XM_013963962.2 GO:0016020 GO:0004709 GO:0005524 GO:0051092 GO:0042803 GO:0007254 GO:0046777 GO:0008385 GO:0000186

XM_013965655.2 GO:0043402 GO:0000978 GO:0005815 GO:0005654 GO:0001077 GO:0042921 GO:0045944 GO:0005819 GO:0008270 GO:0005739 GO:0016568 GO:0038051 GO:1990239

XM_018050690.1 GO:0005634 GO:0005524 GO:0008360 GO:0005737 GO:0004674 GO:0018105 GO:0016055 GO:0006897

XR_001918374.1 GO:0006281 GO:0090305 GO:0004518

XM_018049058.1 GO:0010633 GO:0005737 GO:0016021 GO:0030838 GO:1900028 GO:0005856 GO:0070062 GO:2000300 GO:0003785 GO:0043195 GO:0005546 GO:0051496 GO:0032781 GO:0030837 GO:0000774 GO:0033138 GO:0050821

XM_018061537.1 GO:0010629 GO:0032496 GO:0000932 GO:0045668 GO:0090503 GO:0032922 GO:0004535 GO:0005634 GO:0003729 GO:0009991 GO:0045995 GO:0045600 GO:0000290 GO:0048471 GO:0042752 GO:0033962 GO:0048255

XM_018041303.1 GO:0043130

XM_018061340.1 GO:0006810 GO:0043231 GO:0046872

XM_018059340.1 GO:0043154 GO:0035612 GO:0016323 GO:0031434 GO:0035774 GO:0032715 GO:0031625 GO:0002092 GO:0032092 GO:0031896 GO:0043197 GO:0031143 GO:0032088 GO:0005765 GO:0005654 GO:0036276 GO:0005834 GO:0034393 GO:0014069 GO:0070373 GO:0035025 GO:0045944 GO:0031691 GO:0031692 GO:0034260 GO:0032717 GO:0033138 GO:0090240 GO:0031397 GO:0031762 GO:0030331 GO:0043027 GO:0043547 GO:0030168 GO:0000139 GO:0007602 GO:0042699 GO:0045309 GO:0005829 GO:0035615 GO:0005159 GO:0045211 GO:0030659 GO:0043280 GO:0000187 GO:0000785 GO:0004402 GO:0031398 GO:0044212 GO:0044325 GO:0043161 GO:0005905 GO:0070374 GO:0002031 GO:0031701 GO:0008134 GO:0043149 GO:0005096

XM_018052093.1 GO:0030425 GO:0043267 GO:0045634 GO:0031683 GO:0043065 GO:0009791 GO:0031826 GO:0021884 GO:0004871 GO:0007215 GO:0070062 GO:0005765 GO:0035412 GO:0007213 GO:0031965 GO:0007507 GO:0005834 GO:0043066 GO:0001750 GO:0003924 GO:0007202 GO:0001508 GO:0047391 GO:0005525 GO:0016322 GO:0043547 GO:0042733 GO:0005901 GO:0005829 GO:0007189 GO:0070208 GO:0009649 GO:0060158 GO:0006469 GO:0046872 GO:0044297 GO:0007603 GO:0048661 GO:0050821 GO:0001501 GO:0005096 GO:0042711

XM_018056814.1 GO:0007585 GO:0016525 GO:0005576 GO:0016021

XM_018040430.1 GO:0097233 GO:0055085 GO:0005615 GO:0005524 GO:0006869 GO:0042626 GO:0016021 GO:0005886

XM_005679874.3 GO:0005184

XM_018057785.1 GO:0005635 GO:0090286 GO:0016021 GO:0006998

XM_005696349.2 GO:0006412 GO:0005763 GO:0003735

XM_013963318.2 GO:0007186 GO:0005783 GO:0005515 GO:0005057 GO:0005887 GO:0004930

XM_018066407.1 GO:0031012 GO:0005615 GO:0016641 GO:0005507 GO:0055114

XM_018057573.1 GO:0005634 GO:0044822 GO:0097190 GO:0008270 GO:0006351

XM_005695293.3 GO:0006357 GO:0003677 GO:0046872 GO:0000981 GO:0005634

XM_005699882.3 GO:0030176 GO:0005637 GO:0006695 GO:0050613 GO:0055114 GO:0043235

XM_018065073.1 GO:0045095

XM_005693514.3 GO:0004175 GO:0004843 GO:0005730 GO:0016926 GO:0016929

XM_018057552.1 GO:0007186 GO:0016525 GO:0016021 GO:0007166 GO:0007422 GO:0051965 GO:0004930

XM_018050525.1 GO:0003677 GO:0005634 GO:0006355

XM_018041091.1 GO:0035023 GO:0043547 GO:0005089

XM_005679827.3 GO:0006979 GO:0009055 GO:0005747 GO:0055114 GO:0008137

XM_018064411.1 GO:0006886 GO:0012505 GO:0034499 GO:0031338 GO:0017137 GO:0005737 GO:0005096

XM_005679459.3 GO:0031965 GO:0008565 GO:0042802 GO:0035048 GO:0005737 GO:0008139

XM_005689295.2 GO:0010043 GO:0030073 GO:0042803 GO:0005385 GO:0061088 GO:0030141 GO:0016021 GO:0009749 GO:0005886 GO:0071577

XM_013968921.2 GO:0042110 GO:0005070 GO:0005770 GO:0047485 GO:0016023 GO:0000122 GO:0009967 GO:0005886 GO:0050849

XM_018065112.1 GO:0007595 GO:0003677 GO:0007259 GO:0038161 GO:0050729 GO:0005737 GO:0019218 GO:0004871 GO:0003700 GO:0045647 GO:0042104 GO:0007565 GO:0005654 GO:0030856 GO:0045931 GO:0001779 GO:0043066 GO:0045579 GO:0045086 GO:0043029 GO:0019915 GO:0001553 GO:0045944 GO:0040018 GO:0046543

XM_018063034.1 GO:0016021

XM_013967771.2 GO:0007186 GO:0050911 GO:0004984 GO:0016021 GO:0005886 GO:0004930

XM_013962623.2 GO:0061087 GO:0035064 GO:0005737 GO:0035098 GO:0048863 GO:0000122 GO:0061086 GO:0019827 GO:0045944 GO:0008270 GO:0007379

XM_018040289.1 GO:0046872 GO:0003676

XM_005699193.3 GO:2000045 GO:0005654 GO:0008283 GO:0005737

XM_018066828.1 GO:0007129 GO:0000793 GO:0006281 GO:0070182 GO:0005737 GO:0010332 GO:0005730 GO:0007276

XM_018038883.1 GO:0042612 GO:0042605 GO:0006955 GO:0016021 GO:0002474

XM_005693092.3 GO:0005840 GO:0031965 GO:0044822 GO:0045111 GO:0006412 GO:0003735 GO:0005739

XM_018060702.1 GO:0000309 GO:0005524 GO:0005770 GO:0004515 GO:0005802 GO:0045202 GO:0009435

XM_005679511.3 GO:0000978 GO:0005783 GO:0005654 GO:0001077 GO:0002062 GO:0035497 GO:0045944 GO:0006888 GO:0030968

XM_018056962.1 GO:0010951 GO:0004867 GO:0005576 GO:0030212

XM_005676737.2 GO:0010494 GO:0008344 GO:0007283 GO:0003730 GO:0000932 GO:0048863 GO:1900246 GO:2000637 GO:0051726 GO:0061157

XM_005675847.3 GO:0006574 GO:0070062 GO:0009083 GO:0005739 GO:0003860

NM_001314233.1 GO:0046034 GO:0055131 GO:0005524 GO:0005770 GO:0051085 GO:0031072 GO:0031625 GO:0000974 GO:0042470 GO:0005681 GO:0005730 GO:0072318 GO:0005829 GO:0001786 GO:0005886 GO:0045892 GO:0070062 GO:0072562 GO:0005925 GO:0044822 GO:0043209 GO:0042623 GO:0023026 GO:0048026 GO:1902904 GO:0001664 GO:0031647 GO:0051082 GO:0000151 GO:0051726 GO:0042026

XM_018066167.1 GO:0016021

XM_018039424.1 GO:0003779 GO:0005623 GO:0051295 GO:0045010 GO:0016192

XM_018042083.1 GO:0008168 GO:0032259

XM_013974155.2 GO:0070453 GO:0005743 GO:0016021 GO:0030218 GO:0006839

XM_005685069.3 GO:0005654 GO:0030054 GO:0005802 GO:0042589 GO:0055038 GO:0030672 GO:0016021 GO:0015031 GO:0006887

XR_001918083.1 GO:0008277 GO:0001965 GO:0005829 GO:0005813 GO:0043547 GO:0005085 GO:0005886 GO:0005096

XM_018058059.1 GO:0016021

XM_018040914.1 GO:0030308 GO:0045116 GO:0051443 GO:0010332 GO:0031624 GO:0048471 GO:0010225 GO:0043065 GO:0000151 GO:0005886 GO:0032182 GO:0097602

XM_018053281.1 GO:0046872 GO:0005654 GO:0003676

XM_013976222.2 GO:0016758

XM_018067168.1 GO:0016021

XM_013972399.2 GO:0045747 GO:0031490 GO:0019903 GO:0001077 GO:0005737 GO:0097009 GO:0042593 GO:0046983 GO:0019901 GO:0032355 GO:0070102 GO:0008285 GO:0005654 GO:0000978 GO:0042802 GO:0043066 GO:0060019 GO:0000790 GO:0030522 GO:0019827 GO:0045944 GO:0006606 GO:0045820 GO:0051726 GO:0001659 GO:0040014 GO:0001103 GO:0001754 GO:0019953 GO:0033210 GO:0005886 GO:0060397 GO:0090575 GO:0042755 GO:0060259 GO:0004879 GO:0048708 GO:2001223

XM_005683743.3 GO:0098655 GO:0050951 GO:0016048 GO:0005227 GO:0016021 GO:0051262

XM_018065857.1 GO:0004674 GO:0005524 GO:0006468

XM_018063637.1 GO:0050700 GO:0051092 GO:0001934 GO:0043066 GO:0033209 GO:0005737

XM_013972739.2 GO:0007228 GO:0005634 GO:0005524 GO:0006468 GO:0007420 GO:0005737 GO:0003351 GO:0042384 GO:0009791 GO:0004672 GO:0045880 GO:0008134

XM_018047989.1 GO:0005524 GO:0002218 GO:0044565 GO:0032727 GO:0010629 GO:0005737 GO:0003676 GO:0045359 GO:0043123 GO:0004674 GO:0018105 GO:0045944 GO:0050830 GO:0051219

XM_013976203.2 GO:0016021

XM_018053319.1 GO:0010468 GO:0043547 GO:0030193 GO:0017157 GO:0030141 GO:0050708 GO:0016021 GO:0031201 GO:0019905 GO:0005886 GO:0017137 GO:0005096

XM_005699744.3 GO:0042384 GO:0005774 GO:0005929 GO:0016021

XM_018063410.1 GO:0000122 GO:0046872 GO:0000981 GO:0005654 GO:0005737 GO:0006915 GO:0003676

XM_018065979.1 GO:0042393 GO:0070062 GO:0005654 GO:0060218 GO:0044822 GO:0005524 GO:0001046 GO:0006355 GO:0007517 GO:0005730 GO:0006974

XM_018040236.1 GO:0035267 GO:0000812 GO:0016310 GO:1903506 GO:0006281 GO:0016578 GO:0016301 GO:0043968 GO:0033276 GO:0043967 GO:0005794 GO:0030914 GO:0003712

XM_018055591.1 GO:0019915 GO:0005811

XM_018047824.1 GO:0003779 GO:0016477 GO:0017048 GO:0030036 GO:0008360 GO:0005737 GO:0032794

XM_005675264.3 GO:0005874 GO:0018009 GO:0007026 GO:0005798 GO:0005516 GO:0008017 GO:0005801

XM_018039074.1 GO:0008542 GO:0043113 GO:0046580 GO:0016358 GO:0043524 GO:0043547 GO:0043408 GO:0005737 GO:0007389 GO:0043198 GO:0050771 GO:0048169 GO:0031235 GO:0005096

XM_018057118.1 GO:0050829 GO:0042802 GO:0030674 GO:0031410 GO:0001920 GO:0005802 GO:0048471 GO:0070530 GO:0043001 GO:0008022 GO:0000042 GO:0090161 GO:0043124 GO:0017137

XR_001917868.1 GO:0005856 GO:0007165 GO:0008360 GO:0005737 GO:0005095 GO:0005198 GO:0034260 GO:0005886 GO:0006909

XM_005681573.3 GO:0030424 GO:0001505 GO:0005230 GO:0007214 GO:0030054 GO:0034707 GO:0008503 GO:1902711 GO:0005254 GO:0030285 GO:0004890 GO:0006836 GO:1902476 GO:0045211

XM_005686141.3 GO:0008083 GO:0005615 GO:0042127 GO:0032967 GO:0032570 GO:0016049 GO:0010936 GO:0045893 GO:0045216 GO:0007179 GO:0010862 GO:0034714 GO:0070483 GO:0050431 GO:0034713 GO:0008156 GO:0031012 GO:0005125 GO:0005114 GO:0042802 GO:0010718 GO:0043408 GO:0045740 GO:0050714 GO:0060395 GO:0030501 GO:0043932 GO:0007435 GO:0060325 GO:0042981

XM_018043489.1 GO:0046872 GO:0005794 GO:0016021 GO:0016192

XM_018055187.1 GO:2000273 GO:0010763 GO:0030546 GO:0005783 GO:2001031 GO:0050730 GO:0035669 GO:0035276 GO:0031663 GO:0032467 GO:0071361 GO:0005856 GO:0035641 GO:0003785 GO:0050996 GO:0048471 GO:0090303 GO:0018105 GO:0032230 GO:0071380 GO:0008047 GO:0071456 GO:0031397 GO:0032024 GO:0005524 GO:0070257 GO:0043278 GO:0051279 GO:0071889 GO:0010634 GO:0061178 GO:0005829 GO:0002281 GO:0005739 GO:0030838 GO:0010811 GO:0005886 GO:0043085 GO:0005634 GO:0043123 GO:0046872 GO:0004699 GO:0005794 GO:0043410 GO:0019899

XM_005678653.3 GO:0005524 GO:0006400 GO:0052381 GO:0005739

XM_018065113.1 GO:0007595 GO:0003677 GO:0007259 GO:0038161 GO:0050729 GO:0005737 GO:0019218 GO:0004871 GO:0003700 GO:0045647 GO:0042104 GO:0007565 GO:0005654 GO:0030856 GO:0045931 GO:0001779 GO:0043066 GO:0045579 GO:0045086 GO:0043029 GO:0019915 GO:0001553 GO:0045944 GO:0040018 GO:0046543

XM_018047740.1 GO:0008217 GO:0005025 GO:0030513 GO:0060836 GO:0019901 GO:0016361 GO:0061154 GO:0005887 GO:0004702 GO:0045766 GO:0001937 GO:0032924 GO:2000279 GO:0001974 GO:0045944 GO:0061298 GO:0023014 GO:0005524 GO:0048185 GO:0060841 GO:0007179 GO:0010862 GO:0001938 GO:0050431 GO:0045602 GO:0030308 GO:0001701 GO:0045603 GO:0051895 GO:0060840 GO:0009986 GO:0035313 GO:0046332 GO:0001946 GO:0043537

XM_018041873.1 GO:0070062 GO:0005634 GO:0008308 GO:0005741

XM_018042451.1 GO:0043473 GO:0042267 GO:0015630 GO:0032510 GO:0015031 GO:0005737 GO:0007040

XM_018056627.1 GO:0010882 GO:0051533 GO:1903281 GO:0016324 GO:0071468 GO:0071436 GO:0086092 GO:0005887 GO:0090533 GO:0006883 GO:0070886 GO:0005516 GO:0048306 GO:0051259 GO:0098719 GO:0035994 GO:0070062 GO:0005654 GO:0055007 GO:0005925 GO:0015385 GO:0071872 GO:0051453 GO:0010613 GO:1902600 GO:0045121 GO:0045944 GO:0030346 GO:0098735 GO:0005789

XM_005700147.3 GO:0006357 GO:0003713 GO:0008134 GO:0044212 GO:0035035 GO:0005669 GO:0051123

XM_005691844.3 GO:0005654 GO:0005730 GO:0005794 GO:0008270 GO:0016021 GO:0018230 GO:0019706

XM_018063529.1 GO:0046951 GO:0006642 GO:0000038 GO:0004467 GO:0001676 GO:0009925 GO:0015245 GO:0030176 GO:0031957 GO:0015911 GO:0006699

XM_018059727.1 GO:0086016 GO:0086006 GO:0086012 GO:0086091 GO:0010765 GO:0086002 GO:0060078 GO:0044325 GO:2000649 GO:0017080 GO:0001518 GO:0014704 GO:0060307

XM_018063768.1 GO:0070062 GO:0030216 GO:0030674 GO:0045111 GO:0001533 GO:0019215 GO:0005737 GO:0018149 GO:0005198

XM_013974048.2 GO:0097502 GO:0006506 GO:0031501 GO:0004584 GO:0016021 GO:0005789

XM_005682257.3 GO:0006406 GO:0016020 GO:0010468 GO:0000398 GO:0044822 GO:0005524 GO:0005737 GO:0005681 GO:0004004 GO:0010501 GO:0006974

XM_018040249.1 GO:0000132 GO:0016020 GO:0007059 GO:0051298 GO:0051303 GO:0007100 GO:0007405 GO:0051642 GO:0021987 GO:0045202 GO:0047496 GO:0005871 GO:0042802 GO:0008017 GO:0007020 GO:0001764 GO:0000776 GO:0031616

XM_005693703.3 GO:0005856 GO:1901098 GO:0034341 GO:0031410 GO:0042803 GO:0048471 GO:0000421

NM_001285609.1 GO:0006879 GO:0006826 GO:0004322 GO:0006955 GO:0008199 GO:0005739 GO:0055114 GO:0008285

XM_005695796.3 GO:0070062 GO:0030036 GO:0044822 GO:0005925 GO:0005903 GO:0003382 GO:0042802 GO:0005737 GO:0003334 GO:0003779 GO:0001725 GO:0007519 GO:0005886

XR_001918100.1 GO:0071361 GO:0030658 GO:0030667 GO:0005938 GO:0005791 GO:0071320 GO:0001666 GO:0035091 GO:0043235 GO:0005220 GO:0048016 GO:0016021 GO:0051209 GO:0033017 GO:0005886

XM_018065383.1 GO:0005654 GO:0007165 GO:0015914 GO:0008526 GO:0005737

XM_018058858.1 GO:0016021 GO:0031966 GO:0051881 GO:0001666 GO:0006839

XM_013963311.2 GO:2001028 GO:0007169 GO:0005524 GO:0016021 GO:0050918 GO:0071526 GO:0018108 GO:0004714

XM_018039104.1 GO:0042327 GO:0005737

XM_018062793.1 GO:0005634 GO:0044822 GO:0005737 GO:0051001

XM_018062709.1 GO:0007155 GO:0001669 GO:0005886 GO:0031225

XM_005679400.3 GO:0005654 GO:0009267 GO:0045921 GO:0016023 GO:0016082 GO:0005829 GO:0042734 GO:0045211 GO:1990504

XM_018052916.1 GO:0016021

XM_018061226.1 GO:0007095 GO:0005524 GO:0004674 GO:0046329 GO:0005622 GO:0043507 GO:0046777

XM_013967000.2 GO:0003677 GO:0071339 GO:0006355 GO:0003700 GO:0005737 GO:0046983

XM_018065304.1 GO:0005654 GO:0019903 GO:0005813 GO:0070979 GO:0005737 GO:0007091 GO:0005680 GO:0005876

XM_018047748.1 GO:0042552 GO:0035725 GO:0005524 GO:0043194 GO:0034765 GO:0016023 GO:0060078 GO:0019228 GO:0030018 GO:0086010 GO:0001518 GO:0005248 GO:0007422 GO:0033268

XM_005699663.2 GO:0006508 GO:0004190

XM_005684254.3 GO:0010390 GO:0042393 GO:0031062 GO:0031625 GO:0003730 GO:2001168 GO:0045893 GO:0016874 GO:0006511 GO:0005730 GO:0003682 GO:0008270 GO:0016568 GO:0000209 GO:0005654 GO:0033503 GO:0002039 GO:0030336 GO:0004842 GO:1900364 GO:0003713

XR_001917012.1 GO:0046872 GO:0016787 GO:0043234 GO:0016021 GO:0003676

XM_018061078.1 GO:0018117 GO:0016021 GO:0070733

XM_005676572.3 GO:0044805 GO:0031410 GO:0031902 GO:0005802 GO:0000422 GO:0034727 GO:0034497 GO:0016021 GO:0000407 GO:0015031 GO:0055037 GO:0000421

XM_005692956.3 GO:0005886

XM_005677376.3 GO:0045892 GO:0003677 GO:0003714 GO:0030183

XM_018058961.1 GO:0016567 GO:0031463 GO:0004842

XM_018050512.1 GO:0070534 GO:0042059 GO:0070936 GO:0061630 GO:0005634 GO:0006513 GO:0042127 GO:0042147 GO:0005154 GO:0042787 GO:0016874 GO:0071629 GO:0005829 GO:0008270 GO:0043162

XM_005685699.3 GO:0042795 GO:0046982 GO:0001103 GO:0030054 GO:0016032 GO:0042803 GO:0006368 GO:0005672 GO:0006413 GO:0003743 GO:0051091 GO:0003713 GO:0045944 GO:0017025 GO:0051123

XM_005684274.3 GO:0009791 GO:0030901 GO:0021794 GO:0035264 GO:0046983

XM_018054383.1 GO:0005634 GO:0005615 GO:0005524 GO:0035869 GO:0008360 GO:0042384 GO:0005814 GO:0004674 GO:0018105 GO:0005829

XM_005694862.3 GO:0006457 GO:0005524 GO:0051082 GO:0005737

XM_018057882.1 GO:0005665 GO:0001221 GO:0005524 GO:0036091 GO:0008094 GO:0003676 GO:0008026

XM_018053525.1 GO:0005654

XM_018059129.1 GO:0050731 GO:0001822 GO:0031594 GO:0042733 GO:0090090 GO:0016021 GO:0051124 GO:0005886 GO:0097105 GO:0009953 GO:0097104 GO:0030971 GO:0030509 GO:0042803 GO:0042475 GO:1901631 GO:0009986 GO:0030279 GO:0005509 GO:0009954 GO:0050771 GO:0001942

XM_013962622.2 GO:0061087 GO:0035064 GO:0005737 GO:0035098 GO:0048863 GO:0000122 GO:0061086 GO:0019827 GO:0045944 GO:0008270 GO:0007379

XM_013975299.2 GO:0018345 GO:0008270 GO:0016021 GO:0005789 GO:0019706

XM_013969576.2 GO:0002860 GO:0016021 GO:0050715 GO:0008037 GO:0005102 GO:0002355 GO:0005886

XM_005691175.3 GO:0097027 GO:0031625 GO:0051443 GO:0019005 GO:0034644 GO:2000060 GO:2000639 GO:0000209 GO:0030332 GO:0005654 GO:0042802 GO:0016032 GO:1902806 GO:0055088 GO:0010868 GO:0030674 GO:2000346 GO:0031146 GO:1901800 GO:0001944 GO:0005730 GO:0005829 GO:0016021 GO:0006974 GO:0050816 GO:1903378 GO:0045741 GO:0010883 GO:1903146 GO:1990452 GO:0004842 GO:0007062 GO:0045746 GO:0032876 GO:0070374 GO:0050821

XM_018065399.1 GO:0061630 GO:0010792 GO:0032436 GO:0005521 GO:0016874 GO:0031624 GO:0019901 GO:0005638 GO:0035861 GO:0003697 GO:0016055 GO:0008270 GO:0000724 GO:0000209

XM_018049831.1 GO:0016605 GO:0090169 GO:0042802 GO:0090234 GO:0005737 GO:0043161 GO:0045944 GO:0008270 GO:0046685 GO:0008134

XR_001917952.1 GO:0016477 GO:0007010 GO:0008360 GO:0005737

XM_018057252.1 GO:0007165

XM_005684544.3 GO:0046872 GO:0008253 GO:0016311

XM_018051044.1 GO:0045859 GO:0006457 GO:0070062 GO:0005524 GO:0051087 GO:0010608 GO:0019901 GO:0051879 GO:0060334 GO:0051301 GO:0060338 GO:0005829 GO:0051082 GO:0019887 GO:1990565 GO:0050821

XM_005674953.3 GO:0007264 GO:0005622 GO:0043547 GO:0017124 GO:0030027 GO:0005096

XM_018054778.1 GO:0005654 GO:0031965 GO:0044822 GO:0002181 GO:0000027 GO:0022625 GO:0030687 GO:0006364 GO:0005730 GO:0003735 GO:0070180 GO:0000956

XM_005680737.3 GO:0071361 GO:0030658 GO:0030667 GO:0005938 GO:0005791 GO:0071320 GO:0001666 GO:0035091 GO:0043235 GO:0005220 GO:0048016 GO:0016021 GO:0051209 GO:0033017 GO:0005886

XM_005678729.3 GO:0005922 GO:0060713 GO:0007154 GO:0016021 GO:0060708 GO:0060707

XM_018061227.1 GO:0007399 GO:0003729 GO:0005634 GO:0030154 GO:0007605 GO:0000381

XM_005692360.3 GO:0046872 GO:0006355 GO:0005622 GO:0003676

XM_013977034.2 GO:0003779 GO:0070062 GO:0001725 GO:0005737

XM_005684140.3 GO:0006281 GO:0005829 GO:0043240

XR_001917460.1 GO:0005524 GO:0016525 GO:0001701 GO:0030336 GO:0032526 GO:0045026 GO:0016021 GO:0005886 GO:0004714 GO:0018108

XM_018056005.1 GO:0044822

XM_018058039.1 GO:0043231 GO:0034765 GO:0071805 GO:0051260 GO:0005251 GO:0008076

XM_005697627.3 GO:0004896 GO:0016021 GO:0019221

XM_018063812.1 GO:0008593 GO:0005654 GO:0032878 GO:0030866 GO:0043547 GO:0051294 GO:0017157 GO:0030864 GO:0022008 GO:0050708 GO:0030165 GO:0019905 GO:0005886 GO:0017137 GO:0005096

XM_005686033.3 GO:0007186 GO:0005834 GO:0043547 GO:0035556 GO:0004871 GO:0005737 GO:0005096

XM_013964362.2 GO:0044822 GO:0000166 GO:0060213 GO:0031047

XM_005680820.3 GO:0005634 GO:0045600 GO:0045893 GO:0046326 GO:0005515 GO:0043565 GO:0033138 GO:0003700 GO:0050821 GO:0046889

XM_005695231.3 GO:0006616 GO:0005515 GO:0005730 GO:0005829 GO:0008144 GO:0008312 GO:0042493 GO:0005786 GO:0044822 GO:0019003 GO:0016607 GO:0003924 GO:0006617 GO:0030942 GO:0005525 GO:0043021

XM_013974538.2 GO:0046872 GO:0005634 GO:0006974 GO:0003676

XM_005700173.3 GO:0070062

XM_013963032.2 GO:0005524

XM_005681420.2 GO:0003677 GO:0005654 GO:0005524 GO:0070933 GO:0004386 GO:0000729 GO:0035861 GO:0051304 GO:0043044 GO:0000018 GO:0000792 GO:0070932 GO:0043596 GO:0016568

XM_013975643.2 GO:0005791 GO:0006782 GO:0030054 GO:0016023 GO:0055114 GO:0004729 GO:0007631 GO:0031304 GO:0007218 GO:0045202

XM_018057938.1 GO:0010875 GO:0008289 GO:0030317 GO:0005615 GO:0010189 GO:0034375 GO:0006629

XM_018047069.1 GO:0070062 GO:0005886

XM_013973678.2 GO:0008270 GO:0003676

XM_018057048.1 GO:1903679 GO:0005634 GO:0044822 GO:0005829 GO:0043488 GO:1990247 GO:0000932

XM_005692653.3 GO:0003723 GO:0005654 GO:0005524 GO:0042802 GO:0043278 GO:0043123 GO:0043531 GO:0005829 GO:0043234 GO:0004721 GO:0006470 GO:0004871

XM_005687566.3 GO:0007059 GO:0051301 GO:0031110 GO:0000940 GO:0005737 GO:0007067 GO:0005876

XM_005697820.3 GO:0005634 GO:0090502 GO:0004526 GO:0008033 GO:0003676

XM_018053027.1 GO:0005634 GO:0003713 GO:0008022 GO:0045944 GO:0007519 GO:0005737

XM_018040931.1 GO:0005634 GO:2001014 GO:0000381 GO:0000166 GO:0050885 GO:0005737 GO:0003676

XM_018045783.1 GO:0046872 GO:0005622 GO:0043547 GO:0035556 GO:0030165 GO:0005096

XM_018046870.1 GO:0046872 GO:0044822 GO:0003950

XM_018043546.1 GO:0007346 GO:0043010 GO:0001822 GO:0060065 GO:0030099 GO:0007050 GO:0005737 GO:0045893 GO:0000122 GO:0055123 GO:0060669 GO:0071514 GO:0005634 GO:0016301 GO:0071901 GO:0035264 GO:0007568 GO:0004861 GO:0030325 GO:0050680 GO:0042551 GO:0030511 GO:0001501

XM_018062714.1 GO:0003376 GO:0005765 GO:0016310 GO:0046512 GO:0017050 GO:0005829 GO:0001568 GO:0007420 GO:0038036 GO:0008284

XM_018064489.1 GO:0019752 GO:0016831 GO:0030170

NM_001285773.1 GO:0005198 GO:0045095

XM_018040577.1 GO:0045022 GO:0001881 GO:0005524 GO:0005829 GO:0033572 GO:0043086 GO:0016021 GO:0005769 GO:0055037 GO:0004864 GO:0070853 GO:0048471 GO:0046777 GO:0018107 GO:0004674 GO:0018105 GO:0005794 GO:0032456

XM_018066982.1 GO:0006355

XM_018039788.1 GO:0086009 GO:0051117 GO:0042470 GO:0005391 GO:0030007 GO:1903288 GO:1901018 GO:0006883 GO:0070062 GO:0001671 GO:0072659 GO:1903278 GO:0005890 GO:0032781 GO:0050821 GO:0010248

XM_013966112.2 GO:0000287 GO:0046712 GO:0046057 GO:0044715 GO:0046067 GO:0044717 GO:0044716

XM_005695778.3 GO:0001558 GO:0005634 GO:0043005

XM_005684537.3 GO:0016021

NM_001287034.1 GO:0016020 GO:0008083 GO:0008201

XM_018052160.1 GO:0016020 GO:0010951 GO:0008191 GO:0030198 GO:0030336 GO:0001955 GO:0007566 GO:0035115

XM_018047117.1 GO:0007186 GO:0016021 GO:0007166 GO:0004999 GO:0043235

NM_001285681.1 GO:0008083 GO:0005615 GO:0009897 GO:0010633 GO:0045064 GO:0030890 GO:0045348 GO:1901741 GO:1903660 GO:0042104 GO:0045892 GO:0005125 GO:2000352 GO:2000320 GO:0048304 GO:0045944 GO:0002227 GO:0043011 GO:0048295 GO:0045671 GO:0097192 GO:0031296 GO:0008203 GO:0070351 GO:2001237 GO:0051091 GO:0005136 GO:0045582 GO:0002296 GO:0042832 GO:0043306 GO:0035745 GO:0042523 GO:0045080 GO:0032736

XM_005692224.3 GO:0046872 GO:0006355 GO:0005622 GO:0003676

XM_005684320.3 GO:0016607 GO:0015030 GO:0071001

XM_005676275.3 GO:0016021 GO:0030863

XM_018057071.1 GO:0050731 GO:0005615 GO:0048008 GO:0016358 GO:0021785 GO:0021637 GO:0048012 GO:0060978 GO:0021828 GO:0017154 GO:1901998 GO:0061441 GO:0048842 GO:1902336 GO:0005769 GO:0061549 GO:0035729 GO:0005925 GO:0048843 GO:0043524 GO:0097490 GO:1901166 GO:0061299 GO:1902378 GO:1902946 GO:0005883 GO:0001569 GO:0050918 GO:0021612 GO:0043049 GO:0035767 GO:0038085 GO:0097491 GO:0021649 GO:0061551 GO:0097374 GO:0002042 GO:0060666 GO:2001237 GO:0097443 GO:0005829 GO:0016021 GO:0071679 GO:0005886 GO:0007413 GO:0036486 GO:1903375 GO:0030424 GO:1902287 GO:0060982 GO:0005021 GO:0046872 GO:0060385 GO:0048010 GO:0070374

XM_005698034.3 GO:0034618 GO:0042802 GO:0005829

XM_018062080.1 GO:0070062 GO:0030144 GO:0005794 GO:0006487 GO:0016021

XM_013969894.2 GO:0005814 GO:0036064 GO:0006508 GO:0005829 GO:0008270 GO:0004181 GO:0035610

XM_018061897.1 GO:0043154 GO:0005615 GO:0035690 GO:0043027 GO:0016324 GO:0016999 GO:0031225 GO:0008239 GO:0071277 GO:0008270 GO:0070062 GO:0071732 GO:0072341 GO:0030336 GO:0034235 GO:0070573 GO:0050667 GO:0031528

XM_005679431.2 GO:0060076 GO:0016021 GO:0043197 GO:0097119 GO:0050807 GO:0045211

XM_018060231.1 GO:0016020 GO:0000165 GO:0005634 GO:0045638 GO:0007166 GO:0008440 GO:0046638 GO:0001932 GO:0035726 GO:0046579 GO:0005829 GO:0045059 GO:0033030

XM_018044029.1 GO:0000462 GO:0005634

XM_018041158.1 GO:0015693 GO:0016323 GO:0010960 GO:0016021

XM_018047044.1 GO:0060837 GO:0001701 GO:0001885 GO:0005737 GO:0035264 GO:0045216 GO:0061154 GO:0043234 GO:0048839 GO:0048845 GO:0060039 GO:0001570

XM_018054166.1 GO:0030215 GO:0014912 GO:0016021 GO:0021591 GO:0014911

XM_005684273.3 GO:0035269 GO:0005634 GO:0005783 GO:0046329 GO:0005794 GO:0016021 GO:0005801 GO:0008285

XM_018038491.1 GO:0005576

XR_001918906.1 GO:0005730 GO:0005737

XM_018066672.1 GO:0046330 GO:0005615 GO:0048103 GO:0048018 GO:0035659 GO:0010595 GO:0035019 GO:0022009 GO:0043627 GO:0005109 GO:0071560 GO:0007269 GO:0060997 GO:0032355 GO:0048864 GO:0001502 GO:0000578 GO:0061038 GO:0035567 GO:0070062 GO:0008105 GO:0009953 GO:0001525 GO:0005125 GO:0035116 GO:0043066 GO:0031133 GO:2000463 GO:0090263 GO:0030010 GO:0002062 GO:0007548 GO:0014834 GO:0045944 GO:0005788 GO:0036465 GO:0030666 GO:0005796 GO:0042733 GO:0014719 GO:0005829 GO:0070307 GO:0060066 GO:0060021 GO:0005886 GO:0060054 GO:0045165 GO:0050768 GO:1902474 GO:0035115 GO:0021707 GO:0005578 GO:0021846 GO:0009986

XM_018048671.1 GO:0003677 GO:0005634 GO:0010792 GO:0034599 GO:0042802 GO:0043234 GO:2000819 GO:0000730

XM_005691428.2 GO:0070588 GO:0010882 GO:0005524 GO:0090534 GO:0014801 GO:0033292 GO:0045822 GO:0005887 GO:0055119 GO:0006984 GO:1903515 GO:0014704 GO:0033017 GO:0042493 GO:0014883 GO:0005388 GO:0097470 GO:0006996 GO:0070296 GO:0002026 GO:1903233 GO:0032470 GO:0044548 GO:0008022 GO:0034599 GO:0005509 GO:0019899

XM_005680747.2 GO:0015934 GO:0006412 GO:0003735

XM_018040008.1 GO:0007186 GO:0005887 GO:0004930

XM_018060948.1 GO:0000978 GO:0005654 GO:0001077 GO:0007283 GO:0008584 GO:0046872 GO:0003682 GO:0045944 GO:0030217

XM_018044425.1 GO:0005484 GO:0042147 GO:0000139 GO:0005768 GO:0005795 GO:0061025 GO:0005829 GO:0016021 GO:2000156 GO:0015031

XM_005678121.3 GO:0016021

XM_013964365.2 GO:0070062 GO:0003094 GO:0004177 GO:0016485 GO:0005739 GO:0030145

XM_005678679.3 GO:0010369 GO:0051233 GO:0000775 GO:0032133 GO:0030496 GO:0045171 GO:0005730 GO:0007080

XM_018039983.1 GO:0007160 GO:0009986 GO:0016324 GO:0007605 GO:0019226

XM_018042984.1 GO:0050995 GO:0019915 GO:0045444 GO:0030176 GO:0034389

XM_018065816.1 GO:0005634 GO:0005524 GO:0004674 GO:0060416 GO:1990418 GO:0006468 GO:0005737 GO:1990314

XM_018051273.1 GO:0031965 GO:0006607 GO:0008565 GO:0000060 GO:0005737 GO:0008139 GO:0034399 GO:0008536 GO:0006610 GO:0000059

XM_005700756.3 GO:0019898 GO:0032266 GO:0000422 GO:0080025 GO:0034497 GO:0006497 GO:0005829 GO:0044804 GO:0034045

XM_005690895.3 GO:0046872 GO:0003676

XM_005681604.3 GO:0005768 GO:0005743

XR_001918202.1 GO:0008418 GO:0007613 GO:0005634 GO:0008344 GO:0005737

XM_018047681.1 GO:1901970 GO:0072686 GO:0005634 GO:0045143 GO:0005813 GO:0005737 GO:0000212 GO:0006508 GO:0004197 GO:0051307

NM_001314344.1 GO:0032720 GO:0032496 GO:0007283 GO:0001162 GO:0005737 GO:0001892 GO:0000122 GO:0060136 GO:0000979 GO:0003682 GO:0008285 GO:0005654 GO:0000978 GO:0006468 GO:0009299 GO:0034605 GO:0001078 GO:0045944 GO:0043234 GO:0007143 GO:0040018 GO:0045120

XM_018043291.1 GO:0046872 GO:0003676

XM_013969899.2 GO:0005654 GO:0030429 GO:0006569 GO:0097053 GO:0042803 GO:0043420 GO:0034341 GO:0005829 GO:0034516 GO:0034354 GO:0005739 GO:0030170 GO:0019805

XM_018062999.1 GO:0006355 GO:0005622 GO:0003676

XM_005675423.3 GO:0043484 GO:0010494 GO:0046872 GO:0005654 GO:0044822 GO:0005813 GO:0003725

XR_001918763.1 GO:0070062 GO:0042995 GO:0031346 GO:0048550 GO:0071914 GO:0009986 GO:0043087 GO:0001934 GO:0016021 GO:2001287 GO:0005886

XM_018053939.1 GO:0030317 GO:0018095 GO:0009566 GO:0007288 GO:0005813

XM_013975420.2 GO:0043022 GO:0016021

XM_005684823.3 GO:0046872 GO:0008253 GO:0000166 GO:0016311 GO:0031225 GO:0005886 GO:0009166

XM_005700225.3 GO:0007528 GO:0005605 GO:0005201 GO:0005587 GO:0031594

XM_005699500.2 GO:0006810 GO:0009986 GO:0016021 GO:0005215

XM_018054167.1 GO:0030215 GO:0014912 GO:0016021 GO:0021591 GO:0014911

XM_018062023.1 GO:0070062 GO:0016324 GO:0005829 GO:0016021 GO:0006814 GO:0015377 GO:1902476

XM_018038705.1 GO:0005654 GO:0005737

XM_018053302.1 GO:0070062 GO:0001954 GO:0005654 GO:0031594 GO:0017166 GO:0005737 GO:0019901 GO:0003779 GO:0005178 GO:0008270 GO:0042383 GO:0031527 GO:0016010 GO:0070938

XM_005683019.3 GO:0016021

XM_013967524.2 GO:0005783 GO:0005794 GO:0016021 GO:0005886

XM_018050335.1 GO:0007165 GO:0002230

XM_018059605.1 GO:0090136 GO:0045218 GO:0070062 GO:0005634 GO:0005813 GO:0005915 GO:0070097 GO:0005737

XM_018059142.1 GO:0070062 GO:0007264 GO:0016021 GO:0043547 GO:0005737 GO:0005096

XM_018059607.1 GO:0090136 GO:0045218 GO:0070062 GO:0005634 GO:0005813 GO:0005915 GO:0070097 GO:0005737

XM_005695650.3 GO:0005938 GO:0070905 GO:0043025 GO:0005887 GO:0042734 GO:0048306 GO:0032279 GO:0051966 GO:0001662 GO:0030534 GO:0070588 GO:0048786 GO:0005791 GO:0001642 GO:0005245 GO:0019226 GO:0043235 GO:0007194 GO:0010855 GO:0005246 GO:0007196 GO:0005516 GO:0030165 GO:0007614 GO:0045211 GO:0014050 GO:0007608 GO:0001661 GO:0042803 GO:0043195 GO:0043198 GO:0016595 GO:0009986 GO:0005794 GO:0005509 GO:0007605

XM_018060950.1 GO:0005634 GO:0005524 GO:0006468 GO:0046982 GO:0007283 GO:0005737 GO:0005801 GO:0004672 GO:0008270

XM_018056315.1 GO:0000166 GO:0003676

XM_018039066.1 GO:0060914 GO:0043409 GO:0035914 GO:0022008 GO:0005639 GO:0051898 GO:0006998

XM_018066017.1 GO:0006357 GO:0003677 GO:0046872 GO:0005634 GO:0003712

XM_013965630.2 GO:0007156 GO:0005509 GO:0008333 GO:0016021 GO:0005622 GO:0060989 GO:0005886

XM_018039206.1 GO:0016021

XM_005678067.3 GO:0016021

XM_018044332.1 GO:0016021

XM_005693223.3 GO:0003677 GO:0005874 GO:0006355 GO:0007094 GO:0005737 GO:0005730 GO:0008201 GO:0005819 GO:0008270 GO:0000777 GO:0003700 GO:0044822 GO:0008017 GO:0008608 GO:1990047 GO:0090307 GO:0051301 GO:0046785 GO:0001578 GO:0050821

XM_005688662.3 GO:0030054 GO:0051117 GO:0005737 GO:0050998 GO:0017080 GO:0005516 GO:0060307 GO:0005856 GO:0086005 GO:0002027 GO:0003779 GO:0044325 GO:1902083 GO:0043234 GO:1902305 GO:0042383 GO:0005198

XM_005675984.2 GO:0006396 GO:0000784 GO:0044822 GO:0000166 GO:0030529

XM_018059430.1 GO:0006506 GO:0005634 GO:0008565 GO:0042771 GO:0000139 GO:1902230 GO:0042770 GO:0016021 GO:0005789 GO:0015031

XR_001918334.1 GO:0008270 GO:0003676

XM_018057945.1 GO:0040029 GO:0048854 GO:0050671 GO:0051569 GO:0021895 GO:0001701 GO:0035097 GO:0046872 GO:0000979 GO:0048812 GO:0080182 GO:0002052

XM_018063074.1 GO:0016020 GO:0070062 GO:0030425 GO:0044822 GO:0022625 GO:1903146 GO:0044297 GO:0006412 GO:0036464 GO:0003735

XM_018055186.1 GO:2000273 GO:0010763 GO:0030546 GO:0005783 GO:2001031 GO:0050730 GO:0035669 GO:0035276 GO:0031663 GO:0032467 GO:0071361 GO:0005856 GO:0035641 GO:0003785 GO:0050996 GO:0048471 GO:0090303 GO:0018105 GO:0032230 GO:0071380 GO:0008047 GO:0071456 GO:0031397 GO:0032024 GO:0005524 GO:0070257 GO:0043278 GO:0051279 GO:0071889 GO:0010634 GO:0061178 GO:0005829 GO:0002281 GO:0005739 GO:0030838 GO:0010811 GO:0005886 GO:0043085 GO:0005634 GO:0043123 GO:0046872 GO:0004699 GO:0005794 GO:0043410 GO:0019899

XM_018055120.1 GO:0030331 GO:0070016 GO:0005923 GO:0016055 GO:0000159 GO:0051721 GO:0032403 GO:0008285

XM_018039502.1 GO:0016925 GO:0003677 GO:0061665 GO:0016605 GO:0043433 GO:0031625 GO:0060766 GO:0045944 GO:0008270 GO:0008134 GO:0045667

NM_001314229.1 GO:0070062 GO:0032024 GO:0005615 GO:0001654 GO:0042572 GO:0006094 GO:0032526 GO:0042593 GO:0019841 GO:0016918 GO:0005215 GO:0030277

XM_005692184.3 GO:0044822 GO:0006355 GO:0030490 GO:0034455

XM_005690396.3 GO:0016021

XM_018038865.1 GO:0001952 GO:0005615 GO:0038083 GO:0005524 GO:0014909 GO:0060444 GO:0007566 GO:0043235 GO:0005887 GO:0044319 GO:0008285 GO:0070062 GO:0001558 GO:0043583 GO:0010715 GO:0038062 GO:0038063 GO:0060749 GO:0005518 GO:0061302

XM_005697517.3 GO:0070534 GO:0070936 GO:0089720 GO:0061630 GO:0005783 GO:0030674 GO:0043066 GO:0042787 GO:0043161 GO:0051865 GO:0016021 GO:0008270

XM_018046012.1 GO:0046872 GO:0016787

XM_013965725.2 GO:0042383 GO:0016021 GO:0016012

XM_005690788.3 GO:0072562 GO:0016787 GO:0016180 GO:0032039 GO:0005737

XM_005697557.3 GO:0008152 GO:0003824

XM_005685922.3 GO:0006986 GO:0030433 GO:0031625 GO:0016021 GO:0005789

XM_018063557.1 GO:0042130 GO:0008270 GO:0002643

XM_018066106.1 GO:0038083 GO:0005524 GO:0031410 GO:0042127 GO:0031116 GO:0051297 GO:0007173 GO:0031234 GO:0043304 GO:0006935 GO:0045087 GO:0030155 GO:2000251 GO:0005925 GO:0004715 GO:0015630 GO:0010976 GO:2000145 GO:0008017 GO:0008360 GO:0045639 GO:0035091 GO:0034987 GO:0001578

XM_018065506.1 GO:0000124 GO:0006357 GO:0044822 GO:0003712 GO:0016573 GO:1903146

XM_018047015.1 GO:0005524 GO:0004683 GO:0005516 GO:0042803 GO:0046777

XR_310286.3 GO:0016020

XM_018054083.1 GO:0060252 GO:0010744 GO:0005524 GO:2000810 GO:0050861 GO:0035556 GO:0005911 GO:0005829 GO:0017160 GO:0005886 GO:0070062 GO:0051092 GO:0006468 GO:0045618 GO:0004697 GO:0034351 GO:0046872

XM_018045955.1 GO:0016021 GO:0005737

XM_018058560.1 GO:0031434 GO:0005524 GO:0043433 GO:0031665 GO:0031625 GO:0032436 GO:0043405 GO:0005737 GO:0004860 GO:0045645 GO:0004672 GO:0045651 GO:0055106 GO:0048662 GO:0005634 GO:0007254 GO:0014912 GO:0008134 GO:0045659

XM_018043381.1 GO:0009952 GO:0045666 GO:0051057 GO:0043497 GO:0005887 GO:0021987 GO:0007224 GO:0002088 GO:0014816 GO:0007520 GO:0060059 GO:2000179 GO:0010172 GO:0001708 GO:0048643 GO:0045944 GO:0043410 GO:0045663

XM_005696545.3 GO:0070534 GO:0070936 GO:2000785 GO:0071712 GO:0004842 GO:0010507 GO:0016021 GO:0008270 GO:0031648 GO:0009617

XM_018046455.1 GO:0080008 GO:0005634 GO:0005925 GO:0045944 GO:0030374 GO:0005737

XM_018046283.1 GO:0005634 GO:0042826 GO:0031625 GO:0070182 GO:0005737 GO:0035303 GO:0032204 GO:0051721

XR_001919783.1 GO:0006457 GO:0016272 GO:0051131 GO:0051082 GO:0051087

XM_018049267.1 GO:0046873 GO:0031090 GO:0016021 GO:0006829 GO:0005886 GO:0070574

XM_018066337.1 GO:0016020 GO:0005524 GO:0031072 GO:0051087 GO:0009408 GO:0046872 GO:0005829 GO:0051082 GO:0090084 GO:0042026

XM_018064936.1 GO:0005634 GO:0006355 GO:0043565

XM_018041463.1 GO:0008135 GO:0005634 GO:0003730 GO:0005737 GO:0071230 GO:0043005 GO:0000122 GO:0061158 GO:0030014 GO:0000166 GO:0060213

XM_018062428.1 GO:0016020 GO:0002028 GO:0019226 GO:0043194 GO:0010459 GO:0033135 GO:0030506 GO:0030507 GO:0070062 GO:0061337 GO:0016605 GO:0009566 GO:0043203 GO:0070852 GO:0016363 GO:0005200 GO:0045162 GO:0005543 GO:0003779 GO:0008091 GO:0033270 GO:0007628 GO:0019902 GO:0007605 GO:0021952 GO:0040018 GO:0034613

XM_018050396.1 GO:0008023 GO:0035327 GO:0007286

XM_005685483.2 GO:0034501 GO:0005524 GO:0004672 GO:0000778 GO:0006468 GO:0000940 GO:0007094 GO:0048471

XM_013974619.2 GO:0003723

XM_005676485.3 GO:0006936 GO:0005509 GO:0030016

XM_018050896.1 GO:0021549 GO:0070062 GO:0090557 GO:0021766 GO:0035335 GO:0021510 GO:0004725 GO:0021987 GO:0016021 GO:0022038

XM_018039278.1 GO:0070062 GO:0043025 GO:0043005

XM_005679935.3 GO:0005198 GO:0045095

XM_005685130.3 GO:0005783 GO:0005524 GO:0004728 GO:0060502 GO:0030216 GO:0060324 GO:0047485 GO:0043539 GO:0010629 GO:0007050 GO:0070371 GO:0060440 GO:0060711 GO:0030878 GO:0090398 GO:0048870 GO:0060020 GO:0005886 GO:0060674 GO:0008285 GO:0070062 GO:0000187 GO:0021697 GO:0007507 GO:0048538 GO:0050772 GO:0010628 GO:0008022 GO:0004674 GO:0004708 GO:0048679

XM_005684768.3 GO:1902176 GO:0070062 GO:0033089 GO:0002526 GO:0017159 GO:0006807 GO:0002544 GO:0015939 GO:0045087

XM_018066392.1 GO:0016020 GO:0003723 GO:0005847 GO:0006379 GO:0006398 GO:0006378

XM_005676246.3 GO:0016020 GO:0044822 GO:0005524 GO:0005730 GO:0004386

XM_018040654.1 GO:0016477 GO:0045861 GO:0005770 GO:0031410 GO:0051480 GO:0035235 GO:0097352 GO:0016485 GO:0005829 GO:0006897 GO:0043086 GO:0016021 GO:0001934 GO:0048306 GO:0005776 GO:0005769 GO:0051489 GO:0007040 GO:0005765 GO:0072659 GO:0030036 GO:0006520 GO:0043524 GO:0042133 GO:0006865 GO:0001508 GO:0008306 GO:0050885 GO:0016242

XM_005682090.3 GO:0007507

XM_018049292.1 GO:0008270 GO:0055114 GO:0016491

XR_001918227.1 GO:0016020 GO:0030692 GO:0044822 GO:0032040 GO:0005730 GO:0030490 GO:0019899

XM_018058015.1 GO:0003836 GO:0018279 GO:0071354 GO:0052798 GO:0030173 GO:0009311 GO:0097503 GO:0001574

XM_018059329.1 GO:0097680 GO:0060627 GO:0000149 GO:0005783 GO:0007059 GO:0005770 GO:0030496 GO:0051297 GO:0045335 GO:0007051 GO:0032465 GO:0051684 GO:0017124 GO:0005769 GO:0071900 GO:0035493 GO:0032801 GO:0005813 GO:0006890 GO:0005764 GO:0010508 GO:0043234 GO:0070418 GO:0046718

XM_018056908.1 GO:0046330 GO:0040019 GO:0080154 GO:0060466 GO:0070498 GO:0005521 GO:2000560 GO:0021987 GO:0004871 GO:0007215 GO:0045892 GO:0070062 GO:0031965 GO:0007213 GO:0043209 GO:0016607 GO:0000790 GO:0005546 GO:0045663 GO:0016042 GO:0048639 GO:0048009 GO:0043547 GO:0045893 GO:0008277 GO:0032735 GO:0005829 GO:0005516 GO:0035722 GO:1900087 GO:0004435 GO:2000344 GO:2000438 GO:0007613 GO:0035723 GO:0005509 GO:0045444 GO:0000086 GO:0019899 GO:0005096

XM_005676117.3 GO:0006486 GO:0030246 GO:0004653 GO:0016021 GO:0000139

XM_005683853.3 GO:0005634 GO:0045944 GO:0008017 GO:0005737 GO:0032092

XM_018039566.1 GO:0097352 GO:0032456 GO:0005737

XM_005677916.3 GO:0042974 GO:0006355

XM_005699807.3 GO:0055085 GO:0016021

XM_005685224.3 GO:0006355 GO:0003700 GO:0005737 GO:0001047

XM_018043369.1 GO:0016021 GO:0005739

XM_005680302.3 GO:0046872 GO:0042802 GO:0005622 GO:0035556 GO:0003009 GO:0048741 GO:0007274

XM_005695003.3 GO:0015389 GO:0072531 GO:0005887 GO:1901642 GO:0015855

XM_018059074.1 GO:0003677 GO:0005634 GO:0044822 GO:0003682 GO:0005737

XM_018061545.1 GO:0045892 GO:0006357 GO:0005654 GO:0043565 GO:0003700 GO:0005737 GO:0045893

XR_001918823.1 GO:0070062 GO:0016020 GO:0005634 GO:0021503 GO:0060840 GO:0003281

XM_018062313.1 GO:0002244 GO:0005615 GO:0001654 GO:0003382 GO:0043547 GO:0090162 GO:0007010 GO:0051056 GO:0005096

XM_018058788.1 GO:0005634 GO:0006355 GO:0005737

XM_018044883.1 GO:0005856

XM_018056514.1 GO:0072687

XM_005692113.3 GO:0006470 GO:0005739 GO:0004722

XM_005687462.3 GO:0002244 GO:0007264 GO:0005622 GO:0005525

XM_018046354.1 GO:0070062 GO:0005634 GO:0035335 GO:0008138 GO:0004725 GO:0004722 GO:0005737

NM_001285615.1 GO:0004364 GO:0070062 GO:0031090 GO:0005635 GO:0004602 GO:0016021 GO:0005789

XM_005678034.3 GO:0017183 GO:0004164 GO:0032259

XM_018061367.1 GO:0008135 GO:0047485 GO:0070816 GO:0006289 GO:0000439 GO:0005675 GO:0008353 GO:0046872 GO:0003684 GO:0006412 GO:0008094

XM_018054690.1 GO:0032007 GO:0045859 GO:0001822 GO:0005938 GO:0043666 GO:0021766 GO:0047485 GO:0051087 GO:0017148 GO:0021987 GO:0050808 GO:0005886 GO:0008285 GO:0051492 GO:0043379 GO:0042552 GO:0006813 GO:0033596 GO:0055007 GO:0051894 GO:0006407 GO:0032868 GO:0030030 GO:0030027 GO:0002250 GO:0045792 GO:0005884 GO:0046323 GO:0001843 GO:0050821

XM_018053500.1 GO:0005634

XM_018051428.1 GO:0030324 GO:0048790 GO:0007274 GO:0003016 GO:0050975 GO:0051602 GO:0009791 GO:0007264 GO:0005829 GO:0016188 GO:0048172 GO:0031489 GO:0015031 GO:0005886 GO:0061670 GO:0031630 GO:0008021 GO:0001669 GO:1903307 GO:1903561 GO:0043195 GO:0007005 GO:0003924 GO:0007409 GO:0005768 GO:0005525

XM_018066327.1 GO:0000226 GO:0005874 GO:0002230 GO:0008017

XM_005699498.3 GO:0006884 GO:0005654 GO:0044822 GO:0046982 GO:0034709 GO:0034715 GO:0006821 GO:0005829 GO:0005886 GO:0000387

XM_005677633.3 GO:0005856 GO:0007165 GO:0008360 GO:0005737 GO:0005095 GO:0005198 GO:0034260 GO:0005886 GO:0006909

XR_311284.2 GO:0006810 GO:0005549 GO:0005215

XM_018046127.1 GO:0042834 GO:0009253 GO:0016019 GO:0016045 GO:0002221 GO:0008745 GO:0008270 GO:0050830

XM_018050730.1 GO:0016702 GO:0019511 GO:0005783 GO:0005634 GO:0005506 GO:0055114 GO:0031418 GO:0004656

XM_018059139.1 GO:0070062 GO:0007264 GO:0016021 GO:0005622 GO:0043547 GO:0005096

XM_018056549.1 GO:0043065 GO:0000381 GO:0006355 GO:0061574 GO:0016607 GO:0048025 GO:0005737

XM_018042280.1 GO:0007264 GO:0005622 GO:0005525

XM_018047854.1 GO:0070588 GO:0050852 GO:0005245 GO:0005891

XM_018044897.1 GO:0003743 GO:0005622 GO:0005525 GO:0001731 GO:0003924

XM_005696360.3 GO:0016020 GO:0003677 GO:0000398 GO:0006281 GO:0044822 GO:0006355 GO:0072422 GO:0000974 GO:0016607 GO:0005737 GO:0071013 GO:0005730 GO:0071987 GO:0005662

XM_018048543.1 GO:2000045 GO:0002244 GO:0035335 GO:0043407 GO:0051279 GO:0005737 GO:0019901 GO:0050860 GO:0042130 GO:0005730 GO:0030220 GO:0005911 GO:0033277 GO:0050732 GO:0017124 GO:0070062 GO:0045577 GO:0035855 GO:0042267 GO:0070372 GO:0002924 GO:0042105 GO:0005001 GO:0014068 GO:0042169 GO:0001784 GO:0050859 GO:0033630 GO:0070527 GO:0008284

XM_018056107.1 GO:0046855 GO:0004439 GO:0046856 GO:0005930 GO:0000139

XM_018062698.1 GO:0003341 GO:0036157 GO:0036158

XM_018044263.1 GO:0005747 GO:0016021 GO:0055114

XM_005675160.3 GO:0070062 GO:0005615 GO:0010951 GO:0008191 GO:0004869 GO:0007339

XM_018050613.1 GO:0030833 GO:0003779 GO:0008289 GO:0005856 GO:0005634 GO:0032233 GO:0005737

XM_018044850.1 GO:0072686 GO:0005827 GO:0006513 GO:0005813 GO:0007094 GO:0005737 GO:0004842 GO:0051301 GO:0031463

XM_018054900.1 GO:0070062 GO:0046872 GO:0016021 GO:0008454 GO:0006491 GO:0000139

XM_018051793.1 GO:0043154 GO:0016567 GO:0030027 GO:0016874 GO:0048471 GO:2001237 GO:0005829 GO:0005794 GO:0008270 GO:0046328

XM_018050380.1 GO:0060395 GO:0005654 GO:0008270

XM_018041987.1 GO:0008233 GO:0031090 GO:0016021 GO:0006465 GO:0005787

XM_018049556.1 GO:0070062 GO:0019209 GO:0005634 GO:0035329 GO:0016301 GO:0005737 GO:0046777 GO:0042327 GO:0019900

XM_005693035.2 GO:0046872 GO:0003676

XM_018066725.1 GO:0016020 GO:0005783 GO:0030220

XM_005677124.3 GO:0010763 GO:0005524 GO:0030199 GO:0030500 GO:0051091 GO:0005887 GO:0045860 GO:0018108 GO:0070062 GO:0005925 GO:0048146 GO:0038062 GO:0003416 GO:0090091 GO:0045669 GO:0046777 GO:0035988 GO:0038063 GO:0005518

XM_018039207.1 GO:0005783 GO:0071260 GO:0019835 GO:0002352 GO:0002262 GO:0010332 GO:0034644 GO:0044346 GO:1900103 GO:0033137 GO:0001783 GO:0031307 GO:0046902 GO:0032471 GO:0001974 GO:0008053 GO:0048597 GO:0070059 GO:0010524 GO:0001782 GO:0051726 GO:0010248 GO:0097192 GO:0009620 GO:0060068 GO:0046982 GO:0097202 GO:0070242 GO:0046930 GO:0043497 GO:0008283 GO:1902262 GO:0005829 GO:0043496 GO:0010046 GO:0001836 GO:0008630 GO:0035108 GO:0042803 GO:0044325 GO:0008635 GO:0051881

XM_018055157.1 GO:0016021

XM_018064828.1 GO:0051289 GO:0005887 GO:0005242 GO:0034765 GO:0010107

XM_005677436.3 GO:0050731 GO:0030833 GO:0051897 GO:0047485 GO:0005741 GO:0019966 GO:0016529 GO:0005667 GO:0015629 GO:0071345 GO:2000251 GO:0005758 GO:0014068 GO:0043066 GO:0030854 GO:0030027 GO:0045944 GO:0033138

XM_013970784.2 GO:0017089 GO:0070062 GO:0051861 GO:0046836 GO:0005737

XM_013966491.2 GO:0061351 GO:0003677 GO:0000784 GO:0005654 GO:0006260 GO:0005664

XM_005683637.3 GO:0034198 GO:0031625 GO:0046982 GO:1990130 GO:0007050 GO:0005829 GO:0051219 GO:0005765 GO:0045919 GO:0005634 GO:0042803 GO:0071230 GO:0006915 GO:0003924 GO:1990131 GO:0034448 GO:0005794 GO:0010507 GO:0034613 GO:0019048 GO:0005525 GO:0016236

XM_018058423.1 GO:0005654 GO:0014707 GO:0060539 GO:0046983 GO:0000122 GO:0000980 GO:0001206 GO:0060021

XM_018046734.1 GO:0005654 GO:0005525 GO:0070232 GO:0005737

XM_018041036.1 GO:0016310 GO:0007205 GO:0005524 GO:0046982 GO:0051260 GO:0035556 GO:0016023 GO:0042803 GO:0004143 GO:0010033 GO:0005886

XM_013963255.2 GO:0030425 GO:0005615 GO:0050795 GO:0021766 GO:0070326 GO:0005737 GO:0008236 GO:2000310 GO:0051968 GO:0038026 GO:0007411 GO:0097114 GO:0097120 GO:0061098 GO:0021511 GO:0014068 GO:2000969 GO:2000463 GO:0007155 GO:0006508 GO:0061003 GO:0097119 GO:0021800 GO:0051057 GO:0032008 GO:0007616 GO:1902078 GO:0021819 GO:0048265 GO:0005886 GO:0010001 GO:0004712 GO:1900273 GO:0021517 GO:0005578 GO:0046872 GO:0007626 GO:0032793 GO:0008306 GO:0090129

XM_018039337.1 GO:0035094 GO:0006582 GO:0006808 GO:0031625 GO:0008584 GO:0016248 GO:0002931 GO:0002320 GO:0048743 GO:0031965 GO:2001243 GO:0043209 GO:0043029 GO:0033138 GO:0005789 GO:0043375 GO:0010468 GO:0034097 GO:0046671 GO:0048873 GO:0030307 GO:0033077 GO:2000134 GO:0009636 GO:0010559 GO:0022898 GO:0045069 GO:0048536 GO:0043565 GO:0051384 GO:0048041 GO:0050853 GO:0001656 GO:0030308 GO:0007569 GO:0043583 GO:0051607 GO:0042803 GO:0033689 GO:2000811 GO:0010507 GO:0008134 GO:0048599 GO:0001952 GO:0014031 GO:0008625 GO:0010332 GO:0071310 GO:0030890 GO:0001658 GO:0007015 GO:0009791 GO:0051721 GO:0015267 GO:0032835 GO:0000209 GO:0035265 GO:0002326 GO:0031103 GO:0042493 GO:0007565 GO:0021747 GO:0046902 GO:0043524 GO:0010039 GO:0001541 GO:0001662 GO:0007409 GO:0018107 GO:0031069 GO:0070059 GO:2000378 GO:0032880 GO:0001782 GO:2001240 GO:0071456 GO:0032848 GO:0048753 GO:0051434 GO:0046982 GO:0046930 GO:0005741 GO:0042149 GO:0043497 GO:0048546 GO:0045636 GO:0003014 GO:0010523 GO:0014042 GO:0043496 GO:0005829 GO:0006470 GO:0033033 GO:0043085 GO:0014911 GO:0001836 GO:0008630 GO:0002020 GO:0008631 GO:0048538 GO:0030336 GO:0010224 GO:0042542 GO:0030279 GO:0031647 GO:0032469 GO:0040018 GO:0051881 GO:0022612

XM_018048032.1 GO:0044822

XM_005692150.3 GO:0005881

XR_001918834.1 GO:0003677 GO:0000791 GO:0006349 GO:0007283 GO:0005737 GO:0001741 GO:0000122 GO:0043046 GO:0003682 GO:0044027 GO:0090116 GO:0005720 GO:0005654 GO:0042802 GO:0016363 GO:0000775 GO:0071230 GO:0043045 GO:0006346 GO:0003886

XR_001919817.1 GO:0003779 GO:0017048 GO:0030036 GO:0090140 GO:0048471

XM_018041556.1 GO:0046872 GO:0005634 GO:0006355 GO:0003700

XM_005678506.1 GO:0016884 GO:0016021

XM_018049175.1 GO:0005634 GO:0006281 GO:0001205 GO:0001078 GO:0000122 GO:0000083 GO:0045944 GO:0005829 GO:0061418 GO:0000117 GO:0000980 GO:0001206

XM_018047563.1 GO:0005249 GO:0034765 GO:0071805 GO:0008076

XM_018065632.1 GO:0019800 GO:0030054 GO:0005509 GO:0030198 GO:0005614 GO:0005539 GO:0045202 GO:0010811 GO:0005604

XM_018065198.1 GO:0090051 GO:0000122 GO:0051091 GO:0000118 GO:0016568 GO:0001047 GO:0004407 GO:0040029 GO:0043393 GO:0010832 GO:0042826 GO:0070491 GO:0046872 GO:0005080 GO:0001025 GO:0005794 GO:0045944 GO:0016575

XM_018048624.1 GO:0005887 GO:0007166

XM_018047894.1 GO:0030424 GO:0015186 GO:0006868 GO:0016021 GO:0005886

XM_018050318.1 GO:0005634 GO:0000166 GO:0008270 GO:0003676

XM_018050706.1 GO:0005201 GO:0043010 GO:0030326 GO:0045669 GO:0005737 GO:0030501 GO:0035583 GO:0060346 GO:0001527 GO:0005509 GO:0048048 GO:0016021

XM_005684483.3 GO:0070062 GO:0005634 GO:0016787 GO:0000166 GO:0005737

XM_018067072.1 GO:0032868 GO:0015485 GO:0001666 GO:0045540 GO:0032933 GO:0000139 GO:0042304 GO:0016021 GO:0005789

XM_005680550.3 GO:0016757 GO:0005794 GO:0016021 GO:0000271

XM_005679489.3 GO:0070062 GO:0004619 GO:0006096

XM_018059268.1 GO:0016605 GO:0005524 GO:0009299 GO:0043066 GO:0005737 GO:0043508 GO:0018107 GO:0004674 GO:0018105

XM_013976686.2 GO:0051835 GO:0005856 GO:0043197 GO:0005546

XM_013966189.2 GO:0070052 GO:0030198 GO:0008201 GO:0005614 GO:0010811

XM_018041547.1 GO:0005759 GO:0055129 GO:0004587 GO:0042802 GO:0019544 GO:0034214 GO:0030170 GO:0010121

XM_018059380.1 GO:0007200 GO:0045028 GO:0035589 GO:0070257 GO:0016021 GO:0042312 GO:0005886

XM_018039797.1 GO:0005730

XM_018056748.1 GO:0045842 GO:0071466 GO:0071459 GO:0071922 GO:0031625 GO:0045445 GO:0071930 GO:0043353 GO:0045651 GO:0001102 GO:0001047 GO:0016605 GO:0042802 GO:0048667 GO:0035189 GO:0035914 GO:0045944 GO:0034088 GO:0034349 GO:0097284 GO:0051146 GO:0051402 GO:0007050 GO:0045879 GO:0031134 GO:2000134 GO:0000122 GO:0019900 GO:0005819 GO:0031175 GO:0051219 GO:0048565 GO:0006469 GO:0051301 GO:0050680 GO:0042551 GO:0007265 GO:0043550

XM_018054020.1 GO:0070588 GO:0071456 GO:0002244 GO:0035725 GO:0031594 GO:0005741 GO:0051560 GO:0005887 GO:0060291 GO:0042552 GO:0006851 GO:0005432 GO:0048709 GO:0007612 GO:0048471 GO:0014819 GO:0007613 GO:0042383 GO:0005789

XM_018058156.1 GO:0035094 GO:0005892 GO:0098655 GO:0030054 GO:0048630 GO:0007274 GO:0015464 GO:0042166 GO:0004889 GO:0050881 GO:0007271 GO:0042391 GO:0045211

XM_018049534.1 GO:0016504 GO:0070062 GO:0005518 GO:0008201 GO:0010952

XM_005679277.2 GO:0005634 GO:0048702 GO:0050795 GO:0006355 GO:0021599 GO:0090103 GO:0042473 GO:0050890 GO:0048844 GO:0007605 GO:0007634 GO:0043565 GO:0050905 GO:0060876

XM_005701849.3 GO:0070062 GO:0005634 GO:0005198 GO:0045095

XM_005676515.3 GO:0016021

XM_005676364.3 GO:0036462 GO:0071260 GO:0051603 GO:0097199 GO:0097202 GO:0031625 GO:0097342 GO:0097110 GO:0045651 GO:0005739 GO:0043124 GO:0035877 GO:0005654 GO:0005815 GO:0031265 GO:0043123 GO:0097194 GO:0034612 GO:0042981

XM_018042658.1 GO:0030578 GO:0000981 GO:0005654 GO:0045786 GO:0042802 GO:0010595 GO:0050728 GO:0046677 GO:0045765 GO:0045944 GO:0045648 GO:0005667 GO:0043565 GO:0042981

XM_018054244.1 GO:0007528 GO:0005938 GO:0031623 GO:0050815 GO:0016874 GO:0031698 GO:0070063 GO:2000650 GO:0048814 GO:0050807 GO:0042110 GO:0070062 GO:0006622 GO:0061630 GO:0050847 GO:0014068 GO:0003151 GO:0002250 GO:0048471 GO:0046824 GO:0048514 GO:0000151 GO:0043162 GO:0003197 GO:0042787 GO:0019089 GO:0010768 GO:0019871 GO:0030948 GO:0005829 GO:0005886 GO:0070064 GO:0050816 GO:0042391 GO:0032801 GO:0005634 GO:0006513 GO:0000785 GO:0042921 GO:0043130 GO:1901016 GO:0019904 GO:0045732 GO:0005794 GO:0044111

XM_018060734.1 GO:0005856 GO:1903506 GO:0005654 GO:0035335 GO:0030971 GO:0046825 GO:0004725 GO:0005737 GO:0003712 GO:0001946 GO:0008285

XM_018050116.1 GO:0005759 GO:0055129 GO:0004587 GO:0042802 GO:0019544 GO:0034214 GO:0030170 GO:0010121

XM_005685150.3 GO:0005509

XM_005695546.3 GO:0045773 GO:0070062 GO:0005654 GO:0043001 GO:0005794 GO:0000042 GO:0051020

XM_018048718.1 GO:0005654 GO:0055114 GO:0005737 GO:0003779 GO:0008270 GO:0030042 GO:0005886 GO:0016491 GO:0071949

XM_018050198.1 GO:0043402 GO:0000978 GO:0005815 GO:0005654 GO:0001077 GO:0042921 GO:0045944 GO:0005819 GO:0008270 GO:0005739 GO:0016568 GO:0038051 GO:1990239

XM_018057176.1 GO:0002011 GO:0007165

XM_005676292.3 GO:0005634 GO:0005524 GO:0004674 GO:0006468 GO:0035556 GO:2000271

XM_005686897.3 GO:0003677 GO:0000127 GO:0006383

XR_001919541.1 GO:0000978 GO:0005634 GO:0032728 GO:0045351 GO:0032727 GO:0051607 GO:0043330 GO:0042803 GO:0005737 GO:0060340 GO:0001078 GO:0000122 GO:0097300 GO:0031663

XM_018065715.1 GO:0006357 GO:0046872 GO:0000981 GO:0005634 GO:0003676

XM_018060512.1 GO:0043123 GO:0061630 GO:0000151 GO:0016567 GO:0008270 GO:0004871 GO:0005769

XM_005674830.2 GO:0050673 GO:0070062 GO:0043231 GO:0031012 GO:0035987 GO:0048593 GO:0005581 GO:0010811

XM_005695578.3 GO:0000149 GO:0005484 GO:0048280 GO:0031201 GO:0016021 GO:0005789 GO:0006888 GO:0015031 GO:0006887

XM_018044329.1 GO:0005230 GO:0007214 GO:0034220 GO:0030054 GO:0006821 GO:0016021 GO:0004890 GO:0005886 GO:0045202

XM_018051175.1 GO:0005938 GO:0005102 GO:0012506 GO:0005829 GO:0043197 GO:0070062 GO:0032467 GO:0006605 GO:0043542 GO:0008021 GO:0042803 GO:0043198 GO:0003779 GO:0031647 GO:0017022 GO:0030511 GO:0032435 GO:0048167 GO:0014047

XM_018043545.1 GO:0007346 GO:0043010 GO:0001822 GO:0060065 GO:0030099 GO:0007050 GO:0005737 GO:0045893 GO:0000122 GO:0055123 GO:0060669 GO:0071514 GO:0005634 GO:0016301 GO:0071901 GO:0035264 GO:0007568 GO:0004861 GO:0030325 GO:0050680 GO:0042551 GO:0030511 GO:0001501

XM_005684810.3 GO:0098609 GO:0002934 GO:0030054 GO:0016021

XM_018062301.1 GO:0030048 GO:0051497 GO:0005911 GO:0006897 GO:0072673 GO:0005769 GO:0031209 GO:0005856 GO:0070062 GO:0001525 GO:0035855 GO:0030027 GO:0001667 GO:0003779 GO:0001726 GO:0016601 GO:0010592 GO:0032403

XM_005683137.2 GO:0005634 GO:0010976 GO:0005737

XM_005685528.2 GO:0005765 GO:0031902 GO:0097576 GO:0006886 GO:0030897 GO:1990126 GO:1902774 GO:0006914 GO:0008333 GO:0034058 GO:0030123

XM_013972293.2 GO:0005654 GO:0016021

XM_018060829.1 GO:0005524 GO:0004004 GO:0005622 GO:0010501 GO:0003676

XM_005676846.3 GO:0001228 GO:0031490 GO:0043547 GO:0090103 GO:0032956 GO:0005737 GO:0007389 GO:0043565 GO:0005654 GO:0090179 GO:0042060 GO:0061029 GO:0007417 GO:0007398 GO:0008544 GO:0061436 GO:0045944

XM_013974992.2 GO:0006694 GO:0003854 GO:0016021 GO:0055114

XM_018047687.1 GO:0019752 GO:0016831 GO:0030170

XM_018065494.1 GO:0038061 GO:0031146 GO:0045893 GO:0046983 GO:0019005 GO:0005829 GO:0006470 GO:0031648 GO:0000209 GO:0045892 GO:0005634 GO:0061630 GO:0050852 GO:0045862 GO:0005813 GO:0002223 GO:0038095 GO:0042347 GO:0042753 GO:0016055 GO:0000086 GO:0051403

XM_018044456.1 GO:0080144 GO:0005765 GO:0015819 GO:0031301 GO:0015189 GO:0015809 GO:0015181 GO:1903401

XM_013975781.2 GO:0000278 GO:0030335 GO:0005524 GO:0071437 GO:0032587 GO:0005911 GO:0004702 GO:0048754 GO:0060244 GO:0033148 GO:0031532 GO:0005925 GO:0042060 GO:0043507 GO:0046777 GO:0007409 GO:0006915 GO:0051496 GO:0005518 GO:0005794 GO:0043234 GO:0033138 GO:0006887

XM_018060733.1 GO:0005856 GO:1903506 GO:0005654 GO:0035335 GO:0030971 GO:0046825 GO:0004725 GO:0005737 GO:0003712 GO:0001946 GO:0008285

XM_013962581.2 GO:0046658 GO:0007409

XM_018062603.1 GO:0071277 GO:0000978 GO:0005634 GO:0045944 GO:0001077

XM_018062538.1 GO:0016020 GO:0016787 GO:0005525

XM_018062696.1 GO:0003341 GO:0036157 GO:0036158

XM_018048903.1 GO:0060425 GO:0005615 GO:0022009 GO:0005109 GO:0072236 GO:0072205 GO:0044237 GO:0072089 GO:0070062 GO:0061180 GO:0048144 GO:0060428 GO:0071300 GO:0060070 GO:0072054 GO:0005788 GO:0060482 GO:0030666 GO:0005796 GO:0060710 GO:0016332 GO:0032364 GO:0060560 GO:0070307 GO:0050808 GO:0005886 GO:0072053 GO:0045165 GO:0030182 GO:0060535 GO:0003338 GO:0045669 GO:0005578 GO:0021871 GO:0072060 GO:0072061

XM_005697269.3 GO:0045666 GO:0003714 GO:0046982 GO:0001093 GO:0043621 GO:0001077 GO:0000122 GO:0003682 GO:0005667 GO:0016021 GO:1900746 GO:0065004 GO:0005634 GO:0000978 GO:0070888 GO:0016525 GO:0001011 GO:0045944 GO:0001087 GO:0042118 GO:0006367

XM_018049114.1 GO:0042742 GO:0002292 GO:0002250 GO:0038094 GO:0034987 GO:0016021 GO:0005886 GO:0045087

XM_018058000.1 GO:0070062 GO:0030425 GO:0005783 GO:0044822 GO:0044297 GO:0046726 GO:0036464 GO:0045070 GO:0005886

XM_018050090.1 GO:0006810 GO:0032052 GO:0016020 GO:0008206 GO:0005737 GO:0005215

XM_018049882.1 GO:0070062 GO:0030209 GO:0003940 GO:0005975

XR_001297548.2 GO:0003723 GO:0005634

XM_018061289.1 GO:0070062 GO:0005856 GO:0007015 GO:0007264 GO:0005525 GO:0005886 GO:0005737

XM_018048780.1 GO:0000122 GO:0031519 GO:0000792 GO:0003727

XM_018065426.1 GO:0016020 GO:0006739 GO:0005903 GO:0005881 GO:0050038 GO:0043621 GO:0055114 GO:0005739 GO:0016655 GO:0070062 GO:0006006 GO:0005634 GO:0051289 GO:0005902 GO:0004090 GO:0005997 GO:0042732

XM_018065138.1 GO:0005881 GO:0007020 GO:0003924 GO:0000930 GO:0000242 GO:0005525 GO:0005876 GO:0031122

XM_018053636.1 GO:0043547 GO:0017112

XM_018064908.1 GO:1902237 GO:0043433 GO:0031625 GO:0016607 GO:0042787 GO:0001085 GO:0005737 GO:0042593 GO:0019005 GO:2000676 GO:0000122 GO:0043161 GO:0030162 GO:0031463

XM_018048720.1 GO:0016709 GO:0005856 GO:0005654 GO:0055114 GO:0005737 GO:0003779 GO:0008270 GO:0030042 GO:0005886 GO:0017137 GO:0006887 GO:0071949

XM_005697656.3 GO:0045786 GO:0016049 GO:0005737 GO:0009636 GO:0008584 GO:0043065 GO:0031401 GO:0006357 GO:0003682 GO:0005634 GO:2000194 GO:0042771 GO:0002526 GO:0006473 GO:0048147 GO:0035914 GO:0006461

XM_005685913.3 GO:0097368 GO:0045892 GO:0036124 GO:0003677 GO:0005634 GO:0006349 GO:0007283 GO:0034773 GO:0017053 GO:0048821 GO:0045944 GO:0080182

XM_018067209.1 GO:0045116 GO:0005634 GO:0005524 GO:0019781 GO:0046982 GO:0005829 GO:0016881 GO:0007113 GO:0051726

XM_005681645.3 GO:0045665 GO:0043065 GO:0006357 GO:0046872 GO:0000981 GO:0005634 GO:0003676

XM_005684184.3 GO:0050850 GO:0050731 GO:0032481 GO:0090237 GO:0019370 GO:0002092 GO:0010803 GO:0004716 GO:0090330 GO:0002554 GO:0019901 GO:0019815 GO:0005178 GO:0031234 GO:0045087 GO:0042101 GO:0070372 GO:0045579 GO:0002250 GO:0007257 GO:0018105 GO:0048514 GO:0050764 GO:0032928 GO:0071404 GO:0005524 GO:0038083 GO:0032009 GO:0046641 GO:0042742 GO:0007169 GO:0045401 GO:0045425 GO:0071226 GO:0007229 GO:0002281 GO:0043366 GO:0050853 GO:0005634 GO:0030593 GO:0004715 GO:0001945 GO:0043306 GO:0046638 GO:0051090 GO:0042991 GO:0045780 GO:0045588 GO:0004674 GO:0050715 GO:0043313 GO:0033630

XR_001917345.1 GO:0005544 GO:0005635 GO:0010629 GO:0008283 GO:0005178 GO:0005829 GO:0006914 GO:0009651 GO:0048306 GO:0007599 GO:0006874 GO:0042584 GO:0005886 GO:0070062 GO:0044822 GO:0035176 GO:0009992 GO:0008360 GO:0005509 GO:0005789 GO:0030855

XM_005675284.3 GO:0005761 GO:0006412 GO:0003735

XM_005688769.3 GO:0046872 GO:0006355 GO:0003700 GO:0003676

XM_013970212.2 GO:0051015 GO:0005615 GO:0032839 GO:0030036 GO:0016567 GO:0007420 GO:0004842 GO:0015629 GO:0031463

XM_018055117.1 GO:1902902

NM_001314325.1 GO:0071156 GO:0005737 GO:0019901 GO:0000122 GO:0046578 GO:0043565 GO:0003700 GO:0005654 GO:0032873 GO:0090344 GO:0045944 GO:2000781 GO:2000377 GO:0000086 GO:0008284 GO:0006978

XM_018062055.1 GO:0000922 GO:0005874 GO:0045502 GO:0046982 GO:0007026 GO:0008568 GO:0005886 GO:0030424 GO:0008352 GO:0031117 GO:0005634 GO:0005813 GO:0008017 GO:0051301 GO:0007079 GO:0051013

XM_018049554.1 GO:0070062 GO:0019209 GO:0005634 GO:0035329 GO:0016301 GO:0046872 GO:0042327 GO:0005829 GO:0031952 GO:0019900

XM_018046713.1 GO:0005925 GO:0008270 GO:0005737

XM_005675051.3 GO:0070062 GO:0007286

XM_018049648.1 GO:0045892 GO:0000278 GO:0003677 GO:0000981 GO:0005634 GO:0006357 GO:0019904 GO:0005667 GO:0003712 GO:0008134

XM_005683367.3 GO:0030424 GO:0005230 GO:0007214 GO:0030054 GO:1902711 GO:0009791 GO:0005887 GO:0030534 GO:0007268 GO:0071420 GO:0004890 GO:0005254 GO:0045202 GO:1902476

XM_018060755.1 GO:0008152 GO:0016746 GO:0016021 GO:0005737

XM_018051064.1 GO:0043154 GO:0007050 GO:0005737 GO:0019901 GO:1902230 GO:0000731 GO:0048102 GO:0008285 GO:0030308 GO:0005654 GO:0009411 GO:0071901 GO:0033280 GO:0032526 GO:0004861 GO:0007605 GO:0000082 GO:0000079

XM_018045754.1 GO:0005615 GO:0060317 GO:0009897 GO:0034695 GO:0035556 GO:0015026 GO:0043235 GO:0007179 GO:0034673 GO:0016021 GO:0050431 GO:0070062 GO:0005114 GO:0005622 GO:0030509 GO:0032354 GO:0034699 GO:0060389 GO:0006955 GO:0070123 GO:0046332 GO:0051271

XM_018060477.1 GO:0016310 GO:0005524 GO:0004594 GO:0015937

XM_018060460.1 GO:0006432 GO:0004826 GO:0044822 GO:0009328

XM_005678156.3 GO:0070062 GO:0003073 GO:0016403 GO:0008270 GO:0005739 GO:0017014 GO:0045429 GO:0000052 GO:0006527

XM_018062665.1 GO:0016925 GO:0005654 GO:0007346 GO:0019948 GO:0016567 GO:0046982 GO:0043008 GO:0031510 GO:0004839 GO:0008022 GO:0005829 GO:0008047 GO:0043085

XM_018057233.1 GO:0019166 GO:0006631 GO:0008270 GO:0005739 GO:0055114

NM_001285692.1 GO:2000270 GO:0007155 GO:0007275 GO:0042127 GO:0008360

XM_005701281.3 GO:0005615 GO:0050829 GO:0010951 GO:0071222 GO:0005102 GO:0051918 GO:0035491 GO:1902042 GO:0045766 GO:0010469 GO:0070062 GO:0031012 GO:0002020 GO:0004867 GO:0061044 GO:0033629 GO:0090026 GO:0032757 GO:0010757 GO:2000352 GO:2000098 GO:0014912 GO:0048260 GO:0001300

XM_018048407.1 GO:2001275 GO:0005544 GO:0005815 GO:0072659 GO:0038028 GO:0005938 GO:0031340 GO:0032587 GO:0065002 GO:0005509 GO:0090314 GO:0030659

XM_005683864.3 GO:0070062 GO:0010634 GO:0010718 GO:0070374 GO:0042803 GO:0000139

XM_018048396.1 GO:0071560 GO:2000741 GO:0055059 GO:0032332

XM_018062216.1 GO:0000978 GO:0005654 GO:0045944 GO:0001077 GO:0001816 GO:0070884 GO:0005737

XM_018065382.1 GO:0005654 GO:0007165 GO:0015914 GO:0008526 GO:0005737

XM_018062516.1 GO:0050313 GO:0005759 GO:0006749 GO:0005654 GO:0005506 GO:0055114 GO:0070813

XM_005700534.3 GO:0004364 GO:0005634 GO:0004602 GO:0005737 GO:0010880 GO:0006749 GO:0005254 GO:0051099 GO:0060315 GO:1902476

XM_018054567.1 GO:0006397 GO:0016020 GO:0003723 GO:0005634 GO:0000166

XM_013962427.2 GO:0070062 GO:0005654

XM_018049886.1 GO:0031519 GO:0008270

XM_018039268.1 GO:0007186 GO:0016021 GO:0007166 GO:0004930

XM_005689359.3 GO:0001958 GO:0031012 GO:0030282 GO:0004222 GO:0044267 GO:0022617 GO:0006508 GO:0005518 GO:0005509 GO:0003417 GO:0008270 GO:0030574

XM_018053943.1 GO:0030317 GO:0018095 GO:0009566 GO:0007288 GO:0005813

XM_018066360.1 GO:0016021 GO:1902616 GO:0005739 GO:0015227

XM_018043600.1 GO:0005634 GO:0045598 GO:0030395 GO:0097193 GO:0050994 GO:0005739

XM_018038278.1 GO:0008270 GO:0005737

XM_018061592.1 GO:0005654 GO:0005794 GO:0051044 GO:0007010 GO:0022604 GO:0070064 GO:0005886

XM_005674883.3 GO:0030308 GO:0009986 GO:0005887 GO:0042060

XM_018040023.1 GO:0048188 GO:0000166 GO:0042800 GO:0051568 GO:0003676 GO:0008013

XM_018048648.1 GO:0055085 GO:0005332 GO:0005887 GO:0005328 GO:0006836 GO:0015812

XM_018066403.1 GO:0008083 GO:0045747 GO:0042127 GO:1990134 GO:0007283 GO:0003016 GO:0030334 GO:0005887 GO:0042492 GO:0030155 GO:0045061 GO:0009912 GO:0001701 GO:0042475 GO:0016331 GO:0005509 GO:0007220 GO:0005112 GO:0007049 GO:0001501

XM_018055663.1 GO:0070062 GO:0005509

XM_018045102.1 GO:0000978 GO:0005654 GO:0045785 GO:0001077 GO:0005737 GO:0002250 GO:0046983 GO:0006959 GO:0045944 GO:0045670

NM_001314237.1 GO:0038048 GO:0016021 GO:0038003

XM_018059205.1 GO:0005887 GO:0007166

XM_018053367.1 GO:0060065 GO:0043433 GO:0001077 GO:0008584 GO:0003682 GO:0005496 GO:0030520 GO:0000978 GO:0008209 GO:0001046 GO:0042802 GO:0048146 GO:0038052 GO:0000790 GO:0034056 GO:0010863 GO:0060750 GO:0045944 GO:0045429 GO:0007200 GO:0051000 GO:0002064 GO:0001547 GO:0060068 GO:0010629 GO:0051117 GO:0060527 GO:0051091 GO:0060745 GO:0008270 GO:0060523 GO:0005886 GO:0043124 GO:0035327 GO:0060687 GO:0060749 GO:0005794 GO:0071391 GO:0007204 GO:0008134 GO:0042981 GO:0008013 GO:0071392

NM_001285591.1 GO:0007186 GO:0032868 GO:0031625 GO:0030073 GO:0004980 GO:0045780 GO:0007631 GO:0016021 GO:0042923 GO:0005886 GO:0030819

XM_018056765.1 GO:0016021 GO:0031966 GO:0006839

XM_018044429.1 GO:0006509 GO:0016020 GO:0001649 GO:0005615 GO:0000381 GO:0051260 GO:0071347 GO:0005515 GO:0003682 GO:0000166 GO:0044530 GO:0001047 GO:0070062 GO:0005654 GO:0003729 GO:0071013 GO:0048026 GO:0045944 GO:0005719 GO:0048025 GO:0019013

XM_005686312.3 GO:0005783 GO:0016021 GO:0007411 GO:0005886

XM_005684696.3 GO:0061351 GO:0003677 GO:0000784 GO:0005654 GO:0006260 GO:0005664

XM_013976440.1 GO:0016021

XM_018041712.1 GO:0008395 GO:0020037 GO:0005506 GO:0055114 GO:0019373 GO:0008392

XM_018057742.1 GO:0001649 GO:0005634 GO:0010508 GO:0001894 GO:0045893 GO:0043130 GO:0006511 GO:0005829 GO:0005776 GO:0000045

XM_018046246.1 GO:0070062 GO:0003677 GO:0005654 GO:0042800 GO:0051568 GO:0032635 GO:0043409 GO:0045944 GO:0005794 GO:0046975 GO:0003682 GO:0002674 GO:0008270 GO:0097676 GO:0043124

XM_018049743.1 GO:0045892 GO:0046872 GO:0005634 GO:0072562 GO:0006275 GO:0006974 GO:0042803 GO:0008327

XM_005680154.3 GO:0005654 GO:0044822 GO:0005794

XM_018049180.1 GO:0006886 GO:0030127 GO:0008270 GO:0001701 GO:0006888

XM_005696978.3 GO:0004653 GO:0032580 GO:0018242 GO:0016266 GO:0048471 GO:0030246 GO:0016021 GO:0005576 GO:0005789 GO:0018243 GO:0030145

XM_013974450.2 GO:0045666 GO:0003714 GO:0046982 GO:0001093 GO:0043621 GO:0001077 GO:0000122 GO:0003682 GO:0005667 GO:0016021 GO:1900746 GO:0065004 GO:0005634 GO:0000978 GO:0070888 GO:0016525 GO:0001011 GO:0042803 GO:0043425 GO:0045944 GO:0001087 GO:0042118 GO:0006367

XM_013966999.2 GO:0003677 GO:0071339 GO:0006355 GO:0003700 GO:0005737 GO:0046983

XM_018039943.1 GO:0046330 GO:0031434 GO:0030036 GO:0005524 GO:0016023 GO:0008360 GO:0043235 GO:0005730 GO:0016021 GO:0004702 GO:0031572 GO:0048041 GO:0000186

XM_005683226.3 GO:0038083 GO:0005524 GO:0042127 GO:0035556 GO:0005102 GO:0007169 GO:0001865 GO:0005911 GO:0031234 GO:0045087 GO:0004715 GO:0050852 GO:0032609 GO:0002250 GO:0007202 GO:0032633

NM_001285689.1 GO:0030324 GO:0051897 GO:0005783 GO:0005615 GO:0071356 GO:0006032 GO:0008061 GO:0070555 GO:0005975 GO:0004568 GO:0045766 GO:0009612 GO:0070741 GO:0007250 GO:0072606 GO:0048471 GO:0006915 GO:0006954 GO:0010800 GO:0030246 GO:0070374

XM_005682787.2 GO:0016477 GO:0021979 GO:0001205 GO:0001568 GO:0048850 GO:0000122 GO:0060126 GO:0005667 GO:0003682 GO:0005634 GO:0009953 GO:0043066 GO:0060070 GO:0008022 GO:0045944 GO:0001206 GO:0000980 GO:0008013

XM_018063911.1 GO:0005887 GO:0005242 GO:0034765 GO:0010107

XM_013969976.2 GO:0016021 GO:0051726 GO:0005737

XM_018040576.1 GO:0045022 GO:0001881 GO:0005524 GO:0005829 GO:0033572 GO:0043086 GO:0016021 GO:0005769 GO:0055037 GO:0004864 GO:0070853 GO:0048471 GO:0046777 GO:0018107 GO:0004674 GO:0018105 GO:0005794 GO:0032456

XM_018062202.1 GO:0036117 GO:0005737 GO:0045893 GO:0045226 GO:1900106 GO:0016021 GO:0085029 GO:0030213 GO:0050501

XM_018048373.1 GO:0005925

XM_018044780.1 GO:0097038 GO:0035584 GO:0007283 GO:0051561 GO:0000139 GO:0005887 GO:0005739 GO:0042288 GO:0043280 GO:0032471 GO:2001244 GO:0005811 GO:0005784 GO:0007204 GO:0006888 GO:1903071 GO:0032403 GO:0070973

XM_018059308.1 GO:0070062 GO:0004198 GO:0009986 GO:0006508 GO:0005925 GO:0005622

XM_018043783.1 GO:0005654 GO:0018105 GO:0005524 GO:0004674 GO:0035556

XM_013968712.2 GO:0070062 GO:0005765 GO:0008021 GO:0043524 GO:0042470 GO:0016021 GO:0005739 GO:0005886

XM_018062656.1 GO:0070016 GO:0051721 GO:0000159 GO:0032403

XM_018057664.1 GO:0071797 GO:0051092 GO:0050852 GO:0060546 GO:0042346 GO:0004842 GO:0043130 GO:0043161 GO:0008270 GO:2001238 GO:0032088 GO:0097039

XM_018048280.1 GO:0008289 GO:0006869 GO:0005576 GO:0042157

XM_018054617.1 GO:0042060 GO:0035023 GO:0043547 GO:0032956 GO:0005089

XM_018046071.1 GO:0046872

XM_018060151.1 GO:0005874 GO:0021954 GO:0002162 GO:0008017 GO:0005737 GO:0048813 GO:0043005 GO:0005875 GO:0005730 GO:0001578

XM_018057738.1 GO:0070936 GO:0002669 GO:0070534 GO:0090085 GO:0005938 GO:0031410 GO:0042787 GO:0050687 GO:0016874 GO:0046329 GO:0032088 GO:0005886 GO:0070062 GO:0005634 GO:0061630 GO:0035519 GO:0045236 GO:0043066 GO:0046642 GO:0045732 GO:0043021

XM_018041377.1 GO:0005730 GO:0031519 GO:0045944 GO:0008270 GO:0005813

XM_005685063.3 GO:0030425 GO:0033554 GO:0005783 GO:0005615 GO:0035690 GO:0005874 GO:0071356 GO:0097211 GO:2000310 GO:0043196 GO:0043679 GO:0035865 GO:0007565 GO:0051460 GO:1900011 GO:0005767 GO:0005771 GO:0042445 GO:0071320 GO:0071277 GO:0031045 GO:0051424 GO:0009755 GO:0048149 GO:0005634 GO:0045055 GO:0071314 GO:0043204 GO:0006954 GO:0002125 GO:0005794 GO:0071391 GO:0001963 GO:0071392

XM_018058165.1 GO:0045022 GO:0006622 GO:0005770 GO:0042802 GO:0005829 GO:0008333 GO:0031313 GO:0005769 GO:0035091

XM_005686798.3 GO:0070936 GO:0032496 GO:0001819 GO:0016874 GO:0030890 GO:0034450 GO:0043331 GO:0042130 GO:0005515 GO:0005829 GO:0008063 GO:0032088 GO:0005634 GO:0031398 GO:0034141 GO:0043123 GO:0034145

XM_005677168.3 GO:0005791 GO:0006782 GO:0030054 GO:0016023 GO:0055114 GO:0004729 GO:0007631 GO:0031304 GO:0007218 GO:0045202

XM_005676742.2 GO:0010494 GO:0008344 GO:0007283 GO:0003730 GO:0000932 GO:0048863 GO:1900246 GO:2000637 GO:0051726 GO:0061157

XM_018057250.1 GO:0007165

XM_005688980.3 GO:0008152 GO:0016746 GO:0016021

XM_018065008.1 GO:0016021 GO:0006505 GO:0016788 GO:0031227

XM_018040158.1 GO:0008146

XR_001917611.1 GO:0000122 GO:0000978 GO:0005654 GO:0001078

XM_005697510.3 GO:0046872 GO:0044822

XM_018042020.1 GO:0097638 GO:0002537 GO:0043030 GO:0005292 GO:0050727 GO:0006865 GO:0097639 GO:0005887 GO:0097627 GO:0097640 GO:0005289 GO:0006809 GO:0015297 GO:0097626

XM_018058686.1 GO:0006355 GO:0003700 GO:0005488

XM_018040984.1 GO:0035272 GO:0031226 GO:0030667 GO:0045055 GO:0000139 GO:0017157 GO:0043001 GO:0007264 GO:0005525 GO:0019002

XM_018048662.1 GO:0005524 GO:0006468 GO:2000021 GO:0035556 GO:0003084 GO:0032414 GO:0004674 GO:0005829 GO:0019869 GO:0019902 GO:0090188 GO:0010923

XM_018055274.1 GO:0005634 GO:0005524 GO:0043408 GO:2000659 GO:0046777 GO:0019901 GO:0004674 GO:0019904 GO:0034599 GO:0043234 GO:0016021 GO:0031966 GO:0005789

XM_018042832.1 GO:0010923 GO:0007268 GO:0019233 GO:0019900 GO:0030054 GO:0044224 GO:0045211

XM_005699866.3 GO:0007165 GO:0008601 GO:0000159 GO:0034047

XM_018045917.1 GO:0030425 GO:0061337 GO:0086009 GO:0097038 GO:0034765 GO:0051260 GO:0005251 GO:0043025 GO:0046872 GO:0044325 GO:0071435 GO:0042383 GO:0071773 GO:0005250 GO:0008076

XM_018043448.1 GO:0005856

XM_018061272.1 GO:0005212 GO:0007601

XM_005682740.2 GO:0016021 GO:0097250 GO:0005739

XM_018061538.1 GO:0045892 GO:0006357 GO:0005654 GO:0043565 GO:0003700 GO:0005737 GO:0045893

XM_018047208.1 GO:0031290 GO:0086080 GO:0030424 GO:0001525 GO:0019227 GO:0045162 GO:0007417 GO:0008104 GO:0016021 GO:0034113 GO:0030506 GO:0010975 GO:0045202 GO:0005886

XM_018064237.1 GO:0005634 GO:0006355 GO:0043565 GO:0003700

XM_005685465.3 GO:0000398 GO:0044822 GO:0016021 GO:0071013

XM_005690908.3 GO:0031965 GO:0044822 GO:0005763 GO:0005743 GO:0070124 GO:0003735 GO:0070125

XM_018046025.1 GO:0071108 GO:0003677 GO:0004843 GO:0005634 GO:0005737 GO:0070530 GO:0000122 GO:1900181 GO:0008270 GO:0032717 GO:1990380 GO:0043124 GO:0070536 GO:0002385 GO:0035871

XM_018063482.1 GO:0003677 GO:0005634 GO:0008270

XM_018051061.1 GO:0003376 GO:0016021 GO:0038036 GO:0005886

XM_005697494.3 GO:0070062 GO:0004615 GO:0005737 GO:0009298 GO:0043025

XM_005675635.3 GO:0030141 GO:0010906 GO:0005578

XM_013966845.2 GO:0080008 GO:0005654 GO:0016567

XM_018058460.1 GO:0003723

XM_005683745.3 GO:0070062 GO:0016021

XR_001917613.1 GO:0005201 GO:0005587 GO:0030198 GO:0071230

XM_018053861.1 GO:0070062

XM_005676014.3 GO:0045892 GO:0003677 GO:0005634

XM_005677800.3 GO:0005765 GO:0032526

XM_018062539.1 GO:0042802 GO:0000184 GO:0005622

XM_018052017.1 GO:0016020 GO:0042147 GO:0043547 GO:0017112 GO:0005829 GO:1903363 GO:0034066 GO:0017137

XM_018042878.1 GO:0005654 GO:0000398 GO:0044822 GO:0045131 GO:0030238 GO:0042802 GO:0050810 GO:0005681 GO:0033327 GO:0008270

XM_018045757.1 GO:0009301 GO:0005730 GO:0070940 GO:0008420 GO:0005737 GO:0016591

XM_013973718.2 GO:0010494 GO:0008344 GO:0007283 GO:0003730 GO:0000932 GO:0048863 GO:1900246 GO:2000637 GO:0051726 GO:0061157

XM_018067186.1 GO:0045892 GO:0003677 GO:0005634 GO:0047485 GO:0048853 GO:0030916 GO:0008022 GO:0003682 GO:0043584

XM_005675378.3 GO:0007264 GO:0005622 GO:0005525

XM_013974426.2 GO:0006486 GO:0008373 GO:0030173 GO:0097503

XM_005678567.3 GO:0045892 GO:0014898 GO:0031625 GO:0070544 GO:0035064 GO:0005737 GO:1900113 GO:0005730 GO:0051864 GO:0005721 GO:0010507 GO:0008270

XM_005677645.3 GO:0003677 GO:0005654 GO:0005694 GO:0034968 GO:0005737 GO:0008270 GO:0005886 GO:0018024

XM_005697075.3 GO:0005509

XM_005699878.3 GO:0046872 GO:0005794 GO:0016021 GO:0016192

XM_018065122.1 GO:0070062 GO:0000220 GO:0005654 GO:0070072 GO:0051117 GO:0042470 GO:0007035 GO:0015986 GO:0015991 GO:1901998 GO:0005794 GO:0016021 GO:0005886 GO:0030659 GO:0046961

XM_005682692.3 GO:0070062 GO:0005739 GO:0055114 GO:0004043

XM_005675949.3 GO:0000287 GO:0004709 GO:0005634 GO:0005524 GO:0044822 GO:0000077 GO:0007050 GO:0005737 GO:0007257 GO:0043065 GO:0009314 GO:0007010 GO:0000186

XM_018038714.1 GO:0043231 GO:0005525 GO:0003924

XM_005684041.3 GO:0050731 GO:0046330 GO:0043267 GO:0045428 GO:0010595 GO:0007172 GO:0010752 GO:2000060 GO:0031234 GO:0045766 GO:0004871 GO:0032960 GO:0008285 GO:0005856 GO:0005654 GO:0005925 GO:0070098 GO:0043066 GO:0030027 GO:0008360 GO:2000249 GO:0048471 GO:0043552 GO:0071300 GO:0050848 GO:0002040 GO:0051000 GO:0001954 GO:0005524 GO:0038083 GO:0071498 GO:0030502 GO:0051279 GO:0045638 GO:2000538 GO:0007229 GO:0030838 GO:0033209 GO:0045453 GO:0004715 GO:0030826 GO:0010976 GO:2000114 GO:0042976 GO:0010758 GO:0048010 GO:0070374 GO:0002315 GO:0008284

XM_005678190.3 GO:0007186 GO:0030246 GO:0016021 GO:0007166 GO:0004930

XM_005690008.3 GO:0005654

XM_005696376.3 GO:0007186 GO:0043031 GO:0031410 GO:0007166 GO:0071073 GO:0042593 GO:0004930 GO:0009986 GO:0045444 GO:0006112 GO:0016021 GO:0045177 GO:0043129

XM_005688174.3 GO:0030141

XM_005680007.2 GO:0005615 GO:0045747 GO:0022004 GO:0005737 GO:0021527 GO:0043568 GO:0005109 GO:0022408 GO:0001658 GO:0042472 GO:1990403 GO:0009611 GO:0000578 GO:0005125 GO:0048146 GO:0071542 GO:0045599 GO:0090263 GO:0010592 GO:0042770 GO:0045944 GO:2000059 GO:0030514 GO:0033278 GO:0033077 GO:0021797 GO:0071425 GO:0044336 GO:0051091 GO:0030512 GO:0061184 GO:0001934 GO:0060061 GO:0021588 GO:0060348 GO:0007520 GO:0044212 GO:0021536 GO:0005578 GO:0048664 GO:0090344 GO:0030579 GO:0009986 GO:0019904 GO:0010812 GO:0021551

XM_018042225.1 GO:0005654 GO:0000398 GO:0044822 GO:0005515 GO:0005681 GO:0000166 GO:0030855

XM_005695643.3 GO:0003723 GO:0005730 GO:0005737

XM_018052586.1 GO:0051482 GO:0010977 GO:0005887 GO:0060326 GO:0005829 GO:0030165 GO:0035727 GO:0007193 GO:0032060 GO:0000187 GO:0008360 GO:0070915 GO:0005768 GO:0043123 GO:0051496 GO:0009986 GO:0030139 GO:0035025 GO:0007202

XM_018044528.1 GO:0070588 GO:0030315 GO:0086010 GO:0006936 GO:0034765 GO:0031674 GO:0008331 GO:0005891

XM_018066646.1 GO:1900037 GO:0071108 GO:0004843 GO:0030433 GO:0031625 GO:0051879 GO:0005829 GO:0005789 GO:1990380 GO:0050821 GO:1901799 GO:0048642

XM_018055743.1 GO:0003677 GO:0005634 GO:0044822 GO:0015630 GO:0007286 GO:0003725 GO:0005737 GO:0007275 GO:0007638 GO:0003727

XM_005678702.3 GO:0005829 GO:0006888 GO:0030008 GO:0000139

XM_005684256.3 GO:0010390 GO:0042393 GO:0031062 GO:0031625 GO:0003730 GO:2001168 GO:0045893 GO:0016874 GO:0006511 GO:0005730 GO:0003682 GO:0008270 GO:0016568 GO:0000209 GO:0005654 GO:0033503 GO:0002039 GO:0030336 GO:0004842 GO:1900364 GO:0003713

XM_018052950.1 GO:0035257 GO:0045944 GO:0030374 GO:0005622

XM_005678547.3 GO:0005654 GO:0004853 GO:0005829 GO:0006782

XM_005696590.3 GO:0003676

XM_018053870.1 GO:0070062

XM_005689978.3 GO:0043231 GO:0005737 GO:0009313 GO:0052796 GO:0052795 GO:0052794 GO:0005886 GO:0006689

XM_018058852.1 GO:0005605 GO:0005201 GO:0032836 GO:0005587

XM_018055359.1 GO:0016020 GO:0005506 GO:0000976 GO:0030521 GO:0005737 GO:0046293 GO:2000036 GO:2000736 GO:0050681 GO:0051573 GO:0036123 GO:0003700 GO:0001047 GO:0005654 GO:0007290 GO:0045944 GO:0033169

XM_018054420.1 GO:0005524 GO:0060068 GO:0005635 GO:0051250 GO:0046982 GO:0043491 GO:0034122 GO:0007283 GO:0050728 GO:0004713 GO:0016021 GO:0042698 GO:0018108 GO:0043548 GO:0045824 GO:0034446 GO:0001779 GO:0043524 GO:0032940 GO:0043277 GO:0046777 GO:0021885 GO:0070050 GO:0005789 GO:0070527

XM_018043348.1 GO:0070062 GO:0016310 GO:0036010 GO:0007165 GO:0005737 GO:0006954 GO:0035325 GO:0006914 GO:0019900 GO:0045087 GO:0030855

XM_005686697.3 GO:0045892 GO:0005634 GO:0006338 GO:0003714 GO:0007507 GO:0005737 GO:0010831 GO:0046872 GO:0035914 GO:0045663

XM_013965255.1 GO:0016021 GO:0055114 GO:0016491

XM_018043746.1 GO:0030424 GO:0048786 GO:0005524 GO:0043209 GO:0030054 GO:0003824 GO:0014069 GO:0019901 GO:0003779 GO:0007269 GO:0030672 GO:0005794 GO:0000795

XM_018047191.1 GO:0016021

XM_005691200.3 GO:0008217 GO:0004962 GO:0016021 GO:0030818 GO:0005886 GO:0007585 GO:0014032 GO:0007507 GO:0086100 GO:0001701 GO:0001666 GO:0015758 GO:0048484 GO:0060322 GO:0014824 GO:0001569

XM_018055563.1 GO:0003677 GO:0045666 GO:0047485 GO:0000435 GO:2001038 GO:0046983 GO:0043065 GO:0043967 GO:0003682 GO:0004402 GO:0060713 GO:0030374 GO:0000790 GO:0016922 GO:0035257 GO:0002155 GO:0019899 GO:0008134

XM_005692567.3 GO:0070062 GO:0016020 GO:0016042 GO:0003847 GO:0042802 GO:0047179 GO:0007420 GO:0007283 GO:0005737

XM_018061366.1 GO:0032525 GO:0072661 GO:0032580 GO:0035459 GO:0048208 GO:0033116 GO:0036342 GO:0005515 GO:0030663 GO:0048205 GO:0016021 GO:0001893 GO:0060716 GO:0006890 GO:0007030 GO:0010628 GO:0035264 GO:0042589 GO:0001947 GO:0034260 GO:0005789 GO:0001843 GO:0012507

XM_018060743.1 GO:0045930 GO:0070935 GO:0015030 GO:0003730 GO:0005737

XM_018057121.1 GO:0016310 GO:0031965 GO:0005524 GO:0046982 GO:0042803 GO:0005737 GO:0004756 GO:0005886

XM_013962458.2 GO:0001819 GO:0031295 GO:0042104

XM_018040925.1 GO:0005634 GO:2001014 GO:0000381 GO:0000166 GO:0050885 GO:0005737 GO:0003676

XM_005696649.3 GO:0007155 GO:0016021

XR_001917499.1 GO:0046330 GO:0070935 GO:0046982 GO:0032496 GO:0005737 GO:0032760 GO:0090073 GO:0005886 GO:0034137 GO:0035663 GO:0005654 GO:0045410 GO:0051092 GO:0032738 GO:0035665 GO:0032757 GO:0045171 GO:0043123 GO:0005080 GO:0035662 GO:0070374 GO:0034145

XM_005675998.3 GO:0006457 GO:0003755 GO:0005654 GO:0044822 GO:0000413 GO:0005737

XM_018058467.1 GO:0070062 GO:0005634 GO:0005615 GO:0042470 GO:0005764 GO:0006541 GO:0006508 GO:0034722 GO:0046900

XM_005688259.3 GO:0044822 GO:0046982 GO:0042803 GO:0048471

XM_018062466.1 GO:0000122 GO:0005634 GO:0003682 GO:0048286

XM_018060770.1 GO:0018345 GO:0016409 GO:0016021 GO:0007224

XM_018066876.1 GO:0006355 GO:0030275

XM_018065691.1 GO:0016324 GO:0010043 GO:0005385 GO:0061088 GO:0030141 GO:0005730 GO:0006824 GO:0005794 GO:0016021 GO:0071577

XM_018056369.1 GO:0097680 GO:0006260 GO:0003910 GO:0035019 GO:0005737 GO:0010332 GO:0002328 GO:0000012 GO:0000793 GO:0005654 GO:0005925 GO:0048146 GO:0043524 GO:0033152 GO:0051102 GO:0071897 GO:0008022 GO:0033153 GO:0003677 GO:0045190 GO:0005524 GO:0032807 GO:0033077 GO:0051103 GO:0005886 GO:0010165 GO:0051276 GO:0001701 GO:0007417 GO:0005958 GO:0050769 GO:0006297

XM_018051820.1 GO:0000922 GO:0005813

XM_018056845.1 GO:0071404 GO:0031623 GO:0019960 GO:0046982 GO:0030054 GO:0042470 GO:0032587 GO:0071438 GO:0008305 GO:0007229 GO:0001618 GO:0055037 GO:0090004 GO:0030027 GO:0007160 GO:0010710 GO:0046872 GO:0042383 GO:0046718

XM_005678380.3 GO:0016021

NM_001314194.1 GO:0008643 GO:0045815 GO:0005794 GO:0016021 GO:0042946 GO:0005886 GO:0042947

XM_005683657.3 GO:0006260 GO:0001077 GO:0060510 GO:0044300 GO:0005730 GO:0071679 GO:0060662 GO:2000791 GO:0010001 GO:0060486 GO:0007608 GO:0000978 GO:0060689 GO:0021740 GO:2000795 GO:0021960 GO:0001106 GO:0002062 GO:0061141 GO:0060509 GO:0043392 GO:0045944 GO:1902894 GO:0030902

XM_018053428.1 GO:0010332 GO:0048666 GO:0055072 GO:0022904 GO:0009791 GO:0003032 GO:0019430 GO:0007507 GO:0043209 GO:0045599 GO:0048147 GO:0006749 GO:0001315 GO:0055093 GO:0005743 GO:0045429 GO:0005759 GO:0004784 GO:0048773 GO:0006357 GO:0042554 GO:0030097 GO:0030145 GO:0001836 GO:0008630 GO:0001889 GO:0014823 GO:0008631 GO:0048678 GO:0042542 GO:0050790 GO:0007626 GO:0003069 GO:0051881

XM_005699392.3 GO:0070062 GO:0005634 GO:0008270 GO:0016788

XM_018057482.1 GO:0046872

XM_013977197.2 GO:0016021

XM_005680885.3 GO:0005770 GO:0015578 GO:0016021 GO:0015761 GO:0005537 GO:0048471 GO:0000323

XM_018047331.1 GO:0042552 GO:0005634 GO:0035690 GO:0034976 GO:0043547 GO:0005085 GO:0001541 GO:0009408 GO:0014002 GO:0006413 GO:0003743 GO:0005851 GO:0014003

XM_018040336.1 GO:0009000 GO:0005829 GO:0016740

XM_005693748.3 GO:0005768 GO:0005765 GO:0015485 GO:0016021 GO:0006701 GO:0005739

XM_005678935.3 GO:0016021 GO:0000139

XM_005679166.3 GO:0005654 GO:0006355 GO:0034605 GO:0005737 GO:0046983

XM_018059240.1 GO:0000079 GO:0019901

XM_005697719.3 GO:0000149 GO:0016323 GO:0005615 GO:0030335 GO:0033194 GO:0035774 GO:0000322 GO:0043197 GO:0070062 GO:0090004 GO:0035749 GO:0031629 GO:0008021 GO:0030027 GO:0048471 GO:0006886 GO:0005768 GO:1902041 GO:0050921 GO:0031201 GO:0005484 GO:0043311 GO:2000010 GO:0045785 GO:0005901 GO:0051024 GO:0060291 GO:0016021 GO:0048278 GO:0016230 GO:0043085 GO:0035493 GO:0043219 GO:0042581 GO:0005802 GO:0009986 GO:0017022 GO:0008284

XM_018063674.1 GO:0002020 GO:0070062 GO:0005615 GO:0010951 GO:0034097 GO:0005578 GO:0046872 GO:0008191 GO:0009725 GO:0051045

XM_018066338.1 GO:0016020 GO:0005524 GO:0031072 GO:0051087 GO:0009408 GO:0046872 GO:0005829 GO:0051082 GO:0090084 GO:0042026

XM_005678429.3 GO:0006869 GO:0005794

XM_005680216.3 GO:0006979 GO:0033743 GO:0005783 GO:0030091 GO:0008270 GO:0005739 GO:0055114

XM_018066939.1 GO:0003779 GO:0000781 GO:0048188 GO:0072357 GO:0003682 GO:0000785 GO:0042800 GO:0051568

XM_018053919.1 GO:0009072 GO:0003824 GO:0005739

XM_013964473.2 GO:0031267 GO:0005052 GO:0005829 GO:0043235

XM_013966707.2 GO:0005813

XM_018053558.1 GO:0046872 GO:0005524 GO:0003774 GO:0016459 GO:0043547 GO:0035556 GO:0005096

XM_013966779.2 GO:0005509

XM_018066663.1 GO:0070062 GO:0009058 GO:0016779 GO:0005739

XM_005699091.3 GO:0046426 GO:0051965 GO:0005737 GO:0006469 GO:0004860 GO:0016021 GO:1902004 GO:0019221

XM_005691447.3 GO:0042694 GO:0030308 GO:0048747 GO:0055010 GO:0003785 GO:0030016 GO:0009791 GO:0060048 GO:0005509 GO:0098735 GO:0015629 GO:0055003

XM_005676006.3 GO:0042448 GO:0008209 GO:0047035 GO:0055114 GO:0004022 GO:0042904 GO:0030176 GO:0004745

XM_005681375.3 GO:0070062 GO:0032947 GO:0005925 GO:0043547 GO:0005085 GO:0032008 GO:0071986 GO:0071230 GO:0034613

XM_005689835.2 GO:0007186 GO:0050911 GO:0003707 GO:0045777 GO:0004984 GO:0071398 GO:0004930 GO:1900135 GO:0016021 GO:0043401 GO:0005886

XM_005679471.3 GO:0016021

NM_001314169.1 GO:0048617 GO:0019838 GO:0030073 GO:0060836 GO:0005737 GO:0009791 GO:0009749 GO:0004702 GO:0035265 GO:0007368 GO:0001702 GO:0007507 GO:0032147 GO:0030509 GO:0042475 GO:0001974 GO:0061298 GO:0030324 GO:0009952 GO:0023014 GO:0005524 GO:0048185 GO:0060841 GO:0001822 GO:0043235 GO:0000122 GO:0016021 GO:0060021 GO:0005886 GO:0032927 GO:0048705 GO:0007498 GO:0004712 GO:0045669 GO:0060840 GO:0031016 GO:0030501 GO:0046872 GO:0001946 GO:0004675

XM_005691708.3 GO:0006457 GO:0051087 GO:0051259

XM_018050418.1 GO:0010609 GO:0005634 GO:0044822 GO:0017091 GO:0008270 GO:0061157 GO:0048471

XM_005684778.2 GO:0046872 GO:0007605 GO:0006355 GO:0007275 GO:0035335 GO:0004725

XM_005681818.3 GO:0005524 GO:0006468 GO:0004692 GO:0030553

XM_005687976.3 GO:0070062 GO:0008270 GO:0009056 GO:0030855 GO:0016788

XM_018055335.1 GO:0070062 GO:0005615 GO:0045353 GO:0006953 GO:0005151 GO:2000660 GO:0030073 GO:0045352 GO:0005150 GO:0034115 GO:0051384 GO:0006629

XM_005700575.3 GO:0006612 GO:0018344 GO:0050790 GO:0055114 GO:0005092 GO:0005829 GO:0007264 GO:0005968 GO:0017137 GO:0016491

XM_005690784.3 GO:0005654 GO:0045862 GO:0005739

XM_005690364.3 GO:0016021

XM_018047811.1 GO:0070588 GO:0022839 GO:0035725 GO:0044736 GO:0071467 GO:0007613 GO:0005887 GO:0008306 GO:0010447 GO:0050915 GO:0045202 GO:0046929 GO:0042391

XM_018046258.1 GO:0005654 GO:0044822 GO:0005761 GO:0015935 GO:0006915

XM_018038365.1 GO:0007165

XM_018054459.1 GO:0023014 GO:0000278 GO:0005524 GO:0007612 GO:0005737 GO:0007613 GO:0007626 GO:0004702

XM_013963958.2 GO:0045892 GO:0005654 GO:0046982 GO:0005813 GO:0042803 GO:0044212 GO:0046872 GO:0043425 GO:0045944 GO:0043565 GO:0003700

XM_018048607.1 GO:0002020 GO:0070062 GO:0005783 GO:0047485 GO:0019865 GO:0033093 GO:0031589 GO:0051260 GO:0030168 GO:0051087 GO:0042803 GO:0005578 GO:0005178 GO:0005518 GO:0001948

XM_018054820.1 GO:0016021

XM_013977256.2 GO:0005615 GO:0005125 GO:0051607 GO:0043330 GO:0002250 GO:0005132 GO:0042100 GO:0006959 GO:0002286 GO:0033141 GO:0002323 GO:0030183 GO:0019221 GO:0045087

XM_018053556.1 GO:0046872 GO:0005524 GO:0003774 GO:0016459 GO:0043547 GO:0035556 GO:0005096

XM_005675182.3 GO:0051897 GO:0005125 GO:0045654 GO:0005179 GO:0038163 GO:1902035 GO:0005576 GO:0070374

XM_018059353.1 GO:1902260 GO:0030425 GO:0005249 GO:0015459 GO:0005737 GO:0031982 GO:0043204 GO:0045121 GO:0032809 GO:0008076

XM_018054988.1 GO:0007169 GO:0005634 GO:0005057 GO:0007265 GO:0005737 GO:0005158

XM_018045041.1 GO:0005634 GO:0031307 GO:0000422

XM_013966935.2 GO:0003676

XM_005701112.3 GO:0032755 GO:0007252 GO:0005783 GO:0071260 GO:0032009 GO:0003725 GO:0043235 GO:0001932 GO:0005887 GO:0045078 GO:0034154 GO:0004888 GO:0003727 GO:0035197 GO:0051607 GO:0005764 GO:0042346 GO:0006954 GO:0045359 GO:0005768 GO:0002755 GO:0032722 GO:0045416 GO:0045356

XM_018059058.1 GO:0032020 GO:0042296 GO:0005524 GO:0016567 GO:0019941

XM_005682882.3 GO:0043231 GO:0005925 GO:0005886

XM_018041146.1 GO:0060444 GO:0005737 GO:0045893 GO:0046983 GO:0019005 GO:0060828 GO:0071407 GO:0045309 GO:0006470 GO:0031648 GO:0000209 GO:0045892 GO:0005634 GO:0061630 GO:0045862 GO:0033598 GO:0043122 GO:0061136 GO:0043161 GO:0042753 GO:0051726 GO:0008013

XM_005678232.2 GO:0006508 GO:0003923 GO:0008234 GO:0042765 GO:0016255 GO:0034394

XM_018051503.1 GO:0007186 GO:0070915 GO:0009986 GO:0030139 GO:0016021

XM_005697024.2 GO:0071346 GO:0006833 GO:0015250 GO:0009897 GO:0016021 GO:0050891 GO:0005737

XM_005694915.3 GO:0005654 GO:0005739 GO:0003924

XM_018053683.1 GO:0016021

XM_013962628.2 GO:0006886 GO:0005794 GO:0005829 GO:0031338 GO:0042147 GO:0017137 GO:0005096

XR_001919267.1 GO:0005634 GO:0008270 GO:0043066 GO:0071157

XM_018057429.1 GO:0070062 GO:0008286 GO:0035335 GO:0006468 GO:0016021 GO:0004725 GO:0043235

XM_018054197.1 GO:0005615 GO:0008021 GO:0017137

XM_018046061.1 GO:0005634 GO:0005815 GO:0097191 GO:0005737 GO:0051901 GO:0045893 GO:0090200 GO:0097193

XM_018058853.1 GO:0034383 GO:0035356 GO:0071400 GO:0042803 GO:0048471 GO:0019915 GO:0055089 GO:0003846 GO:0035336 GO:0030176 GO:0005811 GO:0004144 GO:0042632 GO:0005739 GO:0046339 GO:0019432 GO:0060613

XM_013975149.2 GO:0046872 GO:0005634 GO:0006355 GO:0003700

XM_018062471.1 GO:0043231 GO:0005524 GO:0036376 GO:0021591 GO:0010107 GO:0005391 GO:0015991 GO:0007626 GO:0046872 GO:0030007 GO:0005887 GO:0006883 GO:0010248

XM_005677624.3 GO:0006397 GO:0046872 GO:0005730 GO:0016607 GO:0008380 GO:0019899

XM_018062625.1 GO:0002028 GO:0005524 GO:0051823 GO:0014853 GO:0010657 GO:0008016 GO:0005829 GO:0006874 GO:0033017 GO:0005886 GO:0006998 GO:0031965 GO:0014722 GO:0031307 GO:0050790 GO:0010830 GO:0004674 GO:0018105 GO:0017020

XM_018059790.1 GO:0070507 GO:0021799 GO:0021869 GO:0032956 GO:0090263 GO:0045665 GO:0043015 GO:0019904 GO:0005829 GO:0016021 GO:0007049

XM_018048727.1 GO:0005925 GO:0005794 GO:0015629 GO:0031274 GO:0008360 GO:0005886

XR_001917212.1 GO:0000149 GO:0016323 GO:0005615 GO:0030335 GO:0033194 GO:0035774 GO:0000322 GO:0043197 GO:0070062 GO:0090004 GO:0035749 GO:0031629 GO:0008021 GO:0030027 GO:0048471 GO:0006886 GO:0005768 GO:1902041 GO:0050921 GO:0031201 GO:0005484 GO:0043311 GO:2000010 GO:0045785 GO:0005901 GO:0051024 GO:0060291 GO:0016021 GO:0048278 GO:0016230 GO:0043085 GO:0035493 GO:0043219 GO:0042581 GO:0005802 GO:0009986 GO:0017022 GO:0008284

XM_018054635.1 GO:0030425 GO:0035556 GO:0005737 GO:0006171 GO:0004016 GO:0000166 GO:0016021 GO:0005886 GO:0007188

XM_018045959.1 GO:0070062 GO:0071356 GO:0005102 GO:0071346 GO:0009986 GO:2000484 GO:0016021 GO:0034113

XM_005698486.3 GO:0018345 GO:0008270 GO:0016021 GO:0005789 GO:0019706

XM_018062706.1 GO:0005887 GO:0005242 GO:0034765 GO:0010107

XM_018065322.1 GO:0001701

XM_018066328.1 GO:0000226 GO:0005874 GO:0002230 GO:0008017

XM_018054818.1 GO:0005911 GO:0031514

XM_005674903.3 GO:0016021

XM_005676119.3 GO:0030315 GO:0051602 GO:0005515 GO:0005887 GO:0009897 GO:0034765 GO:0015467 GO:0010107

XM_018051506.1 GO:0007186 GO:0070915 GO:0009986 GO:0030139 GO:0016021

XM_018066509.1 GO:0010875 GO:0032270 GO:0042127 GO:0031625 GO:0000060 GO:0043330 GO:0045638 GO:0007253 GO:0005829 GO:0031663 GO:0032088 GO:0005886 GO:0034142 GO:0035994 GO:0005634 GO:0070427 GO:0042802 GO:0032495 GO:0070431 GO:0008139 GO:0010745 GO:0051059 GO:0045944 GO:0045746 GO:0010888

NM_001285565.1 GO:0005615 GO:0006508 GO:0030163 GO:0004190

XM_018056981.1 GO:0006376 GO:0003723 GO:0005634 GO:0000166 GO:0005737

XM_013971383.2 GO:0005524 GO:0031410 GO:0035556 GO:0046854 GO:0012506 GO:0005911 GO:0005829 GO:0008270 GO:2000785 GO:0042147 GO:0048471 GO:0032288 GO:0031901 GO:0045121 GO:0016308 GO:0034504

XM_005696595.3 GO:0034445 GO:0070062 GO:0034362 GO:0034384 GO:0034366 GO:0034380 GO:0033344 GO:0005543 GO:0005319 GO:0016209 GO:0034375 GO:0034361 GO:0043691 GO:0034365

XM_018062650.1 GO:0005524 GO:0045785 GO:0038033 GO:0005737 GO:0032743 GO:0089700 GO:0001938 GO:0045766 GO:0005654 GO:0051092 GO:1901727 GO:0032757 GO:0030949 GO:0008219 GO:0004697 GO:0046777 GO:0045743 GO:0046872 GO:0032793 GO:0045944 GO:0033138 GO:0070374 GO:2000573 GO:0043536 GO:0050862

XM_018051320.1 GO:0016705 GO:0020037 GO:0005506 GO:0016021 GO:0004497 GO:0055114

XM_005687453.3 GO:0016337 GO:0009566 GO:0044822 GO:0001669 GO:0007286 GO:0060009 GO:0012506 GO:0005829 GO:0005794 GO:0016021

XM_005678182.3 GO:0007186 GO:0030246 GO:0016021 GO:0007166 GO:0004930

XM_005696017.3 GO:0030335 GO:0033588

XM_013965331.2 GO:0016702 GO:0019511 GO:0005783 GO:0005634 GO:0005506 GO:0055114 GO:0031418 GO:0004656

XM_018057891.1 GO:0045892 GO:0000793 GO:0031493 GO:0005654 GO:0042802 GO:0000785 GO:0035064 GO:0045652 GO:0032093 GO:0007088 GO:0008270 GO:0003700 GO:0005886 GO:0016568

XM_018066358.1 GO:0016021 GO:1902616 GO:0005739 GO:0015227

XM_018064699.1 GO:0016020 GO:0003677 GO:0072368 GO:0003714 GO:0051225 GO:0042826 GO:0016580 GO:0035257 GO:0072362 GO:0017053 GO:0046329 GO:0001102 GO:0005876

XM_005680821.3 GO:0016020 GO:0005515

XM_013963609.2 GO:0016323 GO:0031434 GO:0005783 GO:0005874 GO:0031641 GO:0097016 GO:0001658 GO:0042130 GO:0035748 GO:0009898 GO:0002088 GO:0051898 GO:0001935 GO:0070062 GO:0005605 GO:0090004 GO:0032947 GO:0045930 GO:0051660 GO:0060022 GO:0034629 GO:0032147 GO:0008360 GO:0014069 GO:0043268 GO:0048471 GO:0070373 GO:0070830 GO:0008022 GO:0045121 GO:0048704 GO:0019902 GO:0031579 GO:0097025 GO:0043622 GO:0030866 GO:0031594 GO:0048745 GO:0000122 GO:0048608 GO:0002369 GO:0042982 GO:0005923 GO:0030953 GO:0016328 GO:0030838 GO:0042391 GO:0001771 GO:0005634 GO:0015459 GO:0043219 GO:0001772 GO:0030432 GO:0031253 GO:0044325 GO:1903753 GO:0005794 GO:0050680 GO:0033268 GO:0008284

XM_018055100.1 GO:0071456 GO:0070828 GO:0003677 GO:0005634 GO:0006355 GO:0006334 GO:0042127 GO:0097298 GO:0031491 GO:0000786

XM_005692436.3 GO:0043928 GO:0005730 GO:0051607 GO:0035327 GO:0045006 GO:0000178

XM_018040419.1 GO:0008270

XM_005699095.3 GO:0009353 GO:0005763 GO:0055114 GO:0070125 GO:0005743 GO:0006103 GO:0004591 GO:0070124

XR_001918224.1 GO:0055085 GO:0015218 GO:0000002 GO:0016021 GO:0006864 GO:0005739 GO:0051881

XM_005698978.3 GO:0005634 GO:0043565 GO:0005737

XM_013964600.2 GO:0002244 GO:0005634 GO:0016554 GO:0044822 GO:0000166

XM_018045966.1 GO:0009986 GO:0032808 GO:0016021

XM_018054924.1 GO:0003677 GO:0005634 GO:0005737 GO:0046983 GO:0051775 GO:0051879 GO:0005667 GO:0045944 GO:0045739 GO:0060548 GO:0003700

XM_005681602.3 GO:0005768 GO:0005743

XM_018053310.1 GO:0003677 GO:0005634 GO:0005524 GO:0006334 GO:0004842 GO:0008270 GO:0000786 GO:0000209

XM_018054692.1 GO:0032007 GO:0045859 GO:0001822 GO:0005938 GO:0043666 GO:0021766 GO:0047485 GO:0051087 GO:0017148 GO:0021987 GO:0050808 GO:0005886 GO:0008285 GO:0051492 GO:0043379 GO:0042552 GO:0006813 GO:0033596 GO:0055007 GO:0051894 GO:0006407 GO:0032868 GO:0030030 GO:0030027 GO:0002250 GO:0045792 GO:0005884 GO:0046323 GO:0001843 GO:0050821

XM_005677781.3 GO:0005484 GO:0000149 GO:0005793 GO:0000139 GO:0033043 GO:0048280 GO:0045732 GO:0016021 GO:0031201 GO:0006888 GO:0005789 GO:0006887

XM_018059915.1 GO:0050707 GO:0004888 GO:0016021 GO:0002755 GO:0042742 GO:0001819 GO:0034146

XM_013969121.2 GO:0070062 GO:0005929

XM_005675358.3 GO:0090051 GO:0030335 GO:0045747 GO:0047485 GO:0010629 GO:0090168 GO:0019901 GO:0000139 GO:0005829 GO:0044319 GO:0051683 GO:0005886 GO:1903588 GO:0070062 GO:0043406 GO:0043066 GO:0032874 GO:0010628 GO:0042803 GO:0036481 GO:0035023 GO:0033138 GO:0050821 GO:0043149 GO:0008284

XM_013967424.2 GO:0016021

XM_018057914.1 GO:0070062 GO:0003723 GO:0000166 GO:0001556 GO:0006378 GO:0005737

XM_013972521.2 GO:0043123 GO:0016021 GO:0004871

XM_005701733.3 GO:0030133 GO:0016021

XM_005676643.3 GO:0071934 GO:0016021 GO:0015403

XM_018047071.1 GO:0070062 GO:0005886

XM_005676812.3 GO:0006486 GO:0002094

XM_018059440.1 GO:0014902 GO:0030176 GO:0043234 GO:0005246 GO:0002115 GO:0032237

XM_005695891.3 GO:0070062 GO:0016787 GO:0005730 GO:0045944 GO:0005829

XM_018042257.1 GO:0060441 GO:0045186 GO:0030901 GO:0008092 GO:0071896 GO:0045176 GO:0030859 GO:0072205 GO:0005913 GO:0042981 GO:0006461 GO:0008013 GO:0045197

XR_001919531.1 GO:0016020 GO:0044822 GO:0005524 GO:2000623 GO:0004386

XM_018046131.1 GO:0042834 GO:0009253 GO:0016019 GO:0016045 GO:0002221 GO:0008745 GO:0008270 GO:0050830

XM_018038275.1 GO:0072332 GO:0070062 GO:0042802 GO:0031625 GO:0047710 GO:0005829 GO:0032435 GO:0006163

XM_005684482.3 GO:0004809 GO:0030488 GO:0000049

XM_018041424.1 GO:0048008 GO:0014065 GO:0032587 GO:0007283 GO:0005737 GO:0009791 GO:0060021 GO:0030165 GO:0043325 GO:0051898 GO:0048705 GO:0070301 GO:0045184 GO:0070062 GO:0050853 GO:0005654 GO:0031529 GO:0008209 GO:0035264 GO:0035091 GO:0033327 GO:0001553 GO:0008210 GO:0060325

XM_018062263.1 GO:0044822

XM_018039417.1 GO:0001958 GO:0001649 GO:0000978 GO:0005654 GO:0042826 GO:0007512 GO:0046982 GO:0001077 GO:0042803 GO:0005737 GO:0035914 GO:0002062 GO:0045944 GO:0033613

XM_018050607.1 GO:0016021

XM_005675240.3 GO:0051260 GO:0009925

XM_005686914.3 GO:0046983

XM_005685209.3 GO:0005783 GO:0005743

XM_005676432.3 GO:0035094 GO:0001077 GO:0045672 GO:0003705 GO:0043065 GO:0021983 GO:0030544 GO:0007411 GO:0001102 GO:0048145 GO:0035729 GO:0005654 GO:0000978 GO:0036276 GO:0071294 GO:0008361 GO:0045899 GO:0042802 GO:0006468 GO:0045600 GO:0033363 GO:0016032 GO:0001666 GO:0042752 GO:0034670 GO:0000980 GO:0046889 GO:0007595 GO:0010944 GO:0008542 GO:1901215 GO:0005759 GO:0046982 GO:1990090 GO:0071398 GO:1990314 GO:0001190 GO:0007179 GO:0003712 GO:0036120 GO:0030424 GO:1900273 GO:0060430 GO:0014823 GO:0060251 GO:1902065 GO:0007568 GO:0032916 GO:0046887 GO:0007613 GO:0060509 GO:0033762 GO:0035497 GO:0040018 GO:0005719 GO:0050821 GO:0035035 GO:1990589

XM_018042482.1 GO:0031062 GO:0005506 GO:0080111 GO:0070579 GO:0055114 GO:0090310 GO:0043566 GO:0008270 GO:0008284

XM_018057634.1 GO:0016020 GO:0019905 GO:0042113 GO:0005737

XM_018051789.1 GO:0016477 GO:0005634 GO:0030036 GO:0005925 GO:0003382 GO:0005884 GO:0003779 GO:0003334 GO:0005739 GO:0005886

NM_001285602.1 GO:0045892 GO:0048743 GO:0001228 GO:0000978 GO:0005634 GO:0003705 GO:0060415 GO:0046983 GO:0035914 GO:0045944 GO:0045663 GO:0001756 GO:1901741

XM_005676312.2 GO:0007126 GO:0000166 GO:0008494 GO:0045948 GO:0005737

XM_018056297.1 GO:0055085 GO:0005524 GO:0042626 GO:0016021

NM_001285766.1 GO:0070062 GO:0031069 GO:0045109 GO:0005198 GO:0005882 GO:0005737 GO:0007568

XM_018049724.1 GO:0001223 GO:0046872 GO:0005634 GO:0045944 GO:0043565 GO:0007050 GO:0005737 GO:0008285

XM_005684381.3 GO:0070588 GO:0016358 GO:0042802 GO:0030054 GO:0060134 GO:0045471 GO:0004972 GO:0043025 GO:0043005 GO:0017146 GO:0035235 GO:0016594 GO:0005234 GO:0005262 GO:0045211

XM_005682720.3 GO:0036112 GO:0001649 GO:0000038 GO:0008209 GO:0036111 GO:0005102 GO:0060009 GO:0042803 GO:0006635 GO:0008210 GO:0005739 GO:0003857 GO:0016508 GO:0005778 GO:0044594

XM_018050688.1 GO:0018105 GO:0004674 GO:0005524

XM_018056749.1 GO:0007186 GO:0016021 GO:0005886 GO:0004930

XM_013970971.2 GO:0005654 GO:0005737

XM_018050522.1 GO:0003677 GO:0005634 GO:0006355

XM_018046936.1 GO:0016477 GO:0007010 GO:0008360 GO:0005737

XM_018060378.1 GO:0030335 GO:0016301 GO:0005622 GO:0048015 GO:0010628 GO:0046854

NM_001314333.1 GO:0005634 GO:0030154 GO:0007275 GO:0007283 GO:0001520

XM_005680510.2 GO:0016021 GO:0061588 GO:0046983

XM_018055222.1 GO:0097109 GO:0005783 GO:0051965 GO:0031982 GO:0043025 GO:0033130 GO:0007269 GO:0061178 GO:0051968 GO:0005246 GO:0042734 GO:0016021 GO:0097116 GO:2000821 GO:0031965 GO:0050839 GO:0035176 GO:0060134 GO:2000463 GO:0042297 GO:0009986 GO:0043234 GO:0097118 GO:0030534 GO:0050885 GO:0071625 GO:0097119 GO:0090129

XM_018045131.1 GO:0016020 GO:0005856 GO:0005634 GO:0004715 GO:0005524 GO:0005925 GO:0019903 GO:0031625 GO:0035556 GO:0005737 GO:0046677 GO:0038110 GO:0018108

XM_018057119.1 GO:0008283 GO:0005730 GO:0006260 GO:0042802 GO:0006974 GO:0019899 GO:0005737

XM_005679782.3 GO:0046872 GO:0016021 GO:0008454 GO:0006491 GO:0000139

XM_018058646.1 GO:0007186 GO:0016525 GO:0016021 GO:0007166 GO:0051965 GO:0014069 GO:0004930

XM_018049863.1 GO:0009411 GO:0006283 GO:0016567 GO:0005694 GO:0000993

NM_001285646.1 GO:0008083 GO:0045666 GO:0060113 GO:0060235 GO:2000137 GO:0030513 GO:0048392 GO:0032092 GO:0070700 GO:0072205 GO:0060592 GO:0008201 GO:0072192 GO:0007224 GO:0000186 GO:0002320 GO:0010453 GO:0072104 GO:0007182 GO:0045843 GO:0060363 GO:0045662 GO:0072015 GO:0070244 GO:0045944 GO:0048663 GO:0002043 GO:0003130 GO:0061151 GO:0072101 GO:0021978 GO:0000122 GO:0033088 GO:0072125 GO:0072097 GO:0010862 GO:0003139 GO:0001938 GO:0042056 GO:0003337 GO:0001958 GO:2000105 GO:0072138 GO:0045669 GO:0045603 GO:0060687 GO:0072193 GO:0030218 GO:0060395 GO:0072200 GO:0060686 GO:0045839 GO:0050680 GO:2000007 GO:0039706 GO:0060272 GO:0090184 GO:0060433 GO:0005615 GO:0061155 GO:0060502 GO:0043407 GO:0007500 GO:0010595 GO:2000005 GO:0005160 GO:0032967 GO:0043065 GO:0009791 GO:0021983 GO:0005125 GO:0035116 GO:0021904 GO:0072161 GO:0010159 GO:0007281 GO:0035990 GO:0042487 GO:0060197 GO:0001843 GO:0050918 GO:0060684 GO:0051150 GO:0060440 GO:0003014 GO:0042733 GO:0003323 GO:0061149 GO:0055020 GO:0030225 GO:0071893 GO:0061036 GO:0045606 GO:0060391 GO:0035993 GO:0005578 GO:0061047 GO:0048701 GO:0030501 GO:0032331 GO:0048661 GO:0060449 GO:0070374 GO:0048286 GO:0043401 GO:0009948 GO:0030224 GO:0060503

XM_018044464.1 GO:0007010 GO:0008270 GO:0022604

XM_005676354.3 GO:0006457 GO:0003755 GO:0000413 GO:0071013

XM_018044982.1 GO:0005615 GO:0005622 GO:0035023 GO:0043547 GO:0005089

XM_018059478.1 GO:0071108 GO:0004843 GO:0005634 GO:0007067 GO:0019901 GO:0006511 GO:0004197 GO:0051301 GO:0000082 GO:0035871

XM_018052129.1 GO:0070062 GO:0031410 GO:0017137

XM_005677621.3 GO:0005634 GO:0006355 GO:0043234 GO:0043486 GO:0035019

XM_018038237.1 GO:0070062 GO:0035267 GO:0000812 GO:0006281 GO:0005524 GO:0006355 GO:0043968 GO:0031011 GO:1903146 GO:0032508 GO:0071339 GO:0043967 GO:0005794 GO:0043141 GO:0006310

XM_018047249.1 GO:0042119 GO:0048384 GO:0042800 GO:0030854 GO:0051568 GO:0006306 GO:0030218 GO:0002446 GO:0045893 GO:0071300 GO:0045171 GO:0003713 GO:0070688 GO:0008270 GO:0019899

XM_018040913.1 GO:0030308 GO:0045116 GO:0051443 GO:0010332 GO:0031624 GO:0048471 GO:0010225 GO:0043065 GO:0000151 GO:0005886 GO:0032182 GO:0097602

XM_005685024.3 GO:0006614 GO:0005786 GO:0008312

XM_005683008.3 GO:0005634 GO:0000166 GO:0008270 GO:0003676

XM_018063219.1 GO:0071376 GO:0003707 GO:0008344 GO:0046982 GO:0001077 GO:0035259 GO:0042053 GO:0042416 GO:0000122 GO:0009791 GO:0000979 GO:0008270 GO:0031668 GO:2001234 GO:0043085 GO:0051866 GO:0005654 GO:0043524 GO:0071542 GO:0021986 GO:0004879 GO:0001666 GO:0030522 GO:0001975 GO:0001764 GO:0034599 GO:0045444 GO:0045944 GO:0021952 GO:0043401 GO:0043576 GO:0042551

XM_005678655.2 GO:0006457 GO:0003755 GO:0044822 GO:0006355 GO:0071013 GO:0000166 GO:0000413 GO:0045070

XM_018039403.1 GO:0045666 GO:0003714 GO:0046982 GO:0001093 GO:0043621 GO:0001077 GO:0000122 GO:0003682 GO:0005667 GO:0016021 GO:1900746 GO:0065004 GO:0005634 GO:0000978 GO:0070888 GO:0016525 GO:0001011 GO:0045944 GO:0001087 GO:0042118 GO:0006367

XR_001918964.1 GO:0000287 GO:0004652 GO:0003723 GO:0005654 GO:0000398 GO:0005524 GO:0006369 GO:0006378 GO:0005737 GO:0031440 GO:0005515 GO:0016021 GO:0030145

XM_018062041.1 GO:0005665 GO:0003677 GO:0001055 GO:0015630 GO:0005737 GO:0006366 GO:0046983

XM_018066401.1 GO:0000287 GO:0072562 GO:0001917 GO:0031594 GO:0045494 GO:0042803 GO:0001750 GO:0005178 GO:0071318 GO:0005829 GO:0005509 GO:0007204 GO:0032420 GO:0042383 GO:0005927

XM_018042481.1 GO:0031062 GO:0005506 GO:0080111 GO:0070579 GO:0055114 GO:0090310 GO:0043566 GO:0008270 GO:0008284

XM_018043737.1 GO:0016020 GO:0070062 GO:0005654 GO:0006284 GO:0044822 GO:0006513 GO:0005737 GO:0004842 GO:0016874 GO:0016574 GO:0000209

XM_013970019.2 GO:0046835 GO:0006003 GO:0005524 GO:0006000 GO:0006096 GO:0003873 GO:0019901

XM_018048449.1 GO:0005524 GO:0042127 GO:0006468 GO:0008074 GO:0035556 GO:0009636 GO:0006182 GO:0004672 GO:0004016 GO:0016021 GO:0005789 GO:0005525 GO:0005886 GO:0004383

XM_005696700.3 GO:0046872 GO:0006355 GO:0005622 GO:0003700 GO:0003676

XM_018050324.1 GO:0070498 GO:0001077 GO:0005737 GO:0000122 GO:0090090 GO:0061418 GO:0009749 GO:0072303 GO:0005654 GO:0032868 GO:0071504 GO:0030509 GO:0071480 GO:0071506 GO:0033233 GO:0035914 GO:0046872 GO:0045944 GO:0000977 GO:0030217 GO:0042981 GO:0035035

XM_018065346.1 GO:0000978 GO:0005654 GO:0006355 GO:0000790 GO:0006337 GO:0043044 GO:0016514 GO:0000980 GO:0031492 GO:0016568

XM_005684651.3 GO:0005654 GO:0044822 GO:0005737

XM_018059131.1 GO:0016020 GO:0000922 GO:0035371 GO:0005813 GO:0051297 GO:0000930 GO:0007051 GO:0030951

XM_018044975.1 GO:0046580 GO:0043547 GO:0005737 GO:0015278 GO:0046872 GO:0031235 GO:0051209 GO:0005096

XM_005677146.3 GO:0043154 GO:0044297 GO:0005524 GO:0005739 GO:0009408 GO:0007339 GO:0002199

XM_018056142.1 GO:0006357 GO:0001104 GO:0016592

XM_018057203.1 GO:0005911

XM_018056768.1 GO:0005634 GO:0006355 GO:0003700 GO:0005737

XR_001919951.1 GO:0005634 GO:0043161 GO:0016567

XM_018061114.1 GO:0048266 GO:0005654 GO:0005925 GO:0043547 GO:0032403 GO:0005096

XM_005678629.3 GO:0000978 GO:0005654 GO:0016602 GO:0045944 GO:0046982 GO:0001077 GO:0032993

XM_018050457.1 GO:0005730 GO:0005829

XM_018058063.1 GO:0016021

XM_005674851.3 GO:0016021

XM_018049919.1 GO:0031965 GO:0005654 GO:0005813 GO:0005737

XM_018060849.1 GO:0016020 GO:0007269 GO:0005544 GO:0008021

XM_005696395.3 GO:0005759 GO:0046872 GO:0009791 GO:0031419 GO:0004494 GO:0050667 GO:0072341

XM_005675899.3 GO:0003723 GO:0000398 GO:0071006 GO:0005794 GO:0016607 GO:0071013

XM_005676544.3 GO:0032981 GO:0033617 GO:0034551 GO:0005524 GO:0005743 GO:0016021

XM_018039085.1 GO:0000166 GO:0008597 GO:0016021 GO:0050790 GO:0070884 GO:0005737

XM_018047059.1 GO:2000678 GO:0042994 GO:1901841 GO:0035774 GO:0046982 GO:0010459 GO:0030018 GO:0005829 GO:0086004 GO:0005886 GO:0042584 GO:0070062 GO:0002020 GO:0005654 GO:1901844 GO:1901077 GO:0042802 GO:0070491 GO:0051281 GO:0005509 GO:0005789 GO:0060315

XM_018051619.1 GO:0010744 GO:0007258 GO:0071222 GO:0071356 GO:0032308 GO:0034644 GO:0010770 GO:0071347 GO:0071363 GO:0014075 GO:0031396 GO:0042493 GO:0005654 GO:0009612 GO:0042752 GO:2001235 GO:0046686 GO:0004705 GO:0006626 GO:0006919 GO:0045429 GO:0046328 GO:0031394 GO:0071803 GO:0005524 GO:0009636 GO:0045893 GO:0051770 GO:0005829 GO:0031175 GO:0001934 GO:0005739 GO:0001836 GO:0008656 GO:0031435 GO:0051090 GO:0007417 GO:0038095 GO:0008134 GO:0032722

XM_005695598.3 GO:0070062 GO:0046872 GO:0005634 GO:0006355 GO:0003700 GO:0003676

XM_018058484.1 GO:0061025 GO:0043130 GO:0005634 GO:0043161 GO:0005829 GO:0007030 GO:0031468

XM_013970631.2 GO:0045859 GO:0031588 GO:0005634 GO:0007165 GO:0004672 GO:0006633 GO:0010628

XM_018061239.1 GO:0016020 GO:0010494 GO:0034063 GO:0005654 GO:0044822 GO:0010603 GO:0005154 GO:0005802 GO:0048471 GO:0008022 GO:0002091 GO:0005844

XM_018050374.1 GO:0048471 GO:0000139 GO:0006886 GO:0050714 GO:0045714 GO:0030127 GO:0008270 GO:2000189 GO:0005789 GO:0006888

XM_018063292.1 GO:0042995 GO:0032836 GO:0035418 GO:0036057 GO:0044062 GO:0016021 GO:0030838 GO:0005886 GO:0070062 GO:0005622 GO:0007520 GO:0007254 GO:0007155 GO:0072015 GO:0017022 GO:0007519

XM_018060568.1 GO:0014850 GO:0005634 GO:0006355 GO:0005737

XM_018053504.1 GO:0070062 GO:0003677 GO:0005634 GO:0005737 GO:0046872 GO:1901532 GO:1902035 GO:0006919 GO:0019899

XM_018048466.1 GO:0017017 GO:0005654 GO:0035335 GO:0004725 GO:0000188 GO:0005737

XM_013964330.2 GO:0030246

XM_018050102.1 GO:0003677 GO:0046872 GO:0005634 GO:0006355 GO:0007275

XM_018051778.1 GO:0005615 GO:0051603 GO:0042470 GO:0043394 GO:0005730 GO:0005739 GO:0070062 GO:0046697 GO:0005764 GO:0050790 GO:0048471 GO:0005518 GO:0004197 GO:0097067 GO:0046718 GO:0030855 GO:0030574

XM_018049990.1 GO:0044763 GO:0044421

XM_018042432.1 GO:0044325 GO:0042391

XM_018046615.1 GO:0050702 GO:0071404 GO:0016324 GO:0031623 GO:0070543 GO:0031526 GO:0007166 GO:1990000 GO:1900227 GO:0016021 GO:0070508 GO:0044539 GO:0050909 GO:0034197 GO:0071726 GO:0007155 GO:0045121 GO:0030299 GO:0005794 GO:0006955 GO:0007204 GO:0070542 GO:0070374 GO:2000505

XM_005677123.3 GO:0010763 GO:0005524 GO:0030199 GO:0030500 GO:0051091 GO:0005887 GO:0045860 GO:0018108 GO:0070062 GO:0005925 GO:0048146 GO:0038062 GO:0003416 GO:0090091 GO:0045669 GO:0046777 GO:0035988 GO:0038063 GO:0005518

XM_018043787.1 GO:0003677 GO:0006355 GO:0005669 GO:0006352

XM_018062511.1 GO:0030154 GO:0001525 GO:0005576 GO:0006935

XM_018054146.1 GO:0042446 GO:0016174 GO:0020037 GO:0042335 GO:0016324 GO:0051591 GO:0055114 GO:0006979 GO:0005509 GO:0004601 GO:0006590 GO:0016021 GO:0019221 GO:0042744 GO:0050665

XM_018041421.1 GO:0048008 GO:0014065 GO:0032587 GO:0007283 GO:0005737 GO:0009791 GO:0030165 GO:0043325 GO:0060021 GO:0051898 GO:0048705 GO:0070301 GO:0045184 GO:0070062 GO:0050853 GO:0005654 GO:0031529 GO:0008209 GO:0035264 GO:0033327 GO:0001553 GO:0008210 GO:0060325

XM_018065212.1 GO:0071819 GO:0000124 GO:0016578 GO:0030374 GO:0008270 GO:0045893 GO:0016568

XM_005688102.3 GO:0055114 GO:0016491

XM_018047400.1 GO:0051015 GO:0019722 GO:0014069 GO:0030175 GO:0030864 GO:0007015 GO:0031175 GO:0043197

XM_005698780.3 GO:0035307 GO:0043547 GO:0032587 GO:0051497 GO:0005901 GO:0030864 GO:0005829 GO:1900119 GO:0008285 GO:0005634 GO:0005925 GO:0030336 GO:0008360 GO:0051895 GO:0042169 GO:0008289 GO:0006919 GO:0035024 GO:0005096

XM_018039614.1 GO:0005634 GO:0007165 GO:0009409 GO:0060612 GO:0006366 GO:0045893 GO:0046872 GO:0050873 GO:0000987 GO:0033613 GO:0003700

XM_018064267.1 GO:0005615 GO:0000910 GO:0042803 GO:1903452 GO:0048471 GO:0030496 GO:0003407 GO:0055038 GO:0030139 GO:0032154 GO:0030306 GO:0017137

XM_018042536.1 GO:0005829

XM_018049588.1 GO:0015630 GO:0003676

XM_018044457.1 GO:0070062 GO:0044598 GO:0005739 GO:0019119 GO:0044597

XM_018046160.1 GO:0061484 GO:0044822 GO:0031519 GO:0005671 GO:0007339

XM_018050918.1 GO:0042384 GO:0001675 GO:0006357 GO:0000978 GO:0005634 GO:0003700 GO:0005737

XM_005686841.3 GO:0031965

XM_005674994.3 GO:0005634 GO:0043565 GO:0006367 GO:0005737

XM_018050199.1 GO:0043402 GO:0000978 GO:0005815 GO:0005654 GO:0001077 GO:0042921 GO:0045944 GO:0005819 GO:0008270 GO:0005739 GO:0016568 GO:0038051 GO:1990239

XM_005686725.3 GO:0043547 GO:0005096

XM_018049424.1 GO:0005634 GO:0006355 GO:0008270 GO:0016021 GO:0003700

XM_005689839.2 GO:0060765 GO:0004842 GO:0005730 GO:0050681 GO:0005794 GO:0008270 GO:0051865 GO:0035035

XM_018053141.1 GO:0005654 GO:0042787 GO:0042803 GO:0005737 GO:0004842 GO:0005730 GO:0031463 GO:0005886

XM_005700681.3 GO:0004896 GO:0016021 GO:0019221

XM_018060697.1 GO:0005634 GO:0045111 GO:0051721 GO:0005737

XM_018060785.1 GO:0012505 GO:0005954 GO:0005524 GO:0004683 GO:0006468 GO:0005886

XM_018048070.1 GO:0010535 GO:0032733 GO:0050829 GO:0032693 GO:0032819 GO:0032740 GO:0045672 GO:2000330 GO:0032760 GO:0043382 GO:0032735 GO:0042520 GO:0002827 GO:0070743 GO:0042104 GO:0032729 GO:0042510 GO:0090023 GO:0045519 GO:0005125 GO:0051142 GO:0032725 GO:0042523 GO:0042517 GO:0045944 GO:0001916 GO:0002230

XM_018056045.1 GO:0005524 GO:0006165 GO:0021591 GO:0004017 GO:0004550 GO:0009142 GO:0036126 GO:0005930 GO:0004127

XM_013970552.2 GO:0010226 GO:0034220 GO:0031623 GO:0030054 GO:0004971 GO:0032281 GO:0043025 GO:0035235 GO:0005246 GO:0005234 GO:0043197 GO:0030165 GO:0045211 GO:0045184 GO:0050806 GO:0051966 GO:0042802 GO:0051262 GO:0043198 GO:0005789 GO:0015277 GO:0001919

XM_018057792.1 GO:0046972 GO:0043984 GO:0031965 GO:0043981 GO:0043995 GO:0043996 GO:0005737 GO:0071339 GO:0043982 GO:0008270 GO:0000123

XM_018055506.1 GO:0042552 GO:0051716 GO:0043547 GO:0005085 GO:0001541 GO:0006413 GO:0003743 GO:0005851 GO:0014003

XM_005699864.3 GO:0005980 GO:0030170 GO:0008184 GO:0005737

XM_018057979.1 GO:0005634 GO:0048306

XM_018062273.1 GO:0044822

XR_001919474.1 GO:0000122 GO:0003677 GO:0046872 GO:0005634 GO:0043392 GO:0045893 GO:0001078

XM_018056551.1 GO:0018345 GO:0008270 GO:0016021 GO:0019706 GO:0005886

XM_005682718.3 GO:0045727 GO:0045793

XM_005691391.3 GO:0044822

XM_005678616.3 GO:0045776 GO:0070062 GO:0031284 GO:0007589 GO:0007588 GO:0030250 GO:0001750

XM_013965818.2 GO:0030335 GO:0048008 GO:0030054 GO:0010762 GO:0044331 GO:0005737 GO:0035426 GO:0036119 GO:0070102 GO:0031234 GO:0050904 GO:0033007 GO:0034446 GO:0038028 GO:0015630 GO:0000790 GO:0030027 GO:0046777 GO:0036006 GO:0008289 GO:0008157 GO:0000278 GO:0005524 GO:0034614 GO:0042503 GO:0032496 GO:0038109 GO:0001932 GO:0010591 GO:0015629 GO:0030838 GO:0006935 GO:0031532 GO:0000226 GO:0004715 GO:0051092 GO:0005154 GO:0038095 GO:0042058 GO:0008284

XM_005683393.3 GO:0016021 GO:0000139

XM_018039118.1 GO:0034198 GO:0031625 GO:0010629 GO:0030890 GO:0033158 GO:0000307 GO:0090398 GO:0019912 GO:0005829 GO:2000278 GO:0008285 GO:0071493 GO:0070557 GO:0030332 GO:0030308 GO:0071850 GO:2000379 GO:0048146 GO:0042771 GO:0090399 GO:0045736 GO:0004861 GO:0071479 GO:0000086 GO:0006977 GO:0043068 GO:0007265

XM_018055433.1 GO:0000978 GO:0005634 GO:0035855 GO:0001525 GO:0002089 GO:0046982 GO:0045638 GO:0001077 GO:0003705 GO:0045665 GO:0007626 GO:0045944 GO:0060216 GO:0005667 GO:0003682

XM_018051230.1 GO:0030425 GO:0032353 GO:0007214 GO:0008331 GO:0005737 GO:0043025 GO:0021750 GO:0030644 GO:0000096 GO:0006006 GO:0021679 GO:0043524 GO:0042133 GO:0007628 GO:0035249 GO:0021522 GO:0050885 GO:0005891 GO:0070588 GO:0048791 GO:0019226 GO:0014056 GO:0016049 GO:0007274 GO:0007416 GO:0021590 GO:0050770 GO:0043113 GO:0005634 GO:0050883 GO:0060024 GO:0034765 GO:0014051 GO:0048813 GO:0048266 GO:0021702 GO:0007204 GO:0051899 GO:0019905 GO:0017158

XM_018052438.1 GO:0042384 GO:0016020 GO:0005814 GO:0000226 GO:0090316 GO:0007052 GO:0034451

XR_310363.2 GO:0005840 GO:0005743 GO:0070124 GO:0070125

XM_005680647.3 GO:0005524 GO:0002039 GO:0015630 GO:0035507 GO:0006468 GO:0035556 GO:0005737 GO:0005730 GO:0004674 GO:2000772 GO:0030155

XM_005701295.3 GO:0004331 GO:0005524 GO:0006000 GO:0042802 GO:0003873 GO:0043540 GO:0046835 GO:0006003 GO:0016311

XM_018055751.1 GO:0010259 GO:0003707 GO:0042445 GO:2000020 GO:0009888 GO:0003705 GO:0003682 GO:0008270 GO:0051457 GO:0090575 GO:0030154 GO:0004879 GO:0005543 GO:0030522 GO:0045944 GO:0001553 GO:0030325 GO:2000195 GO:0000980 GO:0043401 GO:0019899

XM_005683051.3 GO:0005730 GO:0006364 GO:0042273 GO:0005737

XM_018054641.1 GO:0007200 GO:0008528 GO:0001632 GO:0005887 GO:0007218 GO:0006954

XM_005683688.3 GO:0016020 GO:0042147 GO:0043547 GO:0017112 GO:0005829 GO:1903363 GO:0034066 GO:0017137

XR_001917157.1 GO:0005355 GO:0016021

XM_005680565.3 GO:0001537 GO:0016051 GO:0050659 GO:0030206 GO:0002063 GO:0042127 GO:0042733 GO:0036342 GO:0033037 GO:0009791 GO:0030512 GO:0016021 GO:0047756 GO:0007585 GO:0048703 GO:0043066 GO:0048589

XM_018048375.1 GO:0005925

XM_018040022.1 GO:0048188 GO:0000166 GO:0042800 GO:0051568 GO:0003676 GO:0008013

XM_018049197.1 GO:0005524 GO:0043194 GO:0031594 GO:0019871 GO:0043025 GO:0016529 GO:1901897 GO:0003254 GO:0060048 GO:0004683 GO:2000650 GO:0005516 GO:0034704 GO:0014704 GO:0051259 GO:0060341 GO:0030315 GO:0005634 GO:0006816 GO:0042803 GO:0046777 GO:0010613 GO:0018107 GO:0044325 GO:0018105 GO:0000082 GO:0031432

XM_018061730.1 GO:0018146 GO:0006044 GO:0001517 GO:0005975 GO:0016021 GO:0000139

XM_018047900.1 GO:0005509

NM_001285734.1 GO:0006886 GO:0030126 GO:1901998 GO:0006890 GO:0006891 GO:0000139

XM_018056666.1 GO:0000124 GO:0035948 GO:0003712

XM_013976542.2 GO:0005654 GO:0005524 GO:0008280 GO:0003682 GO:0000724 GO:0007064

XM_018051199.1 GO:0008063 GO:0032496 GO:0001960

XM_018042309.1 GO:0051533 GO:0035690 GO:0034097 GO:0035774 GO:0017156 GO:0031987 GO:0046983 GO:0001915 GO:0030018 GO:0008144 GO:0006470 GO:0033173 GO:0005516 GO:0005955 GO:0030315 GO:0035176 GO:0007507 GO:0006468 GO:0043029 GO:0045944 GO:0005509 GO:0030346 GO:0001946 GO:0030217 GO:0033192

XM_005687492.3 GO:0008593 GO:0007155 GO:0030198 GO:0009888 GO:0008201 GO:0005802 GO:0005578

XM_018051017.1 GO:0016324 GO:0042802 GO:0031410 GO:0016055 GO:0005923 GO:0055037

XM_005697051.3 GO:0006281 GO:0000077 GO:0000014

XM_018040230.1 GO:0035267 GO:0000812 GO:0016310 GO:1903506 GO:0006281 GO:0016578 GO:0016301 GO:0043968 GO:0033276 GO:0043967 GO:0005794 GO:0030914 GO:0003712

XR_001918534.1 GO:0006457 GO:0003755 GO:0000413

XM_018039425.1 GO:1990050 GO:0015914 GO:0005758

XM_005683737.3 GO:0098655 GO:0050951 GO:0016048 GO:0005227 GO:0016021 GO:0051262

XM_018064739.1 GO:0004028 GO:0005783 GO:0050061 GO:0046577 GO:0005777 GO:0055114 GO:0033306 GO:0006081 GO:0016021 GO:0052814 GO:0070062 GO:0006714 GO:0007417 GO:0004030 GO:0008544 GO:0005743 GO:0007422

XM_005679497.3 GO:0030014 GO:0044822 GO:0000166 GO:0051865 GO:0008270 GO:0004842

XM_018055398.1 GO:0032781 GO:0001671 GO:0006950 GO:0051087

XM_018057216.1 GO:0005654 GO:0007368 GO:0007507 GO:0036158 GO:0021591 GO:0005930 GO:0097546 GO:0003356

XM_005701516.2 GO:0016021

XM_018061334.1 GO:0005856 GO:0030276 GO:0072583 GO:0006915 GO:0035091 GO:0048471 GO:0003779 GO:0005905 GO:0030665

XM_018050814.1 GO:0030032 GO:1903676 GO:0005840 GO:0005783 GO:0042102 GO:1903679 GO:0012506 GO:0071074 GO:0004860 GO:1990441 GO:0005911 GO:0000164 GO:0051707 GO:0030838 GO:0005886 GO:1902237 GO:0033137 GO:0030971 GO:0070262 GO:0019904 GO:0045944 GO:0060548 GO:0006930

XM_005681418.3 GO:0003677 GO:0005654 GO:0005524 GO:0070933 GO:0004386 GO:0000729 GO:0035861 GO:0051304 GO:0043044 GO:0000018 GO:0000792 GO:0070932 GO:0043596 GO:0016568

XM_018047183.1 GO:0045892 GO:0005654 GO:0050434 GO:0030111 GO:0007257 GO:0045893 GO:0005730 GO:0005794 GO:0030957 GO:0030332 GO:0042308

XM_005678756.2 GO:0048483 GO:0032868 GO:0042755 GO:0040014 GO:0002021 GO:0001894 GO:0005179 GO:0006112 GO:0045444 GO:0005576 GO:0009749 GO:0006629

XR_311185.3 GO:0016031 GO:0031012 GO:0031307 GO:0030943 GO:0005742 GO:0015266 GO:0070096 GO:0044233 GO:0051082 GO:0015450 GO:0030150

XM_013970610.2 GO:0016567 GO:0005622 GO:0035556

XM_018042056.1 GO:0044822

XM_018054660.1 GO:0007169 GO:0009986 GO:0016021 GO:0018108 GO:0004714

XM_005686847.3 GO:0004198 GO:0005654 GO:0006508 GO:0005509 GO:0005737

XM_018044783.1 GO:0005524 GO:0004672 GO:0006468

XM_018063710.1 GO:0000166 GO:0060213 GO:0035195 GO:0003676

XM_005675860.2 GO:0005524 GO:0006298 GO:0030983

XM_005677639.3 GO:0070062 GO:0005654

XM_005676913.3 GO:0016042 GO:0005509 GO:0005576 GO:0004623

XM_018056613.1 GO:0005634 GO:0000922 GO:0005524 GO:0005874 GO:0005813 GO:0007283 GO:0008017 GO:0005737 GO:0007067 GO:0030496 GO:0051301 GO:0008568 GO:0051013 GO:0031122

XM_018042042.1 GO:0004252 GO:0006508 GO:0016021 GO:0005886

NM_001285711.1 GO:0003796 GO:0008152

XM_013975445.2 GO:0008168 GO:0032259

XM_018047653.1 GO:0009952 GO:0005654 GO:0006355 GO:0048706 GO:0043565 GO:0003700 GO:0005737

XM_018063921.1 GO:0043231 GO:0055085 GO:0005524 GO:0006869 GO:0042626 GO:0016021

XM_018050813.1 GO:0000122 GO:0000978 GO:0005730 GO:0006260 GO:0045944 GO:0001077 GO:0042475

XM_018045150.1 GO:0051015 GO:0031532 GO:0034446 GO:0042805 GO:0001725 GO:0070830 GO:0031175 GO:0005923 GO:0008270 GO:0031005 GO:0032456 GO:0030041 GO:0055037 GO:0005886 GO:0017137

XM_005697186.3 GO:0051444 GO:0060412 GO:0010717 GO:0043433 GO:0031625 GO:0032436 GO:0005737 GO:0005913 GO:0003700 GO:0032925 GO:0033137 GO:0005654 GO:0034629 GO:0055010 GO:0055117 GO:0005518 GO:0070411 GO:0010801 GO:0060394 GO:0010944 GO:0030514 GO:0048185 GO:0035556 GO:0022409 GO:0000122 GO:0005730 GO:0005667 GO:0030512 GO:0034616 GO:0034713 GO:0030617 GO:0005813 GO:0031398 GO:0044212 GO:0016342 GO:0046872 GO:0048844 GO:0034333 GO:0001657 GO:0050821 GO:0008013

XM_018054611.1 GO:0005790 GO:0043198 GO:0007612 GO:0050885 GO:0030314 GO:0048167

XM_018061997.1 GO:0005634 GO:0006355 GO:0043565

XM_018043286.1 GO:0046872 GO:0004527 GO:0044822 GO:0000467

XM_018061765.1 GO:0010468 GO:0032481 GO:0043069 GO:0035556 GO:0002092 GO:0006661 GO:0032496 GO:0032959 GO:0005829 GO:0004871 GO:0005886 GO:0070062 GO:0050853 GO:0009395 GO:0004435 GO:0050852 GO:0002316 GO:0032237 GO:0051209

XR_001917135.1 GO:0032733 GO:0032743 GO:0014070 GO:0009897 GO:0032713 GO:0016021 GO:0043372

XM_018050353.1 GO:0030291 GO:0001740 GO:0045815 GO:0000182 GO:0000784 GO:0046982 GO:0010385 GO:0019901 GO:0000122 GO:0061086 GO:0000979 GO:0005730 GO:0019216 GO:0051572 GO:0071169 GO:0016568 GO:0000786 GO:0000793 GO:0070062 GO:1901837 GO:0071901 GO:0006334 GO:0045618 GO:0007549 GO:0000790 GO:0035098 GO:0033128 GO:0045814 GO:1902750 GO:0005721 GO:0031492

XM_018064907.1 GO:1902237 GO:0043433 GO:0031625 GO:0016607 GO:0042787 GO:0001085 GO:0005737 GO:0042593 GO:0019005 GO:2000676 GO:0000122 GO:0043161 GO:0030162 GO:0031463

XM_018057377.1 GO:0007155 GO:0043616 GO:0005925 GO:0005829 GO:0051546 GO:0090162 GO:0031941

XM_005693204.3 GO:0005779 GO:0008022 GO:0016558 GO:0008270

XM_013973812.2 GO:0046872 GO:0003676

XM_018064253.1 GO:0019725 GO:0097345 GO:0007264 GO:0047497 GO:0005509 GO:0031307 GO:0005525 GO:0003924

XM_005681112.3 GO:0003899 GO:0003677 GO:0005813 GO:0005666 GO:0006383

XM_005690627.3 GO:0008168 GO:0032259

XM_018067041.1 GO:0019285 GO:0008812 GO:0050660 GO:0005743 GO:0055114

XM_018061231.1 GO:0032467 GO:0016020 GO:0005524 GO:0006468 GO:0005622 GO:0035556 GO:0097110 GO:0046872 GO:0004674 GO:0030165

XM_005690536.3 GO:0006509 GO:0005813 GO:0035556 GO:0004190 GO:0000139 GO:0005887 GO:0016485 GO:0005637 GO:0007219 GO:0000776 GO:0005789 GO:0043085

XM_018044754.1 GO:0016020 GO:0005827 GO:0051298 GO:0000923 GO:0005816 GO:0005200 GO:0051011 GO:0008275 GO:0005814 GO:0043015 GO:0007126 GO:0090307 GO:0051415 GO:0031122

XM_005700786.3 GO:0030425 GO:0005654 GO:0044822 GO:0070417 GO:0045727 GO:0005737 GO:0005730 GO:0000166 GO:0043023 GO:0009631

XM_018066168.1 GO:0016021

XM_013973872.2 GO:0005759 GO:0046872 GO:0009791 GO:0031419 GO:0004494 GO:0050667 GO:0072341

XM_018065037.1 GO:0003723 GO:0047485 GO:0022008 GO:0017053 GO:0043044 GO:0008080 GO:0071565 GO:0045892 GO:0005654 GO:0000978 GO:0000790 GO:0016922 GO:0006337 GO:0071564 GO:0000980 GO:0016514 GO:0031492

XM_005686653.3 GO:0070062 GO:0008146 GO:0051923

XM_018066805.1 GO:0061470 GO:0046982 GO:0048745 GO:0002639 GO:0003705 GO:0072619 GO:0000122 GO:0003682 GO:0060043 GO:0005634 GO:0000978 GO:0002329 GO:0008045 GO:0045214 GO:0002053 GO:0001701 GO:0021517 GO:0042803 GO:0033152 GO:1901250 GO:0045944 GO:2000727 GO:0007519 GO:0050679

XM_018044169.1 GO:0007528 GO:0005605 GO:0005201 GO:0005587 GO:0031594

XM_018049065.1 GO:0035235 GO:0034220 GO:0005234 GO:0016021 GO:0030054 GO:0015277 GO:0045211

XM_018046687.1 GO:0030246 GO:0004653 GO:0005112 GO:0016021 GO:0018243 GO:0000139

XM_013963900.1 GO:0031100 GO:0000781 GO:0045471 GO:0007067 GO:0032355 GO:0004693 GO:0097124 GO:0015030 GO:0002088 GO:0016572 GO:0030332 GO:0000793 GO:0042493 GO:0006813 GO:0005815 GO:0007099 GO:0097134 GO:0005768 GO:0046686 GO:0060968 GO:0018105 GO:0000082 GO:0051726 GO:0000806 GO:0005524 GO:0000805 GO:0045893 GO:0009636 GO:0051602 GO:0035173 GO:0005667 GO:0005829 GO:0051321 GO:0006281 GO:0051591 GO:0097123 GO:0097135 GO:0032869 GO:0046872 GO:0032298 GO:0051301 GO:0007265 GO:0032403 GO:0008284

XM_005701740.3 GO:0046872 GO:0005634 GO:0008152 GO:0004802 GO:0005737

XM_005680717.3 GO:0016021

XM_018055548.1 GO:0003677 GO:0000791 GO:0006349 GO:0007283 GO:0005737 GO:0001741 GO:0000122 GO:0043046 GO:0003682 GO:0044027 GO:0090116 GO:0005720 GO:0005654 GO:0042802 GO:0016363 GO:0000775 GO:0071230 GO:0043045 GO:0006346 GO:0003886

NM_001314312.1 GO:0031012 GO:0070062 GO:0006911 GO:0005615 GO:0006910 GO:0001525 GO:0009897 GO:0019897 GO:0007155 GO:0005178 GO:0008429 GO:2000427 GO:0007338 GO:0001786

XM_018048246.1 GO:0030520 GO:0045892 GO:0010724 GO:0003729 GO:0005654 GO:0003714 GO:0000381 GO:0005737 GO:0048813 GO:0021942 GO:0000166 GO:0050885 GO:0008134

XM_018038565.1 GO:0006357 GO:0005634 GO:0003682 GO:0006334 GO:0070577 GO:0005737 GO:0016568

XM_005682989.3 GO:0005634 GO:0035335 GO:0004725 GO:0005737 GO:0007067 GO:0019901 GO:0050699 GO:0051301 GO:0000086

XM_013967589.2 GO:0005634 GO:0006355 GO:0003682 GO:0043565 GO:0008270 GO:0003700

XM_018042290.1 GO:0097368 GO:0034773 GO:0036124 GO:0003677 GO:0006349 GO:0045944 GO:0007283

XM_013971476.2 GO:0005615 GO:0005791 GO:0035269 GO:0005794 GO:0016485 GO:0042383 GO:0016021 GO:0016010

XM_005709644.3 GO:0019904 GO:0005762 GO:0000002 GO:0005743 GO:0070124 GO:0003735 GO:0070125

XM_018048431.1 GO:0005634 GO:0032797 GO:0034719 GO:0044822 GO:0010719 GO:0005102 GO:0010633 GO:0000122 GO:0005829 GO:0030512 GO:0050680 GO:0060394 GO:0000387 GO:0030277

XM_018059339.1 GO:0043154 GO:0031434 GO:0032715 GO:0035774 GO:0031625 GO:0002092 GO:0016023 GO:0032092 GO:0031143 GO:0032088 GO:0005654 GO:0035025 GO:0045944 GO:0032717 GO:0033138 GO:0090240 GO:0031397 GO:0043027 GO:0043547 GO:0007602 GO:0005829 GO:0005159 GO:0005886 GO:0000785 GO:0044212 GO:0043161 GO:0005905 GO:0031701 GO:0070374 GO:0002031 GO:0008134 GO:0005096 GO:0043149

XM_018065117.1 GO:0038161 GO:0019903 GO:0097531 GO:0001077 GO:0035259 GO:0005737 GO:0046983 GO:0032355 GO:0019218 GO:0048541 GO:0071363 GO:0032825 GO:0000255 GO:0004871 GO:0045647 GO:0006573 GO:0042104 GO:0007565 GO:0042448 GO:0046544 GO:0043066 GO:0045579 GO:0045086 GO:0043029 GO:0045944 GO:0006103 GO:0070670 GO:0007595 GO:0006105 GO:0046449 GO:0032819 GO:0050729 GO:0033077 GO:0070669 GO:0006107 GO:0000979 GO:0006631 GO:0006600 GO:0045954 GO:0060397 GO:0006101 GO:0005634 GO:0019530 GO:0030856 GO:0045931 GO:0006549 GO:0019915 GO:0045588 GO:0070672 GO:0001553 GO:0040018 GO:0046543

XM_005700226.2 GO:0007528 GO:0005605 GO:0005201 GO:0005587 GO:0031594

XM_005684900.3 GO:0005634 GO:0000922 GO:0005524 GO:0005874 GO:0046982 GO:0005813 GO:0008017 GO:0005737 GO:0007067 GO:0030496 GO:0051301 GO:0005509 GO:0008568 GO:0051013 GO:0031122

XM_018065829.1 GO:0046982 GO:0001077 GO:0003705 GO:0005737 GO:0019901 GO:0000122 GO:0071277 GO:0003682 GO:0005667 GO:0033613 GO:0061337 GO:0005654 GO:0000978 GO:0042826 GO:0001105 GO:0070375 GO:0048311 GO:0055005 GO:0000790 GO:0001085 GO:0006915 GO:0048813 GO:0045944 GO:0000002 GO:0046332 GO:0035035

XM_005698386.3 GO:0000287 GO:0030425 GO:0005524 GO:0030054 GO:0006661 GO:0044231 GO:0035838 GO:0046854 GO:0002561 GO:0005887 GO:0030672 GO:0042734 GO:0005739 GO:0005765 GO:0031083 GO:0004430 GO:0043204 GO:0031901 GO:0045121 GO:0005794 GO:0035651

XM_018061256.1 GO:0000781 GO:0047485 GO:0005741 GO:0004722 GO:0019901 GO:0030496 GO:0005977 GO:0005730 GO:0072357 GO:0006470 GO:0070688 GO:0032922 GO:0000777 GO:0043153 GO:0005925 GO:0044822 GO:0016607 GO:0046822 GO:0046872 GO:0032154 GO:0051301 GO:0007049

XM_018054430.1 GO:0003222 GO:0090051 GO:0072554 GO:0003209 GO:0003344 GO:0045747 GO:0035924 GO:0000122 GO:0016021 GO:0061074 GO:0061314 GO:0060579 GO:1903588 GO:0035912 GO:0010628 GO:0044344 GO:2000179 GO:0001974 GO:0005509 GO:0045746 GO:0005112 GO:0001569 GO:0030217 GO:0050767

XM_018047073.1 GO:0031965 GO:0097038 GO:0006869 GO:0015485 GO:0005829 GO:0005886

XM_018056339.1 GO:0005622 GO:0035023 GO:0043547 GO:0005089

XM_013974710.2 GO:0005634 GO:0032695 GO:2001056 GO:1900226 GO:0032691 GO:1900016 GO:0010508 GO:0005737 GO:0003779 GO:0071641 GO:0005875 GO:0034341 GO:0008270

XM_018058569.1 GO:0070062 GO:0005929

XM_018041425.1 GO:0048008 GO:0014065 GO:0032587 GO:0007283 GO:0005737 GO:0009791 GO:0030165 GO:0043325 GO:0060021 GO:0051898 GO:0048705 GO:0070301 GO:0045184 GO:0070062 GO:0050853 GO:0005654 GO:0031529 GO:0008209 GO:0035264 GO:0033327 GO:0001553 GO:0008210 GO:0060325

XM_018046850.1 GO:0004252 GO:0046872 GO:0005615 GO:0006508 GO:0005515 GO:0007586 GO:0016021

XM_013964745.2 GO:0033137 GO:0010977 GO:0034067 GO:0043407 GO:0004872 GO:0005794 GO:0016021

XM_005684376.3 GO:0035368 GO:0005654 GO:2000623 GO:0001514 GO:0003730 GO:0048666 GO:0030529 GO:0005739 GO:0021756 GO:0043021

XM_013967112.2 GO:0005622 GO:0035556

XM_018054460.1 GO:0023014 GO:0000278 GO:0005524 GO:0007612 GO:0005737 GO:0007613 GO:0007626 GO:0004702

XM_005679147.3 GO:0045606 GO:0005634 GO:0005615 GO:0043433 GO:0002092 GO:0017147 GO:0010628 GO:0005737 GO:0055062 GO:2000119 GO:0090263 GO:0090090 GO:0009986 GO:1902174 GO:0008285

XM_018051836.1 GO:0050853 GO:0032024 GO:0030154 GO:0038083 GO:0005524 GO:0004715 GO:0071801 GO:0042127 GO:0043066 GO:0005102 GO:0007169 GO:0031234 GO:0007010 GO:0050764 GO:0045087

XM_018046316.1 GO:0043654 GO:0016021 GO:0001891

XM_018066504.1 GO:0021773 GO:0010837 GO:0033077 GO:0001077 GO:0003334 GO:0021902 GO:0019216 GO:0008285 GO:0046632 GO:0005634 GO:0000978 GO:0007165 GO:0003382 GO:0048538 GO:0043066 GO:0045664 GO:0043368 GO:0042475 GO:0043005 GO:0046872 GO:0071678 GO:0045944 GO:0031077 GO:0033153

XM_018061972.1 GO:2000312 GO:0035255 GO:0016021 GO:0014069

NM_001285732.1 GO:0006457 GO:0005788 GO:0070062 GO:0005634 GO:0044822 GO:0005925 GO:0034976 GO:0043209 GO:0042470 GO:0045454 GO:0009986 GO:0003756 GO:2001238

XM_005690992.3 GO:0007186 GO:0015271 GO:0030322 GO:0071805 GO:0022841 GO:0008076

XM_005679898.3 GO:0030424 GO:0004709 GO:0005524 GO:2000672 GO:0042803 GO:0007254 GO:0046777 GO:0019901 GO:0018107 GO:0030426 GO:0018105 GO:0005829 GO:0016572 GO:0005886 GO:0000186

XM_018038870.1 GO:0001952 GO:0005615 GO:0038083 GO:0005524 GO:0014909 GO:0060444 GO:0007566 GO:0043235 GO:0005887 GO:0044319 GO:0008285 GO:0070062 GO:0001558 GO:0043583 GO:0010715 GO:0038062 GO:0038063 GO:0060749 GO:0005518 GO:0061302

XR_001295466.2 GO:0003677 GO:0005730 GO:0006355 GO:0016021 GO:0005737

XM_013966818.2 GO:0005615 GO:0045202

XM_018057178.1 GO:0002011 GO:0007165

XM_013965964.2 GO:0005634 GO:0044822 GO:0016075 GO:0045111 GO:0071028 GO:0004527 GO:0071044 GO:0005794 GO:0090305 GO:0005886

XM_005674707.2 GO:0005198 GO:0045095

XM_018046088.1 GO:0048026 GO:0030317 GO:0003723 GO:0005634 GO:0000381 GO:0000166 GO:0007283 GO:0005737

XM_018061605.1 GO:0006886 GO:0032588 GO:0019904 GO:0070273 GO:0005829 GO:0050708

XM_018047856.1 GO:0007193 GO:0005622 GO:0006171 GO:0007212 GO:0019933 GO:0071870 GO:0004016 GO:0000166 GO:0071380 GO:0016021 GO:0007189 GO:0042312 GO:0005886

XM_018062196.1 GO:0044822 GO:0006355 GO:0030490 GO:0034455

XM_005683422.3 GO:0014902 GO:0045666 GO:0055012 GO:0001568 GO:0005737 GO:0003211 GO:0071560 GO:2000310 GO:2000987 GO:0000978 GO:0051966 GO:0042826 GO:0048703 GO:0016607 GO:0003151 GO:0002062 GO:0002634 GO:0001764 GO:0045944 GO:2000727 GO:0045663 GO:0014898 GO:0071498 GO:0060998 GO:0071374 GO:0051963 GO:0000122 GO:0072102 GO:0090073 GO:0030220 GO:0003139 GO:0046928 GO:0030318 GO:0060079 GO:0071864 GO:0050853 GO:0003138 GO:0001958 GO:2001013 GO:0000165 GO:0060045 GO:0003680 GO:0045669 GO:0045652 GO:0043234 GO:0050680 GO:0009615 GO:0035690 GO:0072160 GO:0071222 GO:0001077 GO:0060297 GO:0030890 GO:0007521 GO:0006959 GO:0035984 GO:0003682 GO:0033613 GO:0071837 GO:0001046 GO:0043524 GO:0048667 GO:2000311 GO:0000983 GO:0001974 GO:0035198 GO:0060025 GO:0001782 GO:0000980 GO:0048167 GO:0001205 GO:0046982 GO:0071277 GO:0060536 GO:0060021 GO:0003185 GO:0007611 GO:2000111 GO:0014033 GO:0051145 GO:0001947 GO:0030501 GO:0030279 GO:0010694 GO:2001016 GO:0030224

XM_005688176.3 GO:0003677 GO:0005634 GO:0005524 GO:0006260 GO:0048232 GO:0000724 GO:0097362 GO:0007292

XM_005680676.3 GO:0045859 GO:0005635 GO:0060263 GO:0007015 GO:0042129 GO:0007264 GO:0019887 GO:0005829 GO:0043304 GO:0060753 GO:0016021 GO:0007186 GO:0070062 GO:0090023 GO:0005925 GO:0030027 GO:0003676 GO:0003924 GO:0005884 GO:0010592 GO:0010810 GO:0090305 GO:0005525 GO:0004519 GO:0008284

XM_018056191.1 GO:0004864 GO:0005654 GO:0019902 GO:0010923

XM_005682114.3 GO:0046872 GO:0005615 GO:0007165 GO:0004114 GO:0072372

XM_005691695.2 GO:0016021

XM_005687509.3 GO:0030514 GO:0009838 GO:0048698 GO:0031625 GO:0034389 GO:0005741 GO:0060612 GO:0030496 GO:0051301 GO:0005811 GO:0050905 GO:0051881 GO:0005886 GO:0045202

XM_018066318.1 GO:0005783 GO:0005615 GO:0010951 GO:0048406 GO:0043043 GO:0006465 GO:0019058 GO:0016486 GO:0008283 GO:0045714 GO:0032911 GO:0032902 GO:0016021 GO:0005886 GO:0070062 GO:0004252 GO:0002020 GO:0030140 GO:0004867 GO:0042277 GO:0032940 GO:0005802 GO:0032804 GO:0009986 GO:0046872 GO:0045121 GO:0009966

XM_005695792.3 GO:2000124 GO:0009395 GO:0070062 GO:0030336 GO:0032281 GO:0004620 GO:0060292 GO:0047372 GO:0005739 GO:0046464 GO:0046889

XM_005690576.3 GO:0070062 GO:0052689 GO:0008474 GO:0002084 GO:0005737

XM_005686991.3 GO:0005856 GO:0031434 GO:0005634 GO:0005524 GO:0031625 GO:0045599 GO:0045081 GO:0043405 GO:0032436 GO:0005737 GO:0006469 GO:0004860 GO:0004672 GO:0055106 GO:0008134

XM_005683037.3 GO:0001540 GO:0005634 GO:0006355 GO:0015629 GO:0008134 GO:0005737

XM_018055876.1 GO:0015909 GO:0043588 GO:0001579 GO:0031526 GO:0004467 GO:0001676 GO:0005902 GO:0042760 GO:0016021 GO:0005789 GO:0031957 GO:0044539

XM_013962990.2 GO:0060425 GO:0010468 GO:0005654 GO:0005524 GO:0043066 GO:0043408 GO:0005737 GO:0035264 GO:0003016 GO:0005977 GO:0004672 GO:0048286

XM_018057818.1 GO:0070062 GO:0005737

XM_013974950.2 GO:0016021

XM_018044753.1 GO:0000287 GO:0005765 GO:0005783 GO:0005524 GO:0045332 GO:0016021 GO:0004012 GO:0055037 GO:0005886

XM_005688851.3 GO:0050660 GO:0016021 GO:0004506 GO:0055114

XM_018039076.1 GO:0008542 GO:0043113 GO:0046580 GO:0016358 GO:0043524 GO:0043547 GO:0043408 GO:0005737 GO:0007389 GO:0043198 GO:0050771 GO:0048169 GO:0031235 GO:0005096

XM_018056430.1 GO:0046872 GO:0044822 GO:0000381

XM_005679477.2 GO:0005615 GO:0006508 GO:0008270 GO:0004181

XM_005676747.3 GO:0006810 GO:0005765 GO:0005887

XM_018050583.1 GO:0031410

XM_005686514.3 GO:0070062 GO:0005654 GO:0044822 GO:0000166 GO:0008270 GO:0048025

XM_018060522.1 GO:0070062 GO:0005840 GO:0005615 GO:0042802 GO:0048266 GO:0030198 GO:0005614 GO:0006412 GO:0003735 GO:0005604

XM_018057651.1 GO:0017075 GO:0005881 GO:0005739

XM_018046164.1 GO:0061484 GO:0044822 GO:0031519 GO:0005671 GO:0007339

XM_018040804.1 GO:0030515 GO:0034388 GO:0000462 GO:0044822 GO:0032040

XM_005689020.3 GO:0046872 GO:0005634 GO:0005739 GO:0030001

XM_005696338.3 GO:0003677 GO:0006360 GO:0001054 GO:0005666 GO:0046983 GO:0001056 GO:0005736 GO:0006383

XM_018062326.1 GO:0071711 GO:0010951 GO:0004867 GO:0008233 GO:0007163 GO:0016021 GO:0001843 GO:0060672

XM_018040467.1 GO:0060041 GO:0008589 GO:1902017 GO:0021532 GO:0061512 GO:0035721 GO:0036064 GO:0072001 GO:0048705 GO:0035845 GO:0007368 GO:0032391 GO:0007507 GO:0005813 GO:0001750 GO:0035108 GO:0005930 GO:0030991

XM_018067241.1 GO:0003779 GO:0030036 GO:0044822 GO:0007507 GO:0030054

XM_018057857.1 GO:0030018 GO:0005829 GO:0042383 GO:0031594 GO:0005198 GO:0005882 GO:0045103

XM_013973729.2 GO:0010494 GO:0008344 GO:0007283 GO:0003730 GO:0000932 GO:0048863 GO:1900246 GO:2000637 GO:0051726 GO:0061157

XM_005694468.3 GO:0005634 GO:0005794 GO:0016021 GO:0005385 GO:0005886 GO:0071577

XM_018040066.1 GO:0070062 GO:0005524 GO:0006165 GO:0006241 GO:0004550 GO:0006228 GO:0005739 GO:0042981 GO:0006183

XM_018064644.1 GO:0005944 GO:0046935 GO:0043551

XM_018057143.1 GO:0005730

XM_018059124.1 GO:0070062 GO:0005654 GO:0045806 GO:0019855 GO:0008092 GO:0051044 GO:0051926 GO:0005737 GO:0008289 GO:0005886 GO:0097320

NM_001314206.1 GO:0072562 GO:0005524 GO:0031072 GO:0031625 GO:0007339 GO:0044297 GO:0005829 GO:0008180 GO:0051082 GO:0042026 GO:0002199

XM_005675300.3 GO:1900244 GO:0010841 GO:0016080 GO:0097115 GO:0042043 GO:0009897 GO:0060076 GO:0051260 GO:0051965 GO:0002087 GO:0097110 GO:2000310 GO:0048789 GO:0005887 GO:2000809 GO:0030165 GO:0007158 GO:0045184 GO:2000302 GO:0097120 GO:0097104 GO:0016339 GO:0097113 GO:0007157 GO:2000463 GO:0060999 GO:0061002 GO:0097119 GO:0072553

XM_018047544.1 GO:0071837 GO:0000978 GO:0005654 GO:0042826 GO:0042803 GO:0000790 GO:0043923 GO:0000982 GO:0046872 GO:0008022 GO:0045944 GO:0008134

XM_018054868.1 GO:0016021

XM_013969782.2 GO:0005576 GO:0005179

XM_018064703.1 GO:0016020 GO:0003677 GO:0072368 GO:0003714 GO:0051225 GO:0042826 GO:0016580 GO:0035257 GO:0072362 GO:0017053 GO:0046329 GO:0001102 GO:0005876

XM_018061830.1 GO:0007612 GO:0030314 GO:0015278 GO:0007613 GO:0035640 GO:0048168 GO:0040011 GO:0016021 GO:0060314 GO:0050885 GO:0005789 GO:0005886

XM_018058392.1 GO:0070062

XM_018057247.1 GO:0007165

XR_001918899.1 GO:0032502 GO:0004622 GO:0005783 GO:0016021 GO:0006629

XM_018055048.1 GO:0005815 GO:0045055 GO:0005741 GO:0071468 GO:0070164 GO:0035773 GO:2000008 GO:0030141 GO:0043015 GO:0005794 GO:0055037 GO:0005769 GO:0017137

XM_013971163.2 GO:0012505 GO:0019898 GO:0005543

XM_018063453.1 GO:0003677 GO:0005634 GO:0008270

XM_005693449.3 GO:2001235 GO:0004861 GO:0045736 GO:0005737 GO:0008285 GO:0030332

XM_005685503.3 GO:0005783 GO:0071468 GO:0061077 GO:0004860 GO:0010923 GO:0022406 GO:0048306 GO:0032088 GO:0051259 GO:0042308 GO:0070062 GO:0006813 GO:0005925 GO:0015630 GO:0010824 GO:0008017 GO:0043531 GO:0070885 GO:0032417 GO:0090314 GO:0060050 GO:1901214 GO:0005215 GO:0031397 GO:0005524 GO:0030214 GO:0017156 GO:0000139 GO:0061025 GO:0051879 GO:0007264 GO:0005829 GO:0019900 GO:0008270 GO:0006611 GO:0005886 GO:0031122 GO:0005634 GO:0015459 GO:0051453 GO:0005793 GO:0031953 GO:2000299 GO:1900034 GO:0030133 GO:0005509 GO:0050821 GO:0001578

XM_018061857.1 GO:0030174 GO:0003677 GO:0005634 GO:0033262 GO:0000076

XM_018039595.1 GO:0007173 GO:0070374 GO:0071364 GO:0070064 GO:0008284

XM_005684309.3 GO:0016020 GO:0005739 GO:0005777 GO:0055114 GO:0016491
[truncated: 1,014,327 more chars]
