# Supplementary material for: MicroRNA expression profiling of goat peripheral blood mononuclear cells in response to peste des petits ruminants virus infection
Source: Vet Res. 2018 Jul 16;49:62. doi: 10.1186/s13567-018-0565-3 (PMC6048839; doi:10.1186/s13567-018-0565-3)
Supplement: Supplementary file 6 — Additional file 6. KEGG analysis of target genes annotated for miRNA differentially expressed in mock- and PPRV-infected goat PBMC. KEGG pathway annotation revealed that 10 364 background genes were annotated for 317 biological processes. [file 13567_2018_565_MOESM6_ESM.doc]

|  | | | | | | | | |
| --- | --- | --- | --- | --- | --- | --- | --- | --- |
| **#** | **Pathway** | **Cont-vs-PPRV_DEGseq (16510)** | **All-Unigene (28860)** | **Pvalue** | **Qvalue** | **Pathway ID** | **Level 1** | **Level 2** |
| 1 | [Dorso-ventral axis formation](#gene1) | 1821 | 2936 | 1.261418e-08 | 3.998695e-06 | ko04320 | Organismal Systems | Development |
| 2 | [cAMP signaling pathway](#gene2) | 282 | 411 | 1.135602e-06 | 1.799929e-04 | ko04024 | Environmental Information Processing | Signal transduction |
| 3 | [Endocrine resistance](#gene3) | 186 | 262 | 2.612768e-06 | 2.645986e-04 | ko01522 | Human Diseases | Drug resistance: Antineoplastic |
| 4 | [Inflammatory mediator regulation of TRP channels](#gene4) | 90 | 116 | 3.338783e-06 | 2.645986e-04 | ko04750 | Organismal Systems | Sensory system |
| 5 | [ABC transporters](#gene5) | 148 | 206 | 9.359206e-06 | 5.933737e-04 | ko02010 | Environmental Information Processing | Membrane transport |
| 6 | [ErbB signaling pathway](#gene6) | 163 | 234 | 5.62012e-05 | 2.969297e-03 | ko04012 | Environmental Information Processing | Signal transduction |
| 7 | [Epstein-Barr virus infection](#gene7) | 566 | 895 | 0.0001098469 | 4.164134e-03 | ko05169 | Human Diseases | Infectious diseases: Viral |
| 8 | [Adrenergic signaling in cardiomyocytes](#gene8) | 175 | 255 | 0.0001111764 | 4.164134e-03 | ko04261 | Organismal Systems | Circulatory system |
| 9 | [Non-small cell lung cancer](#gene9) | 110 | 153 | 0.0001191075 | 4.164134e-03 | ko05223 | Human Diseases | Cancers: Specific types |
| 10 | [cGMP-PKG signaling pathway](#gene10) | 250 | 376 | 0.0001313607 | 4.164134e-03 | ko04022 | Environmental Information Processing | Signal transduction |
| 11 | [Apelin signaling pathway](#gene11) | 314 | 484 | 0.0003109529 | 8.961097e-03 | ko04371 | Environmental Information Processing | Signal transduction |
| 12 | [Renal cell carcinoma](#gene12) | 150 | 219 | 0.0003798726 | 1.003497e-02 | ko05211 | Human Diseases | Cancers: Specific types |
| 13 | [Focal adhesion](#gene13) | 475 | 752 | 0.0004352887 | 1.061435e-02 | ko04510 | Cellular Processes | Cellular community - eukaryotes |
| 14 | [Dopaminergic synapse](#gene14) | 156 | 230 | 0.0005922992 | 1.341135e-02 | ko04728 | Organismal Systems | Nervous system |
| 15 | [Bile secretion](#gene15) | 116 | 167 | 0.0007355897 | 1.514605e-02 | ko04976 | Organismal Systems | Digestive system |
| 16 | [Chronic myeloid leukemia](#gene16) | 107 | 153 | 0.0007905298 | 1.514605e-02 | ko05220 | Human Diseases | Cancers: Specific types |
| 17 | [Insulin signaling pathway](#gene17) | 360 | 565 | 0.0008533971 | 1.514605e-02 | ko04910 | Organismal Systems | Endocrine system |
| 18 | [Hepatitis C](#gene18) | 137 | 201 | 0.0009078075 | 1.514605e-02 | ko05160 | Human Diseases | Infectious diseases: Viral |
| 19 | [Pancreatic cancer](#gene19) | 137 | 201 | 0.0009078075 | 1.514605e-02 | ko05212 | Human Diseases | Cancers: Specific types |
| 20 | [Proteoglycans in cancer](#gene20) | 271 | 419 | 0.001005158 | 1.575652e-02 | ko05205 | Human Diseases | Cancers: Overview |
| 21 | [Olfactory transduction](#gene21) | 116 | 168 | 0.001043807 | 1.575652e-02 | ko04740 | Organismal Systems | Sensory system |
| 22 | [T cell receptor signaling pathway](#gene22) | 139 | 205 | 0.00116286 | 1.675576e-02 | ko04660 | Organismal Systems | Immune system |
| 23 | [Aldosterone synthesis and secretion](#gene23) | 111 | 161 | 0.001436532 | 1.946279e-02 | ko04925 | Organismal Systems | Endocrine system |
| 24 | [Fc epsilon RI signaling pathway](#gene24) | 121 | 177 | 0.001479385 | 1.946279e-02 | ko04664 | Organismal Systems | Immune system |
| 25 | [Insulin secretion](#gene25) | 21 | 24 | 0.00153492 | 1.946279e-02 | ko04911 | Organismal Systems | Endocrine system |
| 26 | [Acute myeloid leukemia](#gene26) | 137 | 203 | 0.001668873 | 2.034741e-02 | ko05221 | Human Diseases | Cancers: Specific types |
| 27 | [Toxoplasmosis](#gene27) | 498 | 800 | 0.001855716 | 2.101526e-02 | ko05145 | Human Diseases | Infectious diseases: Parasitic |
| 28 | [Vascular smooth muscle contraction](#gene28) | 151 | 226 | 0.001898208 | 2.101526e-02 | ko04270 | Organismal Systems | Circulatory system |
| 29 | [Phospholipase D signaling pathway](#gene29) | 221 | 340 | 0.001922532 | 2.101526e-02 | ko04072 | Environmental Information Processing | Signal transduction |
| 30 | [Oxytocin signaling pathway](#gene30) | 285 | 447 | 0.002619686 | 2.768135e-02 | ko04921 | Organismal Systems | Endocrine system |
| 31 | [Toll-like receptor signaling pathway](#gene31) | 155 | 234 | 0.002845701 | 2.825836e-02 | ko04620 | Organismal Systems | Immune system |
| 32 | [RNA degradation](#gene32) | 229 | 355 | 0.002852579 | 2.825836e-02 | ko03018 | Genetic Information Processing | Folding, sorting and degradation |
| 33 | [Glioma](#gene33) | 157 | 238 | 0.003442348 | 3.306740e-02 | ko05214 | Human Diseases | Cancers: Specific types |
| 34 | [Chagas disease (American trypanosomiasis)](#gene34) | 129 | 193 | 0.003802924 | 3.545667e-02 | ko05142 | Human Diseases | Infectious diseases: Parasitic |
| 35 | [Breast cancer](#gene35) | 178 | 273 | 0.004107004 | 3.585795e-02 | ko05224 | Human Diseases | Cancers: Specific types |
| 36 | [Vibrio cholerae infection](#gene36) | 170 | 260 | 0.004183186 | 3.585795e-02 | ko05110 | Human Diseases | Infectious diseases: Bacterial |
| 37 | [Platelet activation](#gene37) | 290 | 458 | 0.004230591 | 3.585795e-02 | ko04611 | Organismal Systems | Immune system |
| 38 | [Nicotine addiction](#gene38) | 16 | 18 | 0.004300241 | 3.585795e-02 | ko05033 | Human Diseases | Substance dependence |
| 39 | [HIF-1 signaling pathway](#gene39) | 179 | 275 | 0.004456905 | 3.585795e-02 | ko04066 | Environmental Information Processing | Signal transduction |
| 40 | [Insulin resistance](#gene40) | 253 | 397 | 0.004524662 | 3.585795e-02 | ko04931 | Human Diseases | Endocrine and metabolic diseases |
| 41 | [AMPK signaling pathway](#gene41) | 350 | 560 | 0.005768964 | 4.409006e-02 | ko04152 | Environmental Information Processing | Signal transduction |
| 42 | [Phagosome](#gene42) | 266 | 420 | 0.005841585 | 4.409006e-02 | ko04145 | Cellular Processes | Transport and catabolism |
| 43 | [Fatty acid biosynthesis](#gene43) | 29 | 37 | 0.005986801 | 4.413525e-02 | ko00061 | Metabolism | Lipid metabolism |
| 44 | [Carbohydrate digestion and absorption](#gene44) | 73 | 105 | 0.006352276 | 4.576526e-02 | ko04973 | Organismal Systems | Digestive system |
| 45 | [ECM-receptor interaction](#gene45) | 266 | 421 | 0.006928716 | 4.880895e-02 | ko04512 | Environmental Information Processing | Signaling molecules and interaction |
| 46 | [Calcium signaling pathway](#gene46) | 200 | 312 | 0.007464544 | 5.144044e-02 | ko04020 | Environmental Information Processing | Signal transduction |
| 47 | [Gastric acid secretion](#gene47) | 82 | 120 | 0.008076813 | 5.390160e-02 | ko04971 | Organismal Systems | Digestive system |
| 48 | [MAPK signaling pathway](#gene48) | 290 | 462 | 0.008161757 | 5.390160e-02 | ko04010 | Environmental Information Processing | Signal transduction |
| 49 | [Neurotrophin signaling pathway](#gene49) | 231 | 364 | 0.008468386 | 5.478527e-02 | ko04722 | Organismal Systems | Nervous system |
| 50 | [Osteoclast differentiation](#gene50) | 151 | 233 | 0.01058888 | 6.713350e-02 | ko04380 | Organismal Systems | Development |
| 51 | [Morphine addiction](#gene51) | 63 | 91 | 0.01238838 | 7.700228e-02 | ko05032 | Human Diseases | Substance dependence |
| 52 | [Thyroid hormone signaling pathway](#gene52) | 197 | 310 | 0.01306936 | 7.967283e-02 | ko04919 | Organismal Systems | Endocrine system |
| 53 | [MAPK signaling pathway - fly](#gene53) | 163 | 254 | 0.01374302 | 8.034771e-02 | ko04013 | Environmental Information Processing | Signal transduction |
| 54 | [Ras signaling pathway](#gene54) | 289 | 464 | 0.01422616 | 8.034771e-02 | ko04014 | Environmental Information Processing | Signal transduction |
| 55 | [Long-term potentiation](#gene55) | 138 | 213 | 0.01422674 | 8.034771e-02 | ko04720 | Organismal Systems | Nervous system |
| 56 | [Retrograde endocannabinoid signaling](#gene56) | 64 | 93 | 0.01432277 | 8.034771e-02 | ko04723 | Organismal Systems | Nervous system |
| 57 | [Colorectal cancer](#gene57) | 141 | 218 | 0.01444738 | 8.034771e-02 | ko05210 | Human Diseases | Cancers: Specific types |
| 58 | [VEGF signaling pathway](#gene58) | 185 | 291 | 0.01541519 | 8.425199e-02 | ko04370 | Environmental Information Processing | Signal transduction |
| 59 | [Mismatch repair](#gene59) | 41 | 57 | 0.01577688 | 8.476730e-02 | ko03430 | Genetic Information Processing | Replication and repair |
| 60 | [Protein export](#gene60) | 129 | 199 | 0.01689956 | 8.928601e-02 | ko03060 | Genetic Information Processing | Folding, sorting and degradation |
| 61 | [Natural killer cell mediated cytotoxicity](#gene61) | 138 | 214 | 0.01765026 | 9.172348e-02 | ko04650 | Organismal Systems | Immune system |
| 62 | [Longevity regulating pathway - multiple species](#gene62) | 188 | 297 | 0.01851562 | 9.354640e-02 | ko04213 | Organismal Systems | Aging |
| 63 | [Adipocytokine signaling pathway](#gene63) | 153 | 239 | 0.01859124 | 9.354640e-02 | ko04920 | Organismal Systems | Endocrine system |
| 64 | [Purine metabolism](#gene64) | 417 | 682 | 0.01921302 | 9.516449e-02 | ko00230 | Metabolism | Nucleotide metabolism |
| 65 | [Bladder cancer](#gene65) | 64 | 94 | 0.02004752 | 9.777021e-02 | ko05219 | Human Diseases | Cancers: Specific types |
| 66 | [Longevity regulating pathway](#gene66) | 242 | 388 | 0.02133086 | 1.024528e-01 | ko04211 | Organismal Systems | Aging |
| 67 | [Epithelial cell signaling in Helicobacter pylori infection](#gene67) | 71 | 106 | 0.02517157 | 1.163011e-01 | ko05120 | Human Diseases | Infectious diseases: Bacterial |
| 68 | [PI3K-Akt signaling pathway](#gene68) | 602 | 999 | 0.02517345 | 1.163011e-01 | ko04151 | Environmental Information Processing | Signal transduction |
| 69 | [Aldosterone-regulated sodium reabsorption](#gene69) | 46 | 66 | 0.02543218 | 1.163011e-01 | ko04960 | Organismal Systems | Excretory system |
| 70 | [Regulation of lipolysis in adipocytes](#gene70) | 66 | 98 | 0.02568164 | 1.163011e-01 | ko04923 | Organismal Systems | Endocrine system |
| 71 | [B cell receptor signaling pathway](#gene71) | 144 | 226 | 0.02687485 | 1.199905e-01 | ko04662 | Organismal Systems | Immune system |
| 72 | [RIG-I-like receptor signaling pathway](#gene72) | 24 | 32 | 0.02922472 | 1.286699e-01 | ko04622 | Organismal Systems | Immune system |
| 73 | [Jak-STAT signaling pathway](#gene73) | 53 | 78 | 0.03416449 | 1.442001e-01 | ko04630 | Environmental Information Processing | Signal transduction |
| 74 | [Melanoma](#gene74) | 110 | 171 | 0.03433529 | 1.442001e-01 | ko05218 | Human Diseases | Cancers: Specific types |
| 75 | [Amino sugar and nucleotide sugar metabolism](#gene75) | 181 | 289 | 0.03433892 | 1.442001e-01 | ko00520 | Metabolism | Carbohydrate metabolism |
| 76 | [HTLV-I infection](#gene76) | 215 | 346 | 0.03457163 | 1.442001e-01 | ko05166 | Human Diseases | Infectious diseases: Viral |
| 77 | [Progesterone-mediated oocyte maturation](#gene77) | 170 | 271 | 0.03653212 | 1.503985e-01 | ko04914 | Organismal Systems | Endocrine system |
| 78 | [Hippo signaling pathway -multiple species](#gene78) | 39 | 56 | 0.03860517 | 1.540931e-01 | ko04392 | Environmental Information Processing | Signal transduction |
| 79 | [Axon guidance](#gene79) | 159 | 253 | 0.03882338 | 1.540931e-01 | ko04360 | Organismal Systems | Development |
| 80 | [Hippo signaling pathway](#gene80) | 89 | 137 | 0.03888784 | 1.540931e-01 | ko04390 | Environmental Information Processing | Signal transduction |
| 81 | [Rap1 signaling pathway](#gene81) | 172 | 275 | 0.04062061 | 1.589720e-01 | ko04015 | Environmental Information Processing | Signal transduction |
| 82 | [Ubiquitin mediated proteolysis](#gene82) | 283 | 462 | 0.04177041 | 1.605072e-01 | ko04120 | Genetic Information Processing | Folding, sorting and degradation |
| 83 | [Peroxisome](#gene83) | 116 | 182 | 0.04278075 | 1.605072e-01 | ko04146 | Cellular Processes | Transport and catabolism |
| 84 | [Fluid shear stress and atherosclerosis](#gene84) | 242 | 393 | 0.04297133 | 1.605072e-01 | ko05418 | Human Diseases | Cardiovascular diseases |
| 85 | [Glucagon signaling pathway](#gene85) | 223 | 361 | 0.0430382 | 1.605072e-01 | ko04922 | Organismal Systems | Endocrine system |
| 86 | [Estrogen signaling pathway](#gene86) | 144 | 229 | 0.0462358 | 1.704273e-01 | ko04915 | Organismal Systems | Endocrine system |
| 87 | [Choline metabolism in cancer](#gene87) | 501 | 834 | 0.04801604 | 1.749550e-01 | ko05231 | Human Diseases | Cancers: Overview |
| 88 | [Pathogenic Escherichia coli infection](#gene88) | 341 | 562 | 0.0505822 | 1.792254e-01 | ko05130 | Human Diseases | Infectious diseases: Bacterial |
| 89 | [Fc gamma R-mediated phagocytosis](#gene89) | 469 | 780 | 0.05071527 | 1.792254e-01 | ko04666 | Organismal Systems | Immune system |
| 90 | [Terpenoid backbone biosynthesis](#gene90) | 36 | 52 | 0.05162028 | 1.792254e-01 | ko00900 | Metabolism | Metabolism of terpenoids and polyketides |
| 91 | [Prostate cancer](#gene91) | 146 | 233 | 0.05164821 | 1.792254e-01 | ko05215 | Human Diseases | Cancers: Specific types |
| 92 | [Renin secretion](#gene92) | 134 | 213 | 0.05201494 | 1.792254e-01 | ko04924 | Organismal Systems | Endocrine system |
| 93 | [Phosphatidylinositol signaling system](#gene93) | 219 | 356 | 0.0543166 | 1.851437e-01 | ko04070 | Environmental Information Processing | Signal transduction |
| 94 | [Arginine and proline metabolism](#gene94) | 82 | 127 | 0.05496658 | 1.853660e-01 | ko00330 | Metabolism | Amino acid metabolism |
| 95 | [Long-term depression](#gene95) | 100 | 157 | 0.05781544 | 1.929210e-01 | ko04730 | Organismal Systems | Nervous system |
| 96 | [Small cell lung cancer](#gene96) | 205 | 333 | 0.05891234 | 1.945335e-01 | ko05222 | Human Diseases | Cancers: Specific types |
| 97 | [Rheumatoid arthritis](#gene97) | 37 | 54 | 0.05975065 | 1.952676e-01 | ko05323 | Human Diseases | Immune diseases |
| 98 | [Lysine biosynthesis](#gene98) | 5 | 5 | 0.06125494 | 1.981410e-01 | ko00300 | Metabolism | Amino acid metabolism |
| 99 | [TNF signaling pathway](#gene99) | 110 | 174 | 0.06219373 | 1.991456e-01 | ko04668 | Environmental Information Processing | Signal transduction |
| 100 | [Autophagy - animal](#gene100) | 343 | 568 | 0.06589402 | 2.088840e-01 | ko04140 | Cellular Processes | Transport and catabolism |
| 101 | [Cholinergic synapse](#gene101) | 109 | 173 | 0.07021305 | 2.203717e-01 | ko04725 | Organismal Systems | Nervous system |
| 102 | [Measles](#gene102) | 137 | 220 | 0.07210649 | 2.240957e-01 | ko05162 | Human Diseases | Infectious diseases: Viral |
| 103 | [Apoptosis](#gene103) | 122 | 195 | 0.07368216 | 2.267694e-01 | ko04210 | Cellular Processes | Cell growth and death |
| 104 | [RNA polymerase](#gene104) | 141 | 227 | 0.07537556 | 2.297505e-01 | ko03020 | Genetic Information Processing | Transcription |
| 105 | [Collecting duct acid secretion](#gene105) | 29 | 42 | 0.07984096 | 2.410437e-01 | ko04966 | Organismal Systems | Excretory system |
| 106 | [AGE-RAGE signaling pathway in diabetic complications](#gene106) | 378 | 630 | 0.08170115 | 2.443327e-01 | ko04933 | Human Diseases | Endocrine and metabolic diseases |
| 107 | [GnRH signaling pathway](#gene107) | 106 | 169 | 0.08391134 | 2.485971e-01 | ko04912 | Organismal Systems | Endocrine system |
| 108 | [Type II diabetes mellitus](#gene108) | 46 | 70 | 0.09258983 | 2.684584e-01 | ko04930 | Human Diseases | Endocrine and metabolic diseases |
| 109 | [Sphingolipid signaling pathway](#gene109) | 257 | 425 | 0.09301872 | 2.684584e-01 | ko04071 | Environmental Information Processing | Signal transduction |
| 110 | [Tuberculosis](#gene110) | 247 | 408 | 0.09315592 | 2.684584e-01 | ko05152 | Human Diseases | Infectious diseases: Bacterial |
| 111 | [Endometrial cancer](#gene111) | 115 | 185 | 0.09769829 | 2.790122e-01 | ko05213 | Human Diseases | Cancers: Specific types |
| 112 | [Monobactam biosynthesis](#gene112) | 4 | 4 | 0.1070867 | 3.009364e-01 | ko00261 | Metabolism | Biosynthesis of other secondary metabolites |
| 113 | [Herpes simplex infection](#gene113) | 272 | 452 | 0.107557 | 3.009364e-01 | ko05168 | Human Diseases | Infectious diseases: Viral |
| 114 | [Amphetamine addiction](#gene114) | 86 | 137 | 0.1082232 | 3.009364e-01 | ko05031 | Human Diseases | Substance dependence |
| 115 | [Salivary secretion](#gene115) | 153 | 250 | 0.1114196 | 3.071305e-01 | ko04970 | Organismal Systems | Digestive system |
| 116 | [mTOR signaling pathway](#gene116) | 301 | 502 | 0.1125232 | 3.074987e-01 | ko04150 | Environmental Information Processing | Signal transduction |
| 117 | [Circadian entrainment](#gene117) | 109 | 176 | 0.1158268 | 3.138213e-01 | ko04713 | Organismal Systems | Environmental adaptation |
| 118 | [Pathways in cancer](#gene118) | 391 | 657 | 0.1211686 | 3.255123e-01 | ko05200 | Human Diseases | Cancers: Overview |
| 119 | [Basal cell carcinoma](#gene119) | 22 | 32 | 0.1260458 | 3.333593e-01 | ko05217 | Human Diseases | Cancers: Specific types |
| 120 | [Regulation of actin cytoskeleton](#gene120) | 453 | 764 | 0.1261928 | 3.333593e-01 | ko04810 | Cellular Processes | Cell motility |
| 121 | [Prolactin signaling pathway](#gene121) | 102 | 165 | 0.1307689 | 3.398625e-01 | ko04917 | Organismal Systems | Endocrine system |
| 122 | [Hepatitis B](#gene122) | 144 | 236 | 0.1307988 | 3.398625e-01 | ko05161 | Human Diseases | Infectious diseases: Viral |
| 123 | [Synaptic vesicle cycle](#gene123) | 222 | 369 | 0.1351302 | 3.482624e-01 | ko04721 | Organismal Systems | Nervous system |
| 124 | [Protein digestion and absorption](#gene124) | 441 | 745 | 0.1415067 | 3.617550e-01 | ko04974 | Organismal Systems | Digestive system |
| 125 | [Nicotinate and nicotinamide metabolism](#gene125) | 52 | 82 | 0.1523767 | 3.864273e-01 | ko00760 | Metabolism | Metabolism of cofactors and vitamins |
| 126 | [Caffeine metabolism](#gene126) | 11 | 15 | 0.1583703 | 3.984396e-01 | ko00232 | Metabolism | Biosynthesis of other secondary metabolites |
| 127 | [FoxO signaling pathway](#gene127) | 263 | 441 | 0.1608912 | 3.986007e-01 | ko04068 | Environmental Information Processing | Signal transduction |
| 128 | [NF-kappa B signaling pathway](#gene128) | 53 | 84 | 0.1631395 | 3.986007e-01 | ko04064 | Environmental Information Processing | Signal transduction |
| 129 | [Longevity regulating pathway - worm](#gene129) | 140 | 231 | 0.1631998 | 3.986007e-01 | ko04212 | Organismal Systems | Aging |
| 130 | [Taste transduction](#gene130) | 50 | 79 | 0.163464 | 3.986007e-01 | ko04742 | Organismal Systems | Sensory system |
| 131 | [EGFR tyrosine kinase inhibitor resistance](#gene131) | 158 | 262 | 0.1697444 | 4.038445e-01 | ko01521 | Human Diseases | Drug resistance: Antineoplastic |
| 132 | [Chemokine signaling pathway](#gene132) | 386 | 653 | 0.169836 | 4.038445e-01 | ko04062 | Organismal Systems | Immune system |
| 133 | [Renin-angiotensin system](#gene133) | 9 | 12 | 0.1707103 | 4.038445e-01 | ko04614 | Organismal Systems | Endocrine system |
| 134 | [Proximal tubule bicarbonate reclamation](#gene134) | 9 | 12 | 0.1707103 | 4.038445e-01 | ko04964 | Organismal Systems | Excretory system |
| 135 | [N-Glycan biosynthesis](#gene135) | 54 | 86 | 0.1740671 | 4.087353e-01 | ko00510 | Metabolism | Glycan biosynthesis and metabolism |
| 136 | [Leishmaniasis](#gene136) | 70 | 113 | 0.1776643 | 4.099825e-01 | ko05140 | Human Diseases | Infectious diseases: Parasitic |
| 137 | [TGF-beta signaling pathway](#gene137) | 119 | 196 | 0.1780703 | 4.099825e-01 | ko04350 | Environmental Information Processing | Signal transduction |
| 138 | [Sulfur metabolism](#gene138) | 22 | 33 | 0.1784782 | 4.099825e-01 | ko00920 | Metabolism | Energy metabolism |
| 139 | [Hematopoietic cell lineage](#gene139) | 3 | 3 | 0.1872054 | 4.269361e-01 | ko04640 | Organismal Systems | Immune system |
| 140 | [Hedgehog signaling pathway - fly](#gene140) | 78 | 127 | 0.1921503 | 4.350396e-01 | ko04341 | Environmental Information Processing | Signal transduction |
| 141 | [Fat digestion and absorption](#gene141) | 59 | 95 | 0.1945779 | 4.350396e-01 | ko04975 | Organismal Systems | Digestive system |
| 142 | [alpha-Linolenic acid metabolism](#gene142) | 20 | 30 | 0.1948758 | 4.350396e-01 | ko00592 | Metabolism | Lipid metabolism |
| 143 | [Antifolate resistance](#gene143) | 72 | 117 | 0.1966017 | 4.358233e-01 | ko01523 | Human Diseases | Drug resistance: Antineoplastic |
| 144 | [One carbon pool by folate](#gene144) | 23 | 35 | 0.1993711 | 4.388933e-01 | ko00670 | Metabolism | Metabolism of cofactors and vitamins |
| 145 | [Wnt signaling pathway](#gene145) | 171 | 286 | 0.2043293 | 4.467061e-01 | ko04310 | Environmental Information Processing | Signal transduction |
| 146 | [Oocyte meiosis](#gene146) | 226 | 382 | 0.2344748 | 5.090994e-01 | ko04114 | Cellular Processes | Cell growth and death |
| 147 | [Phototransduction - fly](#gene147) | 53 | 86 | 0.2364402 | 5.098744e-01 | ko04745 | Organismal Systems | Sensory system |
| 148 | [Vitamin digestion and absorption](#gene148) | 57 | 93 | 0.2453497 | 5.255125e-01 | ko04977 | Organismal Systems | Digestive system |
| 149 | [Th1 and Th2 cell differentiation](#gene149) | 74 | 122 | 0.2491478 | 5.275543e-01 | ko04658 | Organismal Systems | Immune system |
| 150 | [Signaling pathways regulating pluripotency of stem cells](#gene150) | 161 | 271 | 0.2505193 | 5.275543e-01 | ko04550 | Cellular Processes | Cellular community - eukaryotes |
| 151 | [Pantothenate and CoA biosynthesis](#gene151) | 51 | 83 | 0.2523326 | 5.275543e-01 | ko00770 | Metabolism | Metabolism of cofactors and vitamins |
| 152 | [Serotonergic synapse](#gene152) | 71 | 117 | 0.2529598 | 5.275543e-01 | ko04726 | Organismal Systems | Nervous system |
| 153 | [Non-homologous end-joining](#gene153) | 23 | 36 | 0.2622425 | 5.433390e-01 | ko03450 | Genetic Information Processing | Replication and repair |
| 154 | [Lysine degradation](#gene154) | 140 | 236 | 0.2772223 | 5.706459e-01 | ko00310 | Metabolism | Amino acid metabolism |
| 155 | [Type I diabetes mellitus](#gene155) | 9 | 13 | 0.2796435 | 5.719161e-01 | ko04940 | Human Diseases | Endocrine and metabolic diseases |
| 156 | [Glycosylphosphatidylinositol (GPI)-anchor biosynthesis](#gene156) | 40 | 65 | 0.2821247 | 5.732919e-01 | ko00563 | Metabolism | Glycan biosynthesis and metabolism |
| 157 | [Melanogenesis](#gene157) | 92 | 154 | 0.2903147 | 5.861768e-01 | ko04916 | Organismal Systems | Endocrine system |
| 158 | [Mineral absorption](#gene158) | 41 | 67 | 0.2973709 | 5.966239e-01 | ko04978 | Organismal Systems | Digestive system |
| 159 | [Cell cycle](#gene159) | 217 | 370 | 0.305299 | 6.086779e-01 | ko04110 | Cellular Processes | Cell growth and death |
| 160 | [Glycerolipid metabolism](#gene160) | 130 | 220 | 0.3099884 | 6.135782e-01 | ko00561 | Metabolism | Lipid metabolism |
| 161 | [Pertussis](#gene161) | 98 | 165 | 0.3129894 | 6.135782e-01 | ko05133 | Human Diseases | Infectious diseases: Bacterial |
| 162 | [Histidine metabolism](#gene162) | 32 | 52 | 0.3135636 | 6.135782e-01 | ko00340 | Metabolism | Amino acid metabolism |
| 163 | [Insect hormone biosynthesis](#gene163) | 16 | 25 | 0.3171874 | 6.168614e-01 | ko00981 | Metabolism | Metabolism of terpenoids and polyketides |
| 164 | [Linoleic acid metabolism](#gene164) | 13 | 20 | 0.319815 | 6.181790e-01 | ko00591 | Metabolism | Lipid metabolism |
| 165 | [Prion diseases](#gene165) | 53 | 88 | 0.3223427 | 6.192887e-01 | ko05020 | Human Diseases | Neurodegenerative diseases |
| 166 | [MicroRNAs in cancer](#gene166) | 169 | 288 | 0.3279139 | 6.261970e-01 | ko05206 | Human Diseases | Cancers: Overview |
| 167 | [Fatty acid elongation](#gene167) | 23 | 37 | 0.3314389 | 6.291385e-01 | ko00062 | Metabolism | Lipid metabolism |
| 168 | [Phototransduction](#gene168) | 40 | 66 | 0.3340196 | 6.302632e-01 | ko04744 | Organismal Systems | Sensory system |
| 169 | [Fatty acid metabolism](#gene169) | 55 | 92 | 0.3482902 | 6.533017e-01 | ko01212 | Metabolism | Global and overview maps |
| 170 | [Hypertrophic cardiomyopathy (HCM)](#gene170) | 81 | 137 | 0.3578436 | 6.653857e-01 | ko05410 | Human Diseases | Cardiovascular diseases |
| 171 | [Ribosome biogenesis in eukaryotes](#gene171) | 111 | 189 | 0.3640736 | 6.653857e-01 | ko03008 | Genetic Information Processing | Translation |
| 172 | [SNARE interactions in vesicular transport](#gene172) | 42 | 70 | 0.3645176 | 6.653857e-01 | ko04130 | Genetic Information Processing | Folding, sorting and degradation |
| 173 | [Ovarian steroidogenesis](#gene173) | 42 | 70 | 0.3645176 | 6.653857e-01 | ko04913 | Organismal Systems | Endocrine system |
| 174 | [Notch signaling pathway](#gene174) | 35 | 58 | 0.3652275 | 6.653857e-01 | ko04330 | Environmental Information Processing | Signal transduction |
| 175 | [beta-Alanine metabolism](#gene175) | 57 | 96 | 0.3737282 | 6.769819e-01 | ko00410 | Metabolism | Metabolism of other amino acids |
| 176 | [Phenylalanine metabolism](#gene176) | 29 | 48 | 0.3832791 | 6.903379e-01 | ko00360 | Metabolism | Amino acid metabolism |
| 177 | [Other types of O-glycan biosynthesis](#gene177) | 37 | 62 | 0.3978368 | 7.125100e-01 | ko00514 | Metabolism | Glycan biosynthesis and metabolism |
| 178 | [Starch and sucrose metabolism](#gene178) | 30 | 50 | 0.4014667 | 7.149716e-01 | ko00500 | Metabolism | Carbohydrate metabolism |
| 179 | [Butanoate metabolism](#gene179) | 27 | 45 | 0.4126102 | 7.307119e-01 | ko00650 | Metabolism | Carbohydrate metabolism |
| 180 | [Viral carcinogenesis](#gene180) | 290 | 502 | 0.4172025 | 7.338272e-01 | ko05203 | Human Diseases | Cancers: Overview |
| 181 | [Pyrimidine metabolism](#gene181) | 244 | 422 | 0.4189991 | 7.338272e-01 | ko00240 | Metabolism | Nucleotide metabolism |
| 182 | [Amoebiasis](#gene182) | 343 | 595 | 0.4303975 | 7.496484e-01 | ko05146 | Human Diseases | Infectious diseases: Parasitic |
| 183 | [Asthma](#gene183) | 10 | 16 | 0.43557 | 7.545120e-01 | ko05310 | Human Diseases | Immune diseases |
| 184 | [DNA replication](#gene184) | 48 | 82 | 0.4496766 | 7.747146e-01 | ko03030 | Genetic Information Processing | Replication and repair |
| 185 | [Transcriptional misregulation in cancer](#gene185) | 118 | 204 | 0.4562648 | 7.812279e-01 | ko05202 | Human Diseases | Cancers: Overview |
| 186 | [Mucin type O-glycan biosynthesis](#gene186) | 41 | 70 | 0.4585249 | 7.812279e-01 | ko00512 | Metabolism | Glycan biosynthesis and metabolism |
| 187 | [Salmonella infection](#gene187) | 314 | 546 | 0.4608505 | 7.812279e-01 | ko05132 | Human Diseases | Infectious diseases: Bacterial |
| 188 | [Base excision repair](#gene188) | 57 | 98 | 0.4663628 | 7.833717e-01 | ko03410 | Genetic Information Processing | Replication and repair |
| 189 | [Non-alcoholic fatty liver disease (NAFLD)](#gene189) | 186 | 323 | 0.4685782 | 7.833717e-01 | ko04932 | Human Diseases | Endocrine and metabolic diseases |
| 190 | [Glycerophospholipid metabolism](#gene190) | 144 | 250 | 0.4765427 | 7.833717e-01 | ko00564 | Metabolism | Lipid metabolism |
| 191 | [Lysosome](#gene191) | 148 | 257 | 0.4770876 | 7.833717e-01 | ko04142 | Cellular Processes | Transport and catabolism |
| 192 | [PPAR signaling pathway](#gene192) | 54 | 93 | 0.4771394 | 7.833717e-01 | ko03320 | Organismal Systems | Endocrine system |
| 193 | [Glycosaminoglycan biosynthesis - keratan sulfate](#gene193) | 23 | 39 | 0.4787422 | 7.833717e-01 | ko00533 | Metabolism | Glycan biosynthesis and metabolism |
| 194 | [Tight junction](#gene194) | 440 | 767 | 0.4794136 | 7.833717e-01 | ko04530 | Cellular Processes | Cellular community - eukaryotes |
| 195 | [Central carbon metabolism in cancer](#gene195) | 110 | 191 | 0.4876863 | 7.928029e-01 | ko05230 | Human Diseases | Cancers: Overview |
| 196 | [Gap junction](#gene196) | 91 | 158 | 0.4942929 | 7.971976e-01 | ko04540 | Cellular Processes | Cellular community - eukaryotes |
| 197 | [Ubiquinone and other terpenoid-quinone biosynthesis](#gene197) | 16 | 27 | 0.4954193 | 7.971976e-01 | ko00130 | Metabolism | Metabolism of cofactors and vitamins |
| 198 | [Nucleotide excision repair](#gene198) | 81 | 141 | 0.5126528 | 8.166889e-01 | ko03420 | Genetic Information Processing | Replication and repair |
| 199 | [Homologous recombination](#gene199) | 73 | 127 | 0.5126848 | 8.166889e-01 | ko03440 | Genetic Information Processing | Replication and repair |
| 200 | [Thyroid hormone synthesis](#gene200) | 17 | 29 | 0.5170919 | 8.195907e-01 | ko04918 | Organismal Systems | Endocrine system |
| 201 | [Neomycin, kanamycin and gentamicin biosynthesis](#gene201) | 5 | 8 | 0.5287174 | 8.299095e-01 | ko00524 | Metabolism | Biosynthesis of other secondary metabolites |
| 202 | [Biosynthesis of amino acids](#gene202) | 119 | 208 | 0.5288382 | 8.299095e-01 | ko01230 | Metabolism | Global and overview maps |
| 203 | [Huntington's disease](#gene203) | 229 | 401 | 0.5374071 | 8.392022e-01 | ko05016 | Human Diseases | Neurodegenerative diseases |
| 204 | [Mannose type O-glycan biosynthesis](#gene204) | 10 | 17 | 0.5484592 | 8.522626e-01 | ko00515 | Metabolism | Glycan biosynthesis and metabolism |
| 205 | [Adherens junction](#gene205) | 321 | 563 | 0.5547031 | 8.573973e-01 | ko04520 | Cellular Processes | Cellular community - eukaryotes |
| 206 | [Hippo signaling pathway - fly](#gene206) | 61 | 107 | 0.557173 | 8.573973e-01 | ko04391 | Environmental Information Processing | Signal transduction |
| 207 | [Sulfur relay system](#gene207) | 15 | 26 | 0.5624651 | 8.600619e-01 | ko04122 | Genetic Information Processing | Folding, sorting and degradation |
| 208 | [Aminoacyl-tRNA biosynthesis](#gene208) | 83 | 146 | 0.5695605 | 8.600619e-01 | ko00970 | Genetic Information Processing | Translation |
| 209 | [Taurine and hypotaurine metabolism](#gene209) | 11 | 19 | 0.5720446 | 8.600619e-01 | ko00430 | Metabolism | Metabolism of other amino acids |
| 210 | [Cell adhesion molecules (CAMs)](#gene210) | 1 | 1 | 0.5720721 | 8.600619e-01 | ko04514 | Environmental Information Processing | Signaling molecules and interaction |
| 211 | [Hedgehog signaling pathway](#gene211) | 75 | 132 | 0.5724702 | 8.600619e-01 | ko04340 | Environmental Information Processing | Signal transduction |
| 212 | [Platinum drug resistance](#gene212) | 130 | 229 | 0.5811036 | 8.689143e-01 | ko01524 | Human Diseases | Drug resistance: Antineoplastic |
| 213 | [Amyotrophic lateral sclerosis (ALS)](#gene213) | 81 | 143 | 0.5890307 | 8.766325e-01 | ko05014 | Human Diseases | Neurodegenerative diseases |
| 214 | [Cardiac muscle contraction](#gene214) | 12 | 21 | 0.5932437 | 8.781163e-01 | ko04260 | Organismal Systems | Circulatory system |
| 215 | [Alanine, aspartate and glutamate metabolism](#gene215) | 39 | 69 | 0.5957164 | 8.781163e-01 | ko00250 | Metabolism | Amino acid metabolism |
| 216 | [RNA transport](#gene216) | 1201 | 2108 | 0.5983379 | 8.781163e-01 | ko03013 | Genetic Information Processing | Translation |
| 217 | [Biotin metabolism](#gene217) | 2 | 3 | 0.607363 | 8.872538e-01 | ko00780 | Metabolism | Metabolism of cofactors and vitamins |
| 218 | [Biosynthesis of unsaturated fatty acids](#gene218) | 18 | 32 | 0.6161881 | 8.960166e-01 | ko01040 | Metabolism | Lipid metabolism |
| 219 | [Apoptosis - fly](#gene219) | 107 | 190 | 0.6276826 | 9.085634e-01 | ko04214 | Cellular Processes | Cell growth and death |
| 220 | [C5-Branched dibasic acid metabolism](#gene220) | 3 | 5 | 0.6332839 | 9.125045e-01 | ko00660 | Metabolism | Carbohydrate metabolism |
| 221 | [GABAergic synapse](#gene221) | 52 | 93 | 0.6411501 | 9.196587e-01 | ko04727 | Organismal Systems | Nervous system |
| 222 | [Glyoxylate and dicarboxylate metabolism](#gene222) | 39 | 70 | 0.6473975 | 9.214613e-01 | ko00630 | Metabolism | Carbohydrate metabolism |
| 223 | [Glycosaminoglycan biosynthesis - heparan sulfate / heparin](#gene223) | 25 | 45 | 0.6482204 | 9.214613e-01 | ko00534 | Metabolism | Glycan biosynthesis and metabolism |
| 224 | [Circadian rhythm](#gene224) | 102 | 182 | 0.6540184 | 9.255528e-01 | ko04710 | Organismal Systems | Environmental adaptation |
| 225 | [Thyroid cancer](#gene225) | 107 | 191 | 0.6585763 | 9.278608e-01 | ko05216 | Human Diseases | Cancers: Specific types |
| 226 | [Tyrosine metabolism](#gene226) | 31 | 56 | 0.6628056 | 9.296875e-01 | ko00350 | Metabolism | Amino acid metabolism |
| 227 | [Staphylococcus aureus infection](#gene227) | 5 | 9 | 0.6725648 | 9.367712e-01 | ko05150 | Human Diseases | Infectious diseases: Bacterial |
| 228 | [Fatty acid degradation](#gene228) | 51 | 92 | 0.6748638 | 9.367712e-01 | ko00071 | Metabolism | Lipid metabolism |
| 229 | [Th17 cell differentiation](#gene229) | 65 | 117 | 0.6767819 | 9.367712e-01 | ko04659 | Organismal Systems | Immune system |
| 230 | [Arginine biosynthesis](#gene230) | 12 | 22 | 0.6825122 | 9.367712e-01 | ko00220 | Metabolism | Amino acid metabolism |
| 231 | [IL-17 signaling pathway](#gene231) | 66 | 119 | 0.6849288 | 9.367712e-01 | ko04657 | Organismal Systems | Immune system |
| 232 | [Circadian rhythm - fly](#gene232) | 18 | 33 | 0.6882168 | 9.367712e-01 | ko04711 | Organismal Systems | Environmental adaptation |
| 233 | [Phenylalanine, tyrosine and tryptophan biosynthesis](#gene233) | 6 | 11 | 0.6885416 | 9.367712e-01 | ko00400 | Metabolism | Amino acid metabolism |
| 234 | [Glutamatergic synapse](#gene234) | 104 | 187 | 0.6977909 | 9.452979e-01 | ko04724 | Organismal Systems | Nervous system |
| 235 | [mRNA surveillance pathway](#gene235) | 532 | 943 | 0.7033231 | 9.475482e-01 | ko03015 | Genetic Information Processing | Translation |
| 236 | [Inositol phosphate metabolism](#gene236) | 132 | 237 | 0.7054302 | 9.475482e-01 | ko00562 | Metabolism | Carbohydrate metabolism |
| 237 | [Thiamine metabolism](#gene237) | 36 | 66 | 0.7141998 | 9.552799e-01 | ko00730 | Metabolism | Metabolism of cofactors and vitamins |
| 238 | [Riboflavin metabolism](#gene238) | 16 | 30 | 0.7317685 | 9.746664e-01 | ko00740 | Metabolism | Metabolism of cofactors and vitamins |
| 239 | [Fanconi anemia pathway](#gene239) | 72 | 132 | 0.7610064 | 1.000000e+00 | ko03460 | Genetic Information Processing | Replication and repair |
| 240 | [Glycosaminoglycan biosynthesis - chondroitin sulfate / dermatan sulfate](#gene240) | 32 | 60 | 0.7703849 | 1.000000e+00 | ko00532 | Metabolism | Glycan biosynthesis and metabolism |
| 241 | [Glycosphingolipid biosynthesis - lacto and neolacto series](#gene241) | 4 | 8 | 0.7801584 | 1.000000e+00 | ko00601 | Metabolism | Glycan biosynthesis and metabolism |
| 242 | [Cytosolic DNA-sensing pathway](#gene242) | 15 | 29 | 0.7844968 | 1.000000e+00 | ko04623 | Organismal Systems | Immune system |
| 243 | [NOD-like receptor signaling pathway](#gene243) | 91 | 167 | 0.7855807 | 1.000000e+00 | ko04621 | Organismal Systems | Immune system |
| 244 | [Primary bile acid biosynthesis](#gene244) | 2 | 4 | 0.7871643 | 1.000000e+00 | ko00120 | Metabolism | Lipid metabolism |
| 245 | [2-Oxocarboxylic acid metabolism](#gene245) | 31 | 59 | 0.8045599 | 1.000000e+00 | ko01210 | Metabolism | Global and overview maps |
| 246 | [Phosphonate and phosphinate metabolism](#gene246) | 11 | 22 | 0.8160443 | 1.000000e+00 | ko00440 | Metabolism | Metabolism of other amino acids |
| 247 | [Pentose phosphate pathway](#gene247) | 39 | 74 | 0.816655 | 1.000000e+00 | ko00030 | Metabolism | Carbohydrate metabolism |
| 248 | [Shigellosis](#gene248) | 287 | 519 | 0.8242822 | 1.000000e+00 | ko05131 | Human Diseases | Infectious diseases: Bacterial |
| 249 | [Leukocyte transendothelial migration](#gene249) | 69 | 129 | 0.8277152 | 1.000000e+00 | ko04670 | Organismal Systems | Immune system |
| 250 | [Endocrine and other factor-regulated calcium reabsorption](#gene250) | 29 | 56 | 0.8305723 | 1.000000e+00 | ko04961 | Organismal Systems | Excretory system |
| 251 | [Toll and Imd signaling pathway](#gene251) | 50 | 95 | 0.8429574 | 1.000000e+00 | ko04624 | Organismal Systems | Immune system |
| 252 | [Glycosphingolipid biosynthesis - globo and isoglobo series](#gene252) | 25 | 49 | 0.8462223 | 1.000000e+00 | ko00603 | Metabolism | Glycan biosynthesis and metabolism |
| 253 | [Alzheimer's disease](#gene253) | 153 | 282 | 0.8570011 | 1.000000e+00 | ko05010 | Human Diseases | Neurodegenerative diseases |
| 254 | [Pancreatic secretion](#gene254) | 174 | 320 | 0.8612803 | 1.000000e+00 | ko04972 | Organismal Systems | Digestive system |
| 255 | [Complement and coagulation cascades](#gene255) | 11 | 23 | 0.8684178 | 1.000000e+00 | ko04610 | Organismal Systems | Immune system |
| 256 | [Fructose and mannose metabolism](#gene256) | 69 | 131 | 0.872657 | 1.000000e+00 | ko00051 | Metabolism | Carbohydrate metabolism |
| 257 | [Valine, leucine and isoleucine biosynthesis](#gene257) | 16 | 33 | 0.8824069 | 1.000000e+00 | ko00290 | Metabolism | Amino acid metabolism |
| 258 | [Selenocompound metabolism](#gene258) | 26 | 52 | 0.882912 | 1.000000e+00 | ko00450 | Metabolism | Metabolism of other amino acids |
| 259 | [D-Arginine and D-ornithine metabolism](#gene259) | 2 | 5 | 0.8897513 | 1.000000e+00 | ko00472 | Metabolism | Metabolism of other amino acids |
| 260 | [Synthesis and degradation of ketone bodies](#gene260) | 6 | 14 | 0.9116835 | 1.000000e+00 | ko00072 | Metabolism | Lipid metabolism |
| 261 | [Ether lipid metabolism](#gene261) | 52 | 103 | 0.9301365 | 1.000000e+00 | ko00565 | Metabolism | Lipid metabolism |
| 262 | [Influenza A](#gene262) | 210 | 392 | 0.9349339 | 1.000000e+00 | ko05164 | Human Diseases | Infectious diseases: Viral |
| 263 | [Glycosaminoglycan degradation](#gene263) | 11 | 25 | 0.93714 | 1.000000e+00 | ko00531 | Metabolism | Glycan biosynthesis and metabolism |
| 264 | [Arrhythmogenic right ventricular cardiomyopathy (ARVC)](#gene264) | 9 | 21 | 0.9386908 | 1.000000e+00 | ko05412 | Human Diseases | Cardiovascular diseases |
| 265 | [Propanoate metabolism](#gene265) | 42 | 85 | 0.9405267 | 1.000000e+00 | ko00640 | Metabolism | Carbohydrate metabolism |
| 266 | [Retinol metabolism](#gene266) | 51 | 102 | 0.941665 | 1.000000e+00 | ko00830 | Metabolism | Metabolism of cofactors and vitamins |
| 267 | [Antigen processing and presentation](#gene267) | 35 | 72 | 0.9440181 | 1.000000e+00 | ko04612 | Organismal Systems | Immune system |
| 268 | [Legionellosis](#gene268) | 76 | 149 | 0.9465005 | 1.000000e+00 | ko05134 | Human Diseases | Infectious diseases: Bacterial |
| 269 | [Citrate cycle (TCA cycle)](#gene269) | 37 | 76 | 0.9467084 | 1.000000e+00 | ko00020 | Metabolism | Carbohydrate metabolism |
| 270 | [Alcoholism](#gene270) | 173 | 327 | 0.9488472 | 1.000000e+00 | ko05034 | Human Diseases | Substance dependence |
| 271 | [Primary immunodeficiency](#gene271) | 5 | 13 | 0.9496157 | 1.000000e+00 | ko05340 | Human Diseases | Immune diseases |
| 272 | [Metabolic pathways](#gene272) | 2117 | 3782 | 0.9514096 | 1.000000e+00 | ko01100 | Metabolism | Global and overview maps |
| 273 | [Glycosphingolipid biosynthesis - ganglio series](#gene273) | 31 | 65 | 0.9526167 | 1.000000e+00 | ko00604 | Metabolism | Glycan biosynthesis and metabolism |
| 274 | [Basal transcription factors](#gene274) | 88 | 172 | 0.9534307 | 1.000000e+00 | ko03022 | Genetic Information Processing | Transcription |
| 275 | [Steroid biosynthesis](#gene275) | 26 | 56 | 0.9607061 | 1.000000e+00 | ko00100 | Metabolism | Lipid metabolism |
| 276 | [Proteasome](#gene276) | 27 | 58 | 0.9613464 | 1.000000e+00 | ko03050 | Genetic Information Processing | Folding, sorting and degradation |
| 277 | [Arachidonic acid metabolism](#gene277) | 42 | 87 | 0.9630448 | 1.000000e+00 | ko00590 | Metabolism | Lipid metabolism |
| 278 | [Glycolysis / Gluconeogenesis](#gene278) | 91 | 179 | 0.9638534 | 1.000000e+00 | ko00010 | Metabolism | Carbohydrate metabolism |
| 279 | [Porphyrin and chlorophyll metabolism](#gene279) | 44 | 91 | 0.9647316 | 1.000000e+00 | ko00860 | Metabolism | Metabolism of cofactors and vitamins |
| 280 | [Malaria](#gene280) | 1 | 4 | 0.9664755 | 1.000000e+00 | ko05144 | Human Diseases | Infectious diseases: Parasitic |
| 281 | [Cytokine-cytokine receptor interaction](#gene281) | 1 | 4 | 0.9664755 | 1.000000e+00 | ko04060 | Environmental Information Processing | Signaling molecules and interaction |
| 282 | [Vasopressin-regulated water reabsorption](#gene282) | 35 | 74 | 0.9667036 | 1.000000e+00 | ko04962 | Organismal Systems | Excretory system |
| 283 | [Carbon metabolism](#gene283) | 167 | 320 | 0.9696939 | 1.000000e+00 | ko01200 | Metabolism | Global and overview maps |
| 284 | [Dilated cardiomyopathy](#gene284) | 2 | 7 | 0.9727959 | 1.000000e+00 | ko05414 | Human Diseases | Cardiovascular diseases |
| 285 | [Bacterial invasion of epithelial cells](#gene285) | 323 | 605 | 0.9747381 | 1.000000e+00 | ko05100 | Human Diseases | Infectious diseases: Bacterial |
| 286 | [Glycine, serine and threonine metabolism](#gene286) | 48 | 100 | 0.9747858 | 1.000000e+00 | ko00260 | Metabolism | Amino acid metabolism |
| 287 | [Folate biosynthesis](#gene287) | 6 | 17 | 0.9805948 | 1.000000e+00 | ko00790 | Metabolism | Metabolism of cofactors and vitamins |
| 288 | [Other glycan degradation](#gene288) | 19 | 45 | 0.9850769 | 1.000000e+00 | ko00511 | Metabolism | Glycan biosynthesis and metabolism |
| 289 | [Viral myocarditis](#gene289) | 41 | 89 | 0.9868796 | 1.000000e+00 | ko05416 | Human Diseases | Cardiovascular diseases |
| 290 | [Sphingolipid metabolism](#gene290) | 77 | 158 | 0.9870786 | 1.000000e+00 | ko00600 | Metabolism | Lipid metabolism |
| 291 | [p53 signaling pathway](#gene291) | 68 | 141 | 0.9872868 | 1.000000e+00 | ko04115 | Cellular Processes | Cell growth and death |
| 292 | [Oxidative phosphorylation](#gene292) | 50 | 109 | 0.9934197 | 1.000000e+00 | ko00190 | Metabolism | Energy metabolism |
| 293 | [Valine, leucine and isoleucine degradation](#gene293) | 68 | 144 | 0.9937673 | 1.000000e+00 | ko00280 | Metabolism | Amino acid metabolism |
| 294 | [African trypanosomiasis](#gene294) | 1 | 6 | 0.9938635 | 1.000000e+00 | ko05143 | Human Diseases | Infectious diseases: Parasitic |
| 295 | [Vitamin B6 metabolism](#gene295) | 9 | 26 | 0.9941326 | 1.000000e+00 | ko00750 | Metabolism | Metabolism of cofactors and vitamins |
| 296 | [Cocaine addiction](#gene296) | 3 | 12 | 0.9949172 | 1.000000e+00 | ko05030 | Human Diseases | Substance dependence |
| 297 | [Cysteine and methionine metabolism](#gene297) | 58 | 126 | 0.9955533 | 1.000000e+00 | ko00270 | Metabolism | Amino acid metabolism |
| 298 | [Galactose metabolism](#gene298) | 50 | 111 | 0.9962637 | 1.000000e+00 | ko00052 | Metabolism | Carbohydrate metabolism |
| 299 | [Endocytosis](#gene299) | 642 | 1201 | 0.9966038 | 1.000000e+00 | ko04144 | Cellular Processes | Transport and catabolism |
| 300 | [Pyruvate metabolism](#gene300) | 73 | 157 | 0.9973204 | 1.000000e+00 | ko00620 | Metabolism | Carbohydrate metabolism |
| 301 | [Neuroactive ligand-receptor interaction](#gene301) | 76 | 163 | 0.9974566 | 1.000000e+00 | ko04080 | Environmental Information Processing | Signaling molecules and interaction |
| 302 | [Protein processing in endoplasmic reticulum](#gene302) | 406 | 776 | 0.9975745 | 1.000000e+00 | ko04141 | Genetic Information Processing | Folding, sorting and degradation |
| 303 | [Apoptosis - multiple species](#gene303) | 6 | 21 | 0.9980181 | 1.000000e+00 | ko04215 | Cellular Processes | Cell growth and death |
| 304 | [Ascorbate and aldarate metabolism](#gene304) | 30 | 73 | 0.9980539 | 1.000000e+00 | ko00053 | Metabolism | Carbohydrate metabolism |
| 305 | [Nitrogen metabolism](#gene305) | 24 | 61 | 0.9983356 | 1.000000e+00 | ko00910 | Metabolism | Energy metabolism |
| 306 | [Tryptophan metabolism](#gene306) | 45 | 108 | 0.9995909 | 1.000000e+00 | ko00380 | Metabolism | Amino acid metabolism |
| 307 | [Parkinson's disease](#gene307) | 50 | 119 | 0.999695 | 1.000000e+00 | ko05012 | Human Diseases | Neurodegenerative diseases |
| 308 | [Glutathione metabolism](#gene308) | 47 | 119 | 0.9999658 | 1.000000e+00 | ko00480 | Metabolism | Metabolism of other amino acids |
| 309 | [Drug metabolism - other enzymes](#gene309) | 44 | 113 | 0.9999689 | 1.000000e+00 | ko00983 | Metabolism | Xenobiotics biodegradation and metabolism |
| 310 | [Metabolism of xenobiotics by cytochrome P450](#gene310) | 42 | 111 | 0.9999869 | 1.000000e+00 | ko00980 | Metabolism | Xenobiotics biodegradation and metabolism |
| 311 | [Spliceosome](#gene311) | 419 | 837 | 0.9999896 | 1.000000e+00 | ko03040 | Genetic Information Processing | Transcription |
| 312 | [Steroid hormone biosynthesis](#gene312) | 38 | 104 | 0.999993 | 1.000000e+00 | ko00140 | Metabolism | Lipid metabolism |
| 313 | [Pentose and glucuronate interconversions](#gene313) | 34 | 99 | 0.9999986 | 1.000000e+00 | ko00040 | Metabolism | Carbohydrate metabolism |
| 314 | [Drug metabolism - cytochrome P450](#gene314) | 40 | 115 | 0.9999996 | 1.000000e+00 | ko00982 | Metabolism | Xenobiotics biodegradation and metabolism |
| 315 | [Chemical carcinogenesis](#gene315) | 42 | 121 | 0.9999998 | 1.000000e+00 | ko05204 | Human Diseases | Cancers: Overview |
| 316 | [Systemic lupus erythematosus](#gene316) | 46 | 150 | 1 | 1.000000e+00 | ko05322 | Human Diseases | Immune diseases |
| 317 | [Ribosome](#gene317) | 104 | 353 | 1 | 1.000000e+00 | ko03010 | Genetic Information Processing | Translation |

| **#** | **Pathway** | **Differentially expressed genes** |
| --- | --- | --- |
| 1 | [Dorso-ventral axis formation](../../../AppData/Local/Temp/Temp1_VETR-D-18-00038.zip/Cont-vs-PPRV_DEGseq_map/map04320.html) | XM_018049493.1, XM_018048562.1, XM_018051528.1, XM_018040418.1, XM_005678301.3, XM_018044791.1, XM_018053826.1, XM_018047086.1, XM_018052253.1, XM_018045096.1, XM_018060029.1, XM_018040731.1, XM_018063858.1, XM_018061709.1, XM_018044459.1, XM_013967784.2, XM_005700737.3, XM_013970310.2, XM_018040298.1, XM_018053075.1, XM_018050649.1, XM_018041060.1, XM_005681861.3, XM_018053399.1, XM_018065555.1, XM_005699859.3, XM_013962440.2, XM_018040552.1, XM_018054543.1, XM_013964626.2, XM_018061386.1, XM_018066592.1, XM_018064594.1, XM_013970805.2, XM_018050144.1, XM_018061381.1, XM_018041618.1, XM_018043802.1, XM_018057437.1, XM_018046546.1, XM_013975137.2, XM_018064273.1, XM_018051176.1, XM_018044959.1, XM_018067249.1, XM_018054650.1, XM_018045097.1, XM_018054130.1, XM_018038866.1, XM_018047823.1, XM_018043086.1, XM_018061296.1, XM_018061124.1, XM_018065378.1, XM_018058359.1, XM_018060694.1, XM_005680678.3, XM_018066144.1, XM_018051542.1, XM_018047246.1, XM_018062585.1, XM_018051564.1, XM_018065447.1, XM_018044947.1, XM_018045480.1, XM_018056899.1, XM_013964768.2, XM_018057598.1, XM_018062584.1, XM_018041795.1, XM_018050456.1, XM_018038338.1, XM_018048588.1, XM_018050804.1, XM_018038797.1, XM_018057808.1, XM_005692471.3, XM_018056824.1, XM_018064269.1, XM_018045736.1, XM_018051536.1, XR_001917871.1, XM_018066618.1, XM_018066176.1, XM_018062575.1, XM_018040508.1, XM_018045156.1, XM_018040389.1, XM_018058356.1, XM_018062083.1, XM_018066127.1, XM_018063060.1, XM_005676392.3, XM_018051730.1, XM_018053415.1, XM_005701394.3, XM_018038940.1, XM_018061650.1, XM_018064961.1, XM_013965615.2, XM_018052251.1, XM_018065644.1, XM_018064980.1, XM_018051197.1, XM_018060868.1, XM_018040497.1, XM_018050413.1, XM_018048585.1, XM_018039510.1, XM_018050582.1, XR_001919354.1, XM_018053744.1, XM_018067242.1, XM_018041175.1, XM_018060913.1, XR_001919793.1, XM_018038762.1, XM_018064485.1, XM_018046004.1, XM_018059341.1, XM_018053266.1, XM_018048589.1, NM_001285755.1, XM_018047962.1, XM_018041956.1, XM_018040073.1, XM_018060012.1, XM_018050437.1, XM_018049741.1, XM_018040583.1, XM_018066051.1, XM_018061190.1, XM_018044052.1, XM_005690878.3, XM_018063101.1, XR_001918069.1, XM_005690516.3, XM_013970207.2, XM_018057729.1, XM_018043084.1, XM_018051238.1, XM_018038667.1, XM_018060023.1, XR_001919524.1, XM_018052689.1, XM_018057569.1, XM_018050764.1, XM_018048795.1, XM_018040738.1, XM_018065060.1, XM_018065283.1, XM_018050759.1, XM_018059376.1, XM_018064011.1, XM_018062593.1, XR_001918144.1, XM_018060424.1, XM_018064517.1, XM_018044680.1, XM_018063658.1, XM_018065643.1, XM_018039323.1, XM_018051566.1, XM_018040157.1, XM_018056704.1, XM_018059886.1, XM_018051179.1, XM_018059085.1, XM_018057576.1, XM_018058948.1, XM_018052504.1, XM_018051162.1, XM_018042454.1, XM_018052684.1, XM_018061389.1, XM_018059340.1, XM_018043415.1, XM_018038883.1, XM_018061205.1, XM_018060025.1, XM_018065253.1, XM_018065857.1, XM_018047824.1, XM_018066746.1, XM_018060401.1, XM_018061503.1, XM_018063768.1, XM_018038432.1, XM_018046249.1, XM_018040660.1, XM_018050987.1, XM_018060026.1, XM_013966112.2, XM_005698034.3, XM_018064971.1, XM_005698303.3, XM_018060948.1, XM_018043041.1, XM_018047819.1, XM_018062514.1, XM_018057039.1, XM_018039066.1, XM_018062428.1, XM_018040156.1, XM_005685130.3, XR_001919608.1, XM_018062313.1, XM_018051793.1, XR_001919753.1, XM_018055157.1, XM_018058423.1, XM_018038335.1, XR_001919543.1, XM_018048718.1, XM_018051175.1, XM_018062301.1, XM_005681645.3, NM_001314325.1, XM_018048644.1, XM_018064816.1, XM_018057706.1, XM_018061592.1, XM_018040023.1, XM_018039727.1, XM_018057742.1, XM_018051518.1, XM_018042878.1, XM_018057898.1, XM_018066646.1, XM_018039816.1, XM_018059240.1, XM_013974207.2, XM_018055667.1, XM_018051526.1, XM_018049792.1, XM_018060744.1, XM_018040419.1, XM_013971557.2, XM_018051619.1, XM_013972442.2, XM_018066222.1, XM_018056679.1, XR_001917489.1, XM_018051230.1, XM_018040022.1, XM_018057807.1, XM_018057185.1, XM_018061334.1, XM_018050813.1, XM_018043882.1, XM_018050583.1, XM_018059124.1, XM_018055048.1, XM_018050249.1, XM_018060399.1, XM_018057452.1, XM_018065280.1, XM_018043117.1, XM_018046976.1, XM_018063794.1, XM_018054454.1, XM_018067247.1, XM_018051315.1, XM_005678599.3, XM_018038960.1, XM_018059711.1, XM_018039053.1, XM_018040938.1, XM_018044974.1, XM_018053265.1, XM_013969841.2, XM_018048446.1, XM_013976822.2, XM_005678298.3, XM_018064492.1, XM_018038334.1, XM_018053927.1, XM_005692399.3, XM_018060396.1, XR_310645.3, XM_018050402.1, XM_018054271.1, XM_018043537.1, XM_018051237.1, XM_018057545.1, XM_018038757.1, XM_018050293.1, XM_018062561.1, XM_018063950.1, XM_018040724.1, XM_018055350.1, XM_018053748.1, XM_018051439.1, XM_018066050.1, XM_018038864.1, XM_018051527.1, XM_018063967.1, XM_018060215.1, XM_018064576.1, XM_018050146.1, XM_018056216.1, XM_018040507.1, XM_018039601.1, XM_018038778.1, XM_018066505.1, XM_018057533.1, XM_018057186.1, XM_018059807.1, XM_018047965.1, XM_018043099.1, XM_018048959.1, XM_013976810.2, XM_018050103.1, XM_018054499.1, XM_018061177.1, XM_018058358.1, XM_018063132.1, XM_018049610.1, XM_018053312.1, XM_018060385.1, XM_018044985.1, XM_018059219.1, XM_018051309.1, XM_018049482.1, XM_018063048.1, XM_018053765.1, XM_018038272.1, XM_018054084.1, XM_018060011.1, XM_018045432.1, XM_018064814.1, XM_018064818.1, XM_018043202.1, XM_018054720.1, XM_018057539.1, XM_018064486.1, XM_018065254.1, XM_018043435.1, XM_018051539.1, XM_018065702.1, XR_001919193.1, XM_018045235.1, XM_018060676.1, XM_018062659.1, XM_018042883.1, XM_018055880.1, XM_018040016.1, XM_018039098.1, XM_018043173.1, XM_018045887.1, XM_018043040.1, XM_018045376.1, XM_018052707.1, XM_018039640.1, XM_005680921.3, XM_018062837.1, XM_018042428.1, XM_018061072.1, XM_018052685.1, XM_018066669.1, XM_013976823.2, XM_018045154.1, XM_005678338.3, XM_018065639.1, XM_018049822.1, XM_005681590.3, XM_005698620.3, XM_018051521.1, XM_018040842.1, XM_018040812.1, XM_018051203.1, XM_018051534.1, XM_018060030.1, XM_018049611.1, XR_001918866.1, XM_018040184.1, XM_018059075.1, XM_018047259.1, XM_018057030.1, XM_018043102.1, XM_005688041.3, XM_018064809.1, XM_018051560.1, XM_018044957.1, XM_018066325.1, XM_018041233.1, XM_018053306.1, XM_018058576.1, XM_018046308.1, XM_018045428.1, XM_018061248.1, XM_018067245.1, XM_018048934.1, XM_018063948.1, XM_018046252.1, XM_018066502.1, XM_018058947.1, XM_018040024.1, XM_018041455.1, XM_018043527.1, XM_018063890.1, XM_018063233.1, XM_013966744.2, XM_018058758.1, XM_018060858.1, XM_018057436.1, XM_018054832.1, XM_018066590.1, XM_018061251.1, XM_018039861.1, XM_005685868.3, XM_018058354.1, XR_001918874.1, XM_018065287.1, XM_018062390.1, XM_018039596.1, XM_018042657.1, XM_018065933.1, XM_018061125.1, XM_018042850.1, XM_018040498.1, XM_018064788.1, XM_018058347.1, XM_018064294.1, XM_018041080.1, XM_018057038.1, XM_018057158.1, XM_018064815.1, XM_018064490.1, XM_018049742.1, XM_018038871.1, XM_018052391.1, XM_005693232.3, XM_018052055.1, XM_018051229.1, XM_018048587.1, XM_018046003.1, XM_018058334.1, XM_018060730.1, XM_005679940.3, XM_018039054.1, XM_018051546.1, XM_013973792.2, XM_018041287.1, XM_005684541.3, XM_013966136.2, XM_018060400.1, XM_018063064.1, XM_018051537.1, XM_018060783.1, XM_018057077.1, XM_018066036.1, XM_018045374.1, XM_018066044.1, XM_018051350.1, XM_018050976.1, XM_018065265.1, XM_018041452.1, XM_018060018.1, XM_018045031.1, XM_013964822.2, XR_001919850.1, XM_005682058.3, XM_018053321.1, XM_018061385.1, XM_018038749.1, XM_018055040.1, XM_018056330.1, XM_018057536.1, XM_018040505.1, XR_001918461.1, XM_018059732.1, XM_018048715.1, XM_018062610.1, XM_018065286.1, XM_018063792.1, XM_018050415.1, XM_018040701.1, XM_018058973.1, XM_018056321.1, XM_018044929.1, XM_018056793.1, XM_005693431.3, XM_013967703.2, XM_018060009.1, XM_018041470.1, XM_018057703.1, XM_018040501.1, XM_018050889.1, XM_018040417.1, XR_001919087.1, XM_018042734.1, XM_018063537.1, XM_018064333.1, XM_018040074.1, XM_018048547.1, XM_018061250.1, XM_018051531.1, XM_018058500.1, XR_001918353.1, XM_018054701.1, XM_018062679.1, XM_018060014.1, XR_001918881.1, XM_005694239.3, XR_001918190.1, XM_018041179.1, XM_018042851.1, XM_018058723.1, XM_018060020.1, XM_013966685.2, XM_018064332.1, XM_018048716.1, XR_001918307.1, XM_018060650.1, XR_001918308.1, XM_018043072.1, XR_001919200.1, XM_018062588.1, XM_018062488.1, XM_018038748.1, XM_013969300.2, XM_018044587.1, XM_018055520.1, XR_001919318.1, XM_005699873.3, XM_018051220.1, XR_001919089.1, XM_018066606.1, XM_018044070.1, XM_018059577.1, XM_018059656.1, XM_018039371.1, XM_018064020.1, XM_018038587.1, XM_013967229.2, XM_018059733.1, XM_018043092.1, XM_018051248.1, XM_018039998.1, XM_013965259.2, XM_018041937.1, XM_005688157.3, XM_018048285.1, XM_018040997.1, XM_018062132.1, XM_018047818.1, XM_018062387.1, XM_018064146.1, XM_018053523.1, XM_018040020.1, XR_001918701.1, XM_018043805.1, XM_018057183.1, XM_018053400.1, XM_018060313.1, XM_018046064.1, XM_018064787.1, XM_018054666.1, XM_018054723.1, XM_013975535.2, XM_018057184.1, XM_018064322.1, XM_018043580.1, XM_018067184.1, XM_018038333.1, XM_018051232.1, XM_018051226.1, XM_018055537.1, XM_018067125.1, XM_005674736.2, XM_005695421.3, XM_018062847.1, XM_018042777.1, XM_018059286.1, XM_018039600.1, XM_018049357.1, XM_018062430.1, XM_018058660.1, XM_018059117.1, XM_018066647.1, XM_018064114.1, XM_018056620.1, XM_018063946.1, XM_018045287.1, XM_018067083.1, XM_018047442.1, XM_018049356.1, XM_018050459.1, XM_018062465.1, XM_018051553.1, XM_018062310.1, XM_018041695.1, XM_018040813.1, XM_018048723.1, XM_018064789.1, XM_018053969.1, XM_018056811.1, XR_001919953.1, XM_018040652.1, XM_018061191.1, XM_018066219.1, XM_018066614.1, XM_005683859.3, XM_005696533.3, XM_018065296.1, XM_018045094.1, XM_018057438.1, XM_018062364.1, XM_018045540.1, XM_018039599.1, XM_018040874.1, XM_018063683.1, XM_018056100.1, XM_018051540.1, XM_005675355.3, XM_018046250.1, XM_018051622.1, XM_018050758.1, XR_001918140.1, XM_018063813.1, XM_018053404.1, XM_018062497.1, NM_001287041.1, XM_005688338.3, XM_018063661.1, XM_005699333.2, XR_001918965.1, XM_018062467.1, XM_018060260.1, XM_018062589.1, XM_018053397.1, XM_018038271.1, XM_018043402.1, XM_018041998.1, XM_018038346.1, XM_018051794.1, XM_005683953.3, XM_013969468.2, XM_018058673.1, XM_018060606.1, XM_018042656.1, XM_018038961.1, XM_018059982.1, XM_018042847.1, XM_018040809.1, XM_018039582.1, XM_013966473.2, XM_018064590.1, XM_018065557.1, XM_018043403.1, XM_018060270.1, XM_018067084.1, XM_018065377.1, XM_018043748.1, XM_018050147.1, XM_013966684.2, XR_001917179.1, XM_018039355.1, XM_018043408.1, XM_018060867.1, XM_005681863.3, XM_005689854.3, XM_005696194.3, XM_018044588.1, XM_005679360.3, XM_018062704.1, XM_005677668.3, XM_018048111.1, XM_018045942.1, XM_018044372.1, XM_018040187.1, XM_018059439.1, XM_018051227.1, XM_018062345.1, XM_018065993.1, XM_018044705.1, XM_018043201.1, XM_018056648.1, XM_018059116.1, XM_005679138.3, XM_018064279.1, XM_018064559.1, XM_018051225.1, NM_001314247.1, XR_001918215.1, XR_001919632.1, XM_018060008.1, XM_018052954.1, XM_018038799.1, XM_018050411.1, XM_018056327.1, XM_018053403.1, XM_018038341.1, XM_018053743.1, XM_018054206.1, XM_018056481.1, XM_018041889.1, XM_018051177.1, XM_018065721.1, XM_018056686.1, XM_018061715.1, XR_001919357.1, XM_018058870.1, XM_018061025.1, XM_018048999.1, XM_018050811.1, XM_018044776.1, XM_018049689.1, XM_018039050.1, XM_018046708.1, XM_018062691.1, XM_018040581.1, XM_018039819.1, XM_018042110.1, XM_018047718.1, XM_018061302.1, XM_018062311.1, XM_005700145.2, XM_018044373.1, XM_018063839.1, XM_018063130.1, XM_018039134.1, XM_018067113.1, XM_018065361.1, XM_018038890.1, XM_018066641.1, XM_018042723.1, XM_018065298.1, XM_018051555.1, XM_018044565.1, XM_005696172.3, XM_018040021.1, XM_018065558.1, XM_018051557.1, XM_018038497.1, XM_018052777.1, XM_018046555.1, XM_005699968.3, XM_018060634.1, XM_018040503.1, XM_018052505.1, XM_018051223.1, XM_018065369.1, XM_018055025.1, XM_018057567.1, XM_018048956.1, XM_018044493.1, XM_018066607.1, XM_018049072.1, XM_018061666.1, XM_018040520.1, XM_018066587.1, XM_018059287.1, XM_018038742.1, XM_018038682.1, XM_018051565.1, XM_018062579.1, XM_018065642.1, XM_018050643.1, XM_018040811.1, XM_018048939.1, XM_005693770.3, XM_018065292.1, XM_018050101.1, XM_018042500.1, XM_018062469.1, XM_018047841.1, XM_018060402.1, XM_018063975.1, XM_018047247.1, XM_018042724.1, XM_018049829.1, XM_018045098.1, XM_013966683.2, XM_018047964.1, XR_001918880.1, XM_018042141.1, XM_018043495.1, XM_018050011.1, XM_018053745.1, XM_018043149.1, XM_018060013.1, XM_018066336.1, XM_018063893.1, XM_018058696.1, XM_018040810.1, XM_018066093.1, XM_018038743.1, XM_018050644.1, XM_018065734.1, XM_018058355.1, XR_001919696.1, XM_018064596.1, XM_018066500.1, XM_018043398.1, XM_018060403.1, XM_005693787.2, XM_018053711.1, XM_018043110.1, XM_018048734.1, XM_018056775.1, XM_013964769.2, XM_005691626.3, XM_018045014.1, XM_018056940.1, XM_018041286.1, XM_018064810.1, XM_018052238.1, XR_001919869.1, XM_018057032.1, XM_018065862.1, XM_018042201.1, XM_018044958.1, XM_018065297.1, XM_018044344.1, XM_018047737.1, XM_018042429.1, XM_018064488.1, XM_018048024.1, XM_018048554.1, XM_018058945.1, XM_018043467.1, XM_018054838.1, XM_018066049.1, XM_018061035.1, XM_018039516.1, XM_018062795.1, XM_018061249.1, XM_018042846.1, XM_018040086.1, XM_018065722.1, XM_018053742.1, XM_018043406.1, XM_018064745.1, XM_018050253.1, XM_018043436.1, XM_005686101.3, XM_018050544.1, XM_018041174.1, XM_018044260.1, XM_018066644.1, XM_018045375.1, XM_018041457.1, XM_018056941.1, XM_018041176.1, XR_001919952.1, XM_018041244.1, XM_018051563.1, XM_018056653.1, XM_005675781.3, XR_001919198.1, XM_018039584.1, XM_018043538.1, XM_018050805.1, XM_018058175.1, XM_018052100.1, XM_018046723.1, XM_018058697.1, XM_018059313.1, XM_018044086.1, XM_018052570.1, XM_018057188.1, XM_018065365.1, XM_018053264.1, XM_018064910.1, XM_018057029.1, XM_018047244.1, XM_013962425.2, XM_018055369.1, XM_018048945.1, XM_018039534.1, XM_018065368.1, XM_018064813.1, XM_013962863.2, XR_001919684.1, XM_018054291.1, XM_018038347.1, XM_018058936.1, XM_018056877.1, XM_018065746.1, XM_018054651.1, XM_018040933.1, XM_018054749.1, XM_018066238.1, XM_018041254.1, XM_018057741.1, XM_018052239.1, XM_018047468.1, XM_018053263.1, XM_018040519.1, XM_018052267.1, XM_018063289.1, XM_018045743.1, XM_018053709.1, XM_018063663.1, NM_001314202.1, XM_018047646.1, XM_018060024.1, XM_005690517.3, XM_018058975.1, XM_018059112.1, XM_018043089.1, XM_018054843.1, XM_013964033.2, XM_018040553.1, XM_018060221.1, XM_018042121.1, XM_018049427.1, XM_018052324.1, XM_018051550.1, XM_005693536.3, XM_018052686.1, XM_018048224.1, XM_018053747.1, XM_018066043.1, XM_018043216.1, XM_018066645.1, XM_005698229.3, XM_018062131.1, XM_018038687.1, XM_018051247.1, XM_018047042.1, XM_018061079.1, XM_018062754.1, XM_018043401.1, XM_018063549.1, XM_018047594.1, XM_018048952.1, XR_001919356.1, XM_018038345.1, XM_018058338.1, XM_018048721.1, XM_013962921.2, XM_018043409.1, XM_018041955.1, XM_018050294.1, XR_001919298.1, XM_018039633.1, XM_018051523.1, XM_018065293.1, XM_018040875.1, XM_018050409.1, XM_018038746.1, XM_018049609.1, XM_018050255.1, XM_018064238.1, XM_018048583.1, XM_018062560.1, XM_018051525.1, XM_018047457.1, XM_018061500.1, XM_018050815.1, XM_018050742.1, XM_018039639.1, XM_018049079.1, XM_018065863.1, XM_018040771.1, XM_013970799.2, XM_018046735.1, XM_018049791.1, XM_018053733.1, XM_018040582.1, XM_018044998.1, XM_013967213.2, XM_005677202.3, XM_018039509.1, XM_013962879.2, XM_018048591.1, XM_018051544.1, XM_018053402.1, XM_018059259.1, XM_018052266.1, XM_018043115.1, XM_018057819.1, XM_018050143.1, XM_018049481.1, XM_018054596.1, XM_018060213.1, XM_018050412.1, XM_018049691.1, XM_018045559.1, XM_018046146.1, XM_018051541.1, XM_018065281.1, XM_018063983.1, XM_018059114.1, XM_018052056.1, XM_018057302.1, XM_018045247.1, XM_018057538.1, XM_018066983.1, XM_018065288.1, XM_018049719.1, XM_018052065.1, XM_018063133.1, XM_018051442.1, XM_018065290.1, XM_018040103.1, XM_018059917.1, XM_018064744.1, XM_018063818.1, XM_018038585.1, XM_018062130.1, XM_018038342.1, XM_018066268.1, XM_018051559.1, XM_018060027.1, XM_018054614.1, XM_018040266.1, XM_018049839.1, XM_018063118.1, XM_005698076.3, XM_018066313.1, XM_018038744.1, XM_018042008.1, XM_018050581.1, XM_018057181.1, XM_013969267.2, XM_018063949.1, XM_018051549.1, XM_018065299.1, XM_018051520.1, XM_018051832.1, XM_018059113.1, XM_018059108.1, XM_018047882.1, XM_018049897.1, XM_018046740.1, XM_018045093.1, XM_018050756.1, XM_018061391.1, XM_018053427.1, XM_018061844.1, XM_018064898.1, XM_018056200.1, XM_005692642.3, XM_018052756.1, XM_018043756.1, XM_018064268.1, XM_018062458.1, XM_018064487.1, XM_018061057.1, XM_018039532.1, XM_018065661.1, XM_018051543.1, XM_013973909.2, XM_018054905.1, XM_018042706.1, XM_018050856.1, XM_018057033.1, XM_018041454.1, XM_018057034.1, XM_018054831.1, XM_018066596.1, XM_018050847.1, XM_018056199.1, XM_018041180.1, XM_018043220.1, XM_018050761.1, XM_013971101.2, XM_013973450.2, XM_018046564.1, XR_001295711.2, XM_018062577.1, XM_018043106.1, XM_013976220.2, XM_018051968.1, XM_018043494.1, XM_018054740.1, XM_018059710.1, XM_013966453.2, XM_018051440.1, XM_018050650.1, XM_018064276.1, XM_018042219.1, XM_018044905.1, XM_005685763.3, XR_001919088.1, XM_005677667.3, XM_018039238.1, XM_018052687.1, XM_018057540.1, XM_018048647.1, XM_018048383.1, XM_018066145.1, XM_018051524.1, XM_018038352.1, XM_018057035.1, XR_001919607.1, XM_018038921.1, XM_018064582.1, XM_018065238.1, XM_018043010.1, XR_001919199.1, XM_018051558.1, XM_005692787.3, XM_018038776.1, XM_018049492.1, XM_005696535.3, XM_018056039.1, XM_018060397.1, XM_018066957.1, XM_018039324.1, XM_018057552.1, NM_001314319.1, XM_018054090.1, XM_018053324.1, XM_018050953.1, XM_018053336.1, XR_001917234.1, XM_018064384.1, XM_018052916.1, XM_018061078.1, XM_018066034.1, XM_018054745.1, XM_018063662.1, XM_018053027.1, XM_018066982.1, XM_018038339.1, XM_018040500.1, XM_018045007.1, XM_018042259.1, XR_001918763.1, XM_018039224.1, XR_001918334.1, XM_018038865.1, XM_018050812.1, XM_018056680.1, XM_018040394.1, XM_018040181.1, XR_001919817.1, XM_018066649.1, XM_018064270.1, XM_018043039.1, XM_018062538.1, XM_018045461.1, XM_005683769.3, XM_018056939.1, XM_018048780.1, XM_018046713.1, XM_005683226.3, XM_018050252.1, XM_018052032.1, XM_018048310.1, XM_018044871.1, XM_018044528.1, XM_018060962.1, XM_018066640.1, XM_013964037.2, XM_018062466.1, XM_018042924.1, XM_018058993.1, XM_018061842.1, XM_018052287.1, XM_013967424.2, XM_018059440.1, XR_001918595.1, XM_018049863.1, XM_018047249.1, XM_018060849.1, XM_018050102.1, XM_018053483.1, XM_018063274.1, XM_018051199.1, XM_005687059.2, XM_018056498.1, XM_018039970.1, XM_018045150.1, XM_005690491.3, XM_013971476.2, XM_018059339.1, XM_005698386.3, XM_018066504.1, XM_018054868.1, XM_005676771.3, XM_018039595.1, XM_018062346.1, XM_018059696.1, XM_018051547.1, XM_005699822.3, XM_018052304.1, XM_018049002.1, XM_018038351.1, XM_018048023.1, XM_018060398.1, XM_005680373.3, XM_018065397.1, XM_018046831.1, XM_018062429.1, XM_018066979.1, XM_018063037.1, XM_018062582.1, XM_018053401.1, XM_018066223.1, XM_018059107.1, XM_018051493.1, XM_018041616.1, XM_018041252.1, XR_001919197.1, XM_018059712.1, XM_005698302.3, XM_018040946.1, XM_018058573.1, XM_018056038.1, XM_018043085.1, XM_005676796.3, XM_018061382.1, XM_018050975.1, XM_018043108.1, XM_018054501.1, XM_018056646.1, XM_018050458.1, XM_005677991.3, XM_018040658.1, XM_018061845.1, XM_018055174.1, XM_018063973.1, XM_005693774.3, XM_018039293.1, XM_018065029.1, XM_018051243.1, XM_018052545.1, XM_018067212.1, XM_018038783.1, XM_018050142.1, XM_018046083.1, XM_018051574.1, XM_018062513.1, XM_018045155.1, XM_018038473.1, XM_018049358.1, XM_018038349.1, XM_018059078.1, XM_018061774.1, XM_018057537.1, XM_018056783.1, XM_018063891.1, XM_018057908.1, XM_018059699.1, XM_018046124.1, XM_018042200.1, XM_005687047.3, XM_018065025.1, XM_018044996.1, XM_018062468.1, XM_018062344.1, XM_018043407.1, XM_018046378.1, XM_018045022.1, XM_018058676.1, XM_018060869.1, XM_018065284.1, XM_018047041.1, XM_018061387.1, XR_001919202.1, XM_018062562.1, XM_018062576.1, XM_018045373.1, XM_018044437.1, XM_018038836.1, XM_018058328.1, XM_013964649.2, XM_018059655.1, XM_018051545.1, XM_018067248.1, XM_018051438.1, XM_018038239.1, XM_018054500.1, XM_018065719.1, XM_013969054.2, XM_018038837.1, XM_018051233.1, XM_018048717.1, XM_018048586.1, XM_018040053.1, XM_018052669.1, XR_001919201.1, XM_018044781.1, XM_018062336.1, XM_018041456.1, XM_018051165.1, XM_018064742.1, XR_001918679.1, XM_018051163.1, XM_018053746.1, XM_018053565.1, XM_018043405.1, XM_018044775.1, XM_018059125.1, XM_018051231.1, XM_018046655.1, XM_018057912.1, XM_018048606.1, XM_018041253.1, XM_018050401.1, XM_018064383.1, XM_018043383.1, XM_005685146.3, XM_018050414.1, XM_018059324.1, XM_018053225.1, XM_018038818.1, XM_018054613.1, XM_018057669.1, XM_018064672.1, XM_018050307.1, XM_018067246.1, XM_018038817.1, XM_018054908.1, XM_018061502.1, XM_018059109.1, XM_018065743.1, XM_005681862.2, XM_018044089.1, XM_018038496.1, XM_018065285.1, XM_013974948.2, XM_018061380.1, XM_018039055.1, XM_005677421.3, XM_018040661.1, XM_018047092.1, XM_018052688.1, XM_018063131.1, XM_018046646.1, XM_018060416.1, XM_018062690.1, XM_018066053.1, XM_018050400.1, XM_018062153.1, XM_018056336.1, XM_018049739.1, XM_018045410.1, XM_018063937.1, XM_018054761.1, XM_018051299.1, XM_005695002.3, XM_018048951.1, XM_018056341.1, XM_013962439.2, XM_018064812.1, XM_013973268.2, XM_018058502.1, XM_005680278.3, XM_018061333.1, XM_018060228.1, XM_013967109.2, XM_018050292.1, XM_018062254.1, XM_018042007.1, XM_018062794.1, XM_018038745.1, XM_018065375.1, XR_001918462.1, XM_018065119.1, XM_018053710.1, NM_001314207.1, XM_018039049.1, XM_018042252.1, XM_018062314.1, XM_018057451.1, XM_018042808.1, XM_018062637.1, XM_018040499.1, XM_005685918.3, XM_018041181.1, XM_018060841.1, XM_018057031.1, XM_018059115.1, XR_001919175.1, XM_018054763.1, XM_018038336.1, XM_018054129.1, XM_018054746.1, XM_018059191.1, XM_018056093.1, XM_018053261.1, XM_018039658.1, XM_018038331.1, XM_018051517.1, XM_018039598.1, XM_018064491.1, XR_001918875.1, XM_018058668.1, XM_018064671.1, XM_018051561.1, XM_018050757.1, XM_018054584.1, XM_018065022.1, XM_018039945.1, XM_018051236.1, XM_013964774.2, XM_018062312.1, XM_018063269.1, XM_018051249.1, XM_018063103.1, XM_018056650.1, XM_018056647.1, XM_018052942.1, XM_018064746.1, XM_018051533.1, XM_018059981.1, XM_018050645.1, XM_018054413.1, XM_018066300.1, XM_018043293.1, XM_005682121.3, XR_001919828.1, XM_018059077.1, XM_018057848.1, XM_018043438.1, XM_005676775.3, XM_018058458.1, XM_018045087.1, XM_018057257.1, XM_018049718.1, XM_018062611.1, XM_018047245.1, XM_018063981.1, XM_018045244.1, XM_018066826.1, XM_018066980.1, XM_018060827.1, XM_018058327.1, XM_005684479.2, XM_018043380.1, XM_018045099.1, XM_018062434.1, XM_018045158.1, XM_018047552.1, XM_005697652.3, XM_005692029.2, XM_018047817.1, XM_018065295.1, XM_018050755.1, XM_018061104.1, XM_018049003.1, XM_018065371.1, XM_018043071.1, XM_018042925.1, XM_018056599.1, XM_018051752.1, XM_018049740.1, XM_018044629.1, XM_018065282.1, XM_018053320.1, XM_018066210.1, XM_018038791.1, XM_018045013.1, XM_005692419.3, XM_005687000.3, XM_018042991.1, XM_018061393.1, XM_018039786.1, XM_013968151.2, XM_018039321.1, XM_018060870.1, XM_013976470.2, XM_018061501.1, XM_005675353.3, XM_018052360.1, XM_005675134.3, XM_005694472.3, XM_018051222.1, XM_018051164.1, XM_018051441.1, XM_018043357.1, XM_018038340.1, XM_018057454.1, XM_005690518.3, XM_018046235.1, XM_018044495.1, XM_018053426.1, XM_018061668.1, XM_018064786.1, XM_018043763.1, XM_018066589.1, XM_018045015.1, XM_018060019.1, XM_018057439.1, XM_018040869.1, XM_018060420.1, XM_018061597.1, XM_018055534.1, XR_001918644.1, XM_018051532.1, XM_018047488.1, XM_018066038.1, XM_018057543.1, XM_018053740.1, XM_018065479.1, XM_018051659.1, XM_018042486.1, XM_018051334.1, XM_013976360.2, XM_018062746.1, XM_013964573.2, XM_018045515.1, XM_018061388.1, XM_018054602.1, XM_018066643.1, XM_018040415.1, XM_018055778.1, XM_018048643.1, XM_018055023.1, XM_018038350.1, XR_001918843.1, XM_018042760.1, XM_018051556.1, XM_018038846.1, XM_018054750.1, XM_018038863.1, XM_018050008.1, XM_013964770.2, XM_018051554.1, XM_018042558.1, XM_018048413.1, XM_018053395.1, XM_018048940.1, NM_001285739.1, XM_018045411.1, XM_018064059.1, XM_018043399.1, XM_018043400.1, XM_018051178.1, XM_013966454.2, XM_018056885.1, XM_018064779.1, XM_018067213.1, XM_018056522.1, XM_018059248.1, XM_018063956.1, XM_018038892.1, XM_018066591.1, XM_013975985.2, XM_018038847.1, XM_018059654.1, XM_018047692.1, XM_018060384.1, XM_005701339.3, XM_018064581.1, XM_018042554.1, XM_018051160.1, XM_018042258.1, XM_018062444.1, XM_018041617.1, XM_018066220.1, XM_018066829.1, XM_005685609.3, XM_018048584.1, XM_018047644.1, XM_018065723.1, XM_005690519.3, XM_018038891.1, XM_018059111.1, XM_018050652.1, XM_018044566.1, XM_018061516.1, XM_018058572.1, XM_018057563.1, XM_018048719.1, XM_018065646.1, XM_018052563.1, XM_018057849.1, XM_018064115.1, XM_018040076.1, XM_018064817.1, XM_018060212.1, XM_018065638.1, XM_018038884.1, XM_005688206.2, XM_018046084.1, XM_018040061.1, XM_018058144.1, XM_018049738.1, XM_018061782.1, XM_018057042.1, XM_018065992.1, XM_005678989.3, XM_018038584.1, XM_018052054.1, XM_018062343.1, XM_018054649.1, XM_018045308.1, XM_018042550.1, XM_018042762.1, XM_018049000.1, XM_018038344.1, XM_018050508.1, XM_018058659.1, XM_005678965.3, XM_018051224.1, XM_018047642.1, XM_018043101.1, XM_018048942.1, XM_018041078.1, XM_018043434.1, XM_013962765.2, XM_018051522.1, XM_018046858.1, XM_018061297.1, XM_018051241.1, XM_018047816.1, XM_018057810.1, XM_018062563.1, XM_018051312.1, XM_018055642.1, XM_018064985.1, XM_018049495.1, XM_018046299.1, XR_001917415.1, XM_018051552.1, XM_018066146.1, XM_018054762.1, XM_018043404.1, XM_018041022.1, XM_018045406.1, XM_018051831.1, XM_018066221.1, XR_001919090.1, XM_018059123.1, XM_018063838.1, XM_018061448.1, XM_018047585.1, XM_018064219.1, XM_018066981.1, XM_018055878.1, XM_018054606.1, XM_018056272.1, XM_018044585.1, XM_018057036.1, XM_018058357.1, XM_018039511.1, XM_018049633.1, XM_018064278.1, XM_018053840.1, XM_018045479.1, XR_001917462.1, XM_018051485.1, XM_005694107.3, XM_018058325.1, XM_018045095.1, XM_018047840.1, XM_018039597.1, XM_018041255.1, XM_018061065.1, XM_018057453.1, XM_018038332.1, XM_018051538.1, XM_005677806.3, XM_018043414.1, XM_018057847.1, XM_018056179.1, XM_018041715.1, XM_018040814.1, XM_018047825.1, XM_018064004.1, XM_018054726.1, XM_005681893.3, XM_018065157.1, XM_018048998.1, XM_018038343.1, XR_001919742.1, XR_001917728.1, XM_018063656.1, XM_018040322.1, XM_018064292.1, XM_013971792.2, XM_018046857.1, XM_018060427.1, XM_018048722.1, XM_018061303.1, XM_018041086.1, XM_018051530.1, XM_018051228.1, XM_018038750.1, XM_018065291.1, XM_018060022.1, XM_018051529.1, XM_018046150.1, XM_018064277.1, XM_018040886.1, XM_018050763.1, XM_018065946.1, XM_018039789.1, XM_018056157.1, XM_018046727.1, XM_018050651.1, XM_018042907.1, XM_018042848.1, XM_018040323.1, XM_005679428.3, XM_005688201.3, NM_001285569.1, XM_018057589.1, XM_018041587.1, XM_018038788.1, XM_018061609.1, XM_018066642.1, XM_018062425.1, XM_018053323.1, XM_018057541.1, XM_018062663.1, XR_001918699.1, XM_018040000.1, XM_018038353.1, XM_018046720.1, XM_018053262.1, XM_018056320.1, XM_018044248.1, XM_018064574.1, XM_018065477.1, XM_018041730.1, XM_018039650.1, XM_018043433.1, XM_018056802.1, XM_018045153.1, XM_018038199.1, XM_013974996.2, XM_018065294.1, XR_001919358.1, XM_013973964.2, XM_018062831.1, XM_018040320.1, XM_018064147.1, XM_018066605.1, XM_018051792.1, XM_018045593.1, XM_018038330.1, XM_018054478.1, XM_005696532.3, XM_018045969.1, XM_018041453.1, XM_018064974.1, XM_018061395.1, XM_005685829.3, XM_018039052.1, XM_018065289.1, XM_018064548.1, XM_018052706.1, XM_018057544.1, XM_018060124.1, XM_018040659.1, XM_018042250.1, XM_018060426.1, XM_018051240.1, XM_018052059.1, XM_018040506.1, XM_018066471.1, XM_018066402.1, XM_018051234.1, XM_018062657.1, XM_018049354.1, XM_018060616.1, XM_018044946.1, XM_018045192.1, XM_018050773.1, XM_005679137.3, XM_018060216.1, XM_018053392.1, XM_018040504.1, XM_018039940.1, XM_018051221.1, XM_013962422.2, XM_018060007.1, XM_018061499.1, XM_018050915.1, XM_018054241.1, XM_018050971.1, XM_018054038.1, XM_018064274.1, XM_018051310.1, XM_018047883.1, XM_018062708.1, XM_018061840.1, XM_013976300.2, XM_018052495.1, XM_018051548.1, XM_018042440.1, XM_018045419.1, XM_018042849.1, XM_018061059.1, XM_018067250.1, XM_018055173.1, XM_018050746.1, XM_005685145.3, XM_018061390.1, XM_018042776.1, XM_018049774.1, XM_018045030.1, XM_018038417.1, XM_005696330.3, XR_001917218.1, XM_018039641.1, XM_018043757.1, XR_001919525.1, XM_018041528.1, XM_018063620.1, XM_018059503.1, XM_018052573.1, XM_018049737.1, XM_018064820.1, XM_018065707.1, XM_018043091.1, XR_001918764.1, XM_018065604.1, XM_018059203.1, XM_018061396.1, XM_018050295.1, XM_018065810.1, XM_013962679.2, XM_018051161.1, XR_001918557.1, XM_018039352.1, XM_018045187.1, XM_018042703.1, XM_018051795.1, XM_018058020.1, XM_018061225.1, XM_013962438.2 |
| 2 | [cAMP signaling pathway](../../../AppData/Local/Temp/Temp1_VETR-D-18-00038.zip/Cont-vs-PPRV_DEGseq_map/map04024.html) | XM_018062643.1, XM_018062483.1, XM_005684795.2, XM_013963001.2, XM_005681861.3, XM_018064273.1, XM_018061294.1, XM_018061124.1, XM_018045824.1, XM_013964768.2, XM_018039549.1, XM_018056824.1, XM_005679127.2, XM_018052298.1, XM_018044120.1, XM_018062382.1, XM_005680366.2, XM_018063971.1, XM_018052301.1, XM_018047786.1, XM_018066762.1, XM_018042311.1, XM_018061293.1, XM_018063035.1, XM_018044119.1, XM_018054045.1, XM_018055911.1, XM_018058967.1, XM_018055487.1, XM_005676159.3, XM_018053453.1, XM_005697610.3, XM_018055187.1, XM_018050987.1, XM_018056305.1, XM_005685130.3, XM_018061289.1, XM_005697075.3, XM_018058454.1, XM_005691447.3, XM_018054459.1, XM_018062471.1, XM_018066370.1, XM_018060378.1, XM_018051619.1, XM_018061256.1, XM_018054460.1, XM_005682114.3, XM_013972607.2, XM_018045956.1, XM_018044121.1, XM_018051048.1, XM_005680364.3, XM_005684794.3, XM_018060016.1, XM_005694678.3, XR_001918973.1, XM_005699952.3, XM_018043534.1, XM_018042310.1, XM_013972972.2, XM_018061295.1, XM_018048959.1, XM_018062407.1, XM_018062408.1, XM_018058962.1, XM_013965100.2, XM_018053228.1, XM_018042428.1, XM_005681590.3, XM_018062484.1, XM_018054461.1, XM_018047802.1, XM_018063293.1, XR_001918038.1, XM_013974872.2, XM_018061125.1, XM_018047231.1, XM_018042282.1, XM_005699064.3, XM_018058500.1, XM_018043396.1, NM_001286443.1, XM_018061292.1, XM_005680365.2, XM_005683158.3, XM_005699065.3, XM_018042308.1, XM_018047616.1, XM_018039561.1, XM_018060141.1, XM_005694677.3, XM_018056811.1, XM_018062410.1, XM_013968554.2, XM_018065511.1, XM_018051622.1, XM_005700661.3, XM_005688518.3, XM_018041016.1, XM_018058447.1, XM_005694675.3, XM_005684782.3, XM_013964590.2, XM_018040436.1, XM_018060376.1, XM_018054462.1, XM_005681863.3, XM_018047613.1, XM_018045231.1, XM_005679129.3, XM_018064279.1, XM_018054206.1, XM_018043047.1, XM_018042281.1, XM_018052297.1, XM_005700662.3, XM_018049689.1, XM_018045443.1, XM_005700145.2, XM_018051049.1, XM_018045830.1, XM_005691412.3, XM_013968553.2, XM_005675289.3, XM_018048956.1, XM_018065817.1, XM_018043394.1, XM_005679333.3, XM_018066184.1, XM_018044122.1, XM_018059609.1, XR_001918220.1, XM_013964769.2, XM_018042429.1, XM_018066186.1, XM_018042307.1, XM_018064745.1, XM_018061288.1, XM_018066371.1, XM_018051046.1, XM_018049271.1, XM_018057813.1, XM_018052300.1, XM_018063036.1, NM_001314202.1, XM_018055485.1, XM_018060488.1, XM_018049427.1, XM_018052324.1, XM_018042279.1, XM_018048812.1, XM_013967227.2, XM_018048952.1, NM_001285750.1, XM_013970799.2, XM_018047636.1, XM_018049891.1, XM_005681533.3, XM_018055908.1, XM_018051050.1, XM_018049691.1, XM_018045201.1, XM_018043593.1, XM_018062409.1, XR_001295516.2, XM_018058966.1, XM_018066613.1, XM_018050856.1, XM_005680800.3, XM_018042278.1, XM_018065509.1, XM_018062482.1, XM_018064276.1, XM_005685763.3, XM_005692304.2, XM_018046304.1, XM_005690882.3, XM_005680367.3, XM_018065816.1, XM_018055186.1, XM_018054083.1, XM_005676485.3, XM_018042280.1, XM_005685150.3, XM_013975781.2, XM_005683226.3, XM_013964453.2, XM_005686574.3, XM_013966779.2, XM_005684793.3, XM_018060379.1, XM_018042309.1, XM_005680676.3, XM_018045834.1, XM_005680373.3, XM_005682112.2, XM_005677233.3, XM_018058968.1, XM_013970271.2, XM_018051722.1, XM_018049794.1, XM_013968877.2, XM_005675332.3, XM_005685146.3, XM_018064672.1, XM_005681862.2, XM_005688564.3, XM_018066760.1, XM_018051721.1, XM_018060261.1, XR_001919756.1, XM_013966645.2, XM_018048951.1, XM_018058502.1, XM_013963332.2, XM_005677232.3, XM_018065510.1, XM_018066761.1, XR_001296033.2, XM_018064671.1, XM_018043395.1, XM_005683159.3, XM_018055488.1, XM_013964774.2, XM_018058965.1, XM_018047617.1, XM_018064746.1, XM_018064499.1, XM_018048420.1, XM_018061291.1, XM_018060377.1, XM_018057613.1, XM_018038791.1, XM_018058970.1, XM_005694472.3, XM_018065433.1, XM_018062470.1, XM_018054044.1, XM_018061286.1, XM_013964770.2, XM_005676482.3, XM_018055912.1, XM_018049270.1, XM_005685149.3, XM_018045239.1, XM_018058455.1, NM_001285685.1, XM_018066759.1, XM_018067051.1, XM_018064278.1, XM_018042304.1, XM_018042305.1, XM_005697074.3, XM_018066185.1, XM_005680013.2, XM_018047638.1, XM_018064292.1, XM_018055486.1, XM_018064277.1, XM_018053454.1, XM_018038788.1, XM_005675916.3, XM_018053231.1, XM_018056518.1, XM_005675891.3, XM_018042303.1, XM_018045593.1, XM_013976047.2, XM_005685953.3, XM_018054431.1, XM_018051719.1, XM_018066763.1, XM_018064274.1, XM_005685145.3, XM_005694349.2, XM_005699951.1, XM_005699502.2, XM_018047637.1, XM_018060142.1, XM_005678304.3, XM_018053232.1 |
| 3 | [Endocrine resistance](../../../AppData/Local/Temp/Temp1_VETR-D-18-00038.zip/Cont-vs-PPRV_DEGseq_map/map01522.html) | XM_018055645.1, XM_005681861.3, XM_018055614.1, XM_018064273.1, XM_018041850.1, XM_013964768.2, XM_018056451.1, XM_018056824.1, XM_005679127.2, XM_018063971.1, NM_001285748.1, XM_013970381.2, XM_018063035.1, XM_018064710.1, XM_018053461.1, XM_018055911.1, XM_005683400.3, XM_005697610.3, XM_005683110.3, XM_018047989.1, XM_018055187.1, XM_018050987.1, XM_005685130.3, XM_018060378.1, XM_018051619.1, XM_018054430.1, XM_018064703.1, XM_018060016.1, XM_005692453.3, XM_018048959.1, XM_018062407.1, XM_018062408.1, XM_018043792.1, XM_018053228.1, XM_018042428.1, XM_018058171.1, XM_005681590.3, XM_018064712.1, XM_018043463.1, XM_018064718.1, XM_018051085.1, XM_013974872.2, XM_018064705.1, XM_018041856.1, XM_018047231.1, XM_018053460.1, XM_018064707.1, XM_018058500.1, XM_018064716.1, XM_018050610.1, XR_001918229.1, XM_018056811.1, XM_018062410.1, XM_005675490.2, XM_013968554.2, XM_018051622.1, XM_005698535.3, XM_005689267.3, XM_018058447.1, XM_005684782.3, XM_018042871.1, XM_018060376.1, XM_005681863.3, XM_005699630.3, XM_018060738.1, XM_005679129.3, XM_018064279.1, XM_018053462.1, XM_018054206.1, XM_018049689.1, XM_005700145.2, XM_013968553.2, XM_005675289.3, XM_018048956.1, XM_018065817.1, XM_018049858.1, XM_018041851.1, XM_018043794.1, XM_018043791.1, XM_018059609.1, XM_013964769.2, XM_018042429.1, XM_018064702.1, XM_018056690.1, XM_018064745.1, XM_018064711.1, XM_018041853.1, XM_018064714.1, XM_018064715.1, XM_018063036.1, XR_001917360.1, NM_001314202.1, XM_018060488.1, XM_018049427.1, XM_018060739.1, XM_013967227.2, XM_018048952.1, NM_001285641.1, NM_001285750.1, XM_018055908.1, XM_005701006.3, XM_018049691.1, XM_018063793.1, XM_018062409.1, XM_018053966.1, XM_018064713.1, XM_005686996.3, XM_018059919.1, XM_018050856.1, XM_018039227.1, XM_018041852.1, XM_018039226.1, XM_018064276.1, XM_005685763.3, XM_005693259.3, XM_018065816.1, XM_018055186.1, XM_018054083.1, XM_005683226.3, XM_018064699.1, XM_018060379.1, XM_005680373.3, XM_018060766.1, XM_018064700.1, XR_001918230.1, XM_013970271.2, XM_013968877.2, XM_005678171.3, XM_005675332.3, XM_005685146.3, XM_013971256.2, XM_018064672.1, XM_005681862.2, XM_005688564.3, XM_018060261.1, XM_018058170.1, XM_018048951.1, XM_018049857.1, XM_018058502.1, XM_013975391.2, XM_018049721.1, XM_018053831.1, XM_018064671.1, XM_013964774.2, XM_018064746.1, XM_018050609.1, XM_018064708.1, XR_001918228.1, XM_018048420.1, XM_018060377.1, XM_018038791.1, XM_018043793.1, XM_005694472.3, XM_018060089.1, XM_018064709.1, XM_018061071.1, XM_013965671.2, XM_013964770.2, XM_018053830.1, XM_005678170.3, XM_018041854.1, XM_018061516.1, XM_018055912.1, XM_018052765.1, XM_018063972.1, XM_018067051.1, XM_018059918.1, XM_018064278.1, XM_018063570.1, XM_018064292.1, XM_018064704.1, XM_018064277.1, XM_013968876.2, XM_018038788.1, XM_018053231.1, XM_018064701.1, XM_018045593.1, XM_018064717.1, XM_018043795.1, XM_018041855.1, XM_018060765.1, XM_018064274.1, XM_005685145.3, XM_005691053.3, XR_001918643.1, XM_018053232.1 |
| 4 | [Inflammatory mediator regulation of TRP channels](../../../AppData/Local/Temp/Temp1_VETR-D-18-00038.zip/Cont-vs-PPRV_DEGseq_map/map04750.html) | XM_018062643.1, XM_013963001.2, XM_018061124.1, XM_018045824.1, XM_005679127.2, XM_018052298.1, XM_005680366.2, XM_018052301.1, XM_018042311.1, XM_018054045.1, XM_018058967.1, XM_018055487.1, XM_018054409.1, XR_001918100.1, XM_005680737.3, XM_005697075.3, XM_005691447.3, XM_018066370.1, XM_018060378.1, XM_018061256.1, XM_013972607.2, XM_005680364.3, XM_018060016.1, XR_001918973.1, XM_005699952.3, XM_018042310.1, XM_018058962.1, XM_018061125.1, XM_018047231.1, XM_005680365.2, XM_018054401.1, XM_005690990.2, XM_018042308.1, XM_018060732.1, XM_018039561.1, XM_005688518.3, XM_018041016.1, XM_018060376.1, XM_005679129.3, XM_018043047.1, XM_018052297.1, XM_018045830.1, XM_005675289.3, XM_018043394.1, XM_005679333.3, XM_018059609.1, XM_018054405.1, XM_018042307.1, XM_018054406.1, XM_018066371.1, XM_018049271.1, XM_018057813.1, XM_018052300.1, XM_018055485.1, XM_013970799.2, XM_018043593.1, XR_001295516.2, XM_018058966.1, XM_005680367.3, XM_005676485.3, XM_005685150.3, XM_018054402.1, XM_005686574.3, XM_018054408.1, XM_013966779.2, XM_018060379.1, XM_018042309.1, XM_018045834.1, XM_018058968.1, XM_018054403.1, XM_005685526.3, XR_001296033.2, XM_018055488.1, XM_018058965.1, XM_018048420.1, XM_018060377.1, XM_018058970.1, XM_018054044.1, XM_005676482.3, XM_018054407.1, XM_018049270.1, XM_005685149.3, XM_018042304.1, XM_018042305.1, XM_005697074.3, XM_018055486.1, XM_018042303.1, XM_013976047.2, XM_005694349.2, XM_005699951.1 |
| 5 | [ABC transporters](../../../AppData/Local/Temp/Temp1_VETR-D-18-00038.zip/Cont-vs-PPRV_DEGseq_map/map02010.html) | XM_018050968.1, XM_013963305.2, XM_018053976.1, XM_018042167.1, XM_018040951.1, XM_013969141.2, XM_018050473.1, XM_018039108.1, XM_005696445.3, XM_018056322.1, XM_018044689.1, XM_018046732.1, XM_018040430.1, XM_018056175.1, XM_018063931.1, XM_018056305.1, XM_018047375.1, XM_018040918.1, XM_018063921.1, XM_005676007.3, XM_018050965.1, XM_018063922.1, XM_018063916.1, XM_018047358.1, XR_001917993.1, XM_005675201.3, XM_018040735.1, XM_005686074.3, XM_018059677.1, XM_018050475.1, XM_013975121.2, XM_018047350.1, XM_018059814.1, XM_005686547.3, XM_018049142.1, XM_018056290.1, XM_018064859.1, XM_018047362.1, XM_005680782.3, XM_005684263.3, XM_013964174.2, XM_018044777.1, XM_018040432.1, XM_018050972.1, XM_013963869.2, XM_013967600.2, XM_018050963.1, XM_005689540.3, XM_018045789.1, XM_018064856.1, XM_018056301.1, XM_018052335.1, XM_018038798.1, XM_018056447.1, XM_018047305.1, XM_018056291.1, XM_005684265.3, XM_018040917.1, XM_005698320.3, XM_018049141.1, XM_018054168.1, XM_005676573.3, XM_018063923.1, XM_018045443.1, XM_018040429.1, XM_018052333.1, XM_018050967.1, XM_018056445.1, XM_018046517.1, XM_005698318.3, XM_018064858.1, XM_018056296.1, XM_018039107.1, XM_018063932.1, XM_018064857.1, XM_018049138.1, XM_005680780.3, XM_018052542.1, XM_005680781.3, XM_018056176.1, XM_018049137.1, XM_018063925.1, XM_018056300.1, XM_018063926.1, XM_018040427.1, XM_018047382.1, XM_013964130.2, XM_018039866.1, XM_018063266.1, XM_018040428.1, XM_018050472.1, XM_013967599.2, XM_013969130.2, XM_013975120.2, XM_018056297.1, XM_013969124.2, XM_018063180.1, XM_018047300.1, XM_018056174.1, XM_018045598.1, XM_018056446.1, XM_018063918.1, XM_005696334.3, XM_018050961.1, XM_018052334.1, XM_018050966.1, XM_018056443.1, XM_018050969.1, XM_018056287.1, XM_005700613.3, XM_018056304.1, XR_001917308.1, XM_018050962.1, XM_018047299.1, XM_018063915.1, XM_018047508.1, XM_018056286.1, XM_018050964.1, XM_005691954.3, XM_018050474.1, XM_005691383.3, XM_018059874.1, XM_018063917.1, XM_018056441.1, XR_001297176.2, XM_018063919.1, XM_018059676.1, NM_001285707.1, XM_018045782.1, XM_018047353.1, XM_018056274.1, XM_013969127.2, XM_005676008.3, XM_018059613.1, XM_018056323.1, XM_018046731.1, XM_018052543.1, XM_018063927.1, XM_018063924.1, XM_013969135.2, XM_005679028.2, XM_018063914.1, XM_018049140.1, XM_018050471.1, XM_018053229.1, XM_018053178.1, XM_018050971.1, XM_018049143.1 |
| 6 | [ErbB signaling pathway](../../../AppData/Local/Temp/Temp1_VETR-D-18-00038.zip/Cont-vs-PPRV_DEGseq_map/map04012.html) | XM_005681861.3, XM_018043204.1, XM_018064273.1, XM_018045824.1, XM_013964768.2, XM_018056451.1, XM_018056824.1, XM_005679127.2, XM_018044120.1, XM_018062382.1, XM_018063971.1, NM_001285748.1, XM_013970381.2, XM_018063035.1, XM_018044119.1, XM_018053461.1, XM_018055911.1, XM_018058967.1, XM_005697610.3, XM_005683110.3, XM_018047989.1, XM_018055187.1, XM_018050987.1, XM_005685130.3, XM_018054459.1, XM_018060378.1, XM_018051619.1, XM_018054460.1, XM_018044121.1, XM_018060016.1, XM_018048959.1, XM_018062407.1, XM_018062408.1, XM_018058962.1, XM_018043792.1, XM_018053228.1, XM_018042428.1, XM_005681590.3, XM_018043463.1, XM_018054461.1, XM_013974872.2, XM_018047231.1, XM_018053460.1, XM_018058500.1, XM_018043396.1, XM_018050610.1, XR_001918229.1, XM_018056811.1, XM_018062410.1, XM_005675490.2, XM_013968554.2, XM_018051622.1, XM_005698535.3, XM_018058447.1, XM_005684782.3, XM_018042871.1, XM_018060376.1, XM_018054462.1, XM_005681863.3, XM_018060738.1, XM_005679129.3, XM_018064279.1, XM_018053462.1, XM_018054206.1, XM_018049689.1, XM_005700145.2, XM_018045830.1, XM_013968553.2, XM_005675289.3, XM_018048956.1, XM_018065817.1, XM_018049858.1, XM_018044122.1, XM_018043794.1, XM_018043791.1, XM_018059609.1, XM_013964769.2, XM_018042429.1, XM_018064745.1, XM_018063036.1, NM_001314202.1, XM_018060488.1, XM_018049427.1, XM_018060739.1, XM_013967227.2, XM_018048952.1, NM_001285641.1, NM_001285750.1, XM_018055908.1, XM_005701006.3, XM_018049691.1, XM_018062409.1, XM_018053966.1, XM_018059919.1, XM_018058966.1, XM_018050856.1, XM_005674982.3, XM_018064276.1, XM_005685763.3, XM_005693259.3, XM_018046304.1, XM_018065816.1, XM_018055186.1, XM_018054083.1, XM_013975781.2, XM_005683226.3, XM_018060379.1, XM_018045834.1, XM_005680373.3, XR_001918230.1, XM_018058968.1, XM_013970271.2, XM_013968877.2, XM_005678171.3, XM_005675332.3, XM_005685146.3, XM_018064672.1, XM_005681862.2, XM_005688564.3, XM_018060261.1, XM_018048951.1, XM_018049857.1, XM_018058502.1, XM_018049721.1, XM_018053831.1, XM_018064671.1, XM_018043395.1, XM_013964774.2, XM_018058965.1, XM_018064746.1, XM_018050609.1, XR_001918228.1, XM_018048420.1, XM_018060377.1, XM_018038791.1, XM_018043793.1, XM_018058970.1, XM_005694472.3, XM_018060089.1, XM_018061071.1, XM_013965671.2, XM_013964770.2, XM_018053830.1, XM_005678170.3, XM_018055912.1, XM_018063972.1, XM_018067051.1, XM_018059918.1, XM_018064278.1, XM_018063570.1, XM_018064292.1, XM_018064277.1, XM_013968876.2, XM_018038788.1, XM_018053231.1, XM_018045593.1, XM_018043795.1, XM_018043122.1, XM_018064274.1, XM_005685145.3, XM_005699502.2, XR_001918643.1, XM_018053232.1 |
| 7 | [Epstein-Barr virus infection](../../../AppData/Local/Temp/Temp1_VETR-D-18-00038.zip/Cont-vs-PPRV_DEGseq_map/map05169.html) | XM_018040418.1, XM_018044791.1, XM_018055981.1, XM_018051001.1, XM_018040731.1, XM_018045893.1, XM_018046773.1, XM_018055678.1, XM_018049587.1, XM_018060429.1, XM_018043204.1, XM_018043802.1, XM_018066843.1, XM_013968498.2, XM_018060921.1, XM_018051579.1, XM_018065920.1, XM_018041834.1, XM_018049799.1, XM_018043213.1, XM_018055452.1, XM_005679127.2, XM_018048701.1, XM_018056270.1, XM_018066694.1, XM_005687164.3, XM_018048309.1, XM_018063971.1, XM_018044847.1, XM_018041491.1, XM_018038998.1, XM_018055404.1, XR_001917204.1, XM_018063638.1, XM_018062732.1, XM_005698856.3, XM_018063035.1, XM_018048669.1, XM_018048665.1, XM_018056088.1, XM_018055821.1, XM_018055911.1, XM_018050764.1, XM_018050759.1, XM_018043210.1, XM_005689869.3, XM_018063307.1, XM_018055453.1, XM_018040157.1, XM_018066619.1, XM_018047106.1, XM_018052935.1, XR_001917205.1, XM_005697610.3, XM_018063034.1, NM_001314233.1, XM_018044427.1, XM_018055187.1, XM_018059129.1, XM_013966112.2, XM_018048699.1, XM_018066672.1, XM_018044499.1, XM_018040861.1, XM_018044799.1, XM_018055458.1, XM_018044318.1, XM_018043238.1, XR_311047.3, XM_018048903.1, XM_013972144.2, XM_018056229.1, XM_018061397.1, XM_018064666.1, XM_018054734.1, XM_018062848.1, XM_005692310.3, XM_005680058.3, XM_018060378.1, XM_018060568.1, XM_005674994.3, XM_018054468.1, XM_018047900.1, XM_018062478.1, XM_018058314.1, XM_005696338.3, NM_001314206.1, XM_018055124.1, XM_018051685.1, NM_001314212.1, XM_018063860.1, XM_013964929.2, XM_018065915.1, XM_018051498.1, XM_018047426.1, XM_005680266.3, XM_018052677.1, XM_018060016.1, XM_018065063.1, XM_018060051.1, XM_018051292.1, XR_001918077.1, XM_005680985.3, XM_005677745.3, XM_018043239.1, XM_018059641.1, XM_018058552.1, XM_018044825.1, XM_013973350.2, XM_013975423.2, XM_018060045.1, XM_018059759.1, XM_018048566.1, XM_018062407.1, XM_013975121.2, XM_018060727.1, XM_018062408.1, XM_018052940.1, XM_018039500.1, XM_005679901.2, XM_018053696.1, XM_018053228.1, XM_013976482.2, XM_018044989.1, XM_018057132.1, XM_018065067.1, XM_018045851.1, XM_018044823.1, XM_018041187.1, XM_018040184.1, XM_005682995.3, XM_018063955.1, XM_018058588.1, XM_018055405.1, XM_018046659.1, XM_018053628.1, XM_013974872.2, XM_005680575.3, XM_018042531.1, XM_018039932.1, XM_005675237.3, XM_018047477.1, XM_005695611.3, XM_018064747.1, XM_018039864.1, XM_018047231.1, XM_018050762.1, XM_013974836.2, XM_018065891.1, XR_001918377.1, XR_001917004.1, XM_018064677.1, XM_013972522.2, XM_018052497.1, XM_018056235.1, XM_018059547.1, XM_018039286.1, XM_018038777.1, XM_018043378.1, XM_018053634.1, XM_005692309.3, XM_018048567.1, XM_005680937.3, XM_005677744.3, XM_018040345.1, XR_001917206.1, XM_013969885.2, XM_005693625.3, XM_018065072.1, XM_005694020.3, XM_018060468.1, XM_018049801.1, XM_018041902.1, XM_018043212.1, XM_018048694.1, XM_018059761.1, XM_018043092.1, XM_018050945.1, XM_018041394.1, XM_018064395.1, NM_001314303.1, XM_018059128.1, XR_001917207.1, XM_018043805.1, XM_005679654.2, XM_018059549.1, XM_005676377.3, XM_018041836.1, XM_013976502.2, XM_018053989.1, XM_018057275.1, XM_018062410.1, XM_018046774.1, XM_005683062.3, XM_018040509.1, XM_018065249.1, XM_013968554.2, XM_018061752.1, XM_013963623.2, XM_018057688.1, XM_018066540.1, XM_018058667.1, XM_018059762.1, XM_018041685.1, XM_018058447.1, XM_005684782.3, XM_018062155.1, XM_013963116.2, XR_001917488.1, XM_018063309.1, XM_018039498.1, XR_001917492.1, XM_005675184.3, XM_018060376.1, XM_018054322.1, XM_018061420.1, XM_018055459.1, XM_018048565.1, XM_018062270.1, XM_005696194.3, XM_018066563.1, XM_018044372.1, XM_018048564.1, XM_005675838.3, XM_005679129.3, XM_013976483.2, XM_018058233.1, XM_018038230.1, XM_018046771.1, XM_018049835.1, XM_018046315.1, XM_018059130.1, XM_018063773.1, XM_018056090.1, XM_018048692.1, XM_018063965.1, XM_018046817.1, XM_013968553.2, XM_018048568.1, XM_018039284.1, XM_018056032.1, XM_005675289.3, XM_018048691.1, XM_018065817.1, XM_018039929.1, XM_005681064.3, XM_018043354.1, XM_005698318.3, XM_018041686.1, XM_013968589.2, XM_018046772.1, XM_018044198.1, XM_018048340.1, XM_018059609.1, XM_018057759.1, XM_018055454.1, XM_018041929.1, XM_018041044.1, XM_005691599.3, XM_018051293.1, XM_018048569.1, XM_018059198.1, XM_018060043.1, XM_018062405.1, XM_018046661.1, XM_018062158.1, XM_018062271.1, XM_018045892.1, XM_018047425.1, XM_018057585.1, XM_018063036.1, XM_005679432.3, XM_018039860.1, XM_018050760.1, XM_018060488.1, XM_018043089.1, XR_001296084.2, XM_018055449.1, XM_018055456.1, XM_005695113.3, XM_018048812.1, XM_013967227.2, XM_018056227.1, XM_005676052.2, XM_018066692.1, XM_018062464.1, XM_018062152.1, XM_018065066.1, XM_005682900.3, NM_001285750.1, XM_018051684.1, XM_018056269.1, XM_013966480.2, XM_018041107.1, XM_018055349.1, XM_018066739.1, XM_018044489.1, XM_018062151.1, XM_018064675.1, XM_005692406.3, XM_018056124.1, XM_018055908.1, XM_018047847.1, XM_018060052.1, XM_013972145.2, XM_005681111.3, XM_018057659.1, XR_001917888.1, XM_018065248.1, XM_018063793.1, XM_018062731.1, XM_018062409.1, XR_001917201.1, XM_018049834.1, XR_001295553.2, XM_018066691.1, XM_018040510.1, XM_005688283.3, XM_018048698.1, XM_018044319.1, XM_018052681.1, XM_018048786.1, XM_018062157.1, XR_001917194.1, XM_013973909.2, XM_018044321.1, XM_018066613.1, XM_018060567.1, XM_018058529.1, XM_005674982.3, XM_018040957.1, XR_001917202.1, XM_013969606.2, XM_018044609.1, XM_018039930.1, XM_005694793.3, XM_018041897.1, XM_018061043.1, XM_018066666.1, XR_001919786.1, XM_018038776.1, XM_018045689.1, XM_018065073.1, XM_018063637.1, XM_018045152.1, XM_018055297.1, XM_018055455.1, XR_001918906.1, XM_018065816.1, XM_018039224.1, XM_018055186.1, XM_018039287.1, XM_018054083.1, XM_018045336.1, XM_018053607.1, XM_018050706.1, XM_018066403.1, XM_018058106.1, XM_018039285.1, XM_018065065.1, XM_013975120.2, XM_018066695.1, XM_018058993.1, XM_018052287.1, XM_018060379.1, XM_005677146.3, XM_005681112.3, XM_013963900.1, XM_018046316.1, XM_005679294.1, XM_018064130.1, XM_018059696.1, XM_018066699.1, XM_005680373.3, XR_001917199.1, XM_018047976.1, XM_018048308.1, XM_018041968.1, XM_018052937.1, XM_018063304.1, XM_013970271.2, XM_018057689.1, XM_018039565.1, XM_018048693.1, XM_018048341.1, XM_018065070.1, XM_018059699.1, XM_018062272.1, XM_018043725.1, XM_018060042.1, NM_001285538.1, XM_005677613.3, XM_018065740.1, XM_005684675.3, XM_018048051.1, XR_001918098.1, XM_018066690.1, XM_018056230.1, XM_013968877.2, XM_018058699.1, XM_005692373.3, XM_005675332.3, XM_005698825.3, XR_001917308.1, XR_001917203.1, XM_005688564.3, XR_001296083.2, XM_018056089.1, XM_018060261.1, XM_018044328.1, XM_018045410.1, XM_018059000.1, XM_018062156.1, XM_018063859.1, XM_018065088.1, XM_018055677.1, XM_018040877.1, XM_018055991.1, XM_018063306.1, XM_018054733.1, XM_005682148.2, XM_005687163.3, XM_005695112.3, XM_018059764.1, XM_018066081.1, XM_018058690.1, XM_013964923.2, XM_018044500.1, XM_005693801.3, XM_018065932.1, XM_005689565.3, XM_018039931.1, XM_018059546.1, XM_005695610.2, XM_018055403.1, XM_018041835.1, XM_018063305.1, XM_018044333.1, XM_018048420.1, XM_018040721.1, XM_018063629.1, XM_018060377.1, XM_005697804.3, XM_018048050.1, XM_018040878.1, XM_005686167.2, XM_005675651.3, XM_018059763.1, XM_018040722.1, XM_018048918.1, XM_018053998.1, XM_018055995.1, XM_018064584.1, XM_018044317.1, XM_018043271.1, XM_018064678.1, NM_001285703.1, XM_018059760.1, XM_018066698.1, XM_018059548.1, NM_001285608.1, XM_018060927.1, XM_013965499.2, XM_018053047.1, XR_001917200.1, XM_018060818.1, XM_005679295.1, XM_018053846.1, XM_013972845.2, XM_018043211.1, XM_018064403.1, XM_018046816.1, XM_005697163.3, XM_018062404.1, XM_018047738.1, XM_018065250.1, XM_005700085.3, XM_018066696.1, XM_018056847.1, XM_018043209.1, XM_018053635.1, XM_018046317.1, XM_018039309.1, XM_005676807.3, XM_005688075.3, XR_001919375.1, XM_018055912.1, XM_018040960.1, XM_018052458.1, XM_018047444.1, XM_018057913.1, XM_018062248.1, XM_018055996.1, XM_018043664.1, XM_018055451.1, XM_005687533.3, XM_018059758.1, XM_018051703.1, XM_018044835.1, XM_018041045.1, XM_018050470.1, XM_005684036.3, XM_018060044.1, XM_018045638.1, XM_018065741.1, XM_018056659.1, XM_018040862.1, XM_018038231.1, XM_018057566.1, XM_005674769.3, XM_018067051.1, XM_018044824.1, XM_018041960.1, XM_018047424.1, XM_018053643.1, XR_310720.3, XM_018057840.1, XM_018063958.1, XM_018041715.1, XM_018048550.1, XM_018065918.1, XM_005676378.3, XM_018045151.1, XM_018062154.1, XM_018054172.1, XM_018039288.1, XM_018051443.1, XM_018048902.1, XM_018050763.1, XM_018048695.1, XM_005682215.2, XM_018045894.1, XM_018048563.1, XM_005688087.3, XM_018052683.1, XM_018055618.1, XM_018053231.1, XM_018062269.1, XM_018056226.1, XM_018041696.1, XM_018044821.1, XM_018043377.1, XM_018066693.1, XM_018048700.1, XR_001918907.1, XM_018043122.1, XM_018060728.1, XM_018049163.1, XM_018050765.1, XM_013976300.2, XM_018054469.1, NM_001285537.1, XM_018066697.1, XM_018056234.1, XM_018044822.1, XM_018063954.1, XM_018064394.1, XM_018047229.1, XM_018046775.1, XM_018043091.1, XM_018038614.1, XM_018057863.1, XM_005677612.3, XM_013974863.2, XM_013968497.2, XM_018053232.1 |
| 8 | [Adrenergic signaling in cardiomyocytes](../../../AppData/Local/Temp/Temp1_VETR-D-18-00038.zip/Cont-vs-PPRV_DEGseq_map/map04261.html) | XM_018062643.1, XM_013963001.2, XM_005681861.3, XR_001917331.1, XM_018061124.1, XM_018045824.1, XM_013964768.2, XM_018039549.1, XM_018052298.1, XM_005680366.2, XM_018063971.1, XM_018052301.1, XM_018042311.1, XM_018066062.1, XM_018063035.1, XM_018054045.1, XM_018055911.1, XM_018058967.1, XM_018055487.1, XM_005697610.3, XM_018055187.1, XM_005699866.3, XM_005697075.3, XM_005691447.3, XM_018062471.1, XM_018066370.1, XM_018051619.1, XM_018061256.1, XM_013972607.2, XM_018045956.1, XR_001917329.1, XM_005680364.3, XM_018050190.1, XR_001918973.1, XM_005699952.3, XM_018042310.1, XM_018048959.1, XM_018062407.1, XM_018043452.1, XM_018062408.1, XM_018058962.1, XM_018053228.1, XM_018042428.1, XM_018063293.1, XM_013974872.2, XM_005685955.3, XM_018061125.1, XM_018059802.1, XR_001917337.1, XM_005680365.2, XM_005691015.3, XM_018042308.1, XM_018047616.1, XM_018039561.1, XM_018060141.1, XM_018062410.1, XR_001917328.1, XM_018042061.1, XM_013968554.2, XM_018051622.1, XM_005688518.3, XM_018041016.1, XM_018058447.1, XM_005684782.3, XM_018059800.1, XM_005681863.3, XM_018047613.1, XM_013964782.2, XM_005675537.3, XM_018054206.1, XM_018043047.1, XM_018052297.1, XM_018049689.1, XM_018049709.1, XM_018045830.1, XM_013968553.2, XM_018048956.1, XM_018065817.1, XM_018043394.1, XM_005679333.3, XR_001917338.1, XM_013964769.2, XM_018042429.1, XM_018042307.1, XM_018064745.1, XM_018066371.1, XM_018049271.1, XM_018057813.1, XM_018052300.1, XM_018063036.1, XR_001917332.1, NM_001314202.1, XM_018055485.1, XM_018060488.1, XM_013967227.2, XM_018048952.1, NM_001285750.1, XM_013970799.2, XR_001917335.1, XM_018055908.1, XM_018049691.1, XM_018066065.1, XR_001297343.2, XM_018045201.1, XM_018043593.1, XM_018062409.1, XM_018059799.1, XR_001295516.2, XM_018058966.1, XM_018066068.1, XM_005685763.3, XM_005692304.2, XM_005680367.3, XM_018065816.1, XM_018055186.1, XM_018054083.1, XM_005676485.3, XM_005685150.3, XM_005686574.3, XM_013966779.2, XM_018042309.1, XM_018045834.1, XM_005677233.3, XM_018058968.1, XM_013970271.2, XM_013968877.2, XM_005675332.3, XM_018066067.1, XM_005681862.2, XM_005688564.3, XM_018060261.1, XM_018043454.1, XM_018048951.1, XM_005677232.3, XR_001917330.1, XR_001296033.2, XM_018055488.1, XM_013964774.2, XM_018058965.1, XM_018047617.1, XM_018064746.1, XM_018057613.1, XM_005683109.3, XM_018038791.1, XR_001917336.1, XM_018058970.1, XM_018062470.1, XM_018054044.1, XM_013964770.2, XM_005676482.3, XR_001297342.2, XM_018055912.1, XR_001917333.1, XM_018049270.1, XM_005685149.3, XM_018067051.1, XM_018042304.1, XM_018042305.1, XM_005697074.3, XM_018061406.1, XM_018064292.1, XM_018055486.1, XM_018066069.1, XM_018038788.1, XM_018053231.1, XM_018056518.1, XM_018042303.1, XM_005675538.3, XM_013976047.2, XR_001917334.1, XR_001917327.1, XM_005694349.2, XM_005699951.1, XM_018060142.1, XM_018053232.1 |
| 9 | [Non-small cell lung cancer](../../../AppData/Local/Temp/Temp1_VETR-D-18-00038.zip/Cont-vs-PPRV_DEGseq_map/map05223.html) | XM_018055645.1, XM_005681861.3, XM_018055614.1, XM_018064273.1, XM_013964768.2, XM_018056824.1, XM_005679127.2, XM_018063971.1, XM_018063035.1, XM_018055911.1, XM_005697610.3, XM_018055187.1, XM_018050987.1, XM_005685130.3, XM_018038275.1, XM_018060378.1, XM_018051619.1, XM_018060016.1, XM_005692453.3, XM_018048959.1, XM_018062407.1, XM_018062408.1, XM_018053228.1, XM_018042428.1, XM_018058171.1, XM_005681590.3, XM_013974872.2, XM_018047231.1, XM_018058500.1, XM_018056811.1, XM_018062410.1, XM_013968554.2, XM_018051622.1, XM_005689267.3, XM_018058447.1, XM_005684782.3, XM_018060376.1, XM_005681863.3, XM_005679129.3, XM_018064279.1, XM_018054206.1, XM_018038276.1, XM_018049689.1, XM_005700145.2, XM_013973685.2, XM_013968553.2, XM_005675289.3, XM_018048956.1, XM_018065817.1, XM_018059609.1, XM_013964769.2, XM_018042429.1, XM_018056690.1, XM_018064745.1, XM_018063036.1, NM_001314202.1, XM_018060488.1, XM_018049427.1, XM_013967227.2, XM_018048952.1, NM_001285750.1, XM_018055908.1, XM_018049691.1, XM_018062409.1, XM_005686996.3, XM_018050856.1, XM_018039227.1, XM_018039226.1, XM_018064276.1, XM_005685763.3, XM_018065816.1, XM_018055186.1, XM_018054083.1, XM_005683226.3, XM_018060379.1, XM_005680373.3, XM_018058634.1, XM_013970271.2, XM_018040360.1, XM_013968877.2, XM_005675332.3, XM_005685146.3, XM_013971256.2, XM_018064672.1, XM_005681862.2, XM_005688564.3, XM_018060261.1, XM_018058170.1, XM_018048951.1, XM_018058502.1, XM_018064671.1, XM_013964774.2, XM_018064746.1, XM_018048420.1, XM_018060377.1, XM_018038791.1, XM_005694472.3, XM_013964770.2, XM_005695770.3, XM_018055912.1, XM_018067051.1, XM_018064278.1, XM_018064292.1, XM_018064277.1, XM_018038788.1, XM_018053231.1, XM_018045593.1, XM_018064274.1, XM_005685145.3, XM_018053232.1 |
| 10 | [cGMP-PKG signaling pathway](../../../AppData/Local/Temp/Temp1_VETR-D-18-00038.zip/Cont-vs-PPRV_DEGseq_map/map04022.html) | XM_018062643.1, XM_013963001.2, XM_005681861.3, NM_001314231.1, XM_018061124.1, XM_013964768.2, XM_018065831.1, XM_018039549.1, XM_018052298.1, XM_005695088.3, XM_005680366.2, XM_018063971.1, XM_018052301.1, XM_018042311.1, XM_018055980.1, XM_018063035.1, XM_018054045.1, XM_018055911.1, XM_018055487.1, XM_018064437.1, XM_005697610.3, XM_018055187.1, XM_018041873.1, XR_001918100.1, XM_018050987.1, XM_005680737.3, XM_005691428.2, XM_005685130.3, XM_018054020.1, XM_005697075.3, XM_005691447.3, XM_018062471.1, XM_018066370.1, XM_018066401.1, XM_018051619.1, XM_018065829.1, XM_018061256.1, XM_018047856.1, XM_005683422.3, XM_013972607.2, XM_018045956.1, XM_018065827.1, XM_005680364.3, XM_013965777.2, XR_001918973.1, XM_005699952.3, XM_018042310.1, XM_018041323.1, XM_018048959.1, XM_018049972.1, XM_005683420.3, XM_018062407.1, XM_018062408.1, NM_001285674.1, XM_018053228.1, XM_013965781.2, XM_018042428.1, XM_018054634.1, XM_018062676.1, XM_005683419.3, XR_001919534.1, XM_018058066.1, XM_018063803.1, XM_018066400.1, XM_018063799.1, XM_018063293.1, XM_013974872.2, XM_018061125.1, XM_018055301.1, XM_018065834.1, XM_018054021.1, XM_018065832.1, XM_018064706.1, XM_005698163.3, XM_018049335.1, XM_018047050.1, XM_018051481.1, XM_005691313.3, XM_005680365.2, XM_018045751.1, XM_018042308.1, XM_018047616.1, XM_018039561.1, XM_018060141.1, XM_018063800.1, XM_005691312.2, XM_018061944.1, XM_018062410.1, XM_013968554.2, XM_018051622.1, XM_005688518.3, XM_018041016.1, XM_018058447.1, XM_005684782.3, XM_018061941.1, XM_018051432.1, XM_018044743.1, XM_005681863.3, XM_018047613.1, XM_018063801.1, XM_018054206.1, XM_018055439.1, XM_018043047.1, XM_018052297.1, XM_018054022.1, XM_018049689.1, XM_005683423.3, XM_013968553.2, XM_018048956.1, XM_018065817.1, XM_018043394.1, XM_005679333.3, XM_018063802.1, XM_018064436.1, XM_013964769.2, XM_018042429.1, XM_018042307.1, XM_018064745.1, XM_018066371.1, XM_018049271.1, XM_018057813.1, XM_018052300.1, XM_018064036.1, XM_018051427.1, XM_005675953.3, XM_018063036.1, XM_018041876.1, NM_001314202.1, XM_018055485.1, XM_018060488.1, XM_013967227.2, XM_018048952.1, NM_001285750.1, XM_018063797.1, XM_013970799.2, XM_018055908.1, XM_018046143.1, XM_018049691.1, XM_018064434.1, NM_001314204.1, XM_018045201.1, XM_005693569.2, XM_018043593.1, XM_018062409.1, XM_018064435.1, XR_001295516.2, XM_018065622.1, XM_018050856.1, XM_005691840.3, XM_005685763.3, XM_005694519.3, XM_005692304.2, XM_018061942.1, XM_005680367.3, XM_018061940.1, XM_018065816.1, XM_018055186.1, XM_018054083.1, XM_005676485.3, XM_005685150.3, XM_018055300.1, XM_018055663.1, XM_005686574.3, XM_013966779.2, XM_005681818.3, XM_018054635.1, XM_005678655.2, XM_018048449.1, XM_018042309.1, XM_005685503.3, XM_018050044.1, XM_018065835.1, XM_005677233.3, XM_018058573.1, XM_018065828.1, XM_018044096.1, XM_013970271.2, XR_001919533.1, XM_018065830.1, XR_001919417.1, XM_013968877.2, XM_005678171.3, XM_005675332.3, XM_005685146.3, XM_018064672.1, XM_005681862.2, XM_005688564.3, XM_018041327.1, XM_018060261.1, XM_018048951.1, XM_018061943.1, XM_005677232.3, XM_013965779.2, XM_018051409.1, XM_005681464.3, XR_001296033.2, XM_018064671.1, XM_018055488.1, XM_013964774.2, XM_018047617.1, XM_018064746.1, XR_001296712.2, XM_005691270.2, XM_018061790.1, XM_018055162.1, XM_018057613.1, XM_018055163.1, XM_018038791.1, XM_005694472.3, XM_018055302.1, XM_018062470.1, XM_018054044.1, XM_013964770.2, XM_005676482.3, XM_013965778.2, XM_018041140.1, XM_013971767.1, XM_005678170.3, XM_018041325.1, XM_018055912.1, XM_018049270.1, XM_005685149.3, XM_018041138.1, XM_018067051.1, XM_018042304.1, XM_005686948.3, XM_018042305.1, XM_005697074.3, XM_018064292.1, XM_018055486.1, XM_005688957.3, XM_005695085.3, XM_018038788.1, XM_005678654.3, XM_018053231.1, XM_018056518.1, XM_018065836.1, XM_018042303.1, XM_013976047.2, XM_018063798.1, XM_018041139.1, XM_018055664.1, XM_018055665.1, XM_018047883.1, XM_005685145.3, XM_005694349.2, XM_005699951.1, XM_018051417.1, XM_018060142.1, XM_018053232.1 |
| 11 | [Apelin signaling pathway](../../../AppData/Local/Temp/Temp1_VETR-D-18-00038.zip/Cont-vs-PPRV_DEGseq_map/map04371.html) | XM_005699593.3, XM_018044652.1, XM_018062643.1, XM_013973238.2, XM_018062483.1, XM_018058079.1, XM_013963001.2, XM_018043283.1, XM_005681861.3, XM_013970632.2, NM_001314231.1, XM_018062722.1, XM_018061124.1, XM_013964768.2, XM_018065831.1, XM_018039549.1, XM_018056451.1, XM_005679127.2, XM_005680366.2, XM_018063971.1, XM_018061046.1, NM_001285748.1, XR_001917497.1, XM_013970381.2, XM_018063035.1, XM_018054045.1, XM_018053461.1, XM_018055911.1, XM_018063078.1, XM_013971629.2, XM_018054628.1, XM_005697610.3, XM_005683110.3, XM_018047989.1, XM_018055187.1, XR_001918100.1, XM_018050987.1, XM_005680737.3, XM_018062714.1, XM_018059746.1, XM_018058560.1, XM_005685130.3, XM_018054020.1, XM_018040963.1, XM_005697075.3, XM_018060171.1, XM_005691447.3, XM_018066370.1, XM_018060378.1, XM_018051619.1, XM_013970631.2, XM_005680647.3, XM_018065829.1, XM_005683422.3, XM_018043280.1, XM_005686991.3, XM_013972607.2, XM_018065827.1, XM_005699594.3, XM_005678693.3, XM_005680364.3, XM_018060016.1, XM_013965777.2, XM_005699952.3, XM_018043282.1, XM_018048959.1, XM_018043281.1, XM_018049972.1, XM_018046692.1, XM_005683420.3, XM_018062407.1, XM_018043288.1, XM_018046693.1, XM_018065501.1, XM_018062408.1, XM_018062720.1, XM_018043792.1, XM_018053228.1, XM_013965781.2, XM_018042428.1, XM_018062676.1, XM_018043463.1, XM_005683419.3, XM_018043180.1, XM_018062590.1, XR_001919534.1, XM_018062484.1, XM_018063803.1, XM_018062713.1, XM_018063799.1, XM_013974872.2, XM_018062724.1, XM_018061125.1, XM_018047231.1, XM_018065834.1, XM_018053460.1, XM_018044746.1, XM_018044660.1, XM_018057740.1, XM_018054021.1, XM_018065832.1, XM_018051481.1, XM_018062721.1, XM_005680365.2, XM_018050610.1, XM_018057817.1, XM_018039561.1, XM_018060496.1, XR_001918229.1, XM_018063800.1, XR_001919054.1, XM_018062410.1, XM_018054208.1, XM_005675490.2, XM_013968554.2, XM_018051622.1, XM_005698535.3, XM_018052889.1, XM_005688518.3, XM_018041016.1, XM_018058447.1, XM_005684782.3, XM_018042871.1, XM_018060376.1, XM_018046688.1, XM_018057739.1, XM_005681863.3, XM_018063801.1, XM_018060738.1, XM_005679129.3, XM_018053462.1, XM_018054206.1, XM_018043047.1, XM_018054022.1, XM_005681337.3, XM_018049689.1, XM_018046689.1, XM_005683423.3, XM_005681336.3, XM_013968553.2, XM_005675289.3, XM_018048956.1, XM_018065817.1, XM_018049858.1, XM_005679333.3, XM_018063802.1, XM_018043794.1, XM_018043791.1, XM_018059609.1, XM_013962290.2, XM_013964769.2, NM_001285647.1, XM_018042429.1, XM_018064745.1, XM_018045601.1, XM_018046694.1, XM_018066371.1, XM_018057813.1, XM_005696969.3, XM_018063036.1, XM_018051501.1, NM_001314202.1, XM_018060488.1, XM_018060739.1, XM_013967227.2, XM_018059798.1, XM_018048952.1, NM_001285641.1, NM_001285750.1, XM_018063797.1, XM_013970799.2, XR_001917495.1, XM_018055908.1, XM_005701006.3, XM_018049691.1, XM_018048555.1, NM_001314204.1, XM_018043593.1, XM_018062409.1, XM_018053966.1, XM_013973240.2, XR_001295516.2, XM_018059919.1, XM_018050856.1, XM_018054332.1, XM_005702078.3, XM_018062482.1, XM_005685763.3, XM_018062710.1, XM_005693259.3, XM_018062725.1, XM_018046691.1, XM_013970533.2, XM_005690882.3, XM_005680367.3, XM_018065816.1, XM_018055186.1, XM_018054083.1, XM_005676485.3, XM_005685150.3, XM_005686574.3, XM_013966779.2, XM_018060379.1, XM_013962990.2, XM_018050044.1, XM_018059747.1, XM_018051760.1, XM_018065835.1, XM_018040964.1, XM_018054630.1, XR_001918230.1, XM_018065828.1, XM_013970271.2, XR_001919533.1, XM_018063749.1, XM_005693485.3, XM_018065830.1, XM_018066076.1, XM_013968877.2, XM_018066944.1, XM_005678171.3, XM_005675332.3, XM_005685146.3, XM_018064672.1, XM_005681862.2, XM_005688564.3, XM_018060261.1, XM_018062591.1, XM_018048951.1, XM_018049857.1, XR_001917180.1, XM_005695428.3, XM_013965779.2, XM_005693865.3, XM_018049721.1, XM_018053831.1, XR_001918314.1, XR_001296033.2, XM_018064671.1, XM_018059745.1, XM_013964774.2, XM_018064746.1, XM_013971630.2, XM_018059748.1, XM_018050609.1, XM_018062712.1, XR_001918228.1, XM_018048420.1, XM_018062718.1, XM_018060377.1, XM_018055162.1, XM_018057613.1, XM_018054629.1, XM_018046856.1, XM_018055163.1, XM_018038791.1, XM_018062723.1, XM_018043793.1, XM_005694472.3, XM_005695430.2, XM_018060089.1, XM_018062717.1, XM_018041744.1, XM_018054044.1, XM_018061071.1, XM_013965671.2, XM_018043284.1, XM_013964770.2, XM_005676482.3, XM_018047547.1, XM_013965778.2, XM_018046690.1, XM_018053830.1, XM_005678170.3, XM_018058102.1, XM_018055912.1, XM_018062711.1, XM_018043190.1, XM_005685149.3, XM_005695427.3, XM_005682905.3, XM_013974301.2, XM_018063972.1, XM_018067051.1, XM_018059918.1, XM_018065351.1, XM_018062719.1, XM_018066075.1, XM_005697074.3, XM_018048933.1, XM_018063570.1, XM_018064292.1, XM_005682904.3, XM_005695429.3, XM_013968876.2, XM_018038788.1, XM_018058103.1, XM_018053231.1, XM_018065836.1, XM_018043795.1, XM_018065502.1, XM_013976047.2, XM_005696968.3, XM_018063798.1, XM_005695431.3, XM_005685145.3, XM_005694349.2, XM_018043287.1, XM_005699951.1, XM_018055626.1, XR_001917496.1, XR_001918643.1, XM_013962471.2, XM_018053232.1 |
| 12 | [Renal cell carcinoma](../../../AppData/Local/Temp/Temp1_VETR-D-18-00038.zip/Cont-vs-PPRV_DEGseq_map/map05211.html) | XM_005681861.3, XM_018064273.1, XM_018061294.1, XM_013964768.2, XM_005698973.3, XM_018056824.1, XM_005679127.2, XM_018044120.1, XM_018062382.1, XM_018063971.1, XM_018061293.1, XM_018063035.1, XM_018044119.1, XM_018055911.1, XM_005676159.3, XM_005697610.3, XM_018055187.1, XM_018050987.1, XM_005685130.3, XM_018061289.1, XM_018054459.1, XM_018060378.1, XM_018051619.1, XM_018054460.1, XM_018044121.1, XM_018060016.1, XM_018043534.1, XM_018062448.1, XM_018061295.1, XM_018048959.1, XM_018062407.1, XM_018062408.1, XM_018053228.1, XM_018042428.1, XM_005681590.3, XM_018054461.1, XM_013974872.2, XM_018047231.1, XM_018042282.1, XM_005699064.3, XM_018062449.1, XM_018058500.1, XM_018043396.1, NM_001286443.1, XM_018061292.1, XM_005699065.3, XM_018056811.1, XM_018062410.1, XM_013968554.2, XM_018051622.1, XM_005700661.3, XM_018058447.1, XM_005684782.3, XM_018040436.1, XM_018060376.1, XM_018054462.1, XM_005681863.3, XM_005679129.3, XM_018064279.1, XM_018054206.1, XM_018042281.1, XM_005700662.3, XM_018049689.1, XM_005700145.2, XM_005691412.3, XM_013968553.2, XM_005675289.3, XM_018048956.1, XM_018065817.1, XM_018044122.1, XM_018059609.1, XM_013964769.2, XM_018042429.1, XM_018064745.1, XM_018061288.1, XM_018063036.1, NM_001314202.1, XM_018060488.1, XM_018049427.1, XM_018052324.1, XM_018042279.1, XM_018048812.1, XM_013967227.2, XM_018048952.1, NM_001285750.1, XM_005675491.2, XM_005681533.3, XM_018055908.1, XM_018049691.1, XM_005675493.3, XM_018062409.1, XM_018066613.1, XM_018050856.1, XM_018042278.1, XM_018064276.1, XM_005685763.3, XM_018046304.1, XM_018065816.1, XM_018055186.1, XM_018054083.1, XM_018042280.1, XM_013975781.2, XM_005683226.3, XM_018060379.1, XM_005680676.3, XM_005689072.3, XM_005680373.3, XM_013970271.2, XM_013968877.2, XM_005675332.3, XM_005685146.3, XM_018064672.1, XM_005681862.2, XM_005688564.3, XM_018060261.1, XR_001919756.1, XM_018048951.1, XM_018058502.1, XM_018064671.1, XM_018043395.1, XM_013964774.2, XM_018064746.1, XM_005695224.3, XM_018048420.1, XM_018061291.1, XM_018060377.1, XM_018066659.1, XM_018038791.1, XM_005694472.3, XM_018060277.1, XM_018065433.1, XM_018061286.1, XM_013964770.2, XM_018055912.1, NM_001285685.1, XM_018067051.1, XM_018064278.1, XM_005680013.2, XM_018038280.1, XM_018064292.1, XM_018064277.1, XM_018038788.1, XM_018053231.1, XM_018045593.1, XM_005685953.3, XM_018054431.1, XM_018064274.1, XM_005685145.3, XM_005699502.2, XM_018053232.1 |
| 13 | [Focal adhesion](../../../AppData/Local/Temp/Temp1_VETR-D-18-00038.zip/Cont-vs-PPRV_DEGseq_map/map04510.html) | XM_018063649.1, XM_005678936.3, XM_018038312.1, XM_018051874.1, XM_018059140.1, XM_005676250.3, XM_005681861.3, XM_018050499.1, XM_018059141.1, XM_018053479.1, XM_018043204.1, XM_018054194.1, XM_018064273.1, XM_018061294.1, XM_018052232.1, XM_018038313.1, XM_018052664.1, XM_013964768.2, XM_018065097.1, XM_013975959.2, XM_018045560.1, XM_013962777.2, XM_018056824.1, XM_018055113.1, XM_005695798.3, XM_005679127.2, XM_018052298.1, XM_018044120.1, XM_018058815.1, XM_018048123.1, XM_018040741.1, XM_018062382.1, XM_018065644.1, XM_005682490.2, XM_018066833.1, XM_018063971.1, XM_018052301.1, XM_018042311.1, XM_018061293.1, XM_018049302.1, XM_018054620.1, XM_018054014.1, XM_018063035.1, XM_018044119.1, XM_018055911.1, XM_018050764.1, XM_018047033.1, XM_018050759.1, XM_018055487.1, XM_018065643.1, XM_005676159.3, XM_013975225.2, XM_005697610.3, XM_018043415.1, XM_018055187.1, XM_018050987.1, XM_018049782.1, XM_018054452.1, XM_018047819.1, XM_005685130.3, XM_018058015.1, XM_018049780.1, XM_018059139.1, XR_001919753.1, XM_018049301.1, XM_005674830.2, XM_018061289.1, XM_005674883.3, XM_018066008.1, XM_018043411.1, XM_018054459.1, XM_018049781.1, XM_018039417.1, XM_005680115.3, XM_018060378.1, XM_018051619.1, XM_018056679.1, XM_018053480.1, XM_018055751.1, XM_018051017.1, XM_018044169.1, XM_005700226.2, XM_018061256.1, XM_018054460.1, XM_018053478.1, XM_018054454.1, XM_018044121.1, XM_018046953.1, XM_018051024.1, XM_018049795.1, XM_005693225.3, XM_018060016.1, XM_018039422.1, XR_001918973.1, XM_018043534.1, XM_018042310.1, XM_018038778.1, XM_018040641.1, XM_018066005.1, XM_018061295.1, XM_018049787.1, XM_018048959.1, XM_018062699.1, XM_018051032.1, XM_018040944.1, XM_018062407.1, XM_018049785.1, XM_018062408.1, XM_018039411.1, XM_018049788.1, XM_018048124.1, XM_018041048.1, XM_005681905.3, XM_005699260.3, XM_018053228.1, XM_018056363.1, XM_018042428.1, XM_018064893.1, XM_005681590.3, XM_005683247.3, XM_018040727.1, XM_018041576.1, XM_018066832.1, XM_018066842.1, XM_018042967.1, XM_005688053.3, XM_005695800.3, XM_018054461.1, XM_013974872.2, XM_018039401.1, XM_018051480.1, XM_018038561.1, XM_018047231.1, XM_018050567.1, XM_018052213.1, XM_018051350.1, XM_018045691.1, XM_018042282.1, XM_018058140.1, XM_018055040.1, XM_018044065.1, NM_001287573.1, XM_005682449.3, XM_005699064.3, XM_005693227.3, XM_018058500.1, XM_013965221.2, XM_018050266.1, XM_005709386.3, XM_013964307.2, XM_018043396.1, NM_001286443.1, XM_018061292.1, XM_005681906.3, XM_018038558.1, XM_018063551.1, XM_018040104.1, XM_005699065.3, XM_005695799.3, XM_018049300.1, XM_018047818.1, XM_018042308.1, XM_005680338.3, XM_018042470.1, XM_018040698.1, XM_018046955.1, XM_018039916.1, XM_018051042.1, XM_018056811.1, XM_018052661.1, XM_018062410.1, XM_013963034.2, XM_018058129.1, XM_005676883.3, XM_018063991.1, XM_013968554.2, XM_018051622.1, XM_005694016.2, XM_018063312.1, XM_005700661.3, XM_018058849.1, XM_018058447.1, XM_005684782.3, XM_018040436.1, XM_018060376.1, XM_005681564.3, XM_018060566.1, XM_018054462.1, XM_018043408.1, XM_005681863.3, XM_005679129.3, XM_018064279.1, XM_005682131.3, XM_005687578.3, XM_018054206.1, XM_018042281.1, XM_018052297.1, XM_005700662.3, XM_005675868.3, XM_018049689.1, XM_005700145.2, XM_018046554.1, XM_005675709.3, XM_005691412.3, XM_018057916.1, XM_013968553.2, XM_018066481.1, XM_005675289.3, XM_018048956.1, XM_018065817.1, XR_001919179.1, XM_018065642.1, XM_018051577.1, XM_018043394.1, XM_005694015.2, XM_018044122.1, XM_018066007.1, XM_018059609.1, XM_018047703.1, XM_018057521.1, XM_018052663.1, XM_018042674.1, XM_018052870.1, XM_013964769.2, XR_001295577.2, XM_018042429.1, XM_018066002.1, XM_018042307.1, XM_018061035.1, XM_005675869.3, XM_018053742.1, XM_018064745.1, XM_018052662.1, XM_018047345.1, XM_018054011.1, XM_018061288.1, XM_018052932.1, XM_018052867.1, XM_018049271.1, XM_018057501.1, XM_018045203.1, XM_018057246.1, XM_018052300.1, XM_018065480.1, XM_018063036.1, XM_018063966.1, XM_018040077.1, XM_018054749.1, XM_005681903.3, XM_018058855.1, NM_001314202.1, XM_018055485.1, XM_018058975.1, XM_018060488.1, XM_018049427.1, XM_018052324.1, XM_018042279.1, XM_013967227.2, XM_018048952.1, XM_018043409.1, NM_001285750.1, XM_018057881.1, XM_018058143.1, XM_018060831.1, XM_005681533.3, XM_018049881.1, XM_018055908.1, XM_018049691.1, XM_018045252.1, XM_005675149.3, XM_005689554.3, XM_018066004.1, XM_018062409.1, XM_005695795.3, XM_018050856.1, XM_005676887.3, XM_005674982.3, XM_018042278.1, XM_018038559.1, XM_013973596.2, XM_018064276.1, XM_005685763.3, XM_005693222.3, XM_018063471.1, XM_018054455.1, XM_018052201.1, XM_018042567.1, XM_018041569.1, XM_018046304.1, XM_005677636.3, XM_018058814.1, XM_005684278.2, XM_005690840.2, XM_005695796.3, XM_018048127.1, XM_018065816.1, XM_005700225.3, XM_018059142.1, XM_005693223.3, XM_018055186.1, XR_001918483.1, XM_018054083.1, XM_018042280.1, XM_013975781.2, XM_018045207.1, XM_005683226.3, XM_018054196.1, XM_018054617.1, XM_018058852.1, XM_018054420.1, XM_018053481.1, XM_018060379.1, XM_018044444.1, XM_018042468.1, XM_018042309.1, XM_018056198.1, XM_018058141.1, XM_005680676.3, XM_018066003.1, XM_005676249.3, XM_018048125.1, XM_018052331.1, XM_005680373.3, XM_018049783.1, XM_013974500.2, XM_018054013.1, XM_018058634.1, XM_018049880.1, XM_018038560.1, XM_013970271.2, XM_018054421.1, XM_005678307.2, XM_018049786.1, XM_018043407.1, XM_018065751.1, XM_018054012.1, XM_018040360.1, XM_018042467.1, XM_018045506.1, XM_013968877.2, XM_018052941.1, XM_005675332.3, XM_005685146.3, XM_018064672.1, XM_005681862.2, XM_005688564.3, XM_018059661.1, XM_013963945.2, XM_018060261.1, XM_018061577.1, XR_001919756.1, XM_018066053.1, XM_018046954.1, XM_018048951.1, XM_018063310.1, XM_018058502.1, XM_005678025.3, XM_018042966.1, XM_018066009.1, XM_013975194.2, XM_018066507.1, XM_018056364.1, XM_013975227.2, XM_013968280.2, XM_018040712.1, XM_018047031.1, XM_018044357.1, XM_018059663.1, XM_018042971.1, XM_018064671.1, XM_018043395.1, XM_018055488.1, XM_013964774.2, XM_018064746.1, XM_018044170.1, XM_018056863.1, XM_018048420.1, XM_018061291.1, XM_018047817.1, XM_018042327.1, XM_018060377.1, XM_018052826.1, XM_018057613.1, XM_018066659.1, XM_018038791.1, XM_018057493.1, XM_018047868.1, XM_005694472.3, XM_018042469.1, XM_018043357.1, XM_018066124.1, XM_018042471.1, XM_018061896.1, XM_005676715.3, XM_018065433.1, XR_001918577.1, XM_018066006.1, XM_018061286.1, XM_018051011.1, XM_018054453.1, XM_018050008.1, XM_013964770.2, XM_018049779.1, XM_018040719.1, XM_005694346.3, XM_005701390.3, XM_013962289.2, XM_005699727.3, XM_018051009.1, XM_018053042.1, XM_018059626.1, XM_018061369.1, XM_018055912.1, XM_018038562.1, XM_018065646.1, XM_018053473.1, XM_018049270.1, XM_018056543.1, XM_005682575.3, XM_005695797.3, XM_013972225.2, XM_018052339.1, XM_018045308.1, XM_013972226.2, XM_018039344.1, NM_001285685.1, XM_018064895.1, XM_018047816.1, XM_013966975.2, XM_018065939.1, XM_018060834.1, XM_018059143.1, XM_018067051.1, XM_018052868.1, XM_018058603.1, XM_018064278.1, XM_018042304.1, XM_018042305.1, XM_005680013.2, XM_005678961.3, XM_018043414.1, XM_018064292.1, XM_018041567.1, XM_018055486.1, XM_005681904.3, XM_018064277.1, XM_018050763.1, XM_018038788.1, XM_018055703.1, XM_018053231.1, XM_018044342.1, XM_018042303.1, XM_018045593.1, XM_018043410.1, XM_005685953.3, XM_018051010.1, XM_018054431.1, XM_018047091.1, XM_018043122.1, XM_018049784.1, XM_018050766.1, XM_018050765.1, XM_018064274.1, XM_018061570.1, XM_005685145.3, NM_001314200.1, XM_018042492.1, XM_005699502.2, XM_018063056.1, XM_018061557.1, XM_018043757.1, XM_005684543.2, XM_018040704.1, XM_018055450.1, XM_018053232.1 |
| 14 | [Dopaminergic synapse](../../../AppData/Local/Temp/Temp1_VETR-D-18-00038.zip/Cont-vs-PPRV_DEGseq_map/map04728.html) | XM_018062643.1, XM_013963001.2, XR_001917331.1, XM_018043204.1, XM_018061124.1, XM_018045824.1, XM_018039549.1, XM_018052298.1, XM_005680366.2, XM_018063971.1, XM_018052301.1, XM_018042311.1, XM_018066062.1, XM_018063035.1, XM_018054045.1, XM_018055911.1, XM_018058967.1, XM_018055487.1, XM_005697610.3, XM_018055187.1, XR_001918100.1, XM_005680737.3, XM_005699866.3, XM_005697075.3, XM_005691447.3, XM_018066370.1, XM_018061256.1, XM_013972607.2, XR_001917329.1, XM_005680364.3, XM_018050190.1, XR_001918973.1, XM_005699952.3, XM_018042310.1, XM_018062407.1, XM_018043452.1, XM_018062408.1, XM_018058962.1, XM_018053228.1, XM_013974872.2, XM_005685955.3, XM_018061125.1, XM_018059802.1, XM_018061348.1, XR_001917337.1, XM_005680365.2, XM_005691015.3, XM_018042308.1, XM_018039561.1, XM_018060496.1, XM_018062410.1, XM_018054208.1, XR_001917328.1, XM_018042061.1, XM_013968554.2, XM_005688518.3, XM_018041016.1, XM_018058447.1, XM_005684782.3, XM_018059800.1, XM_005687994.3, XM_013964782.2, XM_005675537.3, XM_018043047.1, XM_018052297.1, XM_018049709.1, XM_018045830.1, XM_013968553.2, XM_018065817.1, XM_018043394.1, XM_005679333.3, XR_001917338.1, XM_018048314.1, XM_018042307.1, XM_018066371.1, XM_018057171.1, XM_018049271.1, XM_018057813.1, XM_018052300.1, XM_018063036.1, XR_001917332.1, XM_018055485.1, XM_018060488.1, XM_018044693.1, XM_013967227.2, NM_001285750.1, XM_013970799.2, XR_001917335.1, XM_018055908.1, XM_018048555.1, XM_018066065.1, XR_001297343.2, XM_018043593.1, XM_018062409.1, XM_018059799.1, XR_001295516.2, XM_018058966.1, XM_005674982.3, XM_005680281.3, XM_018066068.1, XM_005680367.3, XM_018065816.1, XM_018055186.1, XM_018054083.1, XM_018061349.1, XM_005676485.3, XM_005685150.3, XM_005686574.3, XM_013966779.2, XM_018042309.1, XM_018045834.1, XM_018058968.1, XM_013970271.2, XM_013968877.2, XM_005675332.3, XM_018066067.1, XM_005688564.3, XM_018060261.1, XM_018043454.1, XR_001917330.1, XR_001296033.2, XM_018055488.1, XM_018058965.1, XM_018061347.1, XM_018057613.1, XM_005683109.3, XR_001917336.1, XM_018058970.1, XM_018062660.1, XM_018054044.1, XM_018048017.1, XM_005676482.3, XM_018047547.1, XR_001297342.2, XM_018055912.1, XR_001917333.1, XM_018049270.1, XM_005685149.3, XM_018061351.1, XM_018067051.1, XM_018042304.1, XM_018042305.1, XM_005697074.3, XM_018061406.1, XM_018055486.1, XM_018066069.1, XM_018053231.1, XM_018042303.1, XM_005675538.3, XM_013976047.2, XM_018043122.1, XR_001917334.1, XR_001917327.1, XM_005694349.2, XM_005699951.1, XM_018053232.1 |
| 15 | [Bile secretion](../../../AppData/Local/Temp/Temp1_VETR-D-18-00038.zip/Cont-vs-PPRV_DEGseq_map/map04976.html) | XM_018050968.1, XM_018042167.1, XM_018058923.1, XM_018050284.1, XM_013969141.2, XM_018050287.1, XM_005676051.2, XM_018047812.1, XM_018044689.1, XM_018046732.1, XM_018057415.1, XM_018056305.1, XM_005697024.2, XM_018062471.1, XM_005676007.3, XM_018045956.1, XM_018050965.1, XM_018050288.1, XM_018059677.1, XM_018047814.1, XM_018059814.1, XM_005686547.3, XM_018049142.1, XM_018063293.1, XM_018056290.1, XM_018065223.1, XR_001918298.1, XM_005680782.3, XM_018047749.1, XM_018050972.1, XM_013967600.2, XM_018050963.1, XM_005689540.3, XM_018064856.1, XM_018065222.1, XM_018056447.1, XM_013963209.2, XM_018047305.1, XM_018061401.1, XM_018056291.1, XM_005698320.3, XM_018049141.1, XM_018067221.1, XM_018054168.1, XM_005697025.3, XM_018045443.1, XM_018058927.1, XM_018050967.1, XM_018046517.1, XM_018046614.1, XM_018063754.1, NM_001285578.1, XM_005681740.3, XM_013964709.2, XM_005697026.3, XM_018067218.1, XM_018064857.1, XM_018049138.1, XM_005680780.3, XM_005680781.3, XM_018061402.1, XM_018049137.1, XR_001918297.1, XM_018046617.1, XM_018063266.1, XM_018047277.1, XM_005692304.2, XM_013967599.2, XM_013969130.2, XM_018046615.1, XM_013969124.2, XM_018063180.1, XM_018047300.1, XM_018049558.1, XM_018045598.1, XM_018056446.1, XM_005677233.3, XM_005676049.3, XM_018067222.1, XM_018050961.1, XM_018050966.1, XM_018056443.1, XM_018050969.1, XM_018056304.1, XM_018050962.1, XM_018061403.1, XM_018047299.1, XM_005677232.3, XM_018056286.1, XM_018050964.1, XM_018050283.1, XM_005691954.3, XM_018063738.1, XM_018063767.1, XM_005681792.3, XM_018058920.1, XM_018062470.1, XM_018045615.1, XM_018046613.1, XM_018059676.1, NM_001285707.1, XM_013969127.2, XM_005676008.3, XM_018056323.1, XM_018046731.1, XM_018056518.1, XM_013969135.2, XM_005679028.2, XM_018049140.1, XM_018053229.1, XM_018053178.1, XM_018050971.1, XM_018049143.1, XM_013963212.2, XM_018053703.1, XM_018046616.1 |
| 16 | [Chronic myeloid leukemia](../../../AppData/Local/Temp/Temp1_VETR-D-18-00038.zip/Cont-vs-PPRV_DEGseq_map/map05220.html) | XM_018055645.1, XM_005681861.3, XM_018055614.1, XM_018064273.1, XM_013964768.2, XM_018056824.1, XM_005679127.2, XM_018063971.1, XM_018063035.1, XM_018055911.1, XM_005697610.3, XM_018044427.1, XM_018055187.1, XM_018050987.1, XM_005685130.3, XM_018060378.1, XM_018051619.1, XM_018060016.1, XM_005692453.3, XM_018048959.1, XM_018062407.1, XM_018062408.1, XM_018053228.1, XM_018042428.1, XM_018058171.1, XM_005681590.3, XM_013974872.2, XM_018047231.1, XM_018058500.1, XM_018056811.1, XM_018062410.1, XM_005683062.3, XM_013968554.2, XM_018051622.1, XM_005689267.3, XM_018058447.1, XM_005684782.3, XM_018060376.1, XM_005681863.3, XM_005679129.3, XM_018064279.1, XM_018054206.1, XM_018049689.1, XM_005700145.2, XM_013968553.2, XM_005675289.3, XM_018048956.1, XM_018065817.1, XM_018059609.1, XM_018057759.1, XM_013964769.2, XM_018042429.1, XM_018056690.1, XM_018064745.1, XM_018063036.1, NM_001314202.1, XM_018060488.1, XM_018049427.1, XM_013967227.2, XM_018048952.1, NM_001285750.1, XM_018055908.1, XM_018049691.1, XM_018062409.1, XM_005686996.3, XM_018050856.1, XM_018039227.1, XM_018039226.1, XM_018064276.1, XM_005685763.3, XM_018065816.1, XM_018055186.1, XM_018054083.1, XM_005683226.3, XM_018060379.1, XM_005680373.3, XM_013970271.2, XM_013968877.2, XM_005675332.3, XM_005685146.3, XM_013971256.2, XM_018064672.1, XM_005681862.2, XM_005688564.3, XM_018060261.1, XM_018058170.1, XM_018048951.1, XM_018058502.1, XM_018064671.1, XM_013964774.2, XM_018064746.1, XM_018048420.1, XM_018060377.1, XM_018038791.1, XM_005694472.3, XM_013964770.2, XM_018055912.1, XM_018067051.1, XM_018064278.1, XM_018064292.1, XM_018064277.1, XM_018038788.1, XM_018053231.1, XM_018045593.1, XM_018064274.1, XM_005685145.3, XM_018053232.1 |
| 17 | [Insulin signaling pathway](../../../AppData/Local/Temp/Temp1_VETR-D-18-00038.zip/Cont-vs-PPRV_DEGseq_map/map04910.html) | XM_005699593.3, XM_018044652.1, XM_018062643.1, XM_013973238.2, XM_018062483.1, XM_018058079.1, XM_013963001.2, XM_018043283.1, XM_005681861.3, XM_013970632.2, XM_018064326.1, XM_018043204.1, XM_018064273.1, NM_001285629.1, XM_018061124.1, XM_013964768.2, XM_013962541.2, XM_018056451.1, XM_018056824.1, XM_005679127.2, XM_018052298.1, XM_005680366.2, XM_018063971.1, XM_018052301.1, XM_018061046.1, XM_018058022.1, XM_018042311.1, NM_001285748.1, XR_001917497.1, XM_013970381.2, XM_018066935.1, XM_018063035.1, NM_001285656.1, XM_018054045.1, XM_018053461.1, XM_018055911.1, XM_018063078.1, XM_018055487.1, XM_013971629.2, XM_018043567.1, XM_018054628.1, XM_005697610.3, XM_005683110.3, XM_018047989.1, XM_018055187.1, XM_018050987.1, XM_018059746.1, XM_018058560.1, XM_005685130.3, XM_018040963.1, XM_005697075.3, XM_018060171.1, XM_005691447.3, XM_018066370.1, XM_018060378.1, XM_018051619.1, XM_013970631.2, XM_005680647.3, XM_005680005.2, XM_018061256.1, XM_018043280.1, XM_005686991.3, XM_018064170.1, XM_013972607.2, XM_005680926.2, XM_005699594.3, XM_018063633.1, XM_005678693.3, XM_005680364.3, XM_018060016.1, XM_018057287.1, XR_001918973.1, XM_005699952.3, XM_018043282.1, XM_018042310.1, XM_018048959.1, XM_018043281.1, XM_018046692.1, XM_018062407.1, XM_018043288.1, XM_018046693.1, XM_018065501.1, XM_018062408.1, XM_018043792.1, XM_018053228.1, XM_018048437.1, XM_018042428.1, XM_005681590.3, XM_018043463.1, XM_018043180.1, XM_018062590.1, XM_018062484.1, XR_001919014.1, XM_013974872.2, XM_013962523.2, XM_018061125.1, XM_018047231.1, XM_018042596.1, XM_018042597.1, XM_018053460.1, XM_018044746.1, XM_018049295.1, XM_005693625.3, XM_018064706.1, XM_018058500.1, XM_018042326.1, XM_005680365.2, XM_018050610.1, XM_018042308.1, XM_018057817.1, XM_018039561.1, XR_001918229.1, XM_005695728.3, XM_018056811.1, XR_001919054.1, XM_018062410.1, XM_013976723.2, XM_005675490.2, XM_013968554.2, XM_018051622.1, XM_005698535.3, XM_005688518.3, XM_018041016.1, XM_018058447.1, XM_005684782.3, XM_005698194.3, XM_005676676.2, XM_018042871.1, XM_018060376.1, XM_018046688.1, XM_005681863.3, XM_018060738.1, XM_005679129.3, XM_018064279.1, XM_018053462.1, XM_018054206.1, XM_018043047.1, XM_018052297.1, XM_005681337.3, XM_018049689.1, XM_005700145.2, XM_018046689.1, XM_005681336.3, XM_013968553.2, XM_005675289.3, XM_018048956.1, XM_018052574.1, XM_018065817.1, XM_018064174.1, XM_018063764.1, XM_018043394.1, XM_018049858.1, XM_005679333.3, XM_018043794.1, XM_018043791.1, XM_018059609.1, XM_013962290.2, XM_013964769.2, NM_001285647.1, XM_018042429.1, XM_018042307.1, XM_018064745.1, XM_018043568.1, XM_018063760.1, XM_018046694.1, XM_018066371.1, XM_018049271.1, XM_018054571.1, XM_018064220.1, XM_018057813.1, XM_018052300.1, XM_005675953.3, XM_018053881.1, XM_018063036.1, XM_018039451.1, XM_005676678.3, XM_018051666.1, XM_018051501.1, NM_001314202.1, XM_018055485.1, XM_018060488.1, XM_018049427.1, XM_018060739.1, XM_013967227.2, XM_018059798.1, XM_018048952.1, NM_001285641.1, NM_001285750.1, XM_013970799.2, XM_018061156.1, XR_001917495.1, XM_018055908.1, XM_005701006.3, XM_018049691.1, XM_018054976.1, XM_005684284.3, XM_018043593.1, XM_018062409.1, XM_018053966.1, XM_018047882.1, XM_013973240.2, XR_001295516.2, XM_018059919.1, XM_018050856.1, XM_018054332.1, XM_005674982.3, XM_005702078.3, XM_018062482.1, XM_018064276.1, XM_005685763.3, XM_005693259.3, XM_018046691.1, XM_013970533.2, XM_005690882.3, XM_005680367.3, XM_018054351.1, XM_018065816.1, XM_018055186.1, XM_018054083.1, XM_005676485.3, XM_018048543.1, XM_018060734.1, XM_005685150.3, XM_018060733.1, XM_005683226.3, XM_005686574.3, XM_013966779.2, XM_018060379.1, XM_018064169.1, XM_005699864.3, XM_018042309.1, XM_013962990.2, XM_005684287.3, XM_005680373.3, XM_018059747.1, XM_018051760.1, XM_018058634.1, XM_018040964.1, XM_018054630.1, XR_001918230.1, XM_013970271.2, XM_018038249.1, XM_018040360.1, XM_005676677.3, XM_018064325.1, XM_018066076.1, XM_013968877.2, XM_018064172.1, XM_018066944.1, XM_005678171.3, XM_005675332.3, XM_005685146.3, XM_018064672.1, XM_005681862.2, XM_005688564.3, XM_018060261.1, XM_018060862.1, XM_018062591.1, XM_018048951.1, XM_018049857.1, XM_018058502.1, XM_018047230.1, XM_005695428.3, XM_018049721.1, XM_018053831.1, XR_001918314.1, NM_001285594.1, XR_001296033.2, XM_018064671.1, XM_018059745.1, XM_018055488.1, XM_013964774.2, XM_018064746.1, XM_013971630.2, XM_018064173.1, XM_018059748.1, XM_018050609.1, NM_001287233.1, XR_001918228.1, XM_018048420.1, XM_018060377.1, XM_018047462.1, XM_018054629.1, XM_018038791.1, XM_018043793.1, XM_005694472.3, XM_005695430.2, XM_018060089.1, XM_018061094.1, XM_018041744.1, XM_018054044.1, XM_018044227.1, XM_018061071.1, XM_013965671.2, XM_018064168.1, XM_018043284.1, XM_013964770.2, XM_005676482.3, XM_005684192.3, XM_018050781.1, XM_018046690.1, XM_018053830.1, XM_005684137.3, XM_005678170.3, XM_018053882.1, XM_018058102.1, XM_018055912.1, XM_018049270.1, XM_018043190.1, XM_005685149.3, XM_005695427.3, XM_005682905.3, XM_018063972.1, XM_018067051.1, XM_018059918.1, XM_005684138.3, XM_018064278.1, XM_018042304.1, XM_018066075.1, XM_018042305.1, XM_005697074.3, XM_005691714.3, XM_005684283.3, XM_018048933.1, XM_013968393.2, XM_018063570.1, XM_018064292.1, XM_005682904.3, XM_018055486.1, XM_005695429.3, XM_018064277.1, XM_013968876.2, XM_018038788.1, XM_018058103.1, XM_018058021.1, XM_018053231.1, XM_018047553.1, XM_018042303.1, XM_018045593.1, XM_018043795.1, XM_018065502.1, XM_013976047.2, XM_018050632.1, XM_005695431.3, XM_005675512.3, XM_018043122.1, XM_018064274.1, XM_018047883.1, XM_005685145.3, XM_018065973.1, XM_005694349.2, XM_018043287.1, XM_005699951.1, XM_018055626.1, XM_018048748.1, XR_001917496.1, XR_001918643.1, XM_013962471.2, XM_018064171.1, XM_018053232.1 |
| 18 | [Hepatitis C](../../../AppData/Local/Temp/Temp1_VETR-D-18-00038.zip/Cont-vs-PPRV_DEGseq_map/map05160.html) | XM_005681861.3, XR_001917331.1, XM_018043204.1, XM_018064273.1, XM_013964768.2, XM_018056824.1, XM_005679127.2, XM_018063971.1, XM_018063035.1, XM_018055911.1, XM_005697610.3, XM_018055187.1, XR_311047.3, XM_018060378.1, XM_018051619.1, XM_018054468.1, XM_018055124.1, XR_001917329.1, XM_018060016.1, XM_018050190.1, XM_018048959.1, XM_018062407.1, XM_018062408.1, XM_018053228.1, XM_018042428.1, XM_005681590.3, XM_005685995.3, XM_013974872.2, XM_018059802.1, XM_018047231.1, XM_018053601.1, XM_018058500.1, XR_001917337.1, XM_018056811.1, XM_013963209.2, XM_018062410.1, XR_001917328.1, XM_018040509.1, XM_018061401.1, XM_018042061.1, XM_013968554.2, XM_018051622.1, XM_018058447.1, XM_005684782.3, XM_018059800.1, XM_018060376.1, XM_005681863.3, XM_013964782.2, XM_005679129.3, XM_018064279.1, XM_018054206.1, XM_018049689.1, XM_018049709.1, XM_005700145.2, XM_013968553.2, XM_005675289.3, XM_018048956.1, XM_018065817.1, XM_018046614.1, XM_018059609.1, XR_001917338.1, XM_013964769.2, XM_018042429.1, NM_001285578.1, XM_018064745.1, XM_018063036.1, XR_001917332.1, NM_001314202.1, XM_018060488.1, XM_018049427.1, XM_013967227.2, XM_018048952.1, NM_001285750.1, XM_018061402.1, XM_018055349.1, XR_001917335.1, XM_018055908.1, XM_018049691.1, XR_001297343.2, XM_018062409.1, XM_018059799.1, XM_018040510.1, XM_018046617.1, XM_005674982.3, XM_018047277.1, XM_018064276.1, XM_005685763.3, XM_018065816.1, XM_018055186.1, XM_018054083.1, XM_005683226.3, XM_018060379.1, XM_018046615.1, XM_005679294.1, XM_005680373.3, XM_018058634.1, XM_013970271.2, XM_018040360.1, XM_013968877.2, XM_005675332.3, XM_005681862.2, XM_005688564.3, XM_018060261.1, XM_018061403.1, XM_018048951.1, XM_018058502.1, XR_001917330.1, XM_013964774.2, XM_018064746.1, XM_018048420.1, XM_018060377.1, XM_005681792.3, XM_005683109.3, XM_018038791.1, XR_001917336.1, XM_013964770.2, XM_005679295.1, XR_001297342.2, XM_018055912.1, XR_001917333.1, XM_018046613.1, XM_018067051.1, XM_018064278.1, XM_018061406.1, XM_018064292.1, XM_018064277.1, XM_018038788.1, XM_018053231.1, XM_018045593.1, XM_018043122.1, XR_001917334.1, XM_018064274.1, XR_001917327.1, XM_018054469.1, XM_013963212.2, XM_018046616.1, XM_018053232.1 |
| 19 | [Pancreatic cancer](../../../AppData/Local/Temp/Temp1_VETR-D-18-00038.zip/Cont-vs-PPRV_DEGseq_map/map05212.html) | XM_018055645.1, XM_005681861.3, XM_018055614.1, XM_018064273.1, XM_018061294.1, XM_013964768.2, XM_018056824.1, XM_005679127.2, XM_018063971.1, XM_018061293.1, XM_018063035.1, XM_018055911.1, XM_005676159.3, XM_005697610.3, XM_018055187.1, XM_018050987.1, XM_005685130.3, XM_018061289.1, XM_018060378.1, XM_018051619.1, XM_018060016.1, XM_005692453.3, XM_018043534.1, XM_018061295.1, XM_018048959.1, XM_018062407.1, XM_018062408.1, XM_018053228.1, XM_018042428.1, XM_018058171.1, XM_005681590.3, XM_013974872.2, XM_018047231.1, XM_018042282.1, XM_005699064.3, XM_018058500.1, NM_001286443.1, XM_018061292.1, XM_005699065.3, XM_018056811.1, XM_018062410.1, XM_013968554.2, XM_018051622.1, XM_005700661.3, XM_005689267.3, XM_018058447.1, XM_005684782.3, XM_018040436.1, XM_018060376.1, XM_005681863.3, XM_005679129.3, XM_018064279.1, XM_018054206.1, XM_018042281.1, XM_005700662.3, XM_018049689.1, XM_005700145.2, XM_005691412.3, XM_013968553.2, XM_005675289.3, XM_018048956.1, XM_018065817.1, XM_018059609.1, XM_013964769.2, XM_018042429.1, XM_018056690.1, XM_018064745.1, XM_018061288.1, XM_018063036.1, NM_001314202.1, XM_018060488.1, XM_018049427.1, XM_018052324.1, XM_018042279.1, XM_013967227.2, XM_018048952.1, NM_001285750.1, XM_005681533.3, XM_018055908.1, XM_018049691.1, XM_018062409.1, XM_005686996.3, XM_018050856.1, XM_018039227.1, XM_018042278.1, XM_018039226.1, XM_018064276.1, XM_005685763.3, XM_018065816.1, XM_018055186.1, XM_018054083.1, XM_018042280.1, XM_005683226.3, XM_018060379.1, XM_005680676.3, XM_005680373.3, XM_013970271.2, XM_013968877.2, XM_005675332.3, XM_005685146.3, XM_013971256.2, XM_018064672.1, XM_005681862.2, XM_005688564.3, XM_018060261.1, XR_001919756.1, XM_018058170.1, XM_018048951.1, XM_018058502.1, XM_018064671.1, XM_013964774.2, XM_018064746.1, XM_018048420.1, XM_018061291.1, XM_018060377.1, XM_018038791.1, XM_005694472.3, XM_018065433.1, XM_018061286.1, XM_013964770.2, XM_018056629.1, XM_018055912.1, XM_018056628.1, NM_001285685.1, XM_018067051.1, XM_018064278.1, XM_005680013.2, XM_018064292.1, XM_018064277.1, XM_018038788.1, XM_018053231.1, XM_018045593.1, XM_005685953.3, XM_018054431.1, XM_018064274.1, XM_005685145.3, XM_018053232.1 |
| 20 | [Proteoglycans in cancer](../../../AppData/Local/Temp/Temp1_VETR-D-18-00038.zip/Cont-vs-PPRV_DEGseq_map/map05205.html) | XM_005681861.3, XM_005677656.3, NM_001314266.1, XM_018064273.1, XM_018061294.1, XM_018045824.1, XM_013964768.2, XM_018065097.1, XM_018056451.1, XM_018056824.1, XM_005695798.3, XM_005679127.2, XM_018052298.1, XM_018044120.1, XM_018062382.1, XM_005682490.2, XM_018063971.1, XM_018052301.1, XM_018042311.1, NM_001285748.1, XM_018061293.1, XM_013970381.2, XM_018049302.1, XM_018063035.1, XM_018044119.1, XM_018053461.1, XM_018055911.1, XM_018058967.1, XM_018055487.1, XM_005676159.3, XM_013975225.2, XM_005697610.3, XM_005683110.3, XM_018047989.1, XM_018055187.1, XR_001918100.1, XM_018050987.1, XM_005680737.3, XM_018049782.1, XM_005685130.3, XM_018049780.1, XM_018049301.1, XM_018061289.1, XM_018054459.1, XM_018049781.1, XM_018060378.1, XM_018051619.1, XM_018061256.1, XM_018054460.1, XM_018044121.1, XM_018046953.1, XM_018060016.1, XR_001918973.1, XM_018043534.1, XM_018042310.1, XM_018051898.1, XM_018040641.1, XM_018061295.1, XM_018049787.1, XM_018048959.1, XM_018062407.1, XM_018049785.1, XM_018062408.1, XM_018058962.1, XM_018049788.1, XM_018043792.1, XM_018053228.1, XM_018042428.1, XM_005681590.3, XM_018043463.1, XM_005695800.3, XM_018054461.1, XM_013974872.2, XM_018045525.1, XM_018047231.1, XM_018053460.1, XM_018042282.1, XM_005699064.3, XM_018051782.1, XM_018058500.1, XM_018043396.1, NM_001286443.1, XM_018061292.1, XM_005699065.3, XM_018041937.1, XM_005695799.3, XM_018050610.1, XM_018049300.1, XM_018042308.1, XM_018046955.1, XR_001918229.1, XM_018056811.1, XR_001919054.1, XM_018062410.1, XM_005675490.2, XM_013968554.2, XM_018051622.1, XM_005694016.2, XM_005698535.3, XM_005700661.3, XM_018048766.1, XM_018058447.1, XM_005684782.3, XM_018040436.1, XM_018042871.1, XM_018060376.1, XM_018054462.1, XM_005681863.3, XM_018060738.1, XM_005679129.3, XM_018064279.1, XM_018053462.1, XM_018054206.1, XM_018042281.1, XM_018052297.1, XM_005700662.3, XM_018049689.1, XM_005700145.2, XM_018045830.1, XM_005691412.3, XM_013968553.2, XM_005675289.3, XM_018048956.1, XM_018065817.1, XM_018043394.1, XM_018049858.1, XM_005694015.2, XM_018044122.1, XM_018043794.1, XM_018043791.1, XM_018059609.1, XM_013964769.2, XR_001295577.2, XM_018042429.1, XM_018042307.1, XM_018064745.1, XM_018061288.1, XM_018049271.1, XM_018052300.1, XM_018051785.1, XM_018048765.1, XM_018063036.1, NM_001314202.1, XM_018055485.1, XM_018060488.1, XM_018049427.1, XM_018060739.1, XM_018052324.1, XM_018042279.1, XM_013967227.2, XM_018048952.1, NM_001285641.1, NM_001285750.1, XM_005681533.3, XM_018055908.1, XM_005701006.3, XM_018049691.1, XM_018051784.1, XM_005698313.2, XM_018062409.1, XM_018053966.1, XM_018059919.1, XM_018058966.1, XM_005695795.3, XM_018050856.1, XM_018042278.1, XM_018064276.1, XM_005685763.3, XM_005693259.3, XM_018046304.1, XM_005684278.2, XM_005695796.3, XM_018065816.1, XM_018055186.1, XM_018054083.1, XM_018042280.1, XM_013975781.2, XM_005683226.3, XM_018060379.1, XM_018042309.1, XM_005680676.3, XM_018045834.1, XM_005680373.3, XM_018049783.1, XM_018058634.1, XM_018047709.1, XR_001918230.1, XM_018058968.1, XM_013970271.2, XM_018047710.1, XM_018049786.1, XM_018041278.1, XM_018040360.1, XM_013968877.2, XM_005678171.3, XM_005675332.3, XM_005685146.3, XM_018064672.1, XM_005681862.2, XM_005688564.3, XM_018060261.1, XR_001919756.1, XM_018046954.1, XM_018048951.1, XM_018049857.1, XM_018058502.1, XM_018049721.1, XM_013975227.2, XM_018053831.1, XM_018064671.1, XM_018043395.1, XM_018055488.1, XM_013964774.2, XM_018058965.1, XM_018064746.1, XM_018050609.1, XR_001918228.1, XM_018048420.1, XM_018061291.1, XM_018060377.1, XM_018041467.1, XM_018066659.1, XM_018038791.1, XM_018043793.1, XM_018058970.1, XM_005694472.3, XM_018060089.1, XM_005676715.3, XM_018065433.1, XM_018061286.1, XM_018061071.1, XM_013965671.2, XM_013964770.2, XM_018049779.1, XM_018051783.1, XM_018053830.1, XM_005678170.3, XM_018055912.1, XM_018051781.1, XM_018049270.1, XM_005695797.3, XM_013972225.2, XM_013972226.2, NM_001285685.1, XM_018063972.1, XM_018067051.1, XM_018059918.1, XM_018064278.1, XM_018042304.1, XM_018042305.1, XM_013971274.2, XM_005680013.2, XM_018063570.1, XM_018064292.1, XM_018055486.1, XM_018064277.1, XM_013968876.2, XM_018038788.1, XM_018053231.1, XM_018042303.1, XM_018045593.1, XM_018043795.1, XM_005685953.3, XM_018054431.1, XM_018049784.1, XM_018064274.1, XM_005685145.3, XM_005699502.2, XM_018065360.1, XR_001918643.1, XM_018053232.1 |
| 21 | [Olfactory transduction](../../../AppData/Local/Temp/Temp1_VETR-D-18-00038.zip/Cont-vs-PPRV_DEGseq_map/map04740.html) | XM_018062643.1, XM_005684795.2, XM_013963001.2, XM_018061124.1, XM_018045824.1, XM_005680366.2, XM_018054045.1, XM_018058967.1, XM_018053453.1, XM_018054020.1, XM_005697075.3, XM_018058454.1, XM_005691447.3, XM_018066370.1, XM_005682114.3, XM_013972607.2, XM_018051048.1, XM_005680364.3, XM_005684794.3, XM_005694678.3, XM_005699952.3, XM_013972972.2, XM_018058962.1, XM_018062676.1, XR_001919534.1, XM_018063803.1, XM_013965894.2, XM_018063799.1, XM_018061125.1, XM_018054021.1, XM_018064706.1, XM_005698163.3, XM_013965892.2, XM_018051915.1, XM_005680365.2, XM_018039561.1, XM_018060496.1, XM_005694677.3, XM_018063800.1, XM_018054208.1, XM_018065511.1, XM_005688518.3, XM_018041016.1, XM_005694675.3, XM_018063801.1, XM_018045231.1, XM_018043047.1, XM_018054022.1, XM_018051049.1, XM_018045830.1, XM_005679333.3, XM_018066184.1, XM_018063802.1, XM_018066186.1, XM_005695333.3, XM_018066371.1, XM_018051046.1, XM_018057813.1, XM_005675953.3, XM_018063797.1, XM_013970799.2, XM_018047636.1, XM_018051050.1, XM_018048555.1, XM_018043593.1, XM_013965891.2, XR_001295516.2, XM_018058966.1, XM_018065509.1, XM_005680367.3, XM_005676485.3, XM_005685150.3, XM_013964453.2, XM_005686574.3, XM_013966779.2, XM_005681818.3, XM_005684793.3, XM_018045834.1, XM_018050044.1, XM_005682112.2, XM_018058968.1, XR_001919533.1, XM_005678171.3, XM_013966645.2, XM_013963332.2, XM_018065510.1, XR_001296033.2, XM_018058965.1, XM_018055162.1, XM_018055163.1, XM_018058970.1, XM_018054044.1, XM_005676482.3, XM_018047547.1, XM_018041140.1, XM_005678170.3, XM_005685149.3, XM_018041138.1, XM_018045239.1, XM_018058455.1, XM_005697074.3, XM_018066185.1, XM_018047638.1, XM_018053454.1, XM_005675916.3, XM_005675891.3, XM_005695332.3, XM_013976047.2, XM_005685809.3, XM_018063798.1, XM_018041139.1, XM_018047883.1, XM_005694349.2, XM_005699951.1, XM_018047637.1, XM_005678304.3 |
| 22 | [T cell receptor signaling pathway](../../../AppData/Local/Temp/Temp1_VETR-D-18-00038.zip/Cont-vs-PPRV_DEGseq_map/map04660.html) | XM_005681861.3, XM_018043204.1, XM_013964768.2, XM_005679127.2, XM_018052298.1, XM_018044120.1, XM_005695088.3, XM_018062382.1, XM_018063971.1, XM_018052301.1, XM_018042311.1, XM_018038998.1, XM_018055980.1, XM_018063035.1, XM_018044119.1, XM_018055911.1, XM_005697610.3, XM_018055187.1, XM_018050987.1, XM_005685130.3, XM_018054459.1, XM_018060378.1, XM_018066401.1, XM_018051619.1, XM_018054460.1, XM_018044121.1, XM_018060016.1, XM_018042310.1, XM_018041323.1, XM_018048959.1, XM_018060045.1, XM_018062407.1, XM_018062408.1, XM_005679901.2, XM_018053228.1, XM_018042428.1, XM_018058066.1, XM_018066400.1, XM_018054461.1, XM_013974872.2, XM_018047231.1, XM_018049335.1, XM_018043396.1, XM_018042308.1, XM_018062410.1, XM_013968554.2, XM_018051622.1, XM_018058447.1, XM_005684782.3, XM_018060376.1, XM_018054462.1, XM_005681863.3, XM_005679129.3, XM_018054206.1, XM_018055439.1, XM_018052297.1, XM_018049689.1, XM_013968553.2, XM_005675289.3, XM_018048956.1, XM_018065817.1, XM_018044122.1, XM_018059609.1, XM_013964769.2, XM_018042429.1, XM_018042307.1, XM_018064745.1, XM_018060043.1, XM_018049271.1, XM_018052300.1, XM_018063036.1, NM_001314202.1, XM_018060488.1, XR_001296084.2, XM_013967227.2, XM_018048952.1, NM_001285750.1, XM_013966480.2, XM_018055908.1, XM_018049691.1, XM_005693569.2, XM_018062409.1, XM_018050856.1, XM_005674982.3, XM_005685763.3, XM_005694519.3, XM_018046304.1, XM_018065816.1, XM_018055186.1, XM_018054083.1, XM_013975781.2, XM_018055663.1, XM_018060379.1, XM_018042309.1, XM_005685503.3, XM_018058634.1, XM_013970271.2, XM_018060042.1, XM_018040360.1, XM_005684675.3, XM_013968877.2, XM_005675332.3, XM_005685146.3, XM_018064672.1, XM_005681862.2, XM_005688564.3, XR_001296083.2, XM_018041327.1, XM_018060261.1, XM_018048951.1, XM_005681464.3, XM_018064671.1, XM_018043395.1, XM_013964774.2, XM_018064746.1, XM_018048420.1, XM_018060377.1, XM_018038791.1, XM_005694472.3, XM_013964770.2, XM_018041325.1, XM_018055912.1, XM_018049270.1, XM_018060044.1, XM_018067051.1, XM_018042304.1, XM_018042305.1, XM_018064292.1, XM_005688957.3, XM_005695085.3, XM_018038788.1, XM_018053231.1, XM_018042303.1, XM_018043122.1, XM_018055664.1, XM_018055665.1, XM_005685145.3, XM_005699502.2, XM_018053232.1 |
| 23 | [Aldosterone synthesis and secretion](../../../AppData/Local/Temp/Temp1_VETR-D-18-00038.zip/Cont-vs-PPRV_DEGseq_map/map04925.html) | XM_018043789.1, XM_018062643.1, XM_018062483.1, XM_018058079.1, XM_013963001.2, XM_018040434.1, XM_018061124.1, XM_018045824.1, XM_018054331.1, XM_005680366.2, XM_018061046.1, XM_018054045.1, XM_018058967.1, XM_018044580.1, XR_001918100.1, XM_005680737.3, XM_018040963.1, XM_018043783.1, XM_018060784.1, XM_005697075.3, XM_005691447.3, XM_018066370.1, XM_018060785.1, XM_013972607.2, XM_005680364.3, XM_005699952.3, XM_018058962.1, XM_018062484.1, XM_018061125.1, XM_018039261.1, XM_018050749.1, XM_018062652.1, XM_018043417.1, XM_005680365.2, XM_018044579.1, XM_005697710.3, XM_018039561.1, XM_018039262.1, XM_013963209.2, XM_018061401.1, XM_005688518.3, XM_018041016.1, XM_018043047.1, XM_018045830.1, XM_005679333.3, XM_018046614.1, XM_018044574.1, NM_001285578.1, XM_018066371.1, XM_018057813.1, XM_018039260.1, XM_018057013.1, XM_013970799.2, XM_018061402.1, XM_018061047.1, XM_018043593.1, XM_018049980.1, XR_001295516.2, XM_018058966.1, XM_018046617.1, XM_018047277.1, XM_018062482.1, XM_018044577.1, XM_018044573.1, XM_005682573.3, XM_005690882.3, XM_005680367.3, XM_005676485.3, XM_005685150.3, XM_005686574.3, XM_013966779.2, XM_018062650.1, XM_018046615.1, XM_018045834.1, XM_018058968.1, XM_005691608.3, XM_018044575.1, XM_018049981.1, XM_013973627.2, XM_018055130.1, XM_018061403.1, XM_005695630.3, XM_018043416.1, XM_018062173.1, XM_018053836.1, XR_001296033.2, XM_018058965.1, XM_018066628.1, XM_018044583.1, XM_005681792.3, XM_018062649.1, XM_018058970.1, XM_018057012.1, XM_013976574.2, XM_018054044.1, XM_018044572.1, XM_018039259.1, XM_005676482.3, XM_018062651.1, XM_018044576.1, XM_018061207.1, XM_005685149.3, XM_018046613.1, XM_018044578.1, XM_005697074.3, XM_018044000.1, XM_013976047.2, XM_013963212.2, XM_005694349.2, XM_005699951.1, XM_018046616.1 |
| 24 | [Fc epsilon RI signaling pathway](../../../AppData/Local/Temp/Temp1_VETR-D-18-00038.zip/Cont-vs-PPRV_DEGseq_map/map04664.html) | XM_005681861.3, XM_018061294.1, XM_013964768.2, XM_005679127.2, XM_018063971.1, XM_018061293.1, XM_018063035.1, XM_018055911.1, XM_018054409.1, XM_005676159.3, XM_005697610.3, XM_018055187.1, XM_018050987.1, XM_005685130.3, XM_018061289.1, XM_018060378.1, XM_018051619.1, XM_018060016.1, XM_018043534.1, XM_018061295.1, XM_018048959.1, XM_018062407.1, XM_018062408.1, XM_018053228.1, XM_018042428.1, XM_013974872.2, XM_018047231.1, XM_018042282.1, XM_005699064.3, NM_001286443.1, XM_018061292.1, XM_018054401.1, XM_005699065.3, XM_005690990.2, XM_018060732.1, XM_018062410.1, XM_013968554.2, XM_018051622.1, XM_005700661.3, XM_018058447.1, XM_005684782.3, XM_018040436.1, XM_018060376.1, XM_005681863.3, XM_005679129.3, XM_018054206.1, XM_018042281.1, XM_005700662.3, XM_018049689.1, XM_005691412.3, XM_013968553.2, XM_005675289.3, XM_018048956.1, XM_018065817.1, XM_018059609.1, XM_018054405.1, XM_013964769.2, XM_018042429.1, XM_018064745.1, XM_018054406.1, XM_018061288.1, XM_018063036.1, NM_001314202.1, XM_018060488.1, XM_018052324.1, XM_018042279.1, XM_013967227.2, XM_018048952.1, NM_001285750.1, XM_005681533.3, XM_018055908.1, XM_018049691.1, XM_018062409.1, XM_018050856.1, XM_018042278.1, XM_005685763.3, XM_018065816.1, XM_018055186.1, XM_018054083.1, XM_018042280.1, XM_018054402.1, XM_018054408.1, XM_018060379.1, XM_005680676.3, XM_018058634.1, XM_013970271.2, XM_018040360.1, XM_018054403.1, XM_013968877.2, XM_005675332.3, XM_005685146.3, XM_018064672.1, XM_005681862.2, XM_005688564.3, XM_018060261.1, XR_001919756.1, XM_018048951.1, XM_005685526.3, XM_018064671.1, XM_013964774.2, XM_018064746.1, XM_018048420.1, XM_018061291.1, XM_018060377.1, XM_018038791.1, XM_005694472.3, XM_018065433.1, XM_018061286.1, XM_013964770.2, XM_018054407.1, XM_018055912.1, NM_001285685.1, XM_018067051.1, XM_005680013.2, XM_018064292.1, XM_018038788.1, XM_018053231.1, XM_005685953.3, XM_018054431.1, XM_005685145.3, XM_018053232.1 |
| 25 | [Insulin secretion](../../../AppData/Local/Temp/Temp1_VETR-D-18-00038.zip/Cont-vs-PPRV_DEGseq_map/map04911.html) | XM_018045824.1, XM_018058967.1, XM_018062471.1, XM_018045956.1, XM_018044403.1, XM_018058962.1, XM_018063293.1, XM_018045830.1, XM_005685542.3, XM_018058966.1, XM_005687813.3, XM_005692304.2, XM_005687812.3, XM_018045834.1, XM_005677233.3, XM_018058968.1, XM_005677232.3, XM_018058965.1, XM_018058970.1, XM_018062470.1, XM_018056518.1 |
| 26 | [Acute myeloid leukemia](../../../AppData/Local/Temp/Temp1_VETR-D-18-00038.zip/Cont-vs-PPRV_DEGseq_map/map05221.html) | XM_005681861.3, XM_018064273.1, XM_013964768.2, XM_018056451.1, XM_018056824.1, XM_005679127.2, XM_018063971.1, NM_001285748.1, XM_013970381.2, XM_018063035.1, XM_018053461.1, XM_018055911.1, XM_005697610.3, XM_005683110.3, XM_018047989.1, XM_018055187.1, XM_018050987.1, XM_005685130.3, XM_018060378.1, XM_018051619.1, XM_018060016.1, XM_018048959.1, XM_018062407.1, XM_018062408.1, XM_018043792.1, XM_018053228.1, XM_018042428.1, XM_005681590.3, XM_018043463.1, XM_013974872.2, XM_018047231.1, XM_018053460.1, XM_018058500.1, XM_018050610.1, XR_001918229.1, XM_018056811.1, XM_018062410.1, XM_005675490.2, XM_013968554.2, XM_018051622.1, XM_005698535.3, XM_018058447.1, XM_005684782.3, XM_018042871.1, XM_018060376.1, XM_005681863.3, XM_018060738.1, XM_005679129.3, XM_018064279.1, XM_018053462.1, XM_018054206.1, XM_018049689.1, XM_005700145.2, XM_013968553.2, XM_005675289.3, XM_018048956.1, XM_018065817.1, XM_018049858.1, XM_018043794.1, XM_018043791.1, XM_018059609.1, XM_013964769.2, XM_018042429.1, XM_018064745.1, XM_018063036.1, NM_001314202.1, XM_018060488.1, XM_018049427.1, XM_018060739.1, XM_013967227.2, XM_018048952.1, NM_001285641.1, NM_001285750.1, XM_018055908.1, XM_005701006.3, XM_018049691.1, XM_018062409.1, XM_018053966.1, XM_018059919.1, XM_018050856.1, XM_018064276.1, XM_005685763.3, XM_005693259.3, XM_018065816.1, XM_018055186.1, XM_018054083.1, XM_005683226.3, XM_018060379.1, XM_005680373.3, XR_001918230.1, XM_013970271.2, XM_013968877.2, XM_005678171.3, XM_005675332.3, XM_005685146.3, XM_018064672.1, XM_005681862.2, XM_005688564.3, XM_018060261.1, XM_018048951.1, XM_018049857.1, XM_018058502.1, XM_018049721.1, XM_018053831.1, XM_018064671.1, XM_013964774.2, XM_018064746.1, XM_018050609.1, XR_001918228.1, XM_018048420.1, XM_018060377.1, XM_018038791.1, XM_018043793.1, XM_005694472.3, XM_018060089.1, XM_018061071.1, XM_013965671.2, XM_013964770.2, XM_018053830.1, XM_005678170.3, XM_018055912.1, XM_018063972.1, XM_018067051.1, XM_018059918.1, XM_018064278.1, XM_018063570.1, XM_018064292.1, XM_018064277.1, XM_013968876.2, XM_018038788.1, XM_018053231.1, XM_018045593.1, XM_018043795.1, XM_018064274.1, XM_005685145.3, XR_001918643.1, XM_018053232.1 |
| 27 | [Toxoplasmosis](../../../AppData/Local/Temp/Temp1_VETR-D-18-00038.zip/Cont-vs-PPRV_DEGseq_map/map05145.html) | XM_018043789.1, XM_018063742.1, XM_018056082.1, XM_018042372.1, XM_018043172.1, XM_013976840.2, XM_018066399.1, XM_005681861.3, XM_018041732.1, XM_018052651.1, XM_018066843.1, XM_018042366.1, XM_013968498.2, XM_018045824.1, XM_013964768.2, XM_005682966.2, XM_018063743.1, XM_018039549.1, XM_018067223.1, XM_018042373.1, XM_005691557.3, XM_018054331.1, XM_018039143.1, XM_018048309.1, XM_005684604.3, XM_018063971.1, XM_018044847.1, XM_005699629.3, XM_018038998.1, XM_018062732.1, XM_018063035.1, XM_018059930.1, XM_018055911.1, XM_018047978.1, XM_018066848.1, XM_018062351.1, XM_018044580.1, XM_018040157.1, XM_018052653.1, XM_018064235.1, XM_005697610.3, XM_018063875.1, NM_001314233.1, XM_018055187.1, XM_018063933.1, XM_018061066.1, XM_018058039.1, XM_013966112.2, XM_005691555.3, XM_018039574.1, XM_018059200.1, XM_018044499.1, XM_018047015.1, XM_018059308.1, XM_018043783.1, XM_018038324.1, XM_018060784.1, XM_018063674.1, XM_018048607.1, XM_018062706.1, XM_005680058.3, XR_001917262.1, XM_018044975.1, XM_018051619.1, XM_018060785.1, XM_018047900.1, XM_018044783.1, NM_001314206.1, XM_005695156.3, XM_018056030.1, XM_018065098.1, XM_018062353.1, XM_005683168.3, XM_018041323.1, XM_018056029.1, XM_018048959.1, XM_018038480.1, XM_013973350.2, XM_018060045.1, XM_018045647.1, XM_018063048.1, XM_018062407.1, XM_018060727.1, XM_018062408.1, XM_018048960.1, XM_018061819.1, XM_005679901.2, XM_018062281.1, XM_018056710.1, XM_018038932.1, XM_018038929.1, XM_018053228.1, XM_018049765.1, XM_005682502.2, XM_018042428.1, XM_018056079.1, XM_018057132.1, XM_018044361.1, XM_005682995.3, XM_018065612.1, XM_013974872.2, XM_005688325.2, XM_005695611.3, XM_018061978.1, XM_018039261.1, XM_018057919.1, XM_018039015.1, XM_018063906.1, XM_018043205.1, XM_018050749.1, XM_013974836.2, XM_018051036.1, XM_018055301.1, XM_018063884.1, XM_018047019.1, XM_018061853.1, XM_018040596.1, XM_018066044.1, XM_005709702.3, XM_005680937.3, XM_018046950.1, NM_001285561.1, XM_018060472.1, XM_018062652.1, XM_005695056.3, XM_005709386.3, XM_018042978.1, XM_018044991.1, XM_018043092.1, XM_013969390.2, XM_018050945.1, XM_018044358.1, NM_001314303.1, XM_018044579.1, XM_005697710.3, XM_018041623.1, XM_018039262.1, XM_005676847.3, XM_018061852.1, XM_018062410.1, XM_018063876.1, XM_005697807.3, XM_018047021.1, XM_018041991.1, XM_013968554.2, XM_018051622.1, XM_018052462.1, XM_018058447.1, XM_013962884.2, XM_018042363.1, XM_005684782.3, XM_018047022.1, XM_005682965.2, XM_018044978.1, XM_013964239.2, XM_005681863.3, XM_018040539.1, XM_018066563.1, XM_005700002.1, XM_018042364.1, XM_018038397.1, XM_018054206.1, XM_018052642.1, XM_018056196.1, XM_018039351.1, XM_018052644.1, XM_018059201.1, XM_018049689.1, XM_018063839.1, XM_005697806.3, XM_018052702.1, XM_018045830.1, XM_018057916.1, XM_013968553.2, XM_018061979.1, XM_018048956.1, XM_018065817.1, XM_018043189.1, XM_018049764.1, XM_018053992.1, XM_018042367.1, XM_018056024.1, XM_018042371.1, XM_018038930.1, XM_018041625.1, XM_018052649.1, XM_018040536.1, XM_005691558.3, XM_018041929.1, XM_005677998.3, XM_018041044.1, XM_018044574.1, XM_013964769.2, XM_018042429.1, XM_018052464.1, XM_018054838.1, XM_018044676.1, XM_018064745.1, XM_018059198.1, XM_018060043.1, XM_018047020.1, XM_005678611.3, XM_018039141.1, XM_018040537.1, XM_018041990.1, XM_018042043.1, XM_018039142.1, XM_018065480.1, XM_018063036.1, XM_018040933.1, XM_018040077.1, XM_013972641.2, NM_001314202.1, XM_018049763.1, XR_001917959.1, XM_018060488.1, XM_018043089.1, XR_001296084.2, XM_018058361.1, XM_018039260.1, XM_018040538.1, XM_013967227.2, XM_018060837.1, XM_005694801.1, XM_018061079.1, XM_018057013.1, XM_018048952.1, XM_018065613.1, XM_018039633.1, XM_018044360.1, NM_001285750.1, XM_018056195.1, XM_013966480.2, XM_018049891.1, XM_018038325.1, XM_005687505.3, XM_018046273.1, XM_018055908.1, XM_018045488.1, XM_018049691.1, XM_018042369.1, XM_018038931.1, XM_018062354.1, XM_005693569.2, XM_005684135.3, XM_018061047.1, XM_018056027.1, XM_018049980.1, XM_018062409.1, XM_018049762.1, XM_018050520.1, XM_018038396.1, XM_018052645.1, XM_018061057.1, XM_018066385.1, XM_018052641.1, XM_018066596.1, XM_018049761.1, XM_018047018.1, XM_018045402.1, XM_018064937.1, XM_018044609.1, XM_005695149.3, XM_005685763.3, XM_018044577.1, XM_018044573.1, XM_018066740.1, XM_005682573.3, XM_018052650.1, XM_018063974.1, XM_018066321.1, XR_001296591.2, XM_018044359.1, XM_018043010.1, XM_018047023.1, XM_018039329.1, XM_018039521.1, XM_018066407.1, XM_018061078.1, XM_018043161.1, XM_018065816.1, XR_001917399.1, XM_018055186.1, NM_001314229.1, XM_018054083.1, XM_018050706.1, XM_005676292.3, XM_018062538.1, XM_018055300.1, XM_018065614.1, XM_018063911.1, XM_018056026.1, XM_018061272.1, XM_013974426.2, XM_018062650.1, XM_018045131.1, XM_005678655.2, XM_005677754.2, XM_005677146.3, XM_018061449.1, XM_018067016.1, XM_018049197.1, XM_018046271.1, XM_005679294.1, XM_018064130.1, XM_018064422.1, XM_018045834.1, XM_005679444.3, XM_005680373.3, XM_018049710.1, XM_018058634.1, XM_018044677.1, XM_018052654.1, XM_018048308.1, XM_018052646.1, XM_018044977.1, XM_005683169.3, XM_018042375.1, XM_013970271.2, XR_001919444.1, XM_018039565.1, XM_018063934.1, XM_018061774.1, XM_018052502.1, XM_018060042.1, XM_018041064.1, XM_018040859.1, XM_018040879.1, XM_018044575.1, XM_018040360.1, XM_018043011.1, XM_018049766.1, XM_018038837.1, XM_018060842.1, XM_018049981.1, XM_005684675.3, XM_018051425.1, XM_018056422.1, XM_013968877.2, XM_013973627.2, XM_005675332.3, XM_018063740.1, XM_005709548.3, XM_018042377.1, XM_005681862.2, XM_005688564.3, XR_001296083.2, XM_018041327.1, XM_018060846.1, XR_001919645.1, XM_018060261.1, XM_018040922.1, XM_018052643.1, XM_018048540.1, XM_018048951.1, XM_013968032.2, XM_005697716.3, XM_018039144.1, XM_018040877.1, XM_005682964.2, XM_005695630.3, XM_018062352.1, XM_018044477.1, XM_005679443.3, XM_018052648.1, XM_018062635.1, XM_018045484.1, XM_005694470.3, XM_018044782.1, XM_018062173.1, XM_018042378.1, XM_018053836.1, XM_005697805.3, XM_018062791.1, XM_018044500.1, XM_013964774.2, XM_018043169.1, XM_018064746.1, XM_005689565.3, XM_018039145.1, XM_018066628.1, XM_018052652.1, XM_005695610.2, XM_018065735.1, XM_018042370.1, XM_013971456.2, XM_018044583.1, XM_018057613.1, XM_018040878.1, XM_018044976.1, XM_018062649.1, XM_018038791.1, XM_018039786.1, XM_018056420.1, XM_018057012.1, XM_018055534.1, XM_018047017.1, NM_001285703.1, XM_018055302.1, XM_013976574.2, XM_018041993.1, XM_018044572.1, XM_018042365.1, NM_001314219.1, XM_018039259.1, XM_018063889.1, XM_013964770.2, XM_018044901.1, NM_001285739.1, XM_018051946.1, XM_005679295.1, XM_018065864.1, XM_018047738.1, XM_018045482.1, XM_005688223.3, XM_013971767.1, XM_018062651.1, XM_018039309.1, XM_018041325.1, XM_018052655.1, XR_001919644.1, XM_018044576.1, XM_018061207.1, XM_018055912.1, XM_005684175.3, XM_018064423.1, XM_018044409.1, XM_005691556.3, XM_005687533.3, XM_018061180.1, XM_018052461.1, XM_018044578.1, XM_018051703.1, XM_005685946.3, XM_018047014.1, XM_018041045.1, XM_018066046.1, XM_018065787.1, XM_018060044.1, XM_018062581.1, XM_018042374.1, XM_018044911.1, XM_005674769.3, XM_018067051.1, XM_018038897.1, XM_018063877.1, XM_018041624.1, XM_018064375.1, XM_018066586.1, XM_013971492.2, XM_018056081.1, XM_018057840.1, XM_018063888.1, XM_018044000.1, XM_018051038.1, XM_018048550.1, XM_018040883.1, XM_018043179.1, XM_018064292.1, XM_018041992.1, XM_018054172.1, XM_018054709.1, XM_018039789.1, XM_018038788.1, XM_005688087.3, XM_018056421.1, XM_018052683.1, XM_018044362.1, XM_005678654.3, XM_018053231.1, XM_018038232.1, NM_001285566.1, XM_018045381.1, XM_018045509.1, XM_018053994.1, XM_018044675.1, XM_018059202.1, XM_018060728.1, XM_018056883.1, XM_018056086.1, XM_013974809.2, XM_018061977.1, XM_018063714.1, XM_018045771.1, XM_018061059.1, XM_018043199.1, XM_005694349.2, XM_018047024.1, XM_018063741.1, XM_018051424.1, XM_018044476.1, XM_018058280.1, XM_018043091.1, XM_018049760.1, XM_018062580.1, XM_018038398.1, XM_013968497.2, XM_018053232.1 |
| 28 | [Vascular smooth muscle contraction](../../../AppData/Local/Temp/Temp1_VETR-D-18-00038.zip/Cont-vs-PPRV_DEGseq_map/map04270.html) | XM_018062643.1, XM_013963001.2, XM_005681861.3, XM_018064273.1, XM_018061124.1, XM_013964768.2, XM_018056824.1, XM_018052298.1, XM_005680366.2, XM_018052301.1, XM_018042311.1, XM_018054045.1, XM_018055487.1, XM_018054409.1, XR_001918100.1, XM_018050987.1, XM_005680737.3, XM_005685130.3, XM_005697075.3, XM_005691447.3, XM_018066370.1, XM_018051619.1, XM_018061256.1, XM_018047856.1, XM_013972607.2, XM_005680364.3, XR_001918973.1, XM_005699952.3, XM_018042310.1, XM_018048959.1, NM_001285674.1, XM_018042428.1, XM_005681590.3, XM_018054634.1, XM_018061125.1, XM_018064706.1, XM_018058500.1, XM_005698163.3, XM_018047050.1, XM_005691313.3, XM_005680365.2, XM_018054401.1, XM_005690990.2, XM_018045751.1, XM_018042308.1, XM_018060732.1, XM_018039561.1, XM_005691312.2, XM_018061944.1, XM_018056811.1, XM_018051622.1, XM_005688518.3, XM_018041016.1, XM_018061941.1, XM_005681863.3, XM_018064279.1, XM_018054206.1, XM_018043047.1, XM_018052297.1, XM_018049689.1, XM_005700145.2, XM_018048956.1, XM_018043394.1, XM_005679333.3, XM_018054405.1, XM_013964769.2, XM_018042429.1, XM_018042307.1, XM_018064745.1, XM_018054406.1, XM_018066371.1, XM_018049271.1, XM_018057813.1, XM_018052300.1, XM_018064036.1, XM_005675953.3, NM_001314202.1, XM_018055485.1, XM_018049427.1, XM_018048952.1, XM_013970799.2, XM_018046143.1, XM_018049691.1, XM_018043593.1, XR_001295516.2, XM_018065622.1, XM_018050856.1, XM_018064276.1, XM_005685763.3, XM_018061942.1, XM_005680367.3, XM_018061940.1, XM_005676485.3, XM_005685150.3, XM_018054402.1, XM_005683226.3, XM_005686574.3, XM_018054408.1, XM_013966779.2, XM_005681818.3, XM_018054635.1, XM_018048449.1, XM_018042309.1, XM_005680373.3, XM_018058573.1, XM_018044096.1, XM_018054403.1, XR_001919417.1, XM_005678171.3, XM_005685146.3, XM_018064672.1, XM_005681862.2, XM_018048951.1, XM_018058502.1, XM_018061943.1, XM_005685526.3, XR_001296033.2, XM_018064671.1, XM_018055488.1, XM_013964774.2, XM_018064746.1, XM_018038791.1, XM_005694472.3, XM_018054044.1, XM_013964770.2, XM_005676482.3, XM_018041140.1, XM_005678170.3, XM_018054407.1, XM_018049270.1, XM_005685149.3, XM_018041138.1, XM_018064278.1, XM_018042304.1, XM_005686948.3, XM_018042305.1, XM_005697074.3, XM_018064292.1, XM_018055486.1, XM_018064277.1, XM_018038788.1, XM_018042303.1, XM_018045593.1, XM_013976047.2, XM_018041139.1, XM_018064274.1, XM_018047883.1, XM_005685145.3, XM_005694349.2, XM_005699951.1, XM_013964470.2 |
| 29 | [Phospholipase D signaling pathway](../../../AppData/Local/Temp/Temp1_VETR-D-18-00038.zip/Cont-vs-PPRV_DEGseq_map/map04072.html) | XM_018055177.1, XM_005681861.3, XM_018062722.1, XM_013964768.2, XM_013962541.2, XR_001920008.1, XM_005679127.2, XM_005680381.3, XM_018063971.1, XM_018047786.1, NM_001285748.1, XM_018064091.1, XM_018063035.1, NM_001285656.1, XM_018054049.1, XM_018055911.1, XM_018054409.1, XM_018047363.1, XM_018041498.1, XM_013967930.2, XM_005697610.3, XM_018055187.1, XM_018050987.1, XM_018062714.1, XM_005685130.3, XM_005687462.3, XM_005675378.3, XM_018050170.1, XM_018060378.1, XM_018051619.1, XM_005680005.2, XM_005680008.3, XM_018060016.1, XM_018048959.1, XM_018062407.1, XM_018054372.1, XM_018062408.1, XM_018062720.1, XM_018053228.1, XM_018042428.1, XM_005676376.3, XM_018051810.1, XM_018062713.1, XM_018047802.1, XR_001918038.1, XM_013974872.2, XM_018062724.1, XM_018046894.1, XM_005675655.3, XM_005687933.3, XM_018047231.1, XM_018050527.1, XM_005697888.3, XM_005677615.3, XM_005684957.3, XM_005693625.3, XM_018062721.1, XM_018054401.1, XM_018048105.1, XM_005690990.2, XM_018060732.1, XM_005678977.3, XM_018062410.1, XM_018038356.1, XM_013976723.2, XM_005678056.3, XM_018047360.1, XM_005681855.3, XM_013968554.2, XM_018051622.1, XM_005678985.3, XM_018058447.1, XM_005684782.3, XM_013964590.2, XM_005693960.3, XM_018060376.1, XM_018047364.1, XM_005681863.3, XM_013976935.2, XM_005679129.3, XM_018059162.1, XM_018054206.1, XM_005677614.3, XM_018049689.1, XM_013968553.2, XM_005675289.3, XM_018048956.1, XM_013972738.2, XM_018065817.1, XM_018048018.1, XM_005679429.3, XM_018059609.1, XM_018054405.1, XM_013964769.2, XM_018042429.1, XM_005678357.3, XM_018056791.1, XM_018064745.1, XM_005693961.3, XM_018061554.1, XM_018054406.1, XM_018048019.1, XM_018056794.1, XM_018054571.1, XM_018059164.1, XM_018064220.1, XM_018044352.1, XM_018063036.1, XM_018056792.1, XM_018058397.1, NM_001314202.1, XM_018060488.1, XM_005683717.3, XM_013967227.2, XM_018048952.1, NM_001285750.1, XM_018055908.1, XM_018049691.1, XM_005684958.3, XM_018050833.1, NM_001285761.1, XM_018062409.1, XM_018059160.1, XM_018059919.1, XM_005675639.3, XM_018050856.1, XM_018038495.1, XM_018064986.1, XM_005685763.3, XM_018062710.1, XM_018062725.1, XM_018059166.1, XM_018057897.1, XM_018065816.1, XM_018055186.1, XM_018054083.1, XM_018041036.1, XM_018054402.1, XM_018054408.1, XM_018060379.1, XM_018054611.1, XM_018041425.1, XM_018050834.1, XM_018061830.1, XM_005675640.3, XM_018054048.1, XM_013970271.2, XM_018063749.1, XM_013963165.2, XM_005684959.3, XM_018060838.1, XM_018054403.1, XM_005677617.3, XM_013968877.2, XM_005675332.3, XM_005685146.3, XM_018064672.1, XM_005681862.2, XM_005688564.3, XM_018047359.1, XM_018060261.1, XM_018046893.1, XM_018058396.1, XM_018048951.1, XM_018058395.1, XM_018064988.1, XM_005678984.3, XM_018041043.1, XM_005685526.3, NM_001285594.1, XM_018064671.1, XM_013964774.2, XM_018064746.1, XM_018064499.1, XM_018062712.1, NM_001287233.1, XM_018048420.1, XM_018062718.1, XM_005680383.3, XM_018060377.1, XM_018046856.1, XM_018038791.1, XM_018062723.1, XM_018048104.1, XM_005694472.3, XM_018059161.1, XM_018062717.1, XM_018044227.1, XM_018040905.1, XM_013964770.2, XM_018059165.1, XM_005693177.3, XM_005684192.3, XM_018050781.1, XM_018058394.1, XM_018054407.1, XM_018055912.1, XM_018062711.1, XM_005675103.3, XM_018059167.1, XM_018067051.1, XM_018059918.1, XM_018065351.1, XM_018062719.1, XM_005691714.3, XM_018059163.1, XM_013968393.2, XM_018064292.1, XM_018041050.1, XM_018038788.1, XM_018061555.1, XM_013963166.2, XM_018054047.1, XM_018041495.1, XM_018053231.1, XM_018047553.1, XM_005693897.2, XM_005675512.3, XM_005685145.3, XM_018048748.1, XM_018053232.1 |
| 30 | [Oxytocin signaling pathway](../../../AppData/Local/Temp/Temp1_VETR-D-18-00038.zip/Cont-vs-PPRV_DEGseq_map/map04921.html) | XM_018043789.1, XM_005699593.3, XM_018044652.1, XM_018062643.1, XM_013973238.2, XM_018058079.1, XM_013963001.2, XM_018043283.1, XM_005681861.3, XM_013970632.2, XM_018061268.1, XM_018061124.1, XM_018045824.1, XM_013964768.2, XM_018039549.1, XM_018052298.1, XM_018054331.1, XM_005695088.3, XM_005682490.2, XM_005680366.2, XM_018052301.1, XM_018061046.1, XM_018042311.1, XR_001917497.1, XM_018055980.1, XM_018054045.1, XM_018063078.1, XM_018058967.1, XM_018055487.1, XM_013971629.2, XM_018054409.1, XM_018044580.1, XM_018054628.1, XR_001918100.1, XM_018050987.1, XM_005680737.3, XM_018059746.1, XM_018058560.1, XM_005685130.3, XM_018040963.1, XM_018043783.1, XM_018060784.1, XM_005697075.3, XM_018060171.1, XM_005691447.3, XM_018066370.1, XM_018066401.1, XM_018051619.1, XM_013970631.2, XM_018060785.1, XM_005680647.3, XM_018061256.1, XM_018047856.1, XM_018043280.1, XM_005686991.3, XM_013972607.2, XM_005699594.3, XM_005678693.3, XM_005680364.3, XM_018061265.1, XR_001918973.1, XM_005699952.3, XM_018043282.1, XM_018042310.1, XM_018041323.1, XM_018048959.1, XM_018043281.1, XM_018046692.1, XM_018043288.1, XM_018046693.1, XM_018065501.1, XM_018058962.1, NM_001285674.1, XM_018061266.1, XM_018042428.1, XM_018054634.1, XM_018043180.1, XM_018062590.1, XM_018058066.1, XM_018066400.1, XM_018061125.1, XM_018039261.1, XM_018050749.1, XM_018044746.1, XM_018062652.1, XM_018049335.1, XM_018047050.1, XM_005691313.3, XM_005680365.2, XM_018054401.1, XM_005690990.2, XM_018045751.1, XM_018042308.1, XM_018044579.1, XM_018060732.1, XM_005697710.3, XM_018057817.1, XM_018039561.1, XM_018039262.1, XM_005691312.2, XM_018061944.1, XM_018051622.1, XM_005688518.3, XM_018041016.1, XM_018061941.1, XM_018046688.1, XM_005681863.3, XM_018054206.1, XM_018055439.1, XM_018043047.1, XM_018052297.1, XM_005681337.3, XM_018049689.1, XM_018046689.1, XM_018045830.1, XM_005681336.3, XM_018040605.1, XM_018048956.1, XM_018043394.1, XM_005679333.3, XM_013962290.2, XM_018054405.1, XM_018044574.1, XM_013964769.2, NM_001285647.1, XM_018042429.1, XM_018042307.1, XM_018064745.1, XM_018046694.1, XM_018054406.1, XM_018066371.1, XM_018049271.1, XM_018057813.1, XM_018052300.1, XM_018064036.1, XM_018061267.1, XM_018051501.1, NM_001314202.1, XM_018055485.1, XM_018039260.1, XM_018059798.1, XM_018057013.1, XM_018048952.1, XM_013970799.2, XR_001917495.1, XM_018046143.1, XM_018049691.1, XM_005693569.2, XM_018061047.1, XM_018043593.1, XM_018049980.1, XM_013973240.2, XR_001295516.2, XM_018064432.1, XM_018065622.1, XM_018058966.1, XM_018050856.1, XM_018054332.1, XM_005702078.3, XM_005685763.3, XM_005694519.3, XM_018044577.1, XM_018044573.1, XM_005682573.3, XM_018061942.1, XM_018046691.1, XM_013970533.2, XM_005680367.3, XM_005684278.2, XM_018061940.1, XM_005676485.3, XM_005685150.3, XM_018054402.1, XM_018055663.1, XM_005686574.3, XM_018054408.1, XM_013966779.2, XM_018054635.1, XM_018062650.1, XM_018048449.1, XM_018042309.1, XM_013962990.2, XM_005685503.3, XM_018045834.1, XM_018059747.1, XM_018051760.1, XM_018040964.1, XM_018064433.1, XM_018054630.1, XM_018058573.1, XM_018058968.1, XM_018044096.1, XM_005691608.3, XM_018044575.1, XM_018054403.1, XM_018049981.1, XR_001919417.1, XM_018066076.1, XM_013973627.2, XM_018055130.1, XM_018066944.1, XM_005685146.3, XM_018064672.1, XM_005681862.2, XM_018041327.1, XM_018062591.1, XM_018048951.1, XM_018061943.1, XM_005695630.3, XM_005695428.3, XM_005685526.3, XR_001918314.1, XM_018062173.1, XM_005681464.3, XM_018053836.1, XR_001296033.2, XM_018064671.1, XM_018059745.1, XM_018055488.1, XM_013964774.2, XM_018058965.1, XM_018064746.1, XM_005682572.3, XM_018066628.1, XM_013971630.2, XM_018059748.1, XM_018044583.1, XM_018057613.1, XM_018054629.1, XM_018062649.1, XM_018038791.1, XM_018058970.1, XM_005694472.3, XM_005695430.2, XM_018057012.1, XM_018041744.1, XM_013976574.2, XM_018054044.1, XM_018044572.1, XM_018039259.1, XM_018043284.1, XM_013964770.2, XM_005676482.3, XM_018046690.1, XM_018062651.1, XM_018058102.1, XM_018041325.1, XM_018044576.1, XM_018054407.1, XM_018061207.1, XM_018049270.1, XM_018043190.1, XM_005685149.3, XM_018044578.1, XM_005695427.3, XM_005682905.3, XM_018042304.1, XM_018066075.1, XM_005686948.3, XM_018042305.1, XM_005697074.3, XM_018061264.1, XM_018048933.1, XM_018044000.1, XM_018064292.1, XM_005682904.3, XM_018055486.1, XM_005695429.3, XM_005688957.3, XM_005695085.3, XM_018038788.1, XM_018058103.1, XM_018042303.1, XM_018065502.1, XM_013976047.2, XM_005695431.3, XM_018055664.1, XM_018055665.1, XM_005685145.3, XM_005694349.2, XM_018043287.1, XM_005699951.1, XM_018055626.1, XR_001917496.1, XM_013962471.2 |
| 31 | [Toll-like receptor signaling pathway](../../../AppData/Local/Temp/Temp1_VETR-D-18-00038.zip/Cont-vs-PPRV_DEGseq_map/map04620.html) | XM_005681861.3, XM_018066843.1, XM_018061294.1, XM_013964768.2, XM_005679127.2, XM_018063971.1, XM_018038998.1, XM_018061293.1, XM_018063035.1, XM_018055911.1, XM_018047978.1, XM_005680586.3, XM_005676159.3, XM_018040157.1, XM_005697610.3, XM_018055187.1, XM_018050987.1, XM_013966112.2, XM_005685130.3, XM_018061289.1, XM_005680058.3, XM_018060378.1, XM_018051619.1, XM_018047900.1, XM_018060016.1, XM_018043534.1, XM_018061295.1, XM_018048959.1, XM_013973350.2, XM_018060045.1, XM_018062407.1, XM_018060727.1, XM_018062408.1, XM_005679901.2, XM_018053228.1, XM_018042428.1, XM_013974872.2, XM_005695611.3, XM_018047231.1, XM_018042282.1, XM_005680937.3, XM_005699064.3, NM_001286443.1, XM_018061292.1, XM_005699065.3, XM_018043092.1, XM_018050945.1, XM_018062410.1, XM_013968554.2, XM_018051622.1, XM_005700661.3, XM_018058447.1, XM_005684782.3, XM_018040436.1, XM_018060376.1, XM_005681863.3, XM_018066563.1, XM_005679129.3, XM_018054206.1, XM_018042281.1, XM_005700662.3, XM_018049689.1, XM_005691412.3, XM_013968553.2, XM_005675289.3, XM_018048956.1, XM_018065817.1, XM_018059609.1, XM_018041929.1, XM_013964769.2, NM_001314172.1, XM_018042429.1, XM_018064745.1, XM_018060043.1, XM_018061288.1, XM_018063036.1, NM_001314202.1, XM_018060488.1, XM_018043089.1, XR_001296084.2, XM_018052324.1, XM_018042279.1, XM_013967227.2, XM_018048952.1, NM_001285750.1, XM_013966480.2, XM_005681533.3, XM_018055908.1, XM_018049691.1, XM_018062409.1, XM_018050856.1, XM_018042278.1, XM_018044609.1, XM_005685763.3, XM_018057938.1, XM_018065816.1, XM_018055186.1, XM_018054083.1, XM_018042280.1, XM_018050706.1, XM_018060379.1, XM_005680676.3, XM_005679294.1, XM_018064130.1, XM_005680373.3, XM_018058634.1, XM_005688549.3, XM_013970271.2, XM_018060042.1, XM_005684675.3, XM_013968877.2, XM_005675332.3, XM_005685146.3, XM_018064672.1, XM_005681862.2, XM_005688564.3, XR_001296083.2, XM_018060261.1, XR_001919756.1, XM_018048951.1, XM_013968842.2, XM_018064671.1, XM_013964774.2, XM_018064746.1, XM_005695610.2, XM_018048420.1, XM_018061291.1, XM_018060377.1, XM_018038791.1, XM_005694472.3, XM_018065433.1, XM_018061286.1, XM_013964770.2, XM_005679295.1, XM_018047738.1, XM_018055912.1, XM_005687533.3, XM_018051703.1, NM_001285685.1, XM_018060044.1, XM_018067051.1, XM_005680013.2, XM_018057840.1, XM_018048550.1, XM_018064292.1, XM_018054172.1, XM_018038788.1, XM_005688087.3, XM_018053231.1, XM_005685953.3, XM_018054431.1, XM_018060728.1, XM_005685145.3, XM_018043091.1, XM_018053232.1 |
| 32 | [RNA degradation](../../../AppData/Local/Temp/Temp1_VETR-D-18-00038.zip/Cont-vs-PPRV_DEGseq_map/map03018.html) | XR_001917385.1, XM_018048239.1, XM_018042049.1, XM_013963942.2, XM_005682388.3, XM_018047555.1, XM_005696051.3, XM_005680352.3, XM_018039081.1, XM_005676314.3, XM_018047641.1, XM_018057494.1, XM_013970367.2, XM_018042154.1, XM_013965873.2, XM_005680336.3, XM_005690686.3, XM_018048068.1, XR_001918940.1, XM_018048244.1, XM_018059708.1, XM_018061950.1, XM_018067046.1, XM_005675484.3, XM_013974854.2, XM_018061537.1, XM_005695512.3, XM_018039074.1, XM_005686519.3, XM_013964068.2, XM_005686522.3, XM_018050418.1, XM_018048241.1, XR_001917379.1, XM_005692436.3, XM_005683051.3, XM_005679497.3, XR_001917384.1, XM_005676059.3, XM_018063575.1, XM_018064457.1, XM_018059704.1, XM_005680335.3, XM_013963940.2, XM_018059701.1, XM_005680177.3, XM_005682995.3, XM_005680353.3, XM_018039075.1, XM_018048243.1, XM_018051480.1, XM_013963948.2, XM_018042051.1, XM_018056596.1, XM_018042545.1, XM_018064294.1, XM_018059449.1, XM_018048245.1, XM_018055726.1, XM_018050976.1, XM_018055436.1, XM_018065611.1, XM_005683212.3, XM_018057269.1, XM_018047863.1, XM_018059703.1, XM_013964078.2, XM_018050972.1, XM_013971002.2, XM_005678224.3, XM_018038639.1, XM_005695511.3, XM_018058778.1, XM_018066792.1, XM_018047864.1, XM_005675483.3, XM_005697515.3, XM_018053928.1, XM_018038700.1, XM_018064455.1, NM_001314241.1, XM_018061910.1, XM_018048248.1, XM_005698286.3, XM_013965051.2, XM_018053854.1, XM_005681805.2, XM_005695520.3, XM_018059709.1, XM_018064456.1, XM_005692051.3, XM_013962839.2, XM_005683439.3, XM_018063419.1, XM_005695819.3, XM_018042159.1, XM_018049608.1, XM_013964742.2, XM_018039080.1, XM_018051030.1, XM_018044705.1, XM_005699619.3, XR_001295944.2, XM_018053855.1, XM_018061536.1, XM_018047859.1, XM_005695513.3, XM_005679500.3, XR_001918367.1, XM_018064760.1, XM_018063636.1, XM_018067045.1, XM_018059705.1, XM_018039078.1, XM_005680607.3, XM_005680608.3, XM_005680611.3, XM_018051355.1, XM_018039077.1, XM_005695514.3, XM_005695847.2, XM_018048690.1, XM_018047862.1, XM_005695509.3, XM_018047946.1, XM_005680351.3, XM_013973419.2, XM_005678445.3, XM_018051671.1, XM_018055277.1, XM_018042161.1, XM_005680354.3, XM_005682389.3, XM_005686520.3, XM_005692050.3, XR_001917122.1, XM_005686521.3, XM_018041975.1, XM_018053752.1, XM_018039079.1, XM_018048066.1, XM_005681254.3, XM_018048253.1, XM_018061534.1, XM_018060743.1, XM_005692049.3, XM_018057914.1, XM_005676312.2, XM_018048067.1, XM_013965964.2, XM_018039076.1, XM_005683885.3, XM_018042048.1, XM_005679499.3, XM_018039082.1, XM_018060740.1, XM_005683509.3, XM_018060741.1, XM_013965831.2, XM_013964076.2, XM_018041700.1, XM_013975998.2, XM_018066789.1, XM_013963941.2, XM_018056365.1, XM_018048063.1, XM_018048251.1, XM_018065936.1, XM_018049601.1, XM_018061271.1, XM_005684949.3, XM_018041974.1, XM_018044657.1, XM_018041973.1, XM_005690953.2, XM_005699618.3, XM_005678538.3, XM_005679353.3, XM_005679498.3, XM_018059707.1, XM_018043207.1, XM_013974855.2, XM_018055146.1, XM_013965183.2, XM_005694992.3, XM_013971003.2, XM_018048252.1, XM_005699896.3, XM_018047947.1, XM_018060742.1, XM_005676313.2, XM_005680176.1, XR_001918366.1, XM_018058710.1, XM_018061279.1, XM_005681214.3, XM_018042050.1, XM_018065610.1, XM_005676311.2, XM_005680179.3, XM_005679496.3, XM_013963874.2, XM_005695094.1, XM_013974853.2, XM_018047945.1, XM_005687271.3, XM_018047861.1, XM_018048255.1, NM_001314158.1, XM_018053856.1, XM_018053840.1, XM_013965718.2, XM_005686811.3, XM_005679209.3, XM_018048254.1, XM_005679495.3, XM_018055703.1, XM_013971001.2, XM_018045174.1, XM_018048240.1, XM_018042151.1, XM_018048984.1, XM_018059706.1, XM_005680350.3, XM_018067047.1, XM_018053857.1, XM_013973416.2, XM_018059702.1, XM_018058095.1 |
| 33 | [Glioma](../../../AppData/Local/Temp/Temp1_VETR-D-18-00038.zip/Cont-vs-PPRV_DEGseq_map/map05214.html) | XM_018062643.1, XM_018055645.1, XM_013963001.2, XM_005681861.3, XM_018055614.1, XM_018064273.1, XM_018061124.1, XM_018045824.1, XM_013964768.2, XM_013962777.2, XM_018056824.1, XM_005679127.2, XM_005680366.2, XM_018063971.1, NM_001285748.1, XM_018063035.1, XM_018054045.1, XM_018055911.1, XM_018058967.1, XM_005697610.3, XM_018055187.1, XM_018050987.1, XM_005685130.3, XM_005697075.3, XM_005691447.3, XM_018066370.1, XM_018060378.1, XM_018051619.1, XM_013972607.2, XM_005680364.3, XM_018060016.1, XM_005692453.3, XM_005699952.3, XM_018048959.1, XM_018062407.1, XM_018062408.1, XM_018058962.1, XM_018053228.1, XM_018042428.1, XM_018058171.1, XM_005681590.3, XM_013974872.2, XM_018061125.1, XM_018047231.1, XM_018058500.1, XM_005680365.2, XM_018039561.1, XM_018056811.1, XM_018062410.1, XM_013968554.2, XM_018051622.1, XM_005689267.3, XM_005688518.3, XM_018041016.1, XM_018058447.1, XM_005684782.3, XM_018060376.1, XM_005681863.3, XM_005679129.3, XM_018064279.1, XM_018054206.1, XM_018043047.1, XM_018049689.1, XM_005700145.2, XM_018045830.1, XM_013968553.2, XM_005675289.3, XM_018048956.1, XM_018065817.1, XM_005679333.3, XM_018059609.1, XM_018047703.1, XM_013964769.2, XM_018042429.1, XM_018056690.1, XM_018064745.1, XM_018066371.1, XM_018057813.1, XM_018063036.1, NM_001314202.1, XM_018060488.1, XM_018049427.1, XM_013967227.2, XM_018048952.1, NM_001285750.1, XM_013970799.2, XM_018055908.1, XM_018049691.1, XM_018043593.1, XM_018062409.1, XR_001295516.2, XM_005686996.3, XM_018059919.1, XM_018058966.1, XM_018050856.1, XM_018039227.1, XM_018039226.1, XM_018064276.1, XM_005685763.3, XM_005680367.3, XM_018065816.1, XM_018055186.1, XM_018054083.1, XM_005676485.3, XM_005685150.3, XM_018045207.1, XM_005683226.3, XM_005686574.3, XM_013966779.2, XM_018060379.1, XM_018045834.1, XM_005680373.3, XM_018058968.1, XM_013970271.2, XM_005678307.2, XM_013968877.2, XM_005675332.3, XM_005685146.3, XM_013971256.2, XM_018064672.1, XM_005681862.2, XM_005688564.3, XM_018060261.1, XM_018058170.1, XM_018048951.1, XM_018058502.1, XM_018044357.1, XR_001296033.2, XM_018064671.1, XM_013964774.2, XM_018058965.1, XM_018064746.1, XM_018048420.1, XM_018060377.1, XM_018038791.1, XM_018058970.1, XM_005694472.3, XM_018054044.1, XM_013964770.2, XM_005676482.3, XM_018055912.1, XM_005685149.3, XM_018067051.1, XM_018059918.1, XM_018064278.1, XM_005697074.3, XM_018064292.1, XM_018064277.1, XM_018038788.1, XM_018053231.1, XM_018045593.1, XM_013976047.2, XM_018064274.1, XM_005685145.3, XM_005694349.2, XM_005699951.1, XM_018053232.1 |
| 34 | [Chagas disease (American trypanosomiasis)](../../../AppData/Local/Temp/Temp1_VETR-D-18-00038.zip/Cont-vs-PPRV_DEGseq_map/map05142.html) | XM_005681861.3, XR_001917331.1, XM_018066843.1, XM_013964768.2, XM_018039549.1, XM_005679127.2, XM_018063971.1, XM_018063035.1, XM_018055911.1, XM_018047978.1, XM_018040157.1, XM_005697610.3, XM_018055187.1, XM_013966112.2, XM_005680058.3, XM_018060378.1, XM_018051619.1, XM_018047900.1, XR_001917329.1, XM_018060016.1, XM_018050190.1, XM_018048959.1, XM_013973350.2, XM_018062407.1, XM_018060727.1, XM_018062408.1, XM_005679901.2, XM_018053228.1, XM_018042428.1, XM_013974872.2, XM_005695611.3, XM_005682299.3, XM_018059802.1, XM_018047231.1, XM_005680937.3, XR_001917337.1, XM_018043092.1, XM_018050945.1, XM_018062410.1, XR_001917328.1, XM_018042061.1, XM_013968554.2, XM_018051622.1, XM_018058447.1, XM_005684782.3, XM_018059800.1, XM_018060376.1, XM_005681863.3, XM_018066563.1, XM_013964782.2, XM_005679129.3, XM_018054206.1, XM_018049689.1, XM_018049709.1, XM_013968553.2, XM_005675289.3, XM_018048956.1, XM_018065817.1, XM_018059609.1, XR_001917338.1, XM_018041929.1, XM_013964769.2, XM_018042429.1, XM_018064745.1, XM_018063036.1, XR_001917332.1, NM_001314202.1, XM_018060488.1, XM_018043089.1, XM_013967227.2, XM_018048952.1, NM_001285750.1, XR_001917335.1, XM_018055908.1, XM_018049691.1, XR_001297343.2, XM_018062409.1, XM_018059799.1, XM_018044609.1, XM_005685763.3, XM_018065816.1, XM_018055186.1, XM_018054083.1, XM_018050706.1, XM_018060379.1, XM_005679294.1, XM_018064130.1, XM_005680373.3, XM_018058634.1, XM_013970271.2, XM_013968877.2, XM_005675332.3, XM_005681862.2, XM_005688564.3, XM_018060261.1, XM_018048951.1, XR_001917330.1, XM_013964774.2, XM_018064746.1, XM_005695610.2, XM_018048420.1, XM_018060377.1, XM_018057613.1, XM_005683109.3, XM_005682139.3, XM_018038791.1, XR_001917336.1, XM_013964770.2, XM_005679295.1, XM_018047738.1, XR_001297342.2, XM_018055912.1, XR_001917333.1, XM_005687533.3, XM_018051703.1, XM_018067051.1, XM_018057840.1, XM_018048550.1, XM_018061406.1, XM_018064292.1, XM_018054172.1, XM_018038788.1, XM_005688087.3, XM_018053231.1, XM_018060728.1, XR_001917334.1, XR_001917327.1, XM_018043091.1, XM_018053232.1 |
| 35 | [Breast cancer](../../../AppData/Local/Temp/Temp1_VETR-D-18-00038.zip/Cont-vs-PPRV_DEGseq_map/map05224.html) | XM_018055645.1, XM_005681861.3, XM_018055614.1, XM_018043204.1, XM_018064273.1, XM_018041850.1, XM_013964768.2, XM_018065097.1, XM_013962777.2, XM_018056451.1, XM_018056824.1, XM_005679127.2, XM_018063971.1, NM_001285748.1, XM_013970381.2, XM_018063035.1, XM_018053461.1, XM_018055911.1, XM_005683400.3, XM_005697610.3, XM_005683110.3, XM_018047989.1, XM_018055187.1, XM_018050987.1, XM_005685130.3, XM_018060378.1, XM_018051619.1, XM_018054430.1, XM_018060016.1, XM_005692453.3, XM_018048959.1, XM_018062407.1, XM_018062408.1, XM_018043792.1, XM_018053228.1, XM_018042428.1, XM_018058171.1, XM_005681590.3, XM_018043463.1, XM_013974872.2, XM_018041856.1, XM_018047231.1, XM_018053460.1, XM_018058500.1, XM_018050610.1, XR_001918229.1, XM_018056811.1, XM_018062410.1, XM_005675490.2, XM_013968554.2, XM_018051622.1, XM_005694016.2, XM_005698535.3, XM_005689267.3, XM_018058447.1, XM_005684782.3, XM_018042871.1, XM_018060376.1, XM_005681863.3, XM_018060738.1, XM_005679129.3, XM_018064279.1, XM_018053462.1, XM_018054206.1, XM_018049689.1, XM_005700145.2, XM_013968553.2, XM_005675289.3, XM_018048956.1, XM_018065817.1, XM_018049858.1, XM_005694015.2, XM_018041851.1, XM_018043794.1, XM_018043791.1, XM_018059609.1, XM_018047703.1, XM_013964769.2, XR_001295577.2, XM_018042429.1, XM_018056690.1, XM_018064745.1, XM_018041853.1, XM_018063036.1, XR_001917360.1, NM_001314202.1, XM_018060488.1, XM_018049427.1, XM_018060739.1, XM_013967227.2, XM_018048952.1, NM_001285641.1, NM_001285750.1, XM_018055908.1, XM_005701006.3, XM_018049691.1, XM_018062409.1, XM_018053966.1, XM_005686996.3, XM_018059919.1, XM_018050856.1, XM_018039227.1, XM_018041852.1, XM_005674982.3, XM_018039226.1, XM_018064276.1, XM_005685763.3, XM_005693259.3, XM_018065816.1, XM_018055186.1, XM_018054083.1, XM_018045207.1, XM_005683226.3, XM_018060379.1, XM_005680373.3, XR_001918230.1, XM_013970271.2, XM_005678307.2, XM_013968877.2, XM_005678171.3, XM_005675332.3, XM_005685146.3, XM_013971256.2, XM_018064672.1, XM_005681862.2, XM_005688564.3, XM_018060261.1, XM_018058170.1, XM_018048951.1, XM_018049857.1, XM_018058502.1, XM_013975391.2, XM_018049721.1, XM_018053831.1, XM_018044357.1, XM_018064671.1, XM_013964774.2, XM_018064746.1, XM_018050609.1, XR_001918228.1, XM_018048420.1, XM_018060377.1, XM_018038791.1, XM_018043793.1, XM_005694472.3, XM_018060089.1, XM_005676715.3, XM_018061071.1, XM_013965671.2, XM_013964770.2, XM_018056629.1, XM_018053830.1, XM_005678170.3, XM_018041854.1, XM_018061516.1, XM_018055912.1, XM_013972225.2, XM_018056628.1, XM_013972226.2, XM_018063972.1, XM_018067051.1, XM_018059918.1, XM_018064278.1, XM_018063570.1, XM_018064292.1, XM_018064277.1, XM_013968876.2, XM_018038788.1, XM_018053231.1, XM_018045593.1, XM_018043795.1, XM_018041855.1, XM_018043122.1, XM_018064274.1, XM_005685145.3, XM_005691053.3, XR_001918643.1, XM_018053232.1 |
| 36 | [Vibrio cholerae infection](../../../AppData/Local/Temp/Temp1_VETR-D-18-00038.zip/Cont-vs-PPRV_DEGseq_map/map05110.html) | XM_005687003.3, XM_018042922.1, XM_018044490.1, XM_018048309.1, XM_005684604.3, XM_018050818.1, XM_005682490.2, XM_005674937.3, XM_005695496.3, XM_018058513.1, XM_018044680.1, XM_018064235.1, XM_018061340.1, XM_018061066.1, XM_018042189.1, XM_005687462.3, XM_018057250.1, XM_005675378.3, XM_018065122.1, XR_001917681.1, XM_018048607.1, XM_018064903.1, XM_005680008.3, XM_018042190.1, XM_013970142.2, XM_018051188.1, XM_018064392.1, XM_018053664.1, XM_018042192.1, XM_018040944.1, XM_018054372.1, XM_018057254.1, XM_018053696.1, XM_018044361.1, XM_005688325.2, XM_018055656.1, XM_018055657.1, XM_018061338.1, XM_018054246.1, XM_018044991.1, XM_018044358.1, XM_018055658.1, XM_018042777.1, XM_018042766.1, XM_005675138.1, XM_018038214.1, XM_005678977.3, XM_018039811.1, XM_018042191.1, XM_018038356.1, XM_018058330.1, XM_013963623.2, XM_005687004.3, XM_018066540.1, XM_018049483.1, XM_005693960.3, XM_018042537.1, XM_005683966.3, XM_013976935.2, XM_018056069.1, XM_018050633.1, XM_018065120.1, XM_018044491.1, XM_018053902.1, XM_018039351.1, XM_018051640.1, XM_018061882.1, XM_018060882.1, XM_018053903.1, XM_013972738.2, XM_005679429.3, XM_018054838.1, XM_018053900.1, XM_018044676.1, XM_005693961.3, XM_018059819.1, XM_018059818.1, XM_018051190.1, XM_013962863.2, XM_013965080.2, XM_005686382.3, XM_018044674.1, XM_018061079.1, XM_018044360.1, XM_018049804.1, XM_018044489.1, XM_018050465.1, XM_005675144.3, XM_018046416.1, XM_018041113.1, XM_013972667.2, XM_018066268.1, XM_018048967.1, XM_018042187.1, XM_018066596.1, XM_018066740.1, XM_018044359.1, XM_005684344.3, XM_005684278.2, XM_013972159.2, XM_018052916.1, XM_018061078.1, XM_018057252.1, XR_001917682.1, XM_018039207.1, XM_018062538.1, XM_018050634.1, XM_018053899.1, XM_018042536.1, XM_018065798.1, XM_018061368.1, XM_018057247.1, XM_018061337.1, XM_018044677.1, XM_018065121.1, XM_018048308.1, XM_018059055.1, XM_018042188.1, XM_018053904.1, XM_018058511.1, XM_018044477.1, XM_018049805.1, XM_018057251.1, XM_018056093.1, XM_018057248.1, XM_018039145.1, XM_018060786.1, XM_018060827.1, XM_013972160.2, XM_018053905.1, XM_018057253.1, XM_018063629.1, XM_018056071.1, XM_018043071.1, XM_018061339.1, XM_018039786.1, XM_018062703.1, XM_018049802.1, XM_018040905.1, XM_018059654.1, XM_018039919.1, XM_018062581.1, XM_005674936.3, XM_018050466.1, XM_018058512.1, XM_018048574.1, XM_018044992.1, XM_018039789.1, XM_018062556.1, XM_018050464.1, XM_018053908.1, XM_018044362.1, XM_005679452.3, XM_005689266.3, XM_005693897.2, XM_018065123.1, NM_001285566.1, XM_005685994.3, XM_018045381.1, XM_018044675.1, XM_018059202.1, XM_018056086.1, XM_018050819.1, XR_001918846.1, XM_018039810.1, XM_018044476.1, XM_018053901.1, XM_018062580.1, XM_018057249.1, XM_005676251.3 |
| 37 | [Platelet activation](../../../AppData/Local/Temp/Temp1_VETR-D-18-00038.zip/Cont-vs-PPRV_DEGseq_map/map04611.html) | XM_018059140.1, XM_005681861.3, XM_018050499.1, XM_018059141.1, XM_018040552.1, XM_013964768.2, XM_018039549.1, XM_005679127.2, XM_018052298.1, XM_018048123.1, XM_018065644.1, XM_005682490.2, XM_018066833.1, XM_018063971.1, XM_018052301.1, XM_018042311.1, XM_005679717.3, XM_018063035.1, XM_018055911.1, XM_018047033.1, XM_018055487.1, XM_018054409.1, XM_018065643.1, XM_005697610.3, XM_018043415.1, XM_018055187.1, XR_001918100.1, XM_005680737.3, XM_018054452.1, XM_018058015.1, XM_018059139.1, XM_018045511.1, XM_018066008.1, XM_018039417.1, XM_018060378.1, XM_018051619.1, XM_018056679.1, XM_018061256.1, XM_018047856.1, XM_018057651.1, XR_001918237.1, XM_018054454.1, XM_018049795.1, XM_018060016.1, XM_018050883.1, XM_018039422.1, XR_001918973.1, XM_018039601.1, XM_018042310.1, XM_018066005.1, XM_018048959.1, XM_018038480.1, XM_018062699.1, XM_018062407.1, XM_018056162.1, XM_018062408.1, XM_018039411.1, NM_001285674.1, XM_018048124.1, XM_018053228.1, XM_018042428.1, XM_018054634.1, XM_018066832.1, XM_018066842.1, XM_018038427.1, XM_013973574.2, XM_013974872.2, XM_018039401.1, XM_018051480.1, XM_018045032.1, XM_018047231.1, XM_018064706.1, XM_005698163.3, XM_005697861.3, XM_005709386.3, XM_013964307.2, XM_018047050.1, XM_005691313.3, XM_018063551.1, XM_018054401.1, XM_018057646.1, XM_005690990.2, XM_018045751.1, XM_018042308.1, XM_018060732.1, XM_013973573.2, XM_018045287.1, XM_018067083.1, XM_018039916.1, XM_005691312.2, XM_005695833.3, XM_018061944.1, XM_018062410.1, XM_013968554.2, XM_018051622.1, XM_018046871.1, XM_018058447.1, XM_005684782.3, XM_018061941.1, XM_018060376.1, XM_018042537.1, XM_018067084.1, XM_018060566.1, XM_018043408.1, XM_005681863.3, XM_018040539.1, XM_005679129.3, XM_005687578.3, XM_018054206.1, XM_018052297.1, XM_018049689.1, XM_018046554.1, XM_018066641.1, XM_013968553.2, XM_018066481.1, XM_005675289.3, XM_018048956.1, XM_018065817.1, XM_018065782.1, XM_018065642.1, XM_018051577.1, XM_018043394.1, XR_001919508.1, XM_018047841.1, XM_018066007.1, XM_018059609.1, XM_018040536.1, XM_018054405.1, XM_013964769.2, XM_018042429.1, XM_018066002.1, XM_018042307.1, XM_018053742.1, XM_018064745.1, XM_018038426.1, XM_018054406.1, XM_018040537.1, XM_018046872.1, XM_018049271.1, XM_018052570.1, XM_018066346.1, XM_018052300.1, XM_018064036.1, XM_005675953.3, XM_018065480.1, XM_018063036.1, XM_018063966.1, XM_018054749.1, NM_001314202.1, XM_018055485.1, XM_018058975.1, XM_018060488.1, XM_018040553.1, XM_018040538.1, XM_013967227.2, XM_018048952.1, XM_018043409.1, NM_001285750.1, XM_018042765.1, XM_018057881.1, XM_018043492.1, XM_018042534.1, XM_018049881.1, XM_013972156.2, XM_018055908.1, XM_018046143.1, XM_018049691.1, XM_018062526.1, XM_005689554.3, XM_018066004.1, XM_018062409.1, XM_018065622.1, XM_018065779.1, XM_018039848.1, XM_005685763.3, XM_018063471.1, XM_018054455.1, XM_018061942.1, XM_005677636.3, XM_005684278.2, XR_001919509.1, XM_018061940.1, XM_018048127.1, XM_018065816.1, XM_018059142.1, XM_018055186.1, XM_018054083.1, XM_018045336.1, XM_018062538.1, XM_018054402.1, XM_018054420.1, XM_018054408.1, XM_005681818.3, XM_018054635.1, XM_018060379.1, XM_018048449.1, XM_018044444.1, XM_018042536.1, XM_018042309.1, XM_018066003.1, XM_018048125.1, XM_018057160.1, XM_018058573.1, XM_018049880.1, XM_018044439.1, XM_018044096.1, XM_013970271.2, XM_018054421.1, XM_018043407.1, XM_018057649.1, XM_018054403.1, XR_001919417.1, XM_018050882.1, XM_013968877.2, XM_005678171.3, XM_005675332.3, XM_018050307.1, XM_018063086.1, XM_005681862.2, XM_005688564.3, XM_018060261.1, XM_018048951.1, XM_018066009.1, XM_018061943.1, XM_018066507.1, XM_013968280.2, XM_018047031.1, XM_005685526.3, XM_018043207.1, XM_018065774.1, XM_018042971.1, XM_018055488.1, XM_013964774.2, XM_018064746.1, XM_018057647.1, XM_018048420.1, XM_018060377.1, XM_018057613.1, XM_018038791.1, XM_018043357.1, XM_018043763.1, XR_001918577.1, XM_018062527.1, XM_018066006.1, XM_018054453.1, XR_001918843.1, XM_018050008.1, XM_013964770.2, XM_013962289.2, XM_018041140.1, XM_005678170.3, XM_018059626.1, XM_018061369.1, XM_018054407.1, XM_018055912.1, XM_018065646.1, XM_018049270.1, XM_018056543.1, XM_005682575.3, XM_018041138.1, XM_018039344.1, XM_013966975.2, XM_018059143.1, XM_018067051.1, XM_018042304.1, XM_005686948.3, XM_018047840.1, XM_018042305.1, XM_018043414.1, XM_018054726.1, XM_018064292.1, XM_018055486.1, XM_018057650.1, XM_018054580.1, XM_018038788.1, XM_018055703.1, XM_018053231.1, XM_018042303.1, XM_018041139.1, XM_018050766.1, XM_018050765.1, XM_018047883.1, XM_005695832.3, NM_001314200.1, XM_018044440.1, XM_018043757.1, XM_018052573.1, XM_013962679.2, XM_018051715.1, XM_018053232.1 |
| 38 | [Nicotine addiction](../../../AppData/Local/Temp/Temp1_VETR-D-18-00038.zip/Cont-vs-PPRV_DEGseq_map/map05033.html) | XM_005684688.3, XM_005683368.3, XM_018044329.1, XM_005683367.3, XM_013965743.2, XM_005684689.3, XM_005674816.2, XM_005681573.3, XM_005681575.3, XM_018057858.1, XM_005700490.3, XM_005683260.3, XM_018066604.1, XM_005681572.3, XM_018066601.1, XM_018066603.1 |
| 39 | [HIF-1 signaling pathway](../../../AppData/Local/Temp/Temp1_VETR-D-18-00038.zip/Cont-vs-PPRV_DEGseq_map/map04066.html) | XM_005681861.3, XM_018047555.1, XM_018060112.1, XM_018045824.1, XM_013964768.2, XM_005698973.3, XM_018056451.1, XM_005679127.2, XM_018063971.1, NM_001285748.1, XM_013970381.2, XR_001295907.2, XM_018063035.1, XM_018053461.1, XM_018055911.1, XM_018058967.1, XM_005697610.3, XM_005683110.3, XM_018047989.1, XM_018055187.1, XM_018050987.1, XM_005685130.3, XM_018060378.1, XM_018051619.1, XM_005701295.3, XM_005695991.3, XM_018060016.1, XM_018062448.1, XM_018048959.1, XM_018062407.1, XM_018062408.1, XM_018058962.1, XM_018043792.1, XM_018053228.1, XM_018042428.1, XM_018043463.1, XM_013974872.2, XM_018047231.1, XM_018042596.1, XM_018042597.1, XM_018053460.1, XM_018049295.1, XM_005678224.3, XM_018062449.1, XM_018060111.1, XM_018060109.1, XM_018042326.1, XM_018050610.1, XM_018063404.1, XR_001918229.1, XM_018056956.1, XM_005695728.3, XR_001919054.1, XM_018062410.1, XM_005675490.2, XM_013968554.2, XM_018051622.1, XM_005698535.3, XM_018058447.1, XM_005684782.3, XM_005698194.3, XM_005676676.2, XM_018042871.1, XM_018060376.1, XM_005681863.3, XM_005701297.3, XM_018049688.1, XM_018060738.1, XM_005679129.3, XM_018053462.1, XM_018054206.1, XM_018049689.1, XM_018056955.1, XM_018045830.1, XM_013968553.2, XM_005675289.3, XM_018048956.1, XM_018065817.1, XM_018049858.1, XM_018043794.1, XM_018043791.1, XM_018059609.1, XM_013964769.2, XM_018042429.1, XM_018064745.1, XM_018063036.1, XM_005676678.3, XM_018051666.1, NM_001314202.1, XM_018060488.1, XM_018060739.1, XM_018048812.1, XM_013967227.2, XM_018048952.1, NM_001285641.1, NM_001285750.1, XM_018056959.1, XM_005675491.2, XM_018055908.1, XM_005701006.3, XM_018049691.1, XM_005675552.3, XM_018054976.1, XM_005675493.3, XM_018062409.1, XM_018053966.1, XM_018059919.1, XM_018058966.1, XM_018066613.1, XM_018050856.1, XM_005685763.3, XM_005693259.3, XM_018065816.1, XM_018055186.1, XM_018054083.1, XM_018060379.1, XM_013970019.2, XM_005675553.3, XM_018045834.1, XM_005689072.3, XR_001918230.1, XM_018058968.1, XM_013970271.2, XM_018038249.1, XM_005676677.3, XR_001918676.1, XM_013968877.2, XM_005678171.3, XM_005675332.3, XM_005685146.3, XM_018064672.1, XM_005681862.2, XM_005688564.3, XM_018060261.1, XM_018048951.1, XM_018049857.1, XM_018060110.1, XM_018067021.1, XM_018049721.1, XR_001295477.2, XM_018053831.1, XM_018064671.1, XM_013964774.2, XM_018058965.1, XM_018064746.1, XM_018050609.1, XM_005695224.3, XR_001918228.1, XM_013968474.2, XM_018048420.1, XM_018060377.1, XM_018038791.1, XM_018043793.1, XM_018058970.1, XM_005694472.3, XM_018060089.1, XM_018061071.1, XM_013965671.2, XM_013964770.2, XM_018053830.1, XM_005678170.3, XM_018055912.1, XM_018063972.1, XM_018067051.1, XM_018059918.1, NM_001314158.1, XM_005680968.3, XM_018063570.1, XM_018038280.1, XM_018064292.1, XM_005701296.2, XM_013968876.2, XM_018038788.1, XM_018053231.1, XM_018043795.1, XM_018050632.1, XM_005685145.3, XR_001918643.1, XM_018053232.1 |
| 40 | [Insulin resistance](../../../AppData/Local/Temp/Temp1_VETR-D-18-00038.zip/Cont-vs-PPRV_DEGseq_map/map04931.html) | XM_018043772.1, XM_005699593.3, XM_018044652.1, XM_013973238.2, XM_018058079.1, XM_018043283.1, XM_013970632.2, XM_018043204.1, XM_013962777.2, XM_018056451.1, XM_005679127.2, XM_018052298.1, XM_018063971.1, XM_018052301.1, XM_018061046.1, XM_018058022.1, XM_018042311.1, NM_001285748.1, XR_001917497.1, XM_013970381.2, XM_018066935.1, XM_018063035.1, XM_018053461.1, XM_018055911.1, XM_018063078.1, XM_018055487.1, XM_013971629.2, XM_018043567.1, XM_005679774.3, XM_018054628.1, XM_005697610.3, XM_005683110.3, XM_018047989.1, XM_018055187.1, XM_018059746.1, XM_018058560.1, XM_018040963.1, XM_018060171.1, XM_018060378.1, XM_013970631.2, XM_005680647.3, XM_018061256.1, XM_018043280.1, XM_005686991.3, XM_005680926.2, XM_005699594.3, XM_005678693.3, XM_018060016.1, XM_018057287.1, XR_001918973.1, XM_018043282.1, XM_018042310.1, XM_018043281.1, XM_018046692.1, XM_018055948.1, XM_018062407.1, XM_018043288.1, XM_018046693.1, XM_018065501.1, XM_018062408.1, XM_018043792.1, XM_018053228.1, XM_018048437.1, XM_018043463.1, XM_018043180.1, XM_018062590.1, XR_001919014.1, XM_005685184.3, XM_013974872.2, XM_018044666.1, XM_013962523.2, XM_018047231.1, XM_018053460.1, XM_018044746.1, XM_018056386.1, XM_018043766.1, XM_018061052.1, XM_018050610.1, XM_018042308.1, XM_018044665.1, XM_018057817.1, XR_001918229.1, XM_018062410.1, XM_005675490.2, XM_013968554.2, XM_013963721.2, XM_005698535.3, XM_018058447.1, XM_005684782.3, XM_018042871.1, XM_018060376.1, XM_018046688.1, XM_018060738.1, XM_005679129.3, XM_018053462.1, XM_018061051.1, XM_018052297.1, XM_005681337.3, XM_018046689.1, XM_005681336.3, XM_013968553.2, XM_005675289.3, XM_018052574.1, XM_013976398.2, XM_018065817.1, XM_018043394.1, XM_018049858.1, XM_018055099.1, XM_018043765.1, XM_018043794.1, XM_018043791.1, XM_018059609.1, XM_018047703.1, XM_013962290.2, NM_001285647.1, XM_018042307.1, XM_018044670.1, XM_018043568.1, XM_018046694.1, XM_018044669.1, XM_018049271.1, XM_018052300.1, XM_018053881.1, XM_018063036.1, XM_018039451.1, XM_018051501.1, XM_018055485.1, XM_018060488.1, XM_018060739.1, XM_018043768.1, XM_013967227.2, XM_018044668.1, XM_018043769.1, XM_018059798.1, NM_001285641.1, NM_001285750.1, XM_018061156.1, XR_001917495.1, XM_018055908.1, XM_005701006.3, XM_018051624.1, XM_005684284.3, XM_018062409.1, XM_018053966.1, XM_013973240.2, XM_018059919.1, XM_018054332.1, XM_005674982.3, XM_005702078.3, XM_005693259.3, XM_018046691.1, XM_013970533.2, XM_018054351.1, XM_018065816.1, XM_018055186.1, XM_018054083.1, XM_018048543.1, XM_018060734.1, XM_018060733.1, XM_018045207.1, XM_018060379.1, XM_005699864.3, XM_018042309.1, XM_013962990.2, XM_005684287.3, XM_018059747.1, XM_018051760.1, XM_018058634.1, XM_018040964.1, XM_018054630.1, XR_001918230.1, XM_013970271.2, XM_005678307.2, XM_018040360.1, XM_018066076.1, XM_013968877.2, XM_018066944.1, XM_005678171.3, XM_005675332.3, XM_005688564.3, XM_018055446.1, XM_018060261.1, XM_018060862.1, XM_018062591.1, XM_018043771.1, XM_018049857.1, XM_005695428.3, XM_018049721.1, XM_018053831.1, XM_018044357.1, XM_018061050.1, XR_001918314.1, XM_018043770.1, XM_018059745.1, XM_018055488.1, XM_018044667.1, XM_013971630.2, XM_018059748.1, XM_018050609.1, XR_001918228.1, XM_018048420.1, XM_018060377.1, XM_018047462.1, XM_018054629.1, XM_018043793.1, XM_005695430.2, XM_018060089.1, XM_018041744.1, XM_018061071.1, XM_013965671.2, XM_018043284.1, XM_018043764.1, XM_018046690.1, XM_018053830.1, XM_005678170.3, XM_018053882.1, XM_018058102.1, XM_018055912.1, XM_018049270.1, XM_018043190.1, XM_018043767.1, XM_005695427.3, XM_005682905.3, XM_018063972.1, XM_018067051.1, XM_018059918.1, XM_018042304.1, XM_018066075.1, XM_018042305.1, XM_005684283.3, XM_018048933.1, XM_018063570.1, XM_005682904.3, XM_018055486.1, XM_005695429.3, XM_013968876.2, XM_018058103.1, XM_018058021.1, XM_018053231.1, XM_018042303.1, XM_018043795.1, XM_018044664.1, XM_018065502.1, XM_005695431.3, XM_018043122.1, XM_005679788.3, XM_018065973.1, XM_018043287.1, XM_018055626.1, XR_001917496.1, XR_001918643.1, XM_013962471.2, XM_018053232.1 |
| 41 | [AMPK signaling pathway](../../../AppData/Local/Temp/Temp1_VETR-D-18-00038.zip/Cont-vs-PPRV_DEGseq_map/map04152.html) | XM_018055557.1, XM_005699593.3, XM_018044652.1, XM_013973238.2, XM_018062483.1, XM_018058079.1, XM_018043283.1, XM_013970632.2, XM_018061268.1, XR_001917331.1, XM_018060112.1, NM_001285629.1, XM_005687457.3, XM_018058417.1, XM_013962541.2, XM_018056451.1, XM_005679127.2, XM_018059921.1, XM_005692424.3, XM_018063971.1, XM_018061046.1, NM_001285748.1, XR_001917497.1, XM_018038998.1, XM_013970381.2, XM_018066062.1, XM_018062732.1, XM_005698856.3, XM_018063035.1, NM_001285656.1, XM_018053461.1, XM_018055911.1, XM_018063078.1, XM_005677470.3, XM_013971629.2, XM_018054628.1, XM_005680988.3, XM_005697610.3, XM_005683110.3, XM_018047989.1, XM_018055187.1, XM_018059746.1, XM_018058560.1, XM_018040963.1, XM_018040984.1, XM_005699866.3, XM_018061397.1, XM_018060171.1, XM_018060378.1, XM_013970631.2, XM_005680647.3, XM_005701295.3, XM_005680005.2, XM_013964745.2, XM_018043280.1, XM_005686991.3, XM_005695991.3, XM_018064170.1, XR_001917329.1, XM_018053586.1, XM_005699594.3, XM_018063633.1, XM_005678693.3, XM_018060016.1, XM_018059922.1, XM_018054574.1, XM_018061265.1, XM_018050190.1, XM_018063869.1, XM_005680985.3, XM_005700559.3, XM_018043282.1, XM_018043281.1, XM_018046692.1, XM_018060045.1, XM_018062407.1, XM_018043452.1, XM_018043288.1, XM_018046693.1, XM_018065501.1, XM_018062408.1, XM_018046285.1, XM_005679901.2, XM_018043792.1, XM_018053228.1, XM_018061266.1, XM_013976482.2, XM_018043463.1, XM_018054227.1, XM_018043180.1, XM_018063867.1, XM_018062590.1, XM_018062484.1, XM_013974872.2, XM_005685955.3, XM_005677469.3, XM_018059802.1, XM_018047231.1, XM_018053460.1, XM_018044746.1, XM_005689417.3, XM_018047863.1, XM_005693625.3, XM_018047864.1, XM_018060111.1, XM_018060109.1, XM_005693840.2, XR_001917337.1, XM_005691015.3, XM_018050610.1, XM_018057817.1, XM_018039212.1, XR_001918229.1, NM_001285730.1, XM_018062445.1, XM_018056956.1, XM_018053854.1, XM_018062410.1, XR_001917328.1, XM_013976723.2, XM_018042061.1, XM_005675490.2, XM_013968554.2, NM_001285619.1, XM_005698535.3, XM_018041685.1, XM_018058447.1, XM_005684782.3, XR_001917488.1, XM_018042871.1, XM_018059800.1, XM_018060376.1, XM_018046688.1, XM_005701297.3, XM_013964782.2, XM_018060738.1, XM_005679129.3, XM_013976483.2, XM_005675537.3, XM_018053462.1, XM_013966147.2, XM_018039211.1, XM_005681337.3, XM_018049709.1, XM_018056955.1, XM_018046689.1, XM_018053855.1, XM_005681336.3, XM_018039964.1, XM_018040605.1, XM_018047859.1, XM_013968553.2, XM_005675289.3, XM_018065817.1, XM_018064174.1, XM_018043354.1, XM_018049858.1, XM_018054065.1, XM_018041686.1, XM_018043794.1, XM_018043791.1, XM_018059609.1, XR_001917338.1, XM_013962290.2, NM_001285647.1, XM_018060043.1, XM_018046694.1, XM_018054571.1, XM_018064220.1, XM_018052404.1, XM_018061267.1, XM_018063036.1, XM_018047862.1, XR_001917332.1, XM_018051501.1, XM_018053585.1, XM_018060488.1, XR_001296084.2, XM_018060739.1, XM_013967227.2, XM_018059798.1, NM_001285641.1, NM_001285750.1, XM_018056959.1, XM_013966480.2, XM_005686936.2, XR_001917335.1, XR_001917495.1, XM_018055908.1, XM_005701006.3, XM_018066065.1, XR_001297343.2, XM_018062409.1, XM_018053966.1, XM_018059799.1, XM_013973240.2, XM_018059919.1, XM_018064432.1, XM_018039642.1, XM_018058529.1, XM_018054332.1, NM_001285659.1, XM_005702078.3, XM_018066068.1, XM_018062482.1, XM_005693259.3, XM_018046691.1, XM_013970533.2, XM_005697258.3, XM_005690882.3, XM_018065816.1, XM_018055186.1, XM_018054083.1, XM_018051428.1, XR_001918207.1, XM_018060379.1, XM_013970019.2, XM_018064169.1, XM_013962990.2, XM_005687459.3, XM_018059747.1, XM_018051760.1, XM_018058634.1, XM_018040964.1, XM_018064433.1, XM_018054630.1, NM_001287569.1, XR_001918230.1, XM_013970271.2, XM_018060042.1, XM_018065740.1, XM_018040360.1, XM_005684675.3, XM_018066076.1, XM_013968877.2, XM_018064172.1, XM_018066944.1, XM_005678171.3, XM_005675332.3, XM_018066067.1, XM_005688564.3, XR_001296083.2, XM_018060261.1, XM_018043454.1, XM_005691232.3, XM_018062591.1, XM_018049857.1, XM_018060110.1, XM_018067021.1, XM_005695428.3, XM_018049721.1, XM_005677359.3, XM_018053831.1, XR_001918314.1, NM_001285594.1, XR_001917330.1, XM_005685361.3, XM_018059745.1, XM_005682572.3, XM_013971630.2, XM_018064173.1, XM_018059748.1, XM_018050609.1, NM_001287233.1, XR_001918228.1, XM_013968474.2, XM_018048420.1, XM_018060377.1, XM_018054629.1, XM_005683109.3, XM_018043793.1, XR_001917336.1, XM_005695430.2, XM_018060089.1, XM_018063868.1, XM_018061094.1, XM_018043271.1, XM_018041744.1, XM_018039704.1, XM_018044227.1, XM_018061071.1, XM_013965671.2, XM_018064168.1, XM_018043284.1, XR_001297342.2, XM_005684192.3, XM_018050781.1, XM_018046690.1, XM_018053830.1, XM_005684137.3, XM_005678170.3, XM_018058102.1, XM_018039965.1, XM_018039963.1, XM_018055912.1, XR_001917333.1, XM_018062248.1, XM_018043190.1, XM_018051108.1, XM_005695427.3, XM_005682905.3, XM_018060044.1, XM_018065741.1, XM_018056659.1, XM_018047861.1, XM_018063972.1, XM_018054313.1, XM_018067051.1, XM_018059918.1, XM_018053856.1, XM_005684138.3, XM_018066075.1, XM_005691714.3, XM_018061264.1, XM_018048933.1, NM_001314307.1, XM_018041715.1, XM_013968393.2, XM_018061406.1, XM_018063570.1, XM_005682904.3, XM_005695429.3, XR_001918206.1, XM_018066069.1, XM_005701296.2, XM_013968876.2, XM_018058103.1, XM_018053231.1, XM_018047553.1, XM_005675538.3, XM_018043795.1, XM_018065502.1, XM_005695431.3, XM_005675512.3, XR_001917334.1, XM_018049163.1, XR_001917327.1, XM_013976300.2, XM_018043287.1, XM_018053857.1, XM_018039966.1, XM_018055626.1, XM_018048748.1, XR_001917496.1, XM_018053703.1, XM_018057863.1, XR_001918643.1, XM_013962471.2, XM_018064171.1, XM_018053232.1 |
| 42 | [Phagosome](../../../AppData/Local/Temp/Temp1_VETR-D-18-00038.zip/Cont-vs-PPRV_DEGseq_map/map04145.html) | XR_310868.3, XM_005687003.3, XM_018058656.1, XM_018042922.1, XM_005677656.3, NM_001314266.1, XM_018044490.1, XM_018061294.1, XM_005679127.2, XM_018048309.1, XM_005684604.3, XM_018050818.1, XM_005682490.2, XM_005674937.3, XM_018047395.1, XM_018061293.1, XM_005695496.3, XM_018058513.1, XM_018044680.1, XM_005676159.3, XM_018064235.1, XM_018057623.1, XM_018061340.1, XM_018061066.1, XM_005695578.3, XM_018061289.1, XM_018057250.1, XM_018065122.1, XR_001917681.1, XM_018058647.1, XM_018048607.1, XM_013971383.2, XM_005677781.3, XM_018060378.1, XM_005680005.2, XM_018064903.1, XM_018060016.1, XM_018044635.1, XM_013970142.2, XM_005675968.3, XM_018043534.1, XM_018058652.1, XM_018051188.1, XM_018061295.1, XM_018064392.1, XM_018040944.1, XM_013968871.2, XM_018057254.1, XM_018053696.1, XM_018044831.1, XM_005693844.3, XM_018044361.1, XM_005682299.3, XM_018047231.1, XM_018055656.1, XM_018047393.1, XM_018042282.1, XM_018060065.1, XM_018055657.1, XM_005699064.3, XM_018051782.1, XM_005688299.3, XM_018061338.1, NM_001286443.1, XM_018061292.1, XM_005676582.3, XM_018044991.1, XM_005699065.3, XM_018044358.1, XM_018055658.1, XM_005699431.3, XM_005675138.1, XM_018038214.1, XM_018044832.1, XM_013963209.2, XM_018059034.1, XM_018039811.1, XM_018058330.1, XM_018061401.1, XM_013963623.2, XM_005687004.3, XM_018066540.1, XM_005700661.3, XM_005676789.3, XM_018049483.1, XM_018047394.1, XM_018040436.1, XM_018060376.1, XM_018042537.1, XM_005683966.3, XM_018056069.1, XM_018050633.1, XM_005679129.3, XM_018065120.1, XM_018044491.1, XM_018053902.1, XM_018042281.1, XM_018039351.1, XM_005700662.3, XM_018061882.1, XM_005691412.3, XM_018060882.1, XM_005675289.3, XM_018053903.1, XM_018046614.1, XM_018059609.1, XM_018048103.1, XM_018054838.1, XM_018053900.1, XM_018044676.1, NM_001285578.1, XM_018061288.1, XM_018059819.1, XR_001918321.1, XM_018064220.1, XM_018059818.1, XM_018051785.1, XM_005681895.2, XM_018051190.1, XM_018039605.1, XM_005696969.3, XM_013962863.2, XM_013965080.2, XM_005686382.3, XM_018044674.1, XM_018052324.1, XM_018042279.1, XM_018061079.1, XM_018044360.1, XM_013969394.2, XM_018061402.1, XM_018044489.1, XM_005681533.3, XM_005675036.2, XM_018050465.1, XM_018051784.1, XM_005675144.3, XM_018046416.1, XM_018041113.1, XM_013972667.2, NM_001286091.1, XM_018066268.1, XM_018063150.1, XM_018048967.1, XM_018046617.1, XM_018066596.1, XM_018042278.1, XM_018047277.1, XM_018066740.1, XM_018044359.1, XM_005684344.3, XM_005684278.2, XM_013972159.2, XM_018052916.1, XM_018061078.1, XM_018057252.1, XR_001917682.1, XM_018042280.1, XM_018039207.1, XM_018062538.1, XM_018047843.1, XM_018058649.1, XM_018060379.1, XM_018050634.1, XM_018046615.1, XM_018053899.1, XM_018042536.1, XM_005675970.3, XM_018065798.1, XM_005680676.3, XM_018061368.1, XM_018057247.1, XM_018061337.1, XM_018039556.1, XM_018044677.1, XM_018065121.1, XM_018048308.1, XM_018059055.1, XM_005682749.3, XM_018050940.1, XM_018053904.1, XM_013968891.2, XM_018058511.1, XM_018053230.1, XR_001919756.1, XM_018061403.1, XR_001917180.1, XM_018044477.1, XM_018057251.1, XM_005675967.3, XM_018058648.1, XM_018056093.1, XM_018057248.1, XM_013971389.2, XM_018039145.1, XM_005679831.3, XM_018060786.1, XM_018060827.1, XM_018048420.1, XM_018061291.1, XM_013972160.2, XM_018053905.1, XM_018057253.1, XM_018063629.1, XM_018060377.1, XM_018056071.1, XM_005681792.3, XM_018061339.1, XM_005682139.3, XM_018039786.1, XM_005690407.3, XM_005675966.3, XM_018047396.1, XM_018065433.1, XM_018061286.1, XM_018051783.1, XM_018066085.1, XM_018059654.1, XM_018051781.1, XM_018046613.1, XM_005679832.3, NM_001285685.1, XM_018039919.1, XM_018062581.1, XM_013974301.2, XM_005674936.3, XM_018050466.1, XM_018058512.1, XM_005691714.3, XM_005680013.2, XM_013971403.2, XM_018048574.1, XM_018044992.1, XM_018054006.1, XM_018039789.1, XM_018060186.1, XM_018062556.1, XM_018050464.1, XM_018053908.1, XM_018044362.1, XM_005679452.3, XM_005689266.3, XM_018065123.1, NM_001285566.1, XM_005696968.3, XM_005685994.3, XM_005685953.3, XM_018045381.1, XM_005684757.3, XM_018054431.1, XM_018044675.1, XM_018059202.1, XM_018056086.1, XM_018050819.1, XR_001918846.1, XM_018039810.1, XM_005675969.3, XM_013963212.2, XM_018044476.1, XM_018066086.1, XM_018053901.1, XM_018048238.1, XM_018062580.1, XM_018057249.1, XM_005676251.3, XM_018046616.1 |
| 43 | [Fatty acid biosynthesis](../../../AppData/Local/Temp/Temp1_VETR-D-18-00038.zip/Cont-vs-PPRV_DEGseq_map/map00061.html) | XM_018048869.1, XM_005698484.3, NM_001285629.1, XM_018044177.1, XM_018064170.1, XM_018042131.1, XM_018048870.1, XM_013973019.2, XM_018048871.1, XM_018058912.1, XM_018050924.1, XM_013973012.2, XM_005698717.3, XM_013973016.2, XM_018064174.1, XM_005695090.3, XM_018041881.1, XM_018041882.1, XM_018064169.1, XM_018064172.1, XM_018050725.1, XM_018064173.1, XM_005698716.3, XM_018061094.1, XM_018064168.1, XM_018045331.1, XM_005698718.3, XM_013973015.2, XM_018064171.1 |
| 44 | [Carbohydrate digestion and absorption](../../../AppData/Local/Temp/Temp1_VETR-D-18-00038.zip/Cont-vs-PPRV_DEGseq_map/map04973.html) | XM_018039549.1, XM_005679127.2, XM_018039550.1, XM_018063971.1, XM_018063035.1, XM_018055911.1, XM_005697610.3, XM_018052093.1, XM_018055187.1, XM_018062471.1, XM_018060378.1, XM_018045956.1, XM_018060016.1, XM_018062407.1, XM_018062408.1, XM_018063947.1, XM_018053228.1, XM_018063293.1, XM_013974872.2, XM_018047231.1, XM_018052092.1, XM_018042596.1, XM_018042597.1, XM_005679090.3, XM_018040571.1, XM_018042326.1, XM_018062410.1, XM_013968554.2, XM_018058447.1, XM_005684782.3, XM_018060376.1, XM_005679129.3, XM_013968553.2, XM_005675289.3, XM_018065817.1, XM_018059609.1, XM_018063036.1, XM_018060488.1, XM_013967227.2, XM_018040572.1, NM_001285750.1, XM_018055908.1, XM_018054976.1, XM_018062409.1, XM_005692304.2, XM_018065816.1, XM_018055186.1, XM_018062014.1, XM_018054083.1, XM_013973538.2, XM_018060379.1, XM_005677233.3, XM_013970271.2, XM_013968877.2, XM_005675332.3, XM_005688564.3, XM_018060261.1, XM_005695934.3, XM_005677232.3, XM_018048420.1, XM_018060377.1, XM_018057613.1, XM_018062470.1, XM_018067129.1, XM_005683799.3, XM_018055912.1, XM_018067051.1, XM_005679088.2, XM_005695932.3, XM_018053231.1, XM_018056518.1, XM_018050632.1, XM_018053232.1 |
| 45 | [ECM-receptor interaction](../../../AppData/Local/Temp/Temp1_VETR-D-18-00038.zip/Cont-vs-PPRV_DEGseq_map/map04512.html) | XM_018063649.1, XM_005678936.3, XM_018038312.1, XM_018051874.1, XM_018059140.1, XM_005676250.3, XM_018050499.1, XM_018059141.1, XM_018053479.1, XM_018054194.1, XM_018052232.1, XM_018038313.1, XM_018052664.1, XM_013975959.2, XM_018045560.1, XM_018055113.1, XM_018058815.1, XM_018048123.1, XM_018040741.1, XM_018065644.1, XM_018066833.1, XM_018054620.1, XM_018054014.1, XM_018050764.1, XM_018047033.1, XM_018050759.1, XM_018065643.1, XM_018043415.1, XM_018054452.1, XM_018047819.1, XM_018058015.1, XM_018059139.1, XR_001919753.1, XM_005674830.2, XM_005674883.3, XM_018066008.1, XM_018043411.1, XM_018039417.1, XM_005680115.3, XM_018056679.1, XM_018053480.1, XM_018055751.1, XM_018051017.1, XM_018044169.1, XM_005700226.2, XM_018053478.1, XM_018054454.1, XM_018051024.1, XM_018049795.1, XM_005693225.3, XM_018039422.1, XM_018038778.1, XM_018066005.1, XM_018062699.1, XM_018051032.1, XM_018040944.1, XM_018039411.1, XM_018048124.1, XM_018041048.1, XM_005681905.3, XM_005699260.3, XM_018056363.1, XM_018064893.1, XM_005683247.3, XM_018040727.1, XM_018041576.1, XM_018066832.1, XM_018066842.1, XM_018042967.1, XM_005688053.3, XM_018039401.1, XM_018051480.1, XM_018038561.1, XM_018050567.1, XM_018052213.1, XM_018051350.1, XM_018045691.1, XM_018058140.1, XM_018055040.1, XM_018044065.1, NM_001287573.1, XM_005682449.3, XM_005693227.3, XM_013965221.2, XM_018050266.1, XM_005709386.3, XM_013964307.2, XM_005681906.3, XM_018038558.1, XM_018063551.1, XM_018040104.1, XM_018047818.1, XM_018042470.1, XM_018040698.1, XM_018039916.1, XM_018051042.1, XM_018052661.1, XM_013963034.2, XM_018058129.1, XM_005676883.3, XM_018063991.1, XM_018063312.1, XM_018058849.1, XM_005681564.3, XM_018060566.1, XM_018043408.1, XM_005682131.3, XM_005687578.3, XM_005675868.3, XM_018046554.1, XM_005675709.3, XM_018057916.1, XM_018066481.1, XR_001919179.1, XM_018065642.1, XM_018051577.1, XM_018066007.1, XM_018057521.1, XM_018052663.1, XM_018042674.1, XM_018052870.1, XM_018066002.1, XM_018061035.1, XM_005675869.3, XM_018053742.1, XM_018052662.1, XM_018047345.1, XM_018054011.1, XM_018052932.1, XM_018052867.1, XM_018057501.1, XM_018045203.1, XM_018057246.1, XM_018065480.1, XM_018063966.1, XM_018040077.1, XM_018054749.1, XM_005681903.3, XM_018058855.1, XM_018058975.1, XM_018043409.1, XM_018057881.1, XM_018058143.1, XM_018060831.1, XM_018049881.1, XM_018045252.1, XM_005675149.3, XM_005689554.3, XM_018066004.1, XM_005676887.3, XM_018038559.1, XM_013973596.2, XM_005693222.3, XM_018063471.1, XM_018054455.1, XM_018052201.1, XM_018042567.1, XM_018041569.1, XM_005677636.3, XM_018058814.1, XM_005690840.2, XM_018048127.1, XM_005700225.3, XM_018059142.1, XM_005693223.3, XR_001918483.1, XM_018054196.1, XM_018054617.1, XM_018058852.1, XM_018054420.1, XM_018053481.1, XM_018044444.1, XM_018042468.1, XM_018056198.1, XM_018058141.1, XM_018066003.1, XM_005676249.3, XM_018048125.1, XM_018052331.1, XM_013974500.2, XM_018054013.1, XM_018049880.1, XM_018038560.1, XM_018054421.1, XM_018043407.1, XM_018065751.1, XM_018054012.1, XM_018042467.1, XM_018045506.1, XM_018052941.1, XM_018059661.1, XM_018066053.1, XM_018063310.1, XM_005678025.3, XM_018042966.1, XM_018066009.1, XM_013975194.2, XM_018066507.1, XM_018056364.1, XM_013968280.2, XM_018040712.1, XM_018047031.1, XM_018059663.1, XM_018042971.1, XM_018044170.1, XM_018056863.1, XM_018047817.1, XM_018042327.1, XM_018052826.1, XM_018057613.1, XM_018057493.1, XM_018047868.1, XM_018042469.1, XM_018043357.1, XM_018066124.1, XM_018042471.1, XM_018061896.1, XR_001918577.1, XM_018066006.1, XM_018051011.1, XM_018054453.1, XM_018050008.1, XM_018040719.1, XM_005694346.3, XM_005701390.3, XM_013962289.2, XM_005699727.3, XM_018051009.1, XM_018053042.1, XM_018059626.1, XM_018061369.1, XM_018038562.1, XM_018065646.1, XM_018053473.1, XM_018056543.1, XM_005682575.3, XM_018052339.1, XM_018045308.1, XM_018039344.1, XM_018064895.1, XM_018047816.1, XM_013966975.2, XM_018065939.1, XM_018060834.1, XM_018059143.1, XM_018052868.1, XM_018058603.1, XM_005678961.3, XM_018043414.1, XM_018041567.1, XM_005681904.3, XM_018050763.1, XM_018055703.1, XM_018044342.1, XM_018043410.1, XM_018051010.1, XM_018047091.1, XM_018050766.1, XM_018050765.1, NM_001314200.1, XM_018042492.1, XM_018063056.1, XM_018043757.1, XM_005684543.2, XM_018040704.1, XM_018055450.1 |
| 46 | [Calcium signaling pathway](../../../AppData/Local/Temp/Temp1_VETR-D-18-00038.zip/Cont-vs-PPRV_DEGseq_map/map04020.html) | XM_018062643.1, XM_005684795.2, XM_013963001.2, XM_018062722.1, XM_018061124.1, XM_018045824.1, XM_018052298.1, XM_005695088.3, XM_005680366.2, XM_018052301.1, XM_018061263.1, XM_018042311.1, XM_018055980.1, XM_018054045.1, XM_018058967.1, XM_018064437.1, XM_018053453.1, XM_018041873.1, XR_001918100.1, XM_005680737.3, XM_018062714.1, XM_005691428.2, XM_018054020.1, XM_005697075.3, XM_018058454.1, XM_005691447.3, XM_018066370.1, XM_018066401.1, XM_005682114.3, XM_013972607.2, XM_018051048.1, XM_005680364.3, XM_005684794.3, XM_005694678.3, XM_005699952.3, XM_018042310.1, XM_013972972.2, XM_018041323.1, XM_018048614.1, XM_018058962.1, XM_018062720.1, XM_005680805.2, XM_018062676.1, XR_001919534.1, XM_018058066.1, XM_018063803.1, XM_018062713.1, XM_018066400.1, XM_018063799.1, XM_018065259.1, XM_018062724.1, XM_018061125.1, XM_018055301.1, XM_018064430.1, XM_018054021.1, XM_018049335.1, XM_018062721.1, XM_005680365.2, XM_018056916.1, XM_018042308.1, XM_018056914.1, XM_018047616.1, XM_018039561.1, XM_018060141.1, XM_005694677.3, XM_018063800.1, XM_018065511.1, XM_005688518.3, XM_018041016.1, XM_005694675.3, XM_018051432.1, XM_018044743.1, XM_018047613.1, XM_018063801.1, XM_018045231.1, XM_018055439.1, XM_018043047.1, XM_018052297.1, XM_018054022.1, XM_018051049.1, XM_018045830.1, XM_013968410.2, XM_018056913.1, XM_005691420.3, XM_005679333.3, XM_018066184.1, XM_018063802.1, XM_018042789.1, XM_018064436.1, XM_018066186.1, XM_018042307.1, XM_018066371.1, XM_018051046.1, XM_018049271.1, XM_018057813.1, XM_018052300.1, XM_018051427.1, XM_005691424.3, XM_018041876.1, XM_018041370.1, XM_018065262.1, XM_018063797.1, XM_013970799.2, XM_018047636.1, XM_018051050.1, XM_018064434.1, XM_018045201.1, XM_005693569.2, XM_018043593.1, XM_018064435.1, XR_001295516.2, XM_018056910.1, XM_018058966.1, XM_018065509.1, XM_005691840.3, XM_018062710.1, XM_005694519.3, XM_018062725.1, XM_005680367.3, XM_005676485.3, XM_018056908.1, XM_005685150.3, XM_018055300.1, XM_018055663.1, XM_013964453.2, XM_005686574.3, XM_013966779.2, XM_005684793.3, XM_005678655.2, XM_018042309.1, XM_018061765.1, XM_005685503.3, XM_018045834.1, XM_018050044.1, XM_005682112.2, XM_018056911.1, XM_018058968.1, XR_001919533.1, XM_018063749.1, XM_005676315.3, XM_018041327.1, XM_013966645.2, XM_013963332.2, XM_018051409.1, XM_018065510.1, XM_018048620.1, XM_005681464.3, XR_001296033.2, XM_018041369.1, XM_018058965.1, XM_018047617.1, XR_001296712.2, XM_005691270.2, XM_018062712.1, XM_018061790.1, XM_018062718.1, XM_018055162.1, XM_018046856.1, XM_018055163.1, XM_018062723.1, XM_018058970.1, XM_018062717.1, XM_018055302.1, XM_018054044.1, XM_005676482.3, XM_013971767.1, XM_018048609.1, XM_018041325.1, XM_018062711.1, XM_018049270.1, XM_005685149.3, XM_018045239.1, XM_018058455.1, XM_018065351.1, XM_018062719.1, XM_018042304.1, XM_018042305.1, XM_005697074.3, XM_018066185.1, XM_018047638.1, XM_018065261.1, XM_005688957.3, XM_005695085.3, XM_018053454.1, XM_005675916.3, XM_005678654.3, XM_005675891.3, XM_018042303.1, XM_013976047.2, XM_018056915.1, XM_018063798.1, XM_018064429.1, XM_018055664.1, XM_018055665.1, XM_005694349.2, XM_005699951.1, XM_018047637.1, XM_018051417.1, XM_018060142.1, XM_005678304.3 |
| 47 | [Gastric acid secretion](../../../AppData/Local/Temp/Temp1_VETR-D-18-00038.zip/Cont-vs-PPRV_DEGseq_map/map04971.html) | XM_018062643.1, XM_013963001.2, XM_018058923.1, XM_018050284.1, XM_018061124.1, XM_018045824.1, XM_018050287.1, XM_018039549.1, XM_005676051.2, XM_005680366.2, XM_018054045.1, XM_018058967.1, XM_018057415.1, XR_001918100.1, XM_005680737.3, XM_005697075.3, XM_005691447.3, XM_018062471.1, XM_018066370.1, XM_013972607.2, XM_018045956.1, XM_005680364.3, XM_005699952.3, XM_018050288.1, XM_018058962.1, XM_018063293.1, XM_018061125.1, XM_018065223.1, XR_001918298.1, XM_018047749.1, XM_018065222.1, XM_005680365.2, XM_018039561.1, XM_018067221.1, XM_005688518.3, XM_018041016.1, XM_018043047.1, XM_018058927.1, XM_018045830.1, XM_005679333.3, XM_018063754.1, XM_018066371.1, XM_018057813.1, XM_005681740.3, XM_013964709.2, XM_018067218.1, XM_013970799.2, XR_001918297.1, XM_018043593.1, XR_001295516.2, XM_018058966.1, XM_005692304.2, XM_005680367.3, XM_005676485.3, XM_005685150.3, XM_005686574.3, XM_013966779.2, XM_018045834.1, XM_018049558.1, XM_005677233.3, XM_005676049.3, XM_018067222.1, XM_018058968.1, XM_005677232.3, XR_001296033.2, XM_018050283.1, XM_018058965.1, XM_018063738.1, XM_018063767.1, XM_018057613.1, XM_018058970.1, XM_018058920.1, XM_018062470.1, XM_018054044.1, XM_005676482.3, XM_018045615.1, XM_005685149.3, XM_005697074.3, XM_018056518.1, XM_013976047.2, XM_005694349.2, XM_005699951.1 |
| 48 | [MAPK signaling pathway](../../../AppData/Local/Temp/Temp1_VETR-D-18-00038.zip/Cont-vs-PPRV_DEGseq_map/map04010.html) | XM_018053054.1, XM_018049505.1, XM_005681861.3, XM_018064273.1, XM_018061294.1, XM_013968498.2, XM_005675697.3, XM_013964768.2, XM_018056824.1, XM_005695798.3, XM_018052298.1, XM_018044120.1, XM_005695088.3, XM_018062382.1, XM_018052234.1, XM_018063971.1, XM_018052301.1, XM_018049986.1, XM_018042311.1, XM_018038998.1, XM_018061293.1, XM_018055980.1, XM_018063035.1, XM_018044119.1, XM_018053440.1, XM_018055911.1, XM_018054409.1, XM_005676159.3, XM_018065799.1, XM_005697610.3, NM_001314233.1, XM_018055187.1, XM_018047740.1, XM_005681777.3, XM_018050987.1, XM_005685130.3, XM_018061289.1, XM_018054459.1, XM_018066401.1, XM_018051619.1, XM_013965818.2, XM_018054460.1, NM_001314206.1, XM_018044121.1, XM_018046953.1, XM_018043534.1, XM_018042310.1, NM_001285592.1, XM_018049502.1, XM_018041323.1, XM_018061295.1, XM_018048959.1, XM_005683592.3, XM_018060045.1, XM_018062407.1, XM_018062408.1, XM_005679901.2, XM_018041780.1, XM_018053228.1, XM_005684669.3, XM_018047661.1, XM_018042428.1, XM_018057132.1, XM_005681590.3, XM_018065623.1, XM_005682995.3, XM_005695800.3, XM_005676603.3, XM_018058066.1, XM_018047186.1, XM_018066400.1, XM_018054461.1, XM_013974872.2, XM_018042146.1, XM_018051036.1, XM_013963787.2, XM_005675173.3, XM_005675698.3, XM_018053055.1, XM_018042282.1, XM_018050163.1, XM_005699064.3, XM_018058500.1, XM_018049335.1, XM_018043396.1, NM_001286443.1, XM_018061292.1, XM_013963750.2, XM_018054401.1, XM_005699065.3, XM_005695799.3, XM_005690990.2, NM_001314303.1, XM_018042308.1, XM_018060732.1, XM_018044486.1, XM_018046955.1, XM_018056811.1, XM_018049503.1, XM_018062410.1, XM_018050926.1, XM_013968554.2, XM_018051622.1, XM_005700661.3, XM_018058447.1, XM_005684782.3, XM_018040436.1, XM_018054462.1, XM_005681863.3, XM_005697801.3, XM_018064279.1, XM_005694690.3, XM_018054206.1, XM_018055439.1, XM_018064757.1, XM_018042281.1, XM_018052297.1, XM_005700662.3, XM_018049689.1, XM_005700145.2, XM_018051134.1, XM_013963310.2, XM_005691412.3, XM_013968553.2, XM_005688276.3, XM_018048956.1, XM_018065817.1, XM_013965817.2, XM_005683591.3, XM_018044122.1, XM_005684670.3, XM_018054405.1, XM_013964769.2, XM_018041428.1, XM_018042429.1, XM_018042307.1, XM_018064745.1, XM_018060043.1, XM_018054406.1, XM_018061288.1, XM_005686979.3, XM_018049271.1, XM_005675172.3, XM_018052300.1, XM_018041774.1, XM_018063036.1, NM_001314202.1, XM_018060488.1, XR_001296084.2, XM_018049427.1, XM_018052324.1, XM_018042279.1, XM_013967227.2, XM_018048952.1, NM_001285750.1, XM_018053442.1, XM_018051135.1, XM_013966480.2, XM_005681533.3, XM_018055908.1, XM_018049691.1, XM_005676108.3, XM_018052509.1, XM_005693569.2, XM_018062409.1, XM_018041436.1, XM_005676107.3, XM_005695795.3, XM_018050856.1, XM_018042278.1, XM_018064276.1, XM_018062406.1, XR_001918032.1, XM_005685763.3, XM_005694519.3, XM_005684668.3, XM_018046304.1, XM_018046846.1, XM_013963962.2, XM_005684546.3, XM_005695796.3, XM_013963311.2, XM_005692653.3, XM_018065816.1, XM_018055186.1, XM_018054083.1, XM_018042280.1, XM_013975781.2, XM_018054402.1, XM_018055663.1, XM_005683226.3, XM_005675949.3, XM_018054408.1, XM_005677146.3, XM_018042309.1, XM_005679898.3, XM_005680676.3, XM_005685503.3, XM_018041437.1, XM_018041782.1, XM_005680373.3, XM_018055419.1, XM_018041779.1, XM_013970271.2, XM_005684842.3, XM_013976510.2, XM_018060042.1, XM_018047739.1, XM_018054403.1, XM_018052233.1, XM_005684675.3, XM_013968877.2, XM_018041430.1, XM_005675332.3, XM_005685146.3, XM_018064672.1, XM_005681862.2, XM_005688564.3, XR_001296083.2, XM_018041327.1, XM_018060261.1, XR_001919756.1, XM_018046954.1, XM_018048951.1, XM_018041433.1, XM_018058502.1, XM_018042812.1, XM_005685526.3, XM_005681464.3, XM_005679158.3, XM_018049504.1, XM_018064671.1, XM_018043395.1, XM_013964774.2, XM_018064746.1, XM_005689565.3, XM_018061291.1, XM_005674841.3, XM_018038791.1, XM_005694472.3, XM_018057753.1, XM_018053441.1, XM_018049985.1, XM_018065433.1, NM_001285703.1, XM_018061286.1, XM_013964770.2, XM_018067217.1, XM_018050923.1, XM_018041325.1, XM_005676602.2, XM_018054407.1, XM_018055912.1, XM_018049270.1, XM_005695797.3, XM_013964507.2, XM_018065266.1, NM_001285685.1, XM_018046858.1, XM_018060044.1, XM_018044015.1, XM_005674769.3, XM_018067051.1, XM_018064278.1, XM_018042304.1, XM_018065720.1, XM_018042305.1, XM_005680013.2, XM_018051038.1, XM_018064292.1, XM_018046857.1, XM_005688957.3, XM_018064277.1, XM_005695085.3, XM_018038788.1, XM_018053231.1, XM_018042303.1, XM_018045593.1, XM_005685953.3, XM_018054431.1, XM_018055664.1, XM_018064274.1, XM_018055665.1, XM_005700837.3, XM_005685145.3, XM_005699502.2, XM_005679946.3, XM_005698980.3, XM_013968497.2, XM_018053232.1 |
| 49 | [Neurotrophin signaling pathway](../../../AppData/Local/Temp/Temp1_VETR-D-18-00038.zip/Cont-vs-PPRV_DEGseq_map/map04722.html) | XM_018062643.1, XM_013963001.2, XM_005681861.3, XM_018055678.1, XM_018043204.1, XM_018066843.1, XM_018064273.1, XM_018061294.1, XM_018060921.1, XM_018061124.1, XM_018045824.1, XM_013964768.2, XM_018056824.1, XM_005679127.2, XM_005680366.2, XM_018063971.1, XM_018061293.1, XM_018063035.1, XM_018054045.1, XM_018053440.1, XM_018055911.1, XM_018047978.1, XM_018058967.1, XM_005676159.3, XM_018040157.1, XM_018065799.1, XM_005697610.3, XM_018055187.1, XM_018050987.1, XM_013966112.2, XM_018065457.1, XM_005685130.3, XM_018061289.1, XM_005697075.3, XM_005691447.3, XM_018066370.1, XM_005680058.3, XM_018060378.1, XM_018051619.1, XM_018047900.1, XM_018058314.1, XM_013972607.2, XM_005680364.3, XM_018060016.1, XM_005699952.3, XM_018063090.1, XM_018043534.1, XM_018061295.1, XM_018048959.1, XM_013973350.2, XM_018062407.1, XM_018060727.1, XM_018062408.1, XM_018058962.1, XM_005679901.2, XM_018053228.1, XM_018042428.1, XM_005681590.3, XM_018065623.1, XM_013967270.2, XM_013974872.2, XM_005695611.3, XM_018061125.1, XM_018047231.1, XR_001917004.1, XM_018042282.1, XM_005680937.3, XM_005699064.3, XM_018058500.1, NM_001286443.1, XM_018061292.1, XM_005680365.2, XM_005699065.3, XM_018043092.1, XM_018050945.1, XM_018044486.1, XM_018039561.1, XM_018056811.1, XM_018062410.1, XM_013968554.2, XM_018051622.1, XM_005700661.3, XM_005688518.3, XM_018041016.1, XM_018058447.1, XM_005684782.3, XM_018040436.1, XM_018060376.1, XM_005681863.3, XM_018066563.1, XM_005679129.3, XM_018064279.1, XM_005694690.3, XM_018054206.1, XM_018043047.1, XM_018042281.1, XM_005700662.3, XM_018049689.1, XM_005700145.2, XM_018045830.1, XM_005691412.3, XM_013968553.2, XM_005675289.3, XM_018048956.1, XM_018065817.1, XM_005679333.3, XM_018059609.1, XM_018041929.1, XM_018044848.1, XM_013964769.2, XM_018042429.1, XM_018064745.1, XM_018061288.1, XM_018066371.1, XM_018057813.1, XM_018063036.1, NM_001314202.1, XM_018060488.1, XM_018043089.1, XM_018049427.1, XM_018052324.1, XM_018042279.1, XM_013967227.2, XM_018048952.1, NM_001285750.1, XM_013970799.2, XM_018053442.1, XM_005681533.3, XM_018055908.1, XM_018049691.1, XR_001917888.1, XM_018043593.1, XM_018062409.1, XR_001295516.2, XM_018058966.1, XM_018050856.1, XM_005674982.3, XM_018042278.1, XM_018064276.1, XM_018044609.1, XM_005685763.3, XM_005680367.3, XM_018065816.1, XM_018055186.1, XM_018054083.1, XM_005676485.3, XM_018042280.1, XM_018050706.1, XM_005685150.3, XM_005683226.3, XM_018063091.1, XM_005686574.3, XM_013966779.2, XM_018060379.1, XM_005690536.3, XM_005680676.3, XM_005679294.1, XM_018064130.1, XM_018045834.1, XM_005680373.3, XM_018058634.1, XM_018058968.1, XM_013970271.2, XM_005684842.3, XM_018040360.1, XM_013968877.2, XM_005675332.3, XM_005685146.3, XM_018064672.1, XM_005681862.2, XM_005688564.3, XM_018060261.1, XR_001919756.1, XM_018048951.1, XM_018058502.1, XM_018055677.1, XR_001296033.2, XM_018064671.1, XM_013964774.2, XM_018058965.1, XM_018064746.1, XM_005695610.2, XM_018048420.1, XM_018061291.1, XM_018060377.1, XM_018038791.1, XM_018058970.1, XM_005694472.3, XM_018053441.1, XM_018065433.1, XM_018054044.1, XM_018061286.1, XM_013964770.2, XM_005679295.1, XM_018047738.1, XM_005676482.3, XM_005676807.3, XM_018055912.1, XM_018057913.1, XM_005685149.3, XM_005687533.3, XM_018051703.1, XM_018065266.1, NM_001285685.1, XM_018067051.1, XM_018064278.1, XM_005697074.3, XM_005680013.2, XM_018057840.1, XM_005686045.2, XM_018048550.1, XM_018064292.1, XM_018054172.1, XM_018064277.1, XM_018038788.1, XM_005688087.3, XM_018053231.1, XM_018045593.1, XM_013976047.2, XM_005685953.3, XM_018054431.1, XM_018043122.1, XM_018060728.1, XM_018050766.1, XM_018050765.1, XM_018064274.1, XM_005685145.3, XM_005694349.2, XM_005699951.1, XM_018043091.1, XM_018053232.1 |
| 50 | [Osteoclast differentiation](../../../AppData/Local/Temp/Temp1_VETR-D-18-00038.zip/Cont-vs-PPRV_DEGseq_map/map04380.html) | XM_005681861.3, XM_018061294.1, XM_013964768.2, XM_005679127.2, XM_018052298.1, XM_005695088.3, XM_018063971.1, XM_018052301.1, XM_018042311.1, XM_018038998.1, XM_018061293.1, XM_018055980.1, XM_018063035.1, XM_018055911.1, XM_005676159.3, XM_005697610.3, XM_018055187.1, XM_018050987.1, XM_005685130.3, XM_018061289.1, XM_018060378.1, XM_018066401.1, XM_018051619.1, XM_018060016.1, XM_018043534.1, XM_018042310.1, XM_018041323.1, XM_018061295.1, XM_018048959.1, XM_018060045.1, XM_018062407.1, XM_018062408.1, XM_005679901.2, XM_018053228.1, XM_018042428.1, XM_018058066.1, XM_018066400.1, XM_013974872.2, XM_018047231.1, XM_018042282.1, XM_005699064.3, XM_018049335.1, NM_001286443.1, XM_018061292.1, XM_005699065.3, XM_018042308.1, XM_018062410.1, XM_013968554.2, XM_018051622.1, XM_005700661.3, XM_018058447.1, XM_005684782.3, XM_018040436.1, XM_018060376.1, XM_005681863.3, XM_005679129.3, XM_018054206.1, XM_018055439.1, XM_018042281.1, XM_018052297.1, XM_005700662.3, XM_018049689.1, XM_005691412.3, XM_013968553.2, XM_005675289.3, XM_018048956.1, XM_018065817.1, XM_018059609.1, XM_013964769.2, XM_018042429.1, XM_018042307.1, XM_018064745.1, XM_018060043.1, XM_018061288.1, XM_018049271.1, XM_018052300.1, XM_018063036.1, NM_001314202.1, XM_018060488.1, XR_001296084.2, XM_018052324.1, XM_018042279.1, XM_013967227.2, XM_018048952.1, NM_001285750.1, XM_013966480.2, XM_005681533.3, XM_018055908.1, XM_018049691.1, XM_005693569.2, XM_018062409.1, XM_018050856.1, XM_018042278.1, XM_005685763.3, XM_005694519.3, XM_018065816.1, XM_018055186.1, XM_018054083.1, XM_018042280.1, XM_018055663.1, XM_018060379.1, XM_018042309.1, XM_005680676.3, XM_005685503.3, XM_013970271.2, XM_018060042.1, XM_005684675.3, XM_013968877.2, XM_005675332.3, XM_005685146.3, XM_018064672.1, XM_005681862.2, XM_005688564.3, XR_001296083.2, XM_018041327.1, XM_018060261.1, XR_001919756.1, XM_018048951.1, XM_005681464.3, XM_018064671.1, XM_013964774.2, XM_018064746.1, XM_018048420.1, XM_018061291.1, XM_018060377.1, XM_018038791.1, XM_005694472.3, XM_018065433.1, XM_018061286.1, XM_013964770.2, XM_018041325.1, XM_018055912.1, XM_018049270.1, NM_001285685.1, XM_018060044.1, XM_018067051.1, XM_018042304.1, XM_018042305.1, XM_005680013.2, XM_018064292.1, XM_005688957.3, XM_005695085.3, XM_018038788.1, XM_018053231.1, XM_018042303.1, XM_005685953.3, XM_018054431.1, XM_018055664.1, XM_018055665.1, XM_005685145.3, XM_018053232.1 |
| 51 | [Morphine addiction](../../../AppData/Local/Temp/Temp1_VETR-D-18-00038.zip/Cont-vs-PPRV_DEGseq_map/map05032.html) | XM_005684795.2, XM_018039549.1, XM_005684688.3, XM_005683368.3, XM_018053453.1, XM_018044329.1, XM_005683367.3, XM_018058454.1, XM_005682114.3, XM_018051048.1, XM_005684794.3, XM_005694678.3, XM_013972972.2, XM_005683158.3, XM_018060496.1, XM_005694677.3, XM_018054208.1, XM_018065511.1, XM_005694675.3, XM_018045231.1, XM_013965743.2, XM_018051049.1, XM_018066184.1, XM_018066186.1, XM_018051046.1, XM_005684689.3, XM_005674816.2, XM_018047636.1, XM_018049891.1, XM_018051050.1, XM_018048555.1, XM_005680800.3, XM_018065509.1, XM_005681573.3, XM_013964453.2, XM_005684793.3, XM_005682112.2, XM_005681575.3, XM_018051722.1, XM_018057858.1, XM_018051721.1, XM_013966645.2, XM_013963332.2, XM_018065510.1, XM_005683159.3, XM_005700490.3, XM_005683260.3, XM_018057613.1, XM_018066604.1, XM_018047547.1, XM_005681572.3, XM_018066601.1, XM_018045239.1, XM_018058455.1, XM_018066185.1, XM_018047638.1, XM_018053454.1, XM_005675916.3, XM_005675891.3, XM_018066603.1, XM_018051719.1, XM_018047637.1, XM_005678304.3 |
| 52 | [Thyroid hormone signaling pathway](../../../AppData/Local/Temp/Temp1_VETR-D-18-00038.zip/Cont-vs-PPRV_DEGseq_map/map04919.html) | XM_005681861.3, XM_018043204.1, XM_018051353.1, XM_018060112.1, XM_013964768.2, XM_018065097.1, XM_013962541.2, XM_005679127.2, XM_005682490.2, XM_018063971.1, NM_001285748.1, XM_018063035.1, NM_001285656.1, XM_018064710.1, XM_018055911.1, XM_005697610.3, XM_018044427.1, XM_018055187.1, XM_018050987.1, XM_005685130.3, XM_018062471.1, XM_018060378.1, XM_018051619.1, XM_005701295.3, XM_005680005.2, XM_005695991.3, XM_018064703.1, XM_018045956.1, XM_018060016.1, XM_018048959.1, XM_018048614.1, XM_018062407.1, XM_018062408.1, XM_018053228.1, XM_005680805.2, XM_018042428.1, XM_018064712.1, XM_018064718.1, XM_018063293.1, XM_018065259.1, XM_013974872.2, XM_018064705.1, XM_018047231.1, XM_018064707.1, XM_018048567.1, XM_005693625.3, XM_018061210.1, XM_018060111.1, XM_018060109.1, XM_018064716.1, XM_018056916.1, XM_018056914.1, XM_018056956.1, XM_018062410.1, XM_013976723.2, XM_018048705.1, XM_005683062.3, XM_013968554.2, XM_018051622.1, XM_005694016.2, XM_018058447.1, XM_005684782.3, XM_018060376.1, XM_005681863.3, XM_005699630.3, XM_005701297.3, XM_005679129.3, XM_018054206.1, XM_018049689.1, XM_018056955.1, XM_013968553.2, XM_005675289.3, XM_018048956.1, XM_018065817.1, XM_013968410.2, XM_018056913.1, XM_005694015.2, XM_018059609.1, XM_018042789.1, XM_018057759.1, XM_013964769.2, XR_001295577.2, XM_018042429.1, XM_018064702.1, XM_018065988.1, XM_018064745.1, XM_018064711.1, XM_018064714.1, XM_018054571.1, XM_018064220.1, XM_018064715.1, XM_018063036.1, NM_001314202.1, XM_018048570.1, XM_018060488.1, XM_018041370.1, XM_018048812.1, XM_013967227.2, XM_018048952.1, NM_001285750.1, XM_018065262.1, XM_018056959.1, XM_018055908.1, XM_018049691.1, XM_018063793.1, XM_018062409.1, XM_018064713.1, XM_018048571.1, XM_018059919.1, XM_018056910.1, XM_018066613.1, XM_018050856.1, XM_005674982.3, XM_005685763.3, XM_005692304.2, XM_005684278.2, XM_018065816.1, XM_018055186.1, XM_018054083.1, XM_018056908.1, XM_018050492.1, XM_018064699.1, XM_018060379.1, XM_013970019.2, XM_018061765.1, XM_018051354.1, XM_018058634.1, XM_018060766.1, XM_005677233.3, XM_018064700.1, XM_018056911.1, XM_013970271.2, XM_018040360.1, XM_013968877.2, XM_018055852.1, XM_005675332.3, XM_005685146.3, XM_018064672.1, XM_005681862.2, XM_005688564.3, XM_005676315.3, XM_018061209.1, XM_018060261.1, XM_018048951.1, XM_018060110.1, XM_018067021.1, XM_005677232.3, XM_018048620.1, NM_001285594.1, XM_018064671.1, XM_018041369.1, XM_013964774.2, XM_018064746.1, NM_001287233.1, XM_018064708.1, XM_013968474.2, XM_018048420.1, XM_018060377.1, XM_018038791.1, XM_005694472.3, XM_018064145.1, XM_018064709.1, XM_005676715.3, XM_018062470.1, XM_018044227.1, XM_013964770.2, XM_005684192.3, XM_018050781.1, XM_018048609.1, XM_018055912.1, XM_013972225.2, XM_013972226.2, XM_018067051.1, XM_018059918.1, XM_018048572.1, XM_005691714.3, XM_018043825.1, XM_013968393.2, XM_018064292.1, XM_018065261.1, XM_018064704.1, XM_005701296.2, XM_018038788.1, XM_018053231.1, XM_018047553.1, XM_018056518.1, XM_018064701.1, XM_005695121.3, XM_018064717.1, XM_018055851.1, XM_018056915.1, XM_005675512.3, XM_018043122.1, XM_018060765.1, XM_005685145.3, XM_018048748.1, XM_018053232.1 |
| 53 | [MAPK signaling pathway - fly](../../../AppData/Local/Temp/Temp1_VETR-D-18-00038.zip/Cont-vs-PPRV_DEGseq_map/map04013.html) | XM_005681861.3, NM_001314231.1, XM_018064273.1, XM_018061294.1, XM_013964768.2, XM_018065831.1, XM_018056824.1, XM_018061960.1, XM_018039841.1, XM_018047395.1, XM_018038998.1, XM_018061293.1, XM_018055404.1, XM_005676159.3, XM_018050987.1, XM_005685130.3, XM_018061289.1, XM_018053428.1, XM_018059058.1, XM_018051619.1, XM_018065829.1, XM_005683422.3, XM_018065827.1, XM_013965777.2, XM_018061959.1, XM_005675968.3, XM_018043534.1, XM_018061295.1, XM_018048959.1, XM_018057053.1, XM_018049972.1, XM_018060045.1, XM_005683420.3, XM_005679901.2, XM_018059903.1, XM_013965781.2, XM_018042428.1, XM_005681590.3, XM_005683419.3, XM_018055405.1, XM_018046865.1, XM_018042091.1, XM_005676827.3, XM_018065834.1, XM_018047393.1, XM_018042282.1, XM_018065832.1, XM_018059902.1, XM_005699064.3, XM_018058500.1, XM_018057057.1, NM_001286443.1, XM_018051481.1, XM_018061292.1, XM_005699065.3, XM_018059059.1, XM_018047609.1, XM_018056811.1, XM_018057061.1, XM_018042061.1, XM_018051622.1, XM_005700661.3, XM_018047394.1, XM_018040436.1, XM_005681863.3, XM_018064279.1, XM_018050022.1, XM_018054206.1, XM_018042281.1, NM_001285616.1, XM_005700662.3, XM_018049689.1, XM_005700145.2, XM_005683423.3, XM_005691412.3, XM_018057051.1, XM_018048956.1, XM_018057054.1, XM_013969388.2, XM_013964769.2, XM_018042429.1, XM_018064745.1, XM_018060043.1, XM_018061288.1, NM_001314202.1, XR_001296084.2, XM_018049427.1, XM_018052324.1, XM_018042279.1, XM_018061961.1, XM_018048952.1, XM_013966480.2, XM_018057049.1, XM_005681533.3, XM_018049691.1, NM_001314204.1, XM_018050856.1, XM_018042278.1, XM_018064276.1, XM_005685763.3, XM_018042280.1, XM_005683226.3, XM_005675970.3, XM_005680701.3, XM_005680676.3, XM_005680373.3, XM_018065835.1, XM_018065828.1, XM_018060042.1, XM_018065830.1, XM_005684675.3, XM_013968891.2, XM_005685146.3, XM_018061958.1, XM_018064672.1, XM_005681862.2, XR_001296083.2, XR_001919756.1, XM_018048951.1, XM_018058502.1, XM_018042090.1, XM_013965779.2, XM_005675967.3, XM_018064671.1, XM_013964774.2, XM_018064746.1, XM_018041072.1, XM_018055403.1, XM_018044397.1, XM_018061291.1, XM_018042092.1, XM_018038791.1, XM_005694472.3, XM_005675966.3, XM_018047396.1, XM_018065433.1, XM_018061286.1, XM_018041603.1, XM_013964770.2, XM_018047615.1, XM_013965778.2, XM_005697400.2, NM_001285685.1, XM_018060044.1, XM_018064278.1, XM_018057052.1, XM_005680013.2, XM_005687901.3, XM_018042093.1, XM_018056537.1, XM_018061406.1, XM_018064292.1, XM_018057758.1, XM_018064277.1, XM_018038788.1, XM_018065836.1, XM_018045593.1, XM_005685953.3, XM_018054431.1, XM_013976725.2, XM_018064274.1, XM_018057050.1, XM_005685145.3 |
| 54 | [Ras signaling pathway](../../../AppData/Local/Temp/Temp1_VETR-D-18-00038.zip/Cont-vs-PPRV_DEGseq_map/map04014.html) | XM_005682996.3, XM_018062643.1, XM_018042709.1, XM_013963001.2, XM_005681861.3, XM_018045944.1, XM_018059317.1, NM_001285603.1, XM_018061294.1, XM_018064773.1, XM_018061124.1, XM_018050957.1, XM_013964768.2, XM_005679127.2, XM_018044120.1, XM_018066302.1, XM_018062382.1, XM_005680366.2, XM_018063971.1, XM_013972718.2, XM_018047786.1, XM_018061293.1, XM_018038403.1, XM_018063035.1, XM_018044119.1, XM_018054045.1, XM_018055911.1, XM_018038888.1, XM_018054409.1, XM_018050959.1, XM_005676159.3, XR_001917366.1, XM_018059318.1, XM_018057623.1, XM_005697610.3, XM_018055187.1, XM_018050987.1, XM_005678121.3, XM_005685130.3, XM_013966189.2, XM_018061289.1, XM_018065519.1, XM_005675231.3, XM_005697075.3, XM_005691447.3, XM_018054459.1, XM_018066370.1, XM_018060378.1, XM_018051619.1, XM_018040376.1, XM_005680005.2, XM_018054460.1, XM_013972607.2, XM_013973470.2, XM_018044121.1, XM_018044281.1, XM_005680364.3, XM_018060016.1, XM_005699952.3, XM_018045867.1, XM_018043534.1, XM_018039060.1, XM_013963692.2, XM_018061295.1, XM_018048959.1, XM_018045957.1, XM_018050958.1, XM_018062407.1, XM_018062408.1, XM_018053228.1, XM_018042428.1, XM_018044268.1, XM_005693844.3, XM_018045424.1, XM_018054461.1, XM_018047802.1, XM_018045929.1, XR_001918038.1, XM_018060858.1, XM_013974872.2, XM_018060861.1, XM_018061125.1, XM_018045952.1, XM_018047231.1, XM_013963635.2, XM_018042282.1, XM_018042711.1, XM_005699064.3, XM_018043396.1, NM_001286443.1, XM_018061292.1, XM_018060889.1, XM_005680365.2, XM_018054401.1, XM_005699065.3, XM_018040377.1, XM_005690990.2, XM_005695148.3, XM_018060732.1, XM_013972717.2, XM_005677953.3, XM_018039561.1, XM_018060496.1, XM_005694623.3, XM_018062410.1, XM_018054208.1, XM_013968554.2, XM_018051622.1, XM_005684161.3, XM_005700661.3, XM_005688518.3, XM_018041016.1, XM_018058447.1, XM_005684782.3, XM_013964590.2, XM_018040436.1, XM_018060376.1, XM_018043146.1, XM_005684160.3, XM_018054462.1, XM_018054395.1, XM_005693668.3, XM_005681863.3, XM_005679129.3, XM_018054206.1, XM_018043047.1, XM_018042281.1, XM_005684164.3, XM_005700662.3, XM_018066301.1, XM_018049689.1, XM_018045727.1, XM_018051626.1, XM_005691412.3, XM_013968553.2, XM_005675289.3, XM_018048956.1, XM_018065817.1, XM_005679333.3, XM_018044122.1, XM_018059609.1, XM_018054405.1, XM_018064771.1, XM_013964769.2, XM_018048103.1, XM_018042429.1, XM_018064745.1, XM_018054406.1, XM_018041276.1, XM_018045180.1, XM_018061288.1, XM_018066371.1, XR_001918321.1, XM_018057813.1, XM_018045726.1, XM_018039605.1, XM_018063036.1, XM_013962524.2, NM_001314202.1, XM_018060860.1, XM_018060488.1, XM_018045967.1, XM_005679810.3, XM_018052324.1, XM_018042279.1, XM_013967227.2, XM_018048952.1, NM_001285750.1, XM_013970799.2, XM_005681533.3, XM_018055908.1, XM_018065545.1, XM_018049691.1, XM_018048555.1, XM_018055810.1, XM_018043593.1, XM_018043609.1, XM_018062409.1, XM_013975176.2, XR_001295516.2, XM_018050856.1, XM_018042278.1, XM_018059316.1, XM_005685763.3, XM_018046304.1, XM_005680367.3, XM_005679431.2, XM_018065816.1, XM_018055186.1, XM_018054083.1, XM_018045955.1, XM_005676485.3, XM_018042280.1, XM_005685150.3, XM_013975781.2, XM_018054402.1, XM_013972726.2, XM_005686574.3, XM_018054408.1, XM_013966779.2, XM_018060379.1, XM_005680676.3, XM_018045980.1, XM_018055923.1, XM_005687534.3, XM_005680186.3, XM_013970271.2, XM_018059305.1, XM_005682749.3, XM_018056457.1, XM_018054403.1, XR_001917450.1, XM_018066515.1, XM_013968877.2, XM_018059315.1, XM_018063314.1, XM_005675332.3, XM_005685146.3, XM_018064672.1, XM_005681862.2, XM_005688564.3, XM_018053230.1, XM_018060261.1, XR_001919756.1, XM_013972914.2, XM_018056756.1, XM_018048951.1, XM_005678272.3, XM_005693235.3, XM_018045983.1, XM_005685526.3, XM_018038889.1, XM_013972060.2, XR_001296033.2, XM_018064671.1, XM_018043395.1, XM_013964774.2, XM_018064746.1, XM_018063313.1, XM_018064499.1, XM_018048420.1, XM_018061291.1, XM_018060377.1, XM_018066659.1, XM_018038791.1, XM_005694472.3, XM_005690371.3, XM_018066514.1, XM_018065433.1, XM_018054044.1, XM_018061286.1, XM_018042712.1, XM_013964770.2, XM_018045948.1, XM_018060859.1, XM_005675102.3, XM_005676482.3, XM_005679712.3, XM_018047547.1, XM_018054407.1, XM_018055912.1, XM_005685149.3, XM_005681310.3, XM_018039368.1, NM_001285685.1, XM_005675340.1, XM_018067051.1, XM_018038887.1, XM_018049622.1, XM_005697074.3, XM_005680013.2, XM_018045971.1, XM_018064292.1, XM_005700444.3, XM_018038788.1, XM_018053231.1, XM_018056757.1, XM_018062238.1, XM_013976047.2, XM_005685953.3, XM_018054431.1, XM_018066304.1, XM_005685145.3, XM_005694349.2, XM_005699951.1, XM_005699502.2, XM_018045974.1, XM_013964470.2, XM_018053232.1 |
| 55 | [Long-term potentiation](../../../AppData/Local/Temp/Temp1_VETR-D-18-00038.zip/Cont-vs-PPRV_DEGseq_map/map04720.html) | XM_018062643.1, XM_013963001.2, XM_005681861.3, XM_018064273.1, XM_018061124.1, XM_018045824.1, XM_013964768.2, XM_018056824.1, XM_018052298.1, XM_005695088.3, XM_005680366.2, XM_018052301.1, XM_018042311.1, XM_018055980.1, XM_018054045.1, XM_018058967.1, XM_018055487.1, XR_001918100.1, XM_018050987.1, XM_005680737.3, XM_005685130.3, XM_005697075.3, XM_005691447.3, XM_018066370.1, XM_018066401.1, XM_018051619.1, XM_018061256.1, XM_013972607.2, XM_005680364.3, XR_001918973.1, XM_005699952.3, XM_018042310.1, XM_018041323.1, XM_018048959.1, XM_018058962.1, XM_018042428.1, XM_005681590.3, XM_018058066.1, XM_018066400.1, XM_018061125.1, XM_018058500.1, XM_018049335.1, XM_005680365.2, XM_018042308.1, XM_018039561.1, XM_018056811.1, XM_018051622.1, XM_005688518.3, XM_018041016.1, XM_005681863.3, XM_018064279.1, XM_018054206.1, XM_018055439.1, XM_018043047.1, XM_018052297.1, XM_018049689.1, XM_005700145.2, XM_018045830.1, XM_018048956.1, XM_018043394.1, XM_005679333.3, XM_013964769.2, XM_018042429.1, XM_018042307.1, XM_018064745.1, XM_018066371.1, XM_018049271.1, XM_018057813.1, XM_018052300.1, NM_001314202.1, XM_018055485.1, XM_018049427.1, XM_018048812.1, XM_018048952.1, XM_013970799.2, XM_018049691.1, XM_005693569.2, XM_018043593.1, XR_001295516.2, XM_018058966.1, XM_018066613.1, XM_018050856.1, XM_018064276.1, XM_005685763.3, XM_005694519.3, XM_005680367.3, XM_005676485.3, XM_005685150.3, XM_018055663.1, XM_005683226.3, XM_005686574.3, XM_013966779.2, XM_018042309.1, XM_005685503.3, XM_018045834.1, XM_005680373.3, XM_018058968.1, XM_005685146.3, XM_018064672.1, XM_005681862.2, XM_018041327.1, XM_018048951.1, XM_018058502.1, XM_005681464.3, XR_001296033.2, XM_018064671.1, XM_018055488.1, XM_013964774.2, XM_018058965.1, XM_018064746.1, XM_018038791.1, XM_018058970.1, XM_005694472.3, XM_018054044.1, XM_013964770.2, XM_005676482.3, XM_018041325.1, XM_018049270.1, XM_005685149.3, XM_018064278.1, XM_018042304.1, XM_018042305.1, XM_005697074.3, XM_018064292.1, XM_018055486.1, XM_005688957.3, XM_018064277.1, XM_005695085.3, XM_018038788.1, XM_018042303.1, XM_018045593.1, XM_013976047.2, XM_018055664.1, XM_018064274.1, XM_018055665.1, XM_005685145.3, XM_005694349.2, XM_005699951.1 |
| 56 | [Retrograde endocannabinoid signaling](../../../AppData/Local/Temp/Temp1_VETR-D-18-00038.zip/Cont-vs-PPRV_DEGseq_map/map04723.html) | XM_005681861.3, XM_018040434.1, XM_013964768.2, XM_018039549.1, XM_013962886.2, XM_005684688.3, XM_005683368.3, XR_001918100.1, XM_005680737.3, XM_018044329.1, XM_005683367.3, XM_018051619.1, XM_013963249.2, XM_018048959.1, XM_018042428.1, XM_018043417.1, XM_005678505.1, XM_018060496.1, XM_018054208.1, XM_018051622.1, XM_005681863.3, XM_018054206.1, XM_013965743.2, XM_018049689.1, XM_005678504.3, XM_018048956.1, XM_018067058.1, XM_013964769.2, XM_018042429.1, XM_018064745.1, XM_005679103.3, XM_018067057.1, NM_001314202.1, XM_005684689.3, XM_005674816.2, XM_018048952.1, XM_018067053.1, XM_018049691.1, XM_018048555.1, XM_005685763.3, XM_005681573.3, XM_005678506.1, XM_005681575.3, XM_018067055.1, XM_018057858.1, XM_005681862.2, XM_018048951.1, XM_018043416.1, XM_013964774.2, XM_018064746.1, XM_005700490.3, XM_005683260.3, XM_018057613.1, XM_018038791.1, XM_018066604.1, XM_018067052.1, XM_013964770.2, XM_018047547.1, XM_005681572.3, XM_018066601.1, XM_018064292.1, XM_018067056.1, XM_018038788.1, XM_018066603.1 |
| 57 | [Colorectal cancer](../../../AppData/Local/Temp/Temp1_VETR-D-18-00038.zip/Cont-vs-PPRV_DEGseq_map/map05210.html) | XM_005681861.3, XM_018043204.1, XM_018064273.1, XM_018061294.1, XM_013964768.2, XM_018065097.1, XM_018056824.1, XM_005679127.2, XM_018063971.1, XM_018061293.1, XM_018063035.1, XM_018055911.1, XM_005676159.3, XM_005697610.3, XM_018055187.1, XM_018050987.1, XM_005685130.3, XM_018061289.1, XM_018060378.1, XM_018051619.1, XM_018060016.1, XM_018043534.1, XM_018061295.1, XM_018048959.1, XM_018062407.1, XM_018062408.1, XM_018053228.1, XM_018042428.1, XM_005681590.3, XM_013974872.2, XM_018047231.1, XM_018042282.1, XM_005699064.3, XM_018058500.1, NM_001286443.1, XM_018061292.1, XM_005699065.3, XM_018056811.1, XM_018062410.1, XM_013968554.2, XM_018051622.1, XM_005694016.2, XM_005700661.3, XM_018058447.1, XM_005684782.3, XM_018040436.1, XM_018060376.1, XM_005681863.3, XM_005679129.3, XM_018064279.1, XM_018054206.1, XM_018042281.1, XM_005700662.3, XM_018049689.1, XM_005700145.2, XM_005691412.3, XM_013968553.2, XM_018055204.1, XM_005675289.3, XM_018048956.1, XM_018065817.1, XM_005694015.2, XM_018059609.1, XM_013964769.2, XR_001295577.2, XM_018042429.1, XM_018064745.1, XM_005686575.3, XM_018061288.1, XM_018063036.1, NM_001314202.1, XR_001917959.1, XM_018060488.1, XM_018049427.1, XM_018052324.1, XM_018042279.1, XM_013967227.2, XM_018048952.1, NM_001285750.1, XM_005681533.3, XM_018055908.1, XM_018049691.1, XM_018062409.1, XM_018050856.1, XM_005674982.3, XM_018042278.1, XM_018064276.1, XM_005685763.3, XR_001296591.2, XM_018065816.1, XM_018055186.1, XM_018054083.1, XM_018042280.1, XM_005683226.3, XM_018060379.1, XM_005680676.3, XM_005680373.3, XM_013970271.2, XM_018050062.1, XM_013968877.2, XM_005675332.3, XM_005685146.3, XM_018064672.1, XM_005681862.2, XM_005688564.3, XM_018060261.1, XR_001919756.1, XM_018048951.1, XM_018058502.1, XM_018050063.1, XM_018064671.1, XM_013964774.2, XM_018064746.1, XM_018048420.1, XM_018061291.1, XM_018060377.1, XM_018038791.1, XM_005694472.3, XM_005676715.3, XM_018065433.1, XM_018061286.1, XM_013964770.2, XM_018055912.1, XM_013972225.2, XM_013972226.2, NM_001285685.1, XM_018067051.1, XM_018064278.1, XM_005680013.2, XM_018055203.1, XM_018064292.1, XM_018064277.1, XM_018038788.1, XM_018053231.1, XM_018045593.1, XM_005685953.3, XM_018054431.1, XM_018043122.1, XM_018064274.1, XM_005685145.3, XM_018053232.1 |
| 58 | [VEGF signaling pathway](../../../AppData/Local/Temp/Temp1_VETR-D-18-00038.zip/Cont-vs-PPRV_DEGseq_map/map04370.html) | XM_005681861.3, XM_018062722.1, XM_018061294.1, XM_013964768.2, XM_005679127.2, XM_018052298.1, XM_005695088.3, XM_018063971.1, XM_018052301.1, XM_018042311.1, XM_018061293.1, XM_018049302.1, XM_018055980.1, XM_018063035.1, XM_018055911.1, XM_018054409.1, XM_005676159.3, XM_013975225.2, XM_005697610.3, XM_018055187.1, XM_018050987.1, XM_018049782.1, XM_018062714.1, XM_005685130.3, XM_018049780.1, XM_018049301.1, XM_018061289.1, XM_018049781.1, XM_018060378.1, XM_018066401.1, XM_018051619.1, XM_018060016.1, XM_018043534.1, XM_018042310.1, XM_018041323.1, XM_018040641.1, XM_018061295.1, XM_018049787.1, XM_018048959.1, XM_018062407.1, XM_018049785.1, XM_018062408.1, XM_018062720.1, XM_018049788.1, XM_018053228.1, XM_018042428.1, XM_018058066.1, XM_018062713.1, XM_018066400.1, XM_013974872.2, XM_018062724.1, XM_018047231.1, XM_018042282.1, XM_005699064.3, XM_018049335.1, NM_001286443.1, XM_018062721.1, XM_018061292.1, XM_018054401.1, XM_005699065.3, XM_005690990.2, XM_018049300.1, XM_018042308.1, XM_018060732.1, XM_018062410.1, XM_013968554.2, XM_018051622.1, XM_005700661.3, XM_018058447.1, XM_005684782.3, XM_018040436.1, XM_018060376.1, XM_005681863.3, XM_005679129.3, XM_018054206.1, XM_018055439.1, XM_018042281.1, XM_018052297.1, XM_005700662.3, XM_018049689.1, XM_005691412.3, XM_013968553.2, XM_005675289.3, XM_018048956.1, XM_018065817.1, XM_018059609.1, XM_018054405.1, XM_013964769.2, XM_018042429.1, XM_018042307.1, XM_018064745.1, XM_018054406.1, XM_018061288.1, XM_018049271.1, XM_018052300.1, XM_018063036.1, NM_001314202.1, XM_018060488.1, XM_018052324.1, XM_018042279.1, XM_013967227.2, XM_018048952.1, NM_001285750.1, XM_005681533.3, XM_018055908.1, XM_018049691.1, XM_005693569.2, XM_018062409.1, XM_018050856.1, XM_018042278.1, XM_005685763.3, XM_018062710.1, XM_005694519.3, XM_018062725.1, XM_018065816.1, XM_018055186.1, XM_018054083.1, XM_018042280.1, XM_018054402.1, XM_018055663.1, XM_018054408.1, XM_018060379.1, XM_018042309.1, XM_005680676.3, XM_005685503.3, XM_018049783.1, XM_013970271.2, XM_018049786.1, XM_018063749.1, XM_018054403.1, XM_013968877.2, XM_005675332.3, XM_005685146.3, XM_018064672.1, XM_005681862.2, XM_005688564.3, XM_018041327.1, XM_018060261.1, XR_001919756.1, XM_018048951.1, XM_013975227.2, XM_005685526.3, XM_005681464.3, XM_018064671.1, XM_013964774.2, XM_018064746.1, XM_018062712.1, XM_018048420.1, XM_018061291.1, XM_018062718.1, XM_018060377.1, XM_018046856.1, XM_018038791.1, XM_018062723.1, XM_005694472.3, XM_018065433.1, XM_018062717.1, XM_018061286.1, XM_013964770.2, XM_018049779.1, XM_018041325.1, XM_018054407.1, XM_018055912.1, XM_018062711.1, XM_018049270.1, NM_001285685.1, XM_018067051.1, XM_018065351.1, XM_018062719.1, XM_018042304.1, XM_018042305.1, XM_005680013.2, XM_018064292.1, XM_005688957.3, XM_005695085.3, XM_018038788.1, XM_018053231.1, XM_018042303.1, XM_005685953.3, XM_018054431.1, XM_018049784.1, XM_018055664.1, XM_018055665.1, XM_005685145.3, XM_018053232.1 |
| 59 | [Mismatch repair](../../../AppData/Local/Temp/Temp1_VETR-D-18-00038.zip/Cont-vs-PPRV_DEGseq_map/map03430.html) | XM_018049024.1, XM_018047013.1, XM_018062687.1, XM_005675860.2, XM_018062685.1, XM_013976134.2, XM_018064561.1, XM_013967319.2, XM_018047011.1, XM_018058255.1, XM_018062844.1, XM_005691464.3, XM_018064262.1, XM_005675153.3, XM_018054680.1, XM_018055204.1, XM_018040823.1, XM_005686575.3, XM_005688167.3, XM_018064562.1, XM_018047012.1, XM_018058670.1, XM_005686090.3, XM_005676786.3, XM_013967318.2, XM_013967317.2, XM_005697892.3, XR_001919535.1, XM_018053961.1, XM_018050062.1, XM_018053960.1, XM_005679536.3, XM_018050063.1, XM_018054681.1, XM_018064563.1, XM_018064402.1, XM_013970611.2, XM_018055203.1, XM_013964589.2, XM_018062689.1, XM_018047010.1 |
| 60 | [Protein export](../../../AppData/Local/Temp/Temp1_VETR-D-18-00038.zip/Cont-vs-PPRV_DEGseq_map/map03060.html) | XM_018044490.1, XM_018066080.1, XM_018048309.1, XM_005684604.3, XM_018050818.1, XM_005695496.3, XM_018044680.1, XM_018053100.1, XM_018064235.1, XM_018061340.1, XM_018061066.1, XM_005695231.3, XM_018057250.1, XR_001917681.1, XM_018048607.1, XM_005685024.3, XM_018064903.1, XM_013970142.2, XM_018051188.1, XM_018064392.1, XM_018040944.1, XM_005694119.3, XM_018057254.1, XM_018053696.1, XM_018044361.1, XM_005691458.3, XM_018061338.1, XM_013973098.2, XM_018044991.1, XM_018044358.1, XM_005675138.1, XM_018038214.1, XM_018039811.1, XM_013963623.2, XM_018066540.1, XM_018049483.1, XM_018042537.1, XM_018056069.1, XM_018050633.1, XM_018044491.1, XM_018053902.1, XM_018039351.1, XM_018061882.1, XM_018060882.1, XM_018053903.1, XM_005685277.3, XM_018054838.1, XM_018053900.1, XM_018044676.1, XM_018059819.1, XM_018059818.1, XM_018051190.1, XM_013962863.2, XM_013965080.2, XM_018044674.1, XM_013964712.2, XM_018061079.1, XM_018044360.1, XM_018044489.1, XM_018050465.1, XM_005675144.3, XM_018046416.1, XM_018041113.1, XM_013972667.2, XM_018066268.1, XM_018048967.1, XM_018066596.1, XM_018066740.1, XM_018044359.1, XM_018052916.1, XM_018061078.1, XM_018057252.1, XR_001917682.1, XM_018041987.1, XM_018039207.1, XM_018062538.1, XM_018050634.1, XM_018053899.1, XM_018042536.1, XM_018065798.1, XM_018057247.1, XM_018061337.1, XM_018044677.1, XM_018048308.1, XM_018059055.1, XM_005681637.3, XM_018053904.1, XM_018044477.1, XM_018057251.1, XM_005690525.3, XM_018056093.1, XM_018057248.1, XM_018039145.1, XM_018060786.1, XM_018060827.1, XM_018053905.1, XM_018057253.1, XM_018063629.1, XM_018056071.1, XM_018049561.1, XM_018061339.1, XM_018039786.1, XM_018059654.1, XM_018039919.1, XM_018062581.1, XM_005674769.3, XM_018050466.1, XM_018048574.1, XM_018044992.1, XM_018039789.1, XM_018062556.1, XM_018050464.1, XM_018053908.1, XM_018044362.1, NM_001285566.1, XM_018045381.1, XM_018044675.1, XM_018059202.1, XM_018056086.1, XM_018050819.1, NM_001287571.1, XM_018049562.1, XM_018039810.1, XM_018039773.1, XM_018044476.1, XM_018053901.1, XM_018062580.1, XM_018057249.1, XM_005676251.3 |
| 61 | [Natural killer cell mediated cytotoxicity](../../../AppData/Local/Temp/Temp1_VETR-D-18-00038.zip/Cont-vs-PPRV_DEGseq_map/map04650.html) | XM_005681861.3, XM_018064273.1, XM_018061294.1, XM_013964768.2, XM_018056824.1, XM_005679127.2, XM_018052298.1, XM_018044120.1, XM_005695088.3, XM_018062382.1, XM_018052301.1, XM_018042311.1, XM_018061293.1, XM_018055980.1, XM_018044119.1, XM_005676159.3, XM_018050987.1, XM_005685130.3, XM_018061289.1, XM_018054459.1, XM_018060378.1, XM_018066401.1, XM_018051619.1, XM_018054460.1, XM_018044121.1, XM_018060016.1, XM_018043534.1, XM_018042310.1, XM_018041323.1, XM_018061295.1, XM_018048959.1, XM_018042428.1, XM_005681590.3, XM_018058066.1, XM_018066400.1, XM_018054461.1, XM_018047231.1, XM_018042282.1, XM_005699064.3, XM_018058500.1, XM_018049335.1, XM_018043396.1, NM_001286443.1, XM_018061292.1, XM_005699065.3, XM_018042308.1, XM_018056811.1, XM_018051622.1, XM_005700661.3, XM_018040436.1, XM_018060376.1, XM_018054462.1, XM_005681863.3, XM_005679129.3, XM_018064279.1, XM_018054206.1, XM_018055439.1, XM_018042281.1, XM_018052297.1, XM_005700662.3, XM_018049689.1, XM_005700145.2, XM_005691412.3, XM_005675289.3, XM_018048956.1, XM_018044122.1, XM_018059609.1, XM_013964769.2, XM_018042429.1, XM_018042307.1, XM_018064745.1, XM_018061288.1, XM_018049271.1, XM_018052300.1, NM_001314202.1, XM_018049427.1, XM_018052324.1, XM_018042279.1, XM_018048952.1, XM_005681533.3, XM_018049691.1, XM_005693569.2, XM_018050856.1, XM_018042278.1, XM_018064276.1, XM_005685763.3, XM_005694519.3, XM_018046304.1, XM_018042280.1, XM_013975781.2, XM_018055663.1, XM_005683226.3, XM_018060379.1, XM_018042309.1, XM_005680676.3, XM_005685503.3, XM_005680373.3, XM_005685146.3, XM_018064672.1, XM_005681862.2, XM_018041327.1, XR_001919756.1, XM_018048951.1, XM_018058502.1, XM_005681464.3, XM_018064671.1, XM_018043395.1, XM_013964774.2, XM_018064746.1, XM_018048420.1, XM_018061291.1, XM_018060377.1, XM_018038791.1, XM_005694472.3, XM_018065433.1, XM_018061286.1, XM_013964770.2, XM_018041325.1, XM_018049270.1, NM_001285685.1, XM_018064278.1, XM_018042304.1, XM_018042305.1, XM_005680013.2, XM_018064292.1, XM_005688957.3, XM_018064277.1, XM_005695085.3, XM_018038788.1, XM_018042303.1, XM_018045593.1, XM_005685953.3, XM_018054431.1, XM_018055664.1, XM_018064274.1, XM_018055665.1, XM_005685145.3, XM_005699502.2 |
| 62 | [Longevity regulating pathway - multiple species](../../../AppData/Local/Temp/Temp1_VETR-D-18-00038.zip/Cont-vs-PPRV_DEGseq_map/map04213.html) | XM_005699593.3, XM_018044652.1, XM_013973238.2, XM_018058079.1, XM_018043283.1, XM_013970632.2, XM_013968498.2, XM_018056451.1, XM_005679127.2, XM_018063971.1, XM_018061046.1, NM_001285748.1, XR_001917497.1, XM_013970381.2, XM_018063035.1, XM_018053461.1, XM_018055911.1, XM_018063078.1, XM_013971629.2, XM_018054628.1, XM_005697610.3, NM_001314233.1, XM_005683110.3, XM_018044427.1, XM_018047989.1, XM_018055187.1, XM_018059746.1, XM_018058560.1, XM_018040963.1, XM_018060171.1, XM_018053428.1, XM_018060378.1, XM_013970631.2, XM_005680647.3, XM_018043280.1, XM_005686991.3, NM_001314206.1, XM_005699594.3, XM_018063633.1, XM_005678693.3, XM_018060016.1, XM_018043282.1, XM_018043281.1, XM_018046692.1, XM_018062407.1, XM_018043288.1, XM_018046693.1, XM_018065501.1, XM_018062408.1, XM_018043792.1, XM_018053228.1, XM_018057132.1, XM_018043463.1, XM_018043180.1, XM_005682995.3, XM_018062590.1, XM_013974872.2, XM_018047231.1, XM_018053460.1, XM_018044746.1, XM_018050610.1, NM_001314303.1, XM_018057817.1, XR_001918229.1, XM_018062410.1, XM_005683062.3, XM_005675490.2, XM_013968554.2, XM_005698535.3, XM_018058447.1, XM_005684782.3, XM_018042871.1, XM_018060376.1, XM_018046688.1, XM_018060738.1, XM_005679129.3, XM_018053462.1, XM_005681337.3, XM_018046689.1, XM_005681336.3, XM_013968553.2, XM_005675289.3, XM_018065817.1, XM_018049858.1, XM_018043794.1, XM_018043791.1, XM_018059609.1, NM_001285675.1, XM_018057759.1, XM_013962290.2, NM_001285647.1, XM_018046694.1, XM_018063036.1, XM_018051501.1, XM_018060488.1, XM_018060739.1, XM_013967227.2, XM_018059798.1, NM_001285641.1, NM_001285750.1, XR_001917495.1, XM_018055908.1, XM_005701006.3, XM_005690077.3, XM_018062409.1, XM_018053966.1, XM_013973240.2, XM_018059919.1, XM_018054332.1, XM_005702078.3, XM_005693259.3, XM_018046691.1, XM_013970533.2, XM_018065816.1, XM_018055186.1, XM_018054083.1, XM_018060379.1, XM_005677146.3, XM_013962990.2, XM_018059747.1, XM_018051760.1, XM_018040964.1, XM_018054630.1, XR_001918230.1, XM_013969731.2, XM_013970271.2, XM_018066076.1, XM_013968877.2, XM_018066944.1, XM_005678171.3, XM_005675332.3, XM_005688564.3, XM_018060261.1, XM_018062591.1, XM_018049857.1, XM_005695428.3, XM_018049721.1, XM_018053831.1, XR_001918314.1, XM_018059745.1, XM_005689565.3, XM_013971630.2, XM_018059748.1, XM_018050609.1, XM_013969732.2, XR_001918228.1, XM_018048420.1, XM_018060377.1, XM_018054629.1, XM_018043793.1, XM_005695430.2, XM_018060089.1, NM_001285703.1, XM_018041744.1, XM_018061071.1, XM_013965671.2, XM_018043284.1, XM_018046690.1, XM_018053830.1, XM_005678170.3, XM_018058102.1, XM_018055912.1, XM_018043190.1, XM_005695427.3, XM_005682905.3, XM_018063972.1, XM_005674769.3, XM_018067051.1, XM_018059918.1, XM_018066075.1, XM_018048933.1, XM_018063570.1, XM_005682904.3, XM_005695429.3, XM_013968876.2, XM_018058103.1, XM_018053231.1, XM_018043795.1, XM_018065502.1, XM_005695431.3, XM_005684610.3, XM_018043287.1, XM_018055626.1, XR_001917496.1, XR_001918643.1, XM_013962471.2, XM_013968497.2, XM_018053232.1 |
| 63 | [Adipocytokine signaling pathway](../../../AppData/Local/Temp/Temp1_VETR-D-18-00038.zip/Cont-vs-PPRV_DEGseq_map/map04920.html) | XM_005699593.3, XM_018044652.1, XM_013973238.2, XM_018058079.1, XM_018043283.1, XM_013970632.2, XM_005698484.3, XM_018061268.1, XM_018044177.1, XM_018059921.1, XM_018063971.1, XM_018061046.1, NM_001285748.1, XR_001917497.1, XM_018063035.1, XM_018055911.1, XM_018063078.1, XM_013971629.2, XM_018054628.1, XM_005697610.3, XM_018055187.1, XM_018059746.1, XM_018058560.1, XM_018040963.1, XM_018060171.1, XM_013970631.2, XM_005680647.3, XM_013964745.2, XM_018043280.1, XM_005686991.3, XM_018053586.1, XM_005699594.3, XM_005678693.3, XM_018059922.1, XM_018061265.1, XM_018043282.1, XM_018043281.1, XM_018046692.1, XM_018062407.1, XM_018043288.1, XM_018046693.1, XM_018065501.1, XM_018062408.1, XM_018046285.1, XM_018053228.1, XM_018061266.1, XM_018043180.1, XM_018062590.1, XM_013974872.2, XM_018051036.1, XM_013973019.2, XM_018044746.1, XM_018058912.1, XM_018057817.1, XM_018039212.1, NM_001285730.1, XM_018050924.1, XM_018062410.1, XM_013968554.2, XM_018058447.1, XM_005684782.3, XM_013973012.2, XM_018046688.1, XM_005698717.3, XM_013973016.2, XM_018039211.1, XM_005681337.3, XM_018046689.1, XM_005681336.3, XM_013968553.2, XM_018065817.1, XM_005695090.3, XM_013962290.2, NM_001285647.1, XM_018046694.1, XM_018061267.1, XM_018063036.1, XM_018051501.1, XM_018053585.1, XM_018060488.1, XM_013967227.2, XM_018059798.1, NM_001285750.1, XR_001917495.1, XM_018055908.1, XM_018041881.1, XM_018062409.1, XM_013973240.2, XM_018059919.1, XM_018041882.1, XM_018064432.1, XM_018054332.1, XM_005702078.3, XM_018046691.1, XM_013970533.2, XM_018065816.1, XM_018055186.1, XM_018054083.1, XR_001918207.1, XM_018045131.1, XM_013962990.2, XM_018059747.1, XM_018051760.1, XM_018040964.1, XM_018064433.1, XM_018054630.1, XM_013970271.2, XM_018066076.1, XM_013968877.2, XM_018066944.1, XM_005675332.3, XM_005688564.3, XM_018060261.1, XM_018062591.1, XM_018050725.1, XM_005695428.3, XR_001918314.1, XM_018059745.1, XM_013971630.2, XM_018059748.1, XM_018054629.1, XM_005698716.3, XM_005695430.2, XM_018041744.1, XM_018043284.1, XM_018045331.1, XM_018046690.1, XM_018058102.1, XM_018055912.1, XM_018043190.1, XM_005695427.3, XM_005682905.3, XM_005698718.3, XM_018067051.1, XM_018059918.1, XM_018066075.1, XM_018061264.1, XM_013973015.2, XM_018048933.1, NM_001314307.1, XM_018051038.1, XM_005682904.3, XM_005695429.3, XR_001918206.1, XM_018058103.1, XM_018053231.1, XM_018065502.1, XM_005695431.3, XM_018043287.1, XM_018055626.1, XR_001917496.1, XM_013962471.2, XM_018053232.1 |
| 64 | [Purine metabolism](../../../AppData/Local/Temp/Temp1_VETR-D-18-00038.zip/Cont-vs-PPRV_DEGseq_map/map00230.html) | XM_018040731.1, XM_005684795.2, XM_018041298.1, XM_018044459.1, XM_018047068.1, XM_018049587.1, XM_005689853.3, XM_018043802.1, XM_005681738.3, XR_001919837.1, XM_018042825.1, XM_018043864.1, XM_018048701.1, XM_018056270.1, XM_018066694.1, XM_013973506.2, XM_005676340.3, XM_018055368.1, XM_018044847.1, XM_018041491.1, XM_018050389.1, XM_018054373.1, XR_001917204.1, XM_013963629.2, XM_018041159.1, XM_018063638.1, XM_018043863.1, XM_018062086.1, XM_005679206.3, XM_005689869.3, XM_018047013.1, XM_018063307.1, XM_018062084.1, XM_018053453.1, XM_018050390.1, XM_005685630.3, XR_001917205.1, XM_005693911.3, XM_018066773.1, XM_018063034.1, XM_018047069.1, XM_018048699.1, XM_018040861.1, XM_018064544.1, XM_018041299.1, XM_018042832.1, XM_018056229.1, XM_018064666.1, XM_018058454.1, XM_013976637.2, XR_001919466.1, XM_013963609.2, XM_018038275.1, XM_018062478.1, XM_018047856.1, XM_005682114.3, XM_005696338.3, XM_018040066.1, XM_005686659.2, XM_018063860.1, XM_013964929.2, XM_018051498.1, XM_018051048.1, XM_005679291.3, XM_005677637.3, XM_005676339.3, XM_005684794.3, XM_018050388.1, XM_005694678.3, XM_018065185.1, XM_005695791.3, XM_013965963.2, XM_018044448.1, XM_005677745.3, XM_018039601.1, XM_013972972.2, XM_005684327.3, XM_005700965.3, XM_018058552.1, XM_018065692.1, XM_018061929.1, XM_018048566.1, XM_013975121.2, XM_018064621.1, NM_001285674.1, XM_005698420.3, XM_005683751.3, XM_018053696.1, XM_013975776.2, XM_018047011.1, XM_018047070.1, XM_018054634.1, XM_018063589.1, XM_018063955.1, XM_018058588.1, XM_018053044.1, XM_005678230.3, XM_018046659.1, XM_013963156.2, XM_018064546.1, XM_005680575.3, XM_018042531.1, XM_018039932.1, XM_018064747.1, XM_018062844.1, XR_001918377.1, XM_018064677.1, XM_013972522.2, XM_018042821.1, XM_018040332.1, XM_018042828.1, XM_018048567.1, XM_005677744.3, XM_018042101.1, XM_018065187.1, XR_001917206.1, XM_013965996.2, XM_018047050.1, XM_013965995.2, XM_018061126.1, XM_018048694.1, XM_005691313.3, XM_005683158.3, XM_018063667.1, XM_018065981.1, XM_018045751.1, XM_018059606.1, XR_001917207.1, XM_018043805.1, XM_005694677.3, XM_005691312.2, XM_018061371.1, XM_018061944.1, XM_018061752.1, XM_013963623.2, XM_018065511.1, XM_018058157.1, XM_018066540.1, XM_005698709.3, XM_005681096.2, XM_005694675.3, XM_018040334.1, XM_018062155.1, XM_018061941.1, XM_018063309.1, XM_005675184.3, XM_013963613.2, XM_018042835.1, XM_018048565.1, XM_005696194.3, XM_018048564.1, XM_018045231.1, XM_005675838.3, NM_001285624.1, XM_018038276.1, XM_018065672.1, XM_013975396.2, XM_018041163.1, XM_018045092.1, XM_018048692.1, XM_005685629.3, XM_018063965.1, XM_018051049.1, XM_018048573.1, XM_013973685.2, XM_018048568.1, XM_018042824.1, XM_018048691.1, XM_018041162.1, XM_005685177.3, XM_018039929.1, XM_005681064.3, XM_005695790.3, XM_005676341.3, XM_005698318.3, XM_018056041.1, XM_013968589.2, XM_018066184.1, XM_018065980.1, XM_018043862.1, XM_018041044.1, XM_005691599.3, XM_018044742.1, XM_018064662.1, XM_018064842.1, XM_005679292.3, XM_018066186.1, XM_018041160.1, XM_018059601.1, XM_018042834.1, XM_018048569.1, XM_018059198.1, XM_018046661.1, XM_018045494.1, XM_018046439.1, XM_018051046.1, XM_018049236.1, XM_018062158.1, XM_018064036.1, XM_018041161.1, XM_005676683.2, XM_005693646.3, XM_018065194.1, XM_018047012.1, XM_013963634.2, XM_018064802.1, XM_005678168.3, XM_018065193.1, XM_013967244.2, XM_013975774.2, XM_018056227.1, XM_018059600.1, XM_018066692.1, XM_018062152.1, XM_005682900.3, XM_018045191.1, XM_018064545.1, XM_005686919.3, XM_018047636.1, XM_018049891.1, XM_018045182.1, XM_018062151.1, XM_018064675.1, XM_018046143.1, XM_018051050.1, XM_018061370.1, XM_013965993.2, XM_005681111.3, XM_018056043.1, XM_018057659.1, XR_001918305.1, XM_005697012.2, XR_001917201.1, XM_018042823.1, XM_018053078.1, XM_018066691.1, XM_005688283.3, XM_018048698.1, XM_018048786.1, XM_018046966.1, XM_018062157.1, XM_018065622.1, XM_013973909.2, XM_005680800.3, XM_013965994.2, XM_018065509.1, XM_018040957.1, XR_001917202.1, XM_018063752.1, XM_018039930.1, XM_018066740.1, XM_018061942.1, XR_001919786.1, XM_018061940.1, XM_018063637.1, XM_005684544.3, XM_005684823.3, XM_018059605.1, XM_018039224.1, XM_018059607.1, XM_018058106.1, XM_013964453.2, XM_013975120.2, XM_018066695.1, XM_005684793.3, XM_018054635.1, XM_018047071.1, XM_018048449.1, XM_018056045.1, XM_005681112.3, XM_013963622.2, XM_018059696.1, XM_018066699.1, XM_005682112.2, XR_001917199.1, XM_018041830.1, XM_018064021.1, XM_018058573.1, XM_005681330.3, XM_005695789.3, XM_018063304.1, XM_018039497.1, XM_005690614.3, XM_018044096.1, XM_018051722.1, XM_018048693.1, XM_018059699.1, XM_005679146.1, XM_018042186.1, XM_018042837.1, XM_005693910.3, XR_001919417.1, XM_013963625.2, XM_018066690.1, XM_018056230.1, XM_018058699.1, XR_001917308.1, XR_001917203.1, XM_018059604.1, XM_018051721.1, XM_013966645.2, XM_018059000.1, XM_005686386.3, XM_018062156.1, XM_018061450.1, XM_018063859.1, XM_013963332.2, XM_018061943.1, XM_005676342.3, XM_013975399.2, XM_018063306.1, XM_005682148.2, XM_013964939.2, XM_018065510.1, XM_018042836.1, XM_005681045.3, XM_018059531.1, XM_018052951.1, XM_005683159.3, XM_013964923.2, XM_018039003.1, XM_018065932.1, XM_018059532.1, XM_013965997.2, XM_018039931.1, XM_018062611.1, XM_018045101.1, XM_005690615.3, XM_005689693.3, XM_018063305.1, XM_018040721.1, XM_018063629.1, XM_005697804.3, XM_018064547.1, XM_018059533.1, XM_005675651.3, XM_018040722.1, XM_018041832.1, XM_018064584.1, XM_018064678.1, XM_018065479.1, XM_018066698.1, XM_018059855.1, XM_005676343.3, XR_001917200.1, XM_018060818.1, XM_018048183.1, XM_005697163.3, XM_005695770.3, XM_018066696.1, XM_018042838.1, XM_018061369.1, XM_005688075.3, XM_018040960.1, XM_005689969.3, XM_018065960.1, XM_018045239.1, XM_005683795.3, XM_018051703.1, XM_018058455.1, XM_018039344.1, XM_018064622.1, XM_018041045.1, XM_018050470.1, XM_005684036.3, XM_018045638.1, XM_018041833.1, XM_013962559.2, XM_018040862.1, XM_018057566.1, XM_005690557.3, XM_005689692.3, XM_005686948.3, XM_018053643.1, XM_018066185.1, XM_018047638.1, XM_018063958.1, XM_018059603.1, XM_018059602.1, XM_018062154.1, XM_018042833.1, XM_005685212.2, XM_018051443.1, XM_018040333.1, XM_018048695.1, XM_005682215.2, XM_018053454.1, XM_018042826.1, XM_018048563.1, XM_005683761.3, XM_005675916.3, XM_018043866.1, XR_001917277.1, XM_005675891.3, XR_001296369.2, XM_013967404.2, XM_018063669.1, XM_018056226.1, XM_018041696.1, XM_018063668.1, XM_018066693.1, XM_018048700.1, XM_005676684.3, XM_018039002.1, XM_018051719.1, XM_005681043.3, XM_018043696.1, XM_018064763.1, XM_005675082.3, XM_018057906.1, XM_018066697.1, XM_005679024.3, XM_018047637.1, XM_018063954.1, XM_013976777.2, XM_018047010.1, XM_005678304.3 |
| 65 | [Bladder cancer](../../../AppData/Local/Temp/Temp1_VETR-D-18-00038.zip/Cont-vs-PPRV_DEGseq_map/map05219.html) | XM_018055645.1, XM_005681861.3, XM_018055614.1, XM_018064273.1, XM_013964768.2, XM_018056824.1, XM_018050987.1, XM_005685130.3, XM_018051619.1, XM_005692453.3, XM_018048959.1, XM_018042428.1, XM_018058171.1, XM_005681590.3, XM_018059896.1, XM_018058500.1, XM_018056811.1, XM_018051622.1, XM_005689361.3, XM_005689267.3, XM_005681863.3, XM_018064279.1, XM_018054206.1, XM_018049689.1, XM_005700145.2, XM_018048956.1, XM_013964769.2, XM_018042429.1, XM_018056690.1, XM_018064745.1, NM_001314202.1, XM_018049427.1, XM_018048952.1, XM_018049691.1, XM_005686996.3, XM_018050856.1, XM_018039227.1, XM_018039226.1, XM_018064276.1, XM_005685763.3, XM_005683226.3, XM_005680373.3, XM_018064218.1, XM_005685146.3, XM_013971256.2, XM_018064672.1, XM_005681862.2, XM_018058170.1, XM_018048951.1, XM_018058502.1, XM_018064671.1, XM_013964774.2, XM_018064746.1, XM_018038791.1, XM_005694472.3, XM_013964770.2, XM_018064278.1, XM_018061431.1, XM_018064292.1, XM_018064277.1, XM_018038788.1, XM_018045593.1, XM_018064274.1, XM_005685145.3 |
| 66 | [Longevity regulating pathway](../../../AppData/Local/Temp/Temp1_VETR-D-18-00038.zip/Cont-vs-PPRV_DEGseq_map/map04211.html) | XM_005699593.3, XM_018044652.1, XM_013973238.2, XM_018058079.1, XM_018043283.1, XM_013970632.2, XM_018061268.1, XM_013962541.2, XM_018056451.1, XM_005679127.2, XM_018059921.1, XM_018057448.1, XM_018063971.1, XM_018061046.1, NM_001285748.1, XR_001917497.1, XM_013970381.2, XM_018063035.1, NM_001285656.1, XM_018053461.1, XM_018055911.1, XM_018063078.1, XM_013971629.2, XM_018054628.1, XM_005697610.3, XM_005683110.3, XM_018047989.1, XM_018055187.1, XM_018059746.1, XM_018058560.1, XM_018040963.1, XM_018060171.1, XM_018053428.1, XM_018060378.1, XM_013970631.2, XM_005680647.3, XM_005680005.2, XM_013964745.2, XM_018043280.1, XM_005686991.3, XM_018056201.1, XM_018053586.1, XM_005699594.3, XM_018063633.1, XM_005678693.3, XM_018060016.1, XM_018059922.1, XM_018061265.1, XM_018043282.1, XM_018066234.1, XM_018043281.1, XM_018046692.1, XM_018062407.1, XM_018043288.1, XM_018046693.1, XM_018065501.1, XM_018062408.1, XM_018046285.1, XM_018043792.1, XM_018053228.1, XM_018061266.1, XM_018046051.1, XM_018043463.1, XM_018043180.1, XM_018062590.1, XM_013974872.2, XM_018047231.1, XM_018053460.1, XM_018044746.1, XM_005677644.3, XM_018049295.1, XM_005693625.3, XM_018062652.1, XM_018050610.1, XM_018057817.1, XM_018039212.1, XR_001918229.1, NM_001285730.1, XM_005695728.3, XM_018062410.1, XM_013976723.2, XM_005675490.2, XM_013968554.2, XM_005698535.3, XM_018058447.1, XM_005684782.3, XM_005698194.3, XM_005676676.2, XM_018042871.1, XM_018060376.1, XM_018046688.1, XM_018060738.1, XM_005679129.3, XM_018053462.1, XM_018039211.1, XM_005681337.3, XM_018046689.1, XM_005681336.3, XM_013968553.2, XM_005675289.3, XM_018065817.1, XM_018049858.1, XM_018043794.1, XM_018043791.1, XM_018056726.1, XM_018059609.1, XM_013962290.2, XR_001918898.1, NM_001285647.1, XM_018046694.1, XM_018054571.1, XM_018064220.1, XM_018061267.1, XM_018063036.1, XM_005676678.3, XM_018051666.1, XM_018051501.1, XM_018053585.1, XR_001919874.1, XM_018060488.1, XM_018060739.1, XM_013967227.2, XM_018059798.1, XM_005687460.3, NM_001285641.1, NM_001285750.1, XR_001917495.1, XM_018055908.1, XM_005701006.3, XM_005690077.3, XM_018062409.1, XM_018053966.1, XM_013973240.2, XM_018059919.1, XM_018064432.1, XM_018054332.1, XM_005702078.3, XM_005693259.3, XM_018046691.1, XM_013970533.2, XM_018040577.1, XM_018065816.1, XM_018055186.1, XM_018054083.1, XR_001918207.1, XM_018040576.1, XM_005677645.3, XM_018062650.1, XR_001918897.1, XM_018060379.1, XM_013962990.2, XM_018059747.1, XM_018051760.1, XM_018040964.1, XM_018064433.1, XM_018054630.1, XR_001918230.1, XM_013970271.2, XM_018056276.1, XM_018038249.1, XM_005676677.3, XM_018041348.1, XM_018066076.1, XM_013968877.2, XM_018055130.1, XM_018066944.1, XM_005678171.3, XM_005675332.3, XM_005688564.3, XM_018056275.1, XM_018060261.1, XM_018062591.1, XM_018049857.1, XM_018064014.1, XM_005695428.3, XM_018049721.1, XM_018053831.1, XR_001918314.1, NM_001285594.1, XM_018059745.1, XM_013971630.2, XM_018059748.1, XM_018050609.1, NM_001287233.1, XR_001918228.1, XM_018048420.1, XM_018060377.1, XM_018054629.1, XM_018062649.1, XM_018043793.1, XM_005695430.2, XM_018041970.1, XM_018060089.1, XM_018041744.1, XM_018044227.1, XM_018061071.1, XM_013965671.2, XM_018043284.1, XM_005684192.3, XM_018050781.1, XM_018046690.1, XM_018053830.1, XM_018062651.1, XM_005678170.3, XM_018058102.1, XM_018055912.1, XM_018043190.1, XM_005695427.3, XM_018065266.1, XM_005682905.3, XM_018063972.1, XM_018067051.1, XM_018059918.1, XM_018066075.1, XM_005691714.3, XM_018061264.1, XM_018048933.1, NM_001314307.1, XM_013968393.2, XM_018063570.1, XM_005682904.3, XM_005695429.3, XR_001918206.1, XM_013968876.2, XM_018058103.1, XM_018053231.1, XM_018047553.1, XM_018043795.1, XM_018065502.1, XM_005695431.3, XM_005675512.3, XM_005684610.3, XM_018043287.1, XM_018061434.1, XM_018055626.1, XM_018048748.1, XR_001917496.1, XM_018040575.1, XM_018064731.1, XR_001918643.1, XM_013962471.2, XM_018053232.1 |
| 67 | [Epithelial cell signaling in Helicobacter pylori infection](../../../AppData/Local/Temp/Temp1_VETR-D-18-00038.zip/Cont-vs-PPRV_DEGseq_map/map05120.html) | XM_005687003.3, XM_018042922.1, XM_018061294.1, XM_018044120.1, XM_018062382.1, XM_005674937.3, XM_018061293.1, XM_018044119.1, XM_018058513.1, XM_005676159.3, XM_018061289.1, XM_018065122.1, XM_018054459.1, XM_018054460.1, XM_018044121.1, XM_018043534.1, XM_018061295.1, XM_018054461.1, XM_018055656.1, XM_018042282.1, XM_018055657.1, XM_005699064.3, XM_018043396.1, NM_001286443.1, XM_018061292.1, XM_005699065.3, XM_018055658.1, XM_018058330.1, XM_005687004.3, XM_005700661.3, XM_018040436.1, XM_005683966.3, XM_018054462.1, XM_018065120.1, XM_018042281.1, XM_005700662.3, XM_005691412.3, XM_018044122.1, XM_018061288.1, XM_005686382.3, XM_018052324.1, XM_018042279.1, XM_005681533.3, XM_018042278.1, XM_018046304.1, XM_005684344.3, XM_013972159.2, XM_018042280.1, XM_013975781.2, XM_005680676.3, XM_018061368.1, XM_018065121.1, XM_018058511.1, XR_001919756.1, XM_018043395.1, XM_018061291.1, XM_013972160.2, XM_018065433.1, XM_018061286.1, NM_001285685.1, XM_005674936.3, XM_018058512.1, XM_005680013.2, XM_005679452.3, XM_005689266.3, XM_018065123.1, XM_005685994.3, XM_005685953.3, XM_018054431.1, XR_001918846.1, XM_005699502.2 |
| 68 | [PI3K-Akt signaling pathway](../../../AppData/Local/Temp/Temp1_VETR-D-18-00038.zip/Cont-vs-PPRV_DEGseq_map/map04151.html) | XM_018063649.1, XM_005699593.3, XM_018044652.1, XM_005678936.3, XM_013973238.2, XM_018058079.1, XM_018038312.1, XM_018051874.1, XM_018059140.1, XM_018043283.1, XM_005676250.3, XM_005681861.3, XM_018050499.1, XM_018059141.1, XM_018053479.1, XM_018055678.1, XR_001917331.1, XM_018043204.1, XM_018054194.1, XM_018061294.1, XM_018052232.1, XM_018060921.1, XM_018038313.1, XM_018052664.1, XM_013964768.2, XM_013975959.2, XM_018045560.1, XM_013962777.2, XM_013962541.2, XM_018056451.1, XM_018055113.1, XM_005679127.2, XM_018058815.1, XM_018048123.1, XM_018040741.1, XM_018065644.1, XM_005689031.3, XM_018066833.1, XM_018063971.1, XM_018061046.1, NM_001285748.1, XR_001917497.1, XM_018061293.1, XM_013970381.2, XM_018054620.1, XM_018066062.1, XM_018054014.1, XM_018063035.1, NM_001285656.1, XM_018053461.1, XM_018055911.1, XM_018063078.1, XM_018050764.1, XM_018047033.1, XM_018050759.1, XM_018065643.1, XM_005676159.3, XM_018057893.1, XM_018054628.1, XM_005697610.3, XM_018043415.1, XM_005683110.3, XM_018047989.1, XM_018055187.1, XM_018050987.1, XM_018054452.1, XM_018059746.1, XM_018047819.1, XM_018058560.1, XM_005685130.3, XM_018058015.1, XM_018059139.1, XR_001919753.1, XM_018040963.1, XM_005674830.2, XM_013972144.2, XM_018061289.1, XM_005674883.3, XM_018066008.1, XM_005699866.3, XM_018043411.1, XM_018060171.1, XM_018039417.1, XM_005680115.3, XM_018060378.1, XM_018051619.1, XM_018056679.1, XM_018053480.1, XM_005680647.3, XM_018055751.1, XM_018051017.1, XM_005680005.2, XM_018044169.1, XM_005700226.2, XM_018053478.1, XM_018043280.1, XM_005686991.3, XM_018058314.1, XM_018054454.1, XR_001917329.1, XM_018051024.1, XM_005699594.3, XM_018047426.1, XM_018049795.1, XM_018063633.1, XM_005678693.3, XM_005680266.3, XM_005693225.3, XM_018060016.1, XM_018060051.1, XM_018039422.1, XM_018050190.1, XM_018043282.1, XM_018043534.1, XM_018038778.1, XM_018066005.1, XM_018061295.1, XM_018048959.1, XM_018043281.1, XM_018062699.1, XM_018051032.1, XM_018040944.1, XM_018062407.1, XM_018043452.1, XM_018043288.1, XM_018065501.1, XM_018062408.1, XM_018039411.1, XM_018048124.1, XM_018041048.1, XM_018043792.1, XM_005681905.3, XM_005699260.3, XM_018053228.1, XM_018056363.1, XM_018042428.1, XM_018064893.1, XM_005683247.3, XM_018040727.1, XM_018043463.1, XM_018041576.1, XM_018066832.1, XM_018066842.1, XM_018042967.1, XM_018043180.1, XM_018062590.1, XM_005688053.3, XM_013974872.2, XM_018039401.1, XM_005685955.3, XM_018051480.1, XM_018038561.1, XM_018059802.1, XM_018047231.1, XM_018051036.1, XM_018050567.1, XR_001917004.1, XM_018052213.1, XM_018047293.1, XM_018053460.1, XM_018044746.1, XM_018051350.1, XM_018045691.1, XM_018042282.1, XM_018058140.1, XM_018049295.1, XM_018055040.1, XM_018044065.1, NM_001287573.1, XM_005682449.3, XM_005699064.3, XM_005693625.3, XM_005693227.3, XM_013965221.2, XM_018050266.1, XM_005709386.3, XM_013964307.2, NM_001286443.1, XM_018061292.1, XR_001917337.1, XM_005681906.3, XM_018038558.1, XM_018063551.1, XM_018040104.1, XM_005699065.3, XM_005691015.3, XM_018050610.1, XM_018047818.1, XM_018042470.1, XM_018040698.1, XM_005679654.2, XM_018057817.1, XM_018060496.1, XM_005676377.3, XR_001918229.1, XM_018039916.1, XM_013976502.2, XM_005695728.3, XM_018051042.1, XM_018052661.1, XR_001919054.1, XM_018062410.1, XM_018054208.1, XR_001917328.1, XM_013963034.2, XM_013976723.2, XM_018058129.1, XM_005676883.3, XM_018042061.1, XM_005675490.2, XM_018063991.1, XM_013968554.2, XM_018051622.1, XM_018063312.1, XM_005698535.3, XM_005700661.3, XM_018058849.1, XM_018058447.1, XM_005684782.3, XM_013963116.2, XM_005698194.3, XM_005676676.2, XM_018040436.1, XM_018042871.1, XM_018059800.1, XM_018060376.1, XM_005681564.3, XM_018060566.1, XM_018048184.1, XM_018043408.1, XM_005681863.3, XM_013964782.2, XM_018060738.1, XM_005679129.3, XM_005682131.3, XM_005675537.3, XM_018053462.1, XM_005687578.3, XM_018054206.1, XM_018065969.1, XM_018042281.1, XM_005700662.3, XM_018063773.1, XM_005681337.3, XM_005675868.3, XM_018049689.1, XM_018049709.1, XM_018046554.1, XM_005675709.3, XM_005691412.3, XM_005681336.3, XM_018057916.1, XM_013968553.2, XM_018066481.1, XM_005675289.3, XM_018048956.1, XM_018065817.1, XR_001919179.1, XM_018065642.1, XM_018051577.1, XM_018049858.1, XM_018066007.1, XM_018043794.1, XM_018043791.1, XM_018059609.1, XR_001917338.1, XM_018047703.1, XM_018057521.1, XM_013962290.2, XM_018052663.1, XM_018042674.1, XM_018052870.1, XM_013964769.2, XM_018042429.1, XM_018066002.1, XM_018061035.1, XM_018047291.1, XM_005675869.3, XM_018053742.1, XM_018064745.1, XM_018052662.1, XM_018047345.1, XM_018054011.1, XM_018061288.1, XM_018052932.1, XM_018052867.1, XM_018057501.1, XM_018054571.1, XM_018064220.1, XM_018045203.1, XM_018057246.1, XM_018047425.1, XM_018065480.1, XM_018063036.1, XM_018063966.1, XM_018040077.1, XM_018054749.1, XM_005681903.3, XM_018058855.1, XR_001917332.1, XM_005676678.3, XM_018051666.1, XM_018051501.1, NM_001314202.1, XM_018058975.1, XM_018060488.1, XM_018060739.1, XM_018052324.1, XM_018042279.1, XM_013967227.2, XM_005695562.3, XM_018059798.1, XM_018048952.1, NM_001285641.1, XM_018043409.1, NM_001285750.1, XM_018057881.1, XM_018058143.1, XM_018060831.1, XR_001917335.1, XM_005681533.3, XM_018049881.1, XR_001917495.1, XM_018055908.1, XM_005701006.3, XM_018049691.1, XM_018048555.1, XM_018045252.1, XM_018066065.1, XM_005675149.3, XM_018060052.1, XM_013972145.2, XR_001297343.2, XM_005689554.3, XR_001917888.1, XM_018066004.1, XM_018062409.1, XM_018053966.1, XM_018059799.1, XM_013973240.2, XM_018059919.1, XM_018050856.1, XM_005676887.3, XM_005674982.3, XM_018042278.1, XM_005702078.3, XM_018038559.1, XM_018066068.1, XM_013973596.2, XM_005685763.3, XM_005693259.3, XM_005693222.3, XM_018063471.1, XM_018054455.1, XM_018052201.1, XM_018042567.1, XM_013970533.2, XM_018041569.1, XM_005677636.3, XM_018058814.1, XM_005690840.2, XM_018051044.1, XM_018048127.1, XM_018065816.1, XM_005700225.3, XM_018059142.1, XM_005693223.3, XM_018055186.1, XR_001918483.1, XM_018054083.1, XM_018042280.1, XM_018045207.1, XM_018054196.1, XM_018054617.1, XM_018058852.1, XM_018054420.1, XM_018053481.1, XM_018060379.1, XM_018045131.1, XM_018044444.1, XM_018042468.1, XM_018056198.1, XM_018058141.1, XM_013963900.1, XM_005680676.3, XM_013962990.2, XM_018066003.1, XM_005676249.3, XM_018048125.1, XM_018052331.1, XM_018059747.1, XM_013974500.2, XM_018051760.1, XM_018054013.1, XM_018058634.1, XM_005689032.3, XM_018040964.1, XM_018054630.1, XM_018047709.1, XR_001918230.1, XM_018049880.1, XM_018038560.1, XM_013970271.2, XM_018054421.1, XM_018047710.1, XM_005678307.2, XM_018043407.1, XM_018065751.1, XM_018038249.1, XM_018054012.1, XM_018040360.1, XM_005677586.2, XM_005676677.3, XM_018042467.1, XM_018045506.1, XM_018066076.1, XM_013968877.2, XM_018052941.1, XM_018066944.1, XM_005678171.3, XM_005675332.3, XM_005685146.3, XM_018064672.1, XM_018066067.1, XM_005681862.2, XM_005688564.3, XM_018059661.1, XM_018060261.1, XR_001919756.1, XM_018043454.1, XM_018066053.1, XM_018062591.1, XM_018048951.1, XM_018049857.1, XM_018063310.1, XM_018055677.1, XM_005678025.3, XM_018042966.1, XM_018066009.1, XM_013975194.2, XM_018066507.1, XM_005695428.3, XM_018056364.1, XM_018049721.1, XM_013968280.2, XM_018040712.1, XM_018053831.1, XM_018047031.1, XM_018044357.1, XR_001918314.1, NM_001285594.1, XR_001917330.1, XM_018059663.1, XM_018042971.1, XM_018064671.1, XM_018059745.1, XM_013964774.2, XM_018064746.1, XM_018059748.1, XM_018050609.1, NM_001287233.1, XR_001918228.1, XM_018044170.1, XM_018056863.1, XM_018048420.1, XM_018061291.1, XM_018047817.1, XM_018042327.1, XM_018060377.1, XM_018052826.1, XM_018057613.1, XM_018054629.1, XM_005683109.3, XM_018066659.1, XM_018038791.1, XM_018057493.1, XM_018043793.1, XR_001917336.1, XM_018047868.1, XM_005694472.3, XM_018042469.1, XM_018043357.1, XM_018066124.1, XM_018042471.1, XM_005695430.2, XM_018060089.1, XM_018061896.1, XM_018065433.1, XM_018041744.1, XR_001918577.1, XM_018066006.1, XM_018061286.1, XM_018051011.1, NM_001285608.1, XM_018044227.1, XM_018061071.1, XM_013965671.2, XM_018054453.1, XM_018050008.1, XM_018043284.1, XM_013964770.2, XM_018040719.1, XM_005694346.3, XM_005701390.3, XM_018047547.1, XR_001297342.2, XM_005684192.3, XM_018050781.1, XM_013962289.2, XM_018053830.1, XM_005699727.3, XM_005677585.3, XM_018051009.1, XM_018053042.1, XM_005678170.3, XM_018059626.1, XM_018058102.1, XM_018061369.1, XM_005676807.3, XM_018055912.1, XM_018038562.1, XM_018065646.1, XM_018053473.1, XR_001917333.1, XM_018056543.1, XM_018047444.1, XM_018057913.1, XM_018043190.1, XM_018043664.1, XM_005682575.3, XM_018052339.1, XM_018045308.1, XM_005695427.3, XM_018039344.1, XM_005682905.3, NM_001285685.1, XM_018064895.1, XM_018047816.1, XM_018063972.1, XM_013966975.2, XM_018065939.1, XM_018060834.1, XM_018059143.1, XM_018067051.1, XM_018052868.1, XM_018059918.1, XM_018058603.1, XM_018047424.1, XM_018066075.1, XM_005691714.3, XM_005680013.2, XM_005678961.3, XM_018043414.1, XM_018048933.1, XM_018051038.1, XM_013968393.2, XM_018061406.1, XM_005676378.3, XM_018063570.1, XM_018064292.1, XM_018041567.1, XM_005682904.3, XM_005695429.3, XM_018066069.1, XM_005681904.3, XM_018050763.1, XM_013968876.2, XM_018038788.1, XM_018058103.1, XM_018055703.1, XM_018053231.1, XM_018047553.1, XM_018044342.1, XM_005675538.3, XM_018043410.1, XM_018043795.1, XM_018065502.1, XM_005695431.3, XM_005685953.3, XM_018051010.1, XM_018054431.1, XM_018047091.1, XM_005675512.3, XM_018043122.1, XM_018050766.1, XR_001917334.1, XM_018050765.1, XR_001917327.1, XM_005685145.3, XM_018043287.1, NM_001314200.1, XM_018042492.1, XM_018063056.1, XM_018043757.1, XM_018055626.1, XM_018048748.1, XR_001917496.1, XM_005684543.2, XM_018040704.1, XM_018055450.1, XR_001918643.1, XM_018053232.1 |
| 69 | [Aldosterone-regulated sodium reabsorption](../../../AppData/Local/Temp/Temp1_VETR-D-18-00038.zip/Cont-vs-PPRV_DEGseq_map/map04960.html) | XM_005681861.3, XM_013964768.2, XM_005679127.2, XM_018062471.1, XM_018060378.1, XM_018051619.1, XM_018045956.1, XM_018060016.1, XM_018048959.1, XM_018042428.1, XM_018063293.1, XM_018047231.1, XM_018051622.1, XM_018060376.1, XM_005681863.3, XM_005679129.3, XM_018054206.1, XM_018049689.1, XM_005675289.3, XM_018048956.1, XM_018059609.1, XM_013964769.2, XM_018042429.1, XM_018064745.1, NM_001314202.1, XM_018048952.1, XM_018049691.1, XM_005685763.3, XM_005692304.2, XM_018060379.1, XM_018058634.1, XM_005677233.3, XM_018040360.1, XM_005681862.2, XM_018048951.1, XM_005677232.3, XM_013964774.2, XM_018064746.1, XM_018048420.1, XM_018060377.1, XM_018038791.1, XM_018062470.1, XM_013964770.2, XM_018064292.1, XM_018038788.1, XM_018056518.1 |
| 70 | [Regulation of lipolysis in adipocytes](../../../AppData/Local/Temp/Temp1_VETR-D-18-00038.zip/Cont-vs-PPRV_DEGseq_map/map04923.html) | XM_018062483.1, XM_018039549.1, XM_005679127.2, XM_018063971.1, XM_018063035.1, XM_018055911.1, XM_005697610.3, XM_018055187.1, XM_018060378.1, XM_018060016.1, XM_018062407.1, XM_018062408.1, XM_018053228.1, XM_018062484.1, XM_013974872.2, XM_018047231.1, XM_018064706.1, XM_005698163.3, XM_018062410.1, XM_013968554.2, XM_018058447.1, XM_005684782.3, XM_018060376.1, XM_005679129.3, XM_013968553.2, XM_005675289.3, XM_018065817.1, XM_018067058.1, XM_018059609.1, XM_018067057.1, XM_005675953.3, XM_018063036.1, XM_018060488.1, XM_013967227.2, NM_001285750.1, XM_018067053.1, XM_018055908.1, XM_018062409.1, XM_018062482.1, XM_005690882.3, XM_018065816.1, XM_018055186.1, XM_018054083.1, XM_005681818.3, XM_018060379.1, XM_013970271.2, XM_018067055.1, XM_013968877.2, XM_005678171.3, XM_005675332.3, XM_005688564.3, XM_018060261.1, XM_018048420.1, XM_018060377.1, XM_018057613.1, XM_018067052.1, XM_018041140.1, XM_005678170.3, XM_018055912.1, XM_018041138.1, XM_018067051.1, XM_018067056.1, XM_018053231.1, XM_018041139.1, XM_018047883.1, XM_018053232.1 |
| 71 | [B cell receptor signaling pathway](../../../AppData/Local/Temp/Temp1_VETR-D-18-00038.zip/Cont-vs-PPRV_DEGseq_map/map04662.html) | XM_005681861.3, XM_018043204.1, XM_018061294.1, XM_013964768.2, XM_005679127.2, XM_018052298.1, XM_005695088.3, XM_018063971.1, XM_018052301.1, XM_018042311.1, XM_018061293.1, XM_018055980.1, XM_018063035.1, XM_018055911.1, XM_005676159.3, XM_005697610.3, XM_018055187.1, XM_018050987.1, XM_005685130.3, XM_018061289.1, XM_018060378.1, XM_018066401.1, XM_018051619.1, XM_018060016.1, XM_018043534.1, XM_018042310.1, XM_018041323.1, XM_018061295.1, XM_018048959.1, XM_018062407.1, XM_018062408.1, XM_018053228.1, XM_018042428.1, XM_018058066.1, XM_018066400.1, XM_013974872.2, XM_018047231.1, XM_018042282.1, XM_005699064.3, XM_018049335.1, NM_001286443.1, XM_018061292.1, XM_005699065.3, XM_018042308.1, XM_018062410.1, XM_013968554.2, XM_018051622.1, XM_005700661.3, XM_018058447.1, XM_005684782.3, XM_018040436.1, XM_018060376.1, XM_005681863.3, XM_005679129.3, XM_018054206.1, XM_018055439.1, XM_018042281.1, XM_018052297.1, XM_005700662.3, XM_018049689.1, XM_005691412.3, XM_013968553.2, XM_005675289.3, XM_018048956.1, XM_018065817.1, XM_018059609.1, XM_013964769.2, XM_018042429.1, XM_018042307.1, XM_018064745.1, XM_018061288.1, XM_018049271.1, XM_018052300.1, XM_018063036.1, NM_001314202.1, XM_018060488.1, XM_018052324.1, XM_018042279.1, XM_013967227.2, XM_018048952.1, NM_001285750.1, XM_005681533.3, XM_018055908.1, XM_018049691.1, XM_005693569.2, XM_018062409.1, XM_018050856.1, XM_005674982.3, XM_018042278.1, XM_005685763.3, XM_005694519.3, XM_018065816.1, XM_018055186.1, XM_018054083.1, XM_018042280.1, XM_018055663.1, XM_018060379.1, XM_018042309.1, XM_005680676.3, XM_005685503.3, XM_013970271.2, XM_013968877.2, XM_005675332.3, XM_005685146.3, XM_018064672.1, XM_005681862.2, XM_005688564.3, XM_018041327.1, XM_018060261.1, XR_001919756.1, XM_018048951.1, XM_005681464.3, XM_018064671.1, XM_013964774.2, XM_018064746.1, XM_018048420.1, XM_018061291.1, XM_018060377.1, XM_018038791.1, XM_005694472.3, XM_018065433.1, XM_018061286.1, XM_013964770.2, XM_018041325.1, XM_018055912.1, XM_018049270.1, NM_001285685.1, XM_018067051.1, XM_018042304.1, XM_018042305.1, XM_005680013.2, XM_018064292.1, XM_005688957.3, XM_005695085.3, XM_018038788.1, XM_018053231.1, XM_018042303.1, XM_005685953.3, XM_018054431.1, XM_018043122.1, XM_018055664.1, XM_018055665.1, XM_005685145.3, XM_018053232.1 |
| 72 | [RIG-I-like receptor signaling pathway](../../../AppData/Local/Temp/Temp1_VETR-D-18-00038.zip/Cont-vs-PPRV_DEGseq_map/map04622.html) | XM_018038998.1, XM_005700911.3, XM_005694694.3, XM_018060045.1, XM_005679901.2, XM_018045083.1, XM_013975908.2, XM_018045079.1, XM_018060043.1, XM_005700910.3, XM_018061571.1, XR_001296084.2, XM_018044671.1, XM_013966480.2, XM_013976584.2, XM_018045080.1, XM_018060042.1, XM_018045078.1, XM_005684675.3, XR_001296083.2, XM_018045081.1, XM_018060044.1, NM_001285762.1, XM_005684610.3 |
| 73 | [Jak-STAT signaling pathway](../../../AppData/Local/Temp/Temp1_VETR-D-18-00038.zip/Cont-vs-PPRV_DEGseq_map/map04630.html) | XM_005679127.2, XM_018063971.1, NM_001285748.1, XM_018063035.1, XM_018055911.1, XM_005697610.3, XM_018055187.1, XM_018060378.1, XM_018060016.1, XM_018062407.1, XM_018062408.1, XM_018053228.1, XM_013974872.2, XM_018047231.1, XM_018051036.1, XM_018053601.1, XM_018062410.1, XM_013968554.2, XM_018058447.1, XM_005684782.3, XM_018060376.1, XM_005679129.3, XM_013968553.2, XM_005675289.3, XM_018065817.1, XM_018059609.1, XM_018063036.1, XM_018060488.1, XM_018048812.1, XM_013967227.2, NM_001285750.1, XM_018055908.1, XM_018062409.1, XM_018059919.1, XM_018066613.1, XM_018065816.1, XM_018055186.1, XM_018054083.1, XM_018060379.1, XM_018045131.1, XM_013970271.2, XM_013968877.2, XM_005675332.3, XM_005688564.3, XM_018060261.1, XM_018048420.1, XM_018060377.1, XM_018055912.1, XM_018067051.1, XM_018059918.1, XM_018051038.1, XM_018053231.1, XM_018053232.1 |
| 74 | [Melanoma](../../../AppData/Local/Temp/Temp1_VETR-D-18-00038.zip/Cont-vs-PPRV_DEGseq_map/map05218.html) | XM_018055645.1, XM_005681861.3, XM_018055614.1, XM_018064273.1, XM_013964768.2, XM_013962777.2, XM_018056824.1, XM_005679127.2, XM_018063971.1, XM_018063035.1, XM_018055911.1, XM_005697610.3, XM_018055187.1, XM_018050987.1, XM_005685130.3, XM_018060378.1, XM_018051619.1, XM_018060016.1, XM_005692453.3, XM_018048959.1, XM_018062407.1, XM_018062408.1, XM_018053228.1, XM_018042428.1, XM_018058171.1, XM_005681590.3, XM_013974872.2, XM_018047231.1, XM_018058500.1, XM_018056811.1, XM_018062410.1, XM_013968554.2, XM_018051622.1, XM_005689267.3, XM_018058447.1, XM_005684782.3, XM_018060376.1, XM_005681863.3, XM_005679129.3, XM_018064279.1, XM_018054206.1, XM_018049689.1, XM_005700145.2, XM_013968553.2, XM_005675289.3, XM_018048956.1, XM_018065817.1, XM_018059609.1, XM_018047703.1, XM_013964769.2, XM_018042429.1, XM_018056690.1, XM_018064745.1, XM_018063036.1, NM_001314202.1, XM_018060488.1, XM_018049427.1, XM_013967227.2, XM_018048952.1, NM_001285750.1, XM_018055908.1, XM_018049691.1, XM_018062409.1, XM_005686996.3, XM_018050856.1, XM_018039227.1, XM_018039226.1, XM_018064276.1, XM_005685763.3, XM_018065816.1, XM_018055186.1, XM_018054083.1, XM_018045207.1, XM_005683226.3, XM_018060379.1, XM_005680373.3, XM_013970271.2, XM_005678307.2, XM_013968877.2, XM_005675332.3, XM_005685146.3, XM_013971256.2, XM_018064672.1, XM_005681862.2, XM_005688564.3, XM_018060261.1, XM_018058170.1, XM_018048951.1, XM_018058502.1, XM_018044357.1, XM_018064671.1, XM_013964774.2, XM_018064746.1, XM_018048420.1, XM_018060377.1, XM_018066659.1, XM_018038791.1, XM_005694472.3, XM_013964770.2, XM_018055912.1, XM_018067051.1, XM_018064278.1, XM_018064292.1, XM_018064277.1, XM_018038788.1, XM_018053231.1, XM_018045593.1, XM_018064274.1, XM_005685145.3, XM_018053232.1 |
| 75 | [Amino sugar and nucleotide sugar metabolism](../../../AppData/Local/Temp/Temp1_VETR-D-18-00038.zip/Cont-vs-PPRV_DEGseq_map/map00520.html) | XM_018063858.1, XM_018061149.1, XM_018046761.1, XM_018060028.1, XR_001919091.1, XM_018061152.1, XM_018060359.1, XM_018051238.1, XM_013968440.2, XM_018053838.1, XM_018052504.1, XM_013966145.2, XM_018063731.1, XM_005688391.2, XM_018044499.1, XM_018039337.1, NM_001285689.1, XM_018041234.1, XM_013965963.2, XM_018059807.1, XM_018055387.1, XM_018065702.1, XM_018041230.1, XM_018041073.1, XM_018057095.1, XM_018056267.1, XM_005691157.3, XM_018041235.1, XM_005685091.3, XM_018061153.1, XM_018049341.1, XM_018046277.1, XM_005676593.3, XM_018041238.1, XM_013974851.2, XM_018041240.1, XM_018058334.1, XM_018055602.1, XM_018041207.1, XM_018042596.1, XM_018042597.1, XM_018043914.1, XM_018041208.1, XM_005679934.3, XM_005689132.3, XM_018041215.1, XM_018049339.1, XM_018053841.1, XM_005684085.3, XM_018042326.1, XM_005684087.3, XM_018041216.1, XM_005688157.3, XM_018041243.1, XM_018053842.1, XM_013968441.2, XR_001917934.1, XM_018061676.1, XM_018058048.1, XM_005686658.3, NM_001314247.1, XM_018057098.1, XM_013969060.2, XR_001917319.1, XM_005689724.3, XM_018045092.1, XM_005675709.3, XM_018052505.1, XM_005677491.3, XM_018061154.1, XM_018041220.1, XM_005677996.1, XM_018061678.1, XM_018055722.1, XM_013968443.2, XM_005693787.2, XM_018041223.1, XM_018065640.1, XM_018049379.1, XM_018041212.1, XM_018043915.1, XM_018041209.1, XM_018046786.1, XM_018058683.1, XM_018053839.1, XM_018059488.1, XM_005694130.2, XM_018041232.1, XR_001917574.1, XM_005694133.3, XR_001919298.1, XM_018055723.1, XM_018047715.1, XM_018065863.1, XM_018051624.1, XM_018058049.1, XM_005686795.3, XM_018043911.1, XM_018054976.1, XM_018062384.1, XR_001917573.1, XM_018056268.1, XM_018041229.1, XM_005687938.3, XM_018041206.1, XM_018065326.1, XM_013964591.2, XM_018041231.1, XM_005693818.3, XM_018055297.1, XM_018049020.1, XM_018048780.1, XM_005697494.3, XM_018063733.1, XM_018066663.1, XM_018053522.1, XM_018049342.1, XM_018057092.1, XM_018043916.1, XM_018041221.1, XM_018045318.1, XR_001917935.1, XM_005684721.3, XM_018041241.1, XM_018041219.1, XM_018053837.1, NM_001314213.1, XM_018046784.1, XM_018048824.1, XM_018042932.1, XM_005694131.2, XM_018048606.1, XM_018046889.1, XM_018038818.1, XM_018038817.1, XM_018055446.1, XM_018041237.1, XM_018056341.1, NM_001285584.1, XM_018052165.1, XM_018050521.1, XM_018041213.1, XM_018060358.1, XM_018043913.1, XM_018044500.1, XM_018041227.1, XM_018045101.1, XM_018059935.1, XM_018041214.1, XM_018061150.1, XM_018061677.1, XM_018064039.1, XM_018052164.1, XM_013976360.2, XM_018057097.1, XM_018053843.1, XM_018049340.1, XM_018048743.1, XM_018046891.1, XM_018056721.1, XM_005689969.3, XM_018041218.1, XM_005678260.3, XM_018064219.1, XM_018058684.1, XM_018065055.1, XM_018047712.1, XM_018057094.1, XM_018041242.1, XM_018044248.1, XR_001918129.1, XM_018041239.1, XM_005684722.3, XM_018050632.1, XM_018041236.1, XM_018057099.1, XM_018047981.1, XM_018041210.1, XM_018041222.1, XM_018065707.1, XM_005677903.3 |
| 76 | [HTLV-I infection](../../../AppData/Local/Temp/Temp1_VETR-D-18-00038.zip/Cont-vs-PPRV_DEGseq_map/map05166.html) | XM_005678090.3, XM_018055645.1, XM_018052192.1, XM_018065773.1, XM_018055614.1, XM_018043204.1, XM_018065097.1, XM_005679127.2, XM_018052298.1, XM_005677388.3, XM_005695088.3, XM_005689031.3, XM_018063971.1, XM_018052301.1, XM_018042311.1, XM_018055404.1, XM_018055980.1, XM_018063035.1, XM_018053440.1, XM_018055911.1, XM_018047013.1, XM_018057893.1, XM_018040233.1, XM_018065799.1, XM_005697610.3, XM_018040236.1, XM_018055187.1, XM_018041873.1, XM_018065304.1, XM_013976164.2, XM_018060378.1, XM_018066401.1, XM_018060016.1, XM_005678576.3, XM_018054485.1, XM_005700130.3, XM_005692453.3, XM_018042310.1, XM_005684327.3, XM_018041323.1, XM_018040228.1, XM_018040239.1, XM_018062407.1, XM_018062408.1, XM_005695503.3, XM_018053228.1, XM_018058171.1, XM_018047011.1, XM_018065623.1, XM_018058066.1, XM_018055405.1, XM_018066400.1, XM_013974872.2, XM_005682299.3, XM_018047231.1, XM_018062844.1, XM_018047293.1, XM_018038784.1, XM_018056235.1, XM_005693625.3, XM_018049335.1, XM_005686247.3, XM_005695502.3, XM_005693071.3, XM_018059833.1, XM_018042308.1, XM_018044486.1, XM_018040238.1, XM_018051138.1, XM_018040231.1, XM_018062410.1, XM_018048705.1, XM_018042380.1, XM_013968554.2, XM_018065303.1, XM_005694016.2, XM_005689267.3, XM_018058447.1, XM_005684782.3, XM_005682987.1, XM_018040237.1, XM_018060376.1, XM_018061420.1, XM_018065306.1, XM_018044743.1, XM_005679129.3, XM_005675469.3, XM_005694690.3, XM_018055439.1, XM_018052297.1, XM_005677386.3, XM_013968553.2, XM_018054098.1, XM_005675289.3, XM_018065817.1, XM_005694015.2, XM_018059609.1, XR_001295577.2, XM_018042307.1, XM_018047291.1, XR_001918453.1, XM_018056690.1, XM_013972516.2, XM_005691429.3, XM_005688167.3, XM_018049271.1, XR_001918966.1, XM_018052300.1, XM_018063036.1, XM_018041876.1, XM_018047012.1, XM_018040234.1, XM_018060488.1, XM_018048812.1, XM_013967227.2, XM_018063549.1, NM_001285750.1, XM_018053442.1, XM_018056372.1, XM_013972156.2, XM_018055908.1, XM_005693569.2, XM_018062409.1, XM_005686996.3, XM_018048894.1, XM_018066613.1, XM_018039227.1, XM_005674982.3, XM_018039226.1, XM_005694519.3, XM_005686939.3, XM_018065816.1, XM_018054482.1, XM_018055186.1, XM_018054083.1, XM_018046610.1, XM_018055663.1, XM_005690676.3, XM_018060379.1, XM_018042309.1, XM_018040230.1, XM_005685503.3, XM_005689032.3, XM_018051139.1, XM_018041573.1, XM_013970271.2, XM_005684842.3, XM_018040227.1, XM_005677390.3, XM_018038904.1, XR_001918098.1, XM_013968877.2, XM_005675332.3, XM_013971256.2, XM_018055539.1, XM_005688564.3, XM_018041327.1, XM_013976163.2, XM_018060261.1, XM_018058170.1, XM_018061450.1, XM_018049347.1, XR_001917455.1, XM_018054480.1, XM_005681464.3, XM_018055403.1, XM_005691270.2, XM_018048420.1, XM_018060377.1, XM_005682139.3, XM_018053441.1, XM_018043763.1, XM_005676715.3, XM_018040235.1, XM_018049173.1, XM_018055538.1, XM_013973500.2, XM_018052842.1, XM_018054805.1, XM_018040232.1, XM_018041325.1, XR_001919375.1, XM_018055912.1, XM_018049270.1, XM_013972225.2, XM_013972226.2, XM_018065266.1, XR_001919148.1, XM_018067051.1, XM_018045673.1, XM_018042304.1, XM_018065805.1, XM_005677089.2, XM_018042305.1, XM_018059835.1, XM_018065308.1, XM_005688957.3, XM_005695085.3, XM_018053231.1, XM_018042303.1, XM_018065305.1, XM_018059836.1, XM_018043122.1, XM_018054804.1, XM_018055664.1, XM_018054483.1, XM_018055665.1, XM_005675482.2, XM_018056234.1, XM_018059834.1, XM_018041574.1, XM_018061573.1, XM_018065307.1, XM_018047010.1, XM_018053232.1 |
| 77 | [Progesterone-mediated oocyte maturation](../../../AppData/Local/Temp/Temp1_VETR-D-18-00038.zip/Cont-vs-PPRV_DEGseq_map/map04914.html) | XM_005681861.3, XM_018064273.1, XM_013964768.2, XM_018039549.1, XM_018056824.1, XM_005679127.2, XM_005684815.3, XM_018063971.1, XM_018062732.1, XM_005698856.3, XM_018063035.1, XM_013962903.2, XM_018055911.1, XM_005697610.3, XM_018055187.1, XM_018065304.1, XM_018050987.1, XM_005685130.3, XM_013972144.2, XM_018061397.1, XM_018060378.1, XM_018051619.1, XM_018057857.1, XM_018047426.1, XM_005680266.3, XM_018060016.1, XM_018060051.1, XM_005680985.3, XM_018048959.1, XM_018062407.1, XM_018062408.1, XM_018053228.1, XM_013976482.2, XM_018042428.1, XM_005681590.3, XM_013974872.2, XM_018047231.1, XM_018058500.1, XM_018041687.1, XM_005679654.2, XM_005676377.3, XM_013976502.2, XM_018056811.1, XM_018062410.1, XM_013968554.2, XM_018051622.1, XM_018065303.1, XM_018041685.1, XM_018058447.1, XM_005684782.3, XM_013963116.2, XR_001917488.1, XM_005682987.1, XM_018060376.1, XM_018065306.1, XM_005681863.3, XM_005679129.3, XM_013976483.2, XM_005681802.3, XM_018064279.1, XM_018054206.1, XM_018065969.1, XM_018063773.1, XM_018049689.1, XM_005700145.2, XM_013968553.2, XM_005675289.3, XM_018048956.1, XM_018065817.1, XM_018043354.1, XM_018041686.1, XM_018059609.1, XM_013964769.2, XM_018050822.1, XM_018042429.1, XM_018064745.1, XM_013972516.2, XM_005691429.3, XM_018047425.1, XM_018063036.1, NM_001314202.1, XM_018060488.1, XM_018049427.1, XM_013967227.2, XM_018048952.1, NM_001285750.1, XM_013972156.2, XM_018055908.1, XM_018049691.1, XM_018060052.1, XM_013972145.2, XM_018062409.1, XM_018050856.1, XM_018058529.1, XM_005697608.3, XM_018064276.1, XM_005685763.3, XM_013970533.2, XM_018065816.1, XM_018055186.1, XM_018054083.1, XM_005685483.2, XM_005679935.3, XM_005683226.3, XM_005690676.3, XM_018060379.1, XM_013963900.1, XM_005680373.3, XM_013970271.2, XM_018063176.1, XM_018065740.1, XM_013968877.2, XM_005675332.3, XM_005685146.3, XM_018064672.1, XM_005681862.2, XM_005688564.3, XM_018050820.1, XM_018060261.1, XM_018048951.1, XM_018058502.1, XM_005683187.2, XM_018049347.1, XR_001917455.1, XM_018064671.1, XM_018054815.1, XM_013964774.2, XM_018064746.1, XM_018048420.1, XM_018060377.1, XM_018057613.1, XM_018038791.1, XM_005694472.3, XM_018043763.1, XM_018043271.1, XM_018049173.1, NM_001285608.1, XM_018057864.1, XM_013964770.2, XM_018054805.1, XM_018055912.1, XM_018047444.1, XM_018062248.1, XM_018043664.1, XM_018065741.1, XM_018056659.1, XM_005680481.3, XM_018067051.1, XM_018064278.1, XM_018047424.1, XM_005681795.3, XM_018041715.1, XM_005676378.3, XM_018065308.1, XM_018064292.1, XM_018064277.1, XM_018038788.1, XM_018043565.1, XM_018053231.1, XM_018045593.1, XM_018065305.1, XM_018054804.1, XM_018049163.1, XM_018064274.1, XM_013976300.2, XM_005685145.3, XM_018057863.1, XM_018061573.1, XM_018065307.1, XM_018053232.1 |
| 78 | [Hippo signaling pathway -multiple species](../../../AppData/Local/Temp/Temp1_VETR-D-18-00038.zip/Cont-vs-PPRV_DEGseq_map/map04392.html) | XM_005678502.3, XM_005683154.3, XM_018044120.1, XM_018062382.1, XM_018050685.1, XM_018044119.1, XM_018050687.1, XM_018050690.1, XM_018049556.1, XM_018049554.1, XM_018055274.1, XM_018054459.1, XM_018050688.1, XM_018054460.1, XM_018044121.1, XM_018043278.1, XM_005683152.3, XM_018050686.1, XM_018054461.1, XM_018043396.1, XM_005685664.3, XM_018054462.1, XM_018049552.1, XM_018049551.1, XM_018044122.1, XM_018049555.1, XM_018050175.1, XM_018050691.1, XM_005685666.3, XM_018046304.1, XM_018054383.1, XM_013975781.2, XM_018049553.1, XM_018043279.1, XM_018050689.1, XM_018043395.1, XM_018055275.1, XM_018051889.1, XM_005699502.2 |
| 79 | [Axon guidance](../../../AppData/Local/Temp/Temp1_VETR-D-18-00038.zip/Cont-vs-PPRV_DEGseq_map/map04360.html) | XM_018047610.1, XM_018047903.1, XM_005681861.3, XM_018043204.1, XM_013975672.2, XM_018061294.1, XM_018045824.1, XM_013964768.2, XM_018039549.1, XM_005679127.2, XM_018052298.1, XM_018044120.1, XM_005695088.3, XM_018062382.1, XM_018052301.1, XM_018042311.1, XM_018061293.1, XM_018055980.1, XM_018044119.1, XM_018058967.1, XM_005676159.3, XM_018061289.1, XM_018054459.1, XM_018060378.1, XM_018066401.1, XM_018051619.1, XM_018054460.1, XM_018044121.1, XM_018060016.1, XR_001917214.1, XM_018043534.1, XM_018042310.1, XM_018041323.1, XM_018061295.1, XM_018048959.1, XM_018058962.1, XM_018042428.1, XM_018039024.1, XM_018058066.1, XM_018066400.1, XM_018054461.1, XM_018047231.1, XM_018042282.1, XM_005699064.3, XM_018049335.1, XM_018043396.1, NM_001286443.1, XM_018061292.1, XM_005699065.3, XM_018042308.1, XM_005680338.3, XM_018051622.1, XM_005700661.3, XM_018040436.1, XM_018060376.1, XM_018054462.1, XM_005681863.3, XM_005679129.3, XM_018054206.1, XM_018055439.1, XM_018039025.1, XM_018042281.1, XM_018052297.1, XM_018048468.1, XM_005700662.3, XM_018049689.1, XM_018045830.1, XM_005691412.3, XM_005675289.3, XM_018048956.1, XM_005691636.3, XM_018044122.1, XM_018059609.1, XM_013964769.2, XM_018042429.1, XM_018042873.1, XM_018042307.1, XM_005693161.2, XM_018064745.1, XM_018061288.1, XM_018049271.1, XM_018052300.1, XM_018042874.1, NM_001314202.1, XM_018052324.1, XM_018042279.1, XM_018048952.1, XM_005681533.3, XM_018049691.1, XM_005693569.2, XM_018058966.1, XM_005700903.1, XM_018044793.1, XM_005674982.3, XM_018042278.1, XM_005685763.3, XM_005694519.3, XM_018066938.1, XM_018046304.1, XM_018042280.1, XM_013975781.2, XM_018055663.1, XM_018060379.1, XM_018048466.1, XM_018042309.1, XM_005680676.3, XM_005685503.3, XM_018045834.1, XM_018061088.1, XM_018058968.1, XM_018039023.1, XM_018053665.1, XM_005681862.2, XM_018041327.1, XM_013963945.2, XM_018061577.1, XR_001919756.1, XM_018048951.1, XM_005681464.3, XM_018043395.1, XM_013964774.2, XM_018058965.1, XM_018064746.1, XM_005690488.3, XM_018048420.1, XM_018061291.1, XM_018056866.1, XM_018060377.1, XM_018057613.1, XM_018038791.1, XM_018058970.1, XM_018048465.1, XM_018065433.1, XM_018061286.1, XM_013964770.2, XM_018044792.1, XM_018041325.1, XM_018049270.1, NM_001285685.1, XM_018042304.1, XM_018042305.1, XM_005680013.2, XM_018064292.1, XM_005688957.3, XM_005695085.3, XM_018038788.1, XM_018042303.1, XM_005685953.3, XM_018054431.1, XM_018064357.1, XM_018043122.1, XM_018055664.1, XM_005693160.3, XM_018055665.1, XM_018064358.1, XM_018061570.1, XM_018048467.1, XM_005699502.2, XM_018061557.1 |
| 80 | [Hippo signaling pathway](../../../AppData/Local/Temp/Temp1_VETR-D-18-00038.zip/Cont-vs-PPRV_DEGseq_map/map04390.html) | XM_005678502.3, XM_018055678.1, XR_001917331.1, XM_018043204.1, XM_018060921.1, XM_018065097.1, XM_005683154.3, XM_018052298.1, XM_005682490.2, XM_018052301.1, XM_018042311.1, XM_018050685.1, XM_018050687.1, XM_018055487.1, XM_018050690.1, XM_018049556.1, XM_018049554.1, XM_018055274.1, XM_018050688.1, XM_018061256.1, XM_018058314.1, XR_001917329.1, XM_018050190.1, XR_001918973.1, XM_018042310.1, XM_018043278.1, XM_005683152.3, XM_018050686.1, XM_018059802.1, XR_001917004.1, XR_001917337.1, XM_018042308.1, XM_005685664.3, XR_001917328.1, XM_018042061.1, XM_005694016.2, XM_018059800.1, XM_013964782.2, XM_018049552.1, XM_018049551.1, XM_018052297.1, XM_018049709.1, XM_018043394.1, XM_005694015.2, XR_001917338.1, XR_001295577.2, XM_018049555.1, XM_018042307.1, XM_018050175.1, XM_018049271.1, XM_018052300.1, XM_018050691.1, XM_005685666.3, XR_001917332.1, XM_018055485.1, XR_001917335.1, XR_001297343.2, XR_001917888.1, XM_018059799.1, XM_005674982.3, XM_005684278.2, XM_018054383.1, XM_018042309.1, XM_018049553.1, XM_018043279.1, XM_018055677.1, XM_018050689.1, XR_001917330.1, XM_018055488.1, XM_005683109.3, XR_001917336.1, XM_005676715.3, XM_018055275.1, XR_001297342.2, XM_005676807.3, XR_001917333.1, XM_018049270.1, XM_018051889.1, XM_018057913.1, XM_013972225.2, XM_013972226.2, XM_018042304.1, XM_018042305.1, XM_018061406.1, XM_018055486.1, XM_018042303.1, XM_018043122.1, XR_001917334.1, XR_001917327.1 |
| 81 | [Rap1 signaling pathway](../../../AppData/Local/Temp/Temp1_VETR-D-18-00038.zip/Cont-vs-PPRV_DEGseq_map/map04015.html) | XM_018062643.1, XM_013963001.2, XM_005681861.3, XM_018064273.1, XM_018061294.1, XM_018061124.1, XM_013964768.2, XM_018065097.1, XM_018039549.1, XM_018056824.1, XM_005679127.2, XM_005682490.2, XM_005680366.2, XM_018063971.1, XM_018061293.1, XM_018063035.1, XM_018054045.1, XM_018055911.1, XM_005676159.3, XM_005697610.3, XM_018055187.1, XM_018050987.1, XM_005685130.3, XM_018061289.1, XM_005697075.3, XM_005691447.3, XM_018066370.1, XM_018060378.1, XM_018051619.1, XM_013972607.2, XM_005680364.3, XM_018060016.1, XM_005699952.3, XM_018043534.1, XM_018061295.1, XM_018048959.1, XM_018062407.1, XM_018062408.1, XM_018053228.1, XM_018042428.1, XM_005681590.3, XM_013974872.2, XM_018061125.1, XM_018047231.1, XM_018042282.1, XM_005699064.3, XM_018058500.1, NM_001286443.1, XM_018061292.1, XM_005680365.2, XM_005699065.3, XM_018039561.1, XM_018056811.1, XM_018062410.1, XM_013968554.2, XM_018051622.1, XM_005694016.2, XM_005700661.3, XM_005688518.3, XM_018041016.1, XM_018058447.1, XM_005684782.3, XM_018040436.1, XM_018060376.1, XM_005681863.3, XM_005679129.3, XM_018064279.1, XM_018054206.1, XM_018043047.1, XM_018042281.1, XM_005700662.3, XM_018049689.1, XM_005700145.2, XM_005691412.3, XM_013968553.2, XM_005675289.3, XM_018048956.1, XM_018065817.1, XM_005679333.3, XM_005694015.2, XM_018059609.1, XM_013964769.2, XR_001295577.2, XM_018042429.1, XM_018064745.1, XM_018061288.1, XM_018066371.1, XM_018057813.1, XM_018063036.1, NM_001314202.1, XM_018060488.1, XM_018049427.1, XM_018052324.1, XM_018042279.1, XM_013967227.2, XM_018048952.1, NM_001285750.1, XM_013970799.2, XM_005681533.3, XM_018055908.1, XM_018049691.1, XM_018043593.1, XM_018062409.1, XR_001295516.2, XM_018050856.1, XM_018042278.1, XM_018064276.1, XM_005685763.3, XM_005680367.3, XM_005684278.2, XM_018065816.1, XM_018055186.1, XM_018054083.1, XM_005676485.3, XM_018042280.1, XM_005685150.3, XM_005683226.3, XM_005686574.3, XM_013966779.2, XM_018060379.1, XM_005680676.3, XM_005680373.3, XM_013970271.2, XM_013968877.2, XM_005675332.3, XM_005685146.3, XM_018064672.1, XM_005681862.2, XM_005688564.3, XM_018060261.1, XR_001919756.1, XM_018048951.1, XM_018058502.1, XR_001296033.2, XM_018064671.1, XM_013964774.2, XM_018064746.1, XM_018048420.1, XM_018061291.1, XM_018060377.1, XM_018057613.1, XM_018066659.1, XM_018038791.1, XM_005694472.3, XM_005676715.3, XM_018065433.1, XM_018054044.1, XM_018061286.1, XM_013964770.2, XM_005676482.3, XM_018055912.1, XM_005685149.3, XM_013972225.2, XM_013972226.2, NM_001285685.1, XM_018067051.1, XM_018064278.1, XM_005697074.3, XM_005680013.2, XM_018064292.1, XM_018064277.1, XM_018038788.1, XM_018053231.1, XM_018045593.1, XM_013976047.2, XM_005685953.3, XM_018054431.1, XM_018064274.1, XM_005685145.3, XM_005694349.2, XM_005699951.1, XM_018053232.1 |
| 82 | [Ubiquitin mediated proteolysis](../../../AppData/Local/Temp/Temp1_VETR-D-18-00038.zip/Cont-vs-PPRV_DEGseq_map/map04120.html) | XM_018039441.1, XM_013967308.2, XM_018041147.1, XM_018052192.1, XM_013972619.2, XM_018054712.1, XM_018065489.1, XM_018066525.1, XM_005700284.3, XM_005680196.3, XM_018061960.1, XM_018053122.1, XM_018054472.1, XM_018063746.1, XM_018058577.1, XM_018056494.1, XM_018064024.1, XM_018060550.1, XM_018056727.1, XM_018040887.1, XM_018055095.1, XM_005689847.3, XM_018046826.1, XM_005690715.3, XM_018053123.1, XM_018063745.1, XM_018066484.1, XM_018042296.1, XM_018065304.1, XM_018054244.1, XM_018067209.1, XM_018041146.1, XM_018059058.1, XM_018066483.1, XM_018043737.1, XM_018046703.1, XM_018054327.1, XM_018065495.1, XM_018057047.1, XM_018065064.1, XM_018047001.1, XM_018050095.1, XM_018065063.1, XM_018054324.1, XM_005678576.3, XM_018061959.1, XM_018064440.1, XM_018046699.1, XM_018066485.1, XM_005690792.3, XM_018052772.1, XM_018065062.1, XM_018066091.1, XM_018056007.1, XM_018040226.1, XM_018043487.1, XM_018057044.1, XM_018043733.1, XM_018065626.1, XM_005687449.3, XM_018039820.1, XM_018052369.1, XM_018043506.1, XM_018044970.1, XM_018046641.1, XM_018041148.1, XM_018043734.1, XM_018046823.1, XM_018038813.1, XM_018053601.1, XM_018058586.1, XM_018054330.1, XM_018050097.1, XM_018056732.1, XM_018039445.1, XM_018056493.1, XM_018040378.1, XM_018041394.1, XM_018054715.1, XR_001918492.1, XM_013967310.2, XM_018059059.1, XM_018058613.1, XM_018039775.1, XM_018057045.1, XM_018043738.1, XM_018040259.1, XM_018066090.1, XM_018040222.1, XM_018065303.1, XM_005694016.2, XM_018054713.1, XM_018065893.1, XM_018054474.1, XM_018054473.1, XM_018039443.1, XM_005682987.1, XM_018043730.1, XM_018056729.1, XM_018054322.1, XM_005681612.3, XM_018065490.1, XM_018066482.1, XM_018065306.1, XM_018058599.1, XM_018047002.1, XM_018065888.1, XM_013968297.2, XM_018043729.1, XM_018039440.1, XM_005686082.2, XM_018054323.1, XM_018065492.1, XM_005675405.3, XM_018049456.1, XM_018049508.1, XM_018042979.1, XM_018056006.1, XM_018061400.1, XM_005694015.2, XM_018048340.1, XM_005681443.3, XM_005679467.3, XM_018056490.1, XM_018039444.1, XM_018050822.1, XR_001295577.2, XM_005690161.3, XM_018046825.1, XM_013966746.2, XM_005675805.3, XM_018063744.1, XM_005683225.3, XR_001918453.1, XM_018046929.1, XM_013972516.2, XM_018054398.1, XM_005691429.3, XM_018043652.1, XM_018056491.1, XM_018046640.1, XR_310306.3, XR_001918966.1, XM_018062219.1, XM_018053151.1, XM_018067211.1, XM_018067210.1, XM_005686628.2, XM_018059227.1, XM_018043653.1, XM_018039821.1, XM_018061961.1, XM_005689800.3, XM_018054475.1, XM_018043736.1, XM_005691174.3, XM_005675491.2, XM_013972156.2, XM_005691173.3, XM_018048223.1, XM_018052113.1, XM_005675493.3, XM_018039975.1, XM_005700344.3, XM_018057046.1, XM_018046639.1, XM_005694793.3, XM_018056000.1, XM_018066078.1, XM_018040224.1, XM_005691175.3, XM_018056005.1, XM_018039973.1, XM_018054326.1, XM_018065494.1, XM_018062665.1, XM_018057738.1, XM_018062011.1, XM_018039974.1, XM_018047623.1, XM_005681656.3, XM_018053483.1, XM_005689072.3, XM_005681613.3, XM_005686081.3, XM_018039976.1, XM_013970741.2, XM_018040223.1, XM_005689513.3, XM_018054328.1, XM_005685180.3, XM_018048341.1, XR_001918587.1, XM_018061404.1, XM_018061099.1, XM_018043732.1, XM_013967307.2, XM_018043731.1, XM_018056737.1, XM_018061100.1, XM_005688476.3, XM_018061958.1, XM_018046824.1, XM_018043735.1, XM_018050820.1, XM_018060852.1, XM_018059228.1, XM_018049347.1, XR_001919118.1, XM_018056734.1, XM_018058606.1, XM_018058895.1, XM_013968550.2, XM_018058897.1, XR_001917455.1, XM_018065488.1, XM_005682942.3, XM_005695946.3, XM_018043207.1, XM_018042295.1, XM_018056004.1, XM_005693801.3, XM_018062297.1, XM_005695943.2, XM_005699742.3, XM_018056001.1, XM_005690714.3, XM_018044397.1, XM_018058571.1, XM_018056009.1, XM_018066092.1, XM_018065894.1, XM_018054329.1, XM_018043763.1, XM_018066486.1, XM_018066077.1, XM_018046827.1, XM_018041603.1, XM_013972845.2, XM_018054805.1, XM_018061098.1, XM_005678770.3, XM_018065493.1, XM_005697400.2, XM_018058635.1, XM_018066488.1, XM_005689543.3, XM_013970672.2, XM_018042980.1, XM_018040260.1, XM_018054325.1, XM_005695948.3, XM_018039442.1, XM_018050096.1, XM_018039774.1, XM_018054397.1, XM_018062664.1, XM_018045265.1, XM_018065308.1, XM_018038280.1, XM_018058624.1, XM_018061097.1, XM_018041486.1, XM_018040257.1, XM_018049314.1, XM_018040664.1, XM_018041149.1, XM_018040774.1, XM_018058566.1, XM_013966805.2, XM_005686083.3, XM_018065305.1, XM_005685179.3, XM_005687450.3, XM_005690704.3, XM_018066487.1, XM_018054804.1, XM_018058591.1, XM_005689846.3, XR_001296778.2, XM_018043651.1, XM_018061573.1, XM_018065307.1 |
| 83 | [Peroxisome](../../../AppData/Local/Temp/Temp1_VETR-D-18-00038.zip/Cont-vs-PPRV_DEGseq_map/map04146.html) | XM_018053976.1, XM_018047534.1, XM_005698484.3, XM_005682183.3, XM_018044177.1, XM_005676340.3, XM_018066762.1, XM_018051296.1, XM_018048533.1, XM_018053428.1, XM_013964473.2, XM_005689252.3, XM_018053073.1, XM_005676339.3, XM_018047429.1, XM_013964462.2, XM_018060482.1, XM_005686074.3, XM_018059189.1, XM_018047532.1, XM_013975121.2, XM_018061103.1, XM_013965100.2, XM_018047533.1, XM_018049991.1, XM_013967803.2, XM_005684804.3, XM_018042091.1, XM_005686901.3, XM_013965431.2, XM_018044777.1, XM_013963869.2, XM_013967804.2, XM_018058912.1, XM_018045789.1, XM_018061126.1, XM_018052335.1, XM_018045910.1, XM_005691582.3, XM_018066614.1, XM_005688179.3, XM_005698717.3, XM_005676858.2, NM_001285624.1, XM_005691592.3, XM_018052333.1, XM_018039967.1, XM_005676341.3, XM_005698318.3, XM_005684904.3, NM_001285675.1, XM_013967802.2, XR_001918220.1, XM_005679551.3, XM_018061457.1, XM_018060481.1, XM_018059582.1, XM_005690077.3, XM_018041881.1, XM_018066021.1, XM_013964476.2, XM_013975666.1, XM_018041882.1, XM_018048537.1, XM_018051295.1, XR_001295453.2, XM_018039038.1, XM_018048532.1, XM_018048536.1, XM_013975120.2, XM_005693204.3, XM_005688180.3, XM_018048531.1, XM_018052334.1, XM_018049794.1, XM_005685235.3, XM_018044562.1, XM_013967801.2, XR_001917308.1, XM_018066760.1, XM_005686386.3, XM_013971375.2, XM_005676342.3, XM_018050725.1, XM_018042090.1, XM_005677786.3, XM_018066761.1, XM_018047430.1, XM_005678109.3, XM_005692378.3, XM_018048534.1, XM_018059583.1, XM_018042092.1, XM_005677789.3, XM_005698716.3, XM_005676343.3, XM_018045331.1, XM_005689111.3, XM_005691583.3, XM_018049946.1, XM_018039037.1, XM_018066759.1, XM_018060480.1, XM_005698718.3, XM_018042093.1, XM_005675270.3, XM_005686902.3, XM_005682126.3, XM_013967404.2, XM_018048535.1, XM_018038608.1, NM_001285762.1, XM_018066763.1, XM_005684042.2, XM_005677224.3, XM_005684903.3 |
| 84 | [Fluid shear stress and atherosclerosis](../../../AppData/Local/Temp/Temp1_VETR-D-18-00038.zip/Cont-vs-PPRV_DEGseq_map/map05418.html) | XM_005699593.3, XM_018044652.1, XM_018062643.1, XM_013973238.2, XM_018058079.1, XM_013963001.2, XM_018043283.1, XM_005677656.3, NM_001314231.1, NM_001314266.1, XM_018061294.1, XM_018061124.1, XM_018065097.1, XM_018065831.1, XM_005679127.2, XM_018063832.1, XM_005682490.2, XM_005680366.2, XM_018063971.1, XM_018061046.1, XR_001917497.1, XM_018038998.1, XM_018061293.1, XM_018052124.1, XM_018063035.1, XM_018054045.1, XM_018055911.1, XM_018063078.1, XM_005676159.3, XM_018054628.1, XM_005697610.3, XM_018055187.1, XM_018059746.1, XM_018058560.1, XM_018040963.1, XM_018061289.1, XM_005697075.3, XM_018060171.1, XM_005691447.3, XM_018066370.1, XM_018060378.1, XM_005680647.3, XM_018065829.1, XM_005683422.3, XM_018043280.1, XM_005686991.3, XM_013972607.2, XM_018065827.1, XM_005699594.3, XM_005678693.3, XM_005680364.3, XM_018060016.1, XM_013965777.2, XM_005699952.3, XM_018043282.1, XM_018043534.1, XM_018061295.1, XM_018043281.1, XM_018049972.1, XM_018060045.1, XM_005683420.3, XM_018062407.1, XM_018043288.1, XM_018065501.1, XM_018062408.1, XM_005679901.2, XM_018053228.1, XM_013965781.2, XM_005683419.3, XM_018043180.1, XM_018062590.1, XM_013974872.2, XM_018061125.1, XM_005677944.3, XM_018047231.1, XM_018065834.1, XM_018044746.1, XM_018042282.1, XM_018065832.1, XM_005699064.3, XM_018051782.1, NM_001286443.1, XM_018051481.1, XM_018061292.1, XM_005680365.2, XM_005699065.3, XM_018039331.1, XM_018057817.1, XM_018039561.1, XM_018045878.1, XM_018062410.1, XM_013968554.2, XM_005694016.2, XM_005700661.3, XM_005688518.3, XM_018041016.1, XM_018058447.1, XM_005684782.3, XM_018040436.1, XM_018060376.1, XM_018048184.1, XM_005691231.3, XM_005679129.3, XM_018065969.1, XM_018043047.1, XM_018042281.1, XM_005700662.3, XM_005681337.3, XM_005683423.3, XM_005691412.3, XM_005681336.3, XM_013968553.2, XM_005675289.3, XM_018065817.1, XM_005679333.3, XM_005694015.2, XM_018059609.1, XM_005698444.3, XM_013962290.2, XR_001295577.2, XM_018060043.1, XM_018061288.1, XM_018066371.1, XM_018057813.1, XM_013975063.2, XM_018051785.1, XM_018063036.1, XM_018051501.1, XM_018060488.1, XR_001296084.2, XM_018052324.1, XM_018042279.1, XM_013967227.2, XM_018059798.1, NM_001285750.1, XM_013970799.2, XM_013966480.2, XM_005681533.3, XR_001917495.1, XM_018055908.1, XM_018051784.1, NM_001314204.1, XM_018043593.1, XM_018062409.1, XM_013973240.2, XR_001295516.2, XM_018042278.1, XM_005702078.3, XM_013970533.2, XM_005680367.3, XM_005684278.2, XM_018065816.1, XM_018055186.1, XM_018054083.1, XM_005676485.3, NM_001285615.1, XM_018042280.1, XM_005685150.3, XM_005686574.3, XM_013966779.2, XM_018060379.1, XM_005680676.3, XM_013962990.2, XM_018059747.1, XM_018051760.1, XM_018065835.1, XM_018040964.1, XM_018054630.1, XM_018065828.1, XM_013970271.2, XR_001917995.1, XM_018060042.1, XM_005698442.3, XM_018065830.1, XM_005684675.3, XM_018066076.1, XM_013968877.2, XM_018066944.1, XM_005675332.3, XM_005688564.3, XR_001296083.2, XM_018060261.1, XR_001919756.1, XM_018062591.1, XM_018045880.1, XM_005695428.3, XM_013965779.2, XR_001918314.1, XR_001296033.2, XM_018059745.1, XM_018059748.1, XM_018048420.1, XM_018061291.1, XM_018060377.1, XM_018054629.1, XM_005695430.2, XM_005676715.3, XM_018065433.1, XM_018041744.1, XM_018054044.1, XM_018061286.1, XM_018039332.1, XM_018043284.1, XM_018051783.1, XM_005676482.3, XM_013965778.2, XM_018058102.1, XM_018055912.1, XM_018051781.1, XM_018043190.1, XM_005685149.3, XM_013972225.2, XM_005695427.3, XM_013972226.2, XM_005682905.3, NM_001285685.1, XM_018060044.1, XM_018067051.1, NM_001285567.1, XM_005691743.3, XM_018066075.1, XM_005696428.3, XM_005697074.3, XM_005680013.2, XM_018048933.1, XM_005682904.3, XM_005695429.3, XM_018058103.1, XM_018053231.1, XM_018065836.1, XM_018065502.1, XM_013976047.2, XM_005695431.3, XM_005685953.3, XM_018054431.1, XM_005694349.2, XM_018043287.1, XM_005699951.1, XM_018055626.1, XM_018045875.1, XR_001917496.1, XM_018053232.1 |
| 85 | [Glucagon signaling pathway](../../../AppData/Local/Temp/Temp1_VETR-D-18-00038.zip/Cont-vs-PPRV_DEGseq_map/map04922.html) | XM_005699593.3, XM_018044652.1, XM_018062643.1, XM_013973238.2, XM_018058079.1, XM_013963001.2, XM_018043283.1, XM_013970632.2, XM_018061124.1, XM_018045824.1, XM_005684936.3, XM_018052298.1, XM_005695088.3, XM_005680366.2, XM_018063971.1, XM_018052301.1, XM_018061046.1, XM_018042311.1, XR_001917497.1, XM_018055980.1, XR_001295907.2, XM_018063035.1, XM_018054045.1, XM_018055911.1, XM_018063078.1, XM_018058967.1, XM_013971629.2, XM_018054628.1, XM_005697610.3, XM_005686623.2, XM_018055187.1, XR_001918100.1, XM_005680737.3, XM_018059746.1, XM_018058560.1, XM_018040963.1, XM_005697075.3, XM_018060171.1, XM_005691447.3, XM_018066370.1, XM_018066401.1, XM_013970631.2, XM_005680647.3, XM_018043280.1, XM_005686991.3, XM_018064170.1, XM_013972607.2, XM_005699594.3, XM_005678693.3, XM_005680364.3, XM_018057287.1, XM_005699952.3, XM_018043282.1, XM_018042310.1, XM_018041323.1, XM_018043281.1, XM_018046692.1, XM_018062407.1, XM_018043288.1, XM_018046693.1, XM_018065501.1, XM_018062408.1, XM_018058962.1, XM_018053228.1, XM_018046914.1, XM_018043180.1, XM_018062590.1, XM_018058066.1, XM_018066400.1, XM_018066119.1, XR_001919014.1, XM_013974872.2, XM_018061125.1, XM_018044746.1, XM_018049335.1, XM_018062799.1, XM_005680365.2, XM_018042308.1, XM_018057817.1, XM_018039561.1, XM_018062410.1, XM_018046913.1, XM_013968554.2, XM_005688518.3, XM_018041016.1, XM_018058447.1, XM_005684782.3, XM_018046688.1, XM_005686624.3, XM_018055439.1, XM_018043047.1, XM_018052297.1, XM_005681337.3, XM_018046689.1, XM_018045830.1, XM_005681336.3, XM_013968553.2, XM_018065817.1, XM_018064174.1, XM_005685177.3, XM_005679333.3, XM_013962290.2, NM_001285647.1, XM_018042307.1, XM_005699532.3, XM_018046694.1, XM_018066371.1, XM_018049271.1, XM_018057813.1, XM_018052300.1, XM_018063036.1, XM_018051501.1, XM_018060488.1, XM_018048812.1, XM_013967227.2, XM_018059798.1, NM_001285750.1, XM_013970799.2, XR_001917495.1, XM_018055908.1, XM_018039937.1, XM_005693569.2, XM_018043593.1, XM_018062409.1, XM_013973240.2, XR_001295516.2, XM_018058966.1, XM_018066613.1, XM_018054332.1, XM_005702078.3, XM_005694519.3, XM_018046691.1, XM_013970533.2, XM_005680367.3, XM_018054351.1, XM_005699535.3, XM_018065816.1, XM_018055186.1, XM_018054083.1, XM_005676485.3, XM_005679489.3, XM_005685150.3, XM_018055663.1, XM_005686574.3, XM_013966779.2, XM_018064169.1, XM_005699864.3, XM_018042309.1, XM_013962990.2, XM_005685503.3, XM_018045834.1, XM_018059747.1, XM_018051760.1, XM_018040964.1, XM_018054630.1, XM_018058968.1, XM_013970271.2, XM_005699526.3, XM_018066076.1, XM_013968877.2, XM_018064172.1, XM_018066944.1, XM_005675332.3, XM_005688564.3, XM_018041327.1, XM_018060261.1, XM_018062591.1, XM_005695428.3, XR_001918314.1, XM_005681464.3, XR_001296033.2, XM_018059745.1, XM_018058965.1, XM_013971630.2, XM_018064173.1, XM_018059748.1, XM_018054629.1, XM_018058970.1, XM_005695430.2, XM_018061094.1, XM_005695487.3, XM_018041744.1, XM_018054044.1, XM_018064168.1, XM_018043284.1, XM_005676482.3, XM_018046690.1, XM_018058102.1, XM_018041325.1, XM_018055912.1, XM_018049270.1, XM_018043190.1, XM_005685149.3, XM_005695427.3, XM_005682905.3, XM_018067051.1, XM_018042304.1, XM_018066075.1, XM_018042305.1, XM_005697074.3, XM_018048933.1, XM_005682904.3, XM_005695429.3, XM_005688957.3, XM_005695085.3, XM_013973161.2, XM_018058103.1, XM_018053231.1, XM_018042303.1, XM_018065502.1, XM_013976047.2, XM_005695431.3, XM_018055664.1, XM_018055665.1, XM_005694349.2, XM_018043287.1, XM_005699951.1, XM_018055626.1, XR_001917496.1, XM_013976777.2, XM_013962471.2, XM_018064171.1, XM_018053232.1 |
| 86 | [Estrogen signaling pathway](../../../AppData/Local/Temp/Temp1_VETR-D-18-00038.zip/Cont-vs-PPRV_DEGseq_map/map04915.html) | XM_018062643.1, XM_013963001.2, XM_005681861.3, XM_013968498.2, XM_018042992.1, XM_018061124.1, XM_013964768.2, XM_018039549.1, XM_005679127.2, XM_005680366.2, XM_018063971.1, XM_018063035.1, XM_018054045.1, XM_018055911.1, XM_005697610.3, NM_001314233.1, XM_018055187.1, XR_001918100.1, XM_018050987.1, XM_005680737.3, XM_005685130.3, XM_005697510.3, XM_005697075.3, XM_005691447.3, XM_018066370.1, XM_018060378.1, XM_018051619.1, NM_001314206.1, XM_013972607.2, XM_005680364.3, XM_018060016.1, XM_005699952.3, XM_018048959.1, XM_018055448.1, XM_018062407.1, XM_005694317.3, XM_018062408.1, XM_005694986.3, XM_018053228.1, XM_005694985.3, XM_018042428.1, XM_018057132.1, XM_005682995.3, XM_013974872.2, XM_018057915.1, XM_018061125.1, XM_018047231.1, XM_005679207.3, XM_018038959.1, XM_005680365.2, NM_001314303.1, XM_018039561.1, XM_018062410.1, XM_013968554.2, XM_018051622.1, XM_005688518.3, XM_018041016.1, XM_018058447.1, XM_005684782.3, XM_018060376.1, XM_018048184.1, XM_005681863.3, XM_005679129.3, XM_018054206.1, XM_018065969.1, XM_018043047.1, XM_018049689.1, XM_013968553.2, XM_005675289.3, XM_018048956.1, XM_018065817.1, XM_005679333.3, XM_018059609.1, XM_013964769.2, XM_018042429.1, XM_018064745.1, XM_018066371.1, XM_013972970.2, XM_018057813.1, XM_018063036.1, NM_001314202.1, XM_018060488.1, XM_013967227.2, XM_018048952.1, NM_001285750.1, XM_013970799.2, XM_018055908.1, XM_018049691.1, XM_018043593.1, XM_018062409.1, XR_001295516.2, XM_018050856.1, XM_005685763.3, XM_005680367.3, XM_018065816.1, XM_018055186.1, XM_018054083.1, XM_005676485.3, XM_005685150.3, XM_005686574.3, XM_013966779.2, XM_018060379.1, XM_005677146.3, XM_018038957.1, XM_005699791.3, XM_013970271.2, XM_013968877.2, XM_005675332.3, XM_005685146.3, XM_018064672.1, XM_005681862.2, XM_005688564.3, XM_018060261.1, XM_018048951.1, XR_001296033.2, XM_018064671.1, XM_013964774.2, XM_018064746.1, XM_005689565.3, XM_018048420.1, XM_018060377.1, XM_018057613.1, XM_018038791.1, XM_005694472.3, NM_001285703.1, XM_018054044.1, XM_013964770.2, XM_005676482.3, XM_018055912.1, XM_005685149.3, XR_001918647.1, XM_005674769.3, XM_018067051.1, XM_005697074.3, XM_005693206.3, XM_018064292.1, XM_018038788.1, XM_018053231.1, XM_013976047.2, XM_005685145.3, XM_005694349.2, XM_005699951.1, XM_013968497.2, XM_018053232.1 |
| 87 | [Choline metabolism in cancer](../../../AppData/Local/Temp/Temp1_VETR-D-18-00038.zip/Cont-vs-PPRV_DEGseq_map/map05231.html) | XM_005678301.3, XM_005700482.2, XM_005676457.3, XM_005681861.3, XM_018043641.1, XM_005699804.2, XM_018049587.1, XM_018038704.1, XM_005681449.3, XM_005699805.3, XM_018061294.1, XM_018051455.1, XM_018065386.1, XM_018065920.1, XM_018060694.1, XM_013964768.2, XM_013962541.2, XR_001920008.1, XM_005686929.1, XM_018056451.1, XM_005679127.2, XM_005680381.3, XM_005687164.3, XM_005681344.3, XM_018063971.1, XM_018047786.1, XM_018059721.1, XM_005700749.3, XM_018038707.1, NM_001285748.1, XM_018064091.1, XM_018061293.1, NM_001285755.1, XM_018046075.1, XM_013970381.2, XM_018065768.1, XM_018063035.1, NM_001285656.1, XM_018065385.1, XM_018039464.1, XM_018047734.1, XM_018053461.1, XM_018055821.1, XM_018055911.1, XM_018057743.1, XM_018066315.1, XM_018054409.1, XM_005676159.3, XM_018047363.1, XM_018041498.1, XM_018047090.1, XM_013967930.2, XM_005697610.3, XM_018047096.1, XM_018061205.1, XM_005683110.3, XM_018047989.1, XM_018055187.1, XM_018050987.1, XM_018038705.1, XM_005688391.2, XM_018039502.1, XM_005685130.3, XM_005699807.3, XM_018061289.1, XM_018039816.1, XM_018049792.1, XM_018051454.1, XM_018050170.1, XM_018060378.1, XM_018051619.1, XM_005680005.2, XM_018038794.1, XM_018052556.1, XM_018065915.1, XM_018058810.1, XM_018054271.1, XM_018060016.1, XM_018049019.1, XM_018043534.1, XM_018056326.1, XM_018053435.1, XM_005678299.3, XM_018061295.1, XM_018046369.1, XM_018048959.1, XM_018047736.1, XM_018054477.1, XM_005680368.3, XM_018062407.1, XM_005693111.3, XM_018062408.1, XM_018043792.1, XM_018051788.1, XM_018053228.1, XM_018046371.1, XM_018041190.1, XM_018042428.1, XM_005675354.3, XM_005676376.3, XM_018043463.1, XM_018065520.1, XM_005682678.3, XM_018047802.1, XM_018066594.1, XR_001918038.1, XM_018049341.1, XM_018051456.1, XM_013974872.2, XM_018038702.1, XM_018039861.1, XM_018058924.1, XM_018046894.1, XM_005687933.3, XM_018041080.1, XM_018046078.1, XM_018047231.1, XM_018052391.1, XM_018065891.1, XM_018055602.1, XM_005684952.3, XM_018050527.1, XM_018051067.1, XM_018053460.1, XM_018039462.1, XM_018061581.1, XM_018051194.1, XM_005697888.3, XM_005677615.3, XM_018064579.1, XM_018053321.1, XM_018042282.1, XM_013975922.1, XM_018044608.1, XM_005680937.3, XM_005699064.3, XM_005693625.3, XM_018049339.1, NM_001286443.1, XM_018061292.1, XM_018062604.1, XM_018059467.1, XR_001919089.1, XM_018058077.1, XM_018054401.1, XM_018048105.1, XM_005699065.3, XM_005690990.2, XM_018048285.1, XM_018050610.1, XM_018062387.1, XM_018060732.1, XM_018049860.1, XM_018038798.1, XR_001919737.1, XR_001918229.1, XM_018046074.1, XR_001917934.1, XM_018062410.1, XM_013976723.2, XM_018038804.1, XM_005678056.3, XM_018047360.1, XM_005675490.2, XM_018063683.1, XM_013968554.2, XM_018061746.1, XM_018051622.1, XM_018051457.1, XM_018058390.1, XM_018038703.1, XM_005678985.3, XM_005698535.3, XM_018040326.1, XM_005700661.3, XM_018062589.1, XM_018058447.1, XM_005684782.3, XM_013964590.2, XM_018040436.1, XR_001919481.1, XM_018042871.1, XM_018060376.1, XM_018047364.1, XM_005681863.3, XM_018065059.1, XM_018063801.1, XM_018060738.1, XM_005679129.3, XM_018059162.1, XM_018053462.1, XM_018058389.1, XM_018053743.1, XM_018054206.1, XM_005677614.3, XM_018042281.1, XM_013968710.2, XM_005700662.3, XM_018049689.1, XM_018061302.1, XM_018049862.1, XM_005691412.3, XM_005686332.3, XM_018042156.1, XM_013968553.2, XM_018043642.1, XM_005675289.3, XM_018055025.1, XM_018048956.1, XM_018065817.1, XM_005700115.3, XM_018048018.1, XM_018053437.1, XM_018061582.1, XM_018038708.1, XM_018049858.1, XM_018038802.1, XM_018049859.1, XM_018043794.1, XM_018043791.1, XM_018059609.1, XM_018057082.1, XM_018054405.1, XM_018048734.1, XM_013964769.2, XM_018042429.1, XM_005678357.3, XM_018048549.1, XM_018043036.1, XM_018055747.1, XM_018056791.1, XM_018064745.1, XM_018061554.1, XM_018047257.1, XM_018054406.1, XM_005696787.3, XM_018061288.1, XM_018048019.1, XM_018056794.1, XM_018046786.1, XM_018053072.1, XM_018058697.1, XM_018054571.1, XM_018059164.1, XM_018064220.1, XM_018044352.1, XM_018055369.1, XM_018063036.1, XM_018056792.1, XM_018058397.1, XM_018067122.1, NM_001314202.1, XM_013966433.2, XM_018060488.1, XM_018061662.1, XM_018046227.1, XM_018060739.1, XM_018052324.1, XM_018042279.1, XM_005683717.3, XM_013967227.2, XM_018048224.1, XM_013965507.2, XM_018048952.1, XM_013962921.2, NM_001285641.1, NM_001285750.1, XM_018049736.1, XM_018042765.1, XM_018048995.1, XM_018041133.1, XM_018049791.1, XM_005676458.3, XM_005681533.3, XM_018058387.1, XM_018055908.1, XM_005701006.3, XM_018049691.1, XM_018052065.1, XM_018050833.1, XM_013972667.2, NM_001291820.1, XM_018038796.1, XM_018062409.1, XM_018053966.1, XM_018059160.1, XM_005685851.3, XM_018061533.1, XM_018059919.1, XM_018040337.1, XM_005675639.3, XM_018047254.1, XM_018050856.1, XM_018039463.1, XM_018042278.1, XM_018060820.1, XM_018064986.1, XM_005685763.3, XM_005693259.3, XM_018059166.1, XM_005692564.3, XM_018054878.1, XM_018057897.1, XM_018040906.1, XM_018053324.1, XM_018065816.1, XM_018055186.1, XM_018054083.1, XM_018042280.1, XM_018041036.1, XM_018062603.1, XM_018054402.1, XM_018052032.1, XM_018042024.1, XM_018064237.1, XM_005709596.3, XM_018054408.1, XM_018038803.1, XM_018051789.1, XM_018060379.1, XM_018058646.1, XM_018054611.1, XM_018050834.1, XM_005680676.3, XM_018049342.1, XM_018061830.1, XM_018049951.1, XM_005675640.3, XM_018065605.1, XM_018039695.1, XM_005684658.2, XM_005694936.3, XM_005696333.3, XM_018058634.1, XM_018040244.1, XR_001917935.1, XM_018059720.1, XR_001918230.1, XM_013970271.2, XM_018058388.1, XM_018064350.1, XM_018066630.1, XM_018046226.1, XM_013962880.2, XM_018046784.1, XM_013963165.2, XM_018060838.1, XM_018040360.1, XM_018039193.1, XM_018054403.1, XM_018043927.1, XM_005677617.3, XM_018059125.1, XM_013968877.2, XM_005699809.2, XM_005678171.3, XM_018051206.1, XM_005675332.3, XM_005685146.3, XM_018053225.1, XM_018061745.1, XM_018064672.1, XM_005681862.2, XM_005688564.3, XM_018047359.1, XM_018060261.1, XR_001919756.1, XM_018046893.1, XM_005680662.3, XM_018058396.1, XM_018048951.1, XM_018049857.1, XM_018058395.1, XM_018064988.1, XM_018049721.1, XM_005678984.3, XM_018041043.1, XM_018053831.1, XM_005687163.3, XM_005699801.3, XM_005685526.3, XM_018065503.1, NM_001285594.1, XM_005681688.3, XM_018064671.1, XM_018042095.1, XM_013964774.2, XM_018064746.1, XM_018043293.1, XM_018061580.1, XM_018064499.1, XM_018050609.1, NM_001287233.1, XR_001918228.1, XM_018045244.1, XM_018048420.1, XM_018061291.1, XM_018062434.1, XM_005680383.3, XM_018060377.1, XM_018053320.1, XM_018038791.1, XM_018043793.1, XM_018048104.1, XM_005680829.3, XM_005694472.3, XM_018043784.1, XM_018044010.1, XM_018059161.1, XM_018060089.1, XM_018065433.1, XM_018049861.1, XM_018061286.1, XM_018044227.1, XM_018061071.1, XM_013965671.2, XM_018056393.1, XM_018049340.1, XM_018048743.1, XM_005675352.3, XM_013964770.2, XM_018059165.1, XM_018064970.1, XM_005677702.3, XM_005693177.3, XM_018067123.1, XM_005693110.2, XM_005684192.3, XM_018050781.1, XM_018044012.1, XM_018053830.1, XM_018058394.1, XM_005675809.3, XM_018056721.1, XM_005678058.3, XM_005678170.3, XM_018054407.1, XM_018038706.1, XM_018055912.1, XM_018052563.1, XM_018042023.1, XM_018053071.1, XM_018063375.1, XM_005696565.3, NM_001285685.1, XM_018064131.1, XM_018063972.1, XM_018059167.1, XM_018067051.1, XR_001919090.1, XM_018059918.1, XM_018067124.1, XM_005691714.3, XM_018059163.1, XM_005680013.2, XM_018048550.1, XM_013968393.2, XM_018065918.1, XM_018063570.1, XM_005691591.3, XM_005699808.3, XM_018064292.1, XM_018057758.1, XM_018061303.1, XM_018044607.1, XM_018041050.1, XM_018059468.1, XR_001917130.1, XM_018049789.1, XM_013968876.2, XM_018038788.1, XM_018061555.1, XM_013963166.2, XM_018053323.1, XM_018041495.1, XM_018053231.1, XM_018047553.1, XM_018038709.1, XM_005679729.3, XM_005686075.3, XM_018043795.1, XM_018039191.1, XR_001919517.1, XM_005685953.3, XM_018051453.1, XM_018054431.1, XM_005675512.3, XM_018053392.1, XM_018046815.1, XM_005675060.3, XM_018039465.1, XM_005685145.3, XM_018038795.1, XM_018048748.1, XM_018050867.1, XM_018053436.1, XM_018054731.1, XR_001918643.1, XM_018052582.1, XM_005686905.3, XM_018053232.1 |
| 88 | [Pathogenic Escherichia coli infection](../../../AppData/Local/Temp/Temp1_VETR-D-18-00038.zip/Cont-vs-PPRV_DEGseq_map/map05130.html) | XM_005678301.3, XR_310868.3, XM_005700482.2, XM_005676457.3, XM_018048239.1, XM_018040631.1, XM_018043641.1, XM_018049587.1, XM_018038704.1, XM_005681449.3, XR_001917482.1, XM_018051455.1, XM_018065386.1, XM_018065920.1, XM_018060694.1, XM_018065097.1, XM_005686929.1, XM_018040560.1, XM_005687164.3, XM_005682490.2, XM_005681344.3, XM_018059721.1, XM_005700749.3, XM_018038707.1, NM_001285755.1, XM_018046075.1, XM_018065768.1, XM_018048244.1, XM_018040595.1, XM_018040431.1, XM_018065385.1, XM_018039464.1, XM_018047734.1, XM_018055821.1, XM_018057743.1, XR_001918852.1, XM_018066315.1, XM_018047090.1, XM_018047096.1, XM_018061205.1, XM_018040931.1, XM_018038705.1, XM_005688391.2, XM_018039502.1, XM_013964068.2, XM_018039816.1, XM_018040925.1, XM_018048241.1, XM_018049792.1, XM_018051454.1, XM_018040493.1, XM_018040587.1, XM_018038794.1, XM_018052556.1, XM_018065915.1, XM_018058810.1, XM_018060062.1, XM_018054271.1, XM_018044635.1, XM_018049019.1, XM_018056326.1, XM_005678299.3, XM_018046369.1, XR_001919350.1, XM_018047736.1, XM_018054477.1, XM_005680368.3, XM_005693111.3, XM_018048250.1, XM_018051788.1, XM_018046371.1, XM_018041190.1, XM_005675354.3, XM_018044831.1, XM_018049703.1, XM_018065520.1, XM_018040638.1, XM_018050929.1, XM_018049341.1, XM_018043079.1, XM_018048243.1, XM_018051456.1, XM_018038702.1, XM_018039861.1, XM_018058924.1, XM_005693264.3, XM_018041080.1, XM_018046078.1, XM_018052391.1, XM_018065891.1, XM_018040516.1, XM_018048245.1, XM_018055602.1, XM_018049705.1, XM_018039462.1, XM_018061581.1, XM_018051194.1, XM_018064579.1, XM_018040550.1, XM_018053321.1, XM_018066929.1, XM_013964078.2, XM_018044608.1, XM_005680937.3, XM_005688299.3, XM_018049339.1, XM_018040614.1, XM_018040930.1, XM_018040523.1, XM_018048242.1, XM_018062604.1, XM_005676582.3, XM_018059467.1, XR_001919089.1, XM_018058077.1, XM_018048285.1, XM_018040482.1, XM_018062387.1, XM_018049860.1, XM_018040446.1, XM_018038798.1, XM_018048248.1, XR_001919737.1, XM_018049952.1, XM_018046074.1, XM_018044832.1, XR_001917934.1, XM_018059034.1, XM_018063683.1, XM_018061746.1, XM_018051457.1, XR_311259.3, XM_018058390.1, XM_018038703.1, XM_018052492.1, XM_005694016.2, XM_018040326.1, XM_018062589.1, XM_005698355.2, XR_001919481.1, XM_018049706.1, XM_018049701.1, XM_018040465.1, XM_018065059.1, XM_018063801.1, XM_018058389.1, XM_018053743.1, XM_018040542.1, XM_013968710.2, XM_018061302.1, XM_018049862.1, XM_005686332.3, XM_018042156.1, XM_018043642.1, XM_018055025.1, XM_005698354.3, XM_005700115.3, XM_018061582.1, XM_018038708.1, XM_018040411.1, XM_018049859.1, XM_005694015.2, XM_018057082.1, XM_018040533.1, XM_018048734.1, XM_018040927.1, XR_001295577.2, XM_018048549.1, XM_005685969.3, XM_018043036.1, XM_018055747.1, XM_005680607.3, XR_001917485.1, XM_005680608.3, XM_018048249.1, XM_018047257.1, XM_005680611.3, XM_005696787.3, XM_018046786.1, XM_018040457.1, XM_018058697.1, XM_018050156.1, XM_018055369.1, XM_018040474.1, XM_018067122.1, XM_018061662.1, XM_018046227.1, XM_018048224.1, XM_013965507.2, XM_013962921.2, XM_018049736.1, XM_018042765.1, XM_018041133.1, XM_018049791.1, XM_005676458.3, XM_018058387.1, XM_018052065.1, XM_013972667.2, XM_018040421.1, NM_001291820.1, XM_018038796.1, XM_018040623.1, XM_005685851.3, XM_018061533.1, XM_018063150.1, XM_018040337.1, XM_018047254.1, XM_018040578.1, XM_018039463.1, XM_018049707.1, XM_018040929.1, XM_018060820.1, XM_005692564.3, XM_005684278.2, XM_018054878.1, XM_018043080.1, XM_018040906.1, XM_018053324.1, XM_005692692.3, XM_005693262.3, XM_005677582.3, XM_018062603.1, XR_001917484.1, XM_018052032.1, XM_018042024.1, XM_018064237.1, XM_018047843.1, XM_005709596.3, XM_018051789.1, XM_018058646.1, XM_018040502.1, XM_018042796.1, XM_018048246.1, XM_018055757.1, XM_018049342.1, XM_018049951.1, XM_018065605.1, XM_018039695.1, XM_005684658.2, XM_018040607.1, XM_018039556.1, XM_018040244.1, XR_001917935.1, XM_018059720.1, NM_001285577.1, XM_005693263.3, XM_018058388.1, XM_018064350.1, XM_013964076.2, XM_018046226.1, XM_018040566.1, XM_013975998.2, XM_013962880.2, XM_018046784.1, XM_018050940.1, XM_018039193.1, XM_018043927.1, XM_018059125.1, XM_018048251.1, XM_018051206.1, XM_018053225.1, XM_018061745.1, XM_018049704.1, XM_018049702.1, XM_005680662.3, XM_005687163.3, XM_018065503.1, XM_005681688.3, XM_018040928.1, XM_018042095.1, XM_018043293.1, XM_018061580.1, XM_018045244.1, XM_018062434.1, XM_018048252.1, XM_018053320.1, XM_005680829.3, XM_018043784.1, XM_005676715.3, XR_001918036.1, XM_018049861.1, XM_018056393.1, XM_018056932.1, XM_018049340.1, XM_018048743.1, XM_005675352.3, XM_018040511.1, XM_018064970.1, XM_018067123.1, XM_005693110.2, XM_018060064.1, XM_005675809.3, XM_018043081.1, XM_018056721.1, XM_005678058.3, XM_018040440.1, XM_018038706.1, XM_018052563.1, XM_018042023.1, XM_013972225.2, XM_018063375.1, XM_013972226.2, XM_018048247.1, XM_018064131.1, XM_018048255.1, XM_018040554.1, XR_001919090.1, XM_018044236.1, XM_018040574.1, XM_018067124.1, XM_018040600.1, XM_018040924.1, XM_018048550.1, XM_018065918.1, XM_018057758.1, XM_018061303.1, XM_018044607.1, XM_018059468.1, XM_018048254.1, XR_001917130.1, XM_018049789.1, XM_018053323.1, XM_018038709.1, XM_005698279.2, XM_005679729.3, XM_005686075.3, XM_018048240.1, XM_018039191.1, XR_001919517.1, XM_018051453.1, XM_018053392.1, XM_005698156.2, XM_018046815.1, XM_018065540.1, XM_018039465.1, XM_018038795.1, XM_018050867.1, XM_018054731.1, XM_018043078.1, XM_018052582.1, XM_005686905.3 |
| 89 | [Fc gamma R-mediated phagocytosis](../../../AppData/Local/Temp/Temp1_VETR-D-18-00038.zip/Cont-vs-PPRV_DEGseq_map/map04666.html) | XM_005678301.3, XM_005700482.2, XM_005676457.3, XM_018047903.1, XM_005681861.3, XM_018043641.1, XM_018049587.1, XM_018038704.1, XM_018062722.1, XM_013965682.2, XM_005681449.3, XM_018061294.1, XM_018051455.1, XM_018065386.1, XM_018065920.1, XM_018060694.1, XM_013964768.2, XR_001920008.1, XM_005686929.1, XM_018056451.1, XM_005679127.2, XM_018044120.1, XM_005687164.3, XM_018062382.1, XM_005681344.3, XM_018063971.1, XM_018047786.1, XM_018059721.1, XM_005700749.3, XM_018038707.1, XM_018061293.1, NM_001285755.1, XM_018046075.1, XM_013970381.2, XM_018065768.1, XM_018063035.1, XM_018044119.1, XM_018065385.1, XM_018039464.1, XM_018047734.1, XM_018053461.1, XM_018055821.1, XM_018055911.1, XM_018057743.1, XM_018066315.1, XM_005676159.3, XM_018066966.1, XM_018041498.1, XM_018047090.1, XM_013967930.2, XM_005697610.3, XM_018047096.1, XM_018061205.1, XM_005683110.3, XM_018047989.1, XM_018055187.1, XM_018050987.1, XM_018049782.1, XM_018062714.1, XM_018038705.1, XM_005688391.2, XM_018039502.1, XM_005685130.3, XM_018049780.1, XM_018061289.1, XM_018039816.1, XM_018054459.1, XM_018049781.1, XM_018049792.1, XM_018051454.1, XM_018060378.1, XM_018051619.1, XM_018054460.1, XM_018038794.1, XM_018052556.1, XM_018044121.1, XM_018065915.1, XM_018058810.1, XM_018054271.1, XM_018060016.1, XM_018049019.1, XM_018043534.1, XM_018056326.1, XM_005678299.3, XM_018061295.1, XM_018046369.1, XM_018049787.1, XM_018048959.1, XM_018066967.1, XM_018047736.1, XM_018054477.1, XM_005680368.3, XM_018062407.1, XM_018049785.1, XM_005693111.3, XM_018062408.1, XM_018062720.1, XM_018049788.1, XM_018043792.1, XM_018051788.1, XM_018053228.1, XM_018046371.1, XM_018041190.1, XM_018042428.1, XM_005675354.3, XM_005676376.3, XM_018043463.1, XM_018065520.1, XM_018062713.1, XM_018054461.1, XM_018047802.1, XR_001918038.1, XM_018049341.1, XM_018051456.1, XM_013974872.2, XM_018038702.1, XM_018039861.1, XM_018062724.1, XM_018058924.1, XM_005687933.3, XM_018041080.1, XM_018046078.1, XM_018047231.1, XM_018052391.1, XM_018065891.1, XM_018055602.1, XM_018050527.1, XM_018053460.1, XM_018039462.1, XM_018061581.1, XM_018051194.1, XM_005697888.3, XM_005677615.3, XM_018064579.1, XM_018053321.1, XM_018042282.1, XM_018066929.1, XM_018044608.1, XM_005680937.3, XM_005699064.3, XM_018049339.1, XM_013965681.2, XM_018043396.1, NM_001286443.1, XM_018062721.1, XM_018061292.1, XM_018062604.1, XM_018059467.1, XR_001919089.1, XM_018058077.1, XM_005699065.3, XM_018048285.1, XM_018050610.1, XM_018062387.1, XM_018049860.1, XM_018038798.1, XR_001919737.1, XR_001918229.1, XM_018046074.1, XR_001917934.1, XM_018062410.1, XM_005678056.3, XM_005675490.2, XM_018063683.1, XM_013968554.2, XM_018061746.1, XM_018051622.1, XM_018051457.1, XR_311259.3, XM_018058390.1, XM_018038703.1, XM_005698535.3, XM_018040326.1, XM_005700661.3, XM_018062589.1, XM_018058447.1, XM_005684782.3, XM_013964590.2, XM_018040436.1, XR_001919481.1, XM_018042871.1, XM_018060376.1, XM_018054462.1, XM_005681863.3, XM_018065059.1, XM_018063801.1, XM_018060738.1, XM_005679129.3, XM_018053462.1, XM_018058389.1, XM_018053743.1, XM_018054206.1, XM_005677614.3, XM_018042281.1, XM_013968710.2, XM_005700662.3, XM_018049689.1, XM_018061302.1, XM_018049862.1, XM_005691412.3, XM_005686332.3, XM_018042156.1, XM_013968553.2, XM_018043642.1, XM_005675289.3, XM_018055025.1, XM_018048956.1, XM_018065817.1, XM_005700115.3, XM_018048018.1, XM_018061582.1, XM_018038708.1, XM_018049858.1, XM_018049859.1, XM_018044122.1, XM_018043794.1, XM_018043791.1, XM_018059609.1, XM_018057082.1, XM_013965680.2, XM_018048734.1, XM_013964769.2, XM_018042429.1, XM_005678357.3, XM_018048549.1, XM_018043036.1, XM_018055747.1, XM_018064745.1, XM_018061554.1, XM_018047257.1, XM_005696787.3, XM_013965684.2, XM_018061288.1, XM_018048019.1, XM_018046786.1, XM_018058697.1, XM_018052715.1, XM_018055369.1, XM_018063036.1, XM_005680258.3, XM_018058397.1, XM_018067122.1, NM_001314202.1, XM_018059489.1, XM_018060488.1, XM_018061662.1, XM_018046227.1, XM_018060739.1, XM_018052324.1, XM_018042279.1, XM_005683717.3, XM_013967227.2, XM_018048224.1, XM_013965507.2, XM_018057198.1, XM_018048952.1, XM_013962921.2, NM_001285641.1, NM_001285750.1, XM_018049736.1, XM_018042765.1, XM_018041133.1, XM_018049791.1, XM_005676458.3, XM_005681533.3, XM_018058387.1, XM_018055908.1, XM_005701006.3, XM_018049691.1, XM_018052065.1, XM_018050833.1, XM_013972667.2, NM_001291820.1, XM_018038796.1, XM_018062409.1, XM_018053966.1, XM_005685851.3, XM_018061533.1, XM_018040337.1, XM_005675639.3, XM_018047254.1, XM_018050856.1, XM_018039463.1, XM_018042278.1, XM_018060820.1, XM_018064986.1, XM_005685763.3, XM_018062710.1, XM_005693259.3, XM_018062725.1, XM_018066938.1, XM_005692564.3, XM_013965679.2, XM_018046304.1, XM_018054878.1, XM_018057897.1, XM_018040906.1, XM_018053324.1, XM_018065816.1, XM_018055186.1, XM_018054083.1, XM_018042280.1, XM_013975781.2, XM_018062603.1, XM_018052032.1, XM_018042024.1, XM_018064237.1, XM_005709596.3, XM_018051789.1, XM_018060379.1, XM_018058646.1, XM_018054611.1, XM_018050834.1, XM_005680676.3, XM_018055757.1, XM_018049342.1, XM_018061830.1, XM_018049951.1, XM_005675640.3, XM_018065605.1, XM_018039695.1, XM_005684658.2, XM_018049783.1, XM_018040244.1, XR_001917935.1, XM_018059720.1, XR_001918230.1, XM_013965683.2, XM_013970271.2, XM_018058388.1, XM_018064350.1, XM_018049786.1, XM_018063749.1, XM_018046226.1, XM_013962880.2, XM_018046784.1, XM_018060838.1, XM_018047366.1, XM_018039193.1, XM_018043927.1, XM_005677617.3, XM_018059125.1, XM_013968877.2, XM_005678171.3, XM_018051206.1, XM_005675332.3, XM_005685146.3, XM_018053225.1, XM_018061745.1, XM_018064672.1, XM_005681862.2, XM_005688564.3, XM_018060261.1, XR_001919756.1, XM_005680662.3, XM_018058396.1, XM_018048951.1, XM_018049857.1, XM_018058395.1, XM_018064988.1, XM_018049721.1, XM_013975227.2, XM_005683147.3, XM_018053831.1, XM_005687163.3, XM_018065503.1, XM_005681688.3, XM_018064671.1, XM_018043395.1, XM_018042095.1, XM_013964774.2, XM_018064746.1, XM_018043293.1, XM_018061580.1, XM_018064499.1, XM_018050609.1, XM_018062712.1, XR_001918228.1, XM_018045244.1, XM_018048420.1, XM_018061291.1, XM_018062718.1, XM_018062434.1, XM_018060377.1, XM_018046856.1, XM_018053320.1, XM_018038791.1, XM_018062723.1, XM_018043793.1, XM_005680829.3, XM_005694472.3, XM_018043784.1, XM_018060089.1, XM_018065433.1, XM_018062717.1, XM_018049861.1, XM_018061286.1, XM_018061071.1, XM_013965671.2, XM_018056393.1, XM_018049340.1, XM_018048743.1, XM_005675352.3, XM_013964770.2, XM_018064970.1, XM_018049779.1, XM_018067123.1, XM_005693110.2, XM_018053830.1, XM_018058394.1, XM_005675809.3, XM_018056721.1, XM_005678058.3, XM_005678170.3, XM_018038706.1, XM_018055912.1, XM_018052563.1, XM_018062711.1, XM_018042023.1, XM_018063375.1, NM_001285685.1, XM_018064131.1, XM_018063972.1, XM_018067051.1, XR_001919090.1, XM_018065351.1, XM_018062719.1, XM_018067124.1, XM_005680013.2, XM_018048550.1, XM_018065918.1, XM_018063570.1, XM_018064292.1, XM_018057758.1, XM_018061303.1, XM_018044607.1, XM_018059468.1, XR_001917130.1, XM_018049789.1, XM_013968876.2, XM_018038788.1, XM_018061555.1, XM_018053323.1, XM_018041495.1, XM_018053231.1, XM_018038709.1, XM_013965678.2, XM_005679729.3, XM_005686075.3, XM_018043795.1, XM_018039191.1, XM_005683146.3, XR_001919517.1, XM_005685953.3, XM_018051453.1, XM_018054431.1, XM_018049784.1, XM_018053392.1, XM_018046815.1, XM_018039465.1, XM_005685145.3, XM_018038795.1, XM_005699502.2, XM_018050867.1, XM_018054731.1, XR_001918643.1, XM_018052582.1, XM_005686905.3, XM_018053232.1 |
| 90 | [Terpenoid backbone biosynthesis](../../../AppData/Local/Temp/Temp1_VETR-D-18-00038.zip/Cont-vs-PPRV_DEGseq_map/map00900.html) | XM_018043131.1, XM_005677388.3, XM_005694740.3, XR_001917487.1, XM_005676812.3, XM_018043121.1, XM_013974097.2, XM_018056411.1, XM_018061103.1, XM_018043125.1, XM_018053432.1, XM_005691582.3, XM_018053021.1, XM_018043124.1, XM_018043123.1, XM_005677386.3, XM_005678642.3, XM_018043129.1, XM_018043119.1, NM_001285740.1, XM_005694739.3, XM_018043127.1, XM_018043133.1, XM_018065309.1, XM_018050178.1, XM_018043126.1, XM_005677390.3, XM_018043118.1, XM_018061849.1, XM_005691583.3, XM_018055468.1, XR_001919148.1, XM_018043130.1, XM_018043128.1, XM_018060445.1, XM_018053703.1 |
| 91 | [Prostate cancer](../../../AppData/Local/Temp/Temp1_VETR-D-18-00038.zip/Cont-vs-PPRV_DEGseq_map/map05215.html) | XM_018055645.1, XM_005681861.3, XM_018055614.1, XM_018043204.1, XM_018064273.1, XM_013964768.2, XM_018065097.1, XM_013962777.2, XM_018056824.1, XM_005679127.2, XM_018063971.1, NM_001285748.1, XM_018063035.1, XM_018055911.1, XM_005697610.3, XM_018055187.1, XM_018050987.1, XM_005685130.3, XM_013972144.2, XM_018060378.1, XM_018051619.1, XM_018047426.1, XM_005680266.3, XM_018060016.1, XM_018060051.1, XM_005692453.3, XM_018048959.1, XM_018062407.1, XM_018062408.1, XM_018053228.1, XM_018042428.1, XM_018058171.1, XM_005681590.3, XM_013974872.2, XM_018047231.1, XM_018058500.1, XM_005679654.2, XM_005676377.3, XM_013976502.2, XM_018056811.1, XM_018062410.1, XM_013968554.2, XM_018051622.1, XM_005694016.2, XM_005689267.3, XM_018058447.1, XM_005684782.3, XM_013963116.2, XM_018060376.1, XM_018048184.1, XM_005681863.3, XM_005679129.3, XM_018064279.1, XM_018054206.1, XM_018065969.1, XM_018063773.1, XM_018049689.1, XM_005700145.2, XM_013968553.2, XM_005675289.3, XM_018048956.1, XM_018065817.1, XM_005694015.2, XM_018059609.1, XM_018047703.1, XM_013964769.2, XR_001295577.2, XM_018042429.1, XM_018056690.1, XM_018064745.1, XM_018047425.1, XM_018063036.1, NM_001314202.1, XM_018060488.1, XM_018049427.1, XM_018048812.1, XM_013967227.2, XM_018048952.1, NM_001285750.1, XM_018055908.1, XM_018049691.1, XM_018060052.1, XM_013972145.2, XM_018062409.1, XM_005686996.3, XM_018059919.1, XM_018066613.1, XM_018050856.1, XM_018039227.1, XM_005674982.3, XM_018039226.1, XM_018064276.1, XM_005685763.3, XM_018065816.1, XM_018055186.1, XM_018054083.1, XM_018045207.1, XM_005683226.3, XM_018060379.1, XM_013963900.1, XM_005680373.3, XM_018058634.1, XM_013970271.2, XM_005678307.2, XM_018040360.1, XM_013968877.2, XM_005675332.3, XM_005685146.3, XM_013971256.2, XM_018064672.1, XM_005681862.2, XM_005688564.3, XM_018060261.1, XM_018058170.1, XM_018048951.1, XM_018058502.1, XM_018044357.1, XM_018064671.1, XM_013964774.2, XM_018064746.1, XM_018048420.1, XM_018060377.1, XM_018038791.1, XM_005694472.3, XM_005676715.3, NM_001285608.1, XM_013964770.2, XM_018055912.1, XM_018047444.1, XM_018043664.1, XM_013972225.2, XM_013972226.2, XM_018067051.1, XM_018059918.1, XM_018064278.1, XM_018047424.1, XM_005676378.3, XM_018064292.1, XM_018064277.1, XM_018038788.1, XM_018053231.1, XM_018045593.1, XM_018043122.1, XM_018064274.1, XM_005685145.3, XM_018053232.1 |
| 92 | [Renin secretion](../../../AppData/Local/Temp/Temp1_VETR-D-18-00038.zip/Cont-vs-PPRV_DEGseq_map/map04924.html) | XM_018062643.1, XM_005684795.2, XM_013963001.2, XM_018061124.1, NM_001314245.1, XM_018039549.1, XM_018052298.1, XM_005695088.3, XM_005680366.2, XM_018052301.1, XM_018042311.1, XM_018055980.1, XM_018054045.1, XM_018053453.1, XR_001918100.1, XM_005680737.3, XM_005697075.3, XM_018058454.1, XM_005691447.3, XM_018066370.1, XM_018066401.1, XM_018051778.1, XM_018047856.1, XM_005682114.3, XM_013972607.2, XM_018051048.1, XM_005680364.3, XM_005684794.3, XM_005694678.3, XM_018057419.1, XM_005699952.3, XM_018042310.1, XM_013972972.2, XM_018041323.1, NM_001285674.1, XM_018054634.1, XM_018058066.1, XM_018066400.1, XM_018061125.1, XM_018049335.1, XM_018047050.1, XM_005691313.3, XM_018057425.1, XM_005680365.2, XM_018045751.1, XM_018042308.1, XM_018039561.1, XM_005694677.3, XM_005691312.2, XM_018061944.1, XM_018065511.1, XM_005688518.3, XM_018041016.1, XM_005694675.3, XM_018061941.1, XM_018045231.1, XM_018055439.1, XM_018043047.1, XM_018052297.1, XM_018051049.1, XM_005679333.3, XM_018066184.1, XM_018066186.1, XM_018042307.1, XM_018066371.1, XM_018051046.1, XM_018049271.1, XM_018057813.1, XM_018052300.1, XM_018064036.1, XM_013970799.2, XM_018047636.1, XM_018046143.1, XM_018051050.1, XM_005693569.2, XM_018043593.1, XR_001295516.2, XM_018065622.1, XM_018065509.1, XM_005694519.3, XM_018061942.1, XM_005680367.3, XM_018061940.1, XM_005676485.3, XM_005685150.3, XM_018055663.1, XM_013964453.2, XM_005686574.3, XM_013966779.2, XM_005681818.3, XM_005684793.3, XM_018054635.1, XM_018048449.1, XM_018042309.1, XM_005685503.3, XM_005682112.2, XM_018058573.1, XM_018044096.1, XR_001919417.1, XM_018057412.1, XM_018041327.1, XM_013966645.2, XM_013963332.2, XM_018061943.1, XM_018065510.1, XM_005681464.3, XR_001296033.2, XM_018057613.1, XM_018054044.1, XM_005676482.3, XM_018041325.1, XM_018049270.1, XM_005685149.3, XM_018045239.1, XM_018058455.1, XM_018042304.1, XM_005686948.3, XM_018042305.1, XM_005697074.3, XM_018066185.1, XM_018047638.1, XM_005688957.3, XM_005695085.3, XM_018053454.1, XM_005675916.3, XM_005675891.3, XM_018042303.1, XM_013976047.2, XM_018055664.1, XM_018055665.1, XM_005694349.2, XM_005699951.1, XM_018047637.1, XM_005678304.3 |
| 93 | [Phosphatidylinositol signaling system](../../../AppData/Local/Temp/Temp1_VETR-D-18-00038.zip/Cont-vs-PPRV_DEGseq_map/map04070.html) | XM_018062643.1, XM_013963001.2, XM_018053411.1, XM_018061124.1, XM_018066660.1, XM_013962777.2, XR_001920008.1, XM_005679127.2, XM_018044194.1, XM_005680381.3, XM_018047503.1, XM_013965804.2, XM_005680366.2, XM_018056109.1, XM_018064091.1, XM_018066242.1, XM_018067183.1, XM_018054045.1, XM_018044591.1, XM_018047363.1, XM_018041498.1, XM_013967930.2, XM_013962450.2, XR_001918100.1, XM_005680737.3, XM_018060998.1, XM_005697075.3, XM_005691447.3, XM_018066370.1, XM_013971383.2, XM_018050170.1, XM_018060378.1, XM_013972607.2, XM_018056110.1, XM_005680364.3, XM_018060016.1, XM_005699952.3, XM_018059408.1, XM_018048614.1, XM_018044193.1, XM_005695985.3, XM_005680805.2, XM_013963902.2, XM_005676376.3, XM_005677609.3, XM_018065259.1, XM_018046894.1, XM_018060165.1, XM_018061125.1, XM_005687933.3, XM_018047231.1, XM_013965431.2, XM_018059872.1, XM_005697888.3, XM_005677615.3, XM_018047528.1, XM_005680365.2, XM_018047310.1, XM_018056916.1, XM_018053412.1, XM_018048105.1, XM_018047514.1, XM_018066248.1, XM_018056914.1, XM_018039561.1, XM_018060957.1, XM_018047360.1, XM_005695950.3, XM_018066661.1, XM_005678985.3, XM_005688518.3, XM_018041016.1, XM_018060376.1, XM_018047364.1, XM_018041918.1, XM_005679129.3, XM_018059162.1, XM_018056111.1, XM_018056566.1, XM_005677614.3, XM_018043047.1, XM_013965802.2, XM_005683464.3, XM_005675289.3, XM_013968410.2, XM_018048018.1, XM_018056913.1, XM_005679333.3, XM_018059609.1, XM_018047703.1, XM_018042789.1, XM_018041075.1, XM_005693310.3, XM_018056791.1, XM_018059528.1, XM_018061554.1, XM_018066371.1, XM_018048019.1, XM_018056794.1, XM_018056108.1, XM_018059164.1, XM_018057813.1, XM_018044352.1, XM_005696969.3, XM_005683466.3, XM_018056792.1, XM_018058397.1, XM_018066241.1, XM_018041370.1, XM_005683717.3, XM_005677610.3, XM_018065262.1, XM_013970799.2, XM_018060995.1, XM_018066175.1, XM_018050833.1, XM_018060997.1, XM_018043593.1, XM_018059160.1, XM_018039298.1, XM_018052493.1, XR_001295516.2, XM_018056910.1, XM_005675639.3, XM_018060994.1, XM_018064986.1, XM_018059166.1, XM_005680367.3, XM_018057897.1, XM_005676485.3, XM_018056908.1, XM_018056107.1, XM_018041036.1, XM_005685150.3, XM_018041615.1, XM_018045207.1, XM_018061000.1, XM_005686574.3, XM_013966779.2, XM_018060379.1, XM_018054611.1, XM_018061765.1, XM_018050834.1, XM_018061830.1, XM_005675640.3, XM_018066662.1, XM_018056911.1, XM_013965807.2, XM_018045214.1, XM_005678307.2, XM_018049995.1, XM_013963165.2, XM_018060838.1, XM_005677617.3, XM_005676315.3, XM_018047359.1, XM_018046893.1, XM_018058396.1, XM_018044380.1, XR_001917180.1, XM_018058395.1, XM_018064988.1, XM_005678984.3, XM_018041043.1, XM_018047413.1, XM_018056106.1, XM_018044357.1, XM_018048620.1, XR_001296033.2, XM_013971389.2, XM_018041369.1, XM_005679831.3, XM_018048420.1, XM_005680383.3, XM_018060377.1, XM_018048104.1, XM_018059161.1, XM_013965808.2, XM_018057365.1, XM_018047458.1, XM_018054044.1, XM_018059165.1, XM_005693177.3, XM_013973615.2, XM_005676482.3, XM_018066085.1, XM_018060999.1, XM_018058394.1, XM_018040956.1, XM_018049946.1, XM_018044590.1, XM_018048609.1, XM_005685149.3, XM_005679832.3, XM_018060996.1, XM_013974301.2, XM_018059167.1, XM_005697074.3, XM_018059163.1, XM_013971403.2, XM_018054425.1, XM_018039299.1, XM_018065261.1, XM_018054006.1, XM_018041050.1, XM_005683465.3, XM_018060186.1, XM_018061555.1, XM_013963166.2, XM_018065997.1, XM_018041495.1, XM_013976047.2, XM_005696968.3, XM_018056915.1, XM_013965806.2, XM_018065668.1, XM_005694349.2, XM_005699951.1, XM_018066086.1 |
| 94 | [Arginine and proline metabolism](../../../AppData/Local/Temp/Temp1_VETR-D-18-00038.zip/Cont-vs-PPRV_DEGseq_map/map00330.html) | XM_005699184.3, XM_018064736.1, NM_001291699.1, XM_005682625.3, XM_018039542.1, XM_005683753.3, XM_018044859.1, XM_018054382.1, XM_018064737.1, XM_018059356.1, XM_018050730.1, XM_018050116.1, XM_013965331.2, XM_005709669.3, XM_018053073.1, XM_018065148.1, XR_001917214.1, XM_018041531.1, XM_018065154.1, XM_018041548.1, XM_018052863.1, XM_013972176.2, XM_018044856.1, NM_001291698.1, XM_013971563.2, XM_018065153.1, XM_018041532.1, NM_001293756.1, XM_018039687.1, XM_013966479.2, XM_018039689.1, XM_018044860.1, XM_018066322.1, XM_013972175.2, XM_018060222.1, XM_005691592.3, XM_005691559.3, XM_005679653.3, XM_005693870.3, XM_018042528.1, XM_018042906.1, XM_018064735.1, XM_005698316.3, XM_018066757.1, XM_018054270.1, XM_018050728.1, XM_018044693.1, XM_013965542.2, XM_013965333.2, XM_018048201.1, XM_018041265.1, XM_018057378.1, XM_018041545.1, XM_018059357.1, XM_018066948.1, XM_018066949.1, XM_018054760.1, XM_018041547.1, XM_005682692.3, XM_018064739.1, XM_018050726.1, XM_005692097.3, XM_018060223.1, XM_018039688.1, NM_001291697.1, XM_018050727.1, XM_018041546.1, XM_018065441.1, XM_018059355.1, XM_018065147.1, XM_018064738.1, XM_018041266.1, XM_018042527.1, XM_018039543.1, XM_018042905.1, XM_018057379.1, XM_018044854.1, NM_001285705.1, XM_018057380.1, XM_018039544.1, XM_005694051.2, XM_018050729.1 |
| 95 | [Long-term depression](../../../AppData/Local/Temp/Temp1_VETR-D-18-00038.zip/Cont-vs-PPRV_DEGseq_map/map04730.html) | XM_005681861.3, XM_018064273.1, XM_013964768.2, XM_018039549.1, XM_018056824.1, XM_018054409.1, XR_001918100.1, XM_018050987.1, XM_005680737.3, XM_005685130.3, XM_018051619.1, XM_018047856.1, XM_018048959.1, NM_001285674.1, XM_018042428.1, XM_005681590.3, XM_018054634.1, XM_018059802.1, XM_018064706.1, XM_018058500.1, XM_005698163.3, XM_018047050.1, XM_005691313.3, XM_018054401.1, XM_005690990.2, XM_018045751.1, XM_018060732.1, XM_005691312.2, XM_018061944.1, XM_018056811.1, XM_018042061.1, XM_018051622.1, XM_018061941.1, XM_018059800.1, XM_005681863.3, XM_018064279.1, XM_018054206.1, XM_018049689.1, XM_005700145.2, XM_018048956.1, XM_018054405.1, XM_013964769.2, XM_018042429.1, XM_018064745.1, XM_018054406.1, XM_018064036.1, XM_005675953.3, NM_001314202.1, XM_018049427.1, XM_018048952.1, XM_018046143.1, XM_018049691.1, XM_018059799.1, XM_018065622.1, XM_018050856.1, XM_018064276.1, XM_005685763.3, XM_018061942.1, XM_018061940.1, XM_018054402.1, XM_005683226.3, XM_018054408.1, XM_005681818.3, XM_018054635.1, XM_018048449.1, XM_005680373.3, XM_018058573.1, XM_018044096.1, XM_018054403.1, XR_001919417.1, XM_005678171.3, XM_005685146.3, XM_018064672.1, XM_005681862.2, XM_018048951.1, XM_018058502.1, XM_018061943.1, XM_005685526.3, XM_018064671.1, XM_013964774.2, XM_018064746.1, XM_018057613.1, XM_018038791.1, XM_005694472.3, XM_013964770.2, XM_018041140.1, XM_005678170.3, XM_018054407.1, XM_018041138.1, XM_018064278.1, XM_005686948.3, XM_018061406.1, XM_018064292.1, XM_018064277.1, XM_018038788.1, XM_018045593.1, XM_018041139.1, XM_018064274.1, XM_018047883.1, XM_005685145.3 |
| 96 | [Small cell lung cancer](../../../AppData/Local/Temp/Temp1_VETR-D-18-00038.zip/Cont-vs-PPRV_DEGseq_map/map05222.html) | XM_005678936.3, XM_018055645.1, XM_018038312.1, XM_018051874.1, XM_005676250.3, XM_018055614.1, XM_018054194.1, XM_018052232.1, XM_018038313.1, XM_018052664.1, XM_013962777.2, XM_005679127.2, XM_018058815.1, XM_018040741.1, XM_018063971.1, XM_018063035.1, XM_018055911.1, XM_005697610.3, XM_018055187.1, XM_005674830.2, XM_013972144.2, XM_018038275.1, XM_005680115.3, XM_018060378.1, XM_018044169.1, XM_005700226.2, XM_018047426.1, XM_005680266.3, XM_005693225.3, XM_018060016.1, XM_018060051.1, XM_005692453.3, XM_018062407.1, XM_018062408.1, XM_018041048.1, XM_005681905.3, XM_005699260.3, XM_018053228.1, XM_018056363.1, XM_018064893.1, XM_018058171.1, XM_005683247.3, XM_018040727.1, XM_018041576.1, XM_005688053.3, XM_013974872.2, XM_018038561.1, XM_018047231.1, XM_018050567.1, XM_018052213.1, XM_018045691.1, XM_018058140.1, XM_018044065.1, NM_001287573.1, XM_005682449.3, XM_005677418.2, XM_005693227.3, XM_005681906.3, XM_018038558.1, XM_018041394.1, XM_018042470.1, XM_018040698.1, XM_005679654.2, XM_005676377.3, XM_013976502.2, XM_018052661.1, XM_018062410.1, XM_013963034.2, XM_018058129.1, XM_005676883.3, XM_013968554.2, XM_005689267.3, XM_018058849.1, XM_018058447.1, XM_005684782.3, XM_013963116.2, XM_018060376.1, XM_018054322.1, XM_005679129.3, XM_018038276.1, XM_018063773.1, XM_005675868.3, XM_005675709.3, XM_013973685.2, XM_013968553.2, XM_005675289.3, XM_018044493.1, XM_018065817.1, XR_001919179.1, XM_018048340.1, XM_018059609.1, XM_018047703.1, XM_018057521.1, XM_018052663.1, XM_018052870.1, XM_018061035.1, XM_018056690.1, XM_005675869.3, XM_018052662.1, XM_018052932.1, XM_018052867.1, XM_018057501.1, XM_018047425.1, XM_018063036.1, XM_005681903.3, XM_018058855.1, XR_001917959.1, XM_018060488.1, XM_013967227.2, XM_018060763.1, NM_001285750.1, XM_018058143.1, XM_018055908.1, XM_005675149.3, XM_018060052.1, XM_013972145.2, XM_018062409.1, XM_005686996.3, XM_005676887.3, XM_018039227.1, XM_018039226.1, XM_018038559.1, XM_005694793.3, XM_005693222.3, XM_018052201.1, XR_001296591.2, XM_018042567.1, XM_018041569.1, XM_018058814.1, XM_018065816.1, XM_005700225.3, XM_005693223.3, XM_018055186.1, XR_001918483.1, XM_018054083.1, XM_018045207.1, XM_018054196.1, XM_018058852.1, XM_018060379.1, XM_018042468.1, XM_018056198.1, XM_018058141.1, XM_013963900.1, XM_005676249.3, XM_018052331.1, XM_013974500.2, XM_018038560.1, XM_013970271.2, XM_018048341.1, XM_005678307.2, XM_018042467.1, XM_013968877.2, XM_018052941.1, XM_005675332.3, XM_013971256.2, XM_005688564.3, XM_018059661.1, XM_018060261.1, XM_018058170.1, XM_005678025.3, XM_013975194.2, XM_018056364.1, XM_018040712.1, XM_018044357.1, XM_018059663.1, XM_018044170.1, XM_018056863.1, XM_018048420.1, XM_018060377.1, XM_018057493.1, XM_018047868.1, XM_018042469.1, XM_018044495.1, XM_018042471.1, XM_005684236.3, XM_018061896.1, NM_001285608.1, XM_013972845.2, XM_018040719.1, XM_005695770.3, XM_005694346.3, XM_005701390.3, XM_018051009.1, XM_018053042.1, XM_018055912.1, XM_018038562.1, XM_018047444.1, XM_018043664.1, XM_018052339.1, XM_018064895.1, XM_018067051.1, XM_018052868.1, XM_018058603.1, XM_018047424.1, XM_005678961.3, XM_005676378.3, XM_018041567.1, XM_005681904.3, XM_018053231.1, XM_018051010.1, XM_018060764.1, XM_005684543.2, XM_018040704.1, XM_018055450.1, XM_018053232.1 |
| 97 | [Rheumatoid arthritis](../../../AppData/Local/Temp/Temp1_VETR-D-18-00038.zip/Cont-vs-PPRV_DEGseq_map/map05323.html) | XM_005687003.3, XM_018042922.1, XM_005677656.3, NM_001314266.1, XM_005674937.3, XM_018058513.1, XM_018065122.1, XM_018059896.1, XM_018055656.1, XM_018055657.1, XM_018051782.1, XM_018055658.1, XM_018058330.1, XM_005687004.3, XM_005689361.3, XM_005683966.3, XM_018065120.1, XM_018051785.1, XM_005686382.3, XM_018051784.1, XM_005684344.3, XM_013972159.2, XM_018061368.1, XM_018065121.1, XM_018064218.1, XM_018058511.1, XM_013972160.2, XM_018051783.1, XM_018051781.1, XM_005674936.3, XM_018058512.1, XM_018061431.1, XM_005679452.3, XM_005689266.3, XM_018065123.1, XM_005685994.3, XR_001918846.1 |
| 98 | [Lysine biosynthesis](../../../AppData/Local/Temp/Temp1_VETR-D-18-00038.zip/Cont-vs-PPRV_DEGseq_map/map00300.html) | XR_001295944.2, XM_018060976.1, XM_005682692.3, XM_005683509.3, XM_013965831.2 |
| 99 | [TNF signaling pathway](../../../AppData/Local/Temp/Temp1_VETR-D-18-00038.zip/Cont-vs-PPRV_DEGseq_map/map04668.html) | XM_005681861.3, NM_001285765.1, XM_013964768.2, XM_018055854.1, XM_005679127.2, XM_018055863.1, XM_018063971.1, XM_018038998.1, XM_018063035.1, XM_018055911.1, XM_005697610.3, XM_018055187.1, XM_018050987.1, XM_005685130.3, XM_018060378.1, XM_018051619.1, XR_001918862.1, XM_018060016.1, XR_001918863.1, XM_018048959.1, XM_018060045.1, XM_018062407.1, XM_013970162.2, XM_018062408.1, XM_005679901.2, XM_018053228.1, XM_018042428.1, XM_013974872.2, XM_018047231.1, XM_018061455.1, XM_018048319.1, XM_018055855.1, XR_001918861.1, XM_018062410.1, XM_013968554.2, XM_018051622.1, XR_001918860.1, XM_018058447.1, XM_005684782.3, XM_018060376.1, XM_005681863.3, XM_005679129.3, XM_018054206.1, XM_018055857.1, XM_018049689.1, XM_013968553.2, XM_005675289.3, XM_018048956.1, XM_018065817.1, XM_018059609.1, XM_013964769.2, XM_018042429.1, XM_005690651.3, XM_018064745.1, XM_018060043.1, XM_018055858.1, XM_018063036.1, XM_013970161.2, NM_001314202.1, XM_018060488.1, XR_001296084.2, XM_013967227.2, XM_018048952.1, NM_001285750.1, XM_013966480.2, XM_018055908.1, XM_018049691.1, XM_018055864.1, XM_018062409.1, XM_018050856.1, XM_018055856.1, XM_005685763.3, XM_018065816.1, XM_018055186.1, XM_018054083.1, XM_018060379.1, XM_018055859.1, XM_013970271.2, XM_018060042.1, XM_005684675.3, XM_013968877.2, XM_005675332.3, XM_005685146.3, XM_018064672.1, XM_005681862.2, XM_005688564.3, XR_001296083.2, XM_018060261.1, XM_018048951.1, XM_018064671.1, XM_013964774.2, XM_018064746.1, XM_018048420.1, XM_018060377.1, XM_018038791.1, XM_005694472.3, XM_018055860.1, XM_013964770.2, XM_005690650.3, XM_018055912.1, XM_005675103.3, XM_018060044.1, XM_005690649.3, XM_018067051.1, XM_018064292.1, XM_018055861.1, XM_018038788.1, XM_018053231.1, XM_005685145.3, XM_018053232.1 |
| 100 | [Autophagy - animal](../../../AppData/Local/Temp/Temp1_VETR-D-18-00038.zip/Cont-vs-PPRV_DEGseq_map/map04140.html) | XM_005699593.3, XM_018044652.1, XM_013973238.2, XM_018058079.1, XM_018043283.1, XM_005681861.3, XM_018041069.1, XM_005677656.3, NM_001314266.1, XM_018061268.1, XM_005701148.3, XR_001917192.1, NM_001314245.1, XM_013964768.2, XM_013962777.2, XM_013962541.2, XM_018056451.1, XM_005679127.2, XM_018057448.1, XM_018063971.1, XM_018061046.1, NM_001285748.1, XR_001917497.1, XM_018038998.1, XM_018041065.1, XM_013970381.2, XM_018063035.1, NM_001285656.1, XM_005695367.3, XM_018053461.1, XM_018055911.1, XM_018063078.1, XM_018054628.1, XM_005697610.3, XM_005683110.3, XM_018047989.1, XM_018055187.1, XR_001918100.1, XM_018050987.1, XM_005680737.3, XM_018059746.1, XM_005700756.3, XM_018058560.1, XM_005685130.3, XR_311047.3, XM_018040963.1, XM_018060171.1, XM_018060378.1, XM_018051619.1, XM_005675055.2, XM_018051778.1, XM_005680647.3, XM_018054468.1, XM_005680005.2, XM_018057896.1, XM_018043280.1, XM_005686991.3, XM_018055124.1, XM_005699594.3, XM_018063633.1, XM_005678693.3, XM_018060016.1, XM_018061265.1, XM_018057419.1, XM_018043282.1, XM_018066234.1, XM_018048959.1, XM_018043281.1, XM_018060045.1, XM_018062407.1, XM_018043288.1, XM_018065501.1, XM_018062408.1, XM_005679901.2, XM_018043792.1, XM_018053228.1, XM_018061266.1, XM_018042428.1, XM_018043463.1, XM_018043180.1, XM_018062590.1, XM_005685995.3, XM_005693521.3, XM_018065355.1, XM_005678324.2, XM_013974872.2, XM_005675056.2, XM_018047231.1, XM_018053460.1, XM_018044746.1, XM_018044660.1, XM_018066977.1, XM_018060065.1, XM_018057740.1, XM_005693625.3, XM_018062652.1, XM_018051782.1, XM_018057894.1, XM_018057425.1, XM_005678325.3, XM_018050610.1, XM_005699431.3, XM_018043329.1, XM_018059386.1, XM_018057817.1, XR_001918229.1, XM_018062410.1, XM_013976723.2, XM_018040509.1, XM_018042061.1, XM_005675490.2, XM_013968554.2, XM_018051622.1, XM_005698535.3, XM_018052889.1, XM_018058447.1, XM_005684782.3, XM_005683386.2, XM_018042871.1, XM_018060376.1, XM_018057739.1, XM_005681863.3, XM_018053149.1, XM_018051816.1, XM_018060738.1, XM_005679129.3, XM_018053462.1, XM_018054206.1, XM_005681337.3, XM_018049689.1, XM_005681336.3, XM_013968553.2, XM_005675289.3, XM_018048956.1, XM_018065817.1, XM_018049858.1, XM_018041061.1, XM_018043794.1, XM_005695116.3, XM_018043791.1, XM_013976451.2, XM_018059609.1, XM_018057895.1, XM_018047703.1, XM_013962290.2, XM_013964769.2, XM_018066978.1, XM_018042429.1, XM_018064745.1, XM_018060043.1, XM_018045601.1, XM_018066141.1, XM_018054571.1, XM_018064220.1, XM_018051785.1, XM_005696969.3, XR_001917929.1, XM_018061924.1, XM_018061267.1, XM_018063036.1, XM_018061921.1, XM_018051501.1, NM_001314202.1, XR_001919874.1, XM_018060488.1, XR_001296084.2, XM_018060739.1, XM_013967227.2, XM_018059798.1, XM_018048952.1, NM_001285641.1, NM_001285750.1, XM_013966480.2, XM_018055349.1, XR_001917495.1, XM_018055908.1, XM_005701006.3, XM_018049691.1, XM_018051784.1, XM_005700754.3, NM_001286091.1, XR_001918278.1, XM_013963449.2, XM_018062409.1, XM_018053966.1, XM_018040510.1, XM_005678321.2, XM_013973240.2, XM_018059919.1, XM_018064432.1, XM_005695115.3, XM_005675057.2, XM_018050856.1, XM_018039742.1, XM_005702078.3, XM_005685763.3, XM_005701147.2, XM_005693259.3, XM_013970533.2, XM_005676572.3, XM_018040577.1, XM_018065816.1, XM_018055186.1, XM_018054083.1, XM_005683637.3, XM_018045207.1, XM_018040576.1, XM_018040672.1, XM_018062650.1, XM_018060379.1, XM_013962990.2, XM_013972793.2, XM_018059747.1, XM_018051760.1, XM_018058634.1, XM_018040964.1, XM_018064433.1, XM_018054630.1, XR_001918230.1, XM_013970271.2, XM_013972796.2, XM_005678307.2, XM_018056276.1, XM_005693485.3, XM_018060042.1, XM_018040360.1, XM_005684675.3, XM_018041348.1, XM_018066076.1, XM_013968877.2, XM_018055130.1, XM_018066944.1, XM_005678171.3, XM_005675332.3, XM_005685146.3, XM_018064672.1, XM_005681862.2, XM_018057412.1, XM_005688564.3, XR_001296083.2, XM_018056275.1, XM_018060261.1, XM_018062591.1, XM_018048951.1, XM_018049857.1, XR_001917180.1, XM_005695428.3, XM_005700755.3, XM_005693865.3, XM_018049721.1, XM_018053831.1, XM_018044357.1, XR_001918314.1, NM_001285594.1, XM_018064671.1, XM_005676571.3, XM_005678323.2, XM_018059745.1, XM_013964774.2, XM_018064746.1, XM_018059748.1, XM_018050609.1, NM_001287233.1, XR_001918228.1, XM_018048420.1, XM_018060377.1, XM_018054629.1, XM_018062649.1, XM_018038791.1, XM_005676438.3, XM_005678322.3, XM_018043793.1, XM_005694472.3, XM_005690407.3, XM_005695430.2, XM_018041970.1, XM_018060089.1, XM_018041744.1, XM_018044227.1, XM_018061071.1, XM_013965671.2, XM_018043284.1, XM_013964770.2, XM_018061923.1, XM_005687441.3, XM_018051783.1, XM_005684192.3, XM_018050781.1, XM_018053830.1, XM_018062651.1, XM_005678170.3, XM_018058102.1, XM_018055912.1, XM_018051781.1, XM_018043190.1, XM_005695427.3, XM_018065266.1, XM_005682905.3, XM_018060044.1, XM_013974301.2, XM_018063972.1, XM_018067051.1, XM_018059918.1, XM_018059384.1, XM_018066075.1, XM_005685359.3, XM_005691714.3, XM_018061264.1, XM_018048933.1, XM_013968393.2, XM_018061406.1, XM_018063570.1, XM_018064292.1, XM_005682904.3, XM_005695429.3, XM_013968876.2, XM_018038788.1, XM_018058103.1, XM_018050827.1, XM_018053231.1, XM_018061922.1, XM_018047553.1, XM_005676570.3, XM_018043795.1, XM_018065502.1, XM_005696968.3, XM_005695431.3, XM_005675512.3, XM_018041058.1, XM_005684610.3, XM_018054469.1, XM_018059385.1, XM_005685145.3, XM_018043287.1, XM_018061434.1, XM_018055626.1, XM_018048748.1, XR_001917496.1, XM_018048238.1, XM_018040575.1, XM_018064731.1, XR_001918643.1, XM_018053232.1 |
| 101 | [Cholinergic synapse](../../../AppData/Local/Temp/Temp1_VETR-D-18-00038.zip/Cont-vs-PPRV_DEGseq_map/map04725.html) | XM_005681861.3, XM_018045824.1, XM_013964768.2, XM_018039549.1, XM_005679127.2, XM_013971303.1, XM_018063971.1, XM_018040583.1, XM_018063035.1, XM_018055911.1, XM_018058967.1, XM_005697610.3, XM_018055187.1, XR_001918100.1, XM_018050987.1, XM_005680737.3, XM_005685130.3, XM_018060378.1, XM_018051619.1, XM_005675300.3, XM_018060016.1, XM_018048959.1, XM_018062407.1, XM_018062408.1, XM_018058962.1, XM_018053228.1, XM_018042428.1, XM_013974872.2, XM_018047231.1, XM_018051036.1, XM_005692117.3, XM_018060496.1, XM_018062410.1, XM_018054208.1, XM_013968554.2, XM_018051622.1, XM_018058447.1, XM_005684782.3, XM_018060376.1, XM_005681863.3, XM_005679129.3, XM_018054206.1, XM_005692115.3, XM_018049689.1, XM_018040581.1, XM_018045830.1, XM_013968553.2, XM_005675289.3, XM_018048956.1, XM_018065817.1, XM_018059609.1, XM_013964769.2, XM_018042429.1, XM_018064745.1, XM_018063036.1, NM_001314202.1, XM_018060488.1, XM_013967227.2, XM_018048952.1, NM_001285750.1, XM_018040582.1, XM_018055908.1, XM_018049691.1, XM_018048555.1, XM_005675363.2, XM_018062409.1, XM_018058966.1, XM_018050856.1, XM_005685763.3, XM_018065816.1, XM_018055186.1, XM_018054083.1, XM_018060379.1, XM_018045131.1, XM_018045834.1, XM_018058585.1, XM_018062097.1, XM_018058968.1, XM_013970271.2, XM_013968877.2, XM_005675332.3, XM_005685146.3, XM_018064672.1, XM_005681862.2, XM_005688564.3, XM_018060261.1, XM_018048951.1, XM_018044014.1, XM_018064671.1, XM_013964774.2, XM_018058965.1, XM_018064746.1, XM_018048420.1, XM_018060377.1, XM_018057613.1, XM_018038791.1, XM_018058970.1, XM_005694472.3, XM_013964770.2, XM_018047547.1, XM_018055912.1, XM_018067051.1, XM_018051038.1, XM_018064292.1, XM_018038788.1, XM_018053231.1, XM_018044013.1, XM_005685145.3, XM_018053232.1 |
| 102 | [Measles](../../../AppData/Local/Temp/Temp1_VETR-D-18-00038.zip/Cont-vs-PPRV_DEGseq_map/map05162.html) | XM_018043204.1, XM_018066843.1, XM_013968498.2, XM_005679127.2, XM_018052626.1, XM_018063971.1, XM_018038998.1, XM_018063035.1, XM_018055911.1, XM_018047978.1, XM_018040157.1, XM_005697610.3, NM_001314233.1, XM_018055187.1, XM_013966112.2, XR_311047.3, XM_013972144.2, XM_005680058.3, XM_018060378.1, XM_018054468.1, XM_018047900.1, NM_001314206.1, XM_018055124.1, XM_018052627.1, XM_018047426.1, XM_005680266.3, XM_018060016.1, XM_018060051.1, XM_013973350.2, XM_018060045.1, XM_018062407.1, XM_018060727.1, XM_018062408.1, XM_005679901.2, XM_018053228.1, XM_018057132.1, XM_005682995.3, XM_005685995.3, XM_013974872.2, XM_005695611.3, XM_018047231.1, XM_018051036.1, XM_005684323.3, XM_005680937.3, XM_018043092.1, XM_018050945.1, NM_001314303.1, XM_005679654.2, XM_018051352.1, XM_005676377.3, XM_013976502.2, XM_018062410.1, XM_018040509.1, XM_013968554.2, XM_018058447.1, XM_005684782.3, XM_013963116.2, XM_018060376.1, XM_018066563.1, XM_005679129.3, XM_018063773.1, XM_013968553.2, XM_018044844.1, XM_005675289.3, XM_018065817.1, XM_018059609.1, XM_018041929.1, XM_018060043.1, XM_018047425.1, XM_018063036.1, XM_018060488.1, XM_018043089.1, XR_001296084.2, XM_013967227.2, XM_018050555.1, NM_001285750.1, XM_013966480.2, XM_018055349.1, XM_018055908.1, XM_018060052.1, XM_013972145.2, XM_018062409.1, XM_018040510.1, XM_018052624.1, XM_005674982.3, XM_018044609.1, XM_018065816.1, XM_018055186.1, XM_018054083.1, XM_018050706.1, XM_018060379.1, XM_018045131.1, XM_005677146.3, XM_013963900.1, XM_005679294.1, XM_018064130.1, XM_005680373.3, XM_018058634.1, XM_013970271.2, XM_018060042.1, XM_005684675.3, XM_013968877.2, XM_005675332.3, XM_005688564.3, XR_001296083.2, XM_018060261.1, XM_005689565.3, XM_005695610.2, XM_018048420.1, XM_018060377.1, NM_001285703.1, NM_001285608.1, XM_005679295.1, XM_018047738.1, XM_018055912.1, XM_018047444.1, XM_018043664.1, XM_005687533.3, XM_018051703.1, XM_018060044.1, XM_005674769.3, XM_018067051.1, XM_018047424.1, XM_018057840.1, XM_018051038.1, XM_018048550.1, XM_005676378.3, XM_018054172.1, XM_005688087.3, XM_018053231.1, XM_018043122.1, XM_018060728.1, XM_018054469.1, XM_018043091.1, XM_018038614.1, XM_013968497.2, XM_018053232.1 |
| 103 | [Apoptosis](../../../AppData/Local/Temp/Temp1_VETR-D-18-00038.zip/Cont-vs-PPRV_DEGseq_map/map04210.html) | XR_310868.3, XM_005681861.3, XM_005677656.3, NM_001314266.1, NM_001314245.1, XM_018038297.1, XM_013964768.2, XM_005679127.2, XR_001917015.1, XM_013967480.2, XM_005682490.2, XM_018063971.1, XM_018063035.1, XM_018055911.1, XM_005697610.3, XM_018055187.1, XR_001918100.1, XR_001917012.1, XM_018050987.1, XM_005680737.3, XM_005685130.3, XM_018060378.1, XM_018051619.1, XM_018051778.1, XM_018060016.1, XM_018044635.1, XM_018057419.1, XM_018048959.1, XM_018062407.1, XM_018062408.1, XM_018053228.1, XM_018042428.1, XM_018044831.1, XM_005685995.3, NM_001314352.1, XM_018065355.1, XM_013974872.2, XM_018047231.1, XM_018051782.1, XM_005676582.3, XM_018057425.1, XM_018059833.1, XM_018044832.1, XM_018059034.1, XM_018062410.1, XM_013968554.2, XM_018051622.1, XM_018058447.1, XM_005684782.3, XM_018060376.1, XM_005681863.3, XM_005679129.3, XM_018054206.1, XM_018049689.1, XM_013968553.2, XM_005675289.3, XM_018048956.1, XM_018065817.1, XM_018059609.1, XR_001917013.1, XM_013964769.2, XM_018042429.1, XM_018064745.1, XM_018049793.1, XR_001917017.1, XM_018051785.1, XM_018063036.1, NM_001314202.1, XR_001917959.1, XM_018060488.1, XM_013967227.2, XM_018048952.1, NM_001285750.1, XM_018055908.1, XM_018049691.1, XM_018051784.1, XM_018062409.1, XM_018050856.1, XM_005685763.3, XR_001296591.2, XM_005684278.2, XM_018065816.1, XM_018055186.1, XM_018054083.1, XM_018047843.1, XM_018060379.1, XM_018058634.1, XM_013970271.2, XM_018040360.1, XM_013968877.2, XM_005675332.3, XM_005685146.3, XM_018064672.1, XM_005681862.2, XM_018057412.1, XM_005688564.3, XM_018060261.1, XM_018048951.1, NM_001314163.1, XM_018064671.1, XM_013964774.2, XM_018064746.1, XR_001917014.1, XM_018048420.1, XM_018060377.1, XM_018038791.1, XM_005694472.3, XM_005699902.3, XM_013964770.2, XM_018051783.1, XM_018055912.1, XM_018051781.1, XM_018067051.1, XM_018059835.1, XM_018064292.1, XM_018038788.1, XM_018061204.1, XM_018053231.1, XM_018059836.1, XM_005685145.3, XM_018059834.1, XM_018053232.1 |
| 104 | [RNA polymerase](../../../AppData/Local/Temp/Temp1_VETR-D-18-00038.zip/Cont-vs-PPRV_DEGseq_map/map03020.html) | XM_018040731.1, XM_018049587.1, XM_018043802.1, XM_018048701.1, XM_018066694.1, XM_018055368.1, XM_018044847.1, XM_018041491.1, XR_001917204.1, XM_018063638.1, XM_005689869.3, XM_018063307.1, XR_001917205.1, XM_018063034.1, XM_018048699.1, XM_018040861.1, XM_018064666.1, XM_018062478.1, XM_005696338.3, XM_005686659.2, XM_018063860.1, XM_013964929.2, XM_018051498.1, XM_005677745.3, XM_018058552.1, XM_018048566.1, XM_018053696.1, XM_018063955.1, XM_018058588.1, XM_018046659.1, XM_005680575.3, XM_018042531.1, XM_018039932.1, XM_018064747.1, XR_001918377.1, XM_018064677.1, XM_013972522.2, XM_018048567.1, XM_005677744.3, XR_001917206.1, XM_018048694.1, XR_001917207.1, XM_018043805.1, XM_018061752.1, XM_013963623.2, XM_018066540.1, XM_018062155.1, XM_018063309.1, XM_005675184.3, XM_018048565.1, XM_005696194.3, XM_018048564.1, XM_005675838.3, XM_018048692.1, XM_018063965.1, XM_018048568.1, XM_018048691.1, XM_018039929.1, XM_005681064.3, XM_013968589.2, XM_018041044.1, XM_005691599.3, XM_018048569.1, XM_018059198.1, XM_018046661.1, XM_018062158.1, XM_018066692.1, XM_018062152.1, XM_005682900.3, XM_018062151.1, XM_018064675.1, XM_005681111.3, XM_018057659.1, XR_001917201.1, XM_018066691.1, XM_005688283.3, XM_018048698.1, XM_018048786.1, XM_018062157.1, XM_013973909.2, XM_018040957.1, XR_001917202.1, XM_018039930.1, XR_001919786.1, XM_018063637.1, XM_018039224.1, XM_018058106.1, XM_018066695.1, XM_005681112.3, XM_018059696.1, XM_018066699.1, XR_001917199.1, XM_018063304.1, XM_018048693.1, XM_018059699.1, XM_018066690.1, XM_018058699.1, XR_001917203.1, XM_018059000.1, XM_018062156.1, XM_018063859.1, XM_018063306.1, XM_005682148.2, XM_013964923.2, XM_018065932.1, XM_018039931.1, XM_018063305.1, XM_018040721.1, XM_018063629.1, XM_005697804.3, XM_005675651.3, XM_018040722.1, XM_018064584.1, XM_018064678.1, XM_018066698.1, XR_001917200.1, XM_018060818.1, XM_005697163.3, XM_018066696.1, XM_005688075.3, XM_018040960.1, XM_018051703.1, XM_018041045.1, XM_018050470.1, XM_005684036.3, XM_018045638.1, XM_018040862.1, XM_018057566.1, XM_018053643.1, XM_018063958.1, XM_018062154.1, XM_018051443.1, XM_018048695.1, XM_005682215.2, XM_018048563.1, XM_018041696.1, XM_018066693.1, XM_018048700.1, XM_018066697.1, XM_005679024.3, XM_018063954.1 |
| 105 | [Collecting duct acid secretion](../../../AppData/Local/Temp/Temp1_VETR-D-18-00038.zip/Cont-vs-PPRV_DEGseq_map/map04966.html) | XM_005687003.3, XM_018042922.1, XM_005674937.3, XM_018040923.1, XM_018062023.1, XM_018065122.1, XM_018055656.1, XM_018055657.1, XM_018055658.1, XM_018058330.1, XM_005687004.3, XM_005683966.3, XM_018065120.1, XM_005686382.3, XM_005684344.3, XM_013972159.2, XM_018061368.1, XM_018065121.1, XM_013972160.2, XM_018065637.1, XM_018065636.1, XM_018062175.1, XM_005674936.3, XM_005679452.3, XM_005689266.3, XM_018065123.1, XM_018045881.1, XM_005685994.3, XR_001918846.1 |
| 106 | [AGE-RAGE signaling pathway in diabetic complications](../../../AppData/Local/Temp/Temp1_VETR-D-18-00038.zip/Cont-vs-PPRV_DEGseq_map/map04933.html) | XM_005678936.3, XM_018038312.1, XM_018051874.1, XM_018059140.1, XM_005676250.3, XM_005681861.3, XM_018050499.1, XM_018059141.1, XM_018040552.1, XM_018054194.1, XM_018061294.1, XM_018052232.1, XM_018038313.1, XM_018052664.1, XM_013964768.2, XM_005679127.2, XM_018058815.1, XM_018048123.1, XM_018040741.1, XM_018065644.1, XM_018066833.1, XM_018063971.1, XM_018061293.1, XM_005679717.3, XM_018063035.1, XM_018055911.1, XM_018047033.1, XM_018065643.1, XM_005676159.3, XM_005697610.3, XM_018043415.1, XM_018055187.1, XM_018054452.1, XM_018058015.1, XM_018059139.1, XM_018045511.1, XM_005674830.2, XM_018061289.1, XM_018066008.1, XM_018039417.1, XM_005680115.3, XM_018060378.1, XM_018051619.1, XM_018056679.1, XM_018044169.1, XM_005700226.2, XM_018057651.1, XR_001918237.1, XM_018054454.1, XM_018049795.1, XM_005693225.3, XM_018060016.1, XM_018050883.1, XM_018039422.1, XM_018039601.1, XM_018043534.1, XM_018066005.1, XM_018061295.1, XM_018048959.1, XM_018038480.1, XM_018062699.1, XM_018048614.1, XM_018062407.1, XM_018056162.1, XM_018062408.1, XM_018039411.1, XM_018048124.1, XM_018041048.1, XM_005681905.3, XM_005699260.3, XM_018053228.1, XM_005680805.2, XM_018056363.1, XM_018042428.1, XM_018064893.1, XM_005683247.3, XM_018040727.1, XM_018041576.1, XM_018066832.1, XM_018066842.1, XM_005688053.3, XM_018038427.1, XM_018065259.1, XM_013973574.2, XM_013974872.2, XM_018039401.1, XM_018051480.1, XM_018045032.1, XM_018038561.1, XM_018047231.1, XM_018051036.1, XM_018050567.1, XM_018052213.1, XM_018045691.1, XM_018042282.1, XM_018058140.1, XM_018044065.1, NM_001287573.1, XM_005682449.3, XM_005699064.3, XM_005693227.3, XM_005709386.3, XM_013964307.2, NM_001286443.1, XM_018061292.1, XM_005681906.3, XM_018056916.1, XM_018038558.1, XM_018063551.1, XM_005699065.3, XM_018057646.1, XM_018042470.1, XM_013973573.2, XM_018040698.1, XM_018056914.1, XM_018045287.1, XM_018067083.1, XM_018039916.1, XM_005695833.3, XM_018052661.1, XM_018062410.1, XM_013963034.2, XM_018058129.1, XM_005676883.3, XM_013968554.2, XM_018051622.1, XM_005700661.3, XM_018058849.1, XM_018058447.1, XM_005684782.3, XM_018040436.1, XM_018060376.1, XM_018042537.1, XM_018067084.1, XM_018060566.1, XM_018043408.1, XM_005681863.3, XM_018040539.1, XM_005679129.3, XM_005687578.3, XM_018054206.1, XM_018042281.1, XM_005700662.3, XM_005675868.3, XM_018049689.1, XM_018046554.1, XM_005675709.3, XM_018066641.1, XM_005691412.3, XM_013968553.2, XM_018066481.1, XM_005675289.3, XM_018048956.1, XM_018065817.1, XM_013968410.2, XR_001919179.1, XM_018065642.1, XM_018051577.1, XM_018056913.1, XR_001919508.1, XM_018047841.1, XM_018066007.1, XM_018059609.1, XM_018042789.1, XM_018057521.1, XM_018040536.1, XM_018052663.1, XM_018052870.1, XM_013964769.2, XM_018042429.1, XM_018066002.1, XM_018061035.1, XM_005675869.3, XM_018053742.1, XM_018064745.1, XM_018038426.1, XM_018052662.1, XM_018040537.1, XM_018061288.1, XM_018052932.1, XM_018052867.1, XM_018057501.1, XM_018052570.1, XM_018066346.1, XM_018065480.1, XM_018063036.1, XM_018063966.1, XM_018054749.1, XM_005681903.3, XM_018058855.1, NM_001314202.1, XM_018058975.1, XM_018060488.1, XM_018040553.1, XM_018041370.1, XM_018052324.1, XM_018042279.1, XM_018040538.1, XM_013967227.2, XM_018048952.1, XM_018043409.1, NM_001285750.1, XM_018065262.1, XM_018042765.1, XM_018057881.1, XM_018043492.1, XM_018042534.1, XM_018058143.1, XM_005681533.3, XM_018049881.1, XM_013972156.2, XM_018055908.1, XM_018049691.1, XM_018062526.1, XM_005675149.3, XM_005689554.3, XM_018066004.1, XM_018062409.1, XM_018056910.1, XM_018039848.1, XM_005676887.3, XM_018042278.1, XM_018038559.1, XM_005685763.3, XM_005693222.3, XM_018063471.1, XM_018054455.1, XM_018052201.1, XM_018042567.1, XM_018041569.1, XM_005677636.3, XM_018058814.1, XR_001919509.1, XM_018048127.1, XM_018065816.1, XM_005700225.3, XM_018059142.1, XM_005693223.3, XM_018055186.1, XR_001918483.1, XM_018054083.1, XM_018045336.1, XM_018056908.1, XM_018042280.1, XM_018062538.1, XM_018054196.1, XM_018058852.1, XM_018054420.1, XM_018060379.1, XM_018045131.1, XM_018044444.1, XM_018042536.1, XM_018042468.1, XM_018056198.1, XM_018061765.1, XM_018058141.1, XM_005680676.3, XM_018066003.1, XM_005676249.3, XM_018048125.1, XM_018052331.1, XM_013974500.2, XM_018057160.1, XM_018049880.1, XM_018044439.1, XM_018056911.1, XM_018038560.1, XM_013970271.2, XM_018054421.1, XM_018043407.1, XM_018042467.1, XM_018057649.1, XM_018050882.1, XM_013968877.2, XM_018052941.1, XM_005675332.3, XM_018050307.1, XM_018063086.1, XM_005681862.2, XM_005688564.3, XM_005676315.3, XM_018059661.1, XM_018060261.1, XR_001919756.1, XM_018048951.1, XM_005678025.3, XM_018066009.1, XM_013975194.2, XM_018066507.1, XM_018056364.1, XM_013968280.2, XM_018040712.1, XM_018047031.1, XM_018048620.1, XM_018043207.1, XM_018059663.1, XM_018042971.1, XM_018041369.1, XM_013964774.2, XM_018064746.1, XM_018057647.1, XM_018044170.1, XM_018056863.1, XM_018048420.1, XM_018061291.1, XM_018060377.1, XM_018038791.1, XM_018057493.1, XM_018047868.1, XM_018042469.1, XM_018043357.1, XM_018042471.1, XM_018043763.1, XM_018061896.1, XM_018065433.1, XR_001918577.1, XM_018062527.1, XM_018066006.1, XM_018061286.1, XM_018054453.1, XR_001918843.1, XM_018050008.1, XM_013964770.2, XM_018040719.1, XM_005694346.3, XM_005701390.3, XM_013962289.2, XM_018051009.1, XM_018053042.1, XM_018048609.1, XM_018059626.1, XM_018061369.1, XM_018055912.1, XM_018038562.1, XM_018065646.1, XM_018056543.1, XM_005682575.3, XM_018052339.1, XM_018039344.1, NM_001285685.1, XM_018064895.1, XM_013966975.2, XM_018059143.1, XM_018067051.1, XM_018052868.1, XM_018058603.1, XM_018047840.1, XM_005680013.2, XM_005678961.3, XM_018043414.1, XM_018051038.1, XM_018054726.1, XM_018064292.1, XM_018041567.1, XM_018057650.1, XM_018065261.1, XM_018054580.1, XM_005681904.3, XM_018038788.1, XM_018055703.1, XM_018053231.1, XM_018056915.1, XM_005685953.3, XM_018051010.1, XM_018054431.1, XM_018050766.1, XM_018050765.1, XM_005695832.3, NM_001314200.1, XM_018044440.1, XM_018043757.1, XM_018052573.1, XM_005684543.2, XM_018040704.1, XM_018055450.1, XM_013962679.2, XM_018051715.1, XM_018053232.1 |
| 107 | [GnRH signaling pathway](../../../AppData/Local/Temp/Temp1_VETR-D-18-00038.zip/Cont-vs-PPRV_DEGseq_map/map04912.html) | XM_018062643.1, XM_013963001.2, XM_005681861.3, XM_018061124.1, NM_001285765.1, XM_018045824.1, XM_013964768.2, XM_005680366.2, XM_018047786.1, XM_018054045.1, XM_018053440.1, XM_018058967.1, XM_018054409.1, XM_018065799.1, XR_001918100.1, XM_018050987.1, XM_005680737.3, XM_005685130.3, XM_005697075.3, XM_005691447.3, XM_018066370.1, XM_018051619.1, XM_013972607.2, XM_005680364.3, XM_005699952.3, XM_018048959.1, XM_018058962.1, XM_018042428.1, XM_018065623.1, XM_018047802.1, XR_001918038.1, XM_018061125.1, XM_005680365.2, XM_018054401.1, XM_005690990.2, XM_018060732.1, XM_018044486.1, XM_018039561.1, XM_018051622.1, XM_005688518.3, XM_018041016.1, XM_013964590.2, XM_005681863.3, XM_005694690.3, XM_018054206.1, XM_018043047.1, XM_018049689.1, XM_018045830.1, XM_018048956.1, XM_005679333.3, XM_018054405.1, XM_013964769.2, XM_018042429.1, XM_018064745.1, XM_018054406.1, XM_018066371.1, XM_018057813.1, NM_001314202.1, XM_018048952.1, XM_013970799.2, XM_018053442.1, XM_018049691.1, XM_018043593.1, XR_001295516.2, XM_018058966.1, XM_018050856.1, XM_005685763.3, XM_005680367.3, XM_005676485.3, XM_005685150.3, XM_018054402.1, XM_005686574.3, XM_018054408.1, XM_013966779.2, XM_018045834.1, XM_018058968.1, XM_005684842.3, XM_018054403.1, XM_005685146.3, XM_018064672.1, XM_005681862.2, XM_018048951.1, XM_005685526.3, XR_001296033.2, XM_018064671.1, XM_013964774.2, XM_018058965.1, XM_018064746.1, XM_018064499.1, XM_018038791.1, XM_018058970.1, XM_005694472.3, XM_018053441.1, XM_018054044.1, XM_013964770.2, XM_005676482.3, XM_018054407.1, XM_005685149.3, XM_018065266.1, XM_005697074.3, XM_018064292.1, XM_018038788.1, XM_013976047.2, XM_005685145.3, XM_005694349.2, XM_005699951.1 |
| 108 | [Type II diabetes mellitus](../../../AppData/Local/Temp/Temp1_VETR-D-18-00038.zip/Cont-vs-PPRV_DEGseq_map/map04930.html) | XM_005681861.3, XM_013964768.2, XM_005679127.2, NM_001285748.1, XM_018060378.1, XM_018051619.1, XM_018060016.1, XM_018048959.1, XM_018042428.1, XM_018047231.1, XM_018042596.1, XM_018042597.1, XM_018042326.1, XM_018051622.1, XM_018060376.1, XM_005681863.3, XM_005679129.3, XM_018054206.1, XM_018049689.1, XM_005675289.3, XM_018048956.1, XM_005685177.3, XM_018059609.1, XM_013964769.2, XM_018042429.1, XM_018064745.1, NM_001314202.1, XM_018048952.1, XM_018049691.1, XM_018054976.1, XM_018059919.1, XM_005685763.3, XM_018060379.1, XM_005681862.2, XM_018048951.1, XM_013964774.2, XM_018064746.1, XM_018048420.1, XM_018060377.1, XM_018038791.1, XM_013964770.2, XM_018059918.1, XM_018064292.1, XM_018038788.1, XM_018050632.1, XM_013976777.2 |
[truncated: 378,213 more chars]
